# The wild bees (Hymenoptera, Apoidea, Anthophila) of the urban nature reserves of Rome (Italy, Latium): a preliminary survey

**DOI:** 10.3897/BDJ.12.e139087

**Published:** 2024-12-27

**Authors:** Lorenzo Fortini, Enrico Ruzzier, Maurizio Mei, Andrea Di Giulio

**Affiliations:** 1 Department of Science, Roma Tre University, Viale G. Marconi, 446, Rome, Italy Department of Science, Roma Tre University, Viale G. Marconi, 446 Rome Italy; 2 National Biodiversity Future Center (NBFC), Palermo, Italy National Biodiversity Future Center (NBFC) Palermo Italy; 3 Department of Biology and Biotechnology “Charles Darwin”, Sapienza University of Rome, Rome, Italy Department of Biology and Biotechnology “Charles Darwin”, Sapienza University of Rome Rome Italy

**Keywords:** biodiversity, conservation, faunistics, urban fauna, wild bees

## Abstract

**Background:**

Urbanisation is a global phenomenon responsible for negative processes in natural ecosystems, such as degradation, loss of habitat and fragmentation. Large urban green areas could, however, represent shelter for animal species, promoting biodiversity conservation. Urban green spaces can also provide useful habitats for threatened species. Wild bees are amongst the most important and efficient pollinating insects and play an indispensable role in ecosystem functioning. Approximately half of the European wild bee species have been reported from Italy, making it a biodiversity hotspot for this group of insects. Many studies have been conducted on pollinating insects in recent years, but few data and incomplete knowledge on wild bee faunas in strictly urban environments exist. Georeferenced faunal data would be important for conservation efforts and understanding the effects that large cities such as Rome may have on the diversity of wild bee communities.

**New information:**

This work focused on the diversity of wild bees in nature reserves belonging to the RomaNatura network within the urban area of Rome, Italy. A total of 208 wild bee species belonging to 36 genera and six families were identified. Amongst the species surveyed, one species was classified as Endangered (EN) and seven species were classified as Near Threatened (NT) by the European Red List of wild bees. Twenty-four species are new reports for the urban area of Rome. Sampling took place from April to September 2022. The most represented family in terms of abundance was Halictidae, accounting for 36% of all bees collected, followed by Apidae (24% of samples), Andrenidae (17% of samples), Megachilidae (15% of samples), Colletidae (7% of samples) and Melittidae (1% of samples). The Megachilidae family was the richest in terms of the number of species, accounting for 25% of the total species sampled. This research aimed to provide a set of standardised and georeferenced wild bee occurrences that constitute the baseline for any faunistic, ecological and conservation activity of Rome's nature reserves. In addition, the research aims to demonstrate the importance of large urban green areas in one of the largest European cities as biodiversity reservoirs for wild bees.

## Introduction

**Figure 1.**
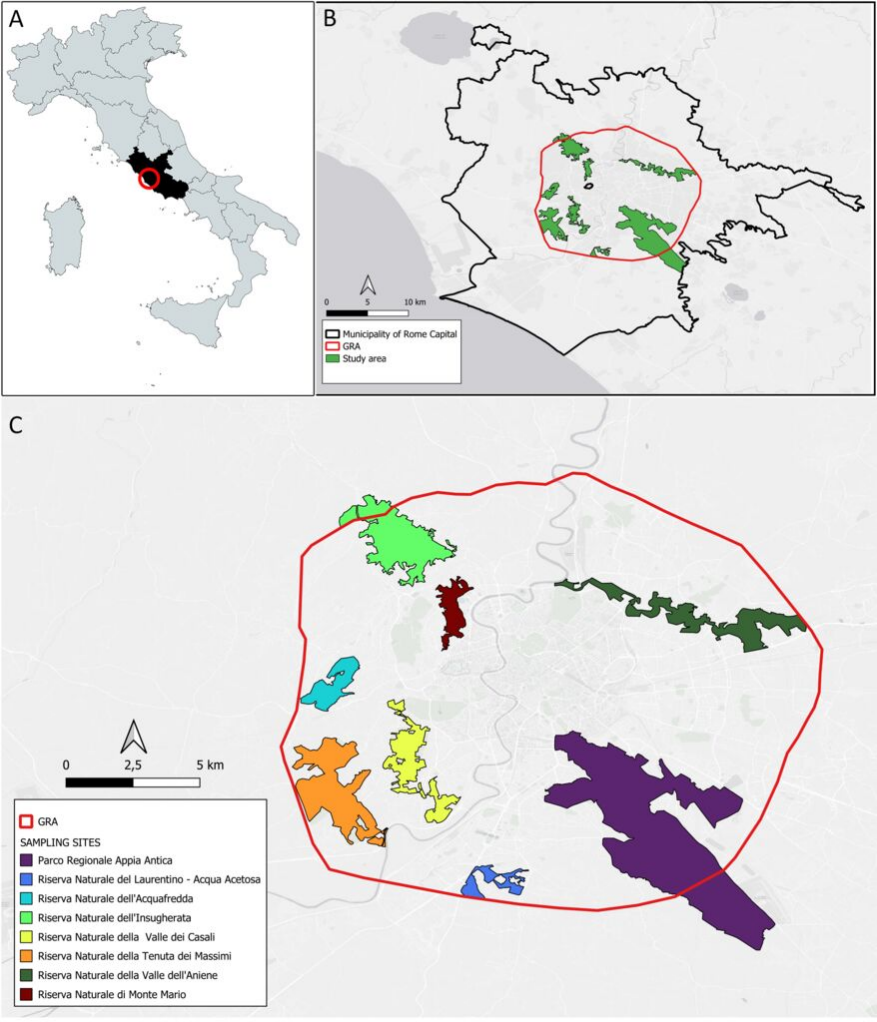
Study area. **A** Location of Rome Municipality (red circle) within the Italian territory. The Latium Region in black; **B** Location of the study area (in green) within the Rome Municipality (black line). GRA is the Grande Raccordo Anulare, a motorway conventionally used to define the urban area of Rome (red line); **C** Nature Reserves within the urban area of Rome belonging to the RomaNatura network.

The urbanisation process represents one of the main threats to biodiversity (Connor et al. 2002, Matteson and Langellotto 2011, Vanbergen and the Insect Pollinators Initiative 2013), causing fragmentation, modification and loss of habitats (Díaz et al. 2006, McKinney 2008, Aguilera et al. 2018) and consequently resulting in richness and abundance loss (Baldock et al. 2015, Fenoglio et al. 2021). However, studies quantifying the effects of urbanisation on biodiversity seem to provide conflicting results. Recent works have shown that the urban environment, particularly urban green areas, can even have positive effects on the conservation of biodiversity compared to other ecosystems strongly impacted by anthropogenic activities, such as areas subject to intensive agriculture (Öckinger et al. 2009, Nielsen et al. 2013, Banaszak-Cibicka et al. 2016, Dyderski et al. 2016, Hall et al. 2017, Ciach and Fröhlich 2019, Dylewski et al. 2019). Not all species are adversely affected by urbanisation and urban areas can provide unique opportunities for conservation (Magura et al. 2010, Moorhead and Philpott 2013, Fattorini et al. 2017, Fattorini et al. 2020, Wenzel et al. 2020, Francoeur et al. 2021). For example, the positive effects on pollinator biodiversity may be the result of biotic factors, such as a greater diversity of plant species in urbanised areas than in rural areas due to the presence of exotic and ornamental plants that make trophic resources available for a longer period during the year and increase the diversity of habitats and microclimates (Zaninotto et al. 2023). Another possible factor can be the degradation in the quality of habitats in rural areas (Wenzel et al. 2020). Abiotic factors can also have important effects. The 'heat island effect' produces higher air temperatures and more stable climatic conditions (Kuttler 2008) in cities than in surrounding areas (Oke 1973), affecting insect phenology and survival (Chick et al. 2019). For this reason, urban green spaces can represent important locations for the conservation of several animal groups of vertebrates and invertebrates (Forman 1995, Angold et al. 2006, Jones and Leather 2012, Fattorini and Galassi 2016), particularly for insect pollinators such as bees. Despite the considerable efforts made in recent years through studies and investments, there is still a lack of knowledge regarding insect urban diversity and ecology. In particular, few studies have focused on pollinators in cities. Italy is a biodiversity hotspot for wild bees (Orr et al. 2021). Focusing on the most important and complete papers published on Apoidea biodiversity in Italy in recent decades, 1173 species were reported in Comba (2019). Lhomme et al. (2020) reduced the number to 1028 species using data provided by Ascher and Pickering (2020) and showing that Italy has the eleventh bee richness in the Mediterranean Basin. Wood et al. (2023), while revising Italian *Andrena*, subsequently added nine more species to those previously listed, confirming that Italy hosts one of the largest Andrena faunas in the world. This addition resulted from the revision of eight species and the description of a new one. Cornalba et al. (2024) recently provided eight new faunistic records for the Italian fauna, taking the number of species to 1045. The diversity of Hymenoptera Apoidea inhabiting Rome was previously discussed in Comba and Comba (1991), in which 450 species were reported for the Latium Region (Fig. 1A) and, in Comba and Comba (1997), 378 wild bee species were recorded within the Grande Raccordo Anulare (GRA; see Materials and Methods). Despite the large number of species listed in these works, unpublished data and specimens housed in the Maurizio Mei collection (Museum of the Institute of Zoology, Sapienza University, Rome, Italy) suggest that the number of species present in the urban area of Rome might exceed 400. One of the most recent papers on this topic is that of Geppert et al. (2022), who studied the functional traits and ecology of wild bees in Rome and provided a list of bee species. The objective of this study is to provide precise and original georeferenced faunistic data on the Apoidea Anthophila inhabiting the urban natural reserves of Rome, making them immediately available in the standard Darwin-core format to facilitate their use in future scientific work focused on the biodiversity of pollinating insects in urban areas. In addition, these data constitute an integration and update of the previously available information, thus setting the new faunistic reference to be used as a starting base. Producing this type of data for a city such as Rome, which is amongst the largest and most urbanised in Europe, is an important starting point for understanding how urbanisation defines and might affect animal biodiversity. Providing precise information on Apoidea distributions and insects in general in large urban green areas is essential for implementing plans to protect and enhance the positive effects on the diversity and abundance of pollinator communities. This type of data and information can also be used to provide guidelines for urban green management actions aimed at encouraging the presence of pollinating insects in the urban environment.

## Materials and methods

### Study area and sites

Rome is one of the largest cities in Europe and the third most populated city in the European Union, with a population of 2,873,494 (Demo Istat 2017). The urban area is conventionally identified with the region delimited by the motorway ring known as the Grande Raccordo Anulare (GRA), with a length of approximately 360 km^2^ (Fattorini 2011, Di Pietro et al. 2021). All nature reserves sampled in this study are located within the boundaries delimited by the freeway that surrounds the more densely urbanised area of the city (ca. 360 km^2^). Sampling took place from April to September 2022 in eight large green areas of the city of Rome, Italy (41.903611, 12.488611) (Fig. 1). Eight nature reserves were selected for sampling, with an extension ranging from 152 hectares (Riserva Naturale Laurentino-Acqua Acetosa) to 4580 hectares (Parco Regionale dell'Appia Antica). Seven nature reserves are part of the RomaNatura network. In addition to these seven areas, we included the Parco Regionale dell'Appia Antica, which is the largest urban green area in Europe and is located southeast of the city.

In the following is reported the list of nature reserves considered in this study, the relative abbreviations, the coordinate of the centroids reported in decimal degrees, the extent and the number of fixed transects performed in each reserve for session:

- Parco Regionale dell'Appia Antica (APP) (41.864167, 12.515833) (4580 hectares/3 transects);
- Riserva Naturale dell'Insugherata (INS) (41.961944, 12.427222) (697 hectares/3 transects);
- Riserva Naturale Valle dei Casali (VDC) (41.867778, 12.432222) (466 hectares/2 transects);
- Riserva Naturale Valle dell’Aniene (VAN) (41.929055, 12.556024) (650 hectares/2 transects);
- Riserva Naturale Tenuta dei Massimi (TDM) (41.846667, 12.400000) (774 hectares/2 transects);
- Riserva Naturale di Monte Mario (MMA) (41.922500, 12.452500) (204 hectares/1 transect);
- Riserva Naturale Tenuta dell'Acquafredda (ACQ) (41.892222, 12.398056) (249 hectares/1 transect);
- Riserva Naturale Laurentino-Acqua Acetosa (LAU) (41.805556, 12.477778) (152 hectares/1 transect).

### Sampling method

Sampling was carried out along 250-metre-long fixed transects (the transects remained the same in length and position throughout the study) and was repeated once a month, from April to September, in all eight nature reserves selected for the study. The number of transects performed in each site depended on the size of each nature reserve. In total, we performed 15 fixed transects, one each 250 hectares of extension, for a maximum of three transects per nature reserve. The transect locations were chosen where the vegetation was representative of the area and at least 100 metres apart. In three out of the eight sites, this ratio was not mathematically respected due to some specific characteristics of the area and limits to accessibility. The total extension of TDM is 774 hectares, but we performed two linear transects, due to the ban on access to a large archaeological private area. In VAN, we performed two transects because the park has elongated and narrow shapes that extend to the sides of a part of the Aniene River. It increased greatly the ratio between the perimeter and the surface of the area and meant that a large part of the park is occupied by the watercourse. In addition, a relevant percentage of the remaining walkable surface is made up of roads to move inside the park reducing the areas where a transect could be performed. APP is a huge urban green area. It extends from the centre of the city to the outskirts and presents, within it, a mosaic of inhabited, grazing, recreational and archaeological areas, but also integral reserves, accessible to citizens. Here, we carried out the transects in three areas, homogeneously distributed along the park. The investigations were conducted under sunny, low-wind weather conditions between 10:00 am and 3:00 pm. Wild bees were collected via an entomological net within 2 m on both sides of each sample line (Westphal et al. 2008, Schindler et al. 2013, Neumüller et al. 2020). Each transect was carried out by walking at a constant speed for 45 minutes. All the observations were conducted by the same person (LF) walking along the sample lines. All the samples were collected while foraging on flowers along the transects.

### Preparation and identification

All collected samples were stored in Falcon tubes, exposed to ethyl acetate and dry-pinned. Morphological identification was performed using reference literature on the group and taxonomic keys such as those provided by Michez et al. (2019) for genera identification, Amiet et al. (2007) for Apidae, Smith (2018) for *Nomada*, Amiet et al. (2001) and Pauly (2015) for Halictidae, Amiet et al. (2004) for Megachilidae, Dathe (1980) for *Hylaeus*, Amiet et al. (1999) for *Colletes*, Wood (2023) for *Andrena* and taking as reference the collection from the Museum of Zoology of La Sapienza University. The identifications have been validated by an expert taxonomist author (MM). The final list of species collected during the study is reported below. The checklist respects the alphabetical order and the latitude/longitude given for each specimen are those of the mid-point of the transect where it was collected. The taxonomic arrangement followed that of Ghisbain et al. (2023), Wood (2024) and Orr et al. (2024).

## Checklists

### Andrenidae Fabricius, 1775

#### Andrena aeneiventris

Morawitz, 1872

399B9BAB-682B-5C67-8A9F-83AC41D67830

##### Materials

- **Type status:** Other material. **Occurrence:** catalogNumber: A0871; recordedBy: L. Fortini; individualCount: 1; sex: female; lifeStage: adult; occurrenceID: 4C0406DB-3BEC-500F-880F-14C2E1AEB131; **Taxon:** scientificName: Andrena (Aenandrena) aeniventris Morawitz, 1872; order: Hymenoptera; family: Andrenidae; genus: Andrena; subgenus: Aenandrena; specificEpithet: aeniventris; scientificNameAuthorship: Morawitz, 1872; **Location:** country: Italy; countryCode: IT; stateProvince: Roma; locality: Riserva Naturale Laurentino-Acqua Acetosa; decimalLatitude: 41.8079275; decimalLongitude: 12.4685548; geodeticDatum: WGS84; coordinatePrecision: 0.0002; **Identification:** identifiedBy: M. Mei; **Event:** eventDate: 2022-06-16; **Record Level:** collectionCode: UR3

#### Andrena agilissima

(Scopoli, 1770)

D6D793C9-3127-5FAB-A737-1CBBD3CD4516

##### Materials

- **Type status:** Other material. **Occurrence:** catalogNumber: A1043, A1044; recordedBy: L. Fortini; individualCount: 2; sex: males; lifeStage: adult; occurrenceID: 7CB582D4-ECBC-5738-B1F6-AC2B6B240ACB; **Taxon:** scientificName: Andrena (Agandrena) agilissima (Scopoli, 1770); order: Hymenoptera; family: Andrenidae; genus: Andrena; subgenus: Agandrena; specificEpithet: agilissima; scientificNameAuthorship: (Scopoli, 1770); **Location:** country: Italy; countryCode: IT; stateProvince: Roma; locality: Riserva Naturale Valle dell'Aniene 2; decimalLatitude: 41.928752; decimalLongitude: 12.5562962; geodeticDatum: WGS84; coordinatePrecision: 0.0002; **Identification:** identifiedBy: M. Mei; **Event:** eventDate: 2022-06-05; **Record Level:** collectionCode: UR3

#### Andrena albopunctata

(Rossi, 1792)

6810E1D9-8DEF-584C-BC92-ECB81B6DBC66

##### Materials

- **Type status:** Other material. **Occurrence:** catalogNumber: A1045; recordedBy: L. Fortini; individualCount: 1; sex: female; lifeStage: adult; occurrenceID: 564C8012-E9E2-5D77-84A9-49A5879ECC9E; **Taxon:** scientificName: Andrena (Melandrena) albopunctata (Rossi, 1792); order: Hymenoptera; family: Andrenidae; genus: Andrena; subgenus: Melandrena; specificEpithet: albopunctata; scientificNameAuthorship: (Rossi, 1792); **Location:** country: Italy; countryCode: IT; stateProvince: Roma; locality: Riserva Naturale dell'Insugherata 2; decimalLatitude: 41.9599247; decimalLongitude: 12.433852; geodeticDatum: WGS84; coordinatePrecision: 0.0002; **Identification:** identifiedBy: M. Mei; **Event:** eventDate: 2022-06-24; **Record Level:** collectionCode: UR3

#### Andrena bisulcata

Morawitz, 1877

1219BF55-FD87-5C81-BBF4-F836A6DF5839

##### Materials

- **Type status:** Other material. **Occurrence:** catalogNumber: A1212, A1213; recordedBy: L. Fortini; individualCount: 2; sex: females; lifeStage: adult; occurrenceID: 6ECB3C0C-1C45-53E4-98AC-F687D620B4E7; **Taxon:** scientificName: Andrena (Aenandrena) bisulcata Morawitz, 1877; order: Hymenoptera; family: Andrenidae; genus: Andrena; subgenus: Aenandrena; specificEpithet: bisulcata; scientificNameAuthorship: Morawitz, 1877; **Location:** country: Italy; countryCode: IT; stateProvince: Roma; locality: Riserva Naturale Valle dell'Aniene 2; decimalLatitude: 41.928752; decimalLongitude: 12.5562962; geodeticDatum: WGS84; coordinatePrecision: 0.0002; **Identification:** identifiedBy: M. Mei; **Event:** eventDate: 2022-04-28; **Record Level:** collectionCode: UR3
- **Type status:** Other material. **Occurrence:** catalogNumber: A1217; recordedBy: L. Fortini; individualCount: 1; sex: female; lifeStage: adult; occurrenceID: 52DAD577-4BE2-5DB5-9F02-D74AD74CFBBF; **Taxon:** scientificName: Andrena (Aenandrena) bisulcata Morawitz, 1877; order: Hymenoptera; family: Andrenidae; genus: Andrena; subgenus: Aenandrena; specificEpithet: bisulcata; scientificNameAuthorship: Morawitz, 1877; **Location:** country: Italy; countryCode: IT; stateProvince: Roma; locality: Riserva Naturale dell'Insugherata 2; decimalLatitude: 41.9599247; decimalLongitude: 12.433852; geodeticDatum: WGS84; coordinatePrecision: 0.0002; **Identification:** identifiedBy: M. Mei; **Event:** eventDate: 2022-04-15; **Record Level:** collectionCode: UR3

#### Andrena braunsiana

Friese, 1887

6C3CEEE2-DE9E-5600-8159-6F2D9A24B1E2

##### Materials

- **Type status:** Other material. **Occurrence:** catalogNumber: A1964; recordedBy: L. Fortini; individualCount: 1; sex: male; lifeStage: adult; occurrenceID: B1AA5D07-7733-58D9-B7BA-E4B717E22E48; **Taxon:** scientificName: Andrena (Pallandrena) braunsiana Friese, 1887; order: Hymenoptera; family: Andrenidae; genus: Andrena; subgenus: Pallandrena; specificEpithet: braunsiana; scientificNameAuthorship: Friese, 1887; **Location:** country: Italy; countryCode: IT; stateProvince: Roma; locality: Riserva Naturale dell'Insugherata 3; decimalLatitude: 41.9644829; decimalLongitude: 12.436101; geodeticDatum: WGS84; coordinatePrecision: 0.0002; **Identification:** identifiedBy: M. Mei; **Event:** eventDate: 2022-04-15; **Record Level:** collectionCode: UR3

#### Andrena cinerea

Brullé, 1832

F21B401B-BF5D-52D7-9235-DA986AC6BF88

##### Materials

- **Type status:** Other material. **Occurrence:** catalogNumber: A0878, A0900; recordedBy: L. Fortini; individualCount: 2; sex: females; lifeStage: adult; occurrenceID: 9404E0B4-C86B-5954-B6FD-4DB99981EC3F; **Taxon:** scientificName: Andrena (Chlorandrena) cinerea Brullé, 1832; order: Hymenoptera; family: Andrenidae; genus: Andrena; subgenus: Chlorandrena; specificEpithet: cinerea; scientificNameAuthorship: Brullé, 1832; **Location:** country: Italy; countryCode: IT; stateProvince: Roma; locality: Riserva Naturale Tenuta dei Massimi 2; decimalLatitude: 41.8316516; decimalLongitude: 12.3999927; geodeticDatum: WGS84; coordinatePrecision: 0.0002; **Identification:** identifiedBy: M. Mei; **Event:** eventDate: 2022-05-04; **Record Level:** collectionCode: UR3
- **Type status:** Other material. **Occurrence:** catalogNumber: A1214; recordedBy: L. Fortini; individualCount: 1; sex: male; lifeStage: adult; occurrenceID: CCFAFC7A-C17D-5294-BEDC-FBF6764C5304; **Taxon:** scientificName: Andrena (Chlorandrena) cinerea Brullé, 1832; order: Hymenoptera; family: Andrenidae; genus: Andrena; subgenus: Chlorandrena; specificEpithet: cinerea; scientificNameAuthorship: Brullé, 1832; **Location:** country: Italy; countryCode: IT; stateProvince: Roma; locality: Riserva Naturale Valle dell'Aniene 2; decimalLatitude: 41.928752; decimalLongitude: 12.5562962; geodeticDatum: WGS84; coordinatePrecision: 0.0002; **Identification:** identifiedBy: M. Mei; **Event:** eventDate: 2022-04-28; **Record Level:** collectionCode: UR3
- **Type status:** Other material. **Occurrence:** catalogNumber: A0894; recordedBy: L. Fortini; individualCount: 1; sex: female; lifeStage: adult; occurrenceID: 8FCE1A74-E5A2-5D12-8F79-37113F51DA18; **Taxon:** scientificName: Andrena (Chlorandrena) cinerea Brullé, 1832; order: Hymenoptera; family: Andrenidae; genus: Andrena; subgenus: Chlorandrena; specificEpithet: cinerea; scientificNameAuthorship: Brullé, 1832; **Location:** country: Italy; countryCode: IT; stateProvince: Roma; locality: Riserva Naturale di Monte Mario; decimalLatitude: 41.9386215; decimalLongitude: 12.4546223; geodeticDatum: WGS84; coordinatePrecision: 0.0002; **Identification:** identifiedBy: M. Mei; **Event:** eventDate: 2022-05-20; **Record Level:** collectionCode: UR3

#### Andrena clypella

Strand, 1921

2B4A52F7-4CE6-578F-B8EC-2C0D9D92644C

##### Materials

- **Type status:** Other material. **Occurrence:** catalogNumber: A0994, A0995, A0996; recordedBy: L. Fortini; individualCount: 3; sex: males; lifeStage: adult; occurrenceID: EA4EAEEB-9E21-55F5-9032-64341B801FDF; **Taxon:** scientificName: Andrena (Chlorandrena) clypella Strand, 1921; order: Hymenoptera; family: Andrenidae; genus: Andrena; subgenus: Chlorandrena; specificEpithet: clypella; scientificNameAuthorship: Strand, 1921; **Location:** country: Italy; countryCode: IT; stateProvince: Roma; locality: Riserva Naturale Valle dei Casali 1; decimalLatitude: 41.8710627; decimalLongitude: 12.4336809; geodeticDatum: WGS84; coordinatePrecision: 0.0002; **Identification:** identifiedBy: M. Mei; **Event:** eventDate: 2022-04-07; **Record Level:** collectionCode: UR3
- **Type status:** Other material. **Occurrence:** catalogNumber: A0880; recordedBy: L. Fortini; individualCount: 1; sex: female; lifeStage: adult; occurrenceID: D8BD948E-5450-5F55-9D69-8D2253AE4A53; **Taxon:** scientificName: Andrena (Chlorandrena) clypella Strand, 1921; order: Hymenoptera; family: Andrenidae; genus: Andrena; subgenus: Chlorandrena; specificEpithet: clypella; scientificNameAuthorship: Strand, 1921; **Location:** country: Italy; countryCode: IT; stateProvince: Roma; locality: Riserva Naturale Valle dell'Aniene 2; decimalLatitude: 41.928752; decimalLongitude: 12.5562962; geodeticDatum: WGS84; coordinatePrecision: 0.0002; **Identification:** identifiedBy: M. Mei; **Event:** eventDate: 2022-06-05; **Record Level:** collectionCode: UR3
- **Type status:** Other material. **Occurrence:** catalogNumber: A0879, A0881; recordedBy: L. Fortini; individualCount: 2; sex: females; lifeStage: adult; occurrenceID: 8FA16DCC-BACB-503C-A49F-B047D56EC5B0; **Taxon:** scientificName: Andrena (Chlorandrena) clypella Strand, 1921; order: Hymenoptera; family: Andrenidae; genus: Andrena; subgenus: Chlorandrena; specificEpithet: clypella; scientificNameAuthorship: Strand, 1921; **Location:** country: Italy; countryCode: IT; stateProvince: Roma; locality: Riserva Naturale dell'Insugherata 3; decimalLatitude: 41.9644829; decimalLongitude: 12.436101; geodeticDatum: WGS84; coordinatePrecision: 0.0002; **Identification:** identifiedBy: M. Mei; **Event:** eventDate: 2022-06-24; **Record Level:** collectionCode: UR3
- **Type status:** Other material. **Occurrence:** catalogNumber: A0967, A0968; recordedBy: L. Fortini; individualCount: 2; sex: male; lifeStage: adult; occurrenceID: 99725ED6-4E59-5455-A9E5-864ACD0CF0C0; **Taxon:** scientificName: Andrena (Chlorandrena) clypella Strand, 1921; order: Hymenoptera; family: Andrenidae; genus: Andrena; subgenus: Chlorandrena; specificEpithet: clypella; scientificNameAuthorship: Strand, 1921; **Location:** country: Italy; countryCode: IT; stateProvince: Roma; locality: Riserva Naturale dell'Insugherata 3; decimalLatitude: 41.9644829; decimalLongitude: 12.436101; geodeticDatum: WGS84; coordinatePrecision: 0.0002; **Identification:** identifiedBy: M. Mei; **Event:** eventDate: 2022-06-24; **Record Level:** collectionCode: UR3

#### Andrena colletiformis

Morawitz, 1874

A4A0AAE1-1213-50DB-A548-8A69631059D7

##### Materials

- **Type status:** Other material. **Occurrence:** catalogNumber: A0956, A0957, A0958, A0959, A0960, A1641; recordedBy: L. Fortini; individualCount: 6; sex: males; lifeStage: adult; occurrenceID: A998AEEE-3606-571A-86A4-C831E1211A7B; **Taxon:** scientificName: Andrena (Brachyandrena) colletiformis Morawitz, 1874; order: Hymenoptera; family: Andrenidae; genus: Andrena; subgenus: Brachyandrena; specificEpithet: colletiformis; scientificNameAuthorship: Morawitz, 1874; **Location:** country: Italy; countryCode: IT; stateProvince: Roma; locality: Riserva Naturale Valle dei Casali 1; decimalLatitude: 41.8710627; decimalLongitude: 12.4336809; geodeticDatum: WGS84; coordinatePrecision: 0.0002; **Identification:** identifiedBy: M. Mei; **Event:** eventDate: 2022-07-13; **Record Level:** collectionCode: UR3

#### Andrena combaella

Warncke, 1966

66BBBF1C-D9FE-5FF9-8092-3D0EA25F8EE7

##### Materials

- **Type status:** Other material. **Occurrence:** catalogNumber: A1070, A1071; recordedBy: L. Fortini; individualCount: 2; sex: females; lifeStage: adult; occurrenceID: 06084046-56AC-5D93-B1AC-6CBD35BC3600; **Taxon:** scientificName: Andrena (Ulandrena) combaella Warncke, 1966; order: Hymenoptera; family: Andrenidae; genus: Andrena; subgenus: Ulandrena; specificEpithet: combaella; scientificNameAuthorship: Warncke, 1966; **Location:** country: Italy; countryCode: IT; stateProvince: Roma; locality: Riserva Regionale dell'Appia Antica 3; decimalLatitude: 41.8298456; decimalLongitude: 12.5432538; geodeticDatum: WGS84; coordinatePrecision: 0.0002; **Identification:** identifiedBy: M. Mei; **Event:** eventDate: 2022-05-24; **Record Level:** collectionCode: UR3

#### Andrena congruens

Schmiedeknecht, 1884

59A6A81E-C7AB-55B6-A70A-8F557679CABE

##### Materials

- **Type status:** Other material. **Occurrence:** catalogNumber: A0965; recordedBy: L. Fortini; individualCount: 1; sex: males; lifeStage: adult; occurrenceID: F354FD60-2344-52F7-95C7-CB14C60EDD98; **Taxon:** scientificName: Andrena (Simandrena) congruens Schmiedeknecht, 1884; order: Hymenoptera; family: Andrenidae; genus: Andrena; subgenus: Simandrena; specificEpithet: congruens; scientificNameAuthorship: Schmiedeknecht, 1884; **Location:** country: Italy; countryCode: IT; stateProvince: Roma; locality: Riserva Naturale di Monte Mario; decimalLatitude: 41.9386215; decimalLongitude: 12.4546223; geodeticDatum: WGS84; coordinatePrecision: 0.0002; **Identification:** identifiedBy: M. Mei; **Event:** eventDate: 2022-06-19; **Record Level:** collectionCode: UR3

#### Andrena decipiens

Schenck, 1861

DB70A8C1-BA0D-5772-A671-2848C8F91AD0

##### Materials

- **Type status:** Other material. **Occurrence:** catalogNumber: A1106, A1107; recordedBy: L. Fortini; individualCount: 2; sex: females; lifeStage: adult; occurrenceID: E03631F7-1346-59FE-AC31-79E7E4F90D6F; **Taxon:** scientificName: Andrena (Holandrena) decipiens Schenck, 1861; order: Hymenoptera; family: Andrenidae; genus: Andrena; subgenus: Holandrena; specificEpithet: decipiens; scientificNameAuthorship: Schenck, 1861; **Location:** country: Italy; countryCode: IT; stateProvince: Roma; locality: Riserva Naturale Tenuta dei Massimi 1; decimalLatitude: 41.8532859; decimalLongitude: 12.3842322; geodeticDatum: WGS84; coordinatePrecision: 0.0002; **Identification:** identifiedBy: M. Mei; **Event:** eventDate: 2022-04-23; **Record Level:** collectionCode: UR3

#### Andrena distinguenda

Schenck, 1871

173927D5-3F8A-5D5E-A870-33CF7E95201D

##### Materials

- **Type status:** Other material. **Occurrence:** catalogNumber: A0846, A0848; recordedBy: L. Fortini; individualCount: 2; sex: females; lifeStage: adult; occurrenceID: 45D6E0F3-7EF5-5932-A645-BDC945965AD6; **Taxon:** scientificName: Andrena (Distandrena) distinguenda Schenck, 1871; order: Hymenoptera; family: Andrenidae; genus: Andrena; subgenus: Distandrena; specificEpithet: distinguenda; scientificNameAuthorship: Schenck, 1871; **Location:** country: Italy; countryCode: IT; stateProvince: Roma; locality: Riserva Naturale dell'Insugherata 3; decimalLatitude: 41.9644829; decimalLongitude: 12.436101; geodeticDatum: WGS84; coordinatePrecision: 0.0002; **Identification:** identifiedBy: M. Mei; **Event:** eventDate: 2022-04-15; **Record Level:** collectionCode: UR3
- **Type status:** Other material. **Occurrence:** catalogNumber: A0847, A0849; recordedBy: L. Fortini; individualCount: 2; sex: females; lifeStage: adult; occurrenceID: CE2A0545-E586-5E8E-886F-E39A1E4C5402; **Taxon:** scientificName: Andrena (Distandrena) distinguenda Schenck, 1871; order: Hymenoptera; family: Andrenidae; genus: Andrena; subgenus: Distandrena; specificEpithet: distinguenda; scientificNameAuthorship: Schenck, 1871; **Location:** country: Italy; countryCode: IT; stateProvince: Roma; locality: Riserva Naturale Valle dell'Aniene 2; decimalLatitude: 41.928752; decimalLongitude: 12.5562962; geodeticDatum: WGS84; coordinatePrecision: 0.0002; **Identification:** identifiedBy: M. Mei; **Event:** eventDate: 2022-04-28; **Record Level:** collectionCode: UR3
- **Type status:** Other material. **Occurrence:** catalogNumber: A1222, A1225; recordedBy: L. Fortini; individualCount: 2; sex: females; lifeStage: adult; occurrenceID: 38296931-1F26-53BD-A75C-F9FDD07EB628; **Taxon:** scientificName: Andrena (Distandrena) distinguenda Schenck, 1871; order: Hymenoptera; family: Andrenidae; genus: Andrena; subgenus: Distandrena; specificEpithet: distinguenda; scientificNameAuthorship: Schenck, 1871; **Location:** country: Italy; countryCode: IT; stateProvince: Roma; locality: Riserva Naturale Valle dei Casali 1; decimalLatitude: 41.8710627; decimalLongitude: 12.4336809; geodeticDatum: WGS84; coordinatePrecision: 0.0002; **Identification:** identifiedBy: M. Mei; **Event:** eventDate: 2022-04-07; **Record Level:** collectionCode: UR3
- **Type status:** Other material. **Occurrence:** catalogNumber: A1223; recordedBy: L. Fortini; individualCount: 1; sex: females; lifeStage: adult; occurrenceID: ACD6506F-CCF6-56F1-B46B-1910631C81B9; **Taxon:** scientificName: Andrena (Distandrena) distinguenda Schenck, 1871; order: Hymenoptera; family: Andrenidae; genus: Andrena; subgenus: Distandrena; specificEpithet: distinguenda; scientificNameAuthorship: Schenck, 1871; **Location:** country: Italy; countryCode: IT; stateProvince: Roma; locality: Riserva Naturale Valle dei Casali 2; decimalLatitude: 41.8596887; decimalLongitude: 12.4355075; geodeticDatum: WGS84; coordinatePrecision: 0.0002; **Identification:** identifiedBy: M. Mei; **Event:** eventDate: 2022-04-10; **Record Level:** collectionCode: UR3
- **Type status:** Other material. **Occurrence:** catalogNumber: A1226; recordedBy: L. Fortini; individualCount: 1; sex: females; lifeStage: adult; occurrenceID: 99CD02F7-1F1A-5838-88F8-DF1319C5B2CF; **Taxon:** scientificName: Andrena (Distandrena) distinguenda Schenck, 1871; order: Hymenoptera; family: Andrenidae; genus: Andrena; subgenus: Distandrena; specificEpithet: distinguenda; scientificNameAuthorship: Schenck, 1871; **Location:** country: Italy; countryCode: IT; stateProvince: Roma; locality: Riserva Naturale Laurentino-Acqua Acetosa; decimalLatitude: 41.8079275; decimalLongitude: 12.4685548; geodeticDatum: WGS84; coordinatePrecision: 0.0002; **Identification:** identifiedBy: M. Mei; **Event:** eventDate: 2022-04-12; **Record Level:** collectionCode: UR3

#### Andrena flavipes

Panzer, 1799

162ABCC9-D4C3-5AC6-AB39-76B86D9EC6F5

##### Materials

- **Type status:** Other material. **Occurrence:** catalogNumber: A1003; recordedBy: L. Fortini; individualCount: 1; sex: female; lifeStage: adult; occurrenceID: B0535F12-179A-5778-B142-F74A42BF7F8C; **Taxon:** scientificName: Andrena (Zonandrena) flavipes Panzer, 1799; order: Hymenoptera; family: Andrenidae; genus: Andrena; subgenus: Zonandrena; specificEpithet: flavipes; scientificNameAuthorship: Panzer, 1799; **Location:** country: Italy; countryCode: IT; stateProvince: Roma; locality: Riserva Naturale dell'Acquafredda; decimalLatitude: 41.8928408; decimalLongitude: 12.39932; geodeticDatum: WGS84; coordinatePrecision: 0.0002; **Identification:** identifiedBy: M. Mei; **Event:** eventDate: 2022-04-13; **Record Level:** collectionCode: UR3
- **Type status:** Other material. **Occurrence:** catalogNumber: A1031; recordedBy: L. Fortini; individualCount: 1; sex: male; lifeStage: adult; occurrenceID: 6E121FFB-B913-5E1D-BA8F-C4172824424C; **Taxon:** scientificName: Andrena (Zonandrena) flavipes Panzer, 1799; order: Hymenoptera; family: Andrenidae; genus: Andrena; subgenus: Zonandrena; specificEpithet: flavipes; scientificNameAuthorship: Panzer, 1799; **Location:** country: Italy; countryCode: IT; stateProvince: Roma; locality: Riserva Naturale dell'Acquafredda; decimalLatitude: 41.8928408; decimalLongitude: 12.39932; geodeticDatum: WGS84; coordinatePrecision: 0.0002; **Identification:** identifiedBy: M. Mei; **Event:** eventDate: 2022-06-10; **Record Level:** collectionCode: UR3
- **Type status:** Other material. **Occurrence:** catalogNumber: A1035; recordedBy: L. Fortini; individualCount: 1; sex: male; lifeStage: adult; occurrenceID: 38410C7A-A20A-5049-B797-0CD23568E0C6; **Taxon:** scientificName: Andrena (Zonandrena) flavipes Panzer, 1799; order: Hymenoptera; family: Andrenidae; genus: Andrena; subgenus: Zonandrena; specificEpithet: flavipes; scientificNameAuthorship: Panzer, 1799; **Location:** country: Italy; countryCode: IT; stateProvince: Roma; locality: Riserva Regionale dell'Appia Antica 1; decimalLatitude: 41.8623941; decimalLongitude: 12.524863; geodeticDatum: WGS84; coordinatePrecision: 0.0002; **Identification:** identifiedBy: M. Mei; **Event:** eventDate: 2022-06-12; **Record Level:** collectionCode: UR3
- **Type status:** Other material. **Occurrence:** catalogNumber: A1011; recordedBy: L. Fortini; individualCount: 1; sex: female; lifeStage: adult; occurrenceID: 9FBBA7B7-038A-593D-B120-124885EB03E7; **Taxon:** scientificName: Andrena (Zonandrena) flavipes Panzer, 1799; order: Hymenoptera; family: Andrenidae; genus: Andrena; subgenus: Zonandrena; specificEpithet: flavipes; scientificNameAuthorship: Panzer, 1799; **Location:** country: Italy; countryCode: IT; stateProvince: Roma; locality: Riserva Naturale dell'Insugherata 1; decimalLatitude: 41.9555045; decimalLongitude: 12.4292321; geodeticDatum: WGS84; coordinatePrecision: 0.0002; **Identification:** identifiedBy: M. Mei; **Event:** eventDate: 2022-04-15; **Record Level:** collectionCode: UR3
- **Type status:** Other material. **Occurrence:** catalogNumber: A1024, A1025; recordedBy: L. Fortini; individualCount: 2; sex: females; lifeStage: adult; occurrenceID: 78199C39-BD5D-5BE1-B0B8-360CF34F67D7; **Taxon:** scientificName: Andrena (Zonandrena) flavipes Panzer, 1799; order: Hymenoptera; family: Andrenidae; genus: Andrena; subgenus: Zonandrena; specificEpithet: flavipes; scientificNameAuthorship: Panzer, 1799; **Location:** country: Italy; countryCode: IT; stateProvince: Roma; locality: Riserva Naturale dell'Insugherata 1; decimalLatitude: 41.9555045; decimalLongitude: 12.4292321; geodeticDatum: WGS84; coordinatePrecision: 0.0002; **Identification:** identifiedBy: M. Mei; **Event:** eventDate: 2022-05-27; **Record Level:** collectionCode: UR3
- **Type status:** Other material. **Occurrence:** catalogNumber: A1015; recordedBy: L. Fortini; individualCount: 1; sex: female; lifeStage: adult; occurrenceID: 381327C4-531D-5AC6-9BFB-C522A47F61E1; **Taxon:** scientificName: Andrena (Zonandrena) flavipes Panzer, 1799; order: Hymenoptera; family: Andrenidae; genus: Andrena; subgenus: Zonandrena; specificEpithet: flavipes; scientificNameAuthorship: Panzer, 1799; **Location:** country: Italy; countryCode: IT; stateProvince: Roma; locality: Riserva Naturale dell'Insugherata 2; decimalLatitude: 41.9599247; decimalLongitude: 12.433852; geodeticDatum: WGS84; coordinatePrecision: 0.0002; **Identification:** identifiedBy: M. Mei; **Event:** eventDate: 2022-06-24; **Record Level:** collectionCode: UR3
- **Type status:** Other material. **Occurrence:** catalogNumber: A1005, A1006, A1019, A1029; recordedBy: L. Fortini; individualCount: 4; sex: females; lifeStage: adult; occurrenceID: 8393DCD6-9179-5F38-8980-57D6EDCF0A79; **Taxon:** scientificName: Andrena (Zonandrena) flavipes Panzer, 1799; order: Hymenoptera; family: Andrenidae; genus: Andrena; subgenus: Zonandrena; specificEpithet: flavipes; scientificNameAuthorship: Panzer, 1799; **Location:** country: Italy; countryCode: IT; stateProvince: Roma; locality: Riserva Naturale dell'Insugherata 3; decimalLatitude: 41.9644829; decimalLongitude: 12.436101; geodeticDatum: WGS84; coordinatePrecision: 0.0002; **Identification:** identifiedBy: M. Mei; **Event:** eventDate: 2022-06-24; **Record Level:** collectionCode: UR3
- **Type status:** Other material. **Occurrence:** catalogNumber: A1016; recordedBy: L. Fortini; individualCount: 1; sex: female; lifeStage: adult; occurrenceID: E66B8546-5E5F-5BA5-BDC4-623390BE374F; **Taxon:** scientificName: Andrena (Zonandrena) flavipes Panzer, 1799; order: Hymenoptera; family: Andrenidae; genus: Andrena; subgenus: Zonandrena; specificEpithet: flavipes; scientificNameAuthorship: Panzer, 1799; **Location:** country: Italy; countryCode: IT; stateProvince: Roma; locality: Riserva Naturale Laurentino-Acqua Acetosa; decimalLatitude: 41.8079275; decimalLongitude: 12.4685548; geodeticDatum: WGS84; coordinatePrecision: 0.0002; **Identification:** identifiedBy: M. Mei; **Event:** eventDate: 2022-04-12; **Record Level:** collectionCode: UR3
- **Type status:** Other material. **Occurrence:** catalogNumber: A1026; recordedBy: L. Fortini; individualCount: 1; sex: female; lifeStage: adult; occurrenceID: 023CD5FA-C4E5-5E93-A5D9-6F256F3F66A6; **Taxon:** scientificName: Andrena (Zonandrena) flavipes Panzer, 1799; order: Hymenoptera; family: Andrenidae; genus: Andrena; subgenus: Zonandrena; specificEpithet: flavipes; scientificNameAuthorship: Panzer, 1799; **Location:** country: Italy; countryCode: IT; stateProvince: Roma; locality: Riserva Naturale Laurentino-Acqua Acetosa; decimalLatitude: 41.8079275; decimalLongitude: 12.4685548; geodeticDatum: WGS84; coordinatePrecision: 0.0002; **Identification:** identifiedBy: M. Mei; **Event:** eventDate: 2022-06-16; **Record Level:** collectionCode: UR3
- **Type status:** Other material. **Occurrence:** catalogNumber: A1002, A1004, A1007, A1013, A1017, A1018, A1021, A1027, A1028; recordedBy: L. Fortini; individualCount: 9; sex: females; lifeStage: adult; occurrenceID: 289470B5-DB50-559C-ACCD-D64442E6FC63; **Taxon:** scientificName: Andrena (Zonandrena) flavipes Panzer, 1799; order: Hymenoptera; family: Andrenidae; genus: Andrena; subgenus: Zonandrena; specificEpithet: flavipes; scientificNameAuthorship: Panzer, 1799; **Location:** country: Italy; countryCode: IT; stateProvince: Roma; locality: Riserva Naturale Tenuta dei Massimi 1; decimalLatitude: 41.8532859; decimalLongitude: 12.3842322; geodeticDatum: WGS84; coordinatePrecision: 0.0002; **Identification:** identifiedBy: M. Mei; **Event:** eventDate: 2022-04-23; **Record Level:** collectionCode: UR3
- **Type status:** Other material. **Occurrence:** catalogNumber: A1032; recordedBy: L. Fortini; individualCount: 1; sex: male; lifeStage: adult; occurrenceID: 14D620B1-7650-5E4F-9D32-A55D06B02EA9; **Taxon:** scientificName: Andrena (Zonandrena) flavipes Panzer, 1799; order: Hymenoptera; family: Andrenidae; genus: Andrena; subgenus: Zonandrena; specificEpithet: flavipes; scientificNameAuthorship: Panzer, 1799; **Location:** country: Italy; countryCode: IT; stateProvince: Roma; locality: Riserva Naturale Tenuta dei Massimi 1; decimalLatitude: 41.8532859; decimalLongitude: 12.3842322; geodeticDatum: WGS84; coordinatePrecision: 0.0002; **Identification:** identifiedBy: M. Mei; **Event:** eventDate: 2022-06-01; **Record Level:** collectionCode: UR3
- **Type status:** Other material. **Occurrence:** catalogNumber: A1037; recordedBy: L. Fortini; individualCount: 1; sex: male; lifeStage: adult; occurrenceID: D4C7A265-FBE7-5C73-8009-E06712E77A02; **Taxon:** scientificName: Andrena (Zonandrena) flavipes Panzer, 1799; order: Hymenoptera; family: Andrenidae; genus: Andrena; subgenus: Zonandrena; specificEpithet: flavipes; scientificNameAuthorship: Panzer, 1799; **Location:** country: Italy; countryCode: IT; stateProvince: Roma; locality: Riserva Naturale Tenuta dei Massimi 1; decimalLatitude: 41.8532859; decimalLongitude: 12.3842322; geodeticDatum: WGS84; coordinatePrecision: 0.0002; **Identification:** identifiedBy: M. Mei; **Event:** eventDate: 2022-04-27; **Record Level:** collectionCode: UR3
- **Type status:** Other material. **Occurrence:** catalogNumber: A1023; recordedBy: L. Fortini; individualCount: 1; sex: female; lifeStage: adult; occurrenceID: EB18E56F-F749-50A3-93B3-47DD6ECE8E8A; **Taxon:** scientificName: Andrena (Zonandrena) flavipes Panzer, 1799; order: Hymenoptera; family: Andrenidae; genus: Andrena; subgenus: Zonandrena; specificEpithet: flavipes; scientificNameAuthorship: Panzer, 1799; **Location:** country: Italy; countryCode: IT; stateProvince: Roma; locality: Riserva Naturale Tenuta dei Massimi 2; decimalLatitude: 41.8316516; decimalLongitude: 12.3999927; geodeticDatum: WGS84; coordinatePrecision: 0.0002; **Identification:** identifiedBy: M. Mei; **Event:** eventDate: 2022-06-01; **Record Level:** collectionCode: UR3
- **Type status:** Other material. **Occurrence:** catalogNumber: A1036; recordedBy: L. Fortini; individualCount: 1; sex: male; lifeStage: adult; occurrenceID: 7D9DE7AF-1641-5089-9232-47554FB276A2; **Taxon:** scientificName: Andrena (Zonandrena) flavipes Panzer, 1799; order: Hymenoptera; family: Andrenidae; genus: Andrena; subgenus: Zonandrena; specificEpithet: flavipes; scientificNameAuthorship: Panzer, 1799; **Location:** country: Italy; countryCode: IT; stateProvince: Roma; locality: Riserva Naturale Tenuta dei Massimi 2; decimalLatitude: 41.8316516; decimalLongitude: 12.3999927; geodeticDatum: WGS84; coordinatePrecision: 0.0002; **Identification:** identifiedBy: M. Mei; **Event:** eventDate: 2022-06-27; **Record Level:** collectionCode: UR3
- **Type status:** Other material. **Occurrence:** catalogNumber: A1009; recordedBy: L. Fortini; individualCount: 1; sex: female; lifeStage: adult; occurrenceID: 970C4BAB-90A8-57C8-92D3-9ACBCF9FDF07; **Taxon:** scientificName: Andrena (Zonandrena) flavipes Panzer, 1799; order: Hymenoptera; family: Andrenidae; genus: Andrena; subgenus: Zonandrena; specificEpithet: flavipes; scientificNameAuthorship: Panzer, 1799; **Location:** country: Italy; countryCode: IT; stateProvince: Roma; locality: Riserva Naturale Valle dell'Aniene 1; decimalLatitude: 41.9345179; decimalLongitude: 12.5453096; geodeticDatum: WGS84; coordinatePrecision: 0.0002; **Identification:** identifiedBy: M. Mei; **Event:** eventDate: 2022-06-05; **Record Level:** collectionCode: UR3
- **Type status:** Other material. **Occurrence:** catalogNumber: A1022, A1038; recordedBy: L. Fortini; individualCount: 2; sex: 1 male, 1 female; lifeStage: adult; occurrenceID: 4F475137-F2C2-5720-964D-A99879DD9CDB; **Taxon:** scientificName: Andrena (Zonandrena) flavipes Panzer, 1799; order: Hymenoptera; family: Andrenidae; genus: Andrena; subgenus: Zonandrena; specificEpithet: flavipes; scientificNameAuthorship: Panzer, 1799; **Location:** country: Italy; countryCode: IT; stateProvince: Roma; locality: Riserva Naturale Valle dell'Aniene 1; decimalLatitude: 41.9345179; decimalLongitude: 12.5453096; geodeticDatum: WGS84; coordinatePrecision: 0.0002; **Identification:** identifiedBy: M. Mei; **Event:** eventDate: 2022-04-28; **Record Level:** collectionCode: UR3
- **Type status:** Other material. **Occurrence:** catalogNumber: A1001, A1020, A1039; recordedBy: L. Fortini; individualCount: 3; sex: females; lifeStage: adult; occurrenceID: 88E991B4-EF0F-5BB7-A8DF-40F07DD29243; **Taxon:** scientificName: Andrena (Zonandrena) flavipes Panzer, 1799; order: Hymenoptera; family: Andrenidae; genus: Andrena; subgenus: Zonandrena; specificEpithet: flavipes; scientificNameAuthorship: Panzer, 1799; **Location:** country: Italy; countryCode: IT; stateProvince: Roma; locality: Riserva Naturale Valle dell'Aniene 2; decimalLatitude: 41.928752; decimalLongitude: 12.5562962; geodeticDatum: WGS84; coordinatePrecision: 0.0002; **Identification:** identifiedBy: M. Mei; **Event:** eventDate: 2022-04-28; **Record Level:** collectionCode: UR3
- **Type status:** Other material. **Occurrence:** catalogNumber: A1030; recordedBy: L. Fortini; individualCount: 1; sex: female; lifeStage: adult; occurrenceID: B494CFDA-59FD-574A-8E0B-76B654878FAB; **Taxon:** scientificName: Andrena (Zonandrena) flavipes Panzer, 1799; order: Hymenoptera; family: Andrenidae; genus: Andrena; subgenus: Zonandrena; specificEpithet: flavipes; scientificNameAuthorship: Panzer, 1799; **Location:** country: Italy; countryCode: IT; stateProvince: Roma; locality: Riserva Naturale Valle dell'Aniene 2; decimalLatitude: 41.928752; decimalLongitude: 12.5562962; geodeticDatum: WGS84; coordinatePrecision: 0.0002; **Identification:** identifiedBy: M. Mei; **Event:** eventDate: 2022-06-05; **Record Level:** collectionCode: UR3
- **Type status:** Other material. **Occurrence:** catalogNumber: A1008, A1033, A1034; recordedBy: L. Fortini; individualCount: 3; sex: 2 males, 1 female; lifeStage: adult; occurrenceID: B8C9237A-42E2-5958-BB0B-6040B435343A; **Taxon:** scientificName: Andrena (Zonandrena) flavipes Panzer, 1799; order: Hymenoptera; family: Andrenidae; genus: Andrena; subgenus: Zonandrena; specificEpithet: flavipes; scientificNameAuthorship: Panzer, 1799; **Location:** country: Italy; countryCode: IT; stateProvince: Roma; locality: Riserva Naturale Valle dei Casali 1; decimalLatitude: 41.8710627; decimalLongitude: 12.4336809; geodeticDatum: WGS84; coordinatePrecision: 0.0002; **Identification:** identifiedBy: M. Mei; **Event:** eventDate: 2022-04-07; **Record Level:** collectionCode: UR3
- **Type status:** Other material. **Occurrence:** catalogNumber: A1010; recordedBy: L. Fortini; individualCount: 1; sex: female; lifeStage: adult; occurrenceID: 1FCD45DE-CAC5-52E8-BC3D-AF9910611C0F; **Taxon:** scientificName: Andrena (Zonandrena) flavipes Panzer, 1799; order: Hymenoptera; family: Andrenidae; genus: Andrena; subgenus: Zonandrena; specificEpithet: flavipes; scientificNameAuthorship: Panzer, 1799; **Location:** country: Italy; countryCode: IT; stateProvince: Roma; locality: Riserva Naturale Valle dei Casali 1; decimalLatitude: 41.8710627; decimalLongitude: 12.4336809; geodeticDatum: WGS84; coordinatePrecision: 0.0002; **Identification:** identifiedBy: M. Mei; **Event:** eventDate: 2022-06-18; **Record Level:** collectionCode: UR3
- **Type status:** Other material. **Occurrence:** catalogNumber: A1012, A1014; recordedBy: L. Fortini; individualCount: 2; sex: females; lifeStage: adult; occurrenceID: B1E6130A-60F4-536B-A0AD-9EF45A19D39D; **Taxon:** scientificName: Andrena (Zonandrena) flavipes Panzer, 1799; order: Hymenoptera; family: Andrenidae; genus: Andrena; subgenus: Zonandrena; specificEpithet: flavipes; scientificNameAuthorship: Panzer, 1799; **Location:** country: Italy; countryCode: IT; stateProvince: Roma; locality: Riserva Naturale Valle dei Casali 2; decimalLatitude: 41.8596887; decimalLongitude: 12.4355075; geodeticDatum: WGS84; coordinatePrecision: 0.0002; **Identification:** identifiedBy: M. Mei; **Event:** eventDate: 2022-04-10; **Record Level:** collectionCode: UR3

#### Andrena foeniculae

Wood, 2020

6D7494E7-C20D-5687-8D18-0D89A4C2F289

##### Materials

- **Type status:** Other material. **Occurrence:** catalogNumber: A0836; recordedBy: L. Fortini; individualCount: 1; sex: female; lifeStage: adult; occurrenceID: C58D0DC7-908D-5C72-9F0D-1E9572B42278; **Taxon:** scientificName: Andrena (Notandrena) foeniculae Wood, 2020; order: Hymenoptera; family: Andrenidae; genus: Andrena; subgenus: Notandrena; specificEpithet: foeniculae; scientificNameAuthorship: Wood, 2020; **Location:** country: Italy; countryCode: IT; stateProvince: Roma; locality: Riserva Naturale Tenuta dei Massimi 2; decimalLatitude: 41.8316516; decimalLongitude: 12.3999927; geodeticDatum: WGS84; coordinatePrecision: 0.0002; **Identification:** identifiedBy: M. Mei; **Event:** eventDate: 2022-09-28; **Record Level:** collectionCode: UR3

#### Andrena fulvago

Christ, 1791

4964CB8E-A2D5-57C8-B534-1288A5CE6DAE

##### Materials

- **Type status:** Other material. **Occurrence:** catalogNumber: A0865; recordedBy: L. Fortini; individualCount: 1; sex: female; lifeStage: adult; occurrenceID: 029A84B9-6B36-5F03-B4D1-EF6F970722D2; **Taxon:** scientificName: Andrena (Chrysandrena) fulvago Christ, 1791; order: Hymenoptera; family: Andrenidae; genus: Andrena; subgenus: Chrysandrena; specificEpithet: fulvago; scientificNameAuthorship: Christ, 1791; **Location:** country: Italy; countryCode: IT; stateProvince: Roma; locality: Riserva Naturale Laurentino-Acqua Acetosa; decimalLatitude: 41.8079275; decimalLongitude: 12.4685548; geodeticDatum: WGS84; coordinatePrecision: 0.0002; **Identification:** identifiedBy: M. Mei; **Event:** eventDate: 2022-05-12; **Record Level:** collectionCode: UR3
- **Type status:** Other material. **Occurrence:** catalogNumber: A0867, A0868, A0974, A0975, A0976, A0977; recordedBy: L. Fortini; individualCount: 6; sex: 4 males, 2 females; lifeStage: adult; occurrenceID: F20AEDBB-7FC8-5520-B22E-BAC9CB747788; **Taxon:** scientificName: Andrena (Chrysandrena) fulvago Christ, 1791; order: Hymenoptera; family: Andrenidae; genus: Andrena; subgenus: Chrysandrena; specificEpithet: fulvago; scientificNameAuthorship: Christ, 1791; **Location:** country: Italy; countryCode: IT; stateProvince: Roma; locality: Riserva Naturale Tenuta dei Massimi 1; decimalLatitude: 41.8532859; decimalLongitude: 12.3842322; geodeticDatum: WGS84; coordinatePrecision: 0.0002; **Identification:** identifiedBy: M. Mei; **Event:** eventDate: 2022-06-01; **Record Level:** collectionCode: UR3
- **Type status:** Other material. **Occurrence:** catalogNumber: A0869; recordedBy: L. Fortini; individualCount: 1; sex: female; lifeStage: adult; occurrenceID: DA225A19-8EDF-5FFB-9444-E9F1AE62D70A; **Taxon:** scientificName: Andrena (Chrysandrena) fulvago Christ, 1791; order: Hymenoptera; family: Andrenidae; genus: Andrena; subgenus: Chrysandrena; specificEpithet: fulvago; scientificNameAuthorship: Christ, 1791; **Location:** country: Italy; countryCode: IT; stateProvince: Roma; locality: Riserva Regionale dell'Appia Antica 1; decimalLatitude: 41.8623941; decimalLongitude: 12.524863; geodeticDatum: WGS84; coordinatePrecision: 0.0002; **Identification:** identifiedBy: M. Mei; **Event:** eventDate: 2022-06-12; **Record Level:** collectionCode: UR3
- **Type status:** Other material. **Occurrence:** catalogNumber: A0872, A0973; recordedBy: L. Fortini; individualCount: 2; sex: 1 male, 1 female; lifeStage: adult; occurrenceID: D94B2F41-BF71-57C7-8700-81FA6C4621EF; **Taxon:** scientificName: Andrena (Chrysandrena) fulvago Christ, 1791; order: Hymenoptera; family: Andrenidae; genus: Andrena; subgenus: Chrysandrena; specificEpithet: fulvago; scientificNameAuthorship: Christ, 1791; **Location:** country: Italy; countryCode: IT; stateProvince: Roma; locality: Riserva Naturale di Monte Mario; decimalLatitude: 41.9386215; decimalLongitude: 12.4546223; geodeticDatum: WGS84; coordinatePrecision: 0.0002; **Identification:** identifiedBy: M. Mei; **Event:** eventDate: 2022-06-19; **Record Level:** collectionCode: UR3
- **Type status:** Other material. **Occurrence:** catalogNumber: A0866, A0873, A0874, A0876; recordedBy: L. Fortini; individualCount: 4; sex: females; lifeStage: adult; occurrenceID: 4C8D95A6-F089-50CB-A7A9-89E73D726685; **Taxon:** scientificName: Andrena (Chrysandrena) fulvago Christ, 1791; order: Hymenoptera; family: Andrenidae; genus: Andrena; subgenus: Chrysandrena; specificEpithet: fulvago; scientificNameAuthorship: Christ, 1791; **Location:** country: Italy; countryCode: IT; stateProvince: Roma; locality: Riserva Naturale Tenuta dei Massimi 1; decimalLatitude: 41.8532859; decimalLongitude: 12.3842322; geodeticDatum: WGS84; coordinatePrecision: 0.0002; **Identification:** identifiedBy: M. Mei; **Event:** eventDate: 2022-06-27; **Record Level:** collectionCode: UR3

#### Andrena fulvitarsis

Brullé, 1832

37D2B920-8D76-5490-80BB-A776E6BE1289

##### Materials

- **Type status:** Other material. **Occurrence:** catalogNumber: A1072, A1073; recordedBy: L. Fortini; individualCount: 2; sex: females; lifeStage: adult; occurrenceID: EA8F5D0C-11E9-5C56-99AD-792004CB2F6D; **Taxon:** scientificName: Andrena (Ulandrena) fulvitarsis Brullé, 1832; order: Hymenoptera; family: Andrenidae; genus: Andrena; subgenus: Ulandrena; specificEpithet: fulvitarsis; scientificNameAuthorship: Brullé, 1832; **Location:** country: Italy; countryCode: IT; stateProvince: Roma; locality: Riserva Naturale dell'Insugherata 1; decimalLatitude: 41.9555045; decimalLongitude: 12.4292321; geodeticDatum: WGS84; coordinatePrecision: 0.0002; **Identification:** identifiedBy: M. Mei; **Event:** eventDate: 2022-05-27; **Record Level:** collectionCode: UR3

#### Andrena fuscosa

Erichson, 1835

39E70B2B-FE11-54DC-8B22-7FF3649D7D23

##### Materials

- **Type status:** Other material. **Occurrence:** catalogNumber: A1063; recordedBy: L. Fortini; individualCount: 1; sex: female; lifeStage: adult; occurrenceID: B8A6C819-4F62-5402-A95A-C3B01224B75A; **Taxon:** scientificName: Andrena (Melanapis) fuscosa Erichson, 1835; order: Hymenoptera; family: Andrenidae; genus: Andrena; subgenus: Melanapis; specificEpithet: fuscosa; scientificNameAuthorship: Erichson, 1835; **Location:** country: Italy; countryCode: IT; stateProvince: Roma; locality: Riserva Naturale dell'Insugherata 2; decimalLatitude: 41.9599247; decimalLongitude: 12.433852; geodeticDatum: WGS84; coordinatePrecision: 0.0002; **Identification:** identifiedBy: M. Mei; **Event:** eventDate: 2022-04-15; **Record Level:** collectionCode: UR3
- **Type status:** Other material. **Occurrence:** catalogNumber: A1058, A1060, A1061, A1062; recordedBy: L. Fortini; individualCount: 4; sex: males; lifeStage: adult; occurrenceID: 0B56A617-BB76-5382-BF6F-C2B776EEECF4; **Taxon:** scientificName: Andrena (Melanapis) fuscosa Erichson, 1835; order: Hymenoptera; family: Andrenidae; genus: Andrena; subgenus: Melanapis; specificEpithet: fuscosa; scientificNameAuthorship: Erichson, 1835; **Location:** country: Italy; countryCode: IT; stateProvince: Roma; locality: Riserva Naturale di Monte Mario; decimalLatitude: 41.9386215; decimalLongitude: 12.4546223; geodeticDatum: WGS84; coordinatePrecision: 0.0002; **Identification:** identifiedBy: M. Mei; **Event:** eventDate: 2022-06-01; **Record Level:** collectionCode: UR3
- **Type status:** Other material. **Occurrence:** catalogNumber: A1059; recordedBy: L. Fortini; individualCount: 1; sex: male; lifeStage: adult; occurrenceID: 08A1C025-1BF6-5A2C-BD6F-007D50525F97; **Taxon:** scientificName: Andrena (Melanapis) fuscosa Erichson, 1835; order: Hymenoptera; family: Andrenidae; genus: Andrena; subgenus: Melanapis; specificEpithet: fuscosa; scientificNameAuthorship: Erichson, 1835; **Location:** country: Italy; countryCode: IT; stateProvince: Roma; locality: Riserva Naturale Tenuta dei Massimi 2; decimalLatitude: 41.8316516; decimalLongitude: 12.3999927; geodeticDatum: WGS84; coordinatePrecision: 0.0002; **Identification:** identifiedBy: M. Mei; **Event:** eventDate: 2022-06-20; **Record Level:** collectionCode: UR3

#### Andrena hattorfiana

Fabricius, 1775

CD091253-7DA3-53F2-8C5E-58F8BD489922

##### Materials

- **Type status:** Other material. **Occurrence:** catalogNumber: A1085; recordedBy: L. Fortini; individualCount: 1; sex: female; lifeStage: adult; occurrenceID: A9FD59D2-EA99-50FE-A83B-7258EA612425; **Taxon:** scientificName: Andrena (Charitandrena) hattorfiana (Fabricius, 1775); order: Hymenoptera; family: Andrenidae; genus: Andrena; subgenus: Charitandrena; specificEpithet: hattorfiana; scientificNameAuthorship: (Fabricius, 1775); **Location:** country: Italy; countryCode: IT; stateProvince: Roma; locality: Riserva Naturale dell'Insugherata 2; decimalLatitude: 41.9599247; decimalLongitude: 12.433852; geodeticDatum: WGS84; coordinatePrecision: 0.0002; **Identification:** identifiedBy: M. Mei; **Event:** eventDate: 2022-05-27; **Record Level:** collectionCode: UR3

#### Andrena hesperia

Smith, 1853

84C33E8F-1F96-582E-B41F-AEBB32EA2699

##### Materials

- **Type status:** Other material. **Occurrence:** catalogNumber: A0864; recordedBy: L. Fortini; individualCount: 1; sex: female; lifeStage: adult; occurrenceID: A5542DC7-C6D0-5A7E-A149-EFA2141383C4; **Taxon:** scientificName: Andrena (Chrysandrena) esperia Smith, 1853; order: Hymenoptera; family: Andrenidae; genus: Andrena; subgenus: Chrysandrena; specificEpithet: hesperia; scientificNameAuthorship: Smith, 1853; **Location:** country: Italy; countryCode: IT; stateProvince: Roma; locality: Riserva Naturale di Monte Mario; decimalLatitude: 41.9386215; decimalLongitude: 12.4546223; geodeticDatum: WGS84; coordinatePrecision: 0.0002; **Identification:** identifiedBy: M. Mei; **Event:** eventDate: 2022-04-20; **Record Level:** collectionCode: UR3
- **Type status:** Other material. **Occurrence:** catalogNumber: A0875; recordedBy: L. Fortini; individualCount: 1; sex: female; lifeStage: adult; occurrenceID: BE3AFED1-D5FA-589C-ABA4-10F5FF04EF1D; **Taxon:** scientificName: Andrena (Chrysandrena) esperia Smith, 1853; order: Hymenoptera; family: Andrenidae; genus: Andrena; subgenus: Chrysandrena; specificEpithet: hesperia; scientificNameAuthorship: Smith, 1853; **Location:** country: Italy; countryCode: IT; stateProvince: Roma; locality: Riserva Naturale Tenuta dei Massimi 1; decimalLatitude: 41.8532859; decimalLongitude: 12.3842322; geodeticDatum: WGS84; coordinatePrecision: 0.0002; **Identification:** identifiedBy: M. Mei; **Event:** eventDate: 2022-04-23; **Record Level:** collectionCode: UR3
- **Type status:** Other material. **Occurrence:** catalogNumber: A2013, A2022; recordedBy: L. Fortini; individualCount: 2; sex: females; lifeStage: adult; occurrenceID: FAC5BA9C-2E0E-5E38-B92D-937E906C150B; **Taxon:** scientificName: Andrena (Chrysandrena) esperia Smith, 1853; order: Hymenoptera; family: Andrenidae; genus: Andrena; subgenus: Chrysandrena; specificEpithet: hesperia; scientificNameAuthorship: Smith, 1853; **Location:** country: Italy; countryCode: IT; stateProvince: Roma; locality: Riserva Regionale dell'Appia Antica 2; decimalLatitude: 41.8402564; decimalLongitude: 12.532773; geodeticDatum: WGS84; coordinatePrecision: 0.0002; **Identification:** identifiedBy: M. Mei; **Event:** eventDate: 2022-04-25; **Record Level:** collectionCode: UR3

#### Andrena humilis

Imhoff, 1832

F1B83EE0-9851-5EFA-9D03-DCECC68A3540

##### Materials

- **Type status:** Other material. **Occurrence:** catalogNumber: A0984, A0985, A0986, A0987, A0988, A0990, A0991, A0993; recordedBy: L. Fortini; individualCount: 8; sex: males; lifeStage: adult; occurrenceID: 9510CFEC-728E-5DB1-9CBE-1E831A907A44; **Taxon:** scientificName: Andrena (Chlorandrena) humilis Imhoff, 1832; order: Hymenoptera; family: Andrenidae; genus: Andrena; subgenus: Chlorandrena; specificEpithet: humilis; scientificNameAuthorship: Imhoff, 1832; **Location:** country: Italy; countryCode: IT; stateProvince: Roma; locality: Riserva Regionale dell'Appia Antica 1; decimalLatitude: 41.8623941; decimalLongitude: 12.524863; geodeticDatum: WGS84; coordinatePrecision: 0.0002; **Identification:** identifiedBy: M. Mei; **Event:** eventDate: 2022-05-10; **Record Level:** collectionCode: UR3
- **Type status:** Other material. **Occurrence:** catalogNumber: A0989; recordedBy: L. Fortini; individualCount: 1; sex: male; lifeStage: adult; occurrenceID: 9A483303-357F-5555-B07C-919441DE870D; **Taxon:** scientificName: Andrena (Chlorandrena) humilis Imhoff, 1832; order: Hymenoptera; family: Andrenidae; genus: Andrena; subgenus: Chlorandrena; specificEpithet: humilis; scientificNameAuthorship: Imhoff, 1832; **Location:** country: Italy; countryCode: IT; stateProvince: Roma; locality: Riserva Naturale Valle dell'Aniene 2; decimalLatitude: 41.928752; decimalLongitude: 12.5562962; geodeticDatum: WGS84; coordinatePrecision: 0.0002; **Identification:** identifiedBy: M. Mei; **Event:** eventDate: 2022-06-05; **Record Level:** collectionCode: UR3
- **Type status:** Other material. **Occurrence:** catalogNumber: A0992; recordedBy: L. Fortini; individualCount: 1; sex: male; lifeStage: adult; occurrenceID: 7D071A4D-166D-5716-8BAF-E5C1173B81B3; **Taxon:** scientificName: Andrena (Chlorandrena) humilis Imhoff, 1832; order: Hymenoptera; family: Andrenidae; genus: Andrena; subgenus: Chlorandrena; specificEpithet: humilis; scientificNameAuthorship: Imhoff, 1832; **Location:** country: Italy; countryCode: IT; stateProvince: Roma; locality: Riserva Naturale Tenuta dei Massimi 1; decimalLatitude: 41.8532859; decimalLongitude: 12.3842322; geodeticDatum: WGS84; coordinatePrecision: 0.0002; **Identification:** identifiedBy: M. Mei; **Event:** eventDate: 2022-06-23; **Record Level:** collectionCode: UR3

#### Andrena impunctata

Pérez, 1895

01AA32C3-E3FA-58D6-8D57-FB170C9ACC1F

##### Materials

- **Type status:** Other material. **Occurrence:** catalogNumber: A1205; recordedBy: L. Fortini; individualCount: 1; sex: male; lifeStage: adult; occurrenceID: C402C1CA-C333-5CB7-8413-9DDF27A6EC69; **Taxon:** scientificName: Andrena (Graecandrena) impunctata Pérez, 1895; order: Hymenoptera; family: Andrenidae; genus: Andrena; subgenus: Graecandrena; specificEpithet: impunctata; scientificNameAuthorship: Pérez, 1895; **Location:** country: Italy; countryCode: IT; stateProvince: Roma; locality: Riserva Naturale Valle dei Casali 2; decimalLatitude: 41.8596887; decimalLongitude: 12.4355075; geodeticDatum: WGS84; coordinatePrecision: 0.0002; **Identification:** identifiedBy: M. Mei; **Event:** eventDate: 2022-04-10; **Record Level:** collectionCode: UR3
- **Type status:** Other material. **Occurrence:** catalogNumber: A1240; recordedBy: L. Fortini; individualCount: 1; sex: male; lifeStage: adult; occurrenceID: 928827E9-DDC3-591D-90F9-39D85D8A4AEC; **Taxon:** scientificName: Andrena (Graecandrena) impunctata Pérez, 1895; order: Hymenoptera; family: Andrenidae; genus: Andrena; subgenus: Graecandrena; specificEpithet: impunctata; scientificNameAuthorship: Pérez, 1895; **Location:** country: Italy; countryCode: IT; stateProvince: Roma; locality: Riserva Naturale Valle dei Casali 2; decimalLatitude: 41.8596887; decimalLongitude: 12.4355075; geodeticDatum: WGS84; coordinatePrecision: 0.0002; **Identification:** identifiedBy: M. Mei; **Event:** eventDate: 2022-05-14; **Record Level:** collectionCode: UR3

#### Andrena labialis

(Kirby, 1802)

72602B1D-39CC-51FF-8EFF-EAE86BAA97DC

##### Materials

- **Type status:** Other material. **Occurrence:** catalogNumber: A1102, A1105; recordedBy: L. Fortini; individualCount: 2; sex: 1 male 1 female; lifeStage: adult; occurrenceID: B62C7B0B-CF2D-5C4F-B8AB-1BD7BC054DF9; **Taxon:** scientificName: Andrena (Holandrena) labialis (Kirby, 1802); order: Hymenoptera; family: Andrenidae; genus: Andrena; subgenus: Holandrena; specificEpithet: labialis; scientificNameAuthorship: (Kirby, 1802); **Location:** country: Italy; countryCode: IT; stateProvince: Roma; locality: Riserva Naturale Valle dell'Aniene 1; decimalLatitude: 41.9345179; decimalLongitude: 12.5453096; geodeticDatum: WGS84; coordinatePrecision: 0.0002; **Identification:** identifiedBy: M. Mei; **Event:** eventDate: 2022-06-05; **Record Level:** collectionCode: UR3
- **Type status:** Other material. **Occurrence:** catalogNumber: A1103; recordedBy: L. Fortini; individualCount: 1; sex: female; lifeStage: adult; occurrenceID: BCC92F3B-2B94-5B3A-A461-5524A1E65421; **Taxon:** scientificName: Andrena (Holandrena) labialis (Kirby, 1802); order: Hymenoptera; family: Andrenidae; genus: Andrena; subgenus: Holandrena; specificEpithet: labialis; scientificNameAuthorship: (Kirby, 1802); **Location:** country: Italy; countryCode: IT; stateProvince: Roma; locality: Riserva Naturale Tenuta dei Massimi 2; decimalLatitude: 41.8316516; decimalLongitude: 12.3999927; geodeticDatum: WGS84; coordinatePrecision: 0.0002; **Identification:** identifiedBy: M. Mei; **Event:** eventDate: 2022-06-01; **Record Level:** collectionCode: UR3
- **Type status:** Other material. **Occurrence:** catalogNumber: A1104; recordedBy: L. Fortini; individualCount: 1; sex: male; lifeStage: adult; occurrenceID: 2E1D06B4-43AD-5A78-8BAB-C8F4132E7C33; **Taxon:** scientificName: Andrena (Holandrena) labialis (Kirby, 1802); order: Hymenoptera; family: Andrenidae; genus: Andrena; subgenus: Holandrena; specificEpithet: labialis; scientificNameAuthorship: (Kirby, 1802); **Location:** country: Italy; countryCode: IT; stateProvince: Roma; locality: Riserva Naturale dell'Insugherata 2; decimalLatitude: 41.9599247; decimalLongitude: 12.433852; geodeticDatum: WGS84; coordinatePrecision: 0.0002; **Identification:** identifiedBy: M. Mei; **Event:** eventDate: 2022-05-27; **Record Level:** collectionCode: UR3

#### Andrena labiata

Fabricius, 1781

A0C1CF0A-7A58-5815-8029-C1C4CA5C925F

##### Materials

- **Type status:** Other material. **Occurrence:** catalogNumber: A1084; recordedBy: L. Fortini; individualCount: 1; sex: male; lifeStage: adult; occurrenceID: 40D713B9-E3A1-5F65-B918-5E912A6C376B; **Taxon:** scientificName: Andrena (Poecilandrena) labiata Fabricius, 1781; order: Hymenoptera; family: Andrenidae; genus: Andrena; subgenus: Poecilandrena; specificEpithet: labiata; scientificNameAuthorship: Fabricius, 1781; **Location:** country: Italy; countryCode: IT; stateProvince: Roma; locality: Riserva Naturale dell'Insugherata 3; decimalLatitude: 41.9644829; decimalLongitude: 12.436101; geodeticDatum: WGS84; coordinatePrecision: 0.0002; **Identification:** identifiedBy: M. Mei; **Event:** eventDate: 2022-04-15; **Record Level:** collectionCode: UR3

#### Andrena lagopus

Latreille, 1809

B6B22079-A01D-572F-BAE0-064839642ECB

##### Materials

- **Type status:** Other material. **Occurrence:** catalogNumber: A1075, A1080, A1095, A1097; recordedBy: L. Fortini; individualCount: 2; sex: 2 males, 2 females; lifeStage: adult; occurrenceID: 7DF0CE11-BE37-5874-A18D-7E275D45B3F7; **Taxon:** scientificName: Andrena (Biareolina) lagopus Latreille, 1809; order: Hymenoptera; family: Andrenidae; genus: Andrena; subgenus: Biareolina; specificEpithet: lagopus; scientificNameAuthorship: Latreille, 1809; **Location:** country: Italy; countryCode: IT; stateProvince: Roma; locality: Riserva Naturale dell'Acquafredda; decimalLatitude: 41.8928408; decimalLongitude: 12.39932; geodeticDatum: WGS84; coordinatePrecision: 0.0002; **Identification:** identifiedBy: M. Mei; **Event:** eventDate: 2022-04-13; **Record Level:** collectionCode: UR3
- **Type status:** Other material. **Occurrence:** catalogNumber: A1078; recordedBy: L. Fortini; individualCount: 1; sex: female; lifeStage: adult; occurrenceID: F660D9A6-3A60-5445-B605-7361B9FD3C8C; **Taxon:** scientificName: Andrena (Biareolina) lagopus Latreille, 1809; order: Hymenoptera; family: Andrenidae; genus: Andrena; subgenus: Biareolina; specificEpithet: lagopus; scientificNameAuthorship: Latreille, 1809; **Location:** country: Italy; countryCode: IT; stateProvince: Roma; locality: Riserva Naturale dell'Insugherata 2; decimalLatitude: 41.9599247; decimalLongitude: 12.433852; geodeticDatum: WGS84; coordinatePrecision: 0.0002; **Identification:** identifiedBy: M. Mei; **Event:** eventDate: 2022-04-15; **Record Level:** collectionCode: UR3
- **Type status:** Other material. **Occurrence:** catalogNumber: A1079, A1094, A1096, A1099, A1100, A1101; recordedBy: L. Fortini; individualCount: 6; sex: 5 males, 1 female; lifeStage: adult; occurrenceID: 787E2002-27E3-517E-B4F7-E43C08C4F51D; **Taxon:** scientificName: Andrena (Biareolina) lagopus Latreille, 1809; order: Hymenoptera; family: Andrenidae; genus: Andrena; subgenus: Biareolina; specificEpithet: lagopus; scientificNameAuthorship: Latreille, 1809; **Location:** country: Italy; countryCode: IT; stateProvince: Roma; locality: Riserva Naturale dell'Insugherata 3; decimalLatitude: 41.9644829; decimalLongitude: 12.436101; geodeticDatum: WGS84; coordinatePrecision: 0.0002; **Identification:** identifiedBy: M. Mei; **Event:** eventDate: 2022-04-15; **Record Level:** collectionCode: UR3
- **Type status:** Other material. **Occurrence:** catalogNumber: A1082; recordedBy: L. Fortini; individualCount: 1; sex: female; lifeStage: adult; occurrenceID: C0EF084A-5C12-5A10-B0C7-B88EC763B1E9; **Taxon:** scientificName: Andrena (Biareolina) lagopus Latreille, 1809; order: Hymenoptera; family: Andrenidae; genus: Andrena; subgenus: Biareolina; specificEpithet: lagopus; scientificNameAuthorship: Latreille, 1809; **Location:** country: Italy; countryCode: IT; stateProvince: Roma; locality: Riserva Naturale di Monte Mario; decimalLatitude: 41.9386215; decimalLongitude: 12.4546223; geodeticDatum: WGS84; coordinatePrecision: 0.0002; **Identification:** identifiedBy: M. Mei; **Event:** eventDate: 2022-04-20; **Record Level:** collectionCode: UR3
- **Type status:** Other material. **Occurrence:** catalogNumber: A1074; recordedBy: L. Fortini; individualCount: 1; sex: female; lifeStage: adult; occurrenceID: B350D8CC-3DE8-5ECD-AF3C-9C89399D5648; **Taxon:** scientificName: Andrena (Biareolina) lagopus Latreille, 1809; order: Hymenoptera; family: Andrenidae; genus: Andrena; subgenus: Biareolina; specificEpithet: lagopus; scientificNameAuthorship: Latreille, 1809; **Location:** country: Italy; countryCode: IT; stateProvince: Roma; locality: Riserva Naturale Tenuta dei Massimi 1; decimalLatitude: 41.8532859; decimalLongitude: 12.3842322; geodeticDatum: WGS84; coordinatePrecision: 0.0002; **Identification:** identifiedBy: M. Mei; **Event:** eventDate: 2022-04-23; **Record Level:** collectionCode: UR3
- **Type status:** Other material. **Occurrence:** catalogNumber: A1076, A1077; recordedBy: L. Fortini; individualCount: 2; sex: females; lifeStage: adult; occurrenceID: 1E3C1FF0-C9BD-55EE-85D3-C60AC59E5908; **Taxon:** scientificName: Andrena (Biareolina) lagopus Latreille, 1809; order: Hymenoptera; family: Andrenidae; genus: Andrena; subgenus: Biareolina; specificEpithet: lagopus; scientificNameAuthorship: Latreille, 1809; **Location:** country: Italy; countryCode: IT; stateProvince: Roma; locality: Riserva Naturale Valle dell'Aniene 2; decimalLatitude: 41.928752; decimalLongitude: 12.5562962; geodeticDatum: WGS84; coordinatePrecision: 0.0002; **Identification:** identifiedBy: M. Mei; **Event:** eventDate: 2022-04-28; **Record Level:** collectionCode: UR3
- **Type status:** Other material. **Occurrence:** catalogNumber: A1081, A1098; recordedBy: L. Fortini; individualCount: 2; sex: 1 male, 1 female; lifeStage: adult; occurrenceID: 0C5985F3-EB60-5686-BC5C-61CB186AE523; **Taxon:** scientificName: Andrena (Biareolina) lagopus Latreille, 1809; order: Hymenoptera; family: Andrenidae; genus: Andrena; subgenus: Biareolina; specificEpithet: lagopus; scientificNameAuthorship: Latreille, 1809; **Location:** country: Italy; countryCode: IT; stateProvince: Roma; locality: Riserva Naturale Valle dei Casali 1; decimalLatitude: 41.8710627; decimalLongitude: 12.4336809; geodeticDatum: WGS84; coordinatePrecision: 0.0002; **Identification:** identifiedBy: M. Mei; **Event:** eventDate: 2022-04-07; **Record Level:** collectionCode: UR3
- **Type status:** Other material. **Occurrence:** catalogNumber: A1083; recordedBy: L. Fortini; individualCount: 1; sex: female; lifeStage: adult; occurrenceID: 968E8676-9D5D-541D-BE21-ABB9F12FBAFE; **Taxon:** scientificName: Andrena (Biareolina) lagopus Latreille, 1809; order: Hymenoptera; family: Andrenidae; genus: Andrena; subgenus: Biareolina; specificEpithet: lagopus; scientificNameAuthorship: Latreille, 1809; **Location:** country: Italy; countryCode: IT; stateProvince: Roma; locality: Riserva Naturale Valle dei Casali 2; decimalLatitude: 41.8596887; decimalLongitude: 12.4355075; geodeticDatum: WGS84; coordinatePrecision: 0.0002; **Identification:** identifiedBy: M. Mei; **Event:** eventDate: 2022-04-10; **Record Level:** collectionCode: UR3

#### Andrena limata

Smith, 1853

30C850E6-D152-53CD-B4F3-6189544F4DF3

##### Materials

- **Type status:** Other material. **Occurrence:** catalogNumber: A0815; recordedBy: L. Fortini; individualCount: 1; sex: female; lifeStage: adult; occurrenceID: 9A8F0963-8003-56F1-9766-A05015129DBA; **Taxon:** scientificName: Andrena (Melandrena) limata Smith, 1853; order: Hymenoptera; family: Andrenidae; genus: Andrena; subgenus: Melandrena; specificEpithet: limata; scientificNameAuthorship: Smith, 1853; **Location:** country: Italy; countryCode: IT; stateProvince: Roma; locality: Riserva Naturale Tenuta dei Massimi 2; decimalLatitude: 41.8316516; decimalLongitude: 12.3999927; geodeticDatum: WGS84; coordinatePrecision: 0.0002; **Identification:** identifiedBy: M. Mei; **Event:** eventDate: 2022-05-04; **Record Level:** collectionCode: UR3
- **Type status:** Other material. **Occurrence:** catalogNumber: A0816; recordedBy: L. Fortini; individualCount: 1; sex: female; lifeStage: adult; occurrenceID: F96E62DF-F265-5E0B-A07B-89401B4A6433; **Taxon:** scientificName: Andrena (Melandrena) limata Smith, 1853; order: Hymenoptera; family: Andrenidae; genus: Andrena; subgenus: Melandrena; specificEpithet: limata; scientificNameAuthorship: Smith, 1853; **Location:** country: Italy; countryCode: IT; stateProvince: Roma; locality: Riserva Naturale dell'Acquafredda; decimalLatitude: 41.8928408; decimalLongitude: 12.39932; geodeticDatum: WGS84; coordinatePrecision: 0.0002; **Identification:** identifiedBy: M. Mei; **Event:** eventDate: 2022-07-12; **Record Level:** collectionCode: UR3
- **Type status:** Other material. **Occurrence:** catalogNumber: A0817; recordedBy: L. Fortini; individualCount: 1; sex: female; lifeStage: adult; occurrenceID: 9F8CA8D5-25A7-5F54-BFA0-A2C18CD49937; **Taxon:** scientificName: Andrena (Melandrena) limata Smith, 1853; order: Hymenoptera; family: Andrenidae; genus: Andrena; subgenus: Melandrena; specificEpithet: limata; scientificNameAuthorship: Smith, 1853; **Location:** country: Italy; countryCode: IT; stateProvince: Roma; locality: Riserva Naturale Tenuta dei Massimi 1; decimalLatitude: 41.8532859; decimalLongitude: 12.3842322; geodeticDatum: WGS84; coordinatePrecision: 0.0002; **Identification:** identifiedBy: M. Mei; **Event:** eventDate: 2022-04-23; **Record Level:** collectionCode: UR3
- **Type status:** Other material. **Occurrence:** catalogNumber: A0818; recordedBy: L. Fortini; individualCount: 1; sex: female; lifeStage: adult; occurrenceID: 75126322-67E3-544A-A748-8DED48C1F998; **Taxon:** scientificName: Andrena (Melandrena) limata Smith, 1853; order: Hymenoptera; family: Andrenidae; genus: Andrena; subgenus: Melandrena; specificEpithet: limata; scientificNameAuthorship: Smith, 1853; **Location:** country: Italy; countryCode: IT; stateProvince: Roma; locality: Riserva Naturale Valle dei Casali 2; decimalLatitude: 41.8596887; decimalLongitude: 12.4355075; geodeticDatum: WGS84; coordinatePrecision: 0.0002; **Identification:** identifiedBy: M. Mei; **Event:** eventDate: 2022-04-10; **Record Level:** collectionCode: UR3

#### Andrena livens

Pérez, 1895

2264FC77-92D9-5ECA-9A1A-141C2CAF54A3

##### Materials

- **Type status:** Other material. **Occurrence:** catalogNumber: A0966; recordedBy: L. Fortini; individualCount: 1; sex: male; lifeStage: adult; occurrenceID: 8E6C4A1F-378B-5F8C-A5CB-BBEB6E552B19; **Taxon:** scientificName: Andrena (Chlorandrena) livens Pérez, 1895; order: Hymenoptera; family: Andrenidae; genus: Andrena; subgenus: Chlorandrena; specificEpithet: livens; scientificNameAuthorship: Pérez, 1895; **Location:** country: Italy; countryCode: IT; stateProvince: Roma; locality: Riserva Naturale Tenuta dei Massimi 2; decimalLatitude: 41.8316516; decimalLongitude: 12.3999927; geodeticDatum: WGS84; coordinatePrecision: 0.0002; **Identification:** identifiedBy: M. Mei; **Event:** eventDate: 2022-05-04; **Record Level:** collectionCode: UR3

#### Andrena minutula

(Kirby, 1802)

DD54E718-BEDA-57C0-A965-11BDEE244A2E

##### Materials

- **Type status:** Other material. **Occurrence:** catalogNumber: A0895; recordedBy: L. Fortini; individualCount: 1; sex: male; lifeStage: adult; occurrenceID: 15B05173-EAF6-5306-B221-8038E6656ADC; **Taxon:** scientificName: Andrena (Micrandrena) minutula (Kirby, 1802); order: Hymenoptera; family: Andrenidae; genus: Andrena; subgenus: Micrandrena; specificEpithet: minutula; scientificNameAuthorship: (Kirby, 1802); **Location:** country: Italy; countryCode: IT; stateProvince: Roma; locality: Riserva Naturale Valle dell'Aniene 2; decimalLatitude: 41.928752; decimalLongitude: 12.5562962; geodeticDatum: WGS84; coordinatePrecision: 0.0002; **Identification:** identifiedBy: M. Mei; **Event:** eventDate: 2022-06-05; **Record Level:** collectionCode: UR3
- **Type status:** Other material. **Occurrence:** catalogNumber: A0963; recordedBy: L. Fortini; individualCount: 1; sex: male; lifeStage: adult; occurrenceID: DF1DB1D9-0F5C-5ABC-9B86-FC9386332E01; **Taxon:** scientificName: Andrena (Micrandrena) minutula (Kirby, 1802); order: Hymenoptera; family: Andrenidae; genus: Andrena; subgenus: Micrandrena; specificEpithet: minutula; scientificNameAuthorship: (Kirby, 1802); **Location:** country: Italy; countryCode: IT; stateProvince: Roma; locality: Riserva Naturale di Monte Mario; decimalLatitude: 41.9386215; decimalLongitude: 12.4546223; geodeticDatum: WGS84; coordinatePrecision: 0.0002; **Identification:** identifiedBy: M. Mei; **Event:** eventDate: 2022-05-20; **Record Level:** collectionCode: UR3
- **Type status:** Other material. **Occurrence:** catalogNumber: A1219; recordedBy: L. Fortini; individualCount: 1; sex: male; lifeStage: adult; occurrenceID: E3F8E256-E753-56E7-9499-4C4C5B355C18; **Taxon:** scientificName: Andrena (Micrandrena) minutula (Kirby, 1802); order: Hymenoptera; family: Andrenidae; genus: Andrena; subgenus: Micrandrena; specificEpithet: minutula; scientificNameAuthorship: (Kirby, 1802); **Location:** country: Italy; countryCode: IT; stateProvince: Roma; locality: Riserva Naturale dell'Insugherata 3; decimalLatitude: 41.9644829; decimalLongitude: 12.436101; geodeticDatum: WGS84; coordinatePrecision: 0.0002; **Identification:** identifiedBy: M. Mei; **Event:** eventDate: 2022-04-15; **Record Level:** collectionCode: UR3
- **Type status:** Other material. **Occurrence:** catalogNumber: A1231; recordedBy: L. Fortini; individualCount: 1; sex: male; lifeStage: adult; occurrenceID: 993B6BB5-3420-52A2-AA3E-992D9B93FC57; **Taxon:** scientificName: Andrena (Micrandrena) minutula (Kirby, 1802); order: Hymenoptera; family: Andrenidae; genus: Andrena; subgenus: Micrandrena; specificEpithet: minutula; scientificNameAuthorship: (Kirby, 1802); **Location:** country: Italy; countryCode: IT; stateProvince: Roma; locality: Riserva Naturale Valle dei Casali 1; decimalLatitude: 41.8710627; decimalLongitude: 12.4336809; geodeticDatum: WGS84; coordinatePrecision: 0.0002; **Identification:** identifiedBy: M. Mei; **Event:** eventDate: 2022-05-14; **Record Level:** collectionCode: UR3

#### Andrena morio

Brullé, 1832

A1D29955-663A-5BF6-9043-D222E3139C5D

##### Materials

- **Type status:** Other material. **Occurrence:** catalogNumber: A1066; recordedBy: L. Fortini; individualCount: 1; sex: female; lifeStage: adult; occurrenceID: 81B7CE2E-A708-5BF8-AC7F-60F6426A3B79; **Taxon:** scientificName: Andrena (Melandrena) morio Brullé, 1832; order: Hymenoptera; family: Andrenidae; genus: Andrena; subgenus: Melandrena; specificEpithet: morio; scientificNameAuthorship: Brullé, 1832; **Location:** country: Italy; countryCode: IT; stateProvince: Roma; locality: Riserva Naturale di Monte Mario; decimalLatitude: 41.9386215; decimalLongitude: 12.4546223; geodeticDatum: WGS84; coordinatePrecision: 0.0002; **Identification:** identifiedBy: M. Mei; **Event:** eventDate: 2022-04-20; **Record Level:** collectionCode: UR3

#### Andrena nana

(Kirby, 1802)

8B7B3B17-5950-5B28-890A-4F8C6ECE4AFD

##### Materials

- **Type status:** Other material. **Occurrence:** catalogNumber: A0925, A0926; recordedBy: L. Fortini; individualCount: 2; sex: females; lifeStage: adult; occurrenceID: 935281FF-8725-5297-A44C-99B051915D6D; **Taxon:** scientificName: Andrena (Micrandrena) nana (Kirby, 1802); order: Hymenoptera; family: Andrenidae; genus: Andrena; subgenus: Micrandrena; specificEpithet: nana; scientificNameAuthorship: (Kirby, 1802); **Location:** country: Italy; countryCode: IT; stateProvince: Roma; locality: Riserva Naturale Laurentino-Acqua Acetosa; decimalLatitude: 41.8079275; decimalLongitude: 12.4685548; geodeticDatum: WGS84; coordinatePrecision: 0.0002; **Identification:** identifiedBy: M. Mei; **Event:** eventDate: 2022-06-16; **Record Level:** collectionCode: UR3
- **Type status:** Other material. **Occurrence:** catalogNumber: A0961; recordedBy: L. Fortini; individualCount: 1; sex: male; lifeStage: adult; occurrenceID: B03EBA66-2F4B-50F1-9055-E18940C5FE8D; **Taxon:** scientificName: Andrena (Micrandrena) nana (Kirby, 1802); order: Hymenoptera; family: Andrenidae; genus: Andrena; subgenus: Micrandrena; specificEpithet: nana; scientificNameAuthorship: (Kirby, 1802); **Location:** country: Italy; countryCode: IT; stateProvince: Roma; locality: Riserva Naturale Valle dell'Aniene 1; decimalLatitude: 41.9345179; decimalLongitude: 12.5453096; geodeticDatum: WGS84; coordinatePrecision: 0.0002; **Identification:** identifiedBy: M. Mei; **Event:** eventDate: 2022-06-05; **Record Level:** collectionCode: UR3
- **Type status:** Other material. **Occurrence:** catalogNumber: A0979; recordedBy: L. Fortini; individualCount: 1; sex: female; lifeStage: adult; occurrenceID: DBC5F89C-79E5-57D6-9840-904B7D28B7F0; **Taxon:** scientificName: Andrena (Micrandrena) nana (Kirby, 1802); order: Hymenoptera; family: Andrenidae; genus: Andrena; subgenus: Micrandrena; specificEpithet: nana; scientificNameAuthorship: (Kirby, 1802); **Location:** country: Italy; countryCode: IT; stateProvince: Roma; locality: Riserva Naturale Valle dell'Aniene 1; decimalLatitude: 41.9345179; decimalLongitude: 12.5453096; geodeticDatum: WGS84; coordinatePrecision: 0.0002; **Identification:** identifiedBy: M. Mei; **Event:** eventDate: 2022-04-28; **Record Level:** collectionCode: UR3
- **Type status:** Other material. **Occurrence:** catalogNumber: A1227; recordedBy: L. Fortini; individualCount: 1; sex: female; lifeStage: adult; occurrenceID: B1EF3131-8804-5612-B284-571B32D10407; **Taxon:** scientificName: Andrena (Micrandrena) nana (Kirby, 1802); order: Hymenoptera; family: Andrenidae; genus: Andrena; subgenus: Micrandrena; specificEpithet: nana; scientificNameAuthorship: (Kirby, 1802); **Location:** country: Italy; countryCode: IT; stateProvince: Roma; locality: Riserva Naturale Valle dei Casali 2; decimalLatitude: 41.8596887; decimalLongitude: 12.4355075; geodeticDatum: WGS84; coordinatePrecision: 0.0002; **Identification:** identifiedBy: M. Mei; **Event:** eventDate: 2022-04-10; **Record Level:** collectionCode: UR3

#### Andrena nigroaenea

(Kirby, 1802)

ED9F7C12-73ED-590C-8AA4-9745D04F54A2

##### Materials

- **Type status:** Other material. **Occurrence:** catalogNumber: A0932; recordedBy: L. Fortini; individualCount: 1; sex: female; lifeStage: adult; occurrenceID: 177B4F9D-35B2-5EC4-AD3C-B8342A047F7F; **Taxon:** scientificName: Andrena (Melandrena) nigroaenea (Kirby, 1802); order: Hymenoptera; family: Andrenidae; genus: Andrena; subgenus: Melandrena; specificEpithet: nigroaenea; scientificNameAuthorship: (Kirby, 1802); **Location:** country: Italy; countryCode: IT; stateProvince: Roma; locality: Riserva Naturale dell'Insugherata 2; decimalLatitude: 41.9599247; decimalLongitude: 12.433852; geodeticDatum: WGS84; coordinatePrecision: 0.0002; **Identification:** identifiedBy: M. Mei; **Event:** eventDate: 2022-06-24; **Record Level:** collectionCode: UR3
- **Type status:** Other material. **Occurrence:** catalogNumber: A0933; recordedBy: L. Fortini; individualCount: 1; sex: female; lifeStage: adult; occurrenceID: C7BFEE58-3DA0-530F-B55C-152362E398DB; **Taxon:** scientificName: Andrena (Melandrena) nigroaenea (Kirby, 1802); order: Hymenoptera; family: Andrenidae; genus: Andrena; subgenus: Melandrena; specificEpithet: nigroaenea; scientificNameAuthorship: (Kirby, 1802); **Location:** country: Italy; countryCode: IT; stateProvince: Roma; locality: Riserva Naturale Valle dell'Aniene 2; decimalLatitude: 41.928752; decimalLongitude: 12.5562962; geodeticDatum: WGS84; coordinatePrecision: 0.0002; **Identification:** identifiedBy: M. Mei; **Event:** eventDate: 2022-06-05; **Record Level:** collectionCode: UR3
- **Type status:** Other material. **Occurrence:** catalogNumber: A0934; recordedBy: L. Fortini; individualCount: 1; sex: female; lifeStage: adult; occurrenceID: FFF6957C-C72F-5BAF-B34C-A324B5589747; **Taxon:** scientificName: Andrena (Melandrena) nigroaenea (Kirby, 1802); order: Hymenoptera; family: Andrenidae; genus: Andrena; subgenus: Melandrena; specificEpithet: nigroaenea; scientificNameAuthorship: (Kirby, 1802); **Location:** country: Italy; countryCode: IT; stateProvince: Roma; locality: Riserva Regionale dell'Appia Antica 1; decimalLatitude: 41.8623941; decimalLongitude: 12.524863; geodeticDatum: WGS84; coordinatePrecision: 0.0002; **Identification:** identifiedBy: M. Mei; **Event:** eventDate: 2022-06-12; **Record Level:** collectionCode: UR3
- **Type status:** Other material. **Occurrence:** catalogNumber: A0935; recordedBy: L. Fortini; individualCount: 1; sex: female; lifeStage: adult; occurrenceID: B0427C5E-B371-561E-B74C-636B75C27FA4; **Taxon:** scientificName: Andrena (Melandrena) nigroaenea (Kirby, 1802); order: Hymenoptera; family: Andrenidae; genus: Andrena; subgenus: Melandrena; specificEpithet: nigroaenea; scientificNameAuthorship: (Kirby, 1802); **Location:** country: Italy; countryCode: IT; stateProvince: Roma; locality: Riserva Naturale di Monte Mario; decimalLatitude: 41.9386215; decimalLongitude: 12.4546223; geodeticDatum: WGS84; coordinatePrecision: 0.0002; **Identification:** identifiedBy: M. Mei; **Event:** eventDate: 2022-05-20; **Record Level:** collectionCode: UR3
- **Type status:** Other material. **Occurrence:** catalogNumber: A0952; recordedBy: L. Fortini; individualCount: 1; sex: male; lifeStage: adult; occurrenceID: 5FBC25FE-FCBD-5867-BF9D-532BEC078B2E; **Taxon:** scientificName: Andrena (Melandrena) nigroaenea (Kirby, 1802); order: Hymenoptera; family: Andrenidae; genus: Andrena; subgenus: Melandrena; specificEpithet: nigroaenea; scientificNameAuthorship: (Kirby, 1802); **Location:** country: Italy; countryCode: IT; stateProvince: Roma; locality: Riserva Regionale dell'Appia Antica 3; decimalLatitude: 41.8298456; decimalLongitude: 12.5432538; geodeticDatum: WGS84; coordinatePrecision: 0.0002; **Identification:** identifiedBy: M. Mei; **Event:** eventDate: 2022-05-24; **Record Level:** collectionCode: UR3

#### Andrena nigroolivacea

Dours, 1873

54423317-6B5F-5639-B127-37697CE875E0

##### Materials

- **Type status:** Other material. **Occurrence:** catalogNumber: A0832, A0835, A0897, A0998, A0999; recordedBy: L. Fortini; individualCount: 5; sex: 3 males, 2 females; lifeStage: adult; occurrenceID: 498BF3CB-C007-5980-8069-78E38A6533D5; **Taxon:** scientificName: Andrena (Chlorandrena) nigroolivacea Dours, 1873; order: Hymenoptera; family: Andrenidae; genus: Andrena; subgenus: Chlorandrena; specificEpithet: nigroolivacea; scientificNameAuthorship: Dours, 1873; **Location:** country: Italy; countryCode: IT; stateProvince: Roma; locality: Riserva Naturale Valle dei Casali 1; decimalLatitude: 41.8710627; decimalLongitude: 12.4336809; geodeticDatum: WGS84; coordinatePrecision: 0.0002; **Identification:** identifiedBy: M. Mei; **Event:** eventDate: 2022-04-07; **Record Level:** collectionCode: UR3
- **Type status:** Other material. **Occurrence:** catalogNumber: A0904, A0953, A0954, A0997; recordedBy: L. Fortini; individualCount: 4; sex: 3 males, 1 female; lifeStage: adult; occurrenceID: 92FAECD9-BEF6-53D7-B97E-15746E9992F3; **Taxon:** scientificName: Andrena (Chlorandrena) nigroolivacea Dours, 1873; order: Hymenoptera; family: Andrenidae; genus: Andrena; subgenus: Chlorandrena; specificEpithet: nigroolivacea; scientificNameAuthorship: Dours, 1873; **Location:** country: Italy; countryCode: IT; stateProvince: Roma; locality: Riserva Naturale Laurentino-Acqua Acetosa; decimalLatitude: 41.8079275; decimalLongitude: 12.4685548; geodeticDatum: WGS84; coordinatePrecision: 0.0002; **Identification:** identifiedBy: M. Mei; **Event:** eventDate: 2022-04-12; **Record Level:** collectionCode: UR3
- **Type status:** Other material. **Occurrence:** catalogNumber: A0861, A0862, A0863, A0917, A0924; recordedBy: L. Fortini; individualCount: 5; sex: females; lifeStage: adult; occurrenceID: 8C1403ED-83E3-5E5E-B3F4-342D00E1AF45; **Taxon:** scientificName: Andrena (Chlorandrena) nigroolivacea Dours, 1873; order: Hymenoptera; family: Andrenidae; genus: Andrena; subgenus: Chlorandrena; specificEpithet: nigroolivacea; scientificNameAuthorship: Dours, 1873; **Location:** country: Italy; countryCode: IT; stateProvince: Roma; locality: Riserva Naturale di Monte Mario; decimalLatitude: 41.9386215; decimalLongitude: 12.4546223; geodeticDatum: WGS84; coordinatePrecision: 0.0002; **Identification:** identifiedBy: M. Mei; **Event:** eventDate: 2022-04-20; **Record Level:** collectionCode: UR3
- **Type status:** Other material. **Occurrence:** catalogNumber: A0903, A0910; recordedBy: L. Fortini; individualCount: 2; sex: females; lifeStage: adult; occurrenceID: 6026CFED-9E2F-54B1-B66C-7055DF2471CF; **Taxon:** scientificName: Andrena (Chlorandrena) nigroolivacea Dours, 1873; order: Hymenoptera; family: Andrenidae; genus: Andrena; subgenus: Chlorandrena; specificEpithet: nigroolivacea; scientificNameAuthorship: Dours, 1873; **Location:** country: Italy; countryCode: IT; stateProvince: Roma; locality: Riserva Naturale Tenuta dei Massimi 1; decimalLatitude: 41.8532859; decimalLongitude: 12.3842322; geodeticDatum: WGS84; coordinatePrecision: 0.0002; **Identification:** identifiedBy: M. Mei; **Event:** eventDate: 2022-04-23; **Record Level:** collectionCode: UR3
- **Type status:** Other material. **Occurrence:** catalogNumber: A2016, A2019; recordedBy: L. Fortini; individualCount: 2; sex: females; lifeStage: adult; occurrenceID: FDE0A39C-F09C-5FF5-B4EB-17A1F5F87225; **Taxon:** scientificName: Andrena (Chlorandrena) nigroolivacea Dours, 1873; order: Hymenoptera; family: Andrenidae; genus: Andrena; subgenus: Chlorandrena; specificEpithet: nigroolivacea; scientificNameAuthorship: Dours, 1873; **Location:** country: Italy; countryCode: IT; stateProvince: Roma; locality: Riserva Regionale dell'Appia Antica 2; decimalLatitude: 41.8402564; decimalLongitude: 12.532773; geodeticDatum: WGS84; coordinatePrecision: 0.0002; **Identification:** identifiedBy: M. Mei; **Event:** eventDate: 2022-04-25; **Record Level:** collectionCode: UR3
- **Type status:** Other material. **Occurrence:** catalogNumber: A0833, A0834, A0898, A0899, A0901, A0902, A0908, A0913, A0922, A0955; recordedBy: L. Fortini; individualCount: 10; sex: 1 male, 9 females; lifeStage: adult; occurrenceID: B6B3E44E-B016-536C-BE6A-729BD3AD4778; **Taxon:** scientificName: Andrena (Chlorandrena) nigroolivacea Dours, 1873; order: Hymenoptera; family: Andrenidae; genus: Andrena; subgenus: Chlorandrena; specificEpithet: nigroolivacea; scientificNameAuthorship: Dours, 1873; **Location:** country: Italy; countryCode: IT; stateProvince: Roma; locality: Riserva Naturale Tenuta dei Massimi 2; decimalLatitude: 41.8316516; decimalLongitude: 12.3999927; geodeticDatum: WGS84; coordinatePrecision: 0.0002; **Identification:** identifiedBy: M. Mei; **Event:** eventDate: 2022-05-04; **Record Level:** collectionCode: UR3
- **Type status:** Other material. **Occurrence:** catalogNumber: A0915, A0916; recordedBy: L. Fortini; individualCount: 2; sex: females; lifeStage: adult; occurrenceID: F79B8F76-9F75-5819-821E-27AEB2C03EB7; **Taxon:** scientificName: Andrena (Chlorandrena) nigroolivacea Dours, 1873; order: Hymenoptera; family: Andrenidae; genus: Andrena; subgenus: Chlorandrena; specificEpithet: nigroolivacea; scientificNameAuthorship: Dours, 1873; **Location:** country: Italy; countryCode: IT; stateProvince: Roma; locality: Riserva Naturale di Monte Mario; decimalLatitude: 41.9386215; decimalLongitude: 12.4546223; geodeticDatum: WGS84; coordinatePrecision: 0.0002; **Identification:** identifiedBy: M. Mei; **Event:** eventDate: 2022-05-20; **Record Level:** collectionCode: UR3

#### Andrena ovata

Schenck, 1853

8BE95420-ED50-583C-8B07-B44F8B1E3C71

##### Materials

- **Type status:** Other material. **Occurrence:** catalogNumber: A0850; recordedBy: L. Fortini; individualCount: 1; sex: female; lifeStage: adult; occurrenceID: E028B8CB-B5AE-56C9-9B02-14AAB4694F66; **Taxon:** scientificName: Andrena (Taeniandrena) ovata Schenck, 1853; order: Hymenoptera; family: Andrenidae; genus: Andrena; subgenus: Taeniandrena; specificEpithet: ovata; scientificNameAuthorship: Schenck, 1853; **Location:** country: Italy; countryCode: IT; stateProvince: Roma; locality: Riserva Naturale Valle dell'Aniene 1; decimalLatitude: 41.9345179; decimalLongitude: 12.5453096; geodeticDatum: WGS84; coordinatePrecision: 0.0002; **Identification:** identifiedBy: M. Mei; **Event:** eventDate: 2022-06-05; **Record Level:** collectionCode: UR3
- **Type status:** Other material. **Occurrence:** catalogNumber: A0851, A0852, A0853, A0854, A0855, A0856, A0857, A0858, A0859; recordedBy: L. Fortini; individualCount: 9; sex: females; lifeStage: adult; occurrenceID: 1EF4B012-4A61-5E86-A30A-144983A63424; **Taxon:** scientificName: Andrena (Taeniandrena) ovata Schenck, 1853; order: Hymenoptera; family: Andrenidae; genus: Andrena; subgenus: Taeniandrena; specificEpithet: ovata; scientificNameAuthorship: Schenck, 1853; **Location:** country: Italy; countryCode: IT; stateProvince: Roma; locality: Riserva Naturale dell'Insugherata 2; decimalLatitude: 41.9599247; decimalLongitude: 12.433852; geodeticDatum: WGS84; coordinatePrecision: 0.0002; **Identification:** identifiedBy: M. Mei; **Event:** eventDate: 2022-05-27; **Record Level:** collectionCode: UR3

#### Andrena pandellei

Pérez, 1895

F9ED2F41-74A4-531C-A7F4-921A75FE687A

##### Materials

- **Type status:** Other material. **Occurrence:** catalogNumber: A0877; recordedBy: L. Fortini; individualCount: 1; sex: female; lifeStage: adult; occurrenceID: BEDF02E8-FBB7-5DE0-BA28-3122199EAEE5; **Taxon:** scientificName: Andrena (Lepidandrena) pandellei Pérez, 1895; order: Hymenoptera; family: Andrenidae; genus: Andrena; subgenus: Lepidandrena; specificEpithet: pandellei; scientificNameAuthorship: Pérez, 1895; **Location:** country: Italy; countryCode: IT; stateProvince: Roma; locality: Riserva Regionale dell'Appia Antica 2; decimalLatitude: 41.8402564; decimalLongitude: 12.532773; geodeticDatum: WGS84; coordinatePrecision: 0.0002; **Identification:** identifiedBy: M. Mei; **Event:** eventDate: 2022-05-24; **Record Level:** collectionCode: UR3

#### Andrena pellucens

Pérez, 1895

51154857-9A8C-5FCD-846B-CC01D2BED5E5

##### Materials

- **Type status:** Other material. **Occurrence:** catalogNumber: A1086, A1087, A1088, A1089, A1090, A1092; recordedBy: L. Fortini; individualCount: 6; sex: females; lifeStage: adult; occurrenceID: B3DC8BE5-62EE-5395-B4BD-2022D3C16474; **Taxon:** scientificName: Andrena (Margandrena) pellucens Pérez, 1895; order: Hymenoptera; family: Andrenidae; genus: Andrena; subgenus: Margandrena; specificEpithet: pellucens; scientificNameAuthorship: Pérez, 1895; **Location:** country: Italy; countryCode: IT; stateProvince: Roma; locality: Riserva Regionale dell'Appia Antica 2; decimalLatitude: 41.8402564; decimalLongitude: 12.532773; geodeticDatum: WGS84; coordinatePrecision: 0.0002; **Identification:** identifiedBy: M. Mei; **Event:** eventDate: 2022-10-01; **Record Level:** collectionCode: UR3
- **Type status:** Other material. **Occurrence:** catalogNumber: A1091; recordedBy: L. Fortini; individualCount: 1; sex: female; lifeStage: adult; occurrenceID: 15D2D054-45A7-5EB2-A2E9-7DDD439C2382; **Taxon:** scientificName: Andrena (Margandrena) pellucens Pérez, 1895; order: Hymenoptera; family: Andrenidae; genus: Andrena; subgenus: Margandrena; specificEpithet: pellucens; scientificNameAuthorship: Pérez, 1895; **Location:** country: Italy; countryCode: IT; stateProvince: Roma; locality: Riserva Naturale dell'Insugherata 2; decimalLatitude: 41.9599247; decimalLongitude: 12.433852; geodeticDatum: WGS84; coordinatePrecision: 0.0002; **Identification:** identifiedBy: M. Mei; **Event:** eventDate: 2022-10-01; **Record Level:** collectionCode: UR3
- **Type status:** Other material. **Occurrence:** catalogNumber: A1093; recordedBy: L. Fortini; individualCount: 1; sex: female; lifeStage: adult; occurrenceID: 643877CA-22AF-5386-A77F-C58C09521C46; **Taxon:** scientificName: Andrena (Margandrena) pellucens Pérez, 1895; order: Hymenoptera; family: Andrenidae; genus: Andrena; subgenus: Margandrena; specificEpithet: pellucens; scientificNameAuthorship: Pérez, 1895; **Location:** country: Italy; countryCode: IT; stateProvince: Roma; locality: Riserva Naturale dell'Insugherata 1; decimalLatitude: 41.9555045; decimalLongitude: 12.4292321; geodeticDatum: WGS84; coordinatePrecision: 0.0002; **Identification:** identifiedBy: M. Mei; **Event:** eventDate: 2022-10-04; **Record Level:** collectionCode: UR3

#### Andrena pilipes

Fabricius, 1781

4BB89F81-8CB8-5C72-9384-6C2DFCCC4C82

##### Materials

- **Type status:** Other material. **Occurrence:** catalogNumber: A1067; recordedBy: L. Fortini; individualCount: 1; sex: female; lifeStage: adult; occurrenceID: 3B3DCE23-B47E-5828-935A-6D72A93F5F22; **Taxon:** scientificName: Andrena (Plastandrena) pilipes Fabricius, 1781; order: Hymenoptera; family: Andrenidae; genus: Andrena; subgenus: Plastandrena; specificEpithet: pilipes; scientificNameAuthorship: Fabricius, 1781; **Location:** country: Italy; countryCode: IT; stateProvince: Roma; locality: Riserva Naturale Tenuta dei Massimi 2; decimalLatitude: 41.8316516; decimalLongitude: 12.3999927; geodeticDatum: WGS84; coordinatePrecision: 0.0002; **Identification:** identifiedBy: M. Mei; **Event:** eventDate: 2022-06-01; **Record Level:** collectionCode: UR3
- **Type status:** Other material. **Occurrence:** catalogNumber: A1068; recordedBy: L. Fortini; individualCount: 1; sex: female; lifeStage: adult; occurrenceID: 0B6256CF-216F-5422-A533-1225EC0B77AE; **Taxon:** scientificName: Andrena (Plastandrena) pilipes Fabricius, 1781; order: Hymenoptera; family: Andrenidae; genus: Andrena; subgenus: Plastandrena; specificEpithet: pilipes; scientificNameAuthorship: Fabricius, 1781; **Location:** country: Italy; countryCode: IT; stateProvince: Roma; locality: Riserva Naturale Tenuta dei Massimi 1; decimalLatitude: 41.8532859; decimalLongitude: 12.3842322; geodeticDatum: WGS84; coordinatePrecision: 0.0002; **Identification:** identifiedBy: M. Mei; **Event:** eventDate: 2022-04-23; **Record Level:** collectionCode: UR3
- **Type status:** Other material. **Occurrence:** catalogNumber: A1069; recordedBy: L. Fortini; individualCount: 1; sex: female; lifeStage: adult; occurrenceID: 6E4C2C63-A74E-5E66-9C51-0927BC3B4D18; **Taxon:** scientificName: Andrena (Plastandrena) pilipes Fabricius, 1781; order: Hymenoptera; family: Andrenidae; genus: Andrena; subgenus: Plastandrena; specificEpithet: pilipes; scientificNameAuthorship: Fabricius, 1781; **Location:** country: Italy; countryCode: IT; stateProvince: Roma; locality: Riserva Naturale dell'Acquafredda; decimalLatitude: 41.8928408; decimalLongitude: 12.39932; geodeticDatum: WGS84; coordinatePrecision: 0.0002; **Identification:** identifiedBy: M. Mei; **Event:** eventDate: 2022-04-13; **Record Level:** collectionCode: UR3
- **Type status:** Other material. **Occurrence:** catalogNumber: A1990, A1992, A1993, A1994; recordedBy: L. Fortini; individualCount: 4; sex: female; lifeStage: adult; occurrenceID: 9C6D71E4-E35E-5A22-8A0F-8F795400A528; **Taxon:** scientificName: Andrena (Plastandrena) pilipes Fabricius, 1781; order: Hymenoptera; family: Andrenidae; genus: Andrena; subgenus: Plastandrena; specificEpithet: pilipes; scientificNameAuthorship: Fabricius, 1781; **Location:** country: Italy; countryCode: IT; stateProvince: Roma; locality: Riserva Regionale dell'Appia Antica 1; decimalLatitude: 41.8623941; decimalLongitude: 12.524863; geodeticDatum: WGS84; coordinatePrecision: 0.0002; **Identification:** identifiedBy: M. Mei; **Event:** eventDate: 2022-04-19; **Record Level:** collectionCode: UR3

#### Andrena pusilla

Pérez, 1903

6EFA2D8C-A082-5C82-9C31-F9D5E2EB173C

##### Materials

- **Type status:** Other material. **Occurrence:** catalogNumber: A1198, A1203, A1204, A1207, A1218, A1229, A1237; recordedBy: L. Fortini; individualCount: 7; sex: males; lifeStage: adult; occurrenceID: 93367599-B2C4-53F2-8A90-E297895F894B; **Taxon:** scientificName: Andrena (Micrandrena) pusilla Pérez, 1903; order: Hymenoptera; family: Andrenidae; genus: Andrena; subgenus: Micrandrena; specificEpithet: pusilla; scientificNameAuthorship: Pérez, 1903; **Location:** country: Italy; countryCode: IT; stateProvince: Roma; locality: Riserva Naturale Valle dei Casali 1; decimalLatitude: 41.8710627; decimalLongitude: 12.4336809; geodeticDatum: WGS84; coordinatePrecision: 0.0002; **Identification:** identifiedBy: M. Mei; **Event:** eventDate: 2022-04-07; **Record Level:** collectionCode: UR3
- **Type status:** Other material. **Occurrence:** catalogNumber: A1202, A1230, A1241, A1243; recordedBy: L. Fortini; individualCount: 4; sex: 3 males, 1 female; lifeStage: adult; occurrenceID: BBB1001B-1A04-549D-AA79-D841C3FE29B7; **Taxon:** scientificName: Andrena (Micrandrena) pusilla Pérez, 1903; order: Hymenoptera; family: Andrenidae; genus: Andrena; subgenus: Micrandrena; specificEpithet: pusilla; scientificNameAuthorship: Pérez, 1903; **Location:** country: Italy; countryCode: IT; stateProvince: Roma; locality: Riserva Naturale Valle dei Casali 2; decimalLatitude: 41.8596887; decimalLongitude: 12.4355075; geodeticDatum: WGS84; coordinatePrecision: 0.0002; **Identification:** identifiedBy: M. Mei; **Event:** eventDate: 2022-04-10; **Record Level:** collectionCode: UR3
- **Type status:** Other material. **Occurrence:** catalogNumber: A1201, A1206; recordedBy: L. Fortini; individualCount: 2; sex: 1 male, 1 female; lifeStage: adult; occurrenceID: 62387C69-0099-5465-A286-E355E7198740; **Taxon:** scientificName: Andrena (Micrandrena) pusilla Pérez, 1903; order: Hymenoptera; family: Andrenidae; genus: Andrena; subgenus: Micrandrena; specificEpithet: pusilla; scientificNameAuthorship: Pérez, 1903; **Location:** country: Italy; countryCode: IT; stateProvince: Roma; locality: Riserva Naturale Laurentino-Acqua Acetosa; decimalLatitude: 41.8079275; decimalLongitude: 12.4685548; geodeticDatum: WGS84; coordinatePrecision: 0.0002; **Identification:** identifiedBy: M. Mei; **Event:** eventDate: 2022-04-12; **Record Level:** collectionCode: UR3
- **Type status:** Other material. **Occurrence:** catalogNumber: A0896, A1209, A1224; recordedBy: L. Fortini; individualCount: 3; sex: females; lifeStage: adult; occurrenceID: 9756C2D4-1660-5F03-A661-B684CF8C9946; **Taxon:** scientificName: Andrena (Micrandrena) pusilla Pérez, 1903; order: Hymenoptera; family: Andrenidae; genus: Andrena; subgenus: Micrandrena; specificEpithet: pusilla; scientificNameAuthorship: Pérez, 1903; **Location:** country: Italy; countryCode: IT; stateProvince: Roma; locality: Riserva Naturale dell'Acquafredda; decimalLatitude: 41.8928408; decimalLongitude: 12.39932; geodeticDatum: WGS84; coordinatePrecision: 0.0002; **Identification:** identifiedBy: M. Mei; **Event:** eventDate: 2022-04-13; **Record Level:** collectionCode: UR3
- **Type status:** Other material. **Occurrence:** catalogNumber: A1221, A1228, A1233; recordedBy: L. Fortini; individualCount: 3; sex: 2 males, 1 female; lifeStage: adult; occurrenceID: D680E7A5-E79D-5D66-829C-2280E0795CFF; **Taxon:** scientificName: Andrena (Micrandrena) pusilla Pérez, 1903; order: Hymenoptera; family: Andrenidae; genus: Andrena; subgenus: Micrandrena; specificEpithet: pusilla; scientificNameAuthorship: Pérez, 1903; **Location:** country: Italy; countryCode: IT; stateProvince: Roma; locality: Riserva Naturale dell'Insugherata 3; decimalLatitude: 41.9644829; decimalLongitude: 12.436101; geodeticDatum: WGS84; coordinatePrecision: 0.0002; **Identification:** identifiedBy: M. Mei; **Event:** eventDate: 2022-04-15; **Record Level:** collectionCode: UR3
- **Type status:** Other material. **Occurrence:** catalogNumber: A1235, A1242; recordedBy: L. Fortini; individualCount: 2; sex: females; lifeStage: adult; occurrenceID: DE70BD79-C0F1-57DD-AE60-342BA76E171A; **Taxon:** scientificName: Andrena (Micrandrena) pusilla Pérez, 1903; order: Hymenoptera; family: Andrenidae; genus: Andrena; subgenus: Micrandrena; specificEpithet: pusilla; scientificNameAuthorship: Pérez, 1903; **Location:** country: Italy; countryCode: IT; stateProvince: Roma; locality: Riserva Naturale dell'Insugherata 2; decimalLatitude: 41.9599247; decimalLongitude: 12.433852; geodeticDatum: WGS84; coordinatePrecision: 0.0002; **Identification:** identifiedBy: M. Mei; **Event:** eventDate: 2022-04-15; **Record Level:** collectionCode: UR3
- **Type status:** Other material. **Occurrence:** catalogNumber: A1234; recordedBy: L. Fortini; individualCount: 1; sex: female; lifeStage: adult; occurrenceID: 08768F27-C0F5-506B-8E70-356E8B14D59E; **Taxon:** scientificName: Andrena (Micrandrena) pusilla Pérez, 1903; order: Hymenoptera; family: Andrenidae; genus: Andrena; subgenus: Micrandrena; specificEpithet: pusilla; scientificNameAuthorship: Pérez, 1903; **Location:** country: Italy; countryCode: IT; stateProvince: Roma; locality: Riserva Naturale Tenuta dei Massimi 1; decimalLatitude: 41.8532859; decimalLongitude: 12.3842322; geodeticDatum: WGS84; coordinatePrecision: 0.0002; **Identification:** identifiedBy: M. Mei; **Event:** eventDate: 2022-04-23; **Record Level:** collectionCode: UR3
- **Type status:** Other material. **Occurrence:** catalogNumber: A0964; recordedBy: L. Fortini; individualCount: 1; sex: male; lifeStage: adult; occurrenceID: 13425868-33CF-5E7F-88D2-6730CF3B8DBE; **Taxon:** scientificName: Andrena (Micrandrena) pusilla Pérez, 1903; order: Hymenoptera; family: Andrenidae; genus: Andrena; subgenus: Micrandrena; specificEpithet: pusilla; scientificNameAuthorship: Pérez, 1903; **Location:** country: Italy; countryCode: IT; stateProvince: Roma; locality: Riserva Naturale Laurentino-Acqua Acetosa; decimalLatitude: 41.8079275; decimalLongitude: 12.4685548; geodeticDatum: WGS84; coordinatePrecision: 0.0002; **Identification:** identifiedBy: M. Mei; **Event:** eventDate: 2022-05-12; **Record Level:** collectionCode: UR3
- **Type status:** Other material. **Occurrence:** catalogNumber: A0962; recordedBy: L. Fortini; individualCount: 1; sex: male; lifeStage: adult; occurrenceID: 1E9442B1-DF3A-5E0C-B079-758CBE20C5BB; **Taxon:** scientificName: Andrena (Micrandrena) pusilla Pérez, 1903; order: Hymenoptera; family: Andrenidae; genus: Andrena; subgenus: Micrandrena; specificEpithet: pusilla; scientificNameAuthorship: Pérez, 1903; **Location:** country: Italy; countryCode: IT; stateProvince: Roma; locality: Riserva Naturale Valle dei Casali 1; decimalLatitude: 41.8710627; decimalLongitude: 12.4336809; geodeticDatum: WGS84; coordinatePrecision: 0.0002; **Identification:** identifiedBy: M. Mei; **Event:** eventDate: 2022-05-14; **Record Level:** collectionCode: UR3
- **Type status:** Other material. **Occurrence:** catalogNumber: A1199, A1220, A1232, A1239; recordedBy: L. Fortini; individualCount: 4; sex: females; lifeStage: adult; occurrenceID: 9BBE2538-1E03-5B8E-A8E9-415CB1DC7D9E; **Taxon:** scientificName: Andrena (Micrandrena) pusilla Pérez, 1903; order: Hymenoptera; family: Andrenidae; genus: Andrena; subgenus: Micrandrena; specificEpithet: pusilla; scientificNameAuthorship: Pérez, 1903; **Location:** country: Italy; countryCode: IT; stateProvince: Roma; locality: Riserva Naturale Valle dei Casali 2; decimalLatitude: 41.8596887; decimalLongitude: 12.4355075; geodeticDatum: WGS84; coordinatePrecision: 0.0002; **Identification:** identifiedBy: M. Mei; **Event:** eventDate: 2022-05-14; **Record Level:** collectionCode: UR3
- **Type status:** Other material. **Occurrence:** catalogNumber: A1208; recordedBy: L. Fortini; individualCount: 1; sex: female; lifeStage: adult; occurrenceID: 0A4FBB52-FEFA-5BA8-986B-E8F0A0B49F0C; **Taxon:** scientificName: Andrena (Micrandrena) pusilla Pérez, 1903; order: Hymenoptera; family: Andrenidae; genus: Andrena; subgenus: Micrandrena; specificEpithet: pusilla; scientificNameAuthorship: Pérez, 1903; **Location:** country: Italy; countryCode: IT; stateProvince: Roma; locality: Riserva Naturale di Monte Mario; decimalLatitude: 41.9386215; decimalLongitude: 12.4546223; geodeticDatum: WGS84; coordinatePrecision: 0.0002; **Identification:** identifiedBy: M. Mei; **Event:** eventDate: 2022-05-20; **Record Level:** collectionCode: UR3
- **Type status:** Other material. **Occurrence:** catalogNumber: A1216; recordedBy: L. Fortini; individualCount: 1; sex: female; lifeStage: adult; occurrenceID: D83920BB-083C-5EAB-A7A6-C1BF43DC7D14; **Taxon:** scientificName: Andrena (Micrandrena) pusilla Pérez, 1903; order: Hymenoptera; family: Andrenidae; genus: Andrena; subgenus: Micrandrena; specificEpithet: pusilla; scientificNameAuthorship: Pérez, 1903; **Location:** country: Italy; countryCode: IT; stateProvince: Roma; locality: Riserva Naturale dell'Insugherata 2; decimalLatitude: 41.9599247; decimalLongitude: 12.433852; geodeticDatum: WGS84; coordinatePrecision: 0.0002; **Identification:** identifiedBy: M. Mei; **Event:** eventDate: 2022-05-27; **Record Level:** collectionCode: UR3
- **Type status:** Other material. **Occurrence:** catalogNumber: A1238; recordedBy: L. Fortini; individualCount: 1; sex: female; lifeStage: adult; occurrenceID: 9C9CB5A8-4A5E-5123-8FB6-DEA04924CC7E; **Taxon:** scientificName: Andrena (Micrandrena) pusilla Pérez, 1903; order: Hymenoptera; family: Andrenidae; genus: Andrena; subgenus: Micrandrena; specificEpithet: pusilla; scientificNameAuthorship: Pérez, 1903; **Location:** country: Italy; countryCode: IT; stateProvince: Roma; locality: Riserva Regionale dell'Appia Antica 2; decimalLatitude: 41.8402564; decimalLongitude: 12.532773; geodeticDatum: WGS84; coordinatePrecision: 0.0002; **Identification:** identifiedBy: M. Mei; **Event:** eventDate: 2022-05-24; **Record Level:** collectionCode: UR3
- **Type status:** Other material. **Occurrence:** catalogNumber: A0982; recordedBy: L. Fortini; individualCount: 1; sex: female; lifeStage: adult; occurrenceID: D731BE4C-7F05-5B83-A1D6-2B991BDC57F8; **Taxon:** scientificName: Andrena (Micrandrena) pusilla Pérez, 1903; order: Hymenoptera; family: Andrenidae; genus: Andrena; subgenus: Micrandrena; specificEpithet: pusilla; scientificNameAuthorship: Pérez, 1903; **Location:** country: Italy; countryCode: IT; stateProvince: Roma; locality: Riserva Naturale Tenuta dei Massimi 2; decimalLatitude: 41.8316516; decimalLongitude: 12.3999927; geodeticDatum: WGS84; coordinatePrecision: 0.0002; **Identification:** identifiedBy: M. Mei; **Event:** eventDate: 2022-06-01; **Record Level:** collectionCode: UR3
- **Type status:** Other material. **Occurrence:** catalogNumber: A0927, A0928, A0929, A0930, A0981; recordedBy: L. Fortini; individualCount: 5; sex: females; lifeStage: adult; occurrenceID: 722F4F84-B087-5944-8105-259F14181B80; **Taxon:** scientificName: Andrena (Micrandrena) pusilla Pérez, 1903; order: Hymenoptera; family: Andrenidae; genus: Andrena; subgenus: Micrandrena; specificEpithet: pusilla; scientificNameAuthorship: Pérez, 1903; **Location:** country: Italy; countryCode: IT; stateProvince: Roma; locality: Riserva Naturale Valle dell'Aniene 2; decimalLatitude: 41.928752; decimalLongitude: 12.5562962; geodeticDatum: WGS84; coordinatePrecision: 0.0002; **Identification:** identifiedBy: M. Mei; **Event:** eventDate: 2022-06-05; **Record Level:** collectionCode: UR3

#### Andrena ranunculi

Schmiedeknecht, 1883

996BD50A-953C-595A-8B83-81429ED989AB

##### Materials

- **Type status:** Other material. **Occurrence:** catalogNumber: A0837, A0870; recordedBy: L. Fortini; individualCount: 2; sex: females; lifeStage: adult; occurrenceID: F758D7B3-24D1-5D70-A331-371166D1C98C; **Taxon:** scientificName: Andrena (Carandrena) ranunculi Schmiedeknecht, 1883; order: Hymenoptera; family: Andrenidae; genus: Andrena; subgenus: Carandrena; specificEpithet: ranunculi; scientificNameAuthorship: Schmiedeknecht, 1883; **Location:** country: Italy; countryCode: IT; stateProvince: Roma; locality: Riserva Naturale Valle dell'Aniene 1; decimalLatitude: 41.9345179; decimalLongitude: 12.5453096; geodeticDatum: WGS84; coordinatePrecision: 0.0002; **Identification:** identifiedBy: M. Mei; **Event:** eventDate: 2022-04-28; **Record Level:** collectionCode: UR3
- **Type status:** Other material. **Occurrence:** catalogNumber: A0838; recordedBy: L. Fortini; individualCount: 1; sex: female; lifeStage: adult; occurrenceID: EA035EC5-42A3-5F02-A9FA-C686E8449F4B; **Taxon:** scientificName: Andrena (Carandrena) ranunculi Schmiedeknecht, 1883; order: Hymenoptera; family: Andrenidae; genus: Andrena; subgenus: Carandrena; specificEpithet: ranunculi; scientificNameAuthorship: Schmiedeknecht, 1883; **Location:** country: Italy; countryCode: IT; stateProvince: Roma; locality: Riserva Naturale Valle dei Casali 1; decimalLatitude: 41.8710627; decimalLongitude: 12.4336809; geodeticDatum: WGS84; coordinatePrecision: 0.0002; **Identification:** identifiedBy: M. Mei; **Event:** eventDate: 2022-04-07; **Record Level:** collectionCode: UR3
- **Type status:** Other material. **Occurrence:** catalogNumber: A0839; recordedBy: L. Fortini; individualCount: 1; sex: female; lifeStage: adult; occurrenceID: FE466561-955F-5B7C-B2E6-D4DB3569789C; **Taxon:** scientificName: Andrena (Carandrena) ranunculi Schmiedeknecht, 1883; order: Hymenoptera; family: Andrenidae; genus: Andrena; subgenus: Carandrena; specificEpithet: ranunculi; scientificNameAuthorship: Schmiedeknecht, 1883; **Location:** country: Italy; countryCode: IT; stateProvince: Roma; locality: Riserva Naturale dell'Insugherata 3; decimalLatitude: 41.9644829; decimalLongitude: 12.436101; geodeticDatum: WGS84; coordinatePrecision: 0.0002; **Identification:** identifiedBy: M. Mei; **Event:** eventDate: 2022-04-15; **Record Level:** collectionCode: UR3

#### Andrena waschulzi

Strand, 1921

D478449D-4E28-5504-80D2-38625DA76F57

##### Materials

- **Type status:** Other material. **Occurrence:** catalogNumber: A1200, A1211; recordedBy: L. Fortini; individualCount: 2; sex: females; lifeStage: adult; occurrenceID: 6573049F-8CF8-519F-9411-6EE5C971F4BE; **Taxon:** scientificName: Andrena (Ulandrena) waschulzi Strand, 1921; order: Hymenoptera; family: Andrenidae; genus: Andrena; subgenus: Ulandrena; specificEpithet: waschulzi; scientificNameAuthorship: Strand, 1921; **Location:** country: Italy; countryCode: IT; stateProvince: Roma; locality: Riserva Regionale dell'Appia Antica 2; decimalLatitude: 41.8402564; decimalLongitude: 12.532773; geodeticDatum: WGS84; coordinatePrecision: 0.0002; **Identification:** identifiedBy: M. Mei; **Event:** eventDate: 2022-05-24; **Record Level:** collectionCode: UR3

#### Andrena senecionis

Pérez, 1895

43A73D84-77A8-52A5-BB86-4A193D0A494F

##### Materials

- **Type status:** Other material. **Occurrence:** catalogNumber: A0969, A0970, A0971, A0972; recordedBy: L. Fortini; individualCount: 4; sex: males; lifeStage: adult; occurrenceID: 8799F076-A412-513B-89C2-2894858F144D; **Taxon:** scientificName: Andrena (Chlorandrena) senecionis Pérez, 1895; order: Hymenoptera; family: Andrenidae; genus: Andrena; subgenus: Chlorandrena; specificEpithet: senecionis; scientificNameAuthorship: Pérez, 1895; **Location:** country: Italy; countryCode: IT; stateProvince: Roma; locality: Riserva Naturale di Monte Mario; decimalLatitude: 41.9386215; decimalLongitude: 12.4546223; geodeticDatum: WGS84; coordinatePrecision: 0.0002; **Identification:** identifiedBy: M. Mei; **Event:** eventDate: 2022-04-20; **Record Level:** collectionCode: UR3

#### Andrena schencki

Morawitz, 1866

D04AC559-85E4-5ED0-9B52-3643A41E2F06

##### Materials

- **Type status:** Other material. **Occurrence:** catalogNumber: A1000; recordedBy: L. Fortini; individualCount: 1; sex: female; lifeStage: adult; occurrenceID: 37D0929C-C7E7-51B4-BFCA-36F43ABB408B; **Taxon:** scientificName: Andrena (Opandrena) schencki Morawitz, 1866; order: Hymenoptera; family: Andrenidae; genus: Andrena; subgenus: Opandrena; specificEpithet: schencki; scientificNameAuthorship: Morawitz, 1866; **Location:** country: Italy; countryCode: IT; stateProvince: Roma; locality: Riserva Naturale dell'Insugherata 2; decimalLatitude: 41.9599247; decimalLongitude: 12.433852; geodeticDatum: WGS84; coordinatePrecision: 0.0002; **Identification:** identifiedBy: M. Mei; **Event:** eventDate: 2022-05-27; **Record Level:** collectionCode: UR3

#### Andrena schmiedeknechti

Magretti, 1883

7C6EAE05-3236-5735-A6D9-088A3C924ABD

##### Materials

- **Type status:** Other material. **Occurrence:** catalogNumber: A0936; recordedBy: L. Fortini; individualCount: 1; sex: male; lifeStage: adult; occurrenceID: C253E370-F94F-548F-A6B5-866ED756310E; **Taxon:** scientificName: Andrena (Truncandrena) schmiedeknechti Magretti,1883; order: Hymenoptera; family: Andrenidae; genus: Andrena; subgenus: Truncandrena; specificEpithet: schmiedeknechti; scientificNameAuthorship: Magretti,1883; **Location:** country: Italy; countryCode: IT; stateProvince: Roma; locality: Riserva Naturale dell'Insugherata 2; decimalLatitude: 41.9599247; decimalLongitude: 12.433852; geodeticDatum: WGS84; coordinatePrecision: 0.0002; **Identification:** identifiedBy: M. Mei; **Event:** eventDate: 2022-04-15; **Record Level:** collectionCode: UR3
- **Type status:** Other material. **Occurrence:** catalogNumber: A1978, A1989, A1995, A1996; recordedBy: L. Fortini; individualCount: 4; sex: females; lifeStage: adult; occurrenceID: 8BF32296-E3F3-5E05-BFB3-513B5669B72F; **Taxon:** scientificName: Andrena (Truncandrena) schmiedeknechti Magretti,1883; order: Hymenoptera; family: Andrenidae; genus: Andrena; subgenus: Truncandrena; specificEpithet: schmiedeknechti; scientificNameAuthorship: Magretti,1883; **Location:** country: Italy; countryCode: IT; stateProvince: Roma; locality: Riserva Regionale dell'Appia Antica 1; decimalLatitude: 41.8623941; decimalLongitude: 12.524863; geodeticDatum: WGS84; coordinatePrecision: 0.0002; **Identification:** identifiedBy: M. Mei; **Event:** eventDate: 2022-04-19; **Record Level:** collectionCode: UR3
- **Type status:** Other material. **Occurrence:** catalogNumber: A2020; recordedBy: L. Fortini; individualCount: 1; sex: female; lifeStage: adult; occurrenceID: 48203296-C3CD-5EA5-AB19-372583B76790; **Taxon:** scientificName: Andrena (Truncandrena) schmiedeknechti Magretti,1883; order: Hymenoptera; family: Andrenidae; genus: Andrena; subgenus: Truncandrena; specificEpithet: schmiedeknechti; scientificNameAuthorship: Magretti,1883; **Location:** country: Italy; countryCode: IT; stateProvince: Roma; locality: Riserva Regionale dell'Appia Antica 2; decimalLatitude: 41.8402564; decimalLongitude: 12.532773; geodeticDatum: WGS84; coordinatePrecision: 0.0002; **Identification:** identifiedBy: M. Mei; **Event:** eventDate: 2022-04-25; **Record Level:** collectionCode: UR3
- **Type status:** Other material. **Occurrence:** catalogNumber: A0823; recordedBy: L. Fortini; individualCount: 1; sex: female; lifeStage: adult; occurrenceID: 58301CF7-C784-5C24-AD66-323C34783C6E; **Taxon:** scientificName: Andrena (Truncandrena) schmiedeknechti Magretti,1883; order: Hymenoptera; family: Andrenidae; genus: Andrena; subgenus: Truncandrena; specificEpithet: schmiedeknechti; scientificNameAuthorship: Magretti,1883; **Location:** country: Italy; countryCode: IT; stateProvince: Roma; locality: Riserva Naturale Valle dell'Aniene 1; decimalLatitude: 41.9345179; decimalLongitude: 12.5453096; geodeticDatum: WGS84; coordinatePrecision: 0.0002; **Identification:** identifiedBy: M. Mei; **Event:** eventDate: 2022-04-28; **Record Level:** collectionCode: UR3
- **Type status:** Other material. **Occurrence:** catalogNumber: A0824, A0825, A0826, A0827, A0828, A0829, A0830, A0831; recordedBy: L. Fortini; individualCount: 8; sex: females; lifeStage: adult; occurrenceID: 1BDC691C-49BE-5B5D-B3B7-1DEA9F1EB152; **Taxon:** scientificName: Andrena (Truncandrena) schmiedeknechti Magretti,1883; order: Hymenoptera; family: Andrenidae; genus: Andrena; subgenus: Truncandrena; specificEpithet: schmiedeknechti; scientificNameAuthorship: Magretti,1883; **Location:** country: Italy; countryCode: IT; stateProvince: Roma; locality: Riserva Naturale Valle dei Casali 2; decimalLatitude: 41.8596887; decimalLongitude: 12.4355075; geodeticDatum: WGS84; coordinatePrecision: 0.0002; **Identification:** identifiedBy: M. Mei; **Event:** eventDate: 2022-05-14; **Record Level:** collectionCode: UR3

#### Andrena simontornyella

Noskiewicz, 1939

E8F9FA3D-E621-50B5-A7D1-9D316C045AF0

##### Materials

- **Type status:** Other material. **Occurrence:** catalogNumber: A1236, A1244; recordedBy: L. Fortini; individualCount: 2; sex: females; lifeStage: adult; occurrenceID: F06470F8-31E1-59C6-9F09-3CE6CB079032; **Taxon:** scientificName: Andrena (Micrandrena) simontornyella Noskiewicz, 1939; order: Hymenoptera; family: Andrenidae; genus: Andrena; subgenus: Micrandrena; specificEpithet: simontornyella; scientificNameAuthorship: Noskiewicz, 1939; **Location:** country: Italy; countryCode: IT; stateProvince: Roma; locality: Riserva Naturale Valle dell'Aniene 2; decimalLatitude: 41.928752; decimalLongitude: 12.5562962; geodeticDatum: WGS84; coordinatePrecision: 0.0002; **Identification:** identifiedBy: M. Mei; **Event:** eventDate: 2022-04-28; **Record Level:** collectionCode: UR3

#### Andrena stabiana

Morice, 1899

A8354DAB-FDCA-5C0D-92D8-6F6350E857E4

##### Materials

- **Type status:** Other material. **Occurrence:** catalogNumber: A0893, A0920; recordedBy: L. Fortini; individualCount: 2; sex: females; lifeStage: adult; occurrenceID: 8A8437A2-E2F4-5246-8849-02E2A119FB53; **Taxon:** scientificName: Andrena (Chlorandrena) stabiana Morice, 1899; order: Hymenoptera; family: Andrenidae; genus: Andrena; subgenus: Chlorandrena; specificEpithet: stabiana; scientificNameAuthorship: Morice, 1899; **Location:** country: Italy; countryCode: IT; stateProvince: Roma; locality: Riserva Naturale Valle dei Casali 1; decimalLatitude: 41.8710627; decimalLongitude: 12.4336809; geodeticDatum: WGS84; coordinatePrecision: 0.0002; **Identification:** identifiedBy: M. Mei; **Event:** eventDate: 2022-04-07; **Record Level:** collectionCode: UR3
- **Type status:** Other material. **Occurrence:** catalogNumber: A0882, A0909; recordedBy: L. Fortini; individualCount: 2; sex: females; lifeStage: adult; occurrenceID: E0D5FAE2-12E7-5773-96E9-CA2614E6D843; **Taxon:** scientificName: Andrena (Chlorandrena) stabiana Morice, 1899; order: Hymenoptera; family: Andrenidae; genus: Andrena; subgenus: Chlorandrena; specificEpithet: stabiana; scientificNameAuthorship: Morice, 1899; **Location:** country: Italy; countryCode: IT; stateProvince: Roma; locality: Riserva Naturale Laurentino-Acqua Acetosa; decimalLatitude: 41.8079275; decimalLongitude: 12.4685548; geodeticDatum: WGS84; coordinatePrecision: 0.0002; **Identification:** identifiedBy: M. Mei; **Event:** eventDate: 2022-04-12; **Record Level:** collectionCode: UR3
- **Type status:** Other material. **Occurrence:** catalogNumber: A0892, A0905, A0918, A0919; recordedBy: L. Fortini; individualCount: 4; sex: females; lifeStage: adult; occurrenceID: 89F90633-69AC-5C86-BCC9-D87EF04750AB; **Taxon:** scientificName: Andrena (Chlorandrena) stabiana Morice, 1899; order: Hymenoptera; family: Andrenidae; genus: Andrena; subgenus: Chlorandrena; specificEpithet: stabiana; scientificNameAuthorship: Morice, 1899; **Location:** country: Italy; countryCode: IT; stateProvince: Roma; locality: Riserva Naturale dell'Insugherata 1; decimalLatitude: 41.9555045; decimalLongitude: 12.4292321; geodeticDatum: WGS84; coordinatePrecision: 0.0002; **Identification:** identifiedBy: M. Mei; **Event:** eventDate: 2022-04-15; **Record Level:** collectionCode: UR3
- **Type status:** Other material. **Occurrence:** catalogNumber: A1986; recordedBy: L. Fortini; individualCount: 1; sex: female; lifeStage: adult; occurrenceID: F512D890-4B35-5D43-BD24-E618705FC157; **Taxon:** scientificName: Andrena (Chlorandrena) stabiana Morice, 1899; order: Hymenoptera; family: Andrenidae; genus: Andrena; subgenus: Chlorandrena; specificEpithet: stabiana; scientificNameAuthorship: Morice, 1899; **Location:** country: Italy; countryCode: IT; stateProvince: Roma; locality: Riserva Regionale dell'Appia Antica 1; decimalLatitude: 41.8623941; decimalLongitude: 12.524863; geodeticDatum: WGS84; coordinatePrecision: 0.0002; **Identification:** identifiedBy: M. Mei; **Event:** eventDate: 2022-04-19; **Record Level:** collectionCode: UR3
- **Type status:** Other material. **Occurrence:** catalogNumber: A0906, A0907, A0911, A0912, A0914, A0921; recordedBy: L. Fortini; individualCount: 6; sex: females; lifeStage: adult; occurrenceID: 09E09928-2551-5ECA-9B4A-8F9C3D54F457; **Taxon:** scientificName: Andrena (Chlorandrena) stabiana Morice, 1899; order: Hymenoptera; family: Andrenidae; genus: Andrena; subgenus: Chlorandrena; specificEpithet: stabiana; scientificNameAuthorship: Morice, 1899; **Location:** country: Italy; countryCode: IT; stateProvince: Roma; locality: Riserva Naturale di Monte Mario; decimalLatitude: 41.9386215; decimalLongitude: 12.4546223; geodeticDatum: WGS84; coordinatePrecision: 0.0002; **Identification:** identifiedBy: M. Mei; **Event:** eventDate: 2022-04-20; **Record Level:** collectionCode: UR3
- **Type status:** Other material. **Occurrence:** catalogNumber: A2017; recordedBy: L. Fortini; individualCount: 1; sex: female; lifeStage: adult; occurrenceID: C03173F8-E656-53D2-B487-2AB731ED6BD0; **Taxon:** scientificName: Andrena (Chlorandrena) stabiana Morice, 1899; order: Hymenoptera; family: Andrenidae; genus: Andrena; subgenus: Chlorandrena; specificEpithet: stabiana; scientificNameAuthorship: Morice, 1899; **Location:** country: Italy; countryCode: IT; stateProvince: Roma; locality: Riserva Regionale dell'Appia Antica 2; decimalLatitude: 41.8402564; decimalLongitude: 12.532773; geodeticDatum: WGS84; coordinatePrecision: 0.0002; **Identification:** identifiedBy: M. Mei; **Event:** eventDate: 2022-04-25; **Record Level:** collectionCode: UR3
- **Type status:** Other material. **Occurrence:** catalogNumber: A0923; recordedBy: L. Fortini; individualCount: 1; sex: female; lifeStage: adult; occurrenceID: 96920DFF-9375-51CD-9F0E-8C23A2E7A85C; **Taxon:** scientificName: Andrena (Chlorandrena) stabiana Morice, 1899; order: Hymenoptera; family: Andrenidae; genus: Andrena; subgenus: Chlorandrena; specificEpithet: stabiana; scientificNameAuthorship: Morice, 1899; **Location:** country: Italy; countryCode: IT; stateProvince: Roma; locality: Riserva Naturale Tenuta dei Massimi 2; decimalLatitude: 41.8316516; decimalLongitude: 12.3999927; geodeticDatum: WGS84; coordinatePrecision: 0.0002; **Identification:** identifiedBy: M. Mei; **Event:** eventDate: 2022-05-04; **Record Level:** collectionCode: UR3

#### Andrena thoracica

Fabricius, 1775

760D156E-4512-5B48-AD68-516577A6D464

##### Materials

- **Type status:** Other material. **Occurrence:** catalogNumber: A1064; recordedBy: L. Fortini; individualCount: 1; sex: male; lifeStage: adult; occurrenceID: F3D126CE-C3B3-57B6-ABA2-045594A0D9F0; **Taxon:** scientificName: Andrena (Melandrena) thoracica (Fabricius, 1775); order: Hymenoptera; family: Andrenidae; genus: Andrena; subgenus: Melandrena; specificEpithet: thoracica; scientificNameAuthorship: (Fabricius, 1775); **Location:** country: Italy; countryCode: IT; stateProvince: Roma; locality: Riserva Naturale dell'Insugherata 3; decimalLatitude: 41.9644829; decimalLongitude: 12.436101; geodeticDatum: WGS84; coordinatePrecision: 0.0002; **Identification:** identifiedBy: M. Mei; **Event:** eventDate: 2022-05-27; **Record Level:** collectionCode: UR3
- **Type status:** Other material. **Occurrence:** catalogNumber: A1065; recordedBy: L. Fortini; individualCount: 1; sex: female; lifeStage: adult; occurrenceID: F885D684-16EB-579F-BFF0-080BBAB211F5; **Taxon:** scientificName: Andrena (Melandrena) thoracica (Fabricius, 1775); order: Hymenoptera; family: Andrenidae; genus: Andrena; subgenus: Melandrena; specificEpithet: thoracica; scientificNameAuthorship: (Fabricius, 1775); **Location:** country: Italy; countryCode: IT; stateProvince: Roma; locality: Riserva Naturale Valle dell'Aniene 2; decimalLatitude: 41.928752; decimalLongitude: 12.5562962; geodeticDatum: WGS84; coordinatePrecision: 0.0002; **Identification:** identifiedBy: M. Mei; **Event:** eventDate: 2022-06-05; **Record Level:** collectionCode: UR3

#### Andrena truncatilabris

Morawitz, 1877

E145D8AD-576F-5A33-A47E-DD48CCA657FB

##### Materials

- **Type status:** Other material. **Occurrence:** catalogNumber: A0937, A0948; recordedBy: L. Fortini; individualCount: 2; sex: males; lifeStage: adult; occurrenceID: 628DDCD6-D9D3-5D58-AECB-8396E8FAB491; **Taxon:** scientificName: Andrena (Truncandrena) truncatilabris Morawitz,1877; order: Hymenoptera; family: Andrenidae; genus: Andrena; subgenus: Truncandrena; specificEpithet: truncatilabris; scientificNameAuthorship: Morawitz,1877; **Location:** country: Italy; countryCode: IT; stateProvince: Roma; locality: Riserva Naturale Valle dei Casali 1; decimalLatitude: 41.8710627; decimalLongitude: 12.4336809; geodeticDatum: WGS84; coordinatePrecision: 0.0002; **Identification:** identifiedBy: M. Mei; **Event:** eventDate: 2022-04-07; **Record Level:** collectionCode: UR3
- **Type status:** Other material. **Occurrence:** catalogNumber: A0943; recordedBy: L. Fortini; individualCount: 1; sex: male; lifeStage: adult; occurrenceID: 6CC48D8B-A2D8-5969-83E3-B49B9F7C5AF1; **Taxon:** scientificName: Andrena (Truncandrena) truncatilabris Morawitz,1877; order: Hymenoptera; family: Andrenidae; genus: Andrena; subgenus: Truncandrena; specificEpithet: truncatilabris; scientificNameAuthorship: Morawitz,1877; **Location:** country: Italy; countryCode: IT; stateProvince: Roma; locality: Riserva Naturale dell'Acquafredda; decimalLatitude: 41.8928408; decimalLongitude: 12.39932; geodeticDatum: WGS84; coordinatePrecision: 0.0002; **Identification:** identifiedBy: M. Mei; **Event:** eventDate: 2022-04-13; **Record Level:** collectionCode: UR3
- **Type status:** Other material. **Occurrence:** catalogNumber: A0941, A0944, A0945; recordedBy: L. Fortini; individualCount: 3; sex: males; lifeStage: adult; occurrenceID: C142A9BA-E71D-5A4E-AE5B-A056E44AAE34; **Taxon:** scientificName: Andrena (Truncandrena) truncatilabris Morawitz,1877; order: Hymenoptera; family: Andrenidae; genus: Andrena; subgenus: Truncandrena; specificEpithet: truncatilabris; scientificNameAuthorship: Morawitz,1877; **Location:** country: Italy; countryCode: IT; stateProvince: Roma; locality: Riserva Regionale dell'Appia Antica 3; decimalLatitude: 41.8298456; decimalLongitude: 12.5432538; geodeticDatum: WGS84; coordinatePrecision: 0.0002; **Identification:** identifiedBy: M. Mei; **Event:** eventDate: 2022-04-15; **Record Level:** collectionCode: UR3
- **Type status:** Other material. **Occurrence:** catalogNumber: A0946; recordedBy: L. Fortini; individualCount: 1; sex: male; lifeStage: adult; occurrenceID: 1B19BCD0-7116-5FED-BE2C-A522EA8AD69C; **Taxon:** scientificName: Andrena (Truncandrena) truncatilabris Morawitz,1877; order: Hymenoptera; family: Andrenidae; genus: Andrena; subgenus: Truncandrena; specificEpithet: truncatilabris; scientificNameAuthorship: Morawitz,1877; **Location:** country: Italy; countryCode: IT; stateProvince: Roma; locality: Riserva Naturale Tenuta dei Massimi 1; decimalLatitude: 41.8532859; decimalLongitude: 12.3842322; geodeticDatum: WGS84; coordinatePrecision: 0.0002; **Identification:** identifiedBy: M. Mei; **Event:** eventDate: 2022-04-23; **Record Level:** collectionCode: UR3
- **Type status:** Other material. **Occurrence:** catalogNumber: A0890, A0891, A0938, A0939, A0940, A0942, A0947, A0949; recordedBy: L. Fortini; individualCount: 8; sex: 6 males, 2 females; lifeStage: adult; occurrenceID: 56218422-3409-5264-B74C-54E30668BD08; **Taxon:** scientificName: Andrena (Truncandrena) truncatilabris Morawitz,1877; order: Hymenoptera; family: Andrenidae; genus: Andrena; subgenus: Truncandrena; specificEpithet: truncatilabris; scientificNameAuthorship: Morawitz,1877; **Location:** country: Italy; countryCode: IT; stateProvince: Roma; locality: Riserva Naturale Valle dell'Aniene 2; decimalLatitude: 41.928752; decimalLongitude: 12.5562962; geodeticDatum: WGS84; coordinatePrecision: 0.0002; **Identification:** identifiedBy: M. Mei; **Event:** eventDate: 2022-04-28; **Record Level:** collectionCode: UR3
- **Type status:** Other material. **Occurrence:** catalogNumber: A0884; recordedBy: L. Fortini; individualCount: 1; sex: female; lifeStage: adult; occurrenceID: BFD1FD60-436B-5AE3-9D18-FA4AC11D4A09; **Taxon:** scientificName: Andrena (Truncandrena) truncatilabris Morawitz,1877; order: Hymenoptera; family: Andrenidae; genus: Andrena; subgenus: Truncandrena; specificEpithet: truncatilabris; scientificNameAuthorship: Morawitz,1877; **Location:** country: Italy; countryCode: IT; stateProvince: Roma; locality: Riserva Regionale dell'Appia Antica 1; decimalLatitude: 41.8623941; decimalLongitude: 12.524863; geodeticDatum: WGS84; coordinatePrecision: 0.0002; **Identification:** identifiedBy: M. Mei; **Event:** eventDate: 2022-05-10; **Record Level:** collectionCode: UR3
- **Type status:** Other material. **Occurrence:** catalogNumber: A0822; recordedBy: L. Fortini; individualCount: 1; sex: female; lifeStage: adult; occurrenceID: 2DA1195A-8E73-5B65-9AEA-B5B3BE94A046; **Taxon:** scientificName: Andrena (Truncandrena) truncatilabris Morawitz,1877; order: Hymenoptera; family: Andrenidae; genus: Andrena; subgenus: Truncandrena; specificEpithet: truncatilabris; scientificNameAuthorship: Morawitz,1877; **Location:** country: Italy; countryCode: IT; stateProvince: Roma; locality: Riserva Naturale Valle dei Casali 1; decimalLatitude: 41.8710627; decimalLongitude: 12.4336809; geodeticDatum: WGS84; coordinatePrecision: 0.0002; **Identification:** identifiedBy: M. Mei; **Event:** eventDate: 2022-05-14; **Record Level:** collectionCode: UR3
- **Type status:** Other material. **Occurrence:** catalogNumber: A0821, A0889; recordedBy: L. Fortini; individualCount: 2; sex: females; lifeStage: adult; occurrenceID: A38E6842-0535-5403-8A8E-0ADC7CFE475B; **Taxon:** scientificName: Andrena (Truncandrena) truncatilabris Morawitz,1877; order: Hymenoptera; family: Andrenidae; genus: Andrena; subgenus: Truncandrena; specificEpithet: truncatilabris; scientificNameAuthorship: Morawitz,1877; **Location:** country: Italy; countryCode: IT; stateProvince: Roma; locality: Riserva Naturale Valle dei Casali 2; decimalLatitude: 41.8596887; decimalLongitude: 12.4355075; geodeticDatum: WGS84; coordinatePrecision: 0.0002; **Identification:** identifiedBy: M. Mei; **Event:** eventDate: 2022-05-14; **Record Level:** collectionCode: UR3
- **Type status:** Other material. **Occurrence:** catalogNumber: A0819, A0886, A0888, A0931; recordedBy: L. Fortini; individualCount: 4; sex: females; lifeStage: adult; occurrenceID: C67DCCE5-7032-5AED-9F75-63D5B7C1502E; **Taxon:** scientificName: Andrena (Truncandrena) truncatilabris Morawitz,1877; order: Hymenoptera; family: Andrenidae; genus: Andrena; subgenus: Truncandrena; specificEpithet: truncatilabris; scientificNameAuthorship: Morawitz,1877; **Location:** country: Italy; countryCode: IT; stateProvince: Roma; locality: Riserva Naturale dell'Acquafredda; decimalLatitude: 41.8928408; decimalLongitude: 12.39932; geodeticDatum: WGS84; coordinatePrecision: 0.0002; **Identification:** identifiedBy: M. Mei; **Event:** eventDate: 2022-05-15; **Record Level:** collectionCode: UR3
- **Type status:** Other material. **Occurrence:** catalogNumber: A0820, A0887; recordedBy: L. Fortini; individualCount: 2; sex: females; lifeStage: adult; occurrenceID: 42E3D922-4E20-5257-B45F-D865649EF3F7; **Taxon:** scientificName: Andrena (Truncandrena) truncatilabris Morawitz,1877; order: Hymenoptera; family: Andrenidae; genus: Andrena; subgenus: Truncandrena; specificEpithet: truncatilabris; scientificNameAuthorship: Morawitz,1877; **Location:** country: Italy; countryCode: IT; stateProvince: Roma; locality: Riserva Regionale dell'Appia Antica 2; decimalLatitude: 41.8402564; decimalLongitude: 12.532773; geodeticDatum: WGS84; coordinatePrecision: 0.0002; **Identification:** identifiedBy: M. Mei; **Event:** eventDate: 2022-05-24; **Record Level:** collectionCode: UR3
- **Type status:** Other material. **Occurrence:** catalogNumber: A0885; recordedBy: L. Fortini; individualCount: 1; sex: female; lifeStage: adult; occurrenceID: 46C8D424-8D5A-56B7-980B-DA0479A2677F; **Taxon:** scientificName: Andrena (Truncandrena) truncatilabris Morawitz,1877; order: Hymenoptera; family: Andrenidae; genus: Andrena; subgenus: Truncandrena; specificEpithet: truncatilabris; scientificNameAuthorship: Morawitz,1877; **Location:** country: Italy; countryCode: IT; stateProvince: Roma; locality: Riserva Naturale dell'Insugherata 2; decimalLatitude: 41.9599247; decimalLongitude: 12.433852; geodeticDatum: WGS84; coordinatePrecision: 0.0002; **Identification:** identifiedBy: M. Mei; **Event:** eventDate: 2022-05-27; **Record Level:** collectionCode: UR3
- **Type status:** Other material. **Occurrence:** catalogNumber: A0883; recordedBy: L. Fortini; individualCount: 1; sex: female; lifeStage: adult; occurrenceID: 32E7E82F-E970-576E-8193-A1BBFC9F3590; **Taxon:** scientificName: Andrena (Truncandrena) truncatilabris Morawitz,1877; order: Hymenoptera; family: Andrenidae; genus: Andrena; subgenus: Truncandrena; specificEpithet: truncatilabris; scientificNameAuthorship: Morawitz,1877; **Location:** country: Italy; countryCode: IT; stateProvince: Roma; locality: Riserva Naturale Laurentino-Acqua Acetosa; decimalLatitude: 41.8079275; decimalLongitude: 12.4685548; geodeticDatum: WGS84; coordinatePrecision: 0.0002; **Identification:** identifiedBy: M. Mei; **Event:** eventDate: 2022-06-16; **Record Level:** collectionCode: UR3

#### Andrena ventricosa

Dours, 1873

493235DC-621E-581D-BD5E-7E6A16BDEDDD

##### Materials

- **Type status:** Other material. **Occurrence:** catalogNumber: A0860; recordedBy: L. Fortini; individualCount: 1; sex: female; lifeStage: adult; occurrenceID: AFC527DA-ED0F-5025-83CB-13EF05B6B100; **Taxon:** scientificName: Andrena (Cryptandrena) ventricosa Dours, 1873; order: Hymenoptera; family: Andrenidae; genus: Andrena; subgenus: Cryptandrena; specificEpithet: ventricosa; scientificNameAuthorship: Dours, 1873; **Location:** country: Italy; countryCode: IT; stateProvince: Roma; locality: Riserva Naturale dell'Acquafredda; decimalLatitude: 41.8928408; decimalLongitude: 12.39932; geodeticDatum: WGS84; coordinatePrecision: 0.0002; **Identification:** identifiedBy: M. Mei; **Event:** eventDate: 2022-04-13; **Record Level:** collectionCode: UR3
- **Type status:** Other material. **Occurrence:** catalogNumber: A0842; recordedBy: L. Fortini; individualCount: 1; sex: female; lifeStage: adult; occurrenceID: DA3C8995-2EC0-527C-AA78-D5E5C8951C89; **Taxon:** scientificName: Andrena (Cryptandrena) ventricosa Dours, 1873; order: Hymenoptera; family: Andrenidae; genus: Andrena; subgenus: Cryptandrena; specificEpithet: ventricosa; scientificNameAuthorship: Dours, 1873; **Location:** country: Italy; countryCode: IT; stateProvince: Roma; locality: Riserva Naturale Tenuta dei Massimi 1; decimalLatitude: 41.8532859; decimalLongitude: 12.3842322; geodeticDatum: WGS84; coordinatePrecision: 0.0002; **Identification:** identifiedBy: M. Mei; **Event:** eventDate: 2022-04-23; **Record Level:** collectionCode: UR3
- **Type status:** Other material. **Occurrence:** catalogNumber: A0840, A0841, A0843, A0844, A0845; recordedBy: L. Fortini; individualCount: 5; sex: females; lifeStage: adult; occurrenceID: 0F84F908-3580-5098-B506-3A44929FB906; **Taxon:** scientificName: Andrena (Cryptandrena) ventricosa Dours, 1873; order: Hymenoptera; family: Andrenidae; genus: Andrena; subgenus: Cryptandrena; specificEpithet: ventricosa; scientificNameAuthorship: Dours, 1873; **Location:** country: Italy; countryCode: IT; stateProvince: Roma; locality: Riserva Naturale Valle dell'Aniene 1; decimalLatitude: 41.9345179; decimalLongitude: 12.5453096; geodeticDatum: WGS84; coordinatePrecision: 0.0002; **Identification:** identifiedBy: M. Mei; **Event:** eventDate: 2022-04-28; **Record Level:** collectionCode: UR3

#### Andrena vetula

Lepeletier, 1841

D775C0FA-438A-5FD6-B3F3-D6E3C01317D1

##### Materials

- **Type status:** Other material. **Occurrence:** catalogNumber: A1055; recordedBy: L. Fortini; individualCount: 1; sex: male; lifeStage: adult; occurrenceID: BB7FF6B5-9DCF-533C-B4A2-C871429D41D4; **Taxon:** scientificName: Andrena (Ptilandrena) vetula Lepeletier, 1841; order: Hymenoptera; family: Andrenidae; genus: Andrena; subgenus: Ptilandrena; specificEpithet: vetula; scientificNameAuthorship: Lepeletier, 1841; **Location:** country: Italy; countryCode: IT; stateProvince: Roma; locality: Riserva Naturale dell'Insugherata 3; decimalLatitude: 41.9644829; decimalLongitude: 12.436101; geodeticDatum: WGS84; coordinatePrecision: 0.0002; **Identification:** identifiedBy: M. Mei; **Event:** eventDate: 2022-04-07; **Record Level:** collectionCode: UR3
- **Type status:** Other material. **Occurrence:** catalogNumber: A1046, A1047; recordedBy: L. Fortini; individualCount: 2; sex: 1 male, 1 female; lifeStage: adult; occurrenceID: 41C973B2-5835-56AA-8A00-5EC9FCC415A9; **Taxon:** scientificName: Andrena (Ptilandrena) vetula Lepeletier, 1841; order: Hymenoptera; family: Andrenidae; genus: Andrena; subgenus: Ptilandrena; specificEpithet: vetula; scientificNameAuthorship: Lepeletier, 1841; **Location:** country: Italy; countryCode: IT; stateProvince: Roma; locality: Riserva Naturale Valle dell'Aniene 2; decimalLatitude: 41.928752; decimalLongitude: 12.5562962; geodeticDatum: WGS84; coordinatePrecision: 0.0002; **Identification:** identifiedBy: M. Mei; **Event:** eventDate: 2022-06-05; **Record Level:** collectionCode: UR3
- **Type status:** Other material. **Occurrence:** catalogNumber: A1056, A1057; recordedBy: L. Fortini; individualCount: 2; sex: males; lifeStage: adult; occurrenceID: 3F626FD5-AA2B-55BC-9C01-9F4071376FF0; **Taxon:** scientificName: Andrena (Ptilandrena) vetula Lepeletier, 1841; order: Hymenoptera; family: Andrenidae; genus: Andrena; subgenus: Ptilandrena; specificEpithet: vetula; scientificNameAuthorship: Lepeletier, 1841; **Location:** country: Italy; countryCode: IT; stateProvince: Roma; locality: Riserva Naturale Valle dell'Aniene 2; decimalLatitude: 41.928752; decimalLongitude: 12.5562962; geodeticDatum: WGS84; coordinatePrecision: 0.0002; **Identification:** identifiedBy: M. Mei; **Event:** eventDate: 2022-04-28; **Record Level:** collectionCode: UR3
- **Type status:** Other material. **Occurrence:** catalogNumber: A1048, A1049, A1050, A1051, A1052, A1053, A1054; recordedBy: L. Fortini; individualCount: 7; sex: males; lifeStage: adult; occurrenceID: CCB4875C-0E39-5B52-8425-E406A4B779D7; **Taxon:** scientificName: Andrena (Ptilandrena) vetula Lepeletier, 1841; order: Hymenoptera; family: Andrenidae; genus: Andrena; subgenus: Ptilandrena; specificEpithet: vetula; scientificNameAuthorship: Lepeletier, 1841; **Location:** country: Italy; countryCode: IT; stateProvince: Roma; locality: Riserva Naturale Valle dei Casali 1; decimalLatitude: 41.8710627; decimalLongitude: 12.4336809; geodeticDatum: WGS84; coordinatePrecision: 0.0002; **Identification:** identifiedBy: M. Mei; **Event:** eventDate: 2022-04-07; **Record Level:** collectionCode: UR3

#### Panurgus canescens

Latreille, 1811

2B4F389A-AB93-51D2-A1F5-DFF22F6305B5

##### Materials

- **Type status:** Other material. **Occurrence:** catalogNumber: A0463; recordedBy: L. Fortini; individualCount: 1; sex: male; lifeStage: adult; occurrenceID: D2E76CCB-DB9B-5725-A94F-AF345355E35D; **Taxon:** scientificName: Panurgus (Pachycepalopanurgus) canescens Latreille, 1811; order: Hymenoptera; family: Andrenidae; genus: Panurgus; subgenus: Pachycepalopanurgus; specificEpithet: canescens; scientificNameAuthorship: Latreille, 1811; **Location:** country: Italy; countryCode: IT; stateProvince: Roma; locality: Riserva Naturale dell'Acquafredda; decimalLatitude: 41.8928408; decimalLongitude: 12.39932; geodeticDatum: WGS84; coordinatePrecision: 0.0002; **Identification:** identifiedBy: L. Fortini; **Event:** eventDate: 2022-07-12; **Record Level:** collectionCode: UR3
- **Type status:** Other material. **Occurrence:** catalogNumber: A0464; recordedBy: L. Fortini; individualCount: 1; sex: male; lifeStage: adult; occurrenceID: 97D6B861-C0A9-5A9C-AB65-60A1F67BAC7B; **Taxon:** scientificName: Panurgus (Pachycepalopanurgus) canescens Latreille, 1811; order: Hymenoptera; family: Andrenidae; genus: Panurgus; subgenus: Pachycepalopanurgus; specificEpithet: canescens; scientificNameAuthorship: Latreille, 1811; **Location:** country: Italy; countryCode: IT; stateProvince: Roma; locality: Riserva Naturale dell'Insugherata 1; decimalLatitude: 41.9555045; decimalLongitude: 12.4292321; geodeticDatum: WGS84; coordinatePrecision: 0.0002; **Identification:** identifiedBy: L. Fortini; **Event:** eventDate: 2022-06-24; **Record Level:** collectionCode: UR3

### Apidae Latreille, 1802

#### Amegilla albigena

(Lepeletier, 1841)

DAF543C5-F0FC-5E6E-982B-8C8A9B9A6EAE

##### Materials

- **Type status:** Other material. **Occurrence:** catalogNumber: A0341; recordedBy: L. Fortini; individualCount: 1; sex: male; lifeStage: adult; occurrenceID: 58565C65-27E9-5086-942E-9DC04ED461AD; **Taxon:** scientificName: Amegilla (Zebramegilla) albigena (Lepeletier, 1841); order: Hymenoptera; family: Apidae; genus: Amegilla; subgenus: Zebramegilla; specificEpithet: albigena; scientificNameAuthorship: (Lepeletier, 1841); **Location:** country: Italy; countryCode: IT; stateProvince: Roma; locality: Riserva Naturale Valle dell'Aniene 2; decimalLatitude: 41.928752; decimalLongitude: 12.5562962; geodeticDatum: WGS84; coordinatePrecision: 0.0002; **Identification:** identifiedBy: L. Fortini; **Event:** eventDate: 2022-07-01; **Record Level:** collectionCode: UR3
- **Type status:** Other material. **Occurrence:** catalogNumber: A0342; recordedBy: L. Fortini; individualCount: 1; sex: male; lifeStage: adult; occurrenceID: 7688A794-5F48-5968-8B40-C55E2AAC776B; **Taxon:** scientificName: Amegilla (Zebramegilla) albigena (Lepeletier, 1841); order: Hymenoptera; family: Apidae; genus: Amegilla; subgenus: Zebramegilla; specificEpithet: albigena; scientificNameAuthorship: (Lepeletier, 1841); **Location:** country: Italy; countryCode: IT; stateProvince: Roma; locality: Riserva Naturale Tenuta dei Massimi 2; decimalLatitude: 41.8316516; decimalLongitude: 12.3999927; geodeticDatum: WGS84; coordinatePrecision: 0.0002; **Identification:** identifiedBy: L. Fortini; **Event:** eventDate: 2022-07-28; **Record Level:** collectionCode: UR3
- **Type status:** Other material. **Occurrence:** catalogNumber: A0343; recordedBy: L. Fortini; individualCount: 1; sex: female; lifeStage: adult; occurrenceID: 7AB2A445-E140-5752-8C7E-CCB1CC164F5B; **Taxon:** scientificName: Amegilla (Zebramegilla) albigena (Lepeletier, 1841); order: Hymenoptera; family: Apidae; genus: Amegilla; subgenus: Zebramegilla; specificEpithet: albigena; scientificNameAuthorship: (Lepeletier, 1841); **Location:** country: Italy; countryCode: IT; stateProvince: Roma; locality: Riserva Regionale dell'Appia Antica 1; decimalLatitude: 41.8623941; decimalLongitude: 12.524863; geodeticDatum: WGS84; coordinatePrecision: 0.0002; **Identification:** identifiedBy: L. Fortini; **Event:** eventDate: 2022-06-12; **Record Level:** collectionCode: UR3

#### Amegilla garrula

(Rossi, 1790)

E7CAF1DA-94C6-59A1-99E1-82D69AD38985

##### Materials

- **Type status:** Other material. **Occurrence:** catalogNumber: A0344; recordedBy: L. Fortini; individualCount: 1; sex: male; lifeStage: adult; occurrenceID: 76A4E4B7-249C-5B9D-9540-A2A3173941D1; **Taxon:** scientificName: Amegilla (Amegilla) garrula (Rossi, 1790); order: Hymenoptera; family: Apidae; genus: Amegilla; subgenus: Amegilla; specificEpithet: garrula; scientificNameAuthorship: (Rossi, 1790); **Location:** country: Italy; countryCode: IT; stateProvince: Roma; locality: Riserva Naturale di Monte Mario; decimalLatitude: 41.9386215; decimalLongitude: 12.4546223; geodeticDatum: WGS84; coordinatePrecision: 0.0002; **Identification:** identifiedBy: L. Fortini; **Event:** eventDate: 2022-07-24; **Record Level:** collectionCode: UR3
- **Type status:** Other material. **Occurrence:** catalogNumber: A0345; recordedBy: L. Fortini; individualCount: 1; sex: male; lifeStage: adult; occurrenceID: C6FBA5B9-A415-53D8-98F7-4385A3546510; **Taxon:** scientificName: Amegilla (Amegilla) garrula (Rossi, 1790); order: Hymenoptera; family: Apidae; genus: Amegilla; subgenus: Amegilla; specificEpithet: garrula; scientificNameAuthorship: (Rossi, 1790); **Location:** country: Italy; countryCode: IT; stateProvince: Roma; locality: Riserva Naturale dell'Insugherata 3; decimalLatitude: 41.9644829; decimalLongitude: 12.436101; geodeticDatum: WGS84; coordinatePrecision: 0.0002; **Identification:** identifiedBy: L. Fortini; **Event:** eventDate: 2022-07-30; **Record Level:** collectionCode: UR3
- **Type status:** Other material. **Occurrence:** catalogNumber: A0346; recordedBy: L. Fortini; individualCount: 1; sex: female; lifeStage: adult; occurrenceID: 491F1BDC-8BC3-5AD8-8B5B-047B50556863; **Taxon:** scientificName: Amegilla (Amegilla) garrula (Rossi, 1790); order: Hymenoptera; family: Apidae; genus: Amegilla; subgenus: Amegilla; specificEpithet: garrula; scientificNameAuthorship: (Rossi, 1790); **Location:** country: Italy; countryCode: IT; stateProvince: Roma; locality: Riserva Naturale di Monte Mario; decimalLatitude: 41.9386215; decimalLongitude: 12.4546223; geodeticDatum: WGS84; coordinatePrecision: 0.0002; **Identification:** identifiedBy: L. Fortini; **Event:** eventDate: 2022-06-19; **Record Level:** collectionCode: UR3

#### Amegilla quadrifasciata

(de Villers, 1789)

B204920E-8966-5C86-8BD4-77D8B89840D1

##### Materials

- **Type status:** Other material. **Occurrence:** catalogNumber: A0337, A0338; recordedBy: L. Fortini; individualCount: 2; sex: females; lifeStage: adult; occurrenceID: 90EC0F15-F45A-55A5-B9F7-C97AA0785F58; **Taxon:** scientificName: Amegilla (Amegilla) quadrifasciata (de Villers, 1789); order: Hymenoptera; family: Apidae; genus: Amegilla; subgenus: Amegilla; specificEpithet: quadrifasciata; scientificNameAuthorship: (de Villers, 1789); **Location:** country: Italy; countryCode: IT; stateProvince: Roma; locality: Riserva Regionale dell'Appia Antica 1; decimalLatitude: 41.8623941; decimalLongitude: 12.524863; geodeticDatum: WGS84; coordinatePrecision: 0.0002; **Identification:** identifiedBy: L. Fortini; **Event:** eventDate: 2022-06-12; **Record Level:** collectionCode: UR3
- **Type status:** Other material. **Occurrence:** catalogNumber: A0372; recordedBy: L. Fortini; individualCount: 1; sex: male; lifeStage: adult; occurrenceID: 9D83FF1C-648A-58AF-B26A-60FFD7527AEC; **Taxon:** scientificName: Amegilla (Amegilla) quadrifasciata (de Villers, 1789); order: Hymenoptera; family: Apidae; genus: Amegilla; subgenus: Amegilla; specificEpithet: quadrifasciata; scientificNameAuthorship: (de Villers, 1789); **Location:** country: Italy; countryCode: IT; stateProvince: Roma; locality: Riserva Regionale dell'Appia Antica 1; decimalLatitude: 41.8623941; decimalLongitude: 12.524863; geodeticDatum: WGS84; coordinatePrecision: 0.0002; **Identification:** identifiedBy: L. Fortini; **Event:** eventDate: 2022-09-25; **Record Level:** collectionCode: UR3
- **Type status:** Other material. **Occurrence:** catalogNumber: A0334; recordedBy: L. Fortini; individualCount: 1; sex: male; lifeStage: adult; occurrenceID: 778D553E-3029-5D19-A4EA-C594F97F0505; **Taxon:** scientificName: Amegilla (Amegilla) quadrifasciata (de Villers, 1789); order: Hymenoptera; family: Apidae; genus: Amegilla; subgenus: Amegilla; specificEpithet: quadrifasciata; scientificNameAuthorship: (de Villers, 1789); **Location:** country: Italy; countryCode: IT; stateProvince: Roma; locality: Riserva Naturale di Monte Mario; decimalLatitude: 41.9386215; decimalLongitude: 12.4546223; geodeticDatum: WGS84; coordinatePrecision: 0.0002; **Identification:** identifiedBy: L. Fortini; **Event:** eventDate: 2022-07-24; **Record Level:** collectionCode: UR3
- **Type status:** Other material. **Occurrence:** catalogNumber: A0331; recordedBy: L. Fortini; individualCount: 1; sex: male; lifeStage: adult; occurrenceID: FA3C4705-628B-5A89-9C5B-7FF711A9ED00; **Taxon:** scientificName: Amegilla (Amegilla) quadrifasciata (de Villers, 1789); order: Hymenoptera; family: Apidae; genus: Amegilla; subgenus: Amegilla; specificEpithet: quadrifasciata; scientificNameAuthorship: (de Villers, 1789); **Location:** country: Italy; countryCode: IT; stateProvince: Roma; locality: Riserva Naturale Tenuta dei Massimi 2; decimalLatitude: 41.8316516; decimalLongitude: 12.3999927; geodeticDatum: WGS84; coordinatePrecision: 0.0002; **Identification:** identifiedBy: L. Fortini; **Event:** eventDate: 2022-07-28; **Record Level:** collectionCode: UR3
- **Type status:** Other material. **Occurrence:** catalogNumber: A0336; recordedBy: L. Fortini; individualCount: 1; sex: female; lifeStage: adult; occurrenceID: 285AA9A7-74F6-5A75-8AA5-CE98121D31C7; **Taxon:** scientificName: Amegilla (Amegilla) quadrifasciata (de Villers, 1789); order: Hymenoptera; family: Apidae; genus: Amegilla; subgenus: Amegilla; specificEpithet: quadrifasciata; scientificNameAuthorship: (de Villers, 1789); **Location:** country: Italy; countryCode: IT; stateProvince: Roma; locality: Riserva Naturale Valle dell'Aniene 1; decimalLatitude: 41.9345179; decimalLongitude: 12.5453096; geodeticDatum: WGS84; coordinatePrecision: 0.0002; **Identification:** identifiedBy: L. Fortini; **Event:** eventDate: 2022-09-04; **Record Level:** collectionCode: UR3
- **Type status:** Other material. **Occurrence:** catalogNumber: A0339, A0340; recordedBy: L. Fortini; individualCount: 2; sex: females; lifeStage: adult; occurrenceID: A929D63A-BF36-56EF-84EF-F00CEAEDFC46; **Taxon:** scientificName: Amegilla (Amegilla) quadrifasciata (de Villers, 1789); order: Hymenoptera; family: Apidae; genus: Amegilla; subgenus: Amegilla; specificEpithet: quadrifasciata; scientificNameAuthorship: (de Villers, 1789); **Location:** country: Italy; countryCode: IT; stateProvince: Roma; locality: Riserva Naturale Valle dell'Aniene 2; decimalLatitude: 41.928752; decimalLongitude: 12.5562962; geodeticDatum: WGS84; coordinatePrecision: 0.0002; **Identification:** identifiedBy: L. Fortini; **Event:** eventDate: 2022-07-01; **Record Level:** collectionCode: UR3
- **Type status:** Other material. **Occurrence:** catalogNumber: A0332, A0333, A0335, A0371; recordedBy: L. Fortini; individualCount: 4; sex: males; lifeStage: adult; occurrenceID: EBC48430-A68A-5C13-87F8-E5C8F4DB1A96; **Taxon:** scientificName: Amegilla (Amegilla) quadrifasciata (de Villers, 1789); order: Hymenoptera; family: Apidae; genus: Amegilla; subgenus: Amegilla; specificEpithet: quadrifasciata; scientificNameAuthorship: (de Villers, 1789); **Location:** country: Italy; countryCode: IT; stateProvince: Roma; locality: Riserva Naturale Valle dei Casali 1; decimalLatitude: 41.8710627; decimalLongitude: 12.4336809; geodeticDatum: WGS84; coordinatePrecision: 0.0002; **Identification:** identifiedBy: L. Fortini; **Event:** eventDate: 2022-09-18; **Record Level:** collectionCode: UR3

#### Anthophora atroalba

Lepeletier, 1841

9AEC3CCF-0B77-5049-BCBC-CCD7A53EBA23

##### Materials

- **Type status:** Other material. **Occurrence:** catalogNumber: A1917; recordedBy: L. Fortini; individualCount: 1; sex: male; lifeStage: adult; occurrenceID: 1C0A49DE-ED44-5AA6-9BEE-67DC2D4A96D8; **Taxon:** scientificName: Anthophora (Pyganthophora) atroalba Lepeletier, 1841; order: Hymenoptera; family: Apidae; genus: Anthophora; subgenus: Pyganthophora; specificEpithet: atroalba; scientificNameAuthorship: Lepeletier, 1841; **Location:** country: Italy; countryCode: IT; stateProvince: Roma; locality: Riserva Naturale Laurentino-Acqua Acetosa; decimalLatitude: 41.8079275; decimalLongitude: 12.4685548; geodeticDatum: WGS84; coordinatePrecision: 0.0002; **Identification:** identifiedBy: D. Lucente; **Event:** eventDate: 2022-04-12; **Record Level:** collectionCode: UR3
- **Type status:** Other material. **Occurrence:** catalogNumber: A1918; recordedBy: L. Fortini; individualCount: 1; sex: male; lifeStage: adult; occurrenceID: AA2BF25F-FE3B-5FC5-8235-A29C63F1DC2C; **Taxon:** scientificName: Anthophora (Pyganthophora) atroalba Lepeletier, 1841; order: Hymenoptera; family: Apidae; genus: Anthophora; subgenus: Pyganthophora; specificEpithet: atroalba; scientificNameAuthorship: Lepeletier, 1841; **Location:** country: Italy; countryCode: IT; stateProvince: Roma; locality: Riserva Naturale Valle dei Casali 1; decimalLatitude: 41.8710627; decimalLongitude: 12.4336809; geodeticDatum: WGS84; coordinatePrecision: 0.0002; **Identification:** identifiedBy: D. Lucente; **Event:** eventDate: 2022-04-13; **Record Level:** collectionCode: UR3
- **Type status:** Other material. **Occurrence:** catalogNumber: A1919; recordedBy: L. Fortini; individualCount: 1; sex: male; lifeStage: adult; occurrenceID: 70B4EA63-D54C-5599-99CE-C66AD4306840; **Taxon:** scientificName: Anthophora (Pyganthophora) atroalba Lepeletier, 1841; order: Hymenoptera; family: Apidae; genus: Anthophora; subgenus: Pyganthophora; specificEpithet: atroalba; scientificNameAuthorship: Lepeletier, 1841; **Location:** country: Italy; countryCode: IT; stateProvince: Roma; locality: Riserva Naturale Valle dei Casali 2; decimalLatitude: 41.8596887; decimalLongitude: 12.4355075; geodeticDatum: WGS84; coordinatePrecision: 0.0002; **Identification:** identifiedBy: D. Lucente; **Event:** eventDate: 2022-04-10; **Record Level:** collectionCode: UR3
- **Type status:** Other material. **Occurrence:** catalogNumber: A1920; recordedBy: L. Fortini; individualCount: 1; sex: male; lifeStage: adult; occurrenceID: 8C4F796C-F218-5BFA-91E3-216B35D500E1; **Taxon:** scientificName: Anthophora (Pyganthophora) atroalba Lepeletier, 1841; order: Hymenoptera; family: Apidae; genus: Anthophora; subgenus: Pyganthophora; specificEpithet: atroalba; scientificNameAuthorship: Lepeletier, 1841; **Location:** country: Italy; countryCode: IT; stateProvince: Roma; locality: Riserva Naturale Valle dei Casali 1; decimalLatitude: 41.8710627; decimalLongitude: 12.4336809; geodeticDatum: WGS84; coordinatePrecision: 0.0002; **Identification:** identifiedBy: D. Lucente; **Event:** eventDate: 2022-04-07; **Record Level:** collectionCode: UR3
- **Type status:** Other material. **Occurrence:** catalogNumber: A1921, A1953; recordedBy: L. Fortini; individualCount: 2; sex: 1 male, 1 female; lifeStage: adult; occurrenceID: DDDD5EF4-57B9-5D3F-83E2-610CE908F288; **Taxon:** scientificName: Anthophora (Pyganthophora) atroalba Lepeletier, 1841; order: Hymenoptera; family: Apidae; genus: Anthophora; subgenus: Pyganthophora; specificEpithet: atroalba; scientificNameAuthorship: Lepeletier, 1841; **Location:** country: Italy; countryCode: IT; stateProvince: Roma; locality: Riserva Naturale Tenuta dei Massimi 1; decimalLatitude: 41.8532859; decimalLongitude: 12.3842322; geodeticDatum: WGS84; coordinatePrecision: 0.0002; **Identification:** identifiedBy: D. Lucente; **Event:** eventDate: 2022-04-23; **Record Level:** collectionCode: UR3
- **Type status:** Other material. **Occurrence:** catalogNumber: A1934; recordedBy: L. Fortini; individualCount: 1; sex: male; lifeStage: adult; occurrenceID: 9902A255-DD94-5678-9B64-73BB78F02D5A; **Taxon:** scientificName: Anthophora (Pyganthophora) atroalba Lepeletier, 1841; order: Hymenoptera; family: Apidae; genus: Anthophora; subgenus: Pyganthophora; specificEpithet: atroalba; scientificNameAuthorship: Lepeletier, 1841; **Location:** country: Italy; countryCode: IT; stateProvince: Roma; locality: Riserva Regionale dell'Appia Antica 1; decimalLatitude: 41.8623941; decimalLongitude: 12.524863; geodeticDatum: WGS84; coordinatePrecision: 0.0002; **Identification:** identifiedBy: D. Lucente; **Event:** eventDate: 2022-05-10; **Record Level:** collectionCode: UR3
- **Type status:** Other material. **Occurrence:** catalogNumber: A1958; recordedBy: L. Fortini; individualCount: 1; sex: female; lifeStage: adult; occurrenceID: D041482F-DE57-57E6-BD9C-71E1A2F5CF7B; **Taxon:** scientificName: Anthophora (Pyganthophora) atroalba Lepeletier, 1841; order: Hymenoptera; family: Apidae; genus: Anthophora; subgenus: Pyganthophora; specificEpithet: atroalba; scientificNameAuthorship: Lepeletier, 1841; **Location:** country: Italy; countryCode: IT; stateProvince: Roma; locality: Riserva Regionale dell'Appia Antica 1; decimalLatitude: 41.8623941; decimalLongitude: 12.524863; geodeticDatum: WGS84; coordinatePrecision: 0.0002; **Identification:** identifiedBy: D. Lucente; **Event:** eventDate: 2022-06-12; **Record Level:** collectionCode: UR3
- **Type status:** Other material. **Occurrence:** catalogNumber: A1957, A1961, A1963; recordedBy: L. Fortini; individualCount: 3; sex: 1 male, 2 females; lifeStage: adult; occurrenceID: 08295F37-99CB-58E2-A29F-B518F554A584; **Taxon:** scientificName: Anthophora (Pyganthophora) atroalba Lepeletier, 1841; order: Hymenoptera; family: Apidae; genus: Anthophora; subgenus: Pyganthophora; specificEpithet: atroalba; scientificNameAuthorship: Lepeletier, 1841; **Location:** country: Italy; countryCode: IT; stateProvince: Roma; locality: Riserva Naturale dell'Insugherata 1; decimalLatitude: 41.9555045; decimalLongitude: 12.4292321; geodeticDatum: WGS84; coordinatePrecision: 0.0002; **Identification:** identifiedBy: D. Lucente; **Event:** eventDate: 2022-05-27; **Record Level:** collectionCode: UR3
- **Type status:** Other material. **Occurrence:** catalogNumber: A1956, A1959, A1960, A1962; recordedBy: L. Fortini; individualCount: 4; sex: females; lifeStage: adult; occurrenceID: 224FC193-9ABB-5236-8491-FE66D8E50FC3; **Taxon:** scientificName: Anthophora (Pyganthophora) atroalba Lepeletier, 1841; order: Hymenoptera; family: Apidae; genus: Anthophora; subgenus: Pyganthophora; specificEpithet: atroalba; scientificNameAuthorship: Lepeletier, 1841; **Location:** country: Italy; countryCode: IT; stateProvince: Roma; locality: Riserva Naturale dell'Insugherata 2; decimalLatitude: 41.9599247; decimalLongitude: 12.433852; geodeticDatum: WGS84; coordinatePrecision: 0.0002; **Identification:** identifiedBy: D. Lucente; **Event:** eventDate: 2022-05-27; **Record Level:** collectionCode: UR3
- **Type status:** Other material. **Occurrence:** catalogNumber: A1991; recordedBy: L. Fortini; individualCount: 1; sex: male; lifeStage: adult; occurrenceID: 19333162-85F2-514C-8B32-EF572C475454; **Taxon:** scientificName: Anthophora (Pyganthophora) atroalba Lepeletier, 1841; order: Hymenoptera; family: Apidae; genus: Anthophora; subgenus: Pyganthophora; specificEpithet: atroalba; scientificNameAuthorship: Lepeletier, 1841; **Location:** country: Italy; countryCode: IT; stateProvince: Roma; locality: Riserva Regionale dell'Appia Antica 1; decimalLatitude: 41.8623941; decimalLongitude: 12.524863; geodeticDatum: WGS84; coordinatePrecision: 0.0002; **Identification:** identifiedBy: D. Lucente; **Event:** eventDate: 2022-04-19; **Record Level:** collectionCode: UR3

#### Anthophora crinipes

Smith, 1854

7B28C5A1-C994-5D2E-9AFE-11F78EFB340B

##### Materials

- **Type status:** Other material. **Occurrence:** catalogNumber: A1932, A1952; recordedBy: L. Fortini; individualCount: 2; sex: 1 male, 1 female; lifeStage: adult; occurrenceID: 8745A82A-F9F9-5EDE-A2FA-42E0DBE1FFE9; **Taxon:** scientificName: Anthophora (Anthophora) crinipes Smith, 1854; order: Hymenoptera; family: Apidae; genus: Anthophora; subgenus: Anthophora; specificEpithet: crinipes; scientificNameAuthorship: Smith, 1854; **Location:** country: Italy; countryCode: IT; stateProvince: Roma; locality: Riserva Naturale di Monte Mario; decimalLatitude: 41.9386215; decimalLongitude: 12.4546223; geodeticDatum: WGS84; coordinatePrecision: 0.0002; **Identification:** identifiedBy: D. Lucente; **Event:** eventDate: 2022-04-20; **Record Level:** collectionCode: UR3
- **Type status:** Other material. **Occurrence:** catalogNumber: A1933; recordedBy: L. Fortini; individualCount: 1; sex: male; lifeStage: adult; occurrenceID: 5E379F0D-2690-5F45-BEAC-D4CD94F9DAD7; **Taxon:** scientificName: Anthophora (Anthophora) crinipes Smith, 1854; order: Hymenoptera; family: Apidae; genus: Anthophora; subgenus: Anthophora; specificEpithet: crinipes; scientificNameAuthorship: Smith, 1854; **Location:** country: Italy; countryCode: IT; stateProvince: Roma; locality: Riserva Naturale Tenuta dei Massimi 1; decimalLatitude: 41.8532859; decimalLongitude: 12.3842322; geodeticDatum: WGS84; coordinatePrecision: 0.0002; **Identification:** identifiedBy: D. Lucente; **Event:** eventDate: 2022-04-23; **Record Level:** collectionCode: UR3
- **Type status:** Other material. **Occurrence:** catalogNumber: A1935, A1936, A1939, A1943, A1944, A1948; recordedBy: L. Fortini; individualCount: 6; sex: males; lifeStage: adult; occurrenceID: 274E2D70-E1A7-5704-B05F-8C1831EBD863; **Taxon:** scientificName: Anthophora (Anthophora) crinipes Smith, 1854; order: Hymenoptera; family: Apidae; genus: Anthophora; subgenus: Anthophora; specificEpithet: crinipes; scientificNameAuthorship: Smith, 1854; **Location:** country: Italy; countryCode: IT; stateProvince: Roma; locality: Riserva Regionale dell'Appia Antica 1; decimalLatitude: 41.8623941; decimalLongitude: 12.524863; geodeticDatum: WGS84; coordinatePrecision: 0.0002; **Identification:** identifiedBy: D. Lucente; **Event:** eventDate: 2022-05-10; **Record Level:** collectionCode: UR3
- **Type status:** Other material. **Occurrence:** catalogNumber: A1955; recordedBy: L. Fortini; individualCount: 1; sex: female; lifeStage: adult; occurrenceID: 59DB206A-391D-5708-9626-EB6F4836300E; **Taxon:** scientificName: Anthophora (Anthophora) crinipes Smith, 1854; order: Hymenoptera; family: Apidae; genus: Anthophora; subgenus: Anthophora; specificEpithet: crinipes; scientificNameAuthorship: Smith, 1854; **Location:** country: Italy; countryCode: IT; stateProvince: Roma; locality: Riserva Regionale dell'Appia Antica 1; decimalLatitude: 41.8623941; decimalLongitude: 12.524863; geodeticDatum: WGS84; coordinatePrecision: 0.0002; **Identification:** identifiedBy: D. Lucente; **Event:** eventDate: 2022-06-12; **Record Level:** collectionCode: UR3
- **Type status:** Other material. **Occurrence:** catalogNumber: A1938, A1941, A1942; recordedBy: L. Fortini; individualCount: 3; sex: males; lifeStage: adult; occurrenceID: 57BF9009-BE38-536F-BD50-D6C2C7627A13; **Taxon:** scientificName: Anthophora (Anthophora) crinipes Smith, 1854; order: Hymenoptera; family: Apidae; genus: Anthophora; subgenus: Anthophora; specificEpithet: crinipes; scientificNameAuthorship: Smith, 1854; **Location:** country: Italy; countryCode: IT; stateProvince: Roma; locality: Riserva Naturale Valle dei Casali 1; decimalLatitude: 41.8710627; decimalLongitude: 12.4336809; geodeticDatum: WGS84; coordinatePrecision: 0.0002; **Identification:** identifiedBy: D. Lucente; **Event:** eventDate: 2022-04-07; **Record Level:** collectionCode: UR3
- **Type status:** Other material. **Occurrence:** catalogNumber: A1954; recordedBy: L. Fortini; individualCount: 1; sex: female; lifeStage: adult; occurrenceID: 46163F5E-79A4-5334-B2AB-7D465C999C44; **Taxon:** scientificName: Anthophora (Anthophora) crinipes Smith, 1854; order: Hymenoptera; family: Apidae; genus: Anthophora; subgenus: Anthophora; specificEpithet: crinipes; scientificNameAuthorship: Smith, 1854; **Location:** country: Italy; countryCode: IT; stateProvince: Roma; locality: Riserva Naturale dell'Acquafredda; decimalLatitude: 41.8928408; decimalLongitude: 12.39932; geodeticDatum: WGS84; coordinatePrecision: 0.0002; **Identification:** identifiedBy: D. Lucente; **Event:** eventDate: 2022-04-13; **Record Level:** collectionCode: UR3

#### Anthophora dispar

Lepeletier, 1841

E03A9421-587F-51BE-BFD8-5CC04269EDD2

##### Materials

- **Type status:** Other material. **Occurrence:** catalogNumber: A1945; recordedBy: L. Fortini; individualCount: 1; sex: male; lifeStage: adult; occurrenceID: 450BD638-22C7-5259-817F-56E515AAED31; **Taxon:** scientificName: Anthophora (Lophanthophora) dispar Lepeletier, 1841; order: Hymenoptera; family: Apidae; genus: Anthophora; subgenus: Lophanthophora; specificEpithet: dispar; scientificNameAuthorship: Lepeletier, 1841; **Location:** country: Italy; countryCode: IT; stateProvince: Roma; locality: Riserva Naturale Valle dell'Aniene 2; decimalLatitude: 41.928752; decimalLongitude: 12.5562962; geodeticDatum: WGS84; coordinatePrecision: 0.0002; **Identification:** identifiedBy: D. Lucente; **Event:** eventDate: 2022-04-28; **Record Level:** collectionCode: UR3

#### Anthophora plumipes

(Pallas, 1772)

28C062BA-1A52-5A3F-9B0D-E2FAFEA7AD19

##### Materials

- **Type status:** Other material. **Occurrence:** catalogNumber: A1922, A1930; recordedBy: L. Fortini; individualCount: 2; sex: males; lifeStage: adult; occurrenceID: D66F4887-528F-5159-AC6B-658482A8C4C4; **Taxon:** scientificName: Anthophora (Anthophora) plumipes (Pallas,1772); order: Hymenoptera; family: Apidae; genus: Anthophora; subgenus: Anthophora; specificEpithet: plumipes; scientificNameAuthorship: (Pallas,1772); **Location:** country: Italy; countryCode: IT; stateProvince: Roma; locality: Riserva Naturale Laurentino-Acqua Acetosa; decimalLatitude: 41.8079275; decimalLongitude: 12.4685548; geodeticDatum: WGS84; coordinatePrecision: 0.0002; **Identification:** identifiedBy: D. Lucente; **Event:** eventDate: 2022-04-12; **Record Level:** collectionCode: UR3
- **Type status:** Other material. **Occurrence:** catalogNumber: A1923, A1924, A1928; recordedBy: L. Fortini; individualCount: 3; sex: males; lifeStage: adult; occurrenceID: 7C8EE0E5-78D8-5E45-94A5-9ED887E6A240; **Taxon:** scientificName: Anthophora (Anthophora) plumipes (Pallas,1772); order: Hymenoptera; family: Apidae; genus: Anthophora; subgenus: Anthophora; specificEpithet: plumipes; scientificNameAuthorship: (Pallas,1772); **Location:** country: Italy; countryCode: IT; stateProvince: Roma; locality: Riserva Naturale Valle dei Casali 2; decimalLatitude: 41.8596887; decimalLongitude: 12.4355075; geodeticDatum: WGS84; coordinatePrecision: 0.0002; **Identification:** identifiedBy: D. Lucente; **Event:** eventDate: 2022-04-10; **Record Level:** collectionCode: UR3
- **Type status:** Other material. **Occurrence:** catalogNumber: A1925, A1927; recordedBy: L. Fortini; individualCount: 2; sex: males; lifeStage: adult; occurrenceID: 1705E960-0935-5B52-85ED-D1F3D4BCF8B4; **Taxon:** scientificName: Anthophora (Anthophora) plumipes (Pallas,1772); order: Hymenoptera; family: Apidae; genus: Anthophora; subgenus: Anthophora; specificEpithet: plumipes; scientificNameAuthorship: (Pallas,1772); **Location:** country: Italy; countryCode: IT; stateProvince: Roma; locality: Riserva Naturale Valle dei Casali 1; decimalLatitude: 41.8710627; decimalLongitude: 12.4336809; geodeticDatum: WGS84; coordinatePrecision: 0.0002; **Identification:** identifiedBy: D. Lucente; **Event:** eventDate: 2022-04-07; **Record Level:** collectionCode: UR3
- **Type status:** Other material. **Occurrence:** catalogNumber: A1926, A1929, A1931, A1937; recordedBy: L. Fortini; individualCount: 4; sex: 3 males, 1 female; lifeStage: adult; occurrenceID: 6A68331A-B47C-596C-8477-257751002BD3; **Taxon:** scientificName: Anthophora (Anthophora) plumipes (Pallas,1772); order: Hymenoptera; family: Apidae; genus: Anthophora; subgenus: Anthophora; specificEpithet: plumipes; scientificNameAuthorship: (Pallas,1772); **Location:** country: Italy; countryCode: IT; stateProvince: Roma; locality: Riserva Naturale dell'Insugherata 1; decimalLatitude: 41.9555045; decimalLongitude: 12.4292321; geodeticDatum: WGS84; coordinatePrecision: 0.0002; **Identification:** identifiedBy: D. Lucente; **Event:** eventDate: 2022-04-15; **Record Level:** collectionCode: UR3
- **Type status:** Other material. **Occurrence:** catalogNumber: A1940, A1951; recordedBy: L. Fortini; individualCount: 2; sex: males; lifeStage: adult; occurrenceID: E0904F02-CA40-5165-A745-B6034300EDF3; **Taxon:** scientificName: Anthophora (Anthophora) plumipes (Pallas,1772); order: Hymenoptera; family: Apidae; genus: Anthophora; subgenus: Anthophora; specificEpithet: plumipes; scientificNameAuthorship: (Pallas,1772); **Location:** country: Italy; countryCode: IT; stateProvince: Roma; locality: Riserva Regionale dell'Appia Antica 1; decimalLatitude: 41.8623941; decimalLongitude: 12.524863; geodeticDatum: WGS84; coordinatePrecision: 0.0002; **Identification:** identifiedBy: D. Lucente; **Event:** eventDate: 2022-05-10; **Record Level:** collectionCode: UR3
- **Type status:** Other material. **Occurrence:** catalogNumber: A1946; recordedBy: L. Fortini; individualCount: 1; sex: male; lifeStage: adult; occurrenceID: C09966AF-822A-53A6-BED7-B03438F6978A; **Taxon:** scientificName: Anthophora (Anthophora) plumipes (Pallas,1772); order: Hymenoptera; family: Apidae; genus: Anthophora; subgenus: Anthophora; specificEpithet: plumipes; scientificNameAuthorship: (Pallas,1772); **Location:** country: Italy; countryCode: IT; stateProvince: Roma; locality: Riserva Naturale Tenuta dei Massimi 2; decimalLatitude: 41.8316516; decimalLongitude: 12.3999927; geodeticDatum: WGS84; coordinatePrecision: 0.0002; **Identification:** identifiedBy: D. Lucente; **Event:** eventDate: 2022-05-04; **Record Level:** collectionCode: UR3
- **Type status:** Other material. **Occurrence:** catalogNumber: A1947, A1949, A1950; recordedBy: L. Fortini; individualCount: 3; sex: males; lifeStage: adult; occurrenceID: 91E071AE-871A-5C32-AA44-5D3688488039; **Taxon:** scientificName: Anthophora (Anthophora) plumipes (Pallas,1772); order: Hymenoptera; family: Apidae; genus: Anthophora; subgenus: Anthophora; specificEpithet: plumipes; scientificNameAuthorship: (Pallas,1772); **Location:** country: Italy; countryCode: IT; stateProvince: Roma; locality: Riserva Naturale Valle dell'Aniene 2; decimalLatitude: 41.928752; decimalLongitude: 12.5562962; geodeticDatum: WGS84; coordinatePrecision: 0.0002; **Identification:** identifiedBy: D. Lucente; **Event:** eventDate: 2022-04-28; **Record Level:** collectionCode: UR3
- **Type status:** Other material. **Occurrence:** catalogNumber: A1968, A1972, A1973, A1982; recordedBy: L. Fortini; individualCount: 4; sex: 2 males, 2 females; lifeStage: adult; occurrenceID: F61376FE-2AD9-56B0-99A6-E3082C62CB79; **Taxon:** scientificName: Anthophora (Anthophora) plumipes (Pallas,1772); order: Hymenoptera; family: Apidae; genus: Anthophora; subgenus: Anthophora; specificEpithet: plumipes; scientificNameAuthorship: (Pallas,1772); **Location:** country: Italy; countryCode: IT; stateProvince: Roma; locality: Riserva Regionale dell'Appia Antica 1; decimalLatitude: 41.8623941; decimalLongitude: 12.524863; geodeticDatum: WGS84; coordinatePrecision: 0.0002; **Identification:** identifiedBy: D. Lucente; **Event:** eventDate: 2022-04-19; **Record Level:** collectionCode: UR3
- **Type status:** Other material. **Occurrence:** catalogNumber: A2028; recordedBy: L. Fortini; individualCount: 1; sex: male; lifeStage: adult; occurrenceID: 2F7B8964-32E7-535D-A9B2-DBE9E9CC4145; **Taxon:** scientificName: Anthophora (Anthophora) plumipes (Pallas,1772); order: Hymenoptera; family: Apidae; genus: Anthophora; subgenus: Anthophora; specificEpithet: plumipes; scientificNameAuthorship: (Pallas,1772); **Location:** country: Italy; countryCode: IT; stateProvince: Roma; locality: Riserva Regionale dell'Appia Antica 2; decimalLatitude: 41.8402564; decimalLongitude: 12.532773; geodeticDatum: WGS84; coordinatePrecision: 0.0002; **Identification:** identifiedBy: D. Lucente; **Event:** eventDate: 2022-04-25; **Record Level:** collectionCode: UR3

#### Bombus pascuorum

(Scopoli, 1763)

2E16B104-35CB-527C-ABE0-F1E66DBCD290

##### Materials

- **Type status:** Other material. **Occurrence:** catalogNumber: A0272, A0274, A0276; recordedBy: L. Fortini; individualCount: 3; sex: females; lifeStage: adult; occurrenceID: 3D7A9EC8-C28F-5B11-B9BA-664B1D5A5DDB; **Taxon:** scientificName: Bombus (Thoracobombus) pascuorum (Scopoli,1763); order: Hymenoptera; family: Apidae; genus: Bombus; subgenus: Thoracobombus; specificEpithet: pascuorum; scientificNameAuthorship: (Scopoli,1763); **Location:** country: Italy; countryCode: IT; stateProvince: Roma; locality: Riserva Naturale dell'Acquafredda; decimalLatitude: 41.8928408; decimalLongitude: 12.39932; geodeticDatum: WGS84; coordinatePrecision: 0.0002; **Identification:** identifiedBy: L. Fortini; **Event:** eventDate: 2022-04-13; **Record Level:** collectionCode: UR3
- **Type status:** Other material. **Occurrence:** catalogNumber: A0302; recordedBy: L. Fortini; individualCount: 1; sex: female; lifeStage: adult; occurrenceID: 5AF8C68C-82C0-555C-9F09-7B072ADBF782; **Taxon:** scientificName: Bombus (Thoracobombus) pascuorum (Scopoli,1763); order: Hymenoptera; family: Apidae; genus: Bombus; subgenus: Thoracobombus; specificEpithet: pascuorum; scientificNameAuthorship: (Scopoli,1763); **Location:** country: Italy; countryCode: IT; stateProvince: Roma; locality: Riserva Naturale dell'Acquafredda; decimalLatitude: 41.8928408; decimalLongitude: 12.39932; geodeticDatum: WGS84; coordinatePrecision: 0.0002; **Identification:** identifiedBy: L. Fortini; **Event:** eventDate: 2022-06-10; **Record Level:** collectionCode: UR3
- **Type status:** Other material. **Occurrence:** catalogNumber: A0266; recordedBy: L. Fortini; individualCount: 1; sex: male; lifeStage: adult; occurrenceID: 073CAFB2-BF3C-5A6F-9B77-05AD0CE9A4CC; **Taxon:** scientificName: Bombus (Thoracobombus) pascuorum (Scopoli,1763); order: Hymenoptera; family: Apidae; genus: Bombus; subgenus: Thoracobombus; specificEpithet: pascuorum; scientificNameAuthorship: (Scopoli,1763); **Location:** country: Italy; countryCode: IT; stateProvince: Roma; locality: Riserva Regionale dell'Appia Antica 1; decimalLatitude: 41.8623941; decimalLongitude: 12.524863; geodeticDatum: WGS84; coordinatePrecision: 0.0002; **Identification:** identifiedBy: L. Fortini; **Event:** eventDate: 2022-09-25; **Record Level:** collectionCode: UR3
- **Type status:** Other material. **Occurrence:** catalogNumber: A0291, A0296, A0317; recordedBy: L. Fortini; individualCount: 3; sex: females; lifeStage: adult; occurrenceID: C5DBF12E-8C4E-5013-9092-CA9E4E4D9978; **Taxon:** scientificName: Bombus (Thoracobombus) pascuorum (Scopoli,1763); order: Hymenoptera; family: Apidae; genus: Bombus; subgenus: Thoracobombus; specificEpithet: pascuorum; scientificNameAuthorship: (Scopoli,1763); **Location:** country: Italy; countryCode: IT; stateProvince: Roma; locality: Riserva Regionale dell'Appia Antica 1; decimalLatitude: 41.8623941; decimalLongitude: 12.524863; geodeticDatum: WGS84; coordinatePrecision: 0.0002; **Identification:** identifiedBy: L. Fortini; **Event:** eventDate: 2022-06-12; **Record Level:** collectionCode: UR3
- **Type status:** Other material. **Occurrence:** catalogNumber: A0263, A0264, A0265, A0267, A0268, A0269, A079; recordedBy: L. Fortini; individualCount: 7; sex: 6 males, 1 female; lifeStage: adult; occurrenceID: 8DC80C77-01C7-50D5-841F-E1F2980BD5E5; **Taxon:** scientificName: Bombus (Thoracobombus) pascuorum (Scopoli,1763); order: Hymenoptera; family: Apidae; genus: Bombus; subgenus: Thoracobombus; specificEpithet: pascuorum; scientificNameAuthorship: (Scopoli,1763); **Location:** country: Italy; countryCode: IT; stateProvince: Roma; locality: Riserva Naturale dell'Insugherata 1; decimalLatitude: 41.9555045; decimalLongitude: 12.4292321; geodeticDatum: WGS84; coordinatePrecision: 0.0002; **Identification:** identifiedBy: L. Fortini; **Event:** eventDate: 2022-10-04; **Record Level:** collectionCode: UR3
- **Type status:** Other material. **Occurrence:** catalogNumber: A0284; recordedBy: L. Fortini; individualCount: 1; sex: female; lifeStage: adult; occurrenceID: 52E4A691-8A6E-52F3-8F0C-471FBE8D75DF; **Taxon:** scientificName: Bombus (Thoracobombus) pascuorum (Scopoli,1763); order: Hymenoptera; family: Apidae; genus: Bombus; subgenus: Thoracobombus; specificEpithet: pascuorum; scientificNameAuthorship: (Scopoli,1763); **Location:** country: Italy; countryCode: IT; stateProvince: Roma; locality: Riserva Naturale dell'Insugherata 1; decimalLatitude: 41.9555045; decimalLongitude: 12.4292321; geodeticDatum: WGS84; coordinatePrecision: 0.0002; **Identification:** identifiedBy: L. Fortini; **Event:** eventDate: 2022-04-15; **Record Level:** collectionCode: UR3
- **Type status:** Other material. **Occurrence:** catalogNumber: A0288; recordedBy: L. Fortini; individualCount: 1; sex: female; lifeStage: adult; occurrenceID: 0522BB3B-07EB-54C7-9166-7169749AAE1E; **Taxon:** scientificName: Bombus (Thoracobombus) pascuorum (Scopoli,1763); order: Hymenoptera; family: Apidae; genus: Bombus; subgenus: Thoracobombus; specificEpithet: pascuorum; scientificNameAuthorship: (Scopoli,1763); **Location:** country: Italy; countryCode: IT; stateProvince: Roma; locality: Riserva Naturale dell'Insugherata 1; decimalLatitude: 41.9555045; decimalLongitude: 12.4292321; geodeticDatum: WGS84; coordinatePrecision: 0.0002; **Identification:** identifiedBy: L. Fortini; **Event:** eventDate: 2022-05-27; **Record Level:** collectionCode: UR3
- **Type status:** Other material. **Occurrence:** catalogNumber: A0293, A0305, A0318; recordedBy: L. Fortini; individualCount: 3; sex: females; lifeStage: adult; occurrenceID: 4A598629-8B55-5A3B-95CD-F94215EF8973; **Taxon:** scientificName: Bombus (Thoracobombus) pascuorum (Scopoli,1763); order: Hymenoptera; family: Apidae; genus: Bombus; subgenus: Thoracobombus; specificEpithet: pascuorum; scientificNameAuthorship: (Scopoli,1763); **Location:** country: Italy; countryCode: IT; stateProvince: Roma; locality: Riserva Naturale dell'Insugherata 1; decimalLatitude: 41.9555045; decimalLongitude: 12.4292321; geodeticDatum: WGS84; coordinatePrecision: 0.0002; **Identification:** identifiedBy: L. Fortini; **Event:** eventDate: 2022-06-24; **Record Level:** collectionCode: UR3
- **Type status:** Other material. **Occurrence:** catalogNumber: A0275; recordedBy: L. Fortini; individualCount: 1; sex: female; lifeStage: adult; occurrenceID: 970119E1-D9DB-59F8-9559-51211039E797; **Taxon:** scientificName: Bombus (Thoracobombus) pascuorum (Scopoli,1763); order: Hymenoptera; family: Apidae; genus: Bombus; subgenus: Thoracobombus; specificEpithet: pascuorum; scientificNameAuthorship: (Scopoli,1763); **Location:** country: Italy; countryCode: IT; stateProvince: Roma; locality: Riserva Naturale dell'Insugherata 2; decimalLatitude: 41.9599247; decimalLongitude: 12.433852; geodeticDatum: WGS84; coordinatePrecision: 0.0002; **Identification:** identifiedBy: L. Fortini; **Event:** eventDate: 2022-04-15; **Record Level:** collectionCode: UR3
- **Type status:** Other material. **Occurrence:** catalogNumber: A0287, A0289, A0298, A0310; recordedBy: L. Fortini; individualCount: 4; sex: females; lifeStage: adult; occurrenceID: 993524D7-6B3B-5AEF-8395-B5BC5A9309B8; **Taxon:** scientificName: Bombus (Thoracobombus) pascuorum (Scopoli,1763); order: Hymenoptera; family: Apidae; genus: Bombus; subgenus: Thoracobombus; specificEpithet: pascuorum; scientificNameAuthorship: (Scopoli,1763); **Location:** country: Italy; countryCode: IT; stateProvince: Roma; locality: Riserva Naturale dell'Insugherata 2; decimalLatitude: 41.9599247; decimalLongitude: 12.433852; geodeticDatum: WGS84; coordinatePrecision: 0.0002; **Identification:** identifiedBy: L. Fortini; **Event:** eventDate: 2022-05-27; **Record Level:** collectionCode: UR3
- **Type status:** Other material. **Occurrence:** catalogNumber: A0294, A0295; recordedBy: L. Fortini; individualCount: 2; sex: females; lifeStage: adult; occurrenceID: E8A21144-76DB-5918-9E18-02CADBADA652; **Taxon:** scientificName: Bombus (Thoracobombus) pascuorum (Scopoli,1763); order: Hymenoptera; family: Apidae; genus: Bombus; subgenus: Thoracobombus; specificEpithet: pascuorum; scientificNameAuthorship: (Scopoli,1763); **Location:** country: Italy; countryCode: IT; stateProvince: Roma; locality: Riserva Naturale dell'Insugherata 2; decimalLatitude: 41.9599247; decimalLongitude: 12.433852; geodeticDatum: WGS84; coordinatePrecision: 0.0002; **Identification:** identifiedBy: L. Fortini; **Event:** eventDate: 2022-06-24; **Record Level:** collectionCode: UR3
- **Type status:** Other material. **Occurrence:** catalogNumber: A0283, A0292, A0312, A0322; recordedBy: L. Fortini; individualCount: 4; sex: females; lifeStage: adult; occurrenceID: B7056FD3-99D6-5F99-8A35-10E260F109A4; **Taxon:** scientificName: Bombus (Thoracobombus) pascuorum (Scopoli,1763); order: Hymenoptera; family: Apidae; genus: Bombus; subgenus: Thoracobombus; specificEpithet: pascuorum; scientificNameAuthorship: (Scopoli,1763); **Location:** country: Italy; countryCode: IT; stateProvince: Roma; locality: Riserva Naturale dell'Insugherata 2; decimalLatitude: 41.9599247; decimalLongitude: 12.433852; geodeticDatum: WGS84; coordinatePrecision: 0.0002; **Identification:** identifiedBy: L. Fortini; **Event:** eventDate: 2022-07-30; **Record Level:** collectionCode: UR3
- **Type status:** Other material. **Occurrence:** catalogNumber: A0301, A0303, A0304, A0306; recordedBy: L. Fortini; individualCount: 4; sex: females; lifeStage: adult; occurrenceID: 1E52830A-05F0-5896-B862-A30CCE6F7B22; **Taxon:** scientificName: Bombus (Thoracobombus) pascuorum (Scopoli,1763); order: Hymenoptera; family: Apidae; genus: Bombus; subgenus: Thoracobombus; specificEpithet: pascuorum; scientificNameAuthorship: (Scopoli,1763); **Location:** country: Italy; countryCode: IT; stateProvince: Roma; locality: Riserva Naturale dell'Insugherata 3; decimalLatitude: 41.9644829; decimalLongitude: 12.436101; geodeticDatum: WGS84; coordinatePrecision: 0.0002; **Identification:** identifiedBy: L. Fortini; **Event:** eventDate: 2022-06-24; **Record Level:** collectionCode: UR3
- **Type status:** Other material. **Occurrence:** catalogNumber: A0308; recordedBy: L. Fortini; individualCount: 1; sex: female; lifeStage: adult; occurrenceID: 16E9EA99-A3A1-5BC8-8E9B-8AA0AB0AD118; **Taxon:** scientificName: Bombus (Thoracobombus) pascuorum (Scopoli,1763); order: Hymenoptera; family: Apidae; genus: Bombus; subgenus: Thoracobombus; specificEpithet: pascuorum; scientificNameAuthorship: (Scopoli,1763); **Location:** country: Italy; countryCode: IT; stateProvince: Roma; locality: Riserva Naturale dell'Insugherata 3; decimalLatitude: 41.9644829; decimalLongitude: 12.436101; geodeticDatum: WGS84; coordinatePrecision: 0.0002; **Identification:** identifiedBy: L. Fortini; **Event:** eventDate: 2022-07-30; **Record Level:** collectionCode: UR3
- **Type status:** Other material. **Occurrence:** catalogNumber: A0290; recordedBy: L. Fortini; individualCount: 1; sex: female; lifeStage: adult; occurrenceID: 874B9F7A-4F57-59B3-A651-554A98E5BB6B; **Taxon:** scientificName: Bombus (Thoracobombus) pascuorum (Scopoli,1763); order: Hymenoptera; family: Apidae; genus: Bombus; subgenus: Thoracobombus; specificEpithet: pascuorum; scientificNameAuthorship: (Scopoli,1763); **Location:** country: Italy; countryCode: IT; stateProvince: Roma; locality: Riserva Naturale dell'Insugherata 3; decimalLatitude: 41.9644829; decimalLongitude: 12.436101; geodeticDatum: WGS84; coordinatePrecision: 0.0002; **Identification:** identifiedBy: L. Fortini; **Event:** eventDate: 2022-09-01; **Record Level:** collectionCode: UR3
- **Type status:** Other material. **Occurrence:** catalogNumber: A0273; recordedBy: L. Fortini; individualCount: 1; sex: female; lifeStage: adult; occurrenceID: 301913D3-245D-5195-A61D-2D48161A4BA5; **Taxon:** scientificName: Bombus (Thoracobombus) pascuorum (Scopoli,1763); order: Hymenoptera; family: Apidae; genus: Bombus; subgenus: Thoracobombus; specificEpithet: pascuorum; scientificNameAuthorship: (Scopoli,1763); **Location:** country: Italy; countryCode: IT; stateProvince: Roma; locality: Riserva Naturale Laurentino-Acqua Acetosa; decimalLatitude: 41.8079275; decimalLongitude: 12.4685548; geodeticDatum: WGS84; coordinatePrecision: 0.0002; **Identification:** identifiedBy: L. Fortini; **Event:** eventDate: 2022-04-12; **Record Level:** collectionCode: UR3
- **Type status:** Other material. **Occurrence:** catalogNumber: A0278, A0282, A0285, A0311; recordedBy: L. Fortini; individualCount: 4; sex: females; lifeStage: adult; occurrenceID: 04DAC108-5643-5808-8C02-498F53A94F0A; **Taxon:** scientificName: Bombus (Thoracobombus) pascuorum (Scopoli,1763); order: Hymenoptera; family: Apidae; genus: Bombus; subgenus: Thoracobombus; specificEpithet: pascuorum; scientificNameAuthorship: (Scopoli,1763); **Location:** country: Italy; countryCode: IT; stateProvince: Roma; locality: Riserva Naturale Laurentino-Acqua Acetosa; decimalLatitude: 41.8079275; decimalLongitude: 12.4685548; geodeticDatum: WGS84; coordinatePrecision: 0.0002; **Identification:** identifiedBy: L. Fortini; **Event:** eventDate: 2022-08-21; **Record Level:** collectionCode: UR3
- **Type status:** Other material. **Occurrence:** catalogNumber: A0280; recordedBy: L. Fortini; individualCount: 1; sex: female; lifeStage: adult; occurrenceID: 788FF58D-8637-54F3-AFDE-31D10B8E823D; **Taxon:** scientificName: Bombus (Thoracobombus) pascuorum (Scopoli,1763); order: Hymenoptera; family: Apidae; genus: Bombus; subgenus: Thoracobombus; specificEpithet: pascuorum; scientificNameAuthorship: (Scopoli,1763); **Location:** country: Italy; countryCode: IT; stateProvince: Roma; locality: Riserva Naturale Laurentino-Acqua Acetosa; decimalLatitude: 41.8079275; decimalLongitude: 12.4685548; geodeticDatum: WGS84; coordinatePrecision: 0.0002; **Identification:** identifiedBy: L. Fortini; **Event:** eventDate: 2022-09-14; **Record Level:** collectionCode: UR3
- **Type status:** Other material. **Occurrence:** catalogNumber: A0300; recordedBy: L. Fortini; individualCount: 1; sex: female; lifeStage: adult; occurrenceID: 03EB0190-4D9E-530C-BC0C-AB69D4C2B631; **Taxon:** scientificName: Bombus (Thoracobombus) pascuorum (Scopoli,1763); order: Hymenoptera; family: Apidae; genus: Bombus; subgenus: Thoracobombus; specificEpithet: pascuorum; scientificNameAuthorship: (Scopoli,1763); **Location:** country: Italy; countryCode: IT; stateProvince: Roma; locality: Riserva Naturale Laurentino-Acqua Acetosa; decimalLatitude: 41.8079275; decimalLongitude: 12.4685548; geodeticDatum: WGS84; coordinatePrecision: 0.0002; **Identification:** identifiedBy: L. Fortini; **Event:** eventDate: 2022-06-16; **Record Level:** collectionCode: UR3
- **Type status:** Other material. **Occurrence:** catalogNumber: A0281; recordedBy: L. Fortini; individualCount: 1; sex: female; lifeStage: adult; occurrenceID: 5E359953-4E41-5200-B674-7FC0DA126531; **Taxon:** scientificName: Bombus (Thoracobombus) pascuorum (Scopoli,1763); order: Hymenoptera; family: Apidae; genus: Bombus; subgenus: Thoracobombus; specificEpithet: pascuorum; scientificNameAuthorship: (Scopoli,1763); **Location:** country: Italy; countryCode: IT; stateProvince: Roma; locality: Riserva Naturale di Monte Mario; decimalLatitude: 41.9386215; decimalLongitude: 12.4546223; geodeticDatum: WGS84; coordinatePrecision: 0.0002; **Identification:** identifiedBy: L. Fortini; **Event:** eventDate: 2022-07-24; **Record Level:** collectionCode: UR3
- **Type status:** Other material. **Occurrence:** catalogNumber: A0270, A0277, A0286; recordedBy: L. Fortini; individualCount: 3; sex: females; lifeStage: adult; occurrenceID: 1AF6F274-C0FB-5B61-BB5B-3CD0FA922DE1; **Taxon:** scientificName: Bombus (Thoracobombus) pascuorum (Scopoli,1763); order: Hymenoptera; family: Apidae; genus: Bombus; subgenus: Thoracobombus; specificEpithet: pascuorum; scientificNameAuthorship: (Scopoli,1763); **Location:** country: Italy; countryCode: IT; stateProvince: Roma; locality: Riserva Naturale Valle dell'Aniene 2; decimalLatitude: 41.928752; decimalLongitude: 12.5562962; geodeticDatum: WGS84; coordinatePrecision: 0.0002; **Identification:** identifiedBy: L. Fortini; **Event:** eventDate: 2022-04-28; **Record Level:** collectionCode: UR3
- **Type status:** Other material. **Occurrence:** catalogNumber: A0297, A0319; recordedBy: L. Fortini; individualCount: 2; sex: females; lifeStage: adult; occurrenceID: 0C388792-8F62-5E95-A734-0D1F7A0A2BAD; **Taxon:** scientificName: Bombus (Thoracobombus) pascuorum (Scopoli,1763); order: Hymenoptera; family: Apidae; genus: Bombus; subgenus: Thoracobombus; specificEpithet: pascuorum; scientificNameAuthorship: (Scopoli,1763); **Location:** country: Italy; countryCode: IT; stateProvince: Roma; locality: Riserva Naturale Valle dell'Aniene 2; decimalLatitude: 41.928752; decimalLongitude: 12.5562962; geodeticDatum: WGS84; coordinatePrecision: 0.0002; **Identification:** identifiedBy: L. Fortini; **Event:** eventDate: 2022-06-05; **Record Level:** collectionCode: UR3
- **Type status:** Other material. **Occurrence:** catalogNumber: A0307, A0313, A0314, A0323; recordedBy: L. Fortini; individualCount: 4; sex: females; lifeStage: adult; occurrenceID: 529886E7-4052-5ADB-86C3-4339D46C1D3D; **Taxon:** scientificName: Bombus (Thoracobombus) pascuorum (Scopoli,1763); order: Hymenoptera; family: Apidae; genus: Bombus; subgenus: Thoracobombus; specificEpithet: pascuorum; scientificNameAuthorship: (Scopoli,1763); **Location:** country: Italy; countryCode: IT; stateProvince: Roma; locality: Riserva Naturale Valle dell'Aniene 2; decimalLatitude: 41.928752; decimalLongitude: 12.5562962; geodeticDatum: WGS84; coordinatePrecision: 0.0002; **Identification:** identifiedBy: L. Fortini; **Event:** eventDate: 2022-09-04; **Record Level:** collectionCode: UR3
- **Type status:** Other material. **Occurrence:** catalogNumber: A0271; recordedBy: L. Fortini; individualCount: 1; sex: female; lifeStage: adult; occurrenceID: 06D69AC8-A16B-5B2C-B4C7-E3D35B9FC66F; **Taxon:** scientificName: Bombus (Thoracobombus) pascuorum (Scopoli,1763); order: Hymenoptera; family: Apidae; genus: Bombus; subgenus: Thoracobombus; specificEpithet: pascuorum; scientificNameAuthorship: (Scopoli,1763); **Location:** country: Italy; countryCode: IT; stateProvince: Roma; locality: Riserva Naturale Valle dei Casali 1; decimalLatitude: 41.8710627; decimalLongitude: 12.4336809; geodeticDatum: WGS84; coordinatePrecision: 0.0002; **Identification:** identifiedBy: L. Fortini; **Event:** eventDate: 2022-04-07; **Record Level:** collectionCode: UR3
- **Type status:** Other material. **Occurrence:** catalogNumber: A0299; recordedBy: L. Fortini; individualCount: 1; sex: female; lifeStage: adult; occurrenceID: 1CE54BA9-A5EF-58DA-82E0-8150E8DA4D92; **Taxon:** scientificName: Bombus (Thoracobombus) pascuorum (Scopoli,1763); order: Hymenoptera; family: Apidae; genus: Bombus; subgenus: Thoracobombus; specificEpithet: pascuorum; scientificNameAuthorship: (Scopoli,1763); **Location:** country: Italy; countryCode: IT; stateProvince: Roma; locality: Riserva Naturale Valle dei Casali 1; decimalLatitude: 41.8710627; decimalLongitude: 12.4336809; geodeticDatum: WGS84; coordinatePrecision: 0.0002; **Identification:** identifiedBy: L. Fortini; **Event:** eventDate: 2022-09-18; **Record Level:** collectionCode: UR3
- **Type status:** Other material. **Occurrence:** catalogNumber: A0309, A0315, A0316, A0320, A0321; recordedBy: L. Fortini; individualCount: 4; sex: females; lifeStage: adult; occurrenceID: 0FD0C90B-62B7-58DE-9DFD-D4DAB4DC99F2; **Taxon:** scientificName: Bombus (Thoracobombus) pascuorum (Scopoli,1763); order: Hymenoptera; family: Apidae; genus: Bombus; subgenus: Thoracobombus; specificEpithet: pascuorum; scientificNameAuthorship: (Scopoli,1763); **Location:** country: Italy; countryCode: IT; stateProvince: Roma; locality: Riserva Naturale Valle dei Casali 1; decimalLatitude: 41.8710627; decimalLongitude: 12.4336809; geodeticDatum: WGS84; coordinatePrecision: 0.0002; **Identification:** identifiedBy: L. Fortini; **Event:** eventDate: 2022-07-13; **Record Level:** collectionCode: UR3
- **Type status:** Other material. **Occurrence:** catalogNumber: A1967; recordedBy: L. Fortini; individualCount: 1; sex: female; lifeStage: adult; occurrenceID: 8E8DBBC7-9E34-5802-8A46-8CCEC16D18E5; **Taxon:** scientificName: Bombus (Thoracobombus) pascuorum (Scopoli,1763); order: Hymenoptera; family: Apidae; genus: Bombus; subgenus: Thoracobombus; specificEpithet: pascuorum; scientificNameAuthorship: (Scopoli,1763); **Location:** country: Italy; countryCode: IT; stateProvince: Roma; locality: Riserva Regionale dell'Appia Antica 1; decimalLatitude: 41.8623941; decimalLongitude: 12.524863; geodeticDatum: WGS84; coordinatePrecision: 0.0002; **Identification:** identifiedBy: L. Fortini; **Event:** eventDate: 2022-04-19; **Record Level:** collectionCode: UR3
- **Type status:** Other material. **Occurrence:** catalogNumber: A2026; recordedBy: L. Fortini; individualCount: 1; sex: female; lifeStage: adult; occurrenceID: D79524C3-3B78-5623-A336-C721C7F3D411; **Taxon:** scientificName: Bombus (Thoracobombus) pascuorum (Scopoli,1763); order: Hymenoptera; family: Apidae; genus: Bombus; subgenus: Thoracobombus; specificEpithet: pascuorum; scientificNameAuthorship: (Scopoli,1763); **Location:** country: Italy; countryCode: IT; stateProvince: Roma; locality: Riserva Regionale dell'Appia Antica 2; decimalLatitude: 41.8402564; decimalLongitude: 12.532773; geodeticDatum: WGS84; coordinatePrecision: 0.0002; **Identification:** identifiedBy: L. Fortini; **Event:** eventDate: 2022-04-25; **Record Level:** collectionCode: UR3

#### Bombus ruderatus

(Fabricius, 1775)

20AFD618-8269-5D34-B69D-2C70E0467BC5

##### Materials

- **Type status:** Other material. **Occurrence:** catalogNumber: A0246, A0260, A0262; recordedBy: L. Fortini; individualCount: 3; sex: 1 male, 2 females; lifeStage: adult; occurrenceID: 66EBAE1D-6FED-5C43-B494-BC5E28094488; **Taxon:** scientificName: Bombus (Megabombus) ruderatus (Fabricius, 1775); order: Hymenoptera; family: Apidae; genus: Bombus; subgenus: Megabombus; specificEpithet: ruderatus; scientificNameAuthorship: (Fabricius, 1775); **Location:** country: Italy; countryCode: IT; stateProvince: Roma; locality: Riserva Naturale dell'Acquafredda; decimalLatitude: 41.8928408; decimalLongitude: 12.39932; geodeticDatum: WGS84; coordinatePrecision: 0.0002; **Identification:** identifiedBy: L. Fortini; **Event:** eventDate: 2022-06-10; **Record Level:** collectionCode: UR3
- **Type status:** Other material. **Occurrence:** catalogNumber: A0250, A0256; recordedBy: L. Fortini; individualCount: 2; sex: females; lifeStage: adult; occurrenceID: C82EBAB9-3BD6-56D7-8502-E5DA77AAA09B; **Taxon:** scientificName: Bombus (Megabombus) ruderatus (Fabricius, 1775); order: Hymenoptera; family: Apidae; genus: Bombus; subgenus: Megabombus; specificEpithet: ruderatus; scientificNameAuthorship: (Fabricius, 1775); **Location:** country: Italy; countryCode: IT; stateProvince: Roma; locality: Riserva Naturale dell'Acquafredda; decimalLatitude: 41.8928408; decimalLongitude: 12.39932; geodeticDatum: WGS84; coordinatePrecision: 0.0002; **Identification:** identifiedBy: L. Fortini; **Event:** eventDate: 2022-04-13; **Record Level:** collectionCode: UR3
- **Type status:** Other material. **Occurrence:** catalogNumber: A0247; recordedBy: L. Fortini; individualCount: 1; sex: male; lifeStage: adult; occurrenceID: 7F14EB65-729D-5DFD-A891-93596D6BB98E; **Taxon:** scientificName: Bombus (Megabombus) ruderatus (Fabricius, 1775); order: Hymenoptera; family: Apidae; genus: Bombus; subgenus: Megabombus; specificEpithet: ruderatus; scientificNameAuthorship: (Fabricius, 1775); **Location:** country: Italy; countryCode: IT; stateProvince: Roma; locality: Riserva Regionale dell'Appia Antica 3; decimalLatitude: 41.8298456; decimalLongitude: 12.5432538; geodeticDatum: WGS84; coordinatePrecision: 0.0002; **Identification:** identifiedBy: L. Fortini; **Event:** eventDate: 2022-05-24; **Record Level:** collectionCode: UR3
- **Type status:** Other material. **Occurrence:** catalogNumber: A0248, A0249, A0254, A0255, A0258, A0259, A0261; recordedBy: L. Fortini; individualCount: 7; sex: 2 males, 5 females; lifeStage: adult; occurrenceID: A6739628-AEF3-5642-91A7-B7FC39734F60; **Taxon:** scientificName: Bombus (Megabombus) ruderatus (Fabricius, 1775); order: Hymenoptera; family: Apidae; genus: Bombus; subgenus: Megabombus; specificEpithet: ruderatus; scientificNameAuthorship: (Fabricius, 1775); **Location:** country: Italy; countryCode: IT; stateProvince: Roma; locality: Riserva Regionale dell'Appia Antica 1; decimalLatitude: 41.8623941; decimalLongitude: 12.524863; geodeticDatum: WGS84; coordinatePrecision: 0.0002; **Identification:** identifiedBy: L. Fortini; **Event:** eventDate: 2022-06-12; **Record Level:** collectionCode: UR3
- **Type status:** Other material. **Occurrence:** catalogNumber: A0257; recordedBy: L. Fortini; individualCount: 1; sex: female; lifeStage: adult; occurrenceID: 2B875720-E890-5DC0-8126-152F98009D68; **Taxon:** scientificName: Bombus (Megabombus) ruderatus (Fabricius, 1775); order: Hymenoptera; family: Apidae; genus: Bombus; subgenus: Megabombus; specificEpithet: ruderatus; scientificNameAuthorship: (Fabricius, 1775); **Location:** country: Italy; countryCode: IT; stateProvince: Roma; locality: Riserva Naturale dell'Insugherata 1; decimalLatitude: 41.9555045; decimalLongitude: 12.4292321; geodeticDatum: WGS84; coordinatePrecision: 0.0002; **Identification:** identifiedBy: L. Fortini; **Event:** eventDate: 2022-05-27; **Record Level:** collectionCode: UR3
- **Type status:** Other material. **Occurrence:** catalogNumber: A0251; recordedBy: L. Fortini; individualCount: 1; sex: female; lifeStage: adult; occurrenceID: 1AD8185A-6472-5821-A1B0-7B3AC6F248BD; **Taxon:** scientificName: Bombus (Megabombus) ruderatus (Fabricius, 1775); order: Hymenoptera; family: Apidae; genus: Bombus; subgenus: Megabombus; specificEpithet: ruderatus; scientificNameAuthorship: (Fabricius, 1775); **Location:** country: Italy; countryCode: IT; stateProvince: Roma; locality: Riserva Naturale Laurentino-Acqua Acetosa; decimalLatitude: 41.8079275; decimalLongitude: 12.4685548; geodeticDatum: WGS84; coordinatePrecision: 0.0002; **Identification:** identifiedBy: L. Fortini; **Event:** eventDate: 2022-04-12; **Record Level:** collectionCode: UR3
- **Type status:** Other material. **Occurrence:** catalogNumber: A0253; recordedBy: L. Fortini; individualCount: 1; sex: female; lifeStage: adult; occurrenceID: F42BC3A9-3386-56B8-BF26-EA967375E7E7; **Taxon:** scientificName: Bombus (Megabombus) ruderatus (Fabricius, 1775); order: Hymenoptera; family: Apidae; genus: Bombus; subgenus: Megabombus; specificEpithet: ruderatus; scientificNameAuthorship: (Fabricius, 1775); **Location:** country: Italy; countryCode: IT; stateProvince: Roma; locality: Riserva Naturale Tenuta dei Massimi 2; decimalLatitude: 41.8316516; decimalLongitude: 12.3999927; geodeticDatum: WGS84; coordinatePrecision: 0.0002; **Identification:** identifiedBy: L. Fortini; **Event:** eventDate: 2022-06-01; **Record Level:** collectionCode: UR3
- **Type status:** Other material. **Occurrence:** catalogNumber: A0252; recordedBy: L. Fortini; individualCount: 1; sex: female; lifeStage: adult; occurrenceID: EC4E4B31-0756-5F3A-9AC7-8E217AE860A5; **Taxon:** scientificName: Bombus (Megabombus) ruderatus (Fabricius, 1775); order: Hymenoptera; family: Apidae; genus: Bombus; subgenus: Megabombus; specificEpithet: ruderatus; scientificNameAuthorship: (Fabricius, 1775); **Location:** country: Italy; countryCode: IT; stateProvince: Roma; locality: Riserva Naturale Valle dei Casali 1; decimalLatitude: 41.8710627; decimalLongitude: 12.4336809; geodeticDatum: WGS84; coordinatePrecision: 0.0002; **Identification:** identifiedBy: L. Fortini; **Event:** eventDate: 2022-04-07; **Record Level:** collectionCode: UR3
- **Type status:** Other material. **Occurrence:** catalogNumber: A2003; recordedBy: L. Fortini; individualCount: 1; sex: female; lifeStage: adult; occurrenceID: 6A187B5D-29AD-5F3C-ABE2-A65A15EAE657; **Taxon:** scientificName: Bombus (Megabombus) ruderatus (Fabricius, 1775); order: Hymenoptera; family: Apidae; genus: Bombus; subgenus: Megabombus; specificEpithet: ruderatus; scientificNameAuthorship: (Fabricius, 1775); **Location:** country: Italy; countryCode: IT; stateProvince: Roma; locality: Riserva Regionale dell'Appia Antica 1; decimalLatitude: 41.8623941; decimalLongitude: 12.524863; geodeticDatum: WGS84; coordinatePrecision: 0.0002; **Identification:** identifiedBy: L. Fortini; **Event:** eventDate: 2022-04-19; **Record Level:** collectionCode: UR3

#### Bombus terrestris

(Linnaeus, 1758)

05399715-E621-53A6-A71A-D6D42E61EBFE

##### Materials

- **Type status:** Other material. **Occurrence:** catalogNumber: A0206, A0211; recordedBy: L. Fortini; individualCount: 2; sex: females; lifeStage: adult; occurrenceID: 252FE685-B827-5A95-9600-A99E08E2A42C; **Taxon:** scientificName: Bombus (Bombus) terrestris (Linnaeus, 1758); order: Hymenoptera; family: Apidae; genus: Bombus; subgenus: Bombus; specificEpithet: terrestris; scientificNameAuthorship: (Linnaeus, 1758); **Location:** country: Italy; countryCode: IT; stateProvince: Roma; locality: Riserva Naturale dell'Acquafredda; decimalLatitude: 41.8928408; decimalLongitude: 12.39932; geodeticDatum: WGS84; coordinatePrecision: 0.0002; **Identification:** identifiedBy: L. Fortini; **Event:** eventDate: 2022-08-17; **Record Level:** collectionCode: UR3
- **Type status:** Other material. **Occurrence:** catalogNumber: A0215, A0243; recordedBy: L. Fortini; individualCount: 2; sex: females; lifeStage: adult; occurrenceID: 2CC53709-EFE6-5428-B97F-2D9E52644130; **Taxon:** scientificName: Bombus (Bombus) terrestris (Linnaeus, 1758); order: Hymenoptera; family: Apidae; genus: Bombus; subgenus: Bombus; specificEpithet: terrestris; scientificNameAuthorship: (Linnaeus, 1758); **Location:** country: Italy; countryCode: IT; stateProvince: Roma; locality: Riserva Naturale dell'Acquafredda; decimalLatitude: 41.8928408; decimalLongitude: 12.39932; geodeticDatum: WGS84; coordinatePrecision: 0.0002; **Identification:** identifiedBy: L. Fortini; **Event:** eventDate: 2022-06-10; **Record Level:** collectionCode: UR3
- **Type status:** Other material. **Occurrence:** catalogNumber: A0244; recordedBy: L. Fortini; individualCount: 1; sex: female; lifeStage: adult; occurrenceID: 0EE805B5-353E-56F6-B268-4515A11A8A33; **Taxon:** scientificName: Bombus (Bombus) terrestris (Linnaeus, 1758); order: Hymenoptera; family: Apidae; genus: Bombus; subgenus: Bombus; specificEpithet: terrestris; scientificNameAuthorship: (Linnaeus, 1758); **Location:** country: Italy; countryCode: IT; stateProvince: Roma; locality: Riserva Naturale dell'Acquafredda; decimalLatitude: 41.8928408; decimalLongitude: 12.39932; geodeticDatum: WGS84; coordinatePrecision: 0.0002; **Identification:** identifiedBy: L. Fortini; **Event:** eventDate: 2022-04-13; **Record Level:** collectionCode: UR3
- **Type status:** Other material. **Occurrence:** catalogNumber: A0242; recordedBy: L. Fortini; individualCount: 1; sex: female; lifeStage: adult; occurrenceID: 494202BF-8B4C-5572-9CC6-AB46E6A30C2E; **Taxon:** scientificName: Bombus (Bombus) terrestris (Linnaeus, 1758); order: Hymenoptera; family: Apidae; genus: Bombus; subgenus: Bombus; specificEpithet: terrestris; scientificNameAuthorship: (Linnaeus, 1758); **Location:** country: Italy; countryCode: IT; stateProvince: Roma; locality: Riserva Regionale dell'Appia Antica 1; decimalLatitude: 41.8623941; decimalLongitude: 12.524863; geodeticDatum: WGS84; coordinatePrecision: 0.0002; **Identification:** identifiedBy: L. Fortini; **Event:** eventDate: 2022-07-22; **Record Level:** collectionCode: UR3
- **Type status:** Other material. **Occurrence:** catalogNumber: A0191, A0205; recordedBy: L. Fortini; individualCount: 2; sex: 1 male, 2 females; lifeStage: adult; occurrenceID: FC57A686-54C5-5F87-A350-1BFE98F732D5; **Taxon:** scientificName: Bombus (Bombus) terrestris (Linnaeus, 1758); order: Hymenoptera; family: Apidae; genus: Bombus; subgenus: Bombus; specificEpithet: terrestris; scientificNameAuthorship: (Linnaeus, 1758); **Location:** country: Italy; countryCode: IT; stateProvince: Roma; locality: Riserva Naturale dell'Insugherata 1; decimalLatitude: 41.9555045; decimalLongitude: 12.4292321; geodeticDatum: WGS84; coordinatePrecision: 0.0002; **Identification:** identifiedBy: L. Fortini; **Event:** eventDate: 2022-07-30; **Record Level:** collectionCode: UR3
- **Type status:** Other material. **Occurrence:** catalogNumber: A0192, A0193, A0195; recordedBy: L. Fortini; individualCount: 3; sex: males; lifeStage: adult; occurrenceID: 89D13E96-7DAE-5AD0-984A-BA2A3ED757D9; **Taxon:** scientificName: Bombus (Bombus) terrestris (Linnaeus, 1758); order: Hymenoptera; family: Apidae; genus: Bombus; subgenus: Bombus; specificEpithet: terrestris; scientificNameAuthorship: (Linnaeus, 1758); **Location:** country: Italy; countryCode: IT; stateProvince: Roma; locality: Riserva Naturale dell'Insugherata 1; decimalLatitude: 41.9555045; decimalLongitude: 12.4292321; geodeticDatum: WGS84; coordinatePrecision: 0.0002; **Identification:** identifiedBy: L. Fortini; **Event:** eventDate: 2022-05-27; **Record Level:** collectionCode: UR3
- **Type status:** Other material. **Occurrence:** catalogNumber: A0212, A0214; recordedBy: L. Fortini; individualCount: 2; sex: females; lifeStage: adult; occurrenceID: 0A2F3F20-914E-50D5-9D4B-835F0D2656BD; **Taxon:** scientificName: Bombus (Bombus) terrestris (Linnaeus, 1758); order: Hymenoptera; family: Apidae; genus: Bombus; subgenus: Bombus; specificEpithet: terrestris; scientificNameAuthorship: (Linnaeus, 1758); **Location:** country: Italy; countryCode: IT; stateProvince: Roma; locality: Riserva Naturale dell'Insugherata 1; decimalLatitude: 41.9555045; decimalLongitude: 12.4292321; geodeticDatum: WGS84; coordinatePrecision: 0.0002; **Identification:** identifiedBy: L. Fortini; **Event:** eventDate: 2022-06-24; **Record Level:** collectionCode: UR3
- **Type status:** Other material. **Occurrence:** catalogNumber: A0194; recordedBy: L. Fortini; individualCount: 1; sex: male; lifeStage: adult; occurrenceID: D64BBE85-4914-5051-B8CC-43AC6D305955; **Taxon:** scientificName: Bombus (Bombus) terrestris (Linnaeus, 1758); order: Hymenoptera; family: Apidae; genus: Bombus; subgenus: Bombus; specificEpithet: terrestris; scientificNameAuthorship: (Linnaeus, 1758); **Location:** country: Italy; countryCode: IT; stateProvince: Roma; locality: Riserva Naturale dell'Insugherata 2; decimalLatitude: 41.9599247; decimalLongitude: 12.433852; geodeticDatum: WGS84; coordinatePrecision: 0.0002; **Identification:** identifiedBy: L. Fortini; **Event:** eventDate: 2022-05-27; **Record Level:** collectionCode: UR3
- **Type status:** Other material. **Occurrence:** catalogNumber: A0199, A0200; recordedBy: L. Fortini; individualCount: 2; sex: males; lifeStage: adult; occurrenceID: FF92BC75-CA92-5C6D-9D64-4042B8C62F00; **Taxon:** scientificName: Bombus (Bombus) terrestris (Linnaeus, 1758); order: Hymenoptera; family: Apidae; genus: Bombus; subgenus: Bombus; specificEpithet: terrestris; scientificNameAuthorship: (Linnaeus, 1758); **Location:** country: Italy; countryCode: IT; stateProvince: Roma; locality: Riserva Naturale dell'Insugherata 2; decimalLatitude: 41.9599247; decimalLongitude: 12.433852; geodeticDatum: WGS84; coordinatePrecision: 0.0002; **Identification:** identifiedBy: L. Fortini; **Event:** eventDate: 2022-07-30; **Record Level:** collectionCode: UR3
- **Type status:** Other material. **Occurrence:** catalogNumber: A0203, A0207, A0210, A0213; recordedBy: L. Fortini; individualCount: 4; sex: females; lifeStage: adult; occurrenceID: 56C403AF-900F-55FB-BC7A-7F873E0B1E30; **Taxon:** scientificName: Bombus (Bombus) terrestris (Linnaeus, 1758); order: Hymenoptera; family: Apidae; genus: Bombus; subgenus: Bombus; specificEpithet: terrestris; scientificNameAuthorship: (Linnaeus, 1758); **Location:** country: Italy; countryCode: IT; stateProvince: Roma; locality: Riserva Naturale dell'Insugherata 2; decimalLatitude: 41.9599247; decimalLongitude: 12.433852; geodeticDatum: WGS84; coordinatePrecision: 0.0002; **Identification:** identifiedBy: L. Fortini; **Event:** eventDate: 2022-06-24; **Record Level:** collectionCode: UR3
- **Type status:** Other material. **Occurrence:** catalogNumber: A0196, A0201, A 0209; recordedBy: L. Fortini; individualCount: 3; sex: 1 male, 2 females; lifeStage: adult; occurrenceID: DB8AC4AB-2208-5010-B2EB-8062D5C133F3; **Taxon:** scientificName: Bombus (Bombus) terrestris (Linnaeus, 1758); order: Hymenoptera; family: Apidae; genus: Bombus; subgenus: Bombus; specificEpithet: terrestris; scientificNameAuthorship: (Linnaeus, 1758); **Location:** country: Italy; countryCode: IT; stateProvince: Roma; locality: Riserva Naturale Laurentino-Acqua Acetosa; decimalLatitude: 41.8079275; decimalLongitude: 12.4685548; geodeticDatum: WGS84; coordinatePrecision: 0.0002; **Identification:** identifiedBy: L. Fortini; **Event:** eventDate: 2022-06-16; **Record Level:** collectionCode: UR3
- **Type status:** Other material. **Occurrence:** catalogNumber: A0198; recordedBy: L. Fortini; individualCount: 1; sex: male; lifeStage: adult; occurrenceID: 3B697297-AA74-54F3-B63C-53FA55DA6365; **Taxon:** scientificName: Bombus (Bombus) terrestris (Linnaeus, 1758); order: Hymenoptera; family: Apidae; genus: Bombus; subgenus: Bombus; specificEpithet: terrestris; scientificNameAuthorship: (Linnaeus, 1758); **Location:** country: Italy; countryCode: IT; stateProvince: Roma; locality: Riserva Naturale di Monte Mario; decimalLatitude: 41.9386215; decimalLongitude: 12.4546223; geodeticDatum: WGS84; coordinatePrecision: 0.0002; **Identification:** identifiedBy: L. Fortini; **Event:** eventDate: 2022-07-19; **Record Level:** collectionCode: UR3
- **Type status:** Other material. **Occurrence:** catalogNumber: A0216; recordedBy: L. Fortini; individualCount: 1; sex: female; lifeStage: adult; occurrenceID: B6715D51-A280-5728-A6EB-B18928DD6A64; **Taxon:** scientificName: Bombus (Bombus) terrestris (Linnaeus, 1758); order: Hymenoptera; family: Apidae; genus: Bombus; subgenus: Bombus; specificEpithet: terrestris; scientificNameAuthorship: (Linnaeus, 1758); **Location:** country: Italy; countryCode: IT; stateProvince: Roma; locality: Riserva Naturale di Monte Mario; decimalLatitude: 41.9386215; decimalLongitude: 12.4546223; geodeticDatum: WGS84; coordinatePrecision: 0.0002; **Identification:** identifiedBy: L. Fortini; **Event:** eventDate: 2022-04-20; **Record Level:** collectionCode: UR3
- **Type status:** Other material. **Occurrence:** catalogNumber: A0241; recordedBy: L. Fortini; individualCount: 1; sex: female; lifeStage: adult; occurrenceID: 507F7321-73DE-55C7-90DF-E3019471DF71; **Taxon:** scientificName: Bombus (Bombus) terrestris (Linnaeus, 1758); order: Hymenoptera; family: Apidae; genus: Bombus; subgenus: Bombus; specificEpithet: terrestris; scientificNameAuthorship: (Linnaeus, 1758); **Location:** country: Italy; countryCode: IT; stateProvince: Roma; locality: Riserva Naturale di Monte Mario; decimalLatitude: 41.9386215; decimalLongitude: 12.4546223; geodeticDatum: WGS84; coordinatePrecision: 0.0002; **Identification:** identifiedBy: L. Fortini; **Event:** eventDate: 2022-06-19; **Record Level:** collectionCode: UR3
- **Type status:** Other material. **Occurrence:** catalogNumber: A0245; recordedBy: L. Fortini; individualCount: 1; sex: female; lifeStage: adult; occurrenceID: B850C62C-D681-53A9-827B-BB3B08FB226D; **Taxon:** scientificName: Bombus (Bombus) terrestris (Linnaeus, 1758); order: Hymenoptera; family: Apidae; genus: Bombus; subgenus: Bombus; specificEpithet: terrestris; scientificNameAuthorship: (Linnaeus, 1758); **Location:** country: Italy; countryCode: IT; stateProvince: Roma; locality: Riserva Naturale Tenuta dei Massimi 2; decimalLatitude: 41.8316516; decimalLongitude: 12.3999927; geodeticDatum: WGS84; coordinatePrecision: 0.0002; **Identification:** identifiedBy: L. Fortini; **Event:** eventDate: 2022-06-01; **Record Level:** collectionCode: UR3
- **Type status:** Other material. **Occurrence:** catalogNumber: A0197; recordedBy: L. Fortini; individualCount: 1; sex: male; lifeStage: adult; occurrenceID: 303C3657-97DC-5D30-9A6D-B4B8820A440E; **Taxon:** scientificName: Bombus (Bombus) terrestris (Linnaeus, 1758); order: Hymenoptera; family: Apidae; genus: Bombus; subgenus: Bombus; specificEpithet: terrestris; scientificNameAuthorship: (Linnaeus, 1758); **Location:** country: Italy; countryCode: IT; stateProvince: Roma; locality: Riserva Naturale Valle dell'Aniene 2; decimalLatitude: 41.928752; decimalLongitude: 12.5562962; geodeticDatum: WGS84; coordinatePrecision: 0.0002; **Identification:** identifiedBy: L. Fortini; **Event:** eventDate: 2022-07-05; **Record Level:** collectionCode: UR3
- **Type status:** Other material. **Occurrence:** catalogNumber: A0202, A0204; recordedBy: L. Fortini; individualCount: 2; sex: females; lifeStage: adult; occurrenceID: 27245525-4F20-5B31-9985-D3971E825F16; **Taxon:** scientificName: Bombus (Bombus) terrestris (Linnaeus, 1758); order: Hymenoptera; family: Apidae; genus: Bombus; subgenus: Bombus; specificEpithet: terrestris; scientificNameAuthorship: (Linnaeus, 1758); **Location:** country: Italy; countryCode: IT; stateProvince: Roma; locality: Riserva Naturale Valle dell'Aniene 2; decimalLatitude: 41.928752; decimalLongitude: 12.5562962; geodeticDatum: WGS84; coordinatePrecision: 0.0002; **Identification:** identifiedBy: L. Fortini; **Event:** eventDate: 2022-04-28; **Record Level:** collectionCode: UR3
- **Type status:** Other material. **Occurrence:** catalogNumber: A0208; recordedBy: L. Fortini; individualCount: 1; sex: female; lifeStage: adult; occurrenceID: DF983F8D-F51B-5FCD-8B27-99BAFAB7BB81; **Taxon:** scientificName: Bombus (Bombus) terrestris (Linnaeus, 1758); order: Hymenoptera; family: Apidae; genus: Bombus; subgenus: Bombus; specificEpithet: terrestris; scientificNameAuthorship: (Linnaeus, 1758); **Location:** country: Italy; countryCode: IT; stateProvince: Roma; locality: Riserva Naturale Valle dell'Aniene 2; decimalLatitude: 41.928752; decimalLongitude: 12.5562962; geodeticDatum: WGS84; coordinatePrecision: 0.0002; **Identification:** identifiedBy: L. Fortini; **Event:** eventDate: 2022-06-05; **Record Level:** collectionCode: UR3
- **Type status:** Other material. **Occurrence:** catalogNumber: A2063; recordedBy: L. Fortini; individualCount: 1; sex: female; lifeStage: adult; occurrenceID: F3DE2E43-7EAA-535C-A562-2F8946B3BCE9; **Taxon:** scientificName: Bombus (Bombus) terrestris (Linnaeus, 1758); order: Hymenoptera; family: Apidae; genus: Bombus; subgenus: Bombus; specificEpithet: terrestris; scientificNameAuthorship: (Linnaeus, 1758); **Location:** country: Italy; countryCode: IT; stateProvince: Roma; locality: Riserva Naturale di Monte Mario; decimalLatitude: 41.9386215; decimalLongitude: 12.4546223; geodeticDatum: WGS84; coordinatePrecision: 0.0002; **Identification:** identifiedBy: L. Fortini; **Event:** eventDate: 2022-07-24; **Record Level:** collectionCode: UR3
- **Type status:** Other material. **Occurrence:** catalogNumber: A2097, A2098, A2099, A2105, A2106, A2107, A2110; recordedBy: L. Fortini; individualCount: 7; sex: females; lifeStage: adult; occurrenceID: F1F37226-43AB-50E3-8DE5-924ECC2BD90D; **Taxon:** scientificName: Bombus (Bombus) terrestris (Linnaeus, 1758); order: Hymenoptera; family: Apidae; genus: Bombus; subgenus: Bombus; specificEpithet: terrestris; scientificNameAuthorship: (Linnaeus, 1758); **Location:** country: Italy; countryCode: IT; stateProvince: Roma; locality: Riserva Naturale Valle dell'Aniene 1; decimalLatitude: 41.9345179; decimalLongitude: 12.5453096; geodeticDatum: WGS84; coordinatePrecision: 0.0002; **Identification:** identifiedBy: L. Fortini; **Event:** eventDate: 2022-08-03; **Record Level:** collectionCode: UR3
- **Type status:** Other material. **Occurrence:** catalogNumber: A2138; recordedBy: L. Fortini; individualCount: 1; sex: female; lifeStage: adult; occurrenceID: C59EB8C1-612F-5A5F-9005-B3F08CD6643B; **Taxon:** scientificName: Bombus (Bombus) terrestris (Linnaeus, 1758); order: Hymenoptera; family: Apidae; genus: Bombus; subgenus: Bombus; specificEpithet: terrestris; scientificNameAuthorship: (Linnaeus, 1758); **Location:** country: Italy; countryCode: IT; stateProvince: Roma; locality: Riserva Naturale Valle dell'Aniene 2; decimalLatitude: 41.928752; decimalLongitude: 12.5562962; geodeticDatum: WGS84; coordinatePrecision: 0.0002; **Identification:** identifiedBy: L. Fortini; **Event:** eventDate: 2022-08-03; **Record Level:** collectionCode: UR3

#### Ceratina chalcites

Germar, 1839

567FFA2C-B291-53EC-A777-B282577DBC87

##### Materials

- **Type status:** Other material. **Occurrence:** catalogNumber: A0044; recordedBy: L. Fortini; individualCount: 1; sex: female; lifeStage: adult; occurrenceID: 667DE3DC-A81A-535A-B520-3B816A2F71C5; **Taxon:** scientificName: Ceratina (Euceratina) chalcites Germar, 1839; order: Hymenoptera; family: Apidae; genus: Ceratina; subgenus: Euceratina; specificEpithet: chalcites; scientificNameAuthorship: Germar, 1839; **Location:** country: Italy; countryCode: IT; stateProvince: Roma; locality: Riserva Regionale dell'Appia Antica 1; decimalLatitude: 41.8623941; decimalLongitude: 12.524863; geodeticDatum: WGS84; coordinatePrecision: 0.0002; **Identification:** identifiedBy: L. Fortini; **Event:** eventDate: 2022-05-10; **Record Level:** collectionCode: UR3
- **Type status:** Other material. **Occurrence:** catalogNumber: A0041, A0045; recordedBy: L. Fortini; individualCount: 2; sex: 1 male, 1 female; lifeStage: adult; occurrenceID: 7988AB58-C68D-54FC-9E02-BAE258DD5337; **Taxon:** scientificName: Ceratina (Euceratina) chalcites Germar, 1839; order: Hymenoptera; family: Apidae; genus: Ceratina; subgenus: Euceratina; specificEpithet: chalcites; scientificNameAuthorship: Germar, 1839; **Location:** country: Italy; countryCode: IT; stateProvince: Roma; locality: Riserva Naturale dell'Insugherata 3; decimalLatitude: 41.9644829; decimalLongitude: 12.436101; geodeticDatum: WGS84; coordinatePrecision: 0.0002; **Identification:** identifiedBy: L. Fortini; **Event:** eventDate: 2022-05-27; **Record Level:** collectionCode: UR3
- **Type status:** Other material. **Occurrence:** catalogNumber: A0042; recordedBy: L. Fortini; individualCount: 1; sex: female; lifeStage: adult; occurrenceID: 199D7F28-DB20-5276-B6ED-65C87E70BF03; **Taxon:** scientificName: Ceratina (Euceratina) chalcites Germar, 1839; order: Hymenoptera; family: Apidae; genus: Ceratina; subgenus: Euceratina; specificEpithet: chalcites; scientificNameAuthorship: Germar, 1839; **Location:** country: Italy; countryCode: IT; stateProvince: Roma; locality: Riserva Naturale Laurentino-Acqua Acetosa; decimalLatitude: 41.8079275; decimalLongitude: 12.4685548; geodeticDatum: WGS84; coordinatePrecision: 0.0002; **Identification:** identifiedBy: L. Fortini; **Event:** eventDate: 2022-07-17; **Record Level:** collectionCode: UR3
- **Type status:** Other material. **Occurrence:** catalogNumber: A0039; recordedBy: L. Fortini; individualCount: 1; sex: male; lifeStage: adult; occurrenceID: 7BA9CC65-059B-5E05-9DCD-5195F488A692; **Taxon:** scientificName: Ceratina (Euceratina) chalcites Germar, 1839; order: Hymenoptera; family: Apidae; genus: Ceratina; subgenus: Euceratina; specificEpithet: chalcites; scientificNameAuthorship: Germar, 1839; **Location:** country: Italy; countryCode: IT; stateProvince: Roma; locality: Riserva Naturale di Monte Mario; decimalLatitude: 41.9386215; decimalLongitude: 12.4546223; geodeticDatum: WGS84; coordinatePrecision: 0.0002; **Identification:** identifiedBy: L. Fortini; **Event:** eventDate: 2022-05-20; **Record Level:** collectionCode: UR3
- **Type status:** Other material. **Occurrence:** catalogNumber: A0040, A0043; recordedBy: L. Fortini; individualCount: 2; sex: 1 male, 1 female; lifeStage: adult; occurrenceID: 201E1140-3B31-58BB-9930-38A4AF9116AE; **Taxon:** scientificName: Ceratina (Euceratina) chalcites Germar, 1839; order: Hymenoptera; family: Apidae; genus: Ceratina; subgenus: Euceratina; specificEpithet: chalcites; scientificNameAuthorship: Germar, 1839; **Location:** country: Italy; countryCode: IT; stateProvince: Roma; locality: Riserva Naturale Valle dei Casali 1; decimalLatitude: 41.8710627; decimalLongitude: 12.4336809; geodeticDatum: WGS84; coordinatePrecision: 0.0002; **Identification:** identifiedBy: L. Fortini; **Event:** eventDate: 2022-07-13; **Record Level:** collectionCode: UR3
- **Type status:** Other material. **Occurrence:** catalogNumber: A2089; recordedBy: L. Fortini; individualCount: 1; sex: female; lifeStage: adult; occurrenceID: 3211C056-01BC-5EF9-B61E-E7A860234348; **Taxon:** scientificName: Ceratina (Euceratina) chalcites Germar, 1839; order: Hymenoptera; family: Apidae; genus: Ceratina; subgenus: Euceratina; specificEpithet: chalcites; scientificNameAuthorship: Germar, 1839; **Location:** country: Italy; countryCode: IT; stateProvince: Roma; locality: Riserva Regionale dell'Appia Antica 2; decimalLatitude: 41.8402564; decimalLongitude: 12.532773; geodeticDatum: WGS84; coordinatePrecision: 0.0002; **Identification:** identifiedBy: L. Fortini; **Event:** eventDate: 2022-08-06; **Record Level:** collectionCode: UR3

#### Ceratina cucurbitina

(Rossi, 1972)

F8328E1E-0D24-50DD-8388-B61BD6B38113

##### Materials

- **Type status:** Other material. **Occurrence:** catalogNumber: A0050, A0051, A0058, A0065, A0075, A0079, A0090; recordedBy: L. Fortini; individualCount: 7; sex: 3 males, 4 females; lifeStage: adult; occurrenceID: FA9C88D1-4AA0-5F64-9A5E-B0E134076E20; **Taxon:** scientificName: Ceratina (Ceratina) cucurbitina (Rossi, 1792); order: Hymenoptera; family: Apidae; genus: Ceratina; subgenus: Ceratina; specificEpithet: cucurbitina; scientificNameAuthorship: (Rossi, 1792); **Location:** country: Italy; countryCode: IT; stateProvince: Roma; locality: Riserva Naturale dell'Acquafredda; decimalLatitude: 41.8928408; decimalLongitude: 12.39932; geodeticDatum: WGS84; coordinatePrecision: 0.0002; **Identification:** identifiedBy: L. Fortini; **Event:** eventDate: 2022-04-13; **Record Level:** collectionCode: UR3
- **Type status:** Other material. **Occurrence:** catalogNumber: A0049, A0054, A0059, A0061; recordedBy: L. Fortini; individualCount: 4; sex: males; lifeStage: adult; occurrenceID: 3D12AFEA-C84C-5792-9252-A13DD659E38C; **Taxon:** scientificName: Ceratina (Ceratina) cucurbitina (Rossi, 1792); order: Hymenoptera; family: Apidae; genus: Ceratina; subgenus: Ceratina; specificEpithet: cucurbitina; scientificNameAuthorship: (Rossi, 1792); **Location:** country: Italy; countryCode: IT; stateProvince: Roma; locality: Riserva Regionale dell'Appia Antica 1; decimalLatitude: 41.8623941; decimalLongitude: 12.524863; geodeticDatum: WGS84; coordinatePrecision: 0.0002; **Identification:** identifiedBy: L. Fortini; **Event:** eventDate: 2022-05-10; **Record Level:** collectionCode: UR3
- **Type status:** Other material. **Occurrence:** catalogNumber: A0074, A0086; recordedBy: L. Fortini; individualCount: 2; sex: females; lifeStage: adult; occurrenceID: C6978518-B8D8-5ED2-81F1-B4516875EC17; **Taxon:** scientificName: Ceratina (Ceratina) cucurbitina (Rossi, 1792); order: Hymenoptera; family: Apidae; genus: Ceratina; subgenus: Ceratina; specificEpithet: cucurbitina; scientificNameAuthorship: (Rossi, 1792); **Location:** country: Italy; countryCode: IT; stateProvince: Roma; locality: Riserva Regionale dell'Appia Antica 1; decimalLatitude: 41.8623941; decimalLongitude: 12.524863; geodeticDatum: WGS84; coordinatePrecision: 0.0002; **Identification:** identifiedBy: L. Fortini; **Event:** eventDate: 2022-07-22; **Record Level:** collectionCode: UR3
- **Type status:** Other material. **Occurrence:** catalogNumber: A0052; recordedBy: L. Fortini; individualCount: 1; sex: male; lifeStage: adult; occurrenceID: DEB0BF34-2722-527A-9F01-839200C1B3D2; **Taxon:** scientificName: Ceratina (Ceratina) cucurbitina (Rossi, 1792); order: Hymenoptera; family: Apidae; genus: Ceratina; subgenus: Ceratina; specificEpithet: cucurbitina; scientificNameAuthorship: (Rossi, 1792); **Location:** country: Italy; countryCode: IT; stateProvince: Roma; locality: Riserva Regionale dell'Appia Antica 2; decimalLatitude: 41.8402564; decimalLongitude: 12.532773; geodeticDatum: WGS84; coordinatePrecision: 0.0002; **Identification:** identifiedBy: L. Fortini; **Event:** eventDate: 2022-10-01; **Record Level:** collectionCode: UR3
- **Type status:** Other material. **Occurrence:** catalogNumber: A0063; recordedBy: L. Fortini; individualCount: 1; sex: female; lifeStage: adult; occurrenceID: 2812A8BD-C098-5F8D-8386-F3343F6E4FF1; **Taxon:** scientificName: Ceratina (Ceratina) cucurbitina (Rossi, 1792); order: Hymenoptera; family: Apidae; genus: Ceratina; subgenus: Ceratina; specificEpithet: cucurbitina; scientificNameAuthorship: (Rossi, 1792); **Location:** country: Italy; countryCode: IT; stateProvince: Roma; locality: Riserva Regionale dell'Appia Antica 2; decimalLatitude: 41.8402564; decimalLongitude: 12.532773; geodeticDatum: WGS84; coordinatePrecision: 0.0002; **Identification:** identifiedBy: L. Fortini; **Event:** eventDate: 2022-07-07; **Record Level:** collectionCode: UR3
- **Type status:** Other material. **Occurrence:** catalogNumber: A0069, A0070; recordedBy: L. Fortini; individualCount: 2; sex: females; lifeStage: adult; occurrenceID: 0FD66ED4-2878-5671-80ED-E90617D0335D; **Taxon:** scientificName: Ceratina (Ceratina) cucurbitina (Rossi, 1792); order: Hymenoptera; family: Apidae; genus: Ceratina; subgenus: Ceratina; specificEpithet: cucurbitina; scientificNameAuthorship: (Rossi, 1792); **Location:** country: Italy; countryCode: IT; stateProvince: Roma; locality: Riserva Regionale dell'Appia Antica 2; decimalLatitude: 41.8402564; decimalLongitude: 12.532773; geodeticDatum: WGS84; coordinatePrecision: 0.0002; **Identification:** identifiedBy: L. Fortini; **Event:** eventDate: 2022-08-29; **Record Level:** collectionCode: UR3
- **Type status:** Other material. **Occurrence:** catalogNumber: A0067; recordedBy: L. Fortini; individualCount: 1; sex: female; lifeStage: adult; occurrenceID: BA4DA449-5BEF-5C86-9EB5-1EA3C8947D11; **Taxon:** scientificName: Ceratina (Ceratina) cucurbitina (Rossi, 1792); order: Hymenoptera; family: Apidae; genus: Ceratina; subgenus: Ceratina; specificEpithet: cucurbitina; scientificNameAuthorship: (Rossi, 1792); **Location:** country: Italy; countryCode: IT; stateProvince: Roma; locality: Riserva Regionale dell'Appia Antica 3; decimalLatitude: 41.8298456; decimalLongitude: 12.5432538; geodeticDatum: WGS84; coordinatePrecision: 0.0002; **Identification:** identifiedBy: L. Fortini; **Event:** eventDate: 2022-05-24; **Record Level:** collectionCode: UR3
- **Type status:** Other material. **Occurrence:** catalogNumber: A0056; recordedBy: L. Fortini; individualCount: 1; sex: male; lifeStage: adult; occurrenceID: 88DB5910-FDB4-50A0-8D67-76A5D69E3F27; **Taxon:** scientificName: Ceratina (Ceratina) cucurbitina (Rossi, 1792); order: Hymenoptera; family: Apidae; genus: Ceratina; subgenus: Ceratina; specificEpithet: cucurbitina; scientificNameAuthorship: (Rossi, 1792); **Location:** country: Italy; countryCode: IT; stateProvince: Roma; locality: Riserva Naturale dell'Insugherata 2; decimalLatitude: 41.9599247; decimalLongitude: 12.433852; geodeticDatum: WGS84; coordinatePrecision: 0.0002; **Identification:** identifiedBy: L. Fortini; **Event:** eventDate: 2022-04-15; **Record Level:** collectionCode: UR3
- **Type status:** Other material. **Occurrence:** catalogNumber: A0088; recordedBy: L. Fortini; individualCount: 1; sex: female; lifeStage: adult; occurrenceID: 98AC6CF4-04E7-530E-AA45-2A2F5F3D750F; **Taxon:** scientificName: Ceratina (Ceratina) cucurbitina (Rossi, 1792); order: Hymenoptera; family: Apidae; genus: Ceratina; subgenus: Ceratina; specificEpithet: cucurbitina; scientificNameAuthorship: (Rossi, 1792); **Location:** country: Italy; countryCode: IT; stateProvince: Roma; locality: Riserva Naturale dell'Insugherata 2; decimalLatitude: 41.9599247; decimalLongitude: 12.433852; geodeticDatum: WGS84; coordinatePrecision: 0.0002; **Identification:** identifiedBy: L. Fortini; **Event:** eventDate: 2022-09-01; **Record Level:** collectionCode: UR3
- **Type status:** Other material. **Occurrence:** catalogNumber: A0055, A0057; recordedBy: L. Fortini; individualCount: 2; sex: males; lifeStage: adult; occurrenceID: E09F4F9A-A819-5923-AA38-2526CDC7A327; **Taxon:** scientificName: Ceratina (Ceratina) cucurbitina (Rossi, 1792); order: Hymenoptera; family: Apidae; genus: Ceratina; subgenus: Ceratina; specificEpithet: cucurbitina; scientificNameAuthorship: (Rossi, 1792); **Location:** country: Italy; countryCode: IT; stateProvince: Roma; locality: Riserva Naturale dell'Insugherata 3; decimalLatitude: 41.9644829; decimalLongitude: 12.436101; geodeticDatum: WGS84; coordinatePrecision: 0.0002; **Identification:** identifiedBy: L. Fortini; **Event:** eventDate: 2022-04-15; **Record Level:** collectionCode: UR3
- **Type status:** Other material. **Occurrence:** catalogNumber: A0083; recordedBy: L. Fortini; individualCount: 1; sex: female; lifeStage: adult; occurrenceID: 9E84CA1B-6C36-5655-BAF0-78BFC6EDC1A4; **Taxon:** scientificName: Ceratina (Ceratina) cucurbitina (Rossi, 1792); order: Hymenoptera; family: Apidae; genus: Ceratina; subgenus: Ceratina; specificEpithet: cucurbitina; scientificNameAuthorship: (Rossi, 1792); **Location:** country: Italy; countryCode: IT; stateProvince: Roma; locality: Riserva Naturale Laurentino-Acqua Acetosa; decimalLatitude: 41.8079275; decimalLongitude: 12.4685548; geodeticDatum: WGS84; coordinatePrecision: 0.0002; **Identification:** identifiedBy: L. Fortini; **Event:** eventDate: 2022-06-16; **Record Level:** collectionCode: UR3
- **Type status:** Other material. **Occurrence:** catalogNumber: A0087, A0091; recordedBy: L. Fortini; individualCount: 2; sex: females; lifeStage: adult; occurrenceID: 878873DC-8E30-55CB-957C-F734EB93FDF5; **Taxon:** scientificName: Ceratina (Ceratina) cucurbitina (Rossi, 1792); order: Hymenoptera; family: Apidae; genus: Ceratina; subgenus: Ceratina; specificEpithet: cucurbitina; scientificNameAuthorship: (Rossi, 1792); **Location:** country: Italy; countryCode: IT; stateProvince: Roma; locality: Riserva Naturale Laurentino-Acqua Acetosa; decimalLatitude: 41.8079275; decimalLongitude: 12.4685548; geodeticDatum: WGS84; coordinatePrecision: 0.0002; **Identification:** identifiedBy: L. Fortini; **Event:** eventDate: 2022-07-17; **Record Level:** collectionCode: UR3
- **Type status:** Other material. **Occurrence:** catalogNumber: A0048, A0072; recordedBy: L. Fortini; individualCount: 2; sex: 1 male, 1 female; lifeStage: adult; occurrenceID: B24F9C49-80A9-5389-92A4-E9D51487CD72; **Taxon:** scientificName: Ceratina (Ceratina) cucurbitina (Rossi, 1792); order: Hymenoptera; family: Apidae; genus: Ceratina; subgenus: Ceratina; specificEpithet: cucurbitina; scientificNameAuthorship: (Rossi, 1792); **Location:** country: Italy; countryCode: IT; stateProvince: Roma; locality: Riserva Naturale Laurentino-Acqua Acetosa; decimalLatitude: 41.8079275; decimalLongitude: 12.4685548; geodeticDatum: WGS84; coordinatePrecision: 0.0002; **Identification:** identifiedBy: L. Fortini; **Event:** eventDate: 2022-08-21; **Record Level:** collectionCode: UR3
- **Type status:** Other material. **Occurrence:** catalogNumber: A0082, A0089; recordedBy: L. Fortini; individualCount: 2; sex: females; lifeStage: adult; occurrenceID: 3E9F2245-2FD9-5C22-AAA5-35AFF182C7CA; **Taxon:** scientificName: Ceratina (Ceratina) cucurbitina (Rossi, 1792); order: Hymenoptera; family: Apidae; genus: Ceratina; subgenus: Ceratina; specificEpithet: cucurbitina; scientificNameAuthorship: (Rossi, 1792); **Location:** country: Italy; countryCode: IT; stateProvince: Roma; locality: Riserva Naturale Laurentino-Acqua Acetosa; decimalLatitude: 41.8079275; decimalLongitude: 12.4685548; geodeticDatum: WGS84; coordinatePrecision: 0.0002; **Identification:** identifiedBy: L. Fortini; **Event:** eventDate: 2022-09-14; **Record Level:** collectionCode: UR3
- **Type status:** Other material. **Occurrence:** catalogNumber: A0053; recordedBy: L. Fortini; individualCount: 1; sex: male; lifeStage: adult; occurrenceID: 190F7DBD-6CFE-5FF6-92A4-3FECB47E4E44; **Taxon:** scientificName: Ceratina (Ceratina) cucurbitina (Rossi, 1792); order: Hymenoptera; family: Apidae; genus: Ceratina; subgenus: Ceratina; specificEpithet: cucurbitina; scientificNameAuthorship: (Rossi, 1792); **Location:** country: Italy; countryCode: IT; stateProvince: Roma; locality: Riserva Naturale di Monte Mario; decimalLatitude: 41.9386215; decimalLongitude: 12.4546223; geodeticDatum: WGS84; coordinatePrecision: 0.0002; **Identification:** identifiedBy: L. Fortini; **Event:** eventDate: 2022-04-20; **Record Level:** collectionCode: UR3
- **Type status:** Other material. **Occurrence:** catalogNumber: A0046, A0068; recordedBy: L. Fortini; individualCount: 2; sex: 1 male, 1 female; lifeStage: adult; occurrenceID: 0C4AF20F-8FD4-5AC4-9926-D89C7D8053F3; **Taxon:** scientificName: Ceratina (Ceratina) cucurbitina (Rossi, 1792); order: Hymenoptera; family: Apidae; genus: Ceratina; subgenus: Ceratina; specificEpithet: cucurbitina; scientificNameAuthorship: (Rossi, 1792); **Location:** country: Italy; countryCode: IT; stateProvince: Roma; locality: Riserva Naturale di Monte Mario; decimalLatitude: 41.9386215; decimalLongitude: 12.4546223; geodeticDatum: WGS84; coordinatePrecision: 0.0002; **Identification:** identifiedBy: L. Fortini; **Event:** eventDate: 2022-05-20; **Record Level:** collectionCode: UR3
- **Type status:** Other material. **Occurrence:** catalogNumber: A0064, A0076; recordedBy: L. Fortini; individualCount: 2; sex: females; lifeStage: adult; occurrenceID: 6BB7AFB9-7EA8-513E-B52C-24AD67B224F6; **Taxon:** scientificName: Ceratina (Ceratina) cucurbitina (Rossi, 1792); order: Hymenoptera; family: Apidae; genus: Ceratina; subgenus: Ceratina; specificEpithet: cucurbitina; scientificNameAuthorship: (Rossi, 1792); **Location:** country: Italy; countryCode: IT; stateProvince: Roma; locality: Riserva Naturale di Monte Mario; decimalLatitude: 41.9386215; decimalLongitude: 12.4546223; geodeticDatum: WGS84; coordinatePrecision: 0.0002; **Identification:** identifiedBy: L. Fortini; **Event:** eventDate: 2022-07-24; **Record Level:** collectionCode: UR3
- **Type status:** Other material. **Occurrence:** catalogNumber: A0081; recordedBy: L. Fortini; individualCount: 1; sex: female; lifeStage: adult; occurrenceID: EC2DC832-72BF-5423-8C36-1092338C903D; **Taxon:** scientificName: Ceratina (Ceratina) cucurbitina (Rossi, 1792); order: Hymenoptera; family: Apidae; genus: Ceratina; subgenus: Ceratina; specificEpithet: cucurbitina; scientificNameAuthorship: (Rossi, 1792); **Location:** country: Italy; countryCode: IT; stateProvince: Roma; locality: Riserva Naturale di Monte Mario; decimalLatitude: 41.9386215; decimalLongitude: 12.4546223; geodeticDatum: WGS84; coordinatePrecision: 0.0002; **Identification:** identifiedBy: L. Fortini; **Event:** eventDate: 2022-08-23; **Record Level:** collectionCode: UR3
- **Type status:** Other material. **Occurrence:** catalogNumber: A0073, A0092; recordedBy: L. Fortini; individualCount: 2; sex: females; lifeStage: adult; occurrenceID: 28309774-5DE9-50C9-A641-DFE82613CA0A; **Taxon:** scientificName: Ceratina (Ceratina) cucurbitina (Rossi, 1792); order: Hymenoptera; family: Apidae; genus: Ceratina; subgenus: Ceratina; specificEpithet: cucurbitina; scientificNameAuthorship: (Rossi, 1792); **Location:** country: Italy; countryCode: IT; stateProvince: Roma; locality: Riserva Naturale di Monte Mario; decimalLatitude: 41.9386215; decimalLongitude: 12.4546223; geodeticDatum: WGS84; coordinatePrecision: 0.0002; **Identification:** identifiedBy: L. Fortini; **Event:** eventDate: 2022-09-21; **Record Level:** collectionCode: UR3
- **Type status:** Other material. **Occurrence:** catalogNumber: A0060, A0077, A0078; recordedBy: L. Fortini; individualCount: 3; sex: 1 male, 2 females; lifeStage: adult; occurrenceID: 7BBA90CF-2116-5DC8-B015-13ED68C822C8; **Taxon:** scientificName: Ceratina (Ceratina) cucurbitina (Rossi, 1792); order: Hymenoptera; family: Apidae; genus: Ceratina; subgenus: Ceratina; specificEpithet: cucurbitina; scientificNameAuthorship: (Rossi, 1792); **Location:** country: Italy; countryCode: IT; stateProvince: Roma; locality: Riserva Naturale Tenuta dei Massimi 2; decimalLatitude: 41.8316516; decimalLongitude: 12.3999927; geodeticDatum: WGS84; coordinatePrecision: 0.0002; **Identification:** identifiedBy: L. Fortini; **Event:** eventDate: 2022-05-04; **Record Level:** collectionCode: UR3
- **Type status:** Other material. **Occurrence:** catalogNumber: A0062, A0066, A0085; recordedBy: L. Fortini; individualCount: 3; sex: females; lifeStage: adult; occurrenceID: 10FDFE2A-529C-5BAB-87EE-5BA6A8AA2732; **Taxon:** scientificName: Ceratina (Ceratina) cucurbitina (Rossi, 1792); order: Hymenoptera; family: Apidae; genus: Ceratina; subgenus: Ceratina; specificEpithet: cucurbitina; scientificNameAuthorship: (Rossi, 1792); **Location:** country: Italy; countryCode: IT; stateProvince: Roma; locality: Riserva Naturale Tenuta dei Massimi 2; decimalLatitude: 41.8316516; decimalLongitude: 12.3999927; geodeticDatum: WGS84; coordinatePrecision: 0.0002; **Identification:** identifiedBy: L. Fortini; **Event:** eventDate: 2022-06-01; **Record Level:** collectionCode: UR3
- **Type status:** Other material. **Occurrence:** catalogNumber: A0080, A0084; recordedBy: L. Fortini; individualCount: 2; sex: females; lifeStage: adult; occurrenceID: 409CACCB-E6A2-5525-ADFA-3D3DD992C44B; **Taxon:** scientificName: Ceratina (Ceratina) cucurbitina (Rossi, 1792); order: Hymenoptera; family: Apidae; genus: Ceratina; subgenus: Ceratina; specificEpithet: cucurbitina; scientificNameAuthorship: (Rossi, 1792); **Location:** country: Italy; countryCode: IT; stateProvince: Roma; locality: Riserva Naturale Valle dell'Aniene 2; decimalLatitude: 41.928752; decimalLongitude: 12.5562962; geodeticDatum: WGS84; coordinatePrecision: 0.0002; **Identification:** identifiedBy: L. Fortini; **Event:** eventDate: 2022-06-05; **Record Level:** collectionCode: UR3
- **Type status:** Other material. **Occurrence:** catalogNumber: A0047; recordedBy: L. Fortini; individualCount: 1; sex: male; lifeStage: adult; occurrenceID: C4842BCA-A1CB-5D49-A335-27474DAFD3D6; **Taxon:** scientificName: Ceratina (Ceratina) cucurbitina (Rossi, 1792); order: Hymenoptera; family: Apidae; genus: Ceratina; subgenus: Ceratina; specificEpithet: cucurbitina; scientificNameAuthorship: (Rossi, 1792); **Location:** country: Italy; countryCode: IT; stateProvince: Roma; locality: Riserva Naturale Valle dei Casali 1; decimalLatitude: 41.8710627; decimalLongitude: 12.4336809; geodeticDatum: WGS84; coordinatePrecision: 0.0002; **Identification:** identifiedBy: L. Fortini; **Event:** eventDate: 2022-05-14; **Record Level:** collectionCode: UR3
- **Type status:** Other material. **Occurrence:** catalogNumber: A0071; recordedBy: L. Fortini; individualCount: 1; sex: female; lifeStage: adult; occurrenceID: 3119B129-8A30-5DCE-B628-D4A180D4A2E2; **Taxon:** scientificName: Ceratina (Ceratina) cucurbitina (Rossi, 1792); order: Hymenoptera; family: Apidae; genus: Ceratina; subgenus: Ceratina; specificEpithet: cucurbitina; scientificNameAuthorship: (Rossi, 1792); **Location:** country: Italy; countryCode: IT; stateProvince: Roma; locality: Riserva Naturale Valle dei Casali 2; decimalLatitude: 41.8596887; decimalLongitude: 12.4355075; geodeticDatum: WGS84; coordinatePrecision: 0.0002; **Identification:** identifiedBy: L. Fortini; **Event:** eventDate: 2022-07-13; **Record Level:** collectionCode: UR3
- **Type status:** Other material. **Occurrence:** catalogNumber: A2096; recordedBy: L. Fortini; individualCount: 1; sex: female; lifeStage: adult; occurrenceID: 4AECBE07-40F2-549F-8CEA-F614B6A3D559; **Taxon:** scientificName: Ceratina (Ceratina) cucurbitina (Rossi, 1792); order: Hymenoptera; family: Apidae; genus: Ceratina; subgenus: Ceratina; specificEpithet: cucurbitina; scientificNameAuthorship: (Rossi, 1792); **Location:** country: Italy; countryCode: IT; stateProvince: Roma; locality: Riserva Naturale Valle dell'Aniene 1; decimalLatitude: 41.9345179; decimalLongitude: 12.5453096; geodeticDatum: WGS84; coordinatePrecision: 0.0002; **Identification:** identifiedBy: L. Fortini; **Event:** eventDate: 2022-08-03; **Record Level:** collectionCode: UR3

#### Ceratina cyanea

(Kirby, 1802)

B2533808-2E8D-505A-9221-6BD35E342DAA

##### Materials

- **Type status:** Other material. **Occurrence:** catalogNumber: A0112; recordedBy: L. Fortini; individualCount: 1; sex: female; lifeStage: adult; occurrenceID: 0E075004-32A0-5E39-B5B6-5E6B5DF00E35; **Taxon:** scientificName: Ceratina (Euceratina) cyanea (Kirby, 1802); order: Hymenoptera; family: Apidae; genus: Ceratina; subgenus: Euceratina; specificEpithet: cyanea; scientificNameAuthorship: (Kirby, 1802); **Location:** country: Italy; countryCode: IT; stateProvince: Roma; locality: Riserva Naturale dell'Acquafredda; decimalLatitude: 41.8928408; decimalLongitude: 12.39932; geodeticDatum: WGS84; coordinatePrecision: 0.0002; **Identification:** identifiedBy: L. Fortini; **Event:** eventDate: 2022-04-13; **Record Level:** collectionCode: UR3
- **Type status:** Other material. **Occurrence:** catalogNumber: A0103; recordedBy: L. Fortini; individualCount: 1; sex: female; lifeStage: adult; occurrenceID: 039F7CFC-95E1-536C-B947-EBAAB692FB77; **Taxon:** scientificName: Ceratina (Euceratina) cyanea (Kirby, 1802); order: Hymenoptera; family: Apidae; genus: Ceratina; subgenus: Euceratina; specificEpithet: cyanea; scientificNameAuthorship: (Kirby, 1802); **Location:** country: Italy; countryCode: IT; stateProvince: Roma; locality: Riserva Regionale dell'Appia Antica 2; decimalLatitude: 41.8402564; decimalLongitude: 12.532773; geodeticDatum: WGS84; coordinatePrecision: 0.0002; **Identification:** identifiedBy: L. Fortini; **Event:** eventDate: 2022-10-01; **Record Level:** collectionCode: UR3
- **Type status:** Other material. **Occurrence:** catalogNumber: A0107; recordedBy: L. Fortini; individualCount: 1; sex: female; lifeStage: adult; occurrenceID: 6C5E08BD-2B4A-5478-837A-248DC6AA5925; **Taxon:** scientificName: Ceratina (Euceratina) cyanea (Kirby, 1802); order: Hymenoptera; family: Apidae; genus: Ceratina; subgenus: Euceratina; specificEpithet: cyanea; scientificNameAuthorship: (Kirby, 1802); **Location:** country: Italy; countryCode: IT; stateProvince: Roma; locality: Riserva Regionale dell'Appia Antica 2; decimalLatitude: 41.8402564; decimalLongitude: 12.532773; geodeticDatum: WGS84; coordinatePrecision: 0.0002; **Identification:** identifiedBy: L. Fortini; **Event:** eventDate: 2022-07-07; **Record Level:** collectionCode: UR3
- **Type status:** Other material. **Occurrence:** catalogNumber: A0109; recordedBy: L. Fortini; individualCount: 1; sex: female; lifeStage: adult; occurrenceID: E33AED88-C397-59E5-928E-5249021F3F4A; **Taxon:** scientificName: Ceratina (Euceratina) cyanea (Kirby, 1802); order: Hymenoptera; family: Apidae; genus: Ceratina; subgenus: Euceratina; specificEpithet: cyanea; scientificNameAuthorship: (Kirby, 1802); **Location:** country: Italy; countryCode: IT; stateProvince: Roma; locality: Riserva Naturale dell'Insugherata 2; decimalLatitude: 41.9599247; decimalLongitude: 12.433852; geodeticDatum: WGS84; coordinatePrecision: 0.0002; **Identification:** identifiedBy: L. Fortini; **Event:** eventDate: 2022-05-27; **Record Level:** collectionCode: UR3
- **Type status:** Other material. **Occurrence:** catalogNumber: A0094; recordedBy: L. Fortini; individualCount: 1; sex: male; lifeStage: adult; occurrenceID: 903FBB0E-C932-5C2D-B6A6-47EB367D6787; **Taxon:** scientificName: Ceratina (Euceratina) cyanea (Kirby, 1802); order: Hymenoptera; family: Apidae; genus: Ceratina; subgenus: Euceratina; specificEpithet: cyanea; scientificNameAuthorship: (Kirby, 1802); **Location:** country: Italy; countryCode: IT; stateProvince: Roma; locality: Riserva Naturale Laurentino-Acqua Acetosa; decimalLatitude: 41.8079275; decimalLongitude: 12.4685548; geodeticDatum: WGS84; coordinatePrecision: 0.0002; **Identification:** identifiedBy: L. Fortini; **Event:** eventDate: 2022-04-12; **Record Level:** collectionCode: UR3
- **Type status:** Other material. **Occurrence:** catalogNumber: A0097, A0111; recordedBy: L. Fortini; individualCount: 2; sex: females; lifeStage: adult; occurrenceID: 39FD01AC-6950-5AE7-808A-114E42777DCF; **Taxon:** scientificName: Ceratina (Euceratina) cyanea (Kirby, 1802); order: Hymenoptera; family: Apidae; genus: Ceratina; subgenus: Euceratina; specificEpithet: cyanea; scientificNameAuthorship: (Kirby, 1802); **Location:** country: Italy; countryCode: IT; stateProvince: Roma; locality: Riserva Naturale Laurentino-Acqua Acetosa; decimalLatitude: 41.8079275; decimalLongitude: 12.4685548; geodeticDatum: WGS84; coordinatePrecision: 0.0002; **Identification:** identifiedBy: L. Fortini; **Event:** eventDate: 2022-05-12; **Record Level:** collectionCode: UR3
- **Type status:** Other material. **Occurrence:** catalogNumber: A0093, A0105; recordedBy: L. Fortini; individualCount: 2; sex: 1 male, 1 female; lifeStage: adult; occurrenceID: CD2DCECA-C350-5765-ADB8-081B4584BE7A; **Taxon:** scientificName: Ceratina (Euceratina) cyanea (Kirby, 1802); order: Hymenoptera; family: Apidae; genus: Ceratina; subgenus: Euceratina; specificEpithet: cyanea; scientificNameAuthorship: (Kirby, 1802); **Location:** country: Italy; countryCode: IT; stateProvince: Roma; locality: Riserva Naturale Laurentino-Acqua Acetosa; decimalLatitude: 41.8079275; decimalLongitude: 12.4685548; geodeticDatum: WGS84; coordinatePrecision: 0.0002; **Identification:** identifiedBy: L. Fortini; **Event:** eventDate: 2022-08-21; **Record Level:** collectionCode: UR3
- **Type status:** Other material. **Occurrence:** catalogNumber: A0102, A0104; recordedBy: L. Fortini; individualCount: 2; sex: females; lifeStage: adult; occurrenceID: 322FF2DC-5386-5DD9-850B-CF07B90483C4; **Taxon:** scientificName: Ceratina (Euceratina) cyanea (Kirby, 1802); order: Hymenoptera; family: Apidae; genus: Ceratina; subgenus: Euceratina; specificEpithet: cyanea; scientificNameAuthorship: (Kirby, 1802); **Location:** country: Italy; countryCode: IT; stateProvince: Roma; locality: Riserva Naturale Laurentino-Acqua Acetosa; decimalLatitude: 41.8079275; decimalLongitude: 12.4685548; geodeticDatum: WGS84; coordinatePrecision: 0.0002; **Identification:** identifiedBy: L. Fortini; **Event:** eventDate: 2022-09-14; **Record Level:** collectionCode: UR3
- **Type status:** Other material. **Occurrence:** catalogNumber: A0110, A0113, A0114; recordedBy: L. Fortini; individualCount: 3; sex: females; lifeStage: adult; occurrenceID: F8F9F0A2-CD1E-5967-9D04-AB2C183A4764; **Taxon:** scientificName: Ceratina (Euceratina) cyanea (Kirby, 1802); order: Hymenoptera; family: Apidae; genus: Ceratina; subgenus: Euceratina; specificEpithet: cyanea; scientificNameAuthorship: (Kirby, 1802); **Location:** country: Italy; countryCode: IT; stateProvince: Roma; locality: Riserva Naturale di Monte Mario; decimalLatitude: 41.9386215; decimalLongitude: 12.4546223; geodeticDatum: WGS84; coordinatePrecision: 0.0002; **Identification:** identifiedBy: L. Fortini; **Event:** eventDate: 2022-05-20; **Record Level:** collectionCode: UR3
- **Type status:** Other material. **Occurrence:** catalogNumber: A0096; recordedBy: L. Fortini; individualCount: 1; sex: female; lifeStage: adult; occurrenceID: FA4D7883-8665-595A-8CB4-C826E69705DD; **Taxon:** scientificName: Ceratina (Euceratina) cyanea (Kirby, 1802); order: Hymenoptera; family: Apidae; genus: Ceratina; subgenus: Euceratina; specificEpithet: cyanea; scientificNameAuthorship: (Kirby, 1802); **Location:** country: Italy; countryCode: IT; stateProvince: Roma; locality: Riserva Naturale di Monte Mario; decimalLatitude: 41.9386215; decimalLongitude: 12.4546223; geodeticDatum: WGS84; coordinatePrecision: 0.0002; **Identification:** identifiedBy: L. Fortini; **Event:** eventDate: 2022-06-19; **Record Level:** collectionCode: UR3
- **Type status:** Other material. **Occurrence:** catalogNumber: A0099, A0106; recordedBy: L. Fortini; individualCount: 2; sex: female; lifeStage: adult; occurrenceID: 094547CE-35E0-5E01-85F9-17DFD561C2C4; **Taxon:** scientificName: Ceratina (Euceratina) cyanea (Kirby, 1802); order: Hymenoptera; family: Apidae; genus: Ceratina; subgenus: Euceratina; specificEpithet: cyanea; scientificNameAuthorship: (Kirby, 1802); **Location:** country: Italy; countryCode: IT; stateProvince: Roma; locality: Riserva Naturale di Monte Mario; decimalLatitude: 41.9386215; decimalLongitude: 12.4546223; geodeticDatum: WGS84; coordinatePrecision: 0.0002; **Identification:** identifiedBy: L. Fortini; **Event:** eventDate: 2022-09-21; **Record Level:** collectionCode: UR3
- **Type status:** Other material. **Occurrence:** catalogNumber: A0095; recordedBy: L. Fortini; individualCount: 1; sex: female; lifeStage: adult; occurrenceID: C358ECA9-2A89-5ABB-9391-4FBFCABF9D81; **Taxon:** scientificName: Ceratina (Euceratina) cyanea (Kirby, 1802); order: Hymenoptera; family: Apidae; genus: Ceratina; subgenus: Euceratina; specificEpithet: cyanea; scientificNameAuthorship: (Kirby, 1802); **Location:** country: Italy; countryCode: IT; stateProvince: Roma; locality: Riserva Naturale Tenuta dei Massimi 2; decimalLatitude: 41.8316516; decimalLongitude: 12.3999927; geodeticDatum: WGS84; coordinatePrecision: 0.0002; **Identification:** identifiedBy: L. Fortini; **Event:** eventDate: 2022-06-27; **Record Level:** collectionCode: UR3
- **Type status:** Other material. **Occurrence:** catalogNumber: A0100; recordedBy: L. Fortini; individualCount: 1; sex: female; lifeStage: adult; occurrenceID: 0ED9F8F8-B475-537B-806C-53B4E02A8996; **Taxon:** scientificName: Ceratina (Euceratina) cyanea (Kirby, 1802); order: Hymenoptera; family: Apidae; genus: Ceratina; subgenus: Euceratina; specificEpithet: cyanea; scientificNameAuthorship: (Kirby, 1802); **Location:** country: Italy; countryCode: IT; stateProvince: Roma; locality: Riserva Naturale Valle dell'Aniene 2; decimalLatitude: 41.928752; decimalLongitude: 12.5562962; geodeticDatum: WGS84; coordinatePrecision: 0.0002; **Identification:** identifiedBy: L. Fortini; **Event:** eventDate: 2022-07-01; **Record Level:** collectionCode: UR3
- **Type status:** Other material. **Occurrence:** catalogNumber: A0098; recordedBy: L. Fortini; individualCount: 1; sex: female; lifeStage: adult; occurrenceID: 678C7EF9-4FAB-5444-AE05-9826D3F8EB1D; **Taxon:** scientificName: Ceratina (Euceratina) cyanea (Kirby, 1802); order: Hymenoptera; family: Apidae; genus: Ceratina; subgenus: Euceratina; specificEpithet: cyanea; scientificNameAuthorship: (Kirby, 1802); **Location:** country: Italy; countryCode: IT; stateProvince: Roma; locality: Riserva Naturale Valle dei Casali 1; decimalLatitude: 41.8710627; decimalLongitude: 12.4336809; geodeticDatum: WGS84; coordinatePrecision: 0.0002; **Identification:** identifiedBy: L. Fortini; **Event:** eventDate: 2022-06-18; **Record Level:** collectionCode: UR3
- **Type status:** Other material. **Occurrence:** catalogNumber: A0101, A0108; recordedBy: L. Fortini; individualCount: 2; sex: females; lifeStage: adult; occurrenceID: 33577B08-36EE-5BB2-AF6C-290CA7DBB4AC; **Taxon:** scientificName: Ceratina (Euceratina) cyanea (Kirby, 1802); order: Hymenoptera; family: Apidae; genus: Ceratina; subgenus: Euceratina; specificEpithet: cyanea; scientificNameAuthorship: (Kirby, 1802); **Location:** country: Italy; countryCode: IT; stateProvince: Roma; locality: Riserva Naturale Valle dei Casali 1; decimalLatitude: 41.8710627; decimalLongitude: 12.4336809; geodeticDatum: WGS84; coordinatePrecision: 0.0002; **Identification:** identifiedBy: L. Fortini; **Event:** eventDate: 2022-09-18; **Record Level:** collectionCode: UR3
- **Type status:** Other material. **Occurrence:** catalogNumber: A2030; recordedBy: L. Fortini; individualCount: 1; sex: male; lifeStage: adult; occurrenceID: C4B8FCCE-6603-5EAE-877A-3C2E1199CCD0; **Taxon:** scientificName: Ceratina (Euceratina) cyanea (Kirby, 1802); order: Hymenoptera; family: Apidae; genus: Ceratina; subgenus: Euceratina; specificEpithet: cyanea; scientificNameAuthorship: (Kirby, 1802); **Location:** country: Italy; countryCode: IT; stateProvince: Roma; locality: Riserva Naturale Valle dei Casali 1; decimalLatitude: 41.8710627; decimalLongitude: 12.4336809; geodeticDatum: WGS84; coordinatePrecision: 0.0002; **Identification:** identifiedBy: L. Fortini; **Event:** eventDate: 2022-08-19; **Record Level:** collectionCode: UR3

#### Ceratina dallatorreana

Friese, 1896

2CC84ECE-3880-53A0-B739-FC6D91061653

##### Materials

- **Type status:** Other material. **Occurrence:** catalogNumber: A0116; recordedBy: L. Fortini; individualCount: 1; sex: female; lifeStage: adult; occurrenceID: 2E4C83A4-FA34-5A9D-B40A-AFA8E746B38A; **Taxon:** scientificName: Ceratina (Euceratina) dallatorreana Friese, 1896; order: Hymenoptera; family: Apidae; genus: Ceratina; subgenus: Euceratina; specificEpithet: dallatorreana; scientificNameAuthorship: Friese, 1896; **Location:** country: Italy; countryCode: IT; stateProvince: Roma; locality: Riserva Regionale dell'Appia Antica 1; decimalLatitude: 41.8623941; decimalLongitude: 12.524863; geodeticDatum: WGS84; coordinatePrecision: 0.0002; **Identification:** identifiedBy: L. Fortini; **Event:** eventDate: 2022-05-10; **Record Level:** collectionCode: UR3

#### Ceratina dentiventris

Gerstaecker, 1869

7415D5D1-AF77-5EAC-8AC0-988AD4C64AB7

##### Materials

- **Type status:** Other material. **Occurrence:** catalogNumber: A0115; recordedBy: L. Fortini; individualCount: 1; sex: female; lifeStage: adult; occurrenceID: B0232543-8248-5E30-99CB-68FFCF903618; **Taxon:** scientificName: Ceratina (Euceratina) dentiventris Gerstäcker, 1869; order: Hymenoptera; family: Apidae; genus: Ceratina; subgenus: Euceratina; specificEpithet: dentiventris; scientificNameAuthorship: Gerstäcker, 1869; **Location:** country: Italy; countryCode: IT; stateProvince: Roma; locality: Riserva Naturale Valle dei Casali 2; decimalLatitude: 41.8596887; decimalLongitude: 12.4355075; geodeticDatum: WGS84; coordinatePrecision: 0.0002; **Identification:** identifiedBy: L. Fortini; **Event:** eventDate: 2022-06-18; **Record Level:** collectionCode: UR3

#### Ceratina gravidula

Gerstaecker, 1869

648FF869-14EC-5939-955C-BF779DC5F0E4

##### Materials

- **Type status:** Other material. **Occurrence:** catalogNumber: A0117; recordedBy: L. Fortini; individualCount: 1; sex: female; lifeStage: adult; occurrenceID: 75ED00E2-6FE3-51CB-B301-A5C91F9AC5B6; **Taxon:** scientificName: Ceratina (Euceratina) gravidula Gerstäcker, 1869; order: Hymenoptera; family: Apidae; genus: Ceratina; subgenus: Euceratina; specificEpithet: gravidula; scientificNameAuthorship: Gerstäcker, 1869; **Location:** country: Italy; countryCode: IT; stateProvince: Roma; locality: Riserva Naturale Laurentino-Acqua Acetosa; decimalLatitude: 41.8079275; decimalLongitude: 12.4685548; geodeticDatum: WGS84; coordinatePrecision: 0.0002; **Identification:** identifiedBy: L. Fortini; **Event:** eventDate: 2022-06-24; **Record Level:** collectionCode: UR3

#### Ceratina nigrolabiata

Friese, 1896

A46BE5F3-F116-522B-86BE-1C4AC5B0FAF2

##### Materials

- **Type status:** Other material. **Occurrence:** catalogNumber: A0118; recordedBy: L. Fortini; individualCount: 1; sex: male; lifeStage: adult; occurrenceID: F69F0B24-D4FC-5927-9919-CD3A6A553C29; **Taxon:** scientificName: Ceratina (Euceratina) nigrolabiata Friese, 1896; order: Hymenoptera; family: Apidae; genus: Ceratina; subgenus: Euceratina; specificEpithet: nigrolabiata; scientificNameAuthorship: Friese, 1896; **Location:** country: Italy; countryCode: IT; stateProvince: Roma; locality: Riserva Naturale dell'Insugherata 1; decimalLatitude: 41.9555045; decimalLongitude: 12.4292321; geodeticDatum: WGS84; coordinatePrecision: 0.0002; **Identification:** identifiedBy: L. Fortini; **Event:** eventDate: 2022-07-17; **Record Level:** collectionCode: UR3
- **Type status:** Other material. **Occurrence:** catalogNumber: A2080, A2081; recordedBy: L. Fortini; individualCount: 2; sex: males; lifeStage: adult; occurrenceID: 1596B331-77E2-5EAF-879B-3784A8B3B969; **Taxon:** scientificName: Ceratina (Euceratina) nigrolabiata Friese, 1896; order: Hymenoptera; family: Apidae; genus: Ceratina; subgenus: Euceratina; specificEpithet: nigrolabiata; scientificNameAuthorship: Friese, 1896; **Location:** country: Italy; countryCode: IT; stateProvince: Roma; locality: Riserva Regionale dell'Appia Antica 2; decimalLatitude: 41.8402564; decimalLongitude: 12.532773; geodeticDatum: WGS84; coordinatePrecision: 0.0002; **Identification:** identifiedBy: L. Fortini; **Event:** eventDate: 2022-08-06; **Record Level:** collectionCode: UR3

#### Epeolus cruciger

(Panzer, 1799)

79D89057-86C4-5425-97CD-C0570C2C1D21

##### Materials

- **Type status:** Other material. **Occurrence:** catalogNumber: A0185, A0187, A0188; recordedBy: L. Fortini; individualCount: 3; sex: males; lifeStage: adult; occurrenceID: 0D596766-E92A-562D-8C7F-710595353235; **Taxon:** scientificName: Epeolus (Epeolus) cruciger (Panzer,1799); order: Hymenoptera; family: Apidae; genus: Epeolus; subgenus: Epeolus; specificEpithet: cruciger; scientificNameAuthorship: (Panzer,1799); **Location:** country: Italy; countryCode: IT; stateProvince: Roma; locality: Riserva Naturale Tenuta dei Massimi 1; decimalLatitude: 41.8532859; decimalLongitude: 12.3842322; geodeticDatum: WGS84; coordinatePrecision: 0.0002; **Identification:** identifiedBy: L. Fortini; **Event:** eventDate: 2022-09-28; **Record Level:** collectionCode: UR3
- **Type status:** Other material. **Occurrence:** catalogNumber: A0186, A0189, A0190; recordedBy: L. Fortini; individualCount: 3; sex: males; lifeStage: adult; occurrenceID: C960F37E-DC13-5E0D-A35A-74148530D032; **Taxon:** scientificName: Epeolus (Epeolus) cruciger (Panzer,1799); order: Hymenoptera; family: Apidae; genus: Epeolus; subgenus: Epeolus; specificEpithet: cruciger; scientificNameAuthorship: (Panzer,1799); **Location:** country: Italy; countryCode: IT; stateProvince: Roma; locality: Riserva Naturale di Monte Mario; decimalLatitude: 41.9386215; decimalLongitude: 12.4546223; geodeticDatum: WGS84; coordinatePrecision: 0.0002; **Identification:** identifiedBy: L. Fortini; **Event:** eventDate: 2022-09-21; **Record Level:** collectionCode: UR3

#### Eucera clypeata

Erichson, 1835

884F0703-BDE0-5272-9A7C-F700878DA163

##### Materials

- **Type status:** Other material. **Occurrence:** catalogNumber: A0715; recordedBy: L. Fortini; individualCount: 1; sex: male; lifeStage: adult; occurrenceID: ABA34FD6-1489-5B76-ACD9-3A3B9A69F76A; **Taxon:** scientificName: Eucera (Hetereucera) clypeata Erichson, 1835; order: Hymenoptera; family: Apidae; genus: Eucera; subgenus: Hetereucera; specificEpithet: clypeata; scientificNameAuthorship: Erichson, 1835; **Location:** country: Italy; countryCode: IT; stateProvince: Roma; locality: Riserva Naturale Laurentino-Acqua Acetosa; decimalLatitude: 41.8079275; decimalLongitude: 12.4685548; geodeticDatum: WGS84; coordinatePrecision: 0.0002; **Identification:** identifiedBy: M. Mei; **Event:** eventDate: 2022-05-12; **Record Level:** collectionCode: UR3
- **Type status:** Other material. **Occurrence:** catalogNumber: A2006; recordedBy: L. Fortini; individualCount: 1; sex: male; lifeStage: adult; occurrenceID: 426930EF-55E1-50AB-83EB-DC06EB256670; **Taxon:** scientificName: Eucera (Hetereucera) clypeata Erichson, 1835; order: Hymenoptera; family: Apidae; genus: Eucera; subgenus: Hetereucera; specificEpithet: clypeata; scientificNameAuthorship: Erichson, 1835; **Location:** country: Italy; countryCode: IT; stateProvince: Roma; locality: Riserva Regionale dell'Appia Antica 2; decimalLatitude: 41.8402564; decimalLongitude: 12.532773; geodeticDatum: WGS84; coordinatePrecision: 0.0002; **Identification:** identifiedBy: M. Mei; **Event:** eventDate: 2022-04-25; **Record Level:** collectionCode: UR3

#### Eucera grisea

Fabricius, 1793

E824886F-0F44-5E56-9201-6F37D63EA317

##### Materials

- **Type status:** Other material. **Occurrence:** catalogNumber: A0781; recordedBy: L. Fortini; individualCount: 1; sex: female; lifeStage: adult; occurrenceID: 4F75B644-A031-5601-B411-6743ECA47A0D; **Taxon:** scientificName: Eucera (Eucera) grisea Fabricius, 1793; order: Hymenoptera; family: Apidae; genus: Eucera; subgenus: Eucera; specificEpithet: grisea; scientificNameAuthorship: Fabricius, 1793; **Location:** country: Italy; countryCode: IT; stateProvince: Roma; locality: Riserva Naturale Laurentino-Acqua Acetosa; decimalLatitude: 41.8079275; decimalLongitude: 12.4685548; geodeticDatum: WGS84; coordinatePrecision: 0.0002; **Identification:** identifiedBy: M. Mei; **Event:** eventDate: 2022-06-16; **Record Level:** collectionCode: UR3
- **Type status:** Other material. **Occurrence:** catalogNumber: A0711, A0712, A0790, A0791; recordedBy: L. Fortini; individualCount: 4; sex: 2 males, 2 females; lifeStage: adult; occurrenceID: 3B11C93E-D7A7-5CF5-AD3D-D31173908595; **Taxon:** scientificName: Eucera (Eucera) grisea Fabricius, 1793; order: Hymenoptera; family: Apidae; genus: Eucera; subgenus: Eucera; specificEpithet: grisea; scientificNameAuthorship: Fabricius, 1793; **Location:** country: Italy; countryCode: IT; stateProvince: Roma; locality: Riserva Naturale Laurentino-Acqua Acetosa; decimalLatitude: 41.8079275; decimalLongitude: 12.4685548; geodeticDatum: WGS84; coordinatePrecision: 0.0002; **Identification:** identifiedBy: M. Mei; **Event:** eventDate: 2022-05-12; **Record Level:** collectionCode: UR3
- **Type status:** Other material. **Occurrence:** catalogNumber: A0780; recordedBy: L. Fortini; individualCount: 1; sex: female; lifeStage: adult; occurrenceID: 1FEE9332-E2D5-5507-9193-C18084127E3D; **Taxon:** scientificName: Eucera (Eucera) grisea Fabricius, 1793; order: Hymenoptera; family: Apidae; genus: Eucera; subgenus: Hetereucera; specificEpithet: grisea; scientificNameAuthorship: Fabricius, 1793; **Location:** country: Italy; countryCode: IT; stateProvince: Roma; locality: Riserva Naturale Tenuta dei Massimi 1; decimalLatitude: 41.8532859; decimalLongitude: 12.3842322; geodeticDatum: WGS84; coordinatePrecision: 0.0002; **Identification:** identifiedBy: M. Mei; **Event:** eventDate: 2022-06-27; **Record Level:** collectionCode: UR3
- **Type status:** Other material. **Occurrence:** catalogNumber: A0782, A0783, A0785, A0786, A0787, A0792, A0793, A0798, A0799, A0800; recordedBy: L. Fortini; individualCount: 10; sex: females; lifeStage: adult; occurrenceID: A9EA6FE0-5119-5BA0-8AAA-34A77A66152F; **Taxon:** scientificName: Eucera (Eucera) grisea Fabricius, 1793; order: Hymenoptera; family: Apidae; genus: Eucera; subgenus: Eucera; specificEpithet: grisea; scientificNameAuthorship: Fabricius, 1793; **Location:** country: Italy; countryCode: IT; stateProvince: Roma; locality: Riserva Naturale Tenuta dei Massimi 1; decimalLatitude: 41.8532859; decimalLongitude: 12.3842322; geodeticDatum: WGS84; coordinatePrecision: 0.0002; **Identification:** identifiedBy: M. Mei; **Event:** eventDate: 2022-06-01; **Record Level:** collectionCode: UR3
- **Type status:** Other material. **Occurrence:** catalogNumber: A0703, A0704, A0705, A0706, A0707, A0713, A0767; recordedBy: L. Fortini; individualCount: 7; sex: 6 males, 1 female; lifeStage: adult; occurrenceID: C20D837B-7713-5924-A3AD-99267E527AC0; **Taxon:** scientificName: Eucera (Eucera) grisea Fabricius, 1793; order: Hymenoptera; family: Apidae; genus: Eucera; subgenus: Eucera; specificEpithet: grisea; scientificNameAuthorship: Fabricius, 1793; **Location:** country: Italy; countryCode: IT; stateProvince: Roma; locality: Riserva Naturale Tenuta dei Massimi 2; decimalLatitude: 41.8316516; decimalLongitude: 12.3999927; geodeticDatum: WGS84; coordinatePrecision: 0.0002; **Identification:** identifiedBy: M. Mei; **Event:** eventDate: 2022-05-04; **Record Level:** collectionCode: UR3
- **Type status:** Other material. **Occurrence:** catalogNumber: A0784, A0796; recordedBy: L. Fortini; individualCount: 2; sex: females; lifeStage: adult; occurrenceID: 6D3F0BE1-C0B4-5CE6-9FE2-2450AF46C912; **Taxon:** scientificName: Eucera (Eucera) grisea Fabricius, 1793; order: Hymenoptera; family: Apidae; genus: Eucera; subgenus: Eucera; specificEpithet: grisea; scientificNameAuthorship: Fabricius, 1793; **Location:** country: Italy; countryCode: IT; stateProvince: Roma; locality: Riserva Naturale Tenuta dei Massimi 2; decimalLatitude: 41.8316516; decimalLongitude: 12.3999927; geodeticDatum: WGS84; coordinatePrecision: 0.0002; **Identification:** identifiedBy: M. Mei; **Event:** eventDate: 2022-06-01; **Record Level:** collectionCode: UR3
- **Type status:** Other material. **Occurrence:** catalogNumber: A0789; recordedBy: L. Fortini; individualCount: 1; sex: female; lifeStage: adult; occurrenceID: 4CD44DFC-45FD-57B1-BA80-EA3D9B856F92; **Taxon:** scientificName: Eucera (Eucera) grisea Fabricius, 1793; order: Hymenoptera; family: Apidae; genus: Eucera; subgenus: Eucera; specificEpithet: grisea; scientificNameAuthorship: Fabricius, 1793; **Location:** country: Italy; countryCode: IT; stateProvince: Roma; locality: Riserva Naturale Valle dei Casali 1; decimalLatitude: 41.8710627; decimalLongitude: 12.4336809; geodeticDatum: WGS84; coordinatePrecision: 0.0002; **Identification:** identifiedBy: M. Mei; **Event:** eventDate: 2022-05-14; **Record Level:** collectionCode: UR3
- **Type status:** Other material. **Occurrence:** catalogNumber: A1997; recordedBy: L. Fortini; individualCount: 1; sex: male; lifeStage: adult; occurrenceID: 57CDF267-C1E4-5DC2-9711-224EF3BD6036; **Taxon:** scientificName: Eucera (Eucera) grisea Fabricius, 1793; order: Hymenoptera; family: Apidae; genus: Eucera; subgenus: Eucera; specificEpithet: grisea; scientificNameAuthorship: Fabricius, 1793; **Location:** country: Italy; countryCode: IT; stateProvince: Roma; locality: Riserva Regionale dell'Appia Antica 1; decimalLatitude: 41.8623941; decimalLongitude: 12.524863; geodeticDatum: WGS84; coordinatePrecision: 0.0002; **Identification:** identifiedBy: M. Mei; **Event:** eventDate: 2022-04-19; **Record Level:** collectionCode: UR3

#### Eucera interrupta

Bär, 1850

422C0E2A-5588-5757-899C-D2E7ACA110E4

##### Materials

- **Type status:** Other material. **Occurrence:** catalogNumber: A0710, A0746; recordedBy: L. Fortini; individualCount: 2; sex: males; lifeStage: adult; occurrenceID: 94C55DBB-911E-55D0-82AB-575FCF6D05C6; **Taxon:** scientificName: Eucera (Eucera) interrupta Bär, 1850; order: Hymenoptera; family: Apidae; genus: Eucera; subgenus: Eucera; specificEpithet: interrupta; scientificNameAuthorship: Bär, 1850; **Location:** country: Italy; countryCode: IT; stateProvince: Roma; locality: Riserva Naturale dell'Insugherata 2; decimalLatitude: 41.9599247; decimalLongitude: 12.433852; geodeticDatum: WGS84; coordinatePrecision: 0.0002; **Identification:** identifiedBy: M. Mei; **Event:** eventDate: 2022-05-27; **Record Level:** collectionCode: UR3

#### Eucera nigrescens

Pérez, 1879

615CBC40-92C6-5548-8CC0-F3CC87B85C42

##### Materials

- **Type status:** Other material. **Occurrence:** catalogNumber: A0724, A0726, A0727, A0733, A0736; recordedBy: L. Fortini; individualCount: 5; sex: males; lifeStage: adult; occurrenceID: 83D639D9-E877-550D-9CC6-F81D2C5081FE; **Taxon:** scientificName: Eucera (Eucera) nigrescens Pérez, 1879; order: Hymenoptera; family: Apidae; genus: Eucera; subgenus: Eucera; specificEpithet: nigrescens; scientificNameAuthorship: Pérez, 1879; **Location:** country: Italy; countryCode: IT; stateProvince: Roma; locality: Riserva Naturale dell'Acquafredda; decimalLatitude: 41.8928408; decimalLongitude: 12.39932; geodeticDatum: WGS84; coordinatePrecision: 0.0002; **Identification:** identifiedBy: M. Mei; **Event:** eventDate: 2022-04-13; **Record Level:** collectionCode: UR3
- **Type status:** Other material. **Occurrence:** catalogNumber: A0763; recordedBy: L. Fortini; individualCount: 1; sex: female; lifeStage: adult; occurrenceID: FBDFFA55-F194-5599-81E6-DB0FAFD7289D; **Taxon:** scientificName: Eucera (Eucera) nigrescens Pérez, 1879; order: Hymenoptera; family: Apidae; genus: Eucera; subgenus: Eucera; specificEpithet: nigrescens; scientificNameAuthorship: Pérez, 1879; **Location:** country: Italy; countryCode: IT; stateProvince: Roma; locality: Riserva Naturale dell'Acquafredda; decimalLatitude: 41.8928408; decimalLongitude: 12.39932; geodeticDatum: WGS84; coordinatePrecision: 0.0002; **Identification:** identifiedBy: M. Mei; **Event:** eventDate: 2022-05-15; **Record Level:** collectionCode: UR3
- **Type status:** Other material. **Occurrence:** catalogNumber: A0759, A0760; recordedBy: L. Fortini; individualCount: 2; sex: females; lifeStage: adult; occurrenceID: 3BD4B668-98DD-566D-B838-45F7B342E58A; **Taxon:** scientificName: Eucera (Eucera) nigrescens Pérez, 1879; order: Hymenoptera; family: Apidae; genus: Eucera; subgenus: Eucera; specificEpithet: nigrescens; scientificNameAuthorship: Pérez, 1879; **Location:** country: Italy; countryCode: IT; stateProvince: Roma; locality: Riserva Naturale dell'Insugherata 2; decimalLatitude: 41.9599247; decimalLongitude: 12.433852; geodeticDatum: WGS84; coordinatePrecision: 0.0002; **Identification:** identifiedBy: M. Mei; **Event:** eventDate: 2022-05-27; **Record Level:** collectionCode: UR3
- **Type status:** Other material. **Occurrence:** catalogNumber: A0717, A0719, A0721, A0722, A0728, A0729; recordedBy: L. Fortini; individualCount: 6; sex: males; lifeStage: adult; occurrenceID: A9C3F336-D1E0-51A2-86F8-B2D5668D6A7B; **Taxon:** scientificName: Eucera (Eucera) nigrescens Pérez, 1879; order: Hymenoptera; family: Apidae; genus: Eucera; subgenus: Eucera; specificEpithet: nigrescens; scientificNameAuthorship: Pérez, 1879; **Location:** country: Italy; countryCode: IT; stateProvince: Roma; locality: Riserva Naturale Laurentino-Acqua Acetosa; decimalLatitude: 41.8079275; decimalLongitude: 12.4685548; geodeticDatum: WGS84; coordinatePrecision: 0.0002; **Identification:** identifiedBy: M. Mei; **Event:** eventDate: 2022-04-12; **Record Level:** collectionCode: UR3
- **Type status:** Other material. **Occurrence:** catalogNumber: A0732, A0734, A0737; recordedBy: L. Fortini; individualCount: 3; sex: males; lifeStage: adult; occurrenceID: 8D963961-1163-5E2D-8462-8A4E9B0F3FA7; **Taxon:** scientificName: Eucera (Eucera) nigrescens Pérez, 1879; order: Hymenoptera; family: Apidae; genus: Eucera; subgenus: Eucera; specificEpithet: nigrescens; scientificNameAuthorship: Pérez, 1879; **Location:** country: Italy; countryCode: IT; stateProvince: Roma; locality: Riserva Naturale di Monte Mario; decimalLatitude: 41.9386215; decimalLongitude: 12.4546223; geodeticDatum: WGS84; coordinatePrecision: 0.0002; **Identification:** identifiedBy: M. Mei; **Event:** eventDate: 2022-04-20; **Record Level:** collectionCode: UR3
- **Type status:** Other material. **Occurrence:** catalogNumber: A0725, A0735, A0761; recordedBy: L. Fortini; individualCount: 3; sex: 2 males, 1 female; lifeStage: adult; occurrenceID: EAFC054D-CA43-595F-B8A8-99AAB5DB65D3; **Taxon:** scientificName: Eucera (Eucera) nigrescens Pérez, 1879; order: Hymenoptera; family: Apidae; genus: Eucera; subgenus: Eucera; specificEpithet: nigrescens; scientificNameAuthorship: Pérez, 1879; **Location:** country: Italy; countryCode: IT; stateProvince: Roma; locality: Riserva Naturale Tenuta dei Massimi 1; decimalLatitude: 41.8532859; decimalLongitude: 12.3842322; geodeticDatum: WGS84; coordinatePrecision: 0.0002; **Identification:** identifiedBy: M. Mei; **Event:** eventDate: 2022-04-23; **Record Level:** collectionCode: UR3
- **Type status:** Other material. **Occurrence:** catalogNumber: A0723, A0730; recordedBy: L. Fortini; individualCount: 2; sex: males; lifeStage: adult; occurrenceID: FDB9129D-C6FD-56B8-A849-7629B94DB591; **Taxon:** scientificName: Eucera (Eucera) nigrescens Pérez, 1879; order: Hymenoptera; family: Apidae; genus: Eucera; subgenus: Eucera; specificEpithet: nigrescens; scientificNameAuthorship: Pérez, 1879; **Location:** country: Italy; countryCode: IT; stateProvince: Roma; locality: Riserva Naturale Tenuta dei Massimi 2; decimalLatitude: 41.8316516; decimalLongitude: 12.3999927; geodeticDatum: WGS84; coordinatePrecision: 0.0002; **Identification:** identifiedBy: M. Mei; **Event:** eventDate: 2022-05-04; **Record Level:** collectionCode: UR3
- **Type status:** Other material. **Occurrence:** catalogNumber: A0716, A0720; recordedBy: L. Fortini; individualCount: 2; sex: males; lifeStage: adult; occurrenceID: 4E7CDF5F-3B6F-5F54-BF89-325F2C32E05D; **Taxon:** scientificName: Eucera (Eucera) nigrescens Pérez, 1879; order: Hymenoptera; family: Apidae; genus: Eucera; subgenus: Eucera; specificEpithet: nigrescens; scientificNameAuthorship: Pérez, 1879; **Location:** country: Italy; countryCode: IT; stateProvince: Roma; locality: Riserva Naturale Valle dei Casali 1; decimalLatitude: 41.8710627; decimalLongitude: 12.4336809; geodeticDatum: WGS84; coordinatePrecision: 0.0002; **Identification:** identifiedBy: M. Mei; **Event:** eventDate: 2022-04-07; **Record Level:** collectionCode: UR3
- **Type status:** Other material. **Occurrence:** catalogNumber: A0718, A0731, A0738; recordedBy: L. Fortini; individualCount: 3; sex: males; lifeStage: adult; occurrenceID: 0867E134-F3F7-5DBC-AFD1-799BD9575C41; **Taxon:** scientificName: Eucera (Eucera) nigrescens Pérez, 1879; order: Hymenoptera; family: Apidae; genus: Eucera; subgenus: Eucera; specificEpithet: nigrescens; scientificNameAuthorship: Pérez, 1879; **Location:** country: Italy; countryCode: IT; stateProvince: Roma; locality: Riserva Naturale Valle dei Casali 2; decimalLatitude: 41.8596887; decimalLongitude: 12.4355075; geodeticDatum: WGS84; coordinatePrecision: 0.0002; **Identification:** identifiedBy: M. Mei; **Event:** eventDate: 2022-04-10; **Record Level:** collectionCode: UR3
- **Type status:** Other material. **Occurrence:** catalogNumber: A2001; recordedBy: L. Fortini; individualCount: 1; sex: male; lifeStage: adult; occurrenceID: 8C04C882-4DAE-5E76-8A59-0D56C6893880; **Taxon:** scientificName: Eucera (Eucera) nigrescens Pérez, 1879; order: Hymenoptera; family: Apidae; genus: Eucera; subgenus: Eucera; specificEpithet: nigrescens; scientificNameAuthorship: Pérez, 1879; **Location:** country: Italy; countryCode: IT; stateProvince: Roma; locality: Riserva Regionale dell'Appia Antica 1; decimalLatitude: 41.8623941; decimalLongitude: 12.524863; geodeticDatum: WGS84; coordinatePrecision: 0.0002; **Identification:** identifiedBy: M. Mei; **Event:** eventDate: 2022-04-19; **Record Level:** collectionCode: UR3
- **Type status:** Other material. **Occurrence:** catalogNumber: A2005, A2008, A2021; recordedBy: L. Fortini; individualCount: 3; sex: males; lifeStage: adult; occurrenceID: D5C46BD1-EE46-59BB-BD71-947A1DA4E890; **Taxon:** scientificName: Eucera (Eucera) nigrescens Pérez, 1879; order: Hymenoptera; family: Apidae; genus: Eucera; subgenus: Eucera; specificEpithet: nigrescens; scientificNameAuthorship: Pérez, 1879; **Location:** country: Italy; countryCode: IT; stateProvince: Roma; locality: Riserva Regionale dell'Appia Antica 2; decimalLatitude: 41.8402564; decimalLongitude: 12.532773; geodeticDatum: WGS84; coordinatePrecision: 0.0002; **Identification:** identifiedBy: M. Mei; **Event:** eventDate: 2022-04-25; **Record Level:** collectionCode: UR3

#### Eucera nigrifacies

Lepeletier, 1841

B1A3613E-CF84-5A62-AA72-BCF6F9E56C6E

##### Materials

- **Type status:** Other material. **Occurrence:** catalogNumber: A0743; recordedBy: L. Fortini; individualCount: 1; sex: male; lifeStage: adult; occurrenceID: CEEF97CF-996A-543C-A319-AA0D391F33B0; **Taxon:** scientificName: Eucera (Pteneucera) nigrifacies Lepeletier, 1841; order: Hymenoptera; family: Apidae; genus: Eucera; subgenus: Pteneucera; specificEpithet: nigrifacies; scientificNameAuthorship: Lepeletier, 1841; **Location:** country: Italy; countryCode: IT; stateProvince: Roma; locality: Riserva Naturale dell'Acquafredda; decimalLatitude: 41.8928408; decimalLongitude: 12.39932; geodeticDatum: WGS84; coordinatePrecision: 0.0002; **Identification:** identifiedBy: M. Mei; **Event:** eventDate: 2022-06-10; **Record Level:** collectionCode: UR3
- **Type status:** Other material. **Occurrence:** catalogNumber: A0803; recordedBy: L. Fortini; individualCount: 1; sex: female; lifeStage: adult; occurrenceID: 9168F731-6792-5908-9CFF-987A756D19AD; **Taxon:** scientificName: Eucera (Pteneucera) nigrifacies Lepeletier, 1841; order: Hymenoptera; family: Apidae; genus: Eucera; subgenus: Pteneucera; specificEpithet: nigrifacies; scientificNameAuthorship: Lepeletier, 1841; **Location:** country: Italy; countryCode: IT; stateProvince: Roma; locality: Riserva Naturale dell'Acquafredda; decimalLatitude: 41.8928408; decimalLongitude: 12.39932; geodeticDatum: WGS84; coordinatePrecision: 0.0002; **Identification:** identifiedBy: M. Mei; **Event:** eventDate: 2022-07-12; **Record Level:** collectionCode: UR3
- **Type status:** Other material. **Occurrence:** catalogNumber: A0772; recordedBy: L. Fortini; individualCount: 1; sex: female; lifeStage: adult; occurrenceID: AB7F46DF-3F00-5F72-9E29-1D35BE49D2BE; **Taxon:** scientificName: Eucera (Pteneucera) nigrifacies Lepeletier, 1841; order: Hymenoptera; family: Apidae; genus: Eucera; subgenus: Pteneucera; specificEpithet: nigrifacies; scientificNameAuthorship: Lepeletier, 1841; **Location:** country: Italy; countryCode: IT; stateProvince: Roma; locality: Riserva Regionale dell'Appia Antica 2; decimalLatitude: 41.8402564; decimalLongitude: 12.532773; geodeticDatum: WGS84; coordinatePrecision: 0.0002; **Identification:** identifiedBy: M. Mei; **Event:** eventDate: 2022-05-24; **Record Level:** collectionCode: UR3
- **Type status:** Other material. **Occurrence:** catalogNumber: A0804, A0805; recordedBy: L. Fortini; individualCount: 2; sex: females; lifeStage: adult; occurrenceID: D127AD24-D5E4-5E26-AFEC-F8A2A0F7961A; **Taxon:** scientificName: Eucera (Pteneucera) nigrifacies Lepeletier, 1841; order: Hymenoptera; family: Apidae; genus: Eucera; subgenus: Pteneucera; specificEpithet: nigrifacies; scientificNameAuthorship: Lepeletier, 1841; **Location:** country: Italy; countryCode: IT; stateProvince: Roma; locality: Riserva Regionale dell'Appia Antica 2; decimalLatitude: 41.8402564; decimalLongitude: 12.532773; geodeticDatum: WGS84; coordinatePrecision: 0.0002; **Identification:** identifiedBy: M. Mei; **Event:** eventDate: 2022-07-07; **Record Level:** collectionCode: UR3
- **Type status:** Other material. **Occurrence:** catalogNumber: A0770; recordedBy: L. Fortini; individualCount: 1; sex: female; lifeStage: adult; occurrenceID: C4D5A793-C8A0-588A-8D32-DA5C93CE6C02; **Taxon:** scientificName: Eucera (Pteneucera) nigrifacies Lepeletier, 1841; order: Hymenoptera; family: Apidae; genus: Eucera; subgenus: Pteneucera; specificEpithet: nigrifacies; scientificNameAuthorship: Lepeletier, 1841; **Location:** country: Italy; countryCode: IT; stateProvince: Roma; locality: Riserva Naturale dell'Insugherata 3; decimalLatitude: 41.9644829; decimalLongitude: 12.436101; geodeticDatum: WGS84; coordinatePrecision: 0.0002; **Identification:** identifiedBy: M. Mei; **Event:** eventDate: 2022-06-24; **Record Level:** collectionCode: UR3
- **Type status:** Other material. **Occurrence:** catalogNumber: A0708; recordedBy: L. Fortini; individualCount: 1; sex: male; lifeStage: adult; occurrenceID: 83A94ACE-07D7-537F-9326-B2FC1EB1FD26; **Taxon:** scientificName: Eucera (Pteneucera) nigrifacies Lepeletier, 1841; order: Hymenoptera; family: Apidae; genus: Eucera; subgenus: Pteneucera; specificEpithet: nigrifacies; scientificNameAuthorship: Lepeletier, 1841; **Location:** country: Italy; countryCode: IT; stateProvince: Roma; locality: Riserva Naturale Laurentino-Acqua Acetosa; decimalLatitude: 41.8079275; decimalLongitude: 12.4685548; geodeticDatum: WGS84; coordinatePrecision: 0.0002; **Identification:** identifiedBy: M. Mei; **Event:** eventDate: 2022-06-16; **Record Level:** collectionCode: UR3
- **Type status:** Other material. **Occurrence:** catalogNumber: A0774, A0797, A0802, A0808, A0809; recordedBy: L. Fortini; individualCount: 5; sex: females; lifeStage: adult; occurrenceID: BA89BB7D-62CB-5E75-8447-23AD06B95F13; **Taxon:** scientificName: Eucera (Pteneucera) nigrifacies Lepeletier, 1841; order: Hymenoptera; family: Apidae; genus: Eucera; subgenus: Pteneucera; specificEpithet: nigrifacies; scientificNameAuthorship: Lepeletier, 1841; **Location:** country: Italy; countryCode: IT; stateProvince: Roma; locality: Riserva Naturale Tenuta dei Massimi 1; decimalLatitude: 41.8532859; decimalLongitude: 12.3842322; geodeticDatum: WGS84; coordinatePrecision: 0.0002; **Identification:** identifiedBy: M. Mei; **Event:** eventDate: 2022-06-01; **Record Level:** collectionCode: UR3
- **Type status:** Other material. **Occurrence:** catalogNumber: A0742, A0773, A0810; recordedBy: L. Fortini; individualCount: 3; sex: 1 male, 2 females; lifeStage: adult; occurrenceID: E0A7638D-304A-5036-A366-4146456C2B0D; **Taxon:** scientificName: Eucera (Pteneucera) nigrifacies Lepeletier, 1841; order: Hymenoptera; family: Apidae; genus: Eucera; subgenus: Pteneucera; specificEpithet: nigrifacies; scientificNameAuthorship: Lepeletier, 1841; **Location:** country: Italy; countryCode: IT; stateProvince: Roma; locality: Riserva Naturale Tenuta dei Massimi 1; decimalLatitude: 41.8532859; decimalLongitude: 12.3842322; geodeticDatum: WGS84; coordinatePrecision: 0.0002; **Identification:** identifiedBy: M. Mei; **Event:** eventDate: 2022-06-27; **Record Level:** collectionCode: UR3
- **Type status:** Other material. **Occurrence:** catalogNumber: A0769; recordedBy: L. Fortini; individualCount: 1; sex: female; lifeStage: adult; occurrenceID: 7BD5103F-3278-5506-AA3F-8C76F358FF1F; **Taxon:** scientificName: Eucera (Pteneucera) nigrifacies Lepeletier, 1841; order: Hymenoptera; family: Apidae; genus: Eucera; subgenus: Pteneucera; specificEpithet: nigrifacies; scientificNameAuthorship: Lepeletier, 1841; **Location:** country: Italy; countryCode: IT; stateProvince: Roma; locality: Riserva Naturale Tenuta dei Massimi 2; decimalLatitude: 41.8316516; decimalLongitude: 12.3999927; geodeticDatum: WGS84; coordinatePrecision: 0.0002; **Identification:** identifiedBy: M. Mei; **Event:** eventDate: 2022-05-04; **Record Level:** collectionCode: UR3
- **Type status:** Other material. **Occurrence:** catalogNumber: A0709; recordedBy: L. Fortini; individualCount: 1; sex: male; lifeStage: adult; occurrenceID: 44373FA0-0356-5D4B-AA09-1DC0592F61D9; **Taxon:** scientificName: Eucera (Pteneucera) nigrifacies Lepeletier, 1841; order: Hymenoptera; family: Apidae; genus: Eucera; subgenus: Pteneucera; specificEpithet: nigrifacies; scientificNameAuthorship: Lepeletier, 1841; **Location:** country: Italy; countryCode: IT; stateProvince: Roma; locality: Riserva Naturale Valle dell'Aniene 2; decimalLatitude: 41.928752; decimalLongitude: 12.5562962; geodeticDatum: WGS84; coordinatePrecision: 0.0002; **Identification:** identifiedBy: M. Mei; **Event:** eventDate: 2022-06-05; **Record Level:** collectionCode: UR3
- **Type status:** Other material. **Occurrence:** catalogNumber: A0771, A0794, A0795; recordedBy: L. Fortini; individualCount: 3; sex: females; lifeStage: adult; occurrenceID: 8026EF1D-C8EB-5BB1-8C1B-E51B72BBCCEA; **Taxon:** scientificName: Eucera (Pteneucera) nigrifacies Lepeletier, 1841; order: Hymenoptera; family: Apidae; genus: Eucera; subgenus: Pteneucera; specificEpithet: nigrifacies; scientificNameAuthorship: Lepeletier, 1841; **Location:** country: Italy; countryCode: IT; stateProvince: Roma; locality: Riserva Naturale Valle dell'Aniene 2; decimalLatitude: 41.928752; decimalLongitude: 12.5562962; geodeticDatum: WGS84; coordinatePrecision: 0.0002; **Identification:** identifiedBy: M. Mei; **Event:** eventDate: 2022-07-01; **Record Level:** collectionCode: UR3
- **Type status:** Other material. **Occurrence:** catalogNumber: A0744, A0745; recordedBy: L. Fortini; individualCount: 2; sex: males; lifeStage: adult; occurrenceID: DBDD61F5-9E14-51F7-937E-A0C240AA07E7; **Taxon:** scientificName: Eucera (Pteneucera) nigrifacies Lepeletier, 1841; order: Hymenoptera; family: Apidae; genus: Eucera; subgenus: Pteneucera; specificEpithet: nigrifacies; scientificNameAuthorship: Lepeletier, 1841; **Location:** country: Italy; countryCode: IT; stateProvince: Roma; locality: Riserva Naturale Valle dei Casali 1; decimalLatitude: 41.8710627; decimalLongitude: 12.4336809; geodeticDatum: WGS84; coordinatePrecision: 0.0002; **Identification:** identifiedBy: M. Mei; **Event:** eventDate: 2022-06-18; **Record Level:** collectionCode: UR3
- **Type status:** Other material. **Occurrence:** catalogNumber: A0768; recordedBy: L. Fortini; individualCount: 1; sex: female; lifeStage: adult; occurrenceID: AD5B0875-10B4-51DE-A19C-CEB205B9E49B; **Taxon:** scientificName: Eucera (Pteneucera) nigrifacies Lepeletier, 1841; order: Hymenoptera; family: Apidae; genus: Eucera; subgenus: Pteneucera; specificEpithet: nigrifacies; scientificNameAuthorship: Lepeletier, 1841; **Location:** country: Italy; countryCode: IT; stateProvince: Roma; locality: Riserva Naturale Valle dei Casali 2; decimalLatitude: 41.8596887; decimalLongitude: 12.4355075; geodeticDatum: WGS84; coordinatePrecision: 0.0002; **Identification:** identifiedBy: M. Mei; **Event:** eventDate: 2022-06-18; **Record Level:** collectionCode: UR3
- **Type status:** Other material. **Occurrence:** catalogNumber: A0801; recordedBy: L. Fortini; individualCount: 1; sex: female; lifeStage: adult; occurrenceID: FA74B47C-0EC2-5C8B-8D89-66EAF6B35774; **Taxon:** scientificName: Eucera (Pteneucera) nigrifacies Lepeletier, 1841; order: Hymenoptera; family: Apidae; genus: Eucera; subgenus: Pteneucera; specificEpithet: nigrifacies; scientificNameAuthorship: Lepeletier, 1841; **Location:** country: Italy; countryCode: IT; stateProvince: Roma; locality: Riserva Naturale Valle dei Casali 2; decimalLatitude: 41.8596887; decimalLongitude: 12.4355075; geodeticDatum: WGS84; coordinatePrecision: 0.0002; **Identification:** identifiedBy: M. Mei; **Event:** eventDate: 2022-07-13; **Record Level:** collectionCode: UR3

#### Eucera numida

Lepeletier, 1841

FAED67EF-30D5-5C07-871F-F724C5999F5C

##### Materials

- **Type status:** Other material. **Occurrence:** catalogNumber: A0739; recordedBy: L. Fortini; individualCount: 1; sex: male; lifeStage: adult; occurrenceID: A2381863-DB4E-5008-948A-69E330472494; **Taxon:** scientificName: Eucera (Eucera) numida Lepeletier, 1841; order: Hymenoptera; family: Apidae; genus: Eucera; subgenus: Eucera; specificEpithet: numida; scientificNameAuthorship: Lepeletier, 1841; **Location:** country: Italy; countryCode: IT; stateProvince: Roma; locality: Riserva Naturale Valle dei Casali 2; decimalLatitude: 41.8596887; decimalLongitude: 12.4355075; geodeticDatum: WGS84; coordinatePrecision: 0.0002; **Identification:** identifiedBy: M. Mei; **Event:** eventDate: 2022-04-10; **Record Level:** collectionCode: UR3
- **Type status:** Other material. **Occurrence:** catalogNumber: A0747; recordedBy: L. Fortini; individualCount: 1; sex: male; lifeStage: adult; occurrenceID: 62598F3D-9304-5E9C-99C3-22A12C7AE12F; **Taxon:** scientificName: Eucera (Eucera) numida Lepeletier, 1841; order: Hymenoptera; family: Apidae; genus: Eucera; subgenus: Eucera; specificEpithet: numida; scientificNameAuthorship: Lepeletier, 1841; **Location:** country: Italy; countryCode: IT; stateProvince: Roma; locality: Riserva Regionale dell'Appia Antica 1; decimalLatitude: 41.8623941; decimalLongitude: 12.524863; geodeticDatum: WGS84; coordinatePrecision: 0.0002; **Identification:** identifiedBy: M. Mei; **Event:** eventDate: 2022-05-10; **Record Level:** collectionCode: UR3
- **Type status:** Other material. **Occurrence:** catalogNumber: A0811; recordedBy: L. Fortini; individualCount: 1; sex: female; lifeStage: adult; occurrenceID: 4AA4C9D1-5F97-500E-AF76-F2837230BFC7; **Taxon:** scientificName: Eucera (Eucera) numida Lepeletier, 1841; order: Hymenoptera; family: Apidae; genus: Eucera; subgenus: Eucera; specificEpithet: numida; scientificNameAuthorship: Lepeletier, 1841; **Location:** country: Italy; countryCode: IT; stateProvince: Roma; locality: Riserva Naturale dell'Insugherata 1; decimalLatitude: 41.9555045; decimalLongitude: 12.4292321; geodeticDatum: WGS84; coordinatePrecision: 0.0002; **Identification:** identifiedBy: M. Mei; **Event:** eventDate: 2022-04-15; **Record Level:** collectionCode: UR3
- **Type status:** Other material. **Occurrence:** catalogNumber: A0813; recordedBy: L. Fortini; individualCount: 1; sex: female; lifeStage: adult; occurrenceID: AFFB5BE5-8AD6-51E3-B8BD-2C449DB2A9E5; **Taxon:** scientificName: Eucera (Eucera) numida Lepeletier, 1841; order: Hymenoptera; family: Apidae; genus: Eucera; subgenus: Eucera; specificEpithet: numida; scientificNameAuthorship: Lepeletier, 1841; **Location:** country: Italy; countryCode: IT; stateProvince: Roma; locality: Riserva Naturale Tenuta dei Massimi 1; decimalLatitude: 41.8532859; decimalLongitude: 12.3842322; geodeticDatum: WGS84; coordinatePrecision: 0.0002; **Identification:** identifiedBy: M. Mei; **Event:** eventDate: 2022-04-23; **Record Level:** collectionCode: UR3

#### Eucera oraniensis

Lepeletier, 1841

DCBAB724-3582-5BF2-BBDA-89A87965C414

##### Materials

- **Type status:** Other material. **Occurrence:** catalogNumber: A0777, A0778; recordedBy: L. Fortini; individualCount: 2; sex: females; lifeStage: adult; occurrenceID: 4B6C95D5-0355-5889-BDF8-F3EFE91ABE81; **Taxon:** scientificName: Eucera (Hetereucera) oraniensis Lepeletier, 1841; order: Hymenoptera; family: Apidae; genus: Eucera; subgenus: Hetereucera; specificEpithet: oraniensis; scientificNameAuthorship: Lepeletier, 1841; **Location:** country: Italy; countryCode: IT; stateProvince: Roma; locality: Riserva Regionale dell'Appia Antica 3; decimalLatitude: 41.8298456; decimalLongitude: 12.5432538; geodeticDatum: WGS84; coordinatePrecision: 0.0002; **Identification:** identifiedBy: M. Mei; **Event:** eventDate: 2022-05-24; **Record Level:** collectionCode: UR3
- **Type status:** Other material. **Occurrence:** catalogNumber: A0740; recordedBy: L. Fortini; individualCount: 1; sex: male; lifeStage: adult; occurrenceID: 58F510BF-8CDD-5CEF-A96B-7895236BF95E; **Taxon:** scientificName: Eucera (Hetereucera) oraniensis Lepeletier, 1841; order: Hymenoptera; family: Apidae; genus: Eucera; subgenus: Hetereucera; specificEpithet: oraniensis; scientificNameAuthorship: Lepeletier, 1841; **Location:** country: Italy; countryCode: IT; stateProvince: Roma; locality: Riserva Naturale Laurentino-Acqua Acetosa; decimalLatitude: 41.8079275; decimalLongitude: 12.4685548; geodeticDatum: WGS84; coordinatePrecision: 0.0002; **Identification:** identifiedBy: M. Mei; **Event:** eventDate: 2022-04-12; **Record Level:** collectionCode: UR3
- **Type status:** Other material. **Occurrence:** catalogNumber: A0775, A0776, A0779, A0807; recordedBy: L. Fortini; individualCount: 4; sex: females; lifeStage: adult; occurrenceID: 2F494E6E-C844-576C-9BC6-D5B00C4CDC6B; **Taxon:** scientificName: Eucera (Hetereucera) oraniensis Lepeletier, 1841; order: Hymenoptera; family: Apidae; genus: Eucera; subgenus: Hetereucera; specificEpithet: oraniensis; scientificNameAuthorship: Lepeletier, 1841; **Location:** country: Italy; countryCode: IT; stateProvince: Roma; locality: Riserva Naturale Laurentino-Acqua Acetosa; decimalLatitude: 41.8079275; decimalLongitude: 12.4685548; geodeticDatum: WGS84; coordinatePrecision: 0.0002; **Identification:** identifiedBy: M. Mei; **Event:** eventDate: 2022-05-12; **Record Level:** collectionCode: UR3
- **Type status:** Other material. **Occurrence:** catalogNumber: A0788, A0806; recordedBy: L. Fortini; individualCount: 2; sex: females; lifeStage: adult; occurrenceID: 58E058DE-4795-55EE-AB1B-6701EF47AE39; **Taxon:** scientificName: Eucera (Hetereucera) oraniensis Lepeletier, 1841; order: Hymenoptera; family: Apidae; genus: Eucera; subgenus: Hetereucera; specificEpithet: oraniensis; scientificNameAuthorship: Lepeletier, 1841; **Location:** country: Italy; countryCode: IT; stateProvince: Roma; locality: Riserva Naturale Laurentino-Acqua Acetosa; decimalLatitude: 41.8079275; decimalLongitude: 12.4685548; geodeticDatum: WGS84; coordinatePrecision: 0.0002; **Identification:** identifiedBy: M. Mei; **Event:** eventDate: 2022-06-16; **Record Level:** collectionCode: UR3
- **Type status:** Other material. **Occurrence:** catalogNumber: A0741; recordedBy: L. Fortini; individualCount: 1; sex: male; lifeStage: adult; occurrenceID: A0248F6C-370D-55F6-9D24-8D7565A525CD; **Taxon:** scientificName: Eucera (Hetereucera) oraniensis Lepeletier, 1841; order: Hymenoptera; family: Apidae; genus: Eucera; subgenus: Hetereucera; specificEpithet: oraniensis; scientificNameAuthorship: Lepeletier, 1841; **Location:** country: Italy; countryCode: IT; stateProvince: Roma; locality: Riserva Naturale Valle dei Casali 1; decimalLatitude: 41.8710627; decimalLongitude: 12.4336809; geodeticDatum: WGS84; coordinatePrecision: 0.0002; **Identification:** identifiedBy: M. Mei; **Event:** eventDate: 2022-04-07; **Record Level:** collectionCode: UR3

#### Eucera pannonica

Mocsáry, 1878

6EF3334C-E78D-50A8-9B3A-FDDB9318145B

##### Materials

- **Type status:** Other material. **Occurrence:** catalogNumber: A0812; recordedBy: L. Fortini; individualCount: 1; sex: female; lifeStage: adult; occurrenceID: 7ABFBF1A-5E6B-5745-A2EC-2880CA375044; **Taxon:** scientificName: Eucera (Hetereucera) pannonica Mocsáry, 1878; order: Hymenoptera; family: Apidae; genus: Eucera; subgenus: Hetereucera; specificEpithet: pannonica; scientificNameAuthorship: Mocsáry, 1878; **Location:** country: Italy; countryCode: IT; stateProvince: Roma; locality: Riserva Naturale dell'Insugherata 1; decimalLatitude: 41.9555045; decimalLongitude: 12.4292321; geodeticDatum: WGS84; coordinatePrecision: 0.0002; **Identification:** identifiedBy: M. Mei; **Event:** eventDate: 2022-06-24; **Record Level:** collectionCode: UR3

#### Eucera pollinosa

Smith, 1854

FFB307AA-24F8-53FD-A1E8-4E1AFA8C7C1B

##### Materials

- **Type status:** Other material. **Occurrence:** catalogNumber: A0750, A0757, A0758, A0762, A0765, A0766; recordedBy: L. Fortini; individualCount: 6; sex: 2 males, 4 females; lifeStage: adult; occurrenceID: 924D023C-99F5-58D9-9692-A0E26C06B6CD; **Taxon:** scientificName: Eucera (Eucera) pollinosa Smith, 1854; order: Hymenoptera; family: Apidae; genus: Eucera; subgenus: Eucera; specificEpithet: pollinosa; scientificNameAuthorship: Smith, 1854; **Location:** country: Italy; countryCode: IT; stateProvince: Roma; locality: Riserva Naturale dell'Insugherata 2; decimalLatitude: 41.9599247; decimalLongitude: 12.433852; geodeticDatum: WGS84; coordinatePrecision: 0.0002; **Identification:** identifiedBy: M. Mei; **Event:** eventDate: 2022-05-27; **Record Level:** collectionCode: UR3
- **Type status:** Other material. **Occurrence:** catalogNumber: A0756; recordedBy: L. Fortini; individualCount: 1; sex: female; lifeStage: adult; occurrenceID: 4E999525-98BE-5372-AAB2-CA09AD937C67; **Taxon:** scientificName: Eucera (Eucera) pollinosa Smith, 1854; order: Hymenoptera; family: Apidae; genus: Eucera; subgenus: Eucera; specificEpithet: pollinosa; scientificNameAuthorship: Smith, 1854; **Location:** country: Italy; countryCode: IT; stateProvince: Roma; locality: Riserva Naturale Laurentino-Acqua Acetosa; decimalLatitude: 41.8079275; decimalLongitude: 12.4685548; geodeticDatum: WGS84; coordinatePrecision: 0.0002; **Identification:** identifiedBy: M. Mei; **Event:** eventDate: 2022-06-16; **Record Level:** collectionCode: UR3
- **Type status:** Other material. **Occurrence:** catalogNumber: A0764; recordedBy: L. Fortini; individualCount: 1; sex: male; lifeStage: adult; occurrenceID: A155B66B-D00C-59B7-B2AA-2778D67C3545; **Taxon:** scientificName: Eucera (Eucera) pollinosa Smith, 1854; order: Hymenoptera; family: Apidae; genus: Eucera; subgenus: Eucera; specificEpithet: pollinosa; scientificNameAuthorship: Smith, 1854; **Location:** country: Italy; countryCode: IT; stateProvince: Roma; locality: Riserva Naturale Valle dell'Aniene 2; decimalLatitude: 41.928752; decimalLongitude: 12.5562962; geodeticDatum: WGS84; coordinatePrecision: 0.0002; **Identification:** identifiedBy: M. Mei; **Event:** eventDate: 2022-06-05; **Record Level:** collectionCode: UR3

#### Eucera rufa

(Lepeletier, 1841)

9F0BEE64-510F-538E-95DA-01F1E2743D45

##### Materials

- **Type status:** Other material. **Occurrence:** catalogNumber: A0693, A0694, A0695, A0696, A0697, A0698; recordedBy: L. Fortini; individualCount: 6; sex: males; lifeStage: adult; occurrenceID: 19475302-F3FE-5DD6-881E-A05B5871C0D9; **Taxon:** scientificName: Eucera (Synhalonia) rufa (Lepeletier, 1841); order: Hymenoptera; family: Apidae; genus: Eucera; subgenus: Synhalonia; specificEpithet: rufa; scientificNameAuthorship: (Lepeletier, 1841); **Location:** country: Italy; countryCode: IT; stateProvince: Roma; locality: Riserva Regionale dell'Appia Antica 1; decimalLatitude: 41.8623941; decimalLongitude: 12.524863; geodeticDatum: WGS84; coordinatePrecision: 0.0002; **Identification:** identifiedBy: M. Mei; **Event:** eventDate: 2022-05-10; **Record Level:** collectionCode: UR3
- **Type status:** Other material. **Occurrence:** catalogNumber: A0699; recordedBy: L. Fortini; individualCount: 1; sex: female; lifeStage: adult; occurrenceID: 1AEAA81F-2B4D-5BF1-BF8A-509260F8E171; **Taxon:** scientificName: Eucera (Synhalonia) rufa (Lepeletier, 1841); order: Hymenoptera; family: Apidae; genus: Eucera; subgenus: Synhalonia; specificEpithet: rufa; scientificNameAuthorship: (Lepeletier, 1841); **Location:** country: Italy; countryCode: IT; stateProvince: Roma; locality: Riserva Naturale Laurentino-Acqua Acetosa; decimalLatitude: 41.8079275; decimalLongitude: 12.4685548; geodeticDatum: WGS84; coordinatePrecision: 0.0002; **Identification:** identifiedBy: M. Mei; **Event:** eventDate: 2022-05-12; **Record Level:** collectionCode: UR3
- **Type status:** Other material. **Occurrence:** catalogNumber: A2002; recordedBy: L. Fortini; individualCount: 1; sex: male; lifeStage: adult; occurrenceID: 58F3EB29-5F34-5B04-9AC9-43E23146FFE0; **Taxon:** scientificName: Eucera (Synhalonia) rufa (Lepeletier, 1841); order: Hymenoptera; family: Apidae; genus: Eucera; subgenus: Synhalonia; specificEpithet: rufa; scientificNameAuthorship: (Lepeletier, 1841); **Location:** country: Italy; countryCode: IT; stateProvince: Roma; locality: Riserva Regionale dell'Appia Antica 1; decimalLatitude: 41.8623941; decimalLongitude: 12.524863; geodeticDatum: WGS84; coordinatePrecision: 0.0002; **Identification:** identifiedBy: M. Mei; **Event:** eventDate: 2022-04-19; **Record Level:** collectionCode: UR3
- **Type status:** Other material. **Occurrence:** catalogNumber: A2024, A2025, A2027; recordedBy: L. Fortini; individualCount: 3; sex: males; lifeStage: adult; occurrenceID: 8CD8EA5B-AB7B-53C2-977B-76E8BB2295B2; **Taxon:** scientificName: Eucera (Synhalonia) rufa (Lepeletier, 1841); order: Hymenoptera; family: Apidae; genus: Eucera; subgenus: Synhalonia; specificEpithet: rufa; scientificNameAuthorship: (Lepeletier, 1841); **Location:** country: Italy; countryCode: IT; stateProvince: Roma; locality: Riserva Regionale dell'Appia Antica 2; decimalLatitude: 41.8402564; decimalLongitude: 12.532773; geodeticDatum: WGS84; coordinatePrecision: 0.0002; **Identification:** identifiedBy: M. Mei; **Event:** eventDate: 2022-04-25; **Record Level:** collectionCode: UR3

#### Eucera vulpes

Brullé, 1832

5686F196-6685-5A07-98AF-9ECE7236FE52

##### Materials

- **Type status:** Other material. **Occurrence:** catalogNumber: A0714; recordedBy: L. Fortini; individualCount: 1; sex: male; lifeStage: adult; occurrenceID: 30F37CEA-6BDB-5E2B-AA75-62368A3D4395; **Taxon:** scientificName: Eucera (Hetereucera) vulpes Brullé, 1832; order: Hymenoptera; family: Apidae; genus: Eucera; subgenus: Hetereucera; specificEpithet: vulpes; scientificNameAuthorship: Brullé, 1832; **Location:** country: Italy; countryCode: IT; stateProvince: Roma; locality: Riserva Naturale Valle dei Casali 2; decimalLatitude: 41.8596887; decimalLongitude: 12.4355075; geodeticDatum: WGS84; coordinatePrecision: 0.0002; **Identification:** identifiedBy: M. Mei; **Event:** eventDate: 2022-05-14; **Record Level:** collectionCode: UR3

#### Eupavlovskia funeraria

(Smith, 1854)

A17F03F5-576F-5C74-AC04-16E255FE5938

##### Materials

- **Type status:** Other material. **Occurrence:** catalogNumber: A0456; recordedBy: L. Fortini; individualCount: 1; sex: male; lifeStage: adult; occurrenceID: C1C0700C-3818-5740-A880-8B60326C242A; **Taxon:** scientificName: Eupavlovskia (Eupavlovskia) funeraria Smith, 1854; order: Hymenoptera; family: Apidae; genus: Eupavlovskia; subgenus: Eupavlovskia; specificEpithet: funeraria; scientificNameAuthorship: Smith, 1854; **Location:** country: Italy; countryCode: IT; stateProvince: Roma; locality: Riserva Regionale dell'Appia Antica 1; decimalLatitude: 41.8623941; decimalLongitude: 12.524863; geodeticDatum: WGS84; coordinatePrecision: 0.0002; **Identification:** identifiedBy: M. Mei; **Event:** eventDate: 2022-05-10; **Record Level:** collectionCode: UR3

#### Eupavlovskia obscura

(Friese, 1895)

56093BB5-F954-5B2F-8C95-8145A1D33572

##### Materials

- **Type status:** Other material. **Occurrence:** catalogNumber: A0455; recordedBy: L. Fortini; individualCount: 1; sex: male; lifeStage: adult; occurrenceID: F1FED204-E1E0-505C-AA11-3BD329396785; **Taxon:** scientificName: Eupavlovskia (Eupavlovskia) obscura Friese, 1895; order: Hymenoptera; family: Apidae; genus: Eupavlovskia; subgenus: Eupavlovskia; specificEpithet: obscura; scientificNameAuthorship: Friese, 1895; **Location:** country: Italy; countryCode: IT; stateProvince: Roma; locality: Riserva Naturale Laurentino-Acqua Acetosa; decimalLatitude: 41.8079275; decimalLongitude: 12.4685548; geodeticDatum: WGS84; coordinatePrecision: 0.0002; **Identification:** identifiedBy: M. Mei; **Event:** eventDate: 2022-04-12; **Record Level:** collectionCode: UR3

#### Habropoda tarsata

Smith, 1854

B084C7E7-F922-5957-9AE6-C4369A0DF513

##### Materials

- **Type status:** Other material. **Occurrence:** catalogNumber: A0349, A0353, A0363; recordedBy: L. Fortini; individualCount: 3; sex: 2 males, 1 female; lifeStage: adult; occurrenceID: 06450B00-7141-55C9-BC6B-9D657B99D9D4; **Taxon:** scientificName: Habropoda (Habropoda) tarsata (Spinola, 1838); order: Hymenoptera; family: Apidae; genus: Habropoda; specificEpithet: tarsata; scientificNameAuthorship: (Spinola, 1838); **Location:** country: Italy; countryCode: IT; stateProvince: Roma; locality: Riserva Naturale Laurentino-Acqua Acetosa; decimalLatitude: 41.8079275; decimalLongitude: 12.4685548; geodeticDatum: WGS84; coordinatePrecision: 0.0002; **Identification:** identifiedBy: L. Fortini; **Event:** eventDate: 2022-04-12; **Record Level:** collectionCode: UR3
- **Type status:** Other material. **Occurrence:** catalogNumber: A0366; recordedBy: L. Fortini; individualCount: 1; sex: female; lifeStage: adult; occurrenceID: 84C42629-27AE-5145-B026-9F6AD719B50F; **Taxon:** scientificName: Habropoda (Habropoda) tarsata (Spinola, 1838); order: Hymenoptera; family: Apidae; genus: Habropoda; specificEpithet: tarsata; scientificNameAuthorship: (Spinola, 1838); **Location:** country: Italy; countryCode: IT; stateProvince: Roma; locality: Riserva Naturale dell'Insugherata 1; decimalLatitude: 41.9555045; decimalLongitude: 12.4292321; geodeticDatum: WGS84; coordinatePrecision: 0.0002; **Identification:** identifiedBy: L. Fortini; **Event:** eventDate: 2022-04-15; **Record Level:** collectionCode: UR3
- **Type status:** Other material. **Occurrence:** catalogNumber: A0350, A0367; recordedBy: L. Fortini; individualCount: 2; sex: 1 male, 1 female; lifeStage: adult; occurrenceID: 31422F4E-14B9-59A7-B1B4-692835017F5E; **Taxon:** scientificName: Habropoda (Habropoda) tarsata (Spinola, 1838); order: Hymenoptera; family: Apidae; genus: Habropoda; specificEpithet: tarsata; scientificNameAuthorship: (Spinola, 1838); **Location:** country: Italy; countryCode: IT; stateProvince: Roma; locality: Riserva Naturale dell'Insugherata 2; decimalLatitude: 41.9599247; decimalLongitude: 12.433852; geodeticDatum: WGS84; coordinatePrecision: 0.0002; **Identification:** identifiedBy: L. Fortini; **Event:** eventDate: 2022-04-15; **Record Level:** collectionCode: UR3
- **Type status:** Other material. **Occurrence:** catalogNumber: A0351, A0355, A0358; recordedBy: L. Fortini; individualCount: 3; sex: males; lifeStage: adult; occurrenceID: 465E7A4A-BC30-5AB8-9A0B-272C2EB48903; **Taxon:** scientificName: Habropoda (Habropoda) tarsata (Spinola, 1838); order: Hymenoptera; family: Apidae; genus: Habropoda; specificEpithet: tarsata; scientificNameAuthorship: (Spinola, 1838); **Location:** country: Italy; countryCode: IT; stateProvince: Roma; locality: Riserva Naturale dell'Acquafredda; decimalLatitude: 41.8928408; decimalLongitude: 12.39932; geodeticDatum: WGS84; coordinatePrecision: 0.0002; **Identification:** identifiedBy: L. Fortini; **Event:** eventDate: 2022-04-13; **Record Level:** collectionCode: UR3
- **Type status:** Other material. **Occurrence:** catalogNumber: A0352, A0354, A0356, A0357, A359, A0361, A0364, A0368, A0370; recordedBy: L. Fortini; individualCount: 9; sex: 6 males, 3 females; lifeStage: adult; occurrenceID: 9CD9BC1B-00B9-558F-B1E6-69A875E8A3A7; **Taxon:** scientificName: Habropoda (Habropoda) tarsata (Spinola, 1838); order: Hymenoptera; family: Apidae; genus: Habropoda; specificEpithet: tarsata; scientificNameAuthorship: (Spinola, 1838); **Location:** country: Italy; countryCode: IT; stateProvince: Roma; locality: Riserva Naturale di Monte Mario; decimalLatitude: 41.9386215; decimalLongitude: 12.4546223; geodeticDatum: WGS84; coordinatePrecision: 0.0002; **Identification:** identifiedBy: L. Fortini; **Event:** eventDate: 2022-04-20; **Record Level:** collectionCode: UR3
- **Type status:** Other material. **Occurrence:** catalogNumber: A0360, A0362; recordedBy: L. Fortini; individualCount: 2; sex: 1 male, 1 female; lifeStage: adult; occurrenceID: 5ED2A1CB-AF27-5F66-9F46-24EC87B3013B; **Taxon:** scientificName: Habropoda (Habropoda) tarsata (Spinola, 1838); order: Hymenoptera; family: Apidae; genus: Habropoda; specificEpithet: tarsata; scientificNameAuthorship: (Spinola, 1838); **Location:** country: Italy; countryCode: IT; stateProvince: Roma; locality: Riserva Naturale Tenuta dei Massimi 2; decimalLatitude: 41.8316516; decimalLongitude: 12.3999927; geodeticDatum: WGS84; coordinatePrecision: 0.0002; **Identification:** identifiedBy: L. Fortini; **Event:** eventDate: 2022-05-04; **Record Level:** collectionCode: UR3
- **Type status:** Other material. **Occurrence:** catalogNumber: A0365, A0369; recordedBy: L. Fortini; individualCount: 2; sex: females; lifeStage: adult; occurrenceID: 06E5A2DA-27D4-511F-BB34-129FB32DD413; **Taxon:** scientificName: Habropoda (Habropoda) tarsata (Spinola, 1838); order: Hymenoptera; family: Apidae; genus: Habropoda; specificEpithet: tarsata; scientificNameAuthorship: (Spinola, 1838); **Location:** country: Italy; countryCode: IT; stateProvince: Roma; locality: Riserva Regionale dell'Appia Antica 1; decimalLatitude: 41.8623941; decimalLongitude: 12.524863; geodeticDatum: WGS84; coordinatePrecision: 0.0002; **Identification:** identifiedBy: L. Fortini; **Event:** eventDate: 2022-05-10; **Record Level:** collectionCode: UR3
- **Type status:** Other material. **Occurrence:** catalogNumber: A2004, A2007; recordedBy: L. Fortini; individualCount: 2; sex: females; lifeStage: adult; occurrenceID: 39243911-2465-553C-93A7-315860D95320; **Taxon:** scientificName: Habropoda (Habropoda) tarsata (Spinola, 1838); order: Hymenoptera; family: Apidae; genus: Habropoda; specificEpithet: tarsata; scientificNameAuthorship: (Spinola, 1838); **Location:** country: Italy; countryCode: IT; stateProvince: Roma; locality: Riserva Regionale dell'Appia Antica 2; decimalLatitude: 41.8402564; decimalLongitude: 12.532773; geodeticDatum: WGS84; coordinatePrecision: 0.0002; **Identification:** identifiedBy: L. Fortini; **Event:** eventDate: 2022-04-25; **Record Level:** collectionCode: UR3

#### Habropoda zonatula

Smith, 1854

1700662D-EF1D-591E-95BB-7E4501DBCA85

##### Materials

- **Type status:** Other material. **Occurrence:** catalogNumber: A0347, A0348; recordedBy: L. Fortini; individualCount: 2; sex: males; lifeStage: adult; occurrenceID: 82426F10-F49E-5651-87AB-C08484CDFED3; **Taxon:** scientificName: Habropoda (Habropoda) zonatula Smith, 1854; order: Hymenoptera; family: Apidae; genus: Habropoda; subgenus: Habropoda; specificEpithet: zonatula; scientificNameAuthorship: (Smith, 1854); **Location:** country: Italy; countryCode: IT; stateProvince: Roma; locality: Riserva Regionale dell'Appia Antica 1; decimalLatitude: 41.8623941; decimalLongitude: 12.524863; geodeticDatum: WGS84; coordinatePrecision: 0.0002; **Identification:** identifiedBy: L. Fortini; **Event:** eventDate: 2022-05-10; **Record Level:** collectionCode: UR3

#### Melecta aegyptiaca

(Förster, 1771)

3989BEE2-C962-56AC-939C-52FCA8EA3114

##### Materials

- **Type status:** Other material. **Occurrence:** catalogNumber: A0454; recordedBy: L. Fortini; individualCount: 1; sex: male; lifeStage: adult; occurrenceID: FEC38631-0D05-53C7-93E4-0D467DB494C6; **Taxon:** scientificName: Melecta (Melecta) aegyptiaca Radoszkowski, 1876; order: Hymenoptera; family: Apidae; genus: Melecta; subgenus: Melecta; specificEpithet: aegyptiaca; scientificNameAuthorship: Radoszkowski, 1876; **Location:** country: Italy; countryCode: IT; stateProvince: Roma; locality: Riserva Naturale Valle dell'Aniene 1; decimalLatitude: 41.9345179; decimalLongitude: 12.5453096; geodeticDatum: WGS84; coordinatePrecision: 0.0002; **Identification:** identifiedBy: M. Mei; **Event:** eventDate: 2022-04-28; **Record Level:** collectionCode: UR3

#### Melecta albifrons

Herrich-Schäffer, 1839

F31A8554-B599-5CFB-8B89-73B8999B8254

##### Materials

- **Type status:** Other material. **Occurrence:** catalogNumber: A0457; recordedBy: L. Fortini; individualCount: 1; sex: male; lifeStage: adult; occurrenceID: 5238B2FA-3CC1-5EC7-A458-CBB9850F9558; **Taxon:** scientificName: Melecta (Melecta) albifrons (Förster, 1771); order: Hymenoptera; family: Apidae; genus: Melecta; subgenus: Melecta; specificEpithet: albifrons; scientificNameAuthorship: (Forster, 1771); **Location:** country: Italy; countryCode: IT; stateProvince: Roma; locality: Riserva Naturale dell'Acquafredda; decimalLatitude: 41.8928408; decimalLongitude: 12.39932; geodeticDatum: WGS84; coordinatePrecision: 0.0002; **Identification:** identifiedBy: M. Mei; **Event:** eventDate: 2022-04-13; **Record Level:** collectionCode: UR3
- **Type status:** Other material. **Occurrence:** catalogNumber: A0458; recordedBy: L. Fortini; individualCount: 1; sex: male; lifeStage: adult; occurrenceID: 4E60F19D-D4AF-59DB-ADD5-5BB7B94BD28B; **Taxon:** scientificName: Melecta (Melecta) albifrons (Förster, 1771); order: Hymenoptera; family: Apidae; genus: Melecta; subgenus: Melecta; specificEpithet: albifrons; scientificNameAuthorship: (Forster, 1771); **Location:** country: Italy; countryCode: IT; stateProvince: Roma; locality: Riserva Naturale Laurentino-Acqua Acetosa; decimalLatitude: 41.8079275; decimalLongitude: 12.4685548; geodeticDatum: WGS84; coordinatePrecision: 0.0002; **Identification:** identifiedBy: M. Mei; **Event:** eventDate: 2022-04-12; **Record Level:** collectionCode: UR3

#### Nomada argentata

Herrich-Schäffer, 1839

20B2A505-90A2-52C1-951E-C716160DEEC5

##### Materials

- **Type status:** Other material. **Occurrence:** catalogNumber: A0478; recordedBy: L. Fortini; individualCount: 1; sex: male; lifeStage: adult; occurrenceID: 5D9024DA-D390-5933-928B-15A43024A142; **Taxon:** scientificName: Nomada (Nomada) argentata Herrich-Schäffer,1839; order: Hymenoptera; family: Apidae; genus: Nomada; subgenus: Nomada; specificEpithet: argentata; scientificNameAuthorship: Herrich-Schäffer,1839; **Location:** country: Italy; countryCode: IT; stateProvince: Roma; locality: Riserva Regionale dell'Appia Antica 2; decimalLatitude: 41.8402564; decimalLongitude: 12.532773; geodeticDatum: WGS84; coordinatePrecision: 0.0002; **Identification:** identifiedBy: M. Mei; **Event:** eventDate: 2022-10-01; **Record Level:** collectionCode: UR3

#### Nomada basalis

97DE5E9B-E024-5755-A318-1970FF151F86

##### Materials

- **Type status:** Other material. **Occurrence:** catalogNumber: A0470; recordedBy: L. Fortini; individualCount: 1; sex: male; lifeStage: adult; occurrenceID: 1510190C-A9B0-5DB1-A17A-C726FDA0FE05; **Taxon:** scientificName: Nomada (Nomada) basalis Herrich-Schäffer, 1839; order: Hymenoptera; family: Apidae; genus: Nomada; subgenus: Nomada; specificEpithet: basalis; scientificNameAuthorship: Herrich-Schäffer, 1839; **Location:** country: Italy; countryCode: IT; stateProvince: Roma; locality: Riserva Naturale Tenuta dei Massimi 2; decimalLatitude: 41.8316516; decimalLongitude: 12.3999927; geodeticDatum: WGS84; coordinatePrecision: 0.0002; **Identification:** identifiedBy: M. Mei; **Event:** eventDate: 2022-05-04; **Record Level:** collectionCode: UR3
- **Type status:** Other material. **Occurrence:** catalogNumber: A0471; recordedBy: L. Fortini; individualCount: 1; sex: male; lifeStage: adult; occurrenceID: D02060E8-846D-5B89-8C5B-3F213211CD47; **Taxon:** scientificName: Nomada (Nomada) basalis Herrich-Schäffer, 1839; order: Hymenoptera; family: Apidae; genus: Nomada; subgenus: Nomada; specificEpithet: basalis; scientificNameAuthorship: Herrich-Schäffer, 1839; **Location:** country: Italy; countryCode: IT; stateProvince: Roma; locality: Riserva Naturale Laurentino-Acqua Acetosa; decimalLatitude: 41.8079275; decimalLongitude: 12.4685548; geodeticDatum: WGS84; coordinatePrecision: 0.0002; **Identification:** identifiedBy: M. Mei; **Event:** eventDate: 2022-05-12; **Record Level:** collectionCode: UR3
- **Type status:** Other material. **Occurrence:** catalogNumber: A0472; recordedBy: L. Fortini; individualCount: 1; sex: male; lifeStage: adult; occurrenceID: 4AB83BAB-FC99-5A82-814D-28B576808FE3; **Taxon:** scientificName: Nomada (Nomada) basalis Herrich-Schäffer, 1839; order: Hymenoptera; family: Apidae; genus: Nomada; subgenus: Nomada; specificEpithet: basalis; scientificNameAuthorship: Herrich-Schäffer, 1839; **Location:** country: Italy; countryCode: IT; stateProvince: Roma; locality: Riserva Naturale Valle dei Casali 2; decimalLatitude: 41.8596887; decimalLongitude: 12.4355075; geodeticDatum: WGS84; coordinatePrecision: 0.0002; **Identification:** identifiedBy: M. Mei; **Event:** eventDate: 2022-05-14; **Record Level:** collectionCode: UR3

#### Nomada distinguenda

Pérez, 1913

03621743-FB53-5158-B420-51FB48D98FA1

##### Materials

- **Type status:** Other material. **Occurrence:** catalogNumber: A0480; recordedBy: L. Fortini; individualCount: 1; sex: male; lifeStage: adult; occurrenceID: 9A71FD6B-DA92-54E7-A061-971522DEDD45; **Taxon:** scientificName: Nomada (Nomada) distinguenda Morawiz, 1874; order: Hymenoptera; family: Apidae; genus: Nomada; subgenus: Nomada; specificEpithet: distinguenda; scientificNameAuthorship: Morawiz, 1874; **Location:** country: Italy; countryCode: IT; stateProvince: Roma; locality: Riserva Naturale Tenuta dei Massimi 1; decimalLatitude: 41.8532859; decimalLongitude: 12.3842322; geodeticDatum: WGS84; coordinatePrecision: 0.0002; **Identification:** identifiedBy: M. Mei; **Event:** eventDate: 2022-06-27; **Record Level:** collectionCode: UR3

#### Nomada fallax

Pérez, 1913

BA979498-F25A-546F-9967-291DACF328C3

##### Materials

- **Type status:** Other material. **Occurrence:** catalogNumber: A0475; recordedBy: L. Fortini; individualCount: 1; sex: male; lifeStage: adult; occurrenceID: 47240AAE-D1B3-59FE-BB47-A79277D51929; **Taxon:** scientificName: Nomada (Nomada) fallax Pérez, 1913; order: Hymenoptera; family: Apidae; genus: Nomada; subgenus: Nomada; specificEpithet: fallax; scientificNameAuthorship: Pérez, 1913; **Location:** country: Italy; countryCode: IT; stateProvince: Roma; locality: Riserva Naturale Laurentino-Acqua Acetosa; decimalLatitude: 41.8079275; decimalLongitude: 12.4685548; geodeticDatum: WGS84; coordinatePrecision: 0.0002; **Identification:** identifiedBy: M. Mei; **Event:** eventDate: 2022-04-12; **Record Level:** collectionCode: UR3

#### Nomada femoralis

Schmiedeknecht, 1882

328C02DB-7144-5A42-BAFC-A61B36EA877E

##### Materials

- **Type status:** Other material. **Occurrence:** catalogNumber: A0474, A0476; recordedBy: L. Fortini; individualCount: 2; sex: males; lifeStage: adult; occurrenceID: 96713E0D-90B8-57C4-9BAF-C15A2CDFC185; **Taxon:** scientificName: Nomada (Nomada) femoralis Morawitz, 1869; order: Hymenoptera; family: Apidae; genus: Nomada; subgenus: Nomada; specificEpithet: femoralis; scientificNameAuthorship: Morawitz, 1869; **Location:** country: Italy; countryCode: IT; stateProvince: Roma; locality: Riserva Naturale Laurentino-Acqua Acetosa; decimalLatitude: 41.8079275; decimalLongitude: 12.4685548; geodeticDatum: WGS84; coordinatePrecision: 0.0002; **Identification:** identifiedBy: M. Mei; **Event:** eventDate: 2022-04-12; **Record Level:** collectionCode: UR3

#### Nomada insignipes

(Panzer, 1799)

03AB05E1-2CB6-53AA-B9DA-8066C49F76DD

##### Materials

- **Type status:** Other material. **Occurrence:** catalogNumber: A0477; recordedBy: L. Fortini; individualCount: 1; sex: male; lifeStage: adult; occurrenceID: 0FE542C2-361F-5CDB-BEF8-7CD1C6D5EC0F; **Taxon:** scientificName: Nomada (Nomada) insignipes Schmiedeknecht, 1882; order: Hymenoptera; family: Apidae; genus: Nomada; subgenus: Nomada; specificEpithet: insignipes; scientificNameAuthorship: Schmiedeknecht, 1882; **Location:** country: Italy; countryCode: IT; stateProvince: Roma; locality: Riserva Naturale Laurentino-Acqua Acetosa; decimalLatitude: 41.8079275; decimalLongitude: 12.4685548; geodeticDatum: WGS84; coordinatePrecision: 0.0002; **Identification:** identifiedBy: M. Mei; **Event:** eventDate: 2022-05-12; **Record Level:** collectionCode: UR3

#### Nomada sexfasciata

(Kirby, 1802)

FBEAAEC3-3D7E-52FA-9C91-7ADCB19B98D4

##### Materials

- **Type status:** Other material. **Occurrence:** catalogNumber: A0465; recordedBy: L. Fortini; individualCount: 1; sex: female; lifeStage: adult; occurrenceID: A460F609-CEF4-591D-B4F7-18AEB28759E3; **Taxon:** scientificName: Nomada (Nomada) sexfasciata Panzer,1799; order: Hymenoptera; family: Apidae; genus: Nomada; subgenus: Nomada; specificEpithet: sexfasciata; scientificNameAuthorship: Panzer, 1799; **Location:** country: Italy; countryCode: IT; stateProvince: Roma; locality: Riserva Naturale Valle dell'Aniene 2; decimalLatitude: 41.928752; decimalLongitude: 12.5562962; geodeticDatum: WGS84; coordinatePrecision: 0.0002; **Identification:** identifiedBy: M. Mei; **Event:** eventDate: 2022-04-28; **Record Level:** collectionCode: UR3
- **Type status:** Other material. **Occurrence:** catalogNumber: A0466, A0473; recordedBy: L. Fortini; individualCount: 2; sex: males; lifeStage: adult; occurrenceID: E5D99D90-8EC8-589A-85A0-4C5A67CF81EA; **Taxon:** scientificName: Nomada (Nomada) sexfasciata Panzer,1799; order: Hymenoptera; family: Apidae; genus: Nomada; subgenus: Nomada; specificEpithet: sexfasciata; scientificNameAuthorship: Panzer, 1799; **Location:** country: Italy; countryCode: IT; stateProvince: Roma; locality: Riserva Naturale Laurentino-Acqua Acetosa; decimalLatitude: 41.8079275; decimalLongitude: 12.4685548; geodeticDatum: WGS84; coordinatePrecision: 0.0002; **Identification:** identifiedBy: M. Mei; **Event:** eventDate: 2022-04-12; **Record Level:** collectionCode: UR3
- **Type status:** Other material. **Occurrence:** catalogNumber: A1970; recordedBy: L. Fortini; individualCount: 1; sex: female; lifeStage: adult; occurrenceID: 77C0D1FF-8C5D-5E02-BAC3-4F6DB330C304; **Taxon:** scientificName: Nomada (Nomada) sexfasciata Panzer,1799; order: Hymenoptera; family: Apidae; genus: Nomada; subgenus: Nomada; specificEpithet: sexfasciata; scientificNameAuthorship: Panzer, 1799; **Location:** country: Italy; countryCode: IT; stateProvince: Roma; locality: Riserva Regionale dell'Appia Antica 1; decimalLatitude: 41.8623941; decimalLongitude: 12.524863; geodeticDatum: WGS84; coordinatePrecision: 0.0002; **Identification:** identifiedBy: M. Mei; **Event:** eventDate: 2022-04-19; **Record Level:** collectionCode: UR3

#### Nomada sheppardana

Dours, 1873

3B39654C-CB43-521E-BAFF-8BF56AFDBCE1

##### Materials

- **Type status:** Other material. **Occurrence:** catalogNumber: A0481; recordedBy: L. Fortini; individualCount: 1; sex: male; lifeStage: adult; occurrenceID: D08D619E-7E37-53D4-9B59-E244B5FBB400; **Taxon:** scientificName: Nomada (Nomada) sheppardana (Kirby, 1802); order: Hymenoptera; family: Apidae; genus: Nomada; subgenus: Nomada; specificEpithet: sheppardana; scientificNameAuthorship: (Kirby, 1802); **Location:** country: Italy; countryCode: IT; stateProvince: Roma; locality: Riserva Regionale dell'Appia Antica 2; decimalLatitude: 41.8402564; decimalLongitude: 12.532773; geodeticDatum: WGS84; coordinatePrecision: 0.0002; **Identification:** identifiedBy: M. Mei; **Event:** eventDate: 2022-05-24; **Record Level:** collectionCode: UR3

#### Nomada tridentirostris

Schmiedeknecht, 1882

0FE77C3E-C374-5723-A671-F3FA16342613

##### Materials

- **Type status:** Other material. **Occurrence:** catalogNumber: A0479; recordedBy: L. Fortini; individualCount: 1; sex: male; lifeStage: adult; occurrenceID: 0C3E7965-C582-5BE6-9B01-43EEE0C10A67; **Taxon:** scientificName: Nomada (Nomada) tridentirostris Dours, 1873; order: Hymenoptera; family: Apidae; genus: Nomada; subgenus: Nomada; specificEpithet: tridentirostris; scientificNameAuthorship: Dours, 1873; **Location:** country: Italy; countryCode: IT; stateProvince: Roma; locality: Riserva Naturale Valle dell'Aniene 1; decimalLatitude: 41.9345179; decimalLongitude: 12.5453096; geodeticDatum: WGS84; coordinatePrecision: 0.0002; **Identification:** identifiedBy: M. Mei; **Event:** eventDate: 2022-04-28; **Record Level:** collectionCode: UR3

#### Nomada verna

(Rossi, 1790)

E7C22919-3C92-5E5D-BD69-81C31D0A66D6

##### Materials

- **Type status:** Other material. **Occurrence:** catalogNumber: A0467; recordedBy: L. Fortini; individualCount: 1; sex: male; lifeStage: adult; occurrenceID: E0A4ED9B-8A7A-5448-91B5-EC0E46D159E4; **Taxon:** scientificName: Nomada (Nomada) verna Schmiedeknecht, 1882; order: Hymenoptera; family: Apidae; genus: Nomada; subgenus: Nomada; specificEpithet: verna; scientificNameAuthorship: Schmiedeknecht, 1882; **Location:** country: Italy; countryCode: IT; stateProvince: Roma; locality: Riserva Naturale dell'Insugherata 3; decimalLatitude: 41.9644829; decimalLongitude: 12.436101; geodeticDatum: WGS84; coordinatePrecision: 0.0002; **Identification:** identifiedBy: M. Mei; **Event:** eventDate: 2022-04-15; **Record Level:** collectionCode: UR3
- **Type status:** Other material. **Occurrence:** catalogNumber: A0468, A0469; recordedBy: L. Fortini; individualCount: 2; sex: males; lifeStage: adult; occurrenceID: 9E5A437B-9B73-5224-B105-4A721469E827; **Taxon:** scientificName: Nomada (Nomada) verna Schmiedeknecht, 1882; order: Hymenoptera; family: Apidae; genus: Nomada; subgenus: Nomada; specificEpithet: verna; scientificNameAuthorship: Schmiedeknecht, 1882; **Location:** country: Italy; countryCode: IT; stateProvince: Roma; locality: Riserva Naturale di Monte Mario; decimalLatitude: 41.9386215; decimalLongitude: 12.4546223; geodeticDatum: WGS84; coordinatePrecision: 0.0002; **Identification:** identifiedBy: M. Mei; **Event:** eventDate: 2022-04-20; **Record Level:** collectionCode: UR3

#### Tetralonia malvae

(Spinola, 1838)

5FFF57D3-5673-5CAF-A4FD-6C37D60530D8

##### Materials

- **Type status:** Other material. **Occurrence:** catalogNumber: A0441; recordedBy: L. Fortini; individualCount: 1; sex: male; lifeStage: adult; occurrenceID: D6076B84-5321-5540-8506-F1AB31316F40; **Taxon:** scientificName: Tetralonia (Tetralonia) malvae (Rossi, 1790); order: Hymenoptera; family: Apidae; genus: Tetralonia; subgenus: Tetralonia; specificEpithet: malvae; scientificNameAuthorship: (Rossi, 1790); **Location:** country: Italy; countryCode: IT; stateProvince: Roma; locality: Riserva Regionale dell'Appia Antica 1; decimalLatitude: 41.8623941; decimalLongitude: 12.524863; geodeticDatum: WGS84; coordinatePrecision: 0.0002; **Identification:** identifiedBy: L. Fortini; **Event:** eventDate: 2022-06-12; **Record Level:** collectionCode: UR3
- **Type status:** Other material. **Occurrence:** catalogNumber: A0440; recordedBy: L. Fortini; individualCount: 1; sex: female; lifeStage: adult; occurrenceID: 4BAAD0B5-2265-5A73-9F2C-1D14CBE6DCF5; **Taxon:** scientificName: Tetralonia (Tetralonia) malvae (Rossi, 1790); order: Hymenoptera; family: Apidae; genus: Tetralonia; subgenus: Tetralonia; specificEpithet: malvae; scientificNameAuthorship: (Rossi, 1790); **Location:** country: Italy; countryCode: IT; stateProvince: Roma; locality: Riserva Naturale dell'Insugherata 1; decimalLatitude: 41.9555045; decimalLongitude: 12.4292321; geodeticDatum: WGS84; coordinatePrecision: 0.0002; **Identification:** identifiedBy: L. Fortini; **Event:** eventDate: 2022-06-24; **Record Level:** collectionCode: UR3
- **Type status:** Other material. **Occurrence:** catalogNumber: A0443; recordedBy: L. Fortini; individualCount: 1; sex: male; lifeStage: adult; occurrenceID: D4A8141C-3E5C-58F2-B4E4-58B4D5434D8E; **Taxon:** scientificName: Tetralonia (Tetralonia) malvae (Rossi, 1790); order: Hymenoptera; family: Apidae; genus: Tetralonia; subgenus: Tetralonia; specificEpithet: malvae; scientificNameAuthorship: (Rossi, 1790); **Location:** country: Italy; countryCode: IT; stateProvince: Roma; locality: Riserva Naturale dell'Insugherata 1; decimalLatitude: 41.9555045; decimalLongitude: 12.4292321; geodeticDatum: WGS84; coordinatePrecision: 0.0002; **Identification:** identifiedBy: L. Fortini; **Event:** eventDate: 2022-05-27; **Record Level:** collectionCode: UR3
- **Type status:** Other material. **Occurrence:** catalogNumber: A0442; recordedBy: L. Fortini; individualCount: 1; sex: female; lifeStage: adult; occurrenceID: 98B3A01D-3F6A-5CE3-B307-2E54F9A4DF46; **Taxon:** scientificName: Tetralonia (Tetralonia) malvae (Rossi, 1790); order: Hymenoptera; family: Apidae; genus: Tetralonia; subgenus: Tetralonia; specificEpithet: malvae; scientificNameAuthorship: (Rossi, 1790); **Location:** country: Italy; countryCode: IT; stateProvince: Roma; locality: Riserva Naturale Laurentino-Acqua Acetosa; decimalLatitude: 41.8079275; decimalLongitude: 12.4685548; geodeticDatum: WGS84; coordinatePrecision: 0.0002; **Identification:** identifiedBy: L. Fortini; **Event:** eventDate: 2022-06-16; **Record Level:** collectionCode: UR3
- **Type status:** Other material. **Occurrence:** catalogNumber: A0444; recordedBy: L. Fortini; individualCount: 1; sex: male; lifeStage: adult; occurrenceID: E4F1E8C7-A1D2-572B-B662-C63C70AFCA89; **Taxon:** scientificName: Tetralonia (Tetralonia) malvae (Rossi, 1790); order: Hymenoptera; family: Apidae; genus: Tetralonia; subgenus: Tetralonia; specificEpithet: malvae; scientificNameAuthorship: (Rossi, 1790); **Location:** country: Italy; countryCode: IT; stateProvince: Roma; locality: Riserva Naturale Valle dell'Aniene 2; decimalLatitude: 41.928752; decimalLongitude: 12.5562962; geodeticDatum: WGS84; coordinatePrecision: 0.0002; **Identification:** identifiedBy: L. Fortini; **Event:** eventDate: 2022-06-05; **Record Level:** collectionCode: UR3
- **Type status:** Other material. **Occurrence:** catalogNumber: A0700, A0701, A0702; recordedBy: L. Fortini; individualCount: 3; sex: males; lifeStage: adult; occurrenceID: 151A11D8-9184-553D-BBA9-5263A845D363; **Taxon:** scientificName: Tetralonia (Tetralonia) malvae (Rossi, 1790); order: Hymenoptera; family: Apidae; genus: Tetralonia; subgenus: Tetralonia; specificEpithet: malvae; scientificNameAuthorship: (Rossi, 1790); **Location:** country: Italy; countryCode: IT; stateProvince: Roma; locality: Riserva Naturale dell'Insugherata 1; decimalLatitude: 41.9555045; decimalLongitude: 12.4292321; geodeticDatum: WGS84; coordinatePrecision: 0.0002; **Identification:** identifiedBy: L. Fortini; **Event:** eventDate: 2022-05-27; **Record Level:** collectionCode: UR3

#### Xylocopa iris

(Christ, 1791)

B5B23E4B-3E93-5A76-87E8-50E7E7EC1D83

##### Materials

- **Type status:** Other material. **Occurrence:** catalogNumber: A0030; recordedBy: L. Fortini; individualCount: 1; sex: female; lifeStage: adult; occurrenceID: F1F63656-F6B2-543A-B3EE-84D849AFFCA1; **Taxon:** scientificName: Xylocopa (Copoxyla) iris (Christ, 1791); order: Hymenoptera; family: Apidae; genus: Xylocopa; subgenus: Copoxyla; specificEpithet: iris; scientificNameAuthorship: (Christ, 1791); **Location:** country: Italy; countryCode: IT; stateProvince: Roma; locality: Riserva Naturale dell'Acquafredda; decimalLatitude: 41.8928408; decimalLongitude: 12.39932; geodeticDatum: WGS84; coordinatePrecision: 0.0002; **Identification:** identifiedBy: L. Fortini; **Event:** eventDate: 2022-04-13; **Record Level:** collectionCode: UR3
- **Type status:** Other material. **Occurrence:** catalogNumber: A0034; recordedBy: L. Fortini; individualCount: 1; sex: female; lifeStage: adult; occurrenceID: E926F7FD-3EB6-5C8E-A34A-DAAF03939410; **Taxon:** scientificName: Xylocopa (Copoxyla) iris (Christ, 1791); order: Hymenoptera; family: Apidae; genus: Xylocopa; subgenus: Copoxyla; specificEpithet: iris; scientificNameAuthorship: (Christ, 1791); **Location:** country: Italy; countryCode: IT; stateProvince: Roma; locality: Riserva Naturale dell'Acquafredda; decimalLatitude: 41.8928408; decimalLongitude: 12.39932; geodeticDatum: WGS84; coordinatePrecision: 0.0002; **Identification:** identifiedBy: L. Fortini; **Event:** eventDate: 2022-05-15; **Record Level:** collectionCode: UR3
- **Type status:** Other material. **Occurrence:** catalogNumber: A0032, A0033; recordedBy: L. Fortini; individualCount: 2; sex: males; lifeStage: adult; occurrenceID: 62B7B7E4-D3B3-5FC5-9A3D-AB58C1BBF6A7; **Taxon:** scientificName: Xylocopa (Copoxyla) iris (Christ, 1791); order: Hymenoptera; family: Apidae; genus: Xylocopa; subgenus: Copoxyla; specificEpithet: iris; scientificNameAuthorship: (Christ, 1791); **Location:** country: Italy; countryCode: IT; stateProvince: Roma; locality: Riserva Regionale dell'Appia Antica 1; decimalLatitude: 41.8623941; decimalLongitude: 12.524863; geodeticDatum: WGS84; coordinatePrecision: 0.0002; **Identification:** identifiedBy: L. Fortini; **Event:** eventDate: 2022-05-10; **Record Level:** collectionCode: UR3
- **Type status:** Other material. **Occurrence:** catalogNumber: A0031; recordedBy: L. Fortini; individualCount: 1; sex: male; lifeStage: adult; occurrenceID: C27DB691-28BA-57BA-8AFD-16E6C954244B; **Taxon:** scientificName: Xylocopa (Copoxyla) iris (Christ, 1791); order: Hymenoptera; family: Apidae; genus: Xylocopa; subgenus: Copoxyla; specificEpithet: iris; scientificNameAuthorship: (Christ, 1791); **Location:** country: Italy; countryCode: IT; stateProvince: Roma; locality: Riserva Naturale dell'Insugherata 3; decimalLatitude: 41.9644829; decimalLongitude: 12.436101; geodeticDatum: WGS84; coordinatePrecision: 0.0002; **Identification:** identifiedBy: L. Fortini; **Event:** eventDate: 2022-05-27; **Record Level:** collectionCode: UR3
- **Type status:** Other material. **Occurrence:** catalogNumber: A0037; recordedBy: L. Fortini; individualCount: 1; sex: female; lifeStage: adult; occurrenceID: D0A9B93A-13AB-532C-BF19-11156BFFA5AC; **Taxon:** scientificName: Xylocopa (Copoxyla) iris (Christ, 1791); order: Hymenoptera; family: Apidae; genus: Xylocopa; subgenus: Copoxyla; specificEpithet: iris; scientificNameAuthorship: (Christ, 1791); **Location:** country: Italy; countryCode: IT; stateProvince: Roma; locality: Riserva Naturale Laurentino-Acqua Acetosa; decimalLatitude: 41.8079275; decimalLongitude: 12.4685548; geodeticDatum: WGS84; coordinatePrecision: 0.0002; **Identification:** identifiedBy: L. Fortini; **Event:** eventDate: 2022-06-16; **Record Level:** collectionCode: UR3
- **Type status:** Other material. **Occurrence:** catalogNumber: A0036; recordedBy: L. Fortini; individualCount: 1; sex: female; lifeStage: adult; occurrenceID: 05D6358A-AEC6-5663-8242-0A8FB0EB233D; **Taxon:** scientificName: Xylocopa (Copoxyla) iris (Christ, 1791); order: Hymenoptera; family: Apidae; genus: Xylocopa; subgenus: Copoxyla; specificEpithet: iris; scientificNameAuthorship: (Christ, 1791); **Location:** country: Italy; countryCode: IT; stateProvince: Roma; locality: Riserva Naturale Valle dell'Aniene 2; decimalLatitude: 41.928752; decimalLongitude: 12.5562962; geodeticDatum: WGS84; coordinatePrecision: 0.0002; **Identification:** identifiedBy: L. Fortini; **Event:** eventDate: 2022-07-01; **Record Level:** collectionCode: UR3
- **Type status:** Other material. **Occurrence:** catalogNumber: A0038; recordedBy: L. Fortini; individualCount: 1; sex: female; lifeStage: adult; occurrenceID: D720EADD-623F-5DFF-A3F0-1396EFB515BD; **Taxon:** scientificName: Xylocopa (Copoxyla) iris (Christ, 1791); order: Hymenoptera; family: Apidae; genus: Xylocopa; subgenus: Copoxyla; specificEpithet: iris; scientificNameAuthorship: (Christ, 1791); **Location:** country: Italy; countryCode: IT; stateProvince: Roma; locality: Riserva Naturale Valle dell'Aniene 2; decimalLatitude: 41.928752; decimalLongitude: 12.5562962; geodeticDatum: WGS84; coordinatePrecision: 0.0002; **Identification:** identifiedBy: L. Fortini; **Event:** eventDate: 2022-04-28; **Record Level:** collectionCode: UR3
- **Type status:** Other material. **Occurrence:** catalogNumber: A0035; recordedBy: L. Fortini; individualCount: 1; sex: female; lifeStage: adult; occurrenceID: 28040B8B-C66A-5F9B-9F5F-AA2CDD0B4A84; **Taxon:** scientificName: Xylocopa (Copoxyla) iris (Christ, 1791); order: Hymenoptera; family: Apidae; genus: Xylocopa; subgenus: Copoxyla; specificEpithet: iris; scientificNameAuthorship: (Christ, 1791); **Location:** country: Italy; countryCode: IT; stateProvince: Roma; locality: Riserva Naturale Valle dei Casali 2; decimalLatitude: 41.8596887; decimalLongitude: 12.4355075; geodeticDatum: WGS84; coordinatePrecision: 0.0002; **Identification:** identifiedBy: L. Fortini; **Event:** eventDate: 2022-05-14; **Record Level:** collectionCode: UR3

#### Xylocopa violacea

(Linnaeus, 1758)

FD7EEF2B-5251-5487-B567-6A7C60AEA80C

##### Materials

- **Type status:** Other material. **Occurrence:** catalogNumber: A0016, A0026; recordedBy: L. Fortini; individualCount: 2; sex: 1 male, 1 female; lifeStage: adult; occurrenceID: 98987F15-96A6-5042-A588-1A0279917ADF; **Taxon:** scientificName: Xylocopa (Xylocopa) violacea (Linnaeus, 1758); order: Hymenoptera; family: Apidae; genus: Xylocopa; subgenus: Xylocopa; specificEpithet: violacea; scientificNameAuthorship: (Linnaeus, 1758); **Location:** country: Italy; countryCode: IT; stateProvince: Roma; locality: Riserva Regionale dell'Appia Antica 1; decimalLatitude: 41.8623941; decimalLongitude: 12.524863; geodeticDatum: WGS84; coordinatePrecision: 0.0002; **Identification:** identifiedBy: L. Fortini; **Event:** eventDate: 2022-06-12; **Record Level:** collectionCode: UR3
- **Type status:** Other material. **Occurrence:** catalogNumber: A0024; recordedBy: L. Fortini; individualCount: 1; sex: male; lifeStage: adult; occurrenceID: 0FC8C1A7-F71B-5774-A9BF-412F461D9B88; **Taxon:** scientificName: Xylocopa (Xylocopa) violacea (Linnaeus, 1758); order: Hymenoptera; family: Apidae; genus: Xylocopa; subgenus: Xylocopa; specificEpithet: violacea; scientificNameAuthorship: (Linnaeus, 1758); **Location:** country: Italy; countryCode: IT; stateProvince: Roma; locality: Riserva Regionale dell'Appia Antica 1; decimalLatitude: 41.8623941; decimalLongitude: 12.524863; geodeticDatum: WGS84; coordinatePrecision: 0.0002; **Identification:** identifiedBy: L. Fortini; **Event:** eventDate: 2022-07-22; **Record Level:** collectionCode: UR3
- **Type status:** Other material. **Occurrence:** catalogNumber: A0025; recordedBy: L. Fortini; individualCount: 1; sex: male; lifeStage: adult; occurrenceID: 4938CA44-A8B9-56F7-B878-206B3C581F5F; **Taxon:** scientificName: Xylocopa (Xylocopa) violacea (Linnaeus, 1758); order: Hymenoptera; family: Apidae; genus: Xylocopa; subgenus: Xylocopa; specificEpithet: violacea; scientificNameAuthorship: (Linnaeus, 1758); **Location:** country: Italy; countryCode: IT; stateProvince: Roma; locality: Riserva Regionale dell'Appia Antica 2; decimalLatitude: 41.8402564; decimalLongitude: 12.532773; geodeticDatum: WGS84; coordinatePrecision: 0.0002; **Identification:** identifiedBy: L. Fortini; **Event:** eventDate: 2022-07-07; **Record Level:** collectionCode: UR3
- **Type status:** Other material. **Occurrence:** catalogNumber: A0014, A0017, A0022, A0023; recordedBy: L. Fortini; individualCount: 4; sex: 1 male, 3 females; lifeStage: adult; occurrenceID: 36D02EE3-8B72-58CC-A043-1E3ED18C40FD; **Taxon:** scientificName: Xylocopa (Xylocopa) violacea (Linnaeus, 1758); order: Hymenoptera; family: Apidae; genus: Xylocopa; subgenus: Xylocopa; specificEpithet: violacea; scientificNameAuthorship: (Linnaeus, 1758); **Location:** country: Italy; countryCode: IT; stateProvince: Roma; locality: Riserva Naturale dell'Insugherata 1; decimalLatitude: 41.9555045; decimalLongitude: 12.4292321; geodeticDatum: WGS84; coordinatePrecision: 0.0002; **Identification:** identifiedBy: L. Fortini; **Event:** eventDate: 2022-07-30; **Record Level:** collectionCode: UR3
- **Type status:** Other material. **Occurrence:** catalogNumber: A0020; recordedBy: L. Fortini; individualCount: 1; sex: female; lifeStage: adult; occurrenceID: F09DF38C-8DC4-5849-9F46-9A1CC7CB8F9F; **Taxon:** scientificName: Xylocopa (Xylocopa) violacea (Linnaeus, 1758); order: Hymenoptera; family: Apidae; genus: Xylocopa; subgenus: Xylocopa; specificEpithet: violacea; scientificNameAuthorship: (Linnaeus, 1758); **Location:** country: Italy; countryCode: IT; stateProvince: Roma; locality: Riserva Naturale dell'Insugherata 1; decimalLatitude: 41.9555045; decimalLongitude: 12.4292321; geodeticDatum: WGS84; coordinatePrecision: 0.0002; **Identification:** identifiedBy: L. Fortini; **Event:** eventDate: 2022-05-27; **Record Level:** collectionCode: UR3
- **Type status:** Other material. **Occurrence:** catalogNumber: A0013, A0027; recordedBy: L. Fortini; individualCount: 2; sex: 1 male, 1 female; lifeStage: adult; occurrenceID: 1B939261-80BC-5216-8A0F-B59E3AC5CC75; **Taxon:** scientificName: Xylocopa (Xylocopa) violacea (Linnaeus, 1758); order: Hymenoptera; family: Apidae; genus: Xylocopa; subgenus: Xylocopa; specificEpithet: violacea; scientificNameAuthorship: (Linnaeus, 1758); **Location:** country: Italy; countryCode: IT; stateProvince: Roma; locality: Riserva Naturale dell'Insugherata 2; decimalLatitude: 41.9599247; decimalLongitude: 12.433852; geodeticDatum: WGS84; coordinatePrecision: 0.0002; **Identification:** identifiedBy: L. Fortini; **Event:** eventDate: 2022-07-30; **Record Level:** collectionCode: UR3
- **Type status:** Other material. **Occurrence:** catalogNumber: A0018; recordedBy: L. Fortini; individualCount: 1; sex: female; lifeStage: adult; occurrenceID: A0B293BB-494C-56B3-AFA8-7423C39A68FB; **Taxon:** scientificName: Xylocopa (Xylocopa) violacea (Linnaeus, 1758); order: Hymenoptera; family: Apidae; genus: Xylocopa; subgenus: Xylocopa; specificEpithet: violacea; scientificNameAuthorship: (Linnaeus, 1758); **Location:** country: Italy; countryCode: IT; stateProvince: Roma; locality: Riserva Naturale dell'Insugherata 3; decimalLatitude: 41.9644829; decimalLongitude: 12.436101; geodeticDatum: WGS84; coordinatePrecision: 0.0002; **Identification:** identifiedBy: L. Fortini; **Event:** eventDate: 2022-07-30; **Record Level:** collectionCode: UR3
- **Type status:** Other material. **Occurrence:** catalogNumber: A0012; recordedBy: L. Fortini; individualCount: 1; sex: female; lifeStage: adult; occurrenceID: E62582FA-F2C4-5C6A-941E-3CA11AE0193A; **Taxon:** scientificName: Xylocopa (Xylocopa) violacea (Linnaeus, 1758); order: Hymenoptera; family: Apidae; genus: Xylocopa; subgenus: Xylocopa; specificEpithet: violacea; scientificNameAuthorship: (Linnaeus, 1758); **Location:** country: Italy; countryCode: IT; stateProvince: Roma; locality: Riserva Naturale Laurentino-Acqua Acetosa; decimalLatitude: 41.8079275; decimalLongitude: 12.4685548; geodeticDatum: WGS84; coordinatePrecision: 0.0002; **Identification:** identifiedBy: L. Fortini; **Event:** eventDate: 2022-04-12; **Record Level:** collectionCode: UR3
- **Type status:** Other material. **Occurrence:** catalogNumber: A0029; recordedBy: L. Fortini; individualCount: 1; sex: female; lifeStage: adult; occurrenceID: B7A0BAE4-86E3-59E6-8BC0-745EEBE00A24; **Taxon:** scientificName: Xylocopa (Xylocopa) violacea (Linnaeus, 1758); order: Hymenoptera; family: Apidae; genus: Xylocopa; subgenus: Xylocopa; specificEpithet: violacea; scientificNameAuthorship: (Linnaeus, 1758); **Location:** country: Italy; countryCode: IT; stateProvince: Roma; locality: Riserva Naturale Laurentino-Acqua Acetosa; decimalLatitude: 41.8079275; decimalLongitude: 12.4685548; geodeticDatum: WGS84; coordinatePrecision: 0.0002; **Identification:** identifiedBy: L. Fortini; **Event:** eventDate: 2022-09-14; **Record Level:** collectionCode: UR3
- **Type status:** Other material. **Occurrence:** catalogNumber: A0021; recordedBy: L. Fortini; individualCount: 1; sex: female; lifeStage: adult; occurrenceID: 494D724E-4DA9-51C4-BC29-BC0F8CFA3600; **Taxon:** scientificName: Xylocopa (Xylocopa) violacea (Linnaeus, 1758); order: Hymenoptera; family: Apidae; genus: Xylocopa; subgenus: Xylocopa; specificEpithet: violacea; scientificNameAuthorship: (Linnaeus, 1758); **Location:** country: Italy; countryCode: IT; stateProvince: Roma; locality: Riserva Naturale Tenuta dei Massimi 2; decimalLatitude: 41.8316516; decimalLongitude: 12.3999927; geodeticDatum: WGS84; coordinatePrecision: 0.0002; **Identification:** identifiedBy: L. Fortini; **Event:** eventDate: 2022-05-04; **Record Level:** collectionCode: UR3
- **Type status:** Other material. **Occurrence:** catalogNumber: A0015; recordedBy: L. Fortini; individualCount: 1; sex: female; lifeStage: adult; occurrenceID: 1D3A0A0B-3296-5D05-AEE1-2BB2AB91E61B; **Taxon:** scientificName: Xylocopa (Xylocopa) violacea (Linnaeus, 1758); order: Hymenoptera; family: Apidae; genus: Xylocopa; subgenus: Xylocopa; specificEpithet: violacea; scientificNameAuthorship: (Linnaeus, 1758); **Location:** country: Italy; countryCode: IT; stateProvince: Roma; locality: Riserva Naturale Valle dell'Aniene 1; decimalLatitude: 41.9345179; decimalLongitude: 12.5453096; geodeticDatum: WGS84; coordinatePrecision: 0.0002; **Identification:** identifiedBy: L. Fortini; **Event:** eventDate: 2022-04-28; **Record Level:** collectionCode: UR3
- **Type status:** Other material. **Occurrence:** catalogNumber: A0019, A0028; recordedBy: L. Fortini; individualCount: 2; sex: females; lifeStage: adult; occurrenceID: EB0B33FA-8F52-5A5D-ABE2-9A0E42D5B133; **Taxon:** scientificName: Xylocopa (Xylocopa) violacea (Linnaeus, 1758); order: Hymenoptera; family: Apidae; genus: Xylocopa; subgenus: Xylocopa; specificEpithet: violacea; scientificNameAuthorship: (Linnaeus, 1758); **Location:** country: Italy; countryCode: IT; stateProvince: Roma; locality: Riserva Naturale Valle dell'Aniene 2; decimalLatitude: 41.928752; decimalLongitude: 12.5562962; geodeticDatum: WGS84; coordinatePrecision: 0.0002; **Identification:** identifiedBy: L. Fortini; **Event:** eventDate: 2022-07-01; **Record Level:** collectionCode: UR3
- **Type status:** Other material. **Occurrence:** catalogNumber: A2102; recordedBy: L. Fortini; individualCount: 1; sex: male; lifeStage: adult; occurrenceID: 97653AB7-AA59-500C-B38B-FC8E66A7F5B1; **Taxon:** scientificName: Xylocopa (Xylocopa) violacea (Linnaeus, 1758); order: Hymenoptera; family: Apidae; genus: Xylocopa; subgenus: Xylocopa; specificEpithet: violacea; scientificNameAuthorship: (Linnaeus, 1758); **Location:** country: Italy; countryCode: IT; stateProvince: Roma; locality: Riserva Naturale Valle dell'Aniene 1; decimalLatitude: 41.9345179; decimalLongitude: 12.5453096; geodeticDatum: WGS84; coordinatePrecision: 0.0002; **Identification:** identifiedBy: L. Fortini; **Event:** eventDate: 2022-08-03; **Record Level:** collectionCode: UR3
- **Type status:** Other material. **Occurrence:** catalogNumber: A2139; recordedBy: L. Fortini; individualCount: 1; sex: male; lifeStage: adult; occurrenceID: 1CC5EFA5-6F68-53C9-8EA8-B2CD038CD911; **Taxon:** scientificName: Xylocopa (Xylocopa) violacea (Linnaeus, 1758); order: Hymenoptera; family: Apidae; genus: Xylocopa; subgenus: Xylocopa; specificEpithet: violacea; scientificNameAuthorship: (Linnaeus, 1758); **Location:** country: Italy; countryCode: IT; stateProvince: Roma; locality: Riserva Naturale Valle dell'Aniene 2; decimalLatitude: 41.928752; decimalLongitude: 12.5562962; geodeticDatum: WGS84; coordinatePrecision: 0.0002; **Identification:** identifiedBy: L. Fortini; **Event:** eventDate: 2022-08-03; **Record Level:** collectionCode: UR3

### Colletidae Lepeletier, 1841

#### Colletes eous

Morice, 1904

D1B9357D-292E-5831-A2D4-CC82CAE65C73

##### Materials

- **Type status:** Other material. **Occurrence:** catalogNumber: A0676, A0680, A0681, A0682; recordedBy: L. Fortini; individualCount: 4; sex: females; lifeStage: adult; occurrenceID: 073A4FFC-30C8-597D-A6C7-A997727626E4; **Taxon:** scientificName: Colletes (Colletes) eous Morice, 1904; order: Hymenoptera; family: Colletidae; genus: Colletes; subgenus: Colletes; specificEpithet: eous; scientificNameAuthorship: Morice, 1904; **Location:** country: Italy; countryCode: IT; stateProvince: Roma; locality: Riserva Naturale Laurentino-Acqua Acetosa; decimalLatitude: 41.807928; decimalLongitude: 12.468555; geodeticDatum: WGS84; coordinatePrecision: 0.0002; **Identification:** identifiedBy: M. Mei; **Event:** eventDate: 2022-08-21; **Record Level:** collectionCode: UR3

#### Colletes hederae

Schmidt & Westrich, 1993

DE67DD8F-8AB1-5E25-80CF-281A7F22B770

##### Materials

- **Type status:** Other material. **Occurrence:** catalogNumber: A0664; recordedBy: L. Fortini; individualCount: 1; sex: male; lifeStage: adult; occurrenceID: 10940D71-56DA-5219-9701-1698BFB1B128; **Taxon:** scientificName: Colletes (Colletes) hederae Schmidt & Westrich, 1993; order: Hymenoptera; family: Colletidae; genus: Colletes; subgenus: Colletes; specificEpithet: hederae; scientificNameAuthorship: Schmidt & Westrich, 1993; **Location:** country: Italy; countryCode: IT; stateProvince: Roma; locality: Riserva Naturale dell'Acquafredda; decimalLatitude: 41.892841; decimalLongitude: 12.399320; geodeticDatum: WGS84; coordinatePrecision: 0.0002; **Identification:** identifiedBy: M. Mei; **Event:** eventDate: 2022-09-10; **Record Level:** collectionCode: UR3
- **Type status:** Other material. **Occurrence:** catalogNumber: A0665, A0666, A0668, A0669, A0673, A0679; recordedBy: L. Fortini; individualCount: 6; sex: males; lifeStage: adult; occurrenceID: 77BEFB29-43EC-5834-A3C0-14A52D79DBA5; **Taxon:** scientificName: Colletes (Colletes) hederae Schmidt & Westrich, 1993; order: Hymenoptera; family: Colletidae; genus: Colletes; subgenus: Colletes; specificEpithet: hederae; scientificNameAuthorship: Schmidt & Westrich, 1993; **Location:** country: Italy; countryCode: IT; stateProvince: Roma; locality: Riserva Regionale dell'Appia Antica 1; decimalLatitude: 41.862394; decimalLongitude: 12.524863; geodeticDatum: WGS84; coordinatePrecision: 0.0002; **Identification:** identifiedBy: M. Mei; **Event:** eventDate: 2022-09-25; **Record Level:** collectionCode: UR3
- **Type status:** Other material. **Occurrence:** catalogNumber: A0667; recordedBy: L. Fortini; individualCount: 1; sex: male; lifeStage: adult; occurrenceID: 6F08934F-008D-5D77-AD11-975E1F527068; **Taxon:** scientificName: Colletes (Colletes) hederae Schmidt & Westrich, 1993; order: Hymenoptera; family: Colletidae; genus: Colletes; subgenus: Colletes; specificEpithet: hederae; scientificNameAuthorship: Schmidt & Westrich, 1993; **Location:** country: Italy; countryCode: IT; stateProvince: Roma; locality: Riserva Regionale dell'Appia Antica 2; decimalLatitude: 41.840256; decimalLongitude: 12.532773; geodeticDatum: WGS84; coordinatePrecision: 0.0002; **Identification:** identifiedBy: M. Mei; **Event:** eventDate: 2022-07-29; **Record Level:** collectionCode: UR3
- **Type status:** Other material. **Occurrence:** catalogNumber: A0663; recordedBy: L. Fortini; individualCount: 1; sex: male; lifeStage: adult; occurrenceID: FD968AFE-A9AB-5A91-AEAF-579C57D8B76E; **Taxon:** scientificName: Colletes (Colletes) hederae Schmidt & Westrich, 1993; order: Hymenoptera; family: Colletidae; genus: Colletes; subgenus: Colletes; specificEpithet: hederae; scientificNameAuthorship: Schmidt & Westrich, 1993; **Location:** country: Italy; countryCode: IT; stateProvince: Roma; locality: Riserva Naturale Laurentino-Acqua Acetosa; decimalLatitude: 41.807928; decimalLongitude: 12.468555; geodeticDatum: WGS84; coordinatePrecision: 0.0002; **Identification:** identifiedBy: M. Mei; **Event:** eventDate: 2022-09-14; **Record Level:** collectionCode: UR3

#### Colletes marginatus

Smith, 1846

83B7CE56-FC3B-510D-82B2-652DBE9DEE2E

##### Materials

- **Type status:** Other material. **Occurrence:** catalogNumber: A0675; recordedBy: L. Fortini; individualCount: 1; sex: female; lifeStage: adult; occurrenceID: AA90037F-90FA-5A59-AF5A-5ADA8F395444; **Taxon:** scientificName: Colletes (Colletes) marginatus Smith, 1846; order: Hymenoptera; family: Colletidae; genus: Colletes; subgenus: Colletes; specificEpithet: marginatus; scientificNameAuthorship: Smith, 1846; **Location:** country: Italy; countryCode: IT; stateProvince: Roma; locality: Riserva Naturale Laurentino-Acqua Acetosa; decimalLatitude: 41.807928; decimalLongitude: 12.468555; geodeticDatum: WGS84; coordinatePrecision: 0.0002; **Identification:** identifiedBy: M. Mei; **Event:** eventDate: 2022-08-21; **Record Level:** collectionCode: UR3

#### Colletes mlokossewiczi

Radoszkowski, 1891

20B0F6FD-78D2-5BC3-9FFC-35124EA2DD1F

##### Materials

- **Type status:** Other material. **Occurrence:** catalogNumber: A0674; recordedBy: L. Fortini; individualCount: 1; sex: male; lifeStage: adult; occurrenceID: FACBD383-B819-502D-AFAB-44E393DF6D7E; **Taxon:** scientificName: Colletes (Colletes) mlokossewiczi Radoszkowski, 1891; order: Hymenoptera; family: Colletidae; genus: Colletes; subgenus: Colletes; specificEpithet: mlokossewiczi; scientificNameAuthorship: Radoszkowski, 1891; **Location:** country: Italy; countryCode: IT; stateProvince: Roma; locality: Riserva Naturale Laurentino-Acqua Acetosa; decimalLatitude: 41.807928; decimalLongitude: 12.468555; geodeticDatum: WGS84; coordinatePrecision: 0.0002; **Identification:** identifiedBy: M. Mei; **Event:** eventDate: 2022-08-21; **Record Level:** collectionCode: UR3
- **Type status:** Other material. **Occurrence:** catalogNumber: A0677; recordedBy: L. Fortini; individualCount: 1; sex: male; lifeStage: adult; occurrenceID: AF3F2283-B803-51BA-9ED0-FFC3E3DA2B82; **Taxon:** scientificName: Colletes (Colletes) mlokossewiczi Radoszkowski, 1891; order: Hymenoptera; family: Colletidae; genus: Colletes; subgenus: Colletes; specificEpithet: mlokossewiczi; scientificNameAuthorship: Radoszkowski, 1891; **Location:** country: Italy; countryCode: IT; stateProvince: Roma; locality: Riserva Naturale dell'Insugherata 1; decimalLatitude: 41.955505; decimalLongitude: 12.429232; geodeticDatum: WGS84; coordinatePrecision: 0.0002; **Identification:** identifiedBy: M. Mei; **Event:** eventDate: 2022-07-30; **Record Level:** collectionCode: UR3

#### Colletes similis

Schenck, 1853

83BB382E-53E1-5AD4-B3E8-DD572255F4BD

##### Materials

- **Type status:** Other material. **Occurrence:** catalogNumber: A0670; recordedBy: L. Fortini; individualCount: 1; sex: male; lifeStage: adult; occurrenceID: F34D5E03-4B96-5866-92E0-1BC008CDB245; **Taxon:** scientificName: Colletes (Colletes) similis Schenck, 1853; order: Hymenoptera; family: Colletidae; genus: Colletes; subgenus: Colletes; specificEpithet: similis; scientificNameAuthorship: Schenck, 1853; **Location:** country: Italy; countryCode: IT; stateProvince: Roma; locality: Riserva Naturale dell'Acquafredda; decimalLatitude: 41.892841; decimalLongitude: 12.399320; geodeticDatum: WGS84; coordinatePrecision: 0.0002; **Identification:** identifiedBy: M. Mei; **Event:** eventDate: 2022-06-10; **Record Level:** collectionCode: UR3
- **Type status:** Other material. **Occurrence:** catalogNumber: A0671; recordedBy: L. Fortini; individualCount: 1; sex: male; lifeStage: adult; occurrenceID: 4A1E36C2-EA23-5E31-B2E1-57F15F4802B0; **Taxon:** scientificName: Colletes (Colletes) similis Schenck, 1853; order: Hymenoptera; family: Colletidae; genus: Colletes; subgenus: Colletes; specificEpithet: similis; scientificNameAuthorship: Schenck, 1853; **Location:** country: Italy; countryCode: IT; stateProvince: Roma; locality: Riserva Regionale dell'Appia Antica 2; decimalLatitude: 41.840256; decimalLongitude: 12.532773; geodeticDatum: WGS84; coordinatePrecision: 0.0002; **Identification:** identifiedBy: M. Mei; **Event:** eventDate: 2022-05-24; **Record Level:** collectionCode: UR3
- **Type status:** Other material. **Occurrence:** catalogNumber: A0672; recordedBy: L. Fortini; individualCount: 1; sex: male; lifeStage: adult; occurrenceID: F34BBB7C-94CD-55BD-9289-9F674ACEDBCD; **Taxon:** scientificName: Colletes (Colletes) similis Schenck, 1853; order: Hymenoptera; family: Colletidae; genus: Colletes; subgenus: Colletes; specificEpithet: similis; scientificNameAuthorship: Schenck, 1853; **Location:** country: Italy; countryCode: IT; stateProvince: Roma; locality: Riserva Naturale Tenuta dei Massimi 1; decimalLatitude: 41.8532859; decimalLongitude: 12.3842322; geodeticDatum: WGS84; coordinatePrecision: 0.0002; **Identification:** identifiedBy: M. Mei; **Event:** eventDate: 2022-09-28; **Record Level:** collectionCode: UR3
- **Type status:** Other material. **Occurrence:** catalogNumber: A0678; recordedBy: L. Fortini; individualCount: 1; sex: male; lifeStage: adult; occurrenceID: D062808E-06BA-5F09-9381-325E1AE4AB4E; **Taxon:** scientificName: Colletes (Colletes) similis Schenck, 1853; order: Hymenoptera; family: Colletidae; genus: Colletes; subgenus: Colletes; specificEpithet: similis; scientificNameAuthorship: Schenck, 1853; **Location:** country: Italy; countryCode: IT; stateProvince: Roma; locality: Riserva Naturale Tenuta dei Massimi 2; decimalLatitude: 41.831652; decimalLongitude: 12.399993; geodeticDatum: WGS84; coordinatePrecision: 0.0002; **Identification:** identifiedBy: M. Mei; **Event:** eventDate: 2022-06-01; **Record Level:** collectionCode: UR3

#### Hylaeus angustatus

(Schenck, 1861)

B93E3FF1-4C6C-5E11-AA8E-AFB203C5D243

##### Materials

- **Type status:** Other material. **Occurrence:** catalogNumber: A0525; recordedBy: L. Fortini; individualCount: 1; sex: male; lifeStage: adult; occurrenceID: 7D17E395-D18A-5BC5-AB74-872F31D8E863; **Taxon:** scientificName: Hylaeus (Hylaeus) angustatus (Schenck, 1861); order: Hymenoptera; family: Colletidae; genus: Hylaeus; subgenus: Hylaeus; specificEpithet: angustatus; scientificNameAuthorship: (Schenck, 1861); **Location:** country: Italy; countryCode: IT; stateProvince: Roma; locality: Riserva Regionale dell'Appia Antica 1; decimalLatitude: 41.862394; decimalLongitude: 12.524863; geodeticDatum: WGS84; coordinatePrecision: 0.0002; **Identification:** identifiedBy: M. Mei; **Event:** eventDate: 2022-05-10; **Record Level:** collectionCode: UR3
- **Type status:** Other material. **Occurrence:** catalogNumber: A0514; recordedBy: L. Fortini; individualCount: 1; sex: female; lifeStage: adult; occurrenceID: 8A90F9ED-9606-5AB1-9D58-67382D6AD32A; **Taxon:** scientificName: Hylaeus (Hylaeus) angustatus (Schenck, 1861); order: Hymenoptera; family: Colletidae; genus: Hylaeus; subgenus: Hylaeus; specificEpithet: angustatus; scientificNameAuthorship: (Schenck, 1861); **Location:** country: Italy; countryCode: IT; stateProvince: Roma; locality: Riserva Naturale Valle dei Casali 1; decimalLatitude: 41.8710627; decimalLongitude: 12.4336809; geodeticDatum: WGS84; coordinatePrecision: 0.0002; **Identification:** identifiedBy: M. Mei; **Event:** eventDate: 2022-05-14; **Record Level:** collectionCode: UR3
- **Type status:** Other material. **Occurrence:** catalogNumber: A0521, A0546, A0551, A0554, A0563, A0568; recordedBy: L. Fortini; individualCount: 6; sex: 1 male, 5 females; lifeStage: adult; occurrenceID: AF2EBE95-2807-5147-B9B0-72998677D92C; **Taxon:** scientificName: Hylaeus (Hylaeus) angustatus (Schenck, 1861); order: Hymenoptera; family: Colletidae; genus: Hylaeus; subgenus: Hylaeus; specificEpithet: angustatus; scientificNameAuthorship: (Schenck, 1861); **Location:** country: Italy; countryCode: IT; stateProvince: Roma; locality: Riserva Naturale dell'Insugherata 1; decimalLatitude: 41.955505; decimalLongitude: 12.429232; geodeticDatum: WGS84; coordinatePrecision: 0.0002; **Identification:** identifiedBy: M. Mei; **Event:** eventDate: 2022-07-30; **Record Level:** collectionCode: UR3
- **Type status:** Other material. **Occurrence:** catalogNumber: A0600; recordedBy: L. Fortini; individualCount: 1; sex: male; lifeStage: adult; occurrenceID: 9D27299E-881A-595B-97FA-BBB153846A10; **Taxon:** scientificName: Hylaeus (Hylaeus) angustatus (Schenck, 1861); order: Hymenoptera; family: Colletidae; genus: Hylaeus; subgenus: Hylaeus; specificEpithet: angustatus; scientificNameAuthorship: (Schenck, 1861); **Location:** country: Italy; countryCode: IT; stateProvince: Roma; locality: Riserva Regionale dell'Appia Antica 2; decimalLatitude: 41.840256; decimalLongitude: 12.532773; geodeticDatum: WGS84; coordinatePrecision: 0.0002; **Identification:** identifiedBy: M. Mei; **Event:** eventDate: 2022-08-29; **Record Level:** collectionCode: UR3

#### Hylaeus brevicornis

Nylander, 1852

23E54EC0-9D25-5899-A9D8-F5DF2F1D0952

##### Materials

- **Type status:** Other material. **Occurrence:** catalogNumber: A0517; recordedBy: L. Fortini; individualCount: 1; sex: male; lifeStage: adult; occurrenceID: B514E4C7-8628-5F03-AFC1-1BAB76C6FD92; **Taxon:** scientificName: Hylaeus (Dentigera) brevicornis Nylander, 1852; order: Hymenoptera; family: Colletidae; genus: Hylaeus; subgenus: Dentigera; specificEpithet: brevicornis; scientificNameAuthorship: Nylander, 1852; **Location:** country: Italy; countryCode: IT; stateProvince: Roma; locality: Riserva Regionale dell'Appia Antica 1; decimalLatitude: 41.862394; decimalLongitude: 12.524863; geodeticDatum: WGS84; coordinatePrecision: 0.0002; **Identification:** identifiedBy: M. Mei; **Event:** eventDate: 2022-05-10; **Record Level:** collectionCode: UR3
- **Type status:** Other material. **Occurrence:** catalogNumber: A0496, A0584; recordedBy: L. Fortini; individualCount: 2; sex: females; lifeStage: adult; occurrenceID: A80AD53E-6C2F-53AA-93F0-78E0AE3419DB; **Taxon:** scientificName: Hylaeus (Dentigera) brevicornis Nylander, 1852; order: Hymenoptera; family: Colletidae; genus: Hylaeus; subgenus: Dentigera; specificEpithet: brevicornis; scientificNameAuthorship: Nylander, 1853; **Location:** country: Italy; countryCode: IT; stateProvince: Roma; locality: Riserva Regionale dell'Appia Antica 2; decimalLatitude: 41.840256; decimalLongitude: 12.532773; geodeticDatum: WGS84; coordinatePrecision: 0.0002; **Identification:** identifiedBy: M. Mei; **Event:** eventDate: 2022-05-24; **Record Level:** collectionCode: UR3
- **Type status:** Other material. **Occurrence:** catalogNumber: A0565; recordedBy: L. Fortini; individualCount: 1; sex: female; lifeStage: adult; occurrenceID: 63499189-9EFC-5EEB-978B-4B58A28ABEB5; **Taxon:** scientificName: Hylaeus (Dentigera) brevicornis Nylander, 1852; order: Hymenoptera; family: Colletidae; genus: Hylaeus; subgenus: Dentigera; specificEpithet: brevicornis; scientificNameAuthorship: Nylander, 1854; **Location:** country: Italy; countryCode: IT; stateProvince: Roma; locality: Riserva Naturale Tenuta dei Massimi 2; decimalLatitude: 41.831652; decimalLongitude: 12.399993; geodeticDatum: WGS84; coordinatePrecision: 0.0002; **Identification:** identifiedBy: M. Mei; **Event:** eventDate: 2022-06-01; **Record Level:** collectionCode: UR3
- **Type status:** Other material. **Occurrence:** catalogNumber: A0513; recordedBy: L. Fortini; individualCount: 1; sex: female; lifeStage: adult; occurrenceID: 7AFDB2AB-2130-58EC-9D93-DFFD825F0059; **Taxon:** scientificName: Hylaeus (Dentigera) brevicornis Nylander, 1852; order: Hymenoptera; family: Colletidae; genus: Hylaeus; subgenus: Dentigera; specificEpithet: brevicornis; scientificNameAuthorship: Nylander, 1855; **Location:** country: Italy; countryCode: IT; stateProvince: Roma; locality: Riserva Naturale dell'Acquafredda; decimalLatitude: 41.892841; decimalLongitude: 12.399320; geodeticDatum: WGS84; coordinatePrecision: 0.0002; **Identification:** identifiedBy: M. Mei; **Event:** eventDate: 2022-06-10; **Record Level:** collectionCode: UR3
- **Type status:** Other material. **Occurrence:** catalogNumber: A0533; recordedBy: L. Fortini; individualCount: 1; sex: female; lifeStage: adult; occurrenceID: B7FE036A-82EE-53F7-9622-51333FB6CF18; **Taxon:** scientificName: Hylaeus (Dentigera) brevicornis Nylander, 1852; order: Hymenoptera; family: Colletidae; genus: Hylaeus; subgenus: Dentigera; specificEpithet: brevicornis; scientificNameAuthorship: Nylander, 1856; **Location:** country: Italy; countryCode: IT; stateProvince: Roma; locality: Riserva Naturale Valle dei Casali 1; decimalLatitude: 41.8710627; decimalLongitude: 12.4336809; geodeticDatum: WGS84; coordinatePrecision: 0.0002; **Identification:** identifiedBy: M. Mei; **Event:** eventDate: 2022-06-18; **Record Level:** collectionCode: UR3
- **Type status:** Other material. **Occurrence:** catalogNumber: A0550; recordedBy: L. Fortini; individualCount: 1; sex: female; lifeStage: adult; occurrenceID: 760CF177-5E60-542A-9246-9262BF7AFD45; **Taxon:** scientificName: Hylaeus (Dentigera) brevicornis Nylander, 1852; order: Hymenoptera; family: Colletidae; genus: Hylaeus; subgenus: Dentigera; specificEpithet: brevicornis; scientificNameAuthorship: Nylander, 1857; **Location:** country: Italy; countryCode: IT; stateProvince: Roma; locality: Riserva Naturale dell'Acquafredda; decimalLatitude: 41.892841; decimalLongitude: 12.399320; geodeticDatum: WGS84; coordinatePrecision: 0.0002; **Identification:** identifiedBy: M. Mei; **Event:** eventDate: 2022-07-12; **Record Level:** collectionCode: UR3
- **Type status:** Other material. **Occurrence:** catalogNumber: A0530, A0549, A0562; recordedBy: L. Fortini; individualCount: 3; sex: males; lifeStage: adult; occurrenceID: B1E29B85-7C86-5C59-9760-3CA483AFE7D7; **Taxon:** scientificName: Hylaeus (Dentigera) brevicornis Nylander, 1852; order: Hymenoptera; family: Colletidae; genus: Hylaeus; subgenus: Dentigera; specificEpithet: brevicornis; scientificNameAuthorship: Nylander, 1858; **Location:** country: Italy; countryCode: IT; stateProvince: Roma; locality: Riserva Naturale Valle dei Casali 1; decimalLatitude: 41.8710627; decimalLongitude: 12.4336809; geodeticDatum: WGS84; coordinatePrecision: 0.0002; **Identification:** identifiedBy: M. Mei; **Event:** eventDate: 2022-07-13; **Record Level:** collectionCode: UR3
- **Type status:** Other material. **Occurrence:** catalogNumber: A0564; recordedBy: L. Fortini; individualCount: 1; sex: male; lifeStage: adult; occurrenceID: 02769FAB-54FB-5903-B0DB-3934C10CE12A; **Taxon:** scientificName: Hylaeus (Dentigera) brevicornis Nylander, 1852; order: Hymenoptera; family: Colletidae; genus: Hylaeus; subgenus: Dentigera; specificEpithet: brevicornis; scientificNameAuthorship: Nylander, 1859; **Location:** country: Italy; countryCode: IT; stateProvince: Roma; locality: Riserva Regionale dell'Appia Antica 2; decimalLatitude: 41.840256; decimalLongitude: 12.532773; geodeticDatum: WGS84; coordinatePrecision: 0.0002; **Identification:** identifiedBy: M. Mei; **Event:** eventDate: 2022-08-29; **Record Level:** collectionCode: UR3
- **Type status:** Other material. **Occurrence:** catalogNumber: A0597; recordedBy: L. Fortini; individualCount: 1; sex: female; lifeStage: adult; occurrenceID: 61F4D054-1242-5F66-9C17-A89F3791FD2F; **Taxon:** scientificName: Hylaeus (Dentigera) brevicornis Nylander, 1852; order: Hymenoptera; family: Colletidae; genus: Hylaeus; subgenus: Dentigera; specificEpithet: brevicornis; scientificNameAuthorship: Nylander, 1860; **Location:** country: Italy; countryCode: IT; stateProvince: Roma; locality: Riserva Naturale Tenuta dei Massimi 2; decimalLatitude: 41.831652; decimalLongitude: 12.399993; geodeticDatum: WGS84; coordinatePrecision: 0.0002; **Identification:** identifiedBy: M. Mei; **Event:** eventDate: 2022-09-28; **Record Level:** collectionCode: UR3

#### Hylaeus clypearis

(Schenck, 1853)

E643FD7B-CC38-578D-AC6D-F166F42F7508

##### Materials

- **Type status:** Other material. **Occurrence:** catalogNumber: A0491; recordedBy: L. Fortini; individualCount: 1; sex: male; lifeStage: adult; occurrenceID: 9EB75FEE-9966-5681-A6C3-50623E777687; **Taxon:** scientificName: Hylaeus (Paraprosopis) clypearis (Schenck, 1853); order: Hymenoptera; family: Colletidae; genus: Hylaeus; subgenus: Paraprosopis; specificEpithet: clypearis; scientificNameAuthorship: (Schenck, 1853); **Location:** country: Italy; countryCode: IT; stateProvince: Roma; locality: Riserva Naturale Tenuta dei Massimi 2; decimalLatitude: 41.831652; decimalLongitude: 12.399993; geodeticDatum: WGS84; coordinatePrecision: 0.0002; **Identification:** identifiedBy: M. Mei; **Event:** eventDate: 2022-06-01; **Record Level:** collectionCode: UR3
- **Type status:** Other material. **Occurrence:** catalogNumber: A0544; recordedBy: L. Fortini; individualCount: 1; sex: male; lifeStage: adult; occurrenceID: 0FEBDEF0-BA0E-5325-94D3-1E3B78D38E20; **Taxon:** scientificName: Hylaeus (Paraprosopis) clypearis (Schenck, 1853); order: Hymenoptera; family: Colletidae; genus: Hylaeus; subgenus: Paraprosopis; specificEpithet: clypearis; scientificNameAuthorship: (Schenck, 1853); **Location:** country: Italy; countryCode: IT; stateProvince: Roma; locality: Riserva Naturale Tenuta dei Massimi 2; decimalLatitude: 41.831652; decimalLongitude: 12.399993; geodeticDatum: WGS84; coordinatePrecision: 0.0002; **Identification:** identifiedBy: M. Mei; **Event:** eventDate: 2022-06-27; **Record Level:** collectionCode: UR3
- **Type status:** Other material. **Occurrence:** catalogNumber: A0540, A0543; recordedBy: L. Fortini; individualCount: 2; sex: males; lifeStage: adult; occurrenceID: 502B15A1-9063-52BD-8EB4-6BEE211A2538; **Taxon:** scientificName: Hylaeus (Paraprosopis) clypearis (Schenck, 1853); order: Hymenoptera; family: Colletidae; genus: Hylaeus; subgenus: Paraprosopis; specificEpithet: clypearis; scientificNameAuthorship: (Schenck, 1853); **Location:** country: Italy; countryCode: IT; stateProvince: Roma; locality: Riserva Naturale dell'Acquafredda; decimalLatitude: 41.892841; decimalLongitude: 12.399320; geodeticDatum: WGS84; coordinatePrecision: 0.0002; **Identification:** identifiedBy: M. Mei; **Event:** eventDate: 2022-07-12; **Record Level:** collectionCode: UR3
- **Type status:** Other material. **Occurrence:** catalogNumber: A0559; recordedBy: L. Fortini; individualCount: 1; sex: male; lifeStage: adult; occurrenceID: B2214F78-09BD-5ACE-A94C-BC8DCB57EC58; **Taxon:** scientificName: Hylaeus (Paraprosopis) clypearis (Schenck, 1853); order: Hymenoptera; family: Colletidae; genus: Hylaeus; subgenus: Paraprosopis; specificEpithet: clypearis; scientificNameAuthorship: (Schenck, 1853); **Location:** country: Italy; countryCode: IT; stateProvince: Roma; locality: Riserva Naturale Valle dei Casali 1; decimalLatitude: 41.8710627; decimalLongitude: 12.4336809; geodeticDatum: WGS84; coordinatePrecision: 0.0002; **Identification:** identifiedBy: M. Mei; **Event:** eventDate: 2022-07-13; **Record Level:** collectionCode: UR3
- **Type status:** Other material. **Occurrence:** catalogNumber: A0570, A0571, A0573, A0576; recordedBy: L. Fortini; individualCount: 4; sex: females; lifeStage: adult; occurrenceID: 299E454B-46DE-502F-9E26-FBC349800B9E; **Taxon:** scientificName: Hylaeus (Paraprosopis) clypearis (Schenck, 1853); order: Hymenoptera; family: Colletidae; genus: Hylaeus; subgenus: Paraprosopis; specificEpithet: clypearis; scientificNameAuthorship: (Schenck, 1853); **Location:** country: Italy; countryCode: IT; stateProvince: Roma; locality: Riserva Naturale Laurentino-Acqua Acetosa; decimalLatitude: 41.807928; decimalLongitude: 12.468555; geodeticDatum: WGS84; coordinatePrecision: 0.0002; **Identification:** identifiedBy: M. Mei; **Event:** eventDate: 2022-07-17; **Record Level:** collectionCode: UR3
- **Type status:** Other material. **Occurrence:** catalogNumber: A2114, A2115; recordedBy: L. Fortini; individualCount: 2; sex: males; lifeStage: adult; occurrenceID: 871A421E-8FA6-5093-8966-9CCC1679FC8B; **Taxon:** scientificName: Hylaeus (Paraprosopis) clypearis (Schenck, 1853); order: Hymenoptera; family: Colletidae; genus: Hylaeus; subgenus: Paraprosopis; specificEpithet: clypearis; scientificNameAuthorship: (Schenck, 1853); **Location:** country: Italy; countryCode: IT; stateProvince: Roma; locality: Riserva Naturale Valle dell'Aniene 2; decimalLatitude: 41.928752; decimalLongitude: 12.5562962; geodeticDatum: WGS84; coordinatePrecision: 0.0002; **Identification:** identifiedBy: M. Mei; **Event:** eventDate: 2022-08-03; **Record Level:** collectionCode: UR3
- **Type status:** Other material. **Occurrence:** catalogNumber: A2048, A2049; recordedBy: L. Fortini; individualCount: 2; sex: males; lifeStage: adult; occurrenceID: 13049622-7611-5C0D-B7EB-C7942D5ED3C8; **Taxon:** scientificName: Hylaeus (Paraprosopis) clypearis (Schenck, 1853); order: Hymenoptera; family: Colletidae; genus: Hylaeus; subgenus: Paraprosopis; specificEpithet: clypearis; scientificNameAuthorship: (Schenck, 1853); **Location:** country: Italy; countryCode: IT; stateProvince: Roma; locality: Riserva Naturale Valle dei Casali 1; decimalLatitude: 41.8710627; decimalLongitude: 12.4336809; geodeticDatum: WGS84; coordinatePrecision: 0.0002; **Identification:** identifiedBy: M. Mei; **Event:** eventDate: 2022-08-19; **Record Level:** collectionCode: UR3
- **Type status:** Other material. **Occurrence:** catalogNumber: A0599; recordedBy: L. Fortini; individualCount: 1; sex: female; lifeStage: adult; occurrenceID: 99CDCD53-0BA9-57F5-925C-7CDD623A0D5F; **Taxon:** scientificName: Hylaeus (Paraprosopis) clypearis (Schenck, 1853); order: Hymenoptera; family: Colletidae; genus: Hylaeus; subgenus: Paraprosopis; specificEpithet: clypearis; scientificNameAuthorship: (Schenck, 1853); **Location:** country: Italy; countryCode: IT; stateProvince: Roma; locality: Riserva Naturale Tenuta dei Massimi 1; decimalLatitude: 41.8532859; decimalLongitude: 12.3842322; geodeticDatum: WGS84; coordinatePrecision: 0.0002; **Identification:** identifiedBy: M. Mei; **Event:** eventDate: 2022-08-27; **Record Level:** collectionCode: UR3
- **Type status:** Other material. **Occurrence:** catalogNumber: A0497, A0498, A0556, A0558, A0560, A0566, A0575, A0585, A0586, A0594, A0595, A0596; recordedBy: L. Fortini; individualCount: 12; sex: 7 males, 5 females; lifeStage: adult; occurrenceID: 7DC5C4A1-B722-51AF-8445-49014B298687; **Taxon:** scientificName: Hylaeus (Paraprosopis) clypearis (Schenck, 1853); order: Hymenoptera; family: Colletidae; genus: Hylaeus; subgenus: Paraprosopis; specificEpithet: clypearis; scientificNameAuthorship: (Schenck, 1853); **Location:** country: Italy; countryCode: IT; stateProvince: Roma; locality: Riserva Regionale dell'Appia Antica 2; decimalLatitude: 41.840256; decimalLongitude: 12.532773; geodeticDatum: WGS84; coordinatePrecision: 0.0002; **Identification:** identifiedBy: M. Mei; **Event:** eventDate: 2022-08-29; **Record Level:** collectionCode: UR3

#### Hylaeus communis

Nylander, 1852

60BB1E1C-B032-5EF9-B6B9-B954911980E4

##### Materials

- **Type status:** Other material. **Occurrence:** catalogNumber: A0494, A0505; recordedBy: L. Fortini; individualCount: 2; sex: females; lifeStage: adult; occurrenceID: AEC5CE2D-FE9A-5260-A988-81919EBEFE7B; **Taxon:** scientificName: Hylaeus (Hylaeus) communis Nylander, 1852; order: Hymenoptera; family: Colletidae; genus: Hylaeus; subgenus: Hylaeus; specificEpithet: communis; scientificNameAuthorship: Nylander, 1852; **Location:** country: Italy; countryCode: IT; stateProvince: Roma; locality: Riserva Naturale Valle dei Casali 1; decimalLatitude: 41.8710627; decimalLongitude: 12.4336809; geodeticDatum: WGS84; coordinatePrecision: 0.0002; **Identification:** identifiedBy: M. Mei; **Event:** eventDate: 2022-05-14; **Record Level:** collectionCode: UR3
- **Type status:** Other material. **Occurrence:** catalogNumber: A0507; recordedBy: L. Fortini; individualCount: 1; sex: female; lifeStage: adult; occurrenceID: 31E1363B-B150-5CD7-AE34-29A5C8131144; **Taxon:** scientificName: Hylaeus (Hylaeus) communis Nylander, 1852; order: Hymenoptera; family: Colletidae; genus: Hylaeus; subgenus: Hylaeus; specificEpithet: communis; scientificNameAuthorship: Nylander, 1852; **Location:** country: Italy; countryCode: IT; stateProvince: Roma; locality: Riserva Naturale di Monte Mario; decimalLatitude: 41.938622; decimalLongitude: 12.454622; geodeticDatum: WGS84; coordinatePrecision: 0.0002; **Identification:** identifiedBy: M. Mei; **Event:** eventDate: 2022-05-20; **Record Level:** collectionCode: UR3
- **Type status:** Other material. **Occurrence:** catalogNumber: A0490, A0500, A0552, A0553, A0574, A0581, A0582; recordedBy: L. Fortini; individualCount: 7; sex: males; lifeStage: adult; occurrenceID: FFF7633B-D3C9-58AA-A4F9-50BFABA1829C; **Taxon:** scientificName: Hylaeus (Hylaeus) communis Nylander, 1852; order: Hymenoptera; family: Colletidae; genus: Hylaeus; subgenus: Hylaeus; specificEpithet: communis; scientificNameAuthorship: Nylander, 1852; **Location:** country: Italy; countryCode: IT; stateProvince: Roma; locality: Riserva Naturale dell'Insugherata 1; decimalLatitude: 41.955505; decimalLongitude: 12.429232; geodeticDatum: WGS84; coordinatePrecision: 0.0002; **Identification:** identifiedBy: M. Mei; **Event:** eventDate: 2022-07-30; **Record Level:** collectionCode: UR3
- **Type status:** Other material. **Occurrence:** catalogNumber: A0488, A0537; recordedBy: L. Fortini; individualCount: 2; sex: males; lifeStage: adult; occurrenceID: 034E57B7-FD2A-5688-985E-595EE366B9D2; **Taxon:** scientificName: Hylaeus (Hylaeus) communis Nylander, 1852; order: Hymenoptera; family: Colletidae; genus: Hylaeus; subgenus: Hylaeus; specificEpithet: communis; scientificNameAuthorship: Nylander, 1852; **Location:** country: Italy; countryCode: IT; stateProvince: Roma; locality: Riserva Regionale dell'Appia Antica 2; decimalLatitude: 41.840256; decimalLongitude: 12.532773; geodeticDatum: WGS84; coordinatePrecision: 0.0002; **Identification:** identifiedBy: M. Mei; **Event:** eventDate: 2022-08-29; **Record Level:** collectionCode: UR3

#### Hylaeus cornutus

Curtis, 1831

73739934-1ED7-556A-BD3F-D16C52301853

##### Materials

- **Type status:** Other material. **Occurrence:** catalogNumber: A0527; recordedBy: L. Fortini; individualCount: 1; sex: male; lifeStage: adult; occurrenceID: F688D361-92C5-597D-828D-A68E3F4C6575; **Taxon:** scientificName: Hylaeus (Abrupta) cornutus Curtis, 1831; order: Hymenoptera; family: Colletidae; genus: Hylaeus; subgenus: Abrupta; specificEpithet: cornutus; scientificNameAuthorship: Curtis, 1831; **Location:** country: Italy; countryCode: IT; stateProvince: Roma; locality: Riserva Naturale Laurentino-Acqua Acetosa; decimalLatitude: 41.807928; decimalLongitude: 12.468555; geodeticDatum: WGS84; coordinatePrecision: 0.0002; **Identification:** identifiedBy: M. Mei; **Event:** eventDate: 2022-07-17; **Record Level:** collectionCode: UR3

#### Hylaeus difformis

(Eversmann, 1852)

3B1B6B64-6D39-5DE1-88CB-65576E365AB2

##### Materials

- **Type status:** Other material. **Occurrence:** catalogNumber: A0524; recordedBy: L. Fortini; individualCount: 1; sex: female; lifeStage: adult; occurrenceID: 6974954B-B9F5-59CE-B29D-A5E1304EAA62; **Taxon:** scientificName: Hylaeus (Hylaeus) difformis (Eversmann, 1852); order: Hymenoptera; family: Colletidae; genus: Hylaeus; subgenus: Hylaeus; specificEpithet: difformis; scientificNameAuthorship: (Eversmann, 1852); **Location:** country: Italy; countryCode: IT; stateProvince: Roma; locality: Riserva Naturale Valle dell'Aniene 2; decimalLatitude: 41.928752; decimalLongitude: 12.5562962; geodeticDatum: WGS84; coordinatePrecision: 0.0002; **Identification:** identifiedBy: M. Mei; **Event:** eventDate: 2022-06-05; **Record Level:** collectionCode: UR3
- **Type status:** Other material. **Occurrence:** catalogNumber: A0579; recordedBy: L. Fortini; individualCount: 1; sex: female; lifeStage: adult; occurrenceID: 50788FAE-D7F8-51A2-AFAA-8B0020F9BBA3; **Taxon:** scientificName: Hylaeus (Hylaeus) difformis (Eversmann, 1852); order: Hymenoptera; family: Colletidae; genus: Hylaeus; subgenus: Hylaeus; specificEpithet: difformis; scientificNameAuthorship: (Eversmann, 1852); **Location:** country: Italy; countryCode: IT; stateProvince: Roma; locality: Riserva Naturale dell'Insugherata 3; decimalLatitude: 41.964483; decimalLongitude: 12.436101; geodeticDatum: WGS84; coordinatePrecision: 0.0002; **Identification:** identifiedBy: M. Mei; **Event:** eventDate: 2022-07-30; **Record Level:** collectionCode: UR3

#### Hylaeus hyalinatus

Smith, 1842

F19DBA4E-20B6-5A6D-9FB5-747C3D75C97A

##### Materials

- **Type status:** Other material. **Occurrence:** catalogNumber: A0547; recordedBy: L. Fortini; individualCount: 1; sex: female; lifeStage: adult; occurrenceID: 64C5495B-ECB7-592B-A5A4-519131F76C17; **Taxon:** scientificName: Hylaeus (Spatulariella) hyalinatus Smith, 1842; order: Hymenoptera; family: Colletidae; genus: Hylaeus; subgenus: Spatulariella; specificEpithet: hyalinatus; scientificNameAuthorship: Smith, 1842; **Location:** country: Italy; countryCode: IT; stateProvince: Roma; locality: Riserva Regionale dell'Appia Antica 2; decimalLatitude: 41.840256; decimalLongitude: 12.532773; geodeticDatum: WGS84; coordinatePrecision: 0.0002; **Identification:** identifiedBy: M. Mei; **Event:** eventDate: 2022-08-29; **Record Level:** collectionCode: UR3

#### Hylaeus intermedius

Förster, 1871

EC12CB9A-02F5-55C8-A3FD-33F0E7E0CA84

##### Materials

- **Type status:** Other material. **Occurrence:** catalogNumber: A0590; recordedBy: L. Fortini; individualCount: 1; sex: female; lifeStage: adult; occurrenceID: ED46D71A-29BE-5617-9F7B-E84A031C4E76; **Taxon:** scientificName: Hylaeus (Spatulariella) hyperpunctatus (Strand, 1909); order: Hymenoptera; family: Colletidae; genus: Hylaeus; subgenus: Spatulariella; specificEpithet: hyperpunctatus; scientificNameAuthorship: (Strand, 1909); **Location:** country: Italy; countryCode: IT; stateProvince: Roma; locality: Riserva Naturale Laurentino-Acqua Acetosa; decimalLatitude: 41.807928; decimalLongitude: 12.468555; geodeticDatum: WGS84; coordinatePrecision: 0.0002; **Identification:** identifiedBy: M. Mei; **Event:** eventDate: 2022-09-14; **Record Level:** collectionCode: UR3
- **Type status:** Other material. **Occurrence:** catalogNumber: A0541; recordedBy: L. Fortini; individualCount: 1; sex: male; lifeStage: adult; occurrenceID: 433B79EC-79E9-587E-867B-2FD4F9CECAD9; **Taxon:** scientificName: Hylaeus (Dentigera) intermedius Förster, 1871; order: Hymenoptera; family: Colletidae; genus: Hylaeus; subgenus: Dentigera; specificEpithet: intermedius; scientificNameAuthorship: Förster, 1871; **Location:** country: Italy; countryCode: IT; stateProvince: Roma; locality: Riserva Naturale dell'Acquafredda; decimalLatitude: 41.892841; decimalLongitude: 12.399320; geodeticDatum: WGS84; coordinatePrecision: 0.0002; **Identification:** identifiedBy: M. Mei; **Event:** eventDate: 2022-07-12; **Record Level:** collectionCode: UR3

#### Hylaeus punctatus

(Brullé, 1832

8FE93143-753E-535B-AB74-BF467941F46E

##### Materials

- **Type status:** Other material. **Occurrence:** catalogNumber: A0536; recordedBy: L. Fortini; individualCount: 1; sex: female; lifeStage: adult; occurrenceID: CD062032-18B5-5170-B3C3-43C4AF453344; **Taxon:** scientificName: Hylaeus (Spatulariella) punctatus (Brullé, 1832); order: Hymenoptera; family: Colletidae; genus: Hylaeus; subgenus: Spatulariella; specificEpithet: punctatus; scientificNameAuthorship: (Brullé, 1832); **Location:** country: Italy; countryCode: IT; stateProvince: Roma; locality: Riserva Naturale Valle dei Casali 1; decimalLatitude: 41.8710627; decimalLongitude: 12.4336809; geodeticDatum: WGS84; coordinatePrecision: 0.0002; **Identification:** identifiedBy: M. Mei; **Event:** eventDate: 2022-06-18; **Record Level:** collectionCode: UR3
- **Type status:** Other material. **Occurrence:** catalogNumber: A2116; recordedBy: L. Fortini; individualCount: 1; sex: female; lifeStage: adult; occurrenceID: 8CD99734-9E8B-5106-9B02-8389C2AA4B8F; **Taxon:** scientificName: Hylaeus (Spatulariella) punctatus (Brullé, 1832); order: Hymenoptera; family: Colletidae; genus: Hylaeus; subgenus: Spatulariella; specificEpithet: punctatus; scientificNameAuthorship: (Brullé, 1832); **Location:** country: Italy; countryCode: IT; stateProvince: Roma; locality: Riserva Naturale Valle dell'Aniene 2; decimalLatitude: 41.928752; decimalLongitude: 12.5562962; geodeticDatum: WGS84; coordinatePrecision: 0.0002; **Identification:** identifiedBy: M. Mei; **Event:** eventDate: 2022-08-03; **Record Level:** collectionCode: UR3
- **Type status:** Other material. **Occurrence:** catalogNumber: A2047; recordedBy: L. Fortini; individualCount: 1; sex: female; lifeStage: adult; occurrenceID: A25D2D72-E1FD-59F2-876D-9979BF49CE43; **Taxon:** scientificName: Hylaeus (Spatulariella) punctatus (Brullé, 1832); order: Hymenoptera; family: Colletidae; genus: Hylaeus; subgenus: Spatulariella; specificEpithet: punctatus; scientificNameAuthorship: (Brullé, 1832); **Location:** country: Italy; countryCode: IT; stateProvince: Roma; locality: Riserva Naturale Valle dei Casali 1; decimalLatitude: 41.8710627; decimalLongitude: 12.4336809; geodeticDatum: WGS84; coordinatePrecision: 0.0002; **Identification:** identifiedBy: M. Mei; **Event:** eventDate: 2022-08-19; **Record Level:** collectionCode: UR3
- **Type status:** Other material. **Occurrence:** catalogNumber: A0590; recordedBy: L. Fortini; individualCount: 1; sex: female; lifeStage: adult; occurrenceID: E3D42092-F06C-5381-AA39-5C386DBBB60A; **Taxon:** scientificName: Hylaeus (Spatulariella) punctatus (Brullé, 1832); order: Hymenoptera; family: Colletidae; genus: Hylaeus; subgenus: Spatulariella; specificEpithet: punctatus; scientificNameAuthorship: (Brullé, 1832); **Location:** country: Italy; countryCode: IT; stateProvince: Roma; locality: Riserva Naturale Laurentino-Acqua Acetosa; decimalLatitude: 41.807928; decimalLongitude: 12.468555; geodeticDatum: WGS84; coordinatePrecision: 0.0002; **Identification:** identifiedBy: M. Mei; **Event:** eventDate: 2022-09-14; **Record Level:** collectionCode: UR3
- **Type status:** Other material. **Occurrence:** catalogNumber: A0510, A0587; recordedBy: L. Fortini; individualCount: 2; sex: females; lifeStage: adult; occurrenceID: 4885C7EA-40C9-5A91-B73F-680E4DDB5BCF; **Taxon:** scientificName: Hylaeus (Spatulariella) punctatus (Brullé, 1832); order: Hymenoptera; family: Colletidae; genus: Hylaeus; subgenus: Spatulariella; specificEpithet: punctatus; scientificNameAuthorship: (Brullé, 1832); **Location:** country: Italy; countryCode: IT; stateProvince: Roma; locality: Riserva Naturale Laurentino-Acqua Acetosa; decimalLatitude: 41.807928; decimalLongitude: 12.468555; geodeticDatum: WGS84; coordinatePrecision: 0.0002; **Identification:** identifiedBy: M. Mei; **Event:** eventDate: 2022-08-21; **Record Level:** collectionCode: UR3
- **Type status:** Other material. **Occurrence:** catalogNumber: A0489, A0509, A0519, A0528, A0567, A0583, A0588, A0589, A0591, A0593, A0598; recordedBy: L. Fortini; individualCount: 11; sex: 4 males, 7 females; lifeStage: adult; occurrenceID: 9D45DC0F-5520-5E83-A694-7D2F8B1B0F4C; **Taxon:** scientificName: Hylaeus (Spatulariella) punctatus (Brullé, 1832); order: Hymenoptera; family: Colletidae; genus: Hylaeus; subgenus: Spatulariella; specificEpithet: punctatus; scientificNameAuthorship: (Brullé, 1832); **Location:** country: Italy; countryCode: IT; stateProvince: Roma; locality: Riserva Regionale dell'Appia Antica 2; decimalLatitude: 41.840256; decimalLongitude: 12.532773; geodeticDatum: WGS84; coordinatePrecision: 0.0002; **Identification:** identifiedBy: M. Mei; **Event:** eventDate: 2022-08-29; **Record Level:** collectionCode: UR3
- **Type status:** Other material. **Occurrence:** catalogNumber: A0592; recordedBy: L. Fortini; individualCount: 1; sex: female; lifeStage: adult; occurrenceID: 6ECFD9D8-3129-5C80-80FC-11F0D1D0CC42; **Taxon:** scientificName: Hylaeus (Spatulariella) punctatus (Brullé, 1832); order: Hymenoptera; family: Colletidae; genus: Hylaeus; subgenus: Spatulariella; specificEpithet: punctatus; scientificNameAuthorship: (Brullé, 1832); **Location:** country: Italy; countryCode: IT; stateProvince: Roma; locality: Riserva Naturale Valle dell'Aniene 1; decimalLatitude: 41.9345179; decimalLongitude: 12.5453096; geodeticDatum: WGS84; coordinatePrecision: 0.0002; **Identification:** identifiedBy: M. Mei; **Event:** eventDate: 2022-09-04; **Record Level:** collectionCode: UR3

#### Hylaeus punctulatissimus

Smith, 1842

40B42B9D-23E4-55F6-8B14-ADFCB1CD5357

##### Materials

- **Type status:** Other material. **Occurrence:** catalogNumber: A0578; recordedBy: L. Fortini; individualCount: 1; sex: male; lifeStage: adult; occurrenceID: C873D188-65E5-5BAC-8741-7E4D40E35698; **Taxon:** scientificName: Hylaeus (Koptogaster) punctulatissimus Smith, 1842; order: Hymenoptera; family: Colletidae; genus: Hylaeus; subgenus: Koptogaster; specificEpithet: punctulatissimus; scientificNameAuthorship: Smith, 1842; **Location:** country: Italy; countryCode: IT; stateProvince: Roma; locality: Riserva Naturale Laurentino-Acqua Acetosa; decimalLatitude: 41.807928; decimalLongitude: 12.468555; geodeticDatum: WGS84; coordinatePrecision: 0.0002; **Identification:** identifiedBy: M. Mei; **Event:** eventDate: 2022-07-17; **Record Level:** collectionCode: UR3

#### Hylaeus styriacus

Förster, 1871

C301C0AB-4306-57F1-A8F7-35D215F9F6A1

##### Materials

- **Type status:** Other material. **Occurrence:** catalogNumber: A0501; recordedBy: L. Fortini; individualCount: 1; sex: female; lifeStage: adult; occurrenceID: 02CEC1E5-D653-55D2-AD07-F14FF2DDFA0D; **Taxon:** scientificName: Hylaeus (Paraprosopis) styriacus Förster, 1871; order: Hymenoptera; family: Colletidae; genus: Hylaeus; subgenus: Paraprosopis; specificEpithet: styriacus; scientificNameAuthorship: Förster, 1871; **Location:** country: Italy; countryCode: IT; stateProvince: Roma; locality: Riserva Naturale Tenuta dei Massimi 1; decimalLatitude: 41.8532859; decimalLongitude: 12.3842322; geodeticDatum: WGS84; coordinatePrecision: 0.0002; **Identification:** identifiedBy: M. Mei; **Event:** eventDate: 2022-07-28; **Record Level:** collectionCode: UR3
- **Type status:** Other material. **Occurrence:** catalogNumber: A0531; recordedBy: L. Fortini; individualCount: 1; sex: female; lifeStage: adult; occurrenceID: 32E8B8E8-39E6-5FA0-A806-5395B0962B68; **Taxon:** scientificName: Hylaeus (Paraprosopis) styriacus Förster, 1871; order: Hymenoptera; family: Colletidae; genus: Hylaeus; subgenus: Paraprosopis; specificEpithet: styriacus; scientificNameAuthorship: Förster, 1871; **Location:** country: Italy; countryCode: IT; stateProvince: Roma; locality: Riserva Naturale dell'Acquafredda; decimalLatitude: 41.892841; decimalLongitude: 12.399320; geodeticDatum: WGS84; coordinatePrecision: 0.0002; **Identification:** identifiedBy: M. Mei; **Event:** eventDate: 2022-07-12; **Record Level:** collectionCode: UR3

#### Hylaeus taeniolatus

Förster, 1871

77D2C36B-ADA5-5C3C-89B7-DC8FF2475851

##### Materials

- **Type status:** Other material. **Occurrence:** catalogNumber: A0515; recordedBy: L. Fortini; individualCount: 1; sex: male; lifeStage: adult; occurrenceID: A4E7A262-22FE-5BD0-B2D1-D43D9BEA8269; **Taxon:** scientificName: Hylaeus (Paraprosopis) taeniolatus Förster, 1871; order: Hymenoptera; family: Colletidae; genus: Hylaeus; subgenus: Paraprosopis; specificEpithet: taeniolatus; scientificNameAuthorship: Förster, 1871; **Location:** country: Italy; countryCode: IT; stateProvince: Roma; locality: Riserva Naturale di Monte Mario; decimalLatitude: 41.938622; decimalLongitude: 12.454622; geodeticDatum: WGS84; coordinatePrecision: 0.0002; **Identification:** identifiedBy: M. Mei; **Event:** eventDate: 2022-05-20; **Record Level:** collectionCode: UR3
- **Type status:** Other material. **Occurrence:** catalogNumber: A0495, A0506, A0572; recordedBy: L. Fortini; individualCount: 3; sex: 1 male, 2 females; lifeStage: adult; occurrenceID: 646572CA-3D1C-59EE-AEE9-6908892AE56E; **Taxon:** scientificName: Hylaeus (Paraprosopis) taeniolatus Förster, 1871; order: Hymenoptera; family: Colletidae; genus: Hylaeus; subgenus: Paraprosopis; specificEpithet: taeniolatus; scientificNameAuthorship: Förster, 1871; **Location:** country: Italy; countryCode: IT; stateProvince: Roma; locality: Riserva Regionale dell'Appia Antica 2; decimalLatitude: 41.840256; decimalLongitude: 12.532773; geodeticDatum: WGS84; coordinatePrecision: 0.0002; **Identification:** identifiedBy: M. Mei; **Event:** eventDate: 2022-05-24; **Record Level:** collectionCode: UR3
- **Type status:** Other material. **Occurrence:** catalogNumber: A0539; recordedBy: L. Fortini; individualCount: 1; sex: female; lifeStage: adult; occurrenceID: DF22F0D2-37D7-5772-B826-B18EA51B5B9C; **Taxon:** scientificName: Hylaeus (Paraprosopis) taeniolatus Förster, 1871; order: Hymenoptera; family: Colletidae; genus: Hylaeus; subgenus: Paraprosopis; specificEpithet: taeniolatus; scientificNameAuthorship: Förster, 1871; **Location:** country: Italy; countryCode: IT; stateProvince: Roma; locality: Riserva Naturale Valle dei Casali 1; decimalLatitude: 41.8710627; decimalLongitude: 12.4336809; geodeticDatum: WGS84; coordinatePrecision: 0.0002; **Identification:** identifiedBy: M. Mei; **Event:** eventDate: 2022-07-13; **Record Level:** collectionCode: UR3
- **Type status:** Other material. **Occurrence:** catalogNumber: A0548; recordedBy: L. Fortini; individualCount: 1; sex: female; lifeStage: adult; occurrenceID: 01CB3667-1539-562E-B1B9-410A16856557; **Taxon:** scientificName: Hylaeus (Paraprosopis) taeniolatus Förster, 1871; order: Hymenoptera; family: Colletidae; genus: Hylaeus; subgenus: Paraprosopis; specificEpithet: taeniolatus; scientificNameAuthorship: Förster, 1871; **Location:** country: Italy; countryCode: IT; stateProvince: Roma; locality: Riserva Regionale dell'Appia Antica 2; decimalLatitude: 41.840256; decimalLongitude: 12.532773; geodeticDatum: WGS84; coordinatePrecision: 0.0002; **Identification:** identifiedBy: M. Mei; **Event:** eventDate: 2022-08-06; **Record Level:** collectionCode: UR3
- **Type status:** Other material. **Occurrence:** catalogNumber: A2035; recordedBy: L. Fortini; individualCount: 1; sex: female; lifeStage: adult; occurrenceID: A5CEC9A7-7798-569F-9D47-4CB362CA1FC5; **Taxon:** scientificName: Hylaeus (Paraprosopis) taeniolatus Förster, 1871; order: Hymenoptera; family: Colletidae; genus: Hylaeus; subgenus: Paraprosopis; specificEpithet: taeniolatus; scientificNameAuthorship: Förster, 1871; **Location:** country: Italy; countryCode: IT; stateProvince: Roma; locality: Riserva Naturale Valle dei Casali 1; decimalLatitude: 41.8710627; decimalLongitude: 12.4336809; geodeticDatum: WGS84; coordinatePrecision: 0.0002; **Identification:** identifiedBy: M. Mei; **Event:** eventDate: 2022-08-19; **Record Level:** collectionCode: UR3
- **Type status:** Other material. **Occurrence:** catalogNumber: A0557, A0580; recordedBy: L. Fortini; individualCount: 2; sex: 1 male, 1 female; lifeStage: adult; occurrenceID: 976DB222-5E67-5EAE-8F1D-45ABB7B4E911; **Taxon:** scientificName: Hylaeus (Paraprosopis) taeniolatus Förster, 1871; order: Hymenoptera; family: Colletidae; genus: Hylaeus; subgenus: Paraprosopis; specificEpithet: taeniolatus; scientificNameAuthorship: Förster, 1871; **Location:** country: Italy; countryCode: IT; stateProvince: Roma; locality: Riserva Regionale dell'Appia Antica 2; decimalLatitude: 41.840256; decimalLongitude: 12.532773; geodeticDatum: WGS84; coordinatePrecision: 0.0002; **Identification:** identifiedBy: M. Mei; **Event:** eventDate: 2022-08-29; **Record Level:** collectionCode: UR3

#### Hylaeus tyrolensis

Förster, 1871

6092C079-EB68-508E-8463-3F3D66F3EE71

##### Materials

- **Type status:** Other material. **Occurrence:** catalogNumber: A0492, A0502, A0503, A0518, A0523, A0522; recordedBy: L. Fortini; individualCount: 6; sex: 3 males, 3 females; lifeStage: adult; occurrenceID: 08AF274E-5F30-54CE-B950-509B31B553C4; **Taxon:** scientificName: Hylaeus (Hylaeus) tyrolensis Förster, 1871; order: Hymenoptera; family: Colletidae; genus: Hylaeus; subgenus: Hylaeus; specificEpithet: tyrolensis; scientificNameAuthorship: Förster, 1871; **Location:** country: Italy; countryCode: IT; stateProvince: Roma; locality: Riserva Naturale Tenuta dei Massimi 2; decimalLatitude: 41.831652; decimalLongitude: 12.399993; geodeticDatum: WGS84; coordinatePrecision: 0.0002; **Identification:** identifiedBy: M. Mei; **Event:** eventDate: 2022-06-01; **Record Level:** collectionCode: UR3
- **Type status:** Other material. **Occurrence:** catalogNumber: A0526, A0538; recordedBy: L. Fortini; individualCount: 2; sex: 1 male, 1 female; lifeStage: adult; occurrenceID: A7609DC3-CFEC-5D09-9276-70BB6FA2CDE2; **Taxon:** scientificName: Hylaeus (Hylaeus) tyrolensis Förster, 1871; order: Hymenoptera; family: Colletidae; genus: Hylaeus; subgenus: Hylaeus; specificEpithet: tyrolensis; scientificNameAuthorship: Förster, 1871; **Location:** country: Italy; countryCode: IT; stateProvince: Roma; locality: Riserva Naturale dell'Acquafredda; decimalLatitude: 41.892841; decimalLongitude: 12.399320; geodeticDatum: WGS84; coordinatePrecision: 0.0002; **Identification:** identifiedBy: M. Mei; **Event:** eventDate: 2022-06-10; **Record Level:** collectionCode: UR3
- **Type status:** Other material. **Occurrence:** catalogNumber: A0504, A0534; recordedBy: L. Fortini; individualCount: 2; sex: males; lifeStage: adult; occurrenceID: 957C01C3-0CA1-500B-A173-749C726ADD98; **Taxon:** scientificName: Hylaeus (Hylaeus) tyrolensis Förster, 1871; order: Hymenoptera; family: Colletidae; genus: Hylaeus; subgenus: Hylaeus; specificEpithet: tyrolensis; scientificNameAuthorship: Förster, 1871; **Location:** country: Italy; countryCode: IT; stateProvince: Roma; locality: Riserva Regionale dell'Appia Antica 1; decimalLatitude: 41.862394; decimalLongitude: 12.524863; geodeticDatum: WGS84; coordinatePrecision: 0.0002; **Identification:** identifiedBy: M. Mei; **Event:** eventDate: 2022-06-12; **Record Level:** collectionCode: UR3
- **Type status:** Other material. **Occurrence:** catalogNumber: A0535; recordedBy: L. Fortini; individualCount: 1; sex: female; lifeStage: adult; occurrenceID: 73D3456A-2793-5E42-A73F-EA1307FBEA38; **Taxon:** scientificName: Hylaeus (Hylaeus) tyrolensis Förster, 1871; order: Hymenoptera; family: Colletidae; genus: Hylaeus; subgenus: Hylaeus; specificEpithet: tyrolensis; scientificNameAuthorship: Förster, 1871; **Location:** country: Italy; countryCode: IT; stateProvince: Roma; locality: Riserva Naturale Valle dei Casali 1; decimalLatitude: 41.8710627; decimalLongitude: 12.4336809; geodeticDatum: WGS84; coordinatePrecision: 0.0002; **Identification:** identifiedBy: M. Mei; **Event:** eventDate: 2022-06-18; **Record Level:** collectionCode: UR3
- **Type status:** Other material. **Occurrence:** catalogNumber: A0532; recordedBy: L. Fortini; individualCount: 1; sex: female; lifeStage: adult; occurrenceID: 05509B17-9253-5029-BCF7-9142D1C69DB7; **Taxon:** scientificName: Hylaeus (Hylaeus) tyrolensis Förster, 1871; order: Hymenoptera; family: Colletidae; genus: Hylaeus; subgenus: Hylaeus; specificEpithet: tyrolensis; scientificNameAuthorship: Förster, 1871; **Location:** country: Italy; countryCode: IT; stateProvince: Roma; locality: Riserva Naturale di Monte Mario; decimalLatitude: 41.938622; decimalLongitude: 12.454622; geodeticDatum: WGS84; coordinatePrecision: 0.0002; **Identification:** identifiedBy: M. Mei; **Event:** eventDate: 2022-06-19; **Record Level:** collectionCode: UR3
- **Type status:** Other material. **Occurrence:** catalogNumber: A0545; recordedBy: L. Fortini; individualCount: 1; sex: male; lifeStage: adult; occurrenceID: 9446DA88-B3E4-5501-A857-FAEBC7005245; **Taxon:** scientificName: Hylaeus (Hylaeus) tyrolensis Förster, 1871; order: Hymenoptera; family: Colletidae; genus: Hylaeus; subgenus: Hylaeus; specificEpithet: tyrolensis; scientificNameAuthorship: Förster, 1871; **Location:** country: Italy; countryCode: IT; stateProvince: Roma; locality: Riserva Naturale Tenuta dei Massimi 2; decimalLatitude: 41.831652; decimalLongitude: 12.399993; geodeticDatum: WGS84; coordinatePrecision: 0.0002; **Identification:** identifiedBy: M. Mei; **Event:** eventDate: 2022-06-27; **Record Level:** collectionCode: UR3

#### Hylaeus variegatus

(Fabricius, 1798)

31DC05D0-97B2-5483-A93C-A772642F2651

##### Materials

- **Type status:** Other material. **Occurrence:** catalogNumber: A0483; recordedBy: L. Fortini; individualCount: 1; sex: female; lifeStage: adult; occurrenceID: 1C1378F7-53E4-5086-8B74-58702EE76582; **Taxon:** scientificName: Hylaeus (Prosopis) variegatus (Fabricius, 1798); order: Hymenoptera; family: Colletidae; genus: Hylaeus; subgenus: Prosopis; specificEpithet: variegatus; scientificNameAuthorship: (Fabricius, 1798); **Location:** country: Italy; countryCode: IT; stateProvince: Roma; locality: Riserva Naturale dell'Insugherata 2; decimalLatitude: 41.959925; decimalLongitude: 12.433852; geodeticDatum: WGS84; coordinatePrecision: 0.0002; **Identification:** identifiedBy: M. Mei; **Event:** eventDate: 2022-05-27; **Record Level:** collectionCode: UR3
- **Type status:** Other material. **Occurrence:** catalogNumber: A0484; recordedBy: L. Fortini; individualCount: 1; sex: female; lifeStage: adult; occurrenceID: CBEADAE2-5FD7-50E9-88CD-22488556AF0B; **Taxon:** scientificName: Hylaeus (Prosopis) variegatus (Fabricius, 1798); order: Hymenoptera; family: Colletidae; genus: Hylaeus; subgenus: Prosopis; specificEpithet: variegatus; scientificNameAuthorship: (Fabricius, 1798); **Location:** country: Italy; countryCode: IT; stateProvince: Roma; locality: Riserva Regionale dell'Appia Antica 1; decimalLatitude: 41.862394; decimalLongitude: 12.524863; geodeticDatum: WGS84; coordinatePrecision: 0.0002; **Identification:** identifiedBy: M. Mei; **Event:** eventDate: 2022-06-12; **Record Level:** collectionCode: UR3
- **Type status:** Other material. **Occurrence:** catalogNumber: A0542; recordedBy: L. Fortini; individualCount: 1; sex: male; lifeStage: adult; occurrenceID: D78813E9-AD56-5324-90E6-BF3743B55948; **Taxon:** scientificName: Hylaeus (Prosopis) variegatus (Fabricius, 1798); order: Hymenoptera; family: Colletidae; genus: Hylaeus; subgenus: Prosopis; specificEpithet: variegatus; scientificNameAuthorship: (Fabricius, 1798); **Location:** country: Italy; countryCode: IT; stateProvince: Roma; locality: Riserva Naturale dell'Acquafredda; decimalLatitude: 41.892841; decimalLongitude: 12.399320; geodeticDatum: WGS84; coordinatePrecision: 0.0002; **Identification:** identifiedBy: M. Mei; **Event:** eventDate: 2022-07-12; **Record Level:** collectionCode: UR3
- **Type status:** Other material. **Occurrence:** catalogNumber: A0499, A0508, A0555; recordedBy: L. Fortini; individualCount: 3; sex: males; lifeStage: adult; occurrenceID: 8013F707-4281-5DC5-A8E5-D8897FBA4A26; **Taxon:** scientificName: Hylaeus (Prosopis) variegatus (Fabricius, 1798); order: Hymenoptera; family: Colletidae; genus: Hylaeus; subgenus: Prosopis; specificEpithet: variegatus; scientificNameAuthorship: (Fabricius, 1798); **Location:** country: Italy; countryCode: IT; stateProvince: Roma; locality: Riserva Naturale Valle dei Casali 2; decimalLatitude: 41.859689; decimalLongitude: 12.435508; geodeticDatum: WGS84; coordinatePrecision: 0.0002; **Identification:** identifiedBy: M. Mei; **Event:** eventDate: 2022-07-13; **Record Level:** collectionCode: UR3
- **Type status:** Other material. **Occurrence:** catalogNumber: A0569, A0577; recordedBy: L. Fortini; individualCount: 2; sex: males; lifeStage: adult; occurrenceID: 3E376F03-896C-5D52-9A20-43F53E28D5A9; **Taxon:** scientificName: Hylaeus (Prosopis) variegatus (Fabricius, 1798); order: Hymenoptera; family: Colletidae; genus: Hylaeus; subgenus: Prosopis; specificEpithet: variegatus; scientificNameAuthorship: (Fabricius, 1798); **Location:** country: Italy; countryCode: IT; stateProvince: Roma; locality: Riserva Regionale dell'Appia Antica 1; decimalLatitude: 41.862394; decimalLongitude: 12.524863; geodeticDatum: WGS84; coordinatePrecision: 0.0002; **Identification:** identifiedBy: M. Mei; **Event:** eventDate: 2022-07-22; **Record Level:** collectionCode: UR3
- **Type status:** Other material. **Occurrence:** catalogNumber: A0482; recordedBy: L. Fortini; individualCount: 1; sex: female; lifeStage: adult; occurrenceID: C2AE50C5-A348-502C-8676-D6CBB1D4F8EC; **Taxon:** scientificName: Hylaeus (Prosopis) variegatus (Fabricius, 1798); order: Hymenoptera; family: Colletidae; genus: Hylaeus; subgenus: Prosopis; specificEpithet: variegatus; scientificNameAuthorship: (Fabricius, 1798); **Location:** country: Italy; countryCode: IT; stateProvince: Roma; locality: Riserva Naturale Tenuta dei Massimi 1; decimalLatitude: 41.8532859; decimalLongitude: 12.3842322; geodeticDatum: WGS84; coordinatePrecision: 0.0002; **Identification:** identifiedBy: M. Mei; **Event:** eventDate: 2022-07-28; **Record Level:** collectionCode: UR3
- **Type status:** Other material. **Occurrence:** catalogNumber: A0485, A0487, A0529, A0561; recordedBy: L. Fortini; individualCount: 4; sex: 1 male, 3 females; lifeStage: adult; occurrenceID: 4E516013-8197-57EA-8029-2286ED6FB5E4; **Taxon:** scientificName: Hylaeus (Prosopis) variegatus (Fabricius, 1798); order: Hymenoptera; family: Colletidae; genus: Hylaeus; subgenus: Prosopis; specificEpithet: variegatus; scientificNameAuthorship: (Fabricius, 1798); **Location:** country: Italy; countryCode: IT; stateProvince: Roma; locality: Riserva Naturale dell'Insugherata 1; decimalLatitude: 41.955505; decimalLongitude: 12.429232; geodeticDatum: WGS84; coordinatePrecision: 0.0002; **Identification:** identifiedBy: M. Mei; **Event:** eventDate: 2022-07-30; **Record Level:** collectionCode: UR3
- **Type status:** Other material. **Occurrence:** catalogNumber: A2090, A2091; recordedBy: L. Fortini; individualCount: 2; sex: females; lifeStage: adult; occurrenceID: D157C54E-1BA5-5132-9CFD-2DF1ECA4E7A1; **Taxon:** scientificName: Hylaeus (Prosopis) variegatus (Fabricius, 1798); order: Hymenoptera; family: Colletidae; genus: Hylaeus; subgenus: Prosopis; specificEpithet: variegatus; scientificNameAuthorship: (Fabricius, 1798); **Location:** country: Italy; countryCode: IT; stateProvince: Roma; locality: Riserva Regionale dell'Appia Antica 2; decimalLatitude: 41.840256; decimalLongitude: 12.532773; geodeticDatum: WGS84; coordinatePrecision: 0.0002; **Identification:** identifiedBy: M. Mei; **Event:** eventDate: 2022-08-06; **Record Level:** collectionCode: UR3
- **Type status:** Other material. **Occurrence:** catalogNumber: A0486; recordedBy: L. Fortini; individualCount: 1; sex: female; lifeStage: adult; occurrenceID: A154E1D8-2F3A-599D-BE6F-54E9389D0201; **Taxon:** scientificName: Hylaeus (Prosopis) variegatus (Fabricius, 1798); order: Hymenoptera; family: Colletidae; genus: Hylaeus; subgenus: Prosopis; specificEpithet: variegatus; scientificNameAuthorship: (Fabricius, 1798); **Location:** country: Italy; countryCode: IT; stateProvince: Roma; locality: Riserva Naturale Valle dei Casali 1; decimalLatitude: 41.8710627; decimalLongitude: 12.4336809; geodeticDatum: WGS84; coordinatePrecision: 0.0002; **Identification:** identifiedBy: M. Mei; **Event:** eventDate: 2022-09-18; **Record Level:** collectionCode: UR3

### Halictidae Thomson, 1869

#### Halictus compressus

(Walckenaer, 1802)

18BFCE69-1FA0-547E-BA01-F6600DAF783E

##### Materials

- **Type status:** Other material. **Occurrence:** catalogNumber: A1810; recordedBy: L. Fortini; individualCount: 1; sex: female; lifeStage: adult; occurrenceID: D9219092-42C2-5C89-9A74-AB70D03A6203; **Taxon:** scientificName: Halictus (Halictus) compressus (Walckenaer, 1802); order: Hymenoptera; family: Halictidae; genus: Halictus; subgenus: Halictus; specificEpithet: compressus; scientificNameAuthorship: (Walckenaer, 1802); **Location:** country: Italy; countryCode: IT; stateProvince: Roma; locality: Riserva Naturale dell'Acquafredda; decimalLatitude: 41.8928408; decimalLongitude: 12.39932; geodeticDatum: WGS84; coordinatePrecision: 0.0002; **Identification:** identifiedBy: M. Mei; **Event:** eventDate: 2022-07-12; **Record Level:** collectionID: UR3
- **Type status:** Other material. **Occurrence:** catalogNumber: A1811; recordedBy: L. Fortini; individualCount: 1; sex: female; lifeStage: adult; occurrenceID: 63C3F341-532A-5BD8-963C-05A05C8F520A; **Taxon:** scientificName: Halictus (Halictus) compressus (Walckenaer, 1802); order: Hymenoptera; family: Halictidae; genus: Halictus; subgenus: Halictus; specificEpithet: compressus; scientificNameAuthorship: (Walckenaer, 1802); **Location:** country: Italy; countryCode: IT; stateProvince: Roma; locality: Riserva Naturale dell'Acquafredda; decimalLatitude: 41.8928408; decimalLongitude: 12.39932; geodeticDatum: WGS84; coordinatePrecision: 0.0002; **Identification:** identifiedBy: M. Mei; **Event:** eventDate: 2022-06-10; **Record Level:** collectionID: UR3
- **Type status:** Other material. **Occurrence:** catalogNumber: A1618; recordedBy: L. Fortini; individualCount: 1; sex: female; lifeStage: adult; occurrenceID: CCBC2B34-A6EB-5823-95CF-B0634FBCB3F5; **Taxon:** scientificName: Halictus (Halictus) compressus (Walckenaer); order: Hymenoptera; family: Halictidae; genus: Halictus; subgenus: Halictus; specificEpithet: compressus; scientificNameAuthorship: (Walckenaer, 1802); **Location:** country: Italy; countryCode: IT; stateProvince: Roma; locality: Riserva Regionale dell'Appia Antica 1; decimalLatitude: 41.8623941; decimalLongitude: 12.524863; geodeticDatum: WGS84; coordinatePrecision: 0.0002; **Identification:** identifiedBy: M. Mei; **Event:** eventDate: 2022-07-22; **Record Level:** collectionID: UR3
- **Type status:** Other material. **Occurrence:** catalogNumber: A1749; recordedBy: L. Fortini; individualCount: 1; sex: female; lifeStage: adult; occurrenceID: 24515066-5819-5C4E-8AE1-A2C04C97ADCB; **Taxon:** scientificName: Halictus (Halictus) compressus (Walckeaner, 1802); order: Hymenoptera; family: Halictidae; genus: Halictus; subgenus: Halictus; specificEpithet: compressus; scientificNameAuthorship: (Walckenaer, 1802); **Location:** country: Italy; countryCode: IT; stateProvince: Roma; locality: Riserva Regionale dell'Appia Antica 3; decimalLatitude: 41.8298456; decimalLongitude: 12.5432538; geodeticDatum: WGS84; coordinatePrecision: 0.0002; **Identification:** identifiedBy: M. Mei; **Event:** eventDate: 2022-05-24; **Record Level:** collectionID: UR3
- **Type status:** Other material. **Occurrence:** catalogNumber: A1736; recordedBy: L. Fortini; individualCount: 1; sex: female; lifeStage: adult; occurrenceID: E97F8FD3-6623-5D12-8F43-E1E6EA622D4C; **Taxon:** scientificName: Halictus (Halictus) compressus (Walckeaner, 1802); order: Hymenoptera; family: Halictidae; genus: Halictus; subgenus: Halictus; specificEpithet: compressus; scientificNameAuthorship: (Walckenaer, 1802); **Location:** country: Italy; countryCode: IT; stateProvince: Roma; locality: Riserva Naturale Valle dell'Aniene 2; decimalLatitude: 41.928752; decimalLongitude: 12.5562962; geodeticDatum: WGS84; coordinatePrecision: 0.0002; **Identification:** identifiedBy: M. Mei; **Event:** eventDate: 2022-07-01; **Record Level:** collectionID: UR3
- **Type status:** Other material. **Occurrence:** catalogNumber: A1697, A1709; recordedBy: L. Fortini; individualCount: 2; sex: females; lifeStage: adult; occurrenceID: 18C07C73-FEEF-5509-8EDD-5CFCA2CC75BA; **Taxon:** scientificName: Halictus (Halictus) compressus (Walckeanear, 1802); order: Hymenoptera; family: Halictidae; genus: Halictus; subgenus: Halictus; specificEpithet: compressus.; scientificNameAuthorship: (Walckenaer, 1802); **Location:** country: Italy; countryCode: IT; stateProvince: Roma; locality: Riserva Naturale Valle dei Casali 2; decimalLatitude: 41.8596887; decimalLongitude: 12.4355075; geodeticDatum: WGS84; coordinatePrecision: 0.0002; **Identification:** identifiedBy: M. Mei; **Event:** eventDate: 2022-07-13; **Record Level:** collectionID: UR3

#### Halictus fulvipes

(Klug, 1817)

64A4B077-A466-55B5-8067-89E89C34EA05

##### Materials

- **Type status:** Other material. **Occurrence:** catalogNumber: A1717; recordedBy: L. Fortini; individualCount: 1; sex: female; lifeStage: adult; occurrenceID: FA60154E-3DF6-553D-9B56-11D1EE08D4B2; **Taxon:** scientificName: Halictus (Hexataenites) fulvipes (Klug, 1817); order: Hymenoptera; family: Halictidae; genus: Halictus; subgenus: Hexataenites; specificEpithet: fulvipes; scientificNameAuthorship: (Klug, 1817); **Location:** country: Italy; countryCode: IT; stateProvince: Roma; locality: Riserva Naturale dell'Acquafredda; decimalLatitude: 41.8928408; decimalLongitude: 12.39932; geodeticDatum: WGS84; coordinatePrecision: 0.0002; **Identification:** identifiedBy: M. Mei; **Event:** eventDate: 2022-06-10; **Record Level:** collectionID: UR3
- **Type status:** Other material. **Occurrence:** catalogNumber: A1698, A1699, A1700, A1706, A1728; recordedBy: L. Fortini; individualCount: 5; sex: females; lifeStage: adult; occurrenceID: 02A6A71C-DE9C-5C9C-B273-44ACF98F283F; **Taxon:** scientificName: Halictus (Hexataenites) fulvipes (Klug, 1817); order: Hymenoptera; family: Halictidae; genus: Halictus; subgenus: Hexataenites; specificEpithet: fulvipes; scientificNameAuthorship: (Klug, 1817); **Location:** country: Italy; countryCode: IT; stateProvince: Roma; locality: Riserva Naturale dell'Acquafredda; decimalLatitude: 41.8928408; decimalLongitude: 12.39932; geodeticDatum: WGS84; coordinatePrecision: 0.0002; **Identification:** identifiedBy: M. Mei; **Event:** eventDate: 2022-07-12; **Record Level:** collectionID: UR3
- **Type status:** Other material. **Occurrence:** catalogNumber: A1614; recordedBy: L. Fortini; individualCount: 1; sex: female; lifeStage: adult; occurrenceID: DB99C4D5-1FAC-513C-AD33-9A559EE1FA35; **Taxon:** scientificName: Halictus (Hexataenites) fulvipes (Klug, 1817); order: Hymenoptera; family: Halictidae; genus: Halictus; subgenus: Hexataenites; specificEpithet: fulvipes; scientificNameAuthorship: (Klug, 1817); **Location:** country: Italy; countryCode: IT; stateProvince: Roma; locality: Riserva Regionale dell'Appia Antica 1; decimalLatitude: 41.8623941; decimalLongitude: 12.524863; geodeticDatum: WGS84; coordinatePrecision: 0.0002; **Identification:** identifiedBy: M. Mei; **Event:** eventDate: 2022-08-25; **Record Level:** collectionID: UR3
- **Type status:** Other material. **Occurrence:** catalogNumber: A1616, A1701, A1704; recordedBy: L. Fortini; individualCount: 3; sex: females; lifeStage: adult; occurrenceID: 19BE8098-5106-5E85-B3DF-F2FCE630F46B; **Taxon:** scientificName: Halictus (Hexataenites) fulvipes (Klug, 1817); order: Hymenoptera; family: Halictidae; genus: Halictus; subgenus: Hexataenites; specificEpithet: fulvipes; scientificNameAuthorship: (Klug, 1817); **Location:** country: Italy; countryCode: IT; stateProvince: Roma; locality: Riserva Regionale dell'Appia Antica 1; decimalLatitude: 41.8623941; decimalLongitude: 12.524863; geodeticDatum: WGS84; coordinatePrecision: 0.0002; **Identification:** identifiedBy: M. Mei; **Event:** eventDate: 2022-07-22; **Record Level:** collectionID: UR3
- **Type status:** Other material. **Occurrence:** catalogNumber: A1703; recordedBy: L. Fortini; individualCount: 1; sex: female; lifeStage: adult; occurrenceID: EFAFAEFE-BCBD-5F02-A4EA-BA5C78E1A73A; **Taxon:** scientificName: Halictus (Hexataenites) fulvipes (Klug, 1817); order: Hymenoptera; family: Halictidae; genus: Halictus; subgenus: Hexataenites; specificEpithet: fulvipes; scientificNameAuthorship: (Klug, 1817); **Location:** country: Italy; countryCode: IT; stateProvince: Roma; locality: Riserva Naturale dell'Insugherata 3; decimalLatitude: 41.9644829; decimalLongitude: 12.436101; geodeticDatum: WGS84; coordinatePrecision: 0.0002; **Identification:** identifiedBy: M. Mei; **Event:** eventDate: 2022-07-30; **Record Level:** collectionID: UR3
- **Type status:** Other material. **Occurrence:** catalogNumber: A1713; recordedBy: L. Fortini; individualCount: 1; sex: female; lifeStage: adult; occurrenceID: E8978C18-B71D-5C3C-AC43-8C947FAB3002; **Taxon:** scientificName: Halictus (Hexataenites) fulvipes (Klug, 1817); order: Hymenoptera; family: Halictidae; genus: Halictus; subgenus: Hexataenites; specificEpithet: fulvipes; scientificNameAuthorship: (Klug, 1817); **Location:** country: Italy; countryCode: IT; stateProvince: Roma; locality: Riserva Naturale dell'Insugherata 3; decimalLatitude: 41.9644829; decimalLongitude: 12.436101; geodeticDatum: WGS84; coordinatePrecision: 0.0002; **Identification:** identifiedBy: M. Mei; **Event:** eventDate: 2022-06-24; **Record Level:** collectionID: UR3
- **Type status:** Other material. **Occurrence:** catalogNumber: A1615, A1720; recordedBy: L. Fortini; individualCount: 2; sex: females; lifeStage: adult; occurrenceID: 9673BC02-54F8-5B66-BFF8-A2411EEDCE3C; **Taxon:** scientificName: Halictus (Hexataenites) fulvipes (Klug, 1817); order: Hymenoptera; family: Halictidae; genus: Halictus; subgenus: Hexataenites; specificEpithet: fulvipes; scientificNameAuthorship: (Klug, 1817); **Location:** country: Italy; countryCode: IT; stateProvince: Roma; locality: Riserva Naturale Laurentino-Acqua Acetosa; decimalLatitude: 41.8079275; decimalLongitude: 12.4685548; geodeticDatum: WGS84; coordinatePrecision: 0.0002; **Identification:** identifiedBy: M. Mei; **Event:** eventDate: 2022-07-17; **Record Level:** collectionID: UR3
- **Type status:** Other material. **Occurrence:** catalogNumber: A1702; recordedBy: L. Fortini; individualCount: 1; sex: female; lifeStage: adult; occurrenceID: 78E1B925-803F-50A3-8739-AAE8A1B2C356; **Taxon:** scientificName: Halictus (Hexataenites) fulvipes (Klug, 1817); order: Hymenoptera; family: Halictidae; genus: Halictus; subgenus: Hexataenites; specificEpithet: fulvipes; scientificNameAuthorship: (Klug, 1817); **Location:** country: Italy; countryCode: IT; stateProvince: Roma; locality: Riserva Naturale Laurentino-Acqua Acetosa; decimalLatitude: 41.8079275; decimalLongitude: 12.4685548; geodeticDatum: WGS84; coordinatePrecision: 0.0002; **Identification:** identifiedBy: M. Mei; **Event:** eventDate: 2022-08-21; **Record Level:** collectionID: UR3
- **Type status:** Other material. **Occurrence:** catalogNumber: A1718; recordedBy: L. Fortini; individualCount: 1; sex: female; lifeStage: adult; occurrenceID: C9D49C51-5A8D-5D28-A98B-654E7CEC08B2; **Taxon:** scientificName: Halictus (Hexataenites) fulvipes (Klug, 1817); order: Hymenoptera; family: Halictidae; genus: Halictus; subgenus: Hexataenites; specificEpithet: fulvipes; scientificNameAuthorship: (Klug, 1817); **Location:** country: Italy; countryCode: IT; stateProvince: Roma; locality: Riserva Naturale di Monte Mario; decimalLatitude: 41.9386215; decimalLongitude: 12.4546223; geodeticDatum: WGS84; coordinatePrecision: 0.0002; **Identification:** identifiedBy: M. Mei; **Event:** eventDate: 2022-04-20; **Record Level:** collectionID: UR3
- **Type status:** Other material. **Occurrence:** catalogNumber: A1721, A1731, A1740; recordedBy: L. Fortini; individualCount: 3; sex: females; lifeStage: adult; occurrenceID: E9AD5263-07F9-5B1E-9AE9-6FD50D5DE51E; **Taxon:** scientificName: Halictus (Hexataenites) fulvipes (Klug, 1817); order: Hymenoptera; family: Halictidae; genus: Halictus; subgenus: Hexataenites; specificEpithet: fulvipes; scientificNameAuthorship: (Klug, 1817); **Location:** country: Italy; countryCode: IT; stateProvince: Roma; locality: Riserva Naturale di Monte Mario; decimalLatitude: 41.9386215; decimalLongitude: 12.4546223; geodeticDatum: WGS84; coordinatePrecision: 0.0002; **Identification:** identifiedBy: M. Mei; **Event:** eventDate: 2022-06-19; **Record Level:** collectionID: UR3
- **Type status:** Other material. **Occurrence:** catalogNumber: A1741; recordedBy: L. Fortini; individualCount: 1; sex: female; lifeStage: adult; occurrenceID: 571EA02A-AB5C-5E2C-B4E8-D42730B75347; **Taxon:** scientificName: Halictus (Hexataenites) fulvipes (Klug, 1817); order: Hymenoptera; family: Halictidae; genus: Halictus; subgenus: Hexataenites; specificEpithet: fulvipes; scientificNameAuthorship: (Klug, 1817); **Location:** country: Italy; countryCode: IT; stateProvince: Roma; locality: Riserva Naturale di Monte Mario; decimalLatitude: 41.9386215; decimalLongitude: 12.4546223; geodeticDatum: WGS84; coordinatePrecision: 0.0002; **Identification:** identifiedBy: M. Mei; **Event:** eventDate: 2022-05-20; **Record Level:** collectionID: UR3
- **Type status:** Other material. **Occurrence:** catalogNumber: A1714, A1716, A1743; recordedBy: L. Fortini; individualCount: 3; sex: females; lifeStage: adult; occurrenceID: 318905BC-20C6-5D82-8AAF-25946DCD16DB; **Taxon:** scientificName: Halictus (Hexataenites) fulvipes (Klug, 1817); order: Hymenoptera; family: Halictidae; genus: Halictus; subgenus: Hexataenites; specificEpithet: fulvipes; scientificNameAuthorship: (Klug, 1817); **Location:** country: Italy; countryCode: IT; stateProvince: Roma; locality: Riserva Naturale Tenuta dei Massimi 1; decimalLatitude: 41.8532859; decimalLongitude: 12.3842322; geodeticDatum: WGS84; coordinatePrecision: 0.0002; **Identification:** identifiedBy: M. Mei; **Event:** eventDate: 2022-04-23; **Record Level:** collectionID: UR3
- **Type status:** Other material. **Occurrence:** catalogNumber: A1710, A1726, A1730; recordedBy: L. Fortini; individualCount: 3; sex: females; lifeStage: adult; occurrenceID: B977867E-B227-5AA9-9A10-E1699F8F78D9; **Taxon:** scientificName: Halictus (Hexataenites) fulvipes (Klug, 1817); order: Hymenoptera; family: Halictidae; genus: Halictus; subgenus: Hexataenites; specificEpithet: fulvipes; scientificNameAuthorship: (Klug, 1817); **Location:** country: Italy; countryCode: IT; stateProvince: Roma; locality: Riserva Naturale Tenuta dei Massimi 1; decimalLatitude: 41.8532859; decimalLongitude: 12.3842322; geodeticDatum: WGS84; coordinatePrecision: 0.0002; **Identification:** identifiedBy: M. Mei; **Event:** eventDate: 2022-06-27; **Record Level:** collectionID: UR3
- **Type status:** Other material. **Occurrence:** catalogNumber: A1696, A1707; recordedBy: L. Fortini; individualCount: 2; sex: females; lifeStage: adult; occurrenceID: 025D6090-5255-57C2-91F4-9BB2630C8B0D; **Taxon:** scientificName: Halictus (Hexataenites) fulvipes (Klug, 1817); order: Hymenoptera; family: Halictidae; genus: Halictus; subgenus: Hexataenites; specificEpithet: fulvipes; scientificNameAuthorship: (Klug, 1817); **Location:** country: Italy; countryCode: IT; stateProvince: Roma; locality: Riserva Naturale Tenuta dei Massimi 1; decimalLatitude: 41.8532859; decimalLongitude: 12.3842322; geodeticDatum: WGS84; coordinatePrecision: 0.0002; **Identification:** identifiedBy: M. Mei; **Event:** eventDate: 2022-07-28; **Record Level:** collectionID: UR3
- **Type status:** Other material. **Occurrence:** catalogNumber: A1617; recordedBy: L. Fortini; individualCount: 1; sex: female; lifeStage: adult; occurrenceID: 68AF5E42-41E0-55E9-AAAA-B6FBB0002BF8; **Taxon:** scientificName: Halictus (Hexataenites) fulvipes (Klug, 1817); order: Hymenoptera; family: Halictidae; genus: Halictus; subgenus: Hexataenites; specificEpithet: fulvipes; scientificNameAuthorship: (Klug, 1817); **Location:** country: Italy; countryCode: IT; stateProvince: Roma; locality: Riserva Naturale Tenuta dei Massimi 2; decimalLatitude: 41.8316516; decimalLongitude: 12.3999927; geodeticDatum: WGS84; coordinatePrecision: 0.0002; **Identification:** identifiedBy: M. Mei; **Event:** eventDate: 2022-07-28; **Record Level:** collectionID: UR3
- **Type status:** Other material. **Occurrence:** catalogNumber: A1580; recordedBy: L. Fortini; individualCount: 1; sex: female; lifeStage: adult; occurrenceID: B8896583-15AF-5015-8D9F-70F5A5437E1B; **Taxon:** scientificName: Halictus (Hexataenites) fulvipes (Klug, 1817); order: Hymenoptera; family: Halictidae; genus: Halictus; subgenus: Hexataenites; specificEpithet: fulvipes; scientificNameAuthorship: (Klug, 1817); **Location:** country: Italy; countryCode: IT; stateProvince: Roma; locality: Riserva Naturale Valle dell'Aniene 1; decimalLatitude: 41.9345179; decimalLongitude: 12.5453096; geodeticDatum: WGS84; coordinatePrecision: 0.0002; **Identification:** identifiedBy: M. Mei; **Event:** eventDate: 2022-09-04; **Record Level:** collectionID: UR3
- **Type status:** Other material. **Occurrence:** catalogNumber: A1708, A1712, A1725, A1727, A1732; recordedBy: L. Fortini; individualCount: 5; sex: females; lifeStage: adult; occurrenceID: E6D534CB-AD7A-57DA-84C4-C2CC6C966557; **Taxon:** scientificName: Halictus (Hexataenites) fulvipes (Klug, 1817); order: Hymenoptera; family: Halictidae; genus: Halictus; subgenus: Hexataenites; specificEpithet: fulvipes; scientificNameAuthorship: (Klug, 1817); **Location:** country: Italy; countryCode: IT; stateProvince: Roma; locality: Riserva Naturale Valle dell'Aniene 1; decimalLatitude: 41.9345179; decimalLongitude: 12.5453096; geodeticDatum: WGS84; coordinatePrecision: 0.0002; **Identification:** identifiedBy: M. Mei; **Event:** eventDate: 2022-07-01; **Record Level:** collectionID: UR3
- **Type status:** Other material. **Occurrence:** catalogNumber: A1722, A1724, A1729, A1737, A1739, A1742, A1746; recordedBy: L. Fortini; individualCount: 7; sex: females; lifeStage: adult; occurrenceID: D19FFB73-5761-526A-BF8B-4A347562019C; **Taxon:** scientificName: Halictus (Hexataenites) fulvipes (Klug, 1817); order: Hymenoptera; family: Halictidae; genus: Halictus; subgenus: Hexataenites; specificEpithet: fulvipes; scientificNameAuthorship: (Klug, 1817); **Location:** country: Italy; countryCode: IT; stateProvince: Roma; locality: Riserva Naturale Valle dell'Aniene 2; decimalLatitude: 41.928752; decimalLongitude: 12.5562962; geodeticDatum: WGS84; coordinatePrecision: 0.0002; **Identification:** identifiedBy: M. Mei; **Event:** eventDate: 2022-07-01; **Record Level:** collectionID: UR3
- **Type status:** Other material. **Occurrence:** catalogNumber: A1563, A1565; recordedBy: L. Fortini; individualCount: 2; sex: females; lifeStage: adult; occurrenceID: B19F4BD6-88EB-5D0E-9193-5854804D1C47; **Taxon:** scientificName: Halictus (Hexataenites) fulvipes (Klug, 1817); order: Hymenoptera; family: Halictidae; genus: Halictus; subgenus: Hexataenites; specificEpithet: fulvipes; scientificNameAuthorship: (Klug, 1817); **Location:** country: Italy; countryCode: IT; stateProvince: Roma; locality: Riserva Naturale Valle dei Casali 2; decimalLatitude: 41.8596887; decimalLongitude: 12.4355075; geodeticDatum: WGS84; coordinatePrecision: 0.0002; **Identification:** identifiedBy: M. Mei; **Event:** eventDate: 2022-07-13; **Record Level:** collectionID: UR3
- **Type status:** Other material. **Occurrence:** catalogNumber: A1976, A1977, A1979, A1981; recordedBy: L. Fortini; individualCount: 4; sex: females; lifeStage: adult; occurrenceID: 442C63DE-1F22-51DC-8CAF-4F64D6FE6414; **Taxon:** scientificName: Halictus (Hexataenites) fulvipes (Klug, 1817); order: Hymenoptera; family: Halictidae; genus: Halictus; subgenus: Hexataenites; specificEpithet: fulvipes; scientificNameAuthorship: (Klug, 1817); **Location:** country: Italy; countryCode: IT; stateProvince: Roma; locality: Riserva Regionale dell'Appia Antica 1; decimalLatitude: 41.8623941; decimalLongitude: 12.524863; geodeticDatum: WGS84; coordinatePrecision: 0.0002; **Identification:** identifiedBy: M. Mei; **Event:** eventDate: 2022-04-19; **Record Level:** collectionID: UR3
- **Type status:** Other material. **Occurrence:** catalogNumber: A2023; recordedBy: L. Fortini; individualCount: 1; sex: female; lifeStage: adult; occurrenceID: DA6A7FA7-95F1-529F-8D3E-CE5AE3D81FD4; **Taxon:** scientificName: Halictus (Hexataenites) fulvipes (Klug, 1817); order: Hymenoptera; family: Halictidae; genus: Halictus; subgenus: Hexataenites; specificEpithet: fulvipes; scientificNameAuthorship: (Klug, 1817); **Location:** country: Italy; countryCode: IT; stateProvince: Roma; locality: Riserva Regionale dell'Appia Antica 2; decimalLatitude: 41.8402564; decimalLongitude: 12.532773; geodeticDatum: WGS84; coordinatePrecision: 0.0002; **Identification:** identifiedBy: M. Mei; **Event:** eventDate: 2022-04-25; **Record Level:** collectionID: UR3
- **Type status:** Other material. **Occurrence:** catalogNumber: A2074, A2075; recordedBy: L. Fortini; individualCount: 2; sex: females; lifeStage: adult; occurrenceID: 1F0E541F-D36E-5B3F-AE33-A9BF0B708871; **Taxon:** scientificName: Halictus (Hexataenites) fulvipes (Klug, 1817); order: Hymenoptera; family: Halictidae; genus: Halictus; subgenus: Hexataenites; specificEpithet: fulvipes; scientificNameAuthorship: (Klug, 1817); **Location:** country: Italy; countryCode: IT; stateProvince: Roma; locality: Riserva Naturale di Monte Mario; decimalLatitude: 41.9386215; decimalLongitude: 12.4546223; geodeticDatum: WGS84; coordinatePrecision: 0.0002; **Identification:** identifiedBy: M. Mei; **Event:** eventDate: 2022-07-24; **Record Level:** collectionID: UR3
- **Type status:** Other material. **Occurrence:** catalogNumber: A2083; recordedBy: L. Fortini; individualCount: 1; sex: female; lifeStage: adult; occurrenceID: F28D9EBF-BAB6-5570-ABA7-68D44CA9A1CD; **Taxon:** scientificName: Halictus (Hexataenites) fulvipes (Klug, 1817); order: Hymenoptera; family: Halictidae; genus: Halictus; subgenus: Hexataenites; specificEpithet: fulvipes; scientificNameAuthorship: (Klug, 1817); **Location:** country: Italy; countryCode: IT; stateProvince: Roma; locality: Riserva Regionale dell'Appia Antica 2; decimalLatitude: 41.8402564; decimalLongitude: 12.532773; geodeticDatum: WGS84; coordinatePrecision: 0.0002; **Identification:** identifiedBy: M. Mei; **Event:** eventDate: 2022-08-06; **Record Level:** collectionID: UR3
- **Type status:** Other material. **Occurrence:** catalogNumber: A2095; recordedBy: L. Fortini; individualCount: 1; sex: female; lifeStage: adult; occurrenceID: B0273DF5-46F5-5742-8FFD-C24E41B9D0A3; **Taxon:** scientificName: Halictus (Hexataenites) fulvipes (Klug, 1817); order: Hymenoptera; family: Halictidae; genus: Halictus; subgenus: Hexataenites; specificEpithet: fulvipes; scientificNameAuthorship: (Klug, 1817); **Location:** country: Italy; countryCode: IT; stateProvince: Roma; locality: Riserva Naturale Valle dell'Aniene 2; decimalLatitude: 41.928752; decimalLongitude: 12.5562962; geodeticDatum: WGS84; coordinatePrecision: 0.0002; **Identification:** identifiedBy: M. Mei; **Event:** eventDate: 2022-08-03; **Record Level:** collectionID: UR3

#### Halictus maculatus

Smith, 1848

44C30202-608B-53F4-AF38-3BA2F7C5A882

##### Materials

- **Type status:** Other material. **Occurrence:** catalogNumber: A1667, A1669, A1734; recordedBy: L. Fortini; individualCount: 3; sex: 2 males, 1 female; lifeStage: adult; occurrenceID: 879C7C46-8C41-54EE-8054-F02D332BD6E9; **Taxon:** scientificName: Halictus (Protohalictus) maculatus Smith, 1848; order: Hymenoptera; family: Halictidae; genus: Halictus; subgenus: Protohalictus; specificEpithet: maculatus; scientificNameAuthorship: Smith, 1848; **Location:** country: Italy; countryCode: IT; stateProvince: Roma; locality: Riserva Naturale dell'Acquafredda; decimalLatitude: 41.8928408; decimalLongitude: 12.39932; geodeticDatum: WGS84; coordinatePrecision: 0.0002; **Identification:** identifiedBy: M. Mei; **Event:** eventDate: 2022-07-12; **Record Level:** collectionID: UR3
- **Type status:** Other material. **Occurrence:** catalogNumber: A1747, A1748; recordedBy: L. Fortini; individualCount: 2; sex: females; lifeStage: adult; occurrenceID: 5BD93F3A-4415-5A1A-8127-F6ECEA36650E; **Taxon:** scientificName: Halictus (Protohalictus) maculatus Smith, 1848; order: Hymenoptera; family: Halictidae; genus: Halictus; subgenus: Protohalictus; specificEpithet: maculatus; scientificNameAuthorship: Smith, 1848; **Location:** country: Italy; countryCode: IT; stateProvince: Roma; locality: Riserva Naturale dell'Insugherata 3; decimalLatitude: 41.9644829; decimalLongitude: 12.436101; geodeticDatum: WGS84; coordinatePrecision: 0.0002; **Identification:** identifiedBy: M. Mei; **Event:** eventDate: 2022-06-24; **Record Level:** collectionID: UR3
- **Type status:** Other material. **Occurrence:** catalogNumber: A1670; recordedBy: L. Fortini; individualCount: 1; sex: male; lifeStage: adult; occurrenceID: A2E46AB8-A80A-598D-9C70-2AA6B43D6842; **Taxon:** scientificName: Halictus (Protohalictus) maculatus Smith, 1848; order: Hymenoptera; family: Halictidae; genus: Halictus; subgenus: Protohalictus; specificEpithet: maculatus; scientificNameAuthorship: Smith, 1848; **Location:** country: Italy; countryCode: IT; stateProvince: Roma; locality: Riserva Naturale Valle dell'Aniene 2; decimalLatitude: 41.928752; decimalLongitude: 12.5562962; geodeticDatum: WGS84; coordinatePrecision: 0.0002; **Identification:** identifiedBy: M. Mei; **Event:** eventDate: 2022-07-01; **Record Level:** collectionID: UR3
- **Type status:** Other material. **Occurrence:** catalogNumber: A1738, A1751; recordedBy: L. Fortini; individualCount: 2; sex: females; lifeStage: adult; occurrenceID: DDD78A1E-065C-5998-BCF0-CC15C0F204E8; **Taxon:** scientificName: Halictus (Protohalictus) maculatus Smith, 1848; order: Hymenoptera; family: Halictidae; genus: Halictus; subgenus: Protohalictus; specificEpithet: maculatus; scientificNameAuthorship: Smith, 1848; **Location:** country: Italy; countryCode: IT; stateProvince: Roma; locality: Riserva Naturale Valle dell'Aniene 2; decimalLatitude: 41.928752; decimalLongitude: 12.5562962; geodeticDatum: WGS84; coordinatePrecision: 0.0002; **Identification:** identifiedBy: M. Mei; **Event:** eventDate: 2022-06-05; **Record Level:** collectionID: UR3
- **Type status:** Other material. **Occurrence:** catalogNumber: A1613, A1668; recordedBy: L. Fortini; individualCount: 2; sex: 1 male, 1 female; lifeStage: adult; occurrenceID: BA24A16D-76F0-578D-BFF7-1EDA95F74D99; **Taxon:** scientificName: Halictus (Protohalictus) maculatus Smith, 1848; order: Hymenoptera; family: Halictidae; genus: Halictus; subgenus: Protohalictus; specificEpithet: maculatus; scientificNameAuthorship: Smith, 1848; **Location:** country: Italy; countryCode: IT; stateProvince: Roma; locality: Riserva Naturale Valle dei Casali 2; decimalLatitude: 41.8596887; decimalLongitude: 12.4355075; geodeticDatum: WGS84; coordinatePrecision: 0.0002; **Identification:** identifiedBy: M. Mei; **Event:** eventDate: 2022-07-13; **Record Level:** collectionID: UR3

#### Halictus quadricinctus

(Fabricius, 1776)

CF42F857-E31F-591A-AF86-237418A1C82B

##### Materials

- **Type status:** Other material. **Occurrence:** catalogNumber: A1757, A1760; recordedBy: L. Fortini; individualCount: 2; sex: females; lifeStage: adult; occurrenceID: A54C23B6-0164-5490-9B21-5D536B016000; **Taxon:** scientificName: Halictus (Halictus) quadricinctus (Fabricius, 1776); order: Hymenoptera; family: Halictidae; genus: Halictus; subgenus: Halictus; specificEpithet: quadricinctus; scientificNameAuthorship: (Fabricius, 1776); **Location:** country: Italy; countryCode: IT; stateProvince: Roma; locality: Riserva Naturale Laurentino-Acqua Acetosa; decimalLatitude: 41.8079275; decimalLongitude: 12.4685548; geodeticDatum: WGS84; coordinatePrecision: 0.0002; **Identification:** identifiedBy: M. Mei; **Event:** eventDate: 2022-05-12; **Record Level:** collectionID: UR3
- **Type status:** Other material. **Occurrence:** catalogNumber: A1765; recordedBy: L. Fortini; individualCount: 1; sex: female; lifeStage: adult; occurrenceID: D3E9C7F7-F0A4-582D-9748-8F6B5267ED75; **Taxon:** scientificName: Halictus (Halictus) quadricinctus (Fabricius, 1776); order: Hymenoptera; family: Halictidae; genus: Halictus; subgenus: Halictus; specificEpithet: quadricinctus; scientificNameAuthorship: (Fabricius, 1776); **Location:** country: Italy; countryCode: IT; stateProvince: Roma; locality: Riserva Naturale Valle dei Casali 2; decimalLatitude: 41.8596887; decimalLongitude: 12.4355075; geodeticDatum: WGS84; coordinatePrecision: 0.0002; **Identification:** identifiedBy: M. Mei; **Event:** eventDate: 2022-05-13; **Record Level:** collectionID: UR3
- **Type status:** Other material. **Occurrence:** catalogNumber: A1756; recordedBy: L. Fortini; individualCount: 1; sex: female; lifeStage: adult; occurrenceID: B4351BFD-D9CF-51FA-B89A-3ED6009C1E1B; **Taxon:** scientificName: Halictus (Halictus) quadricinctus (Fabricius, 1776); order: Hymenoptera; family: Halictidae; genus: Halictus; subgenus: Halictus; specificEpithet: quadricinctus; scientificNameAuthorship: (Fabricius, 1776); **Location:** country: Italy; countryCode: IT; stateProvince: Roma; locality: Riserva Naturale Valle dei Casali 1; decimalLatitude: 41.8710627; decimalLongitude: 12.4336809; geodeticDatum: WGS84; coordinatePrecision: 0.0002; **Identification:** identifiedBy: M. Mei; **Event:** eventDate: 2022-05-14; **Record Level:** collectionID: UR3
- **Type status:** Other material. **Occurrence:** catalogNumber: A1755, A1767; recordedBy: L. Fortini; individualCount: 2; sex: females; lifeStage: adult; occurrenceID: 04F2D612-8599-5DF6-869A-E915EA2FEB9D; **Taxon:** scientificName: Halictus (Halictus) quadricinctus (Fabricius, 1776); order: Hymenoptera; family: Halictidae; genus: Halictus; subgenus: Halictus; specificEpithet: quadricinctus; scientificNameAuthorship: (Fabricius, 1776); **Location:** country: Italy; countryCode: IT; stateProvince: Roma; locality: Riserva Regionale dell'Appia Antica 3; decimalLatitude: 41.8298456; decimalLongitude: 12.5432538; geodeticDatum: WGS84; coordinatePrecision: 0.0002; **Identification:** identifiedBy: M. Mei; **Event:** eventDate: 2022-05-24; **Record Level:** collectionID: UR3
- **Type status:** Other material. **Occurrence:** catalogNumber: A1758; recordedBy: L. Fortini; individualCount: 1; sex: female; lifeStage: adult; occurrenceID: F26CFA49-9696-5D97-860E-AF80BD6EE429; **Taxon:** scientificName: Halictus (Halictus) quadricinctus (Fabricius, 1776); order: Hymenoptera; family: Halictidae; genus: Halictus; subgenus: Halictus; specificEpithet: quadricinctus; scientificNameAuthorship: (Fabricius, 1776); **Location:** country: Italy; countryCode: IT; stateProvince: Roma; locality: Riserva Naturale dell'Insugherata 1; decimalLatitude: 41.9555045; decimalLongitude: 12.4292321; geodeticDatum: WGS84; coordinatePrecision: 0.0002; **Identification:** identifiedBy: M. Mei; **Event:** eventDate: 2022-05-27; **Record Level:** collectionID: UR3
- **Type status:** Other material. **Occurrence:** catalogNumber: A1759; recordedBy: L. Fortini; individualCount: 1; sex: female; lifeStage: adult; occurrenceID: 6B5A2949-70CA-5908-BEBE-5D54757294C0; **Taxon:** scientificName: Halictus (Halictus) quadricinctus (Fabricius, 1776); order: Hymenoptera; family: Halictidae; genus: Halictus; subgenus: Halictus; specificEpithet: quadricinctus; scientificNameAuthorship: (Fabricius, 1776); **Location:** country: Italy; countryCode: IT; stateProvince: Roma; locality: Riserva Naturale dell'Insugherata 3; decimalLatitude: 41.9644829; decimalLongitude: 12.436101; geodeticDatum: WGS84; coordinatePrecision: 0.0002; **Identification:** identifiedBy: M. Mei; **Event:** eventDate: 2022-05-27; **Record Level:** collectionID: UR3
- **Type status:** Other material. **Occurrence:** catalogNumber: A1761, A1763, A1764; recordedBy: L. Fortini; individualCount: 3; sex: females; lifeStage: adult; occurrenceID: EC870984-92F8-59D8-A9FC-A23188D4F78C; **Taxon:** scientificName: Halictus (Halictus) quadricinctus (Fabricius, 1776); order: Hymenoptera; family: Halictidae; genus: Halictus; subgenus: Halictus; specificEpithet: quadricinctus; scientificNameAuthorship: (Fabricius, 1776); **Location:** country: Italy; countryCode: IT; stateProvince: Roma; locality: Riserva Naturale Valle dell'Aniene 1; decimalLatitude: 41.9345179; decimalLongitude: 12.5453096; geodeticDatum: WGS84; coordinatePrecision: 0.0002; **Identification:** identifiedBy: M. Mei; **Event:** eventDate: 2022-07-01; **Record Level:** collectionID: UR3
- **Type status:** Other material. **Occurrence:** catalogNumber: A1762, A1766; recordedBy: L. Fortini; individualCount: 2; sex: females; lifeStage: adult; occurrenceID: 1A4042D3-E860-50A4-9EAD-D8DD3EAEBA87; **Taxon:** scientificName: Halictus (Halictus) quadricinctus (Fabricius, 1776); order: Hymenoptera; family: Halictidae; genus: Halictus; subgenus: Halictus; specificEpithet: quadricinctus; scientificNameAuthorship: (Fabricius, 1776); **Location:** country: Italy; countryCode: IT; stateProvince: Roma; locality: Riserva Naturale Valle dell'Aniene 2; decimalLatitude: 41.928752; decimalLongitude: 12.5562962; geodeticDatum: WGS84; coordinatePrecision: 0.0002; **Identification:** identifiedBy: M. Mei; **Event:** eventDate: 2022-07-01; **Record Level:** collectionID: UR3
- **Type status:** Other material. **Occurrence:** catalogNumber: A1666; recordedBy: L. Fortini; individualCount: 1; sex: female; lifeStage: adult; occurrenceID: 9AF41CB9-E3A3-5883-9A82-C95F49637703; **Taxon:** scientificName: Halictus (Halictus) quadricinctus (Fabricius, 1776); order: Hymenoptera; family: Halictidae; genus: Halictus; subgenus: Halictus; specificEpithet: quadricinctus; scientificNameAuthorship: (Fabricius, 1776); **Location:** country: Italy; countryCode: IT; stateProvince: Roma; locality: Riserva Naturale dell'Insugherata 1; decimalLatitude: 41.9555045; decimalLongitude: 12.4292321; geodeticDatum: WGS84; coordinatePrecision: 0.0002; **Identification:** identifiedBy: M. Mei; **Event:** eventDate: 2022-07-30; **Record Level:** collectionID: UR3
- **Type status:** Other material. **Occurrence:** catalogNumber: A2103, A2104; recordedBy: L. Fortini; individualCount: 2; sex: males; lifeStage: adult; occurrenceID: 9AF1A9C4-E649-563B-A9D0-A5F65F4FB2EB; **Taxon:** scientificName: Halictus (Halictus) quadricinctus (Fabricius, 1776); order: Hymenoptera; family: Halictidae; genus: Halictus; subgenus: Halictus; specificEpithet: quadricinctus; scientificNameAuthorship: (Fabricius, 1776); **Location:** country: Italy; countryCode: IT; stateProvince: Roma; locality: Riserva Naturale Valle dell'Aniene 2; decimalLatitude: 41.928752; decimalLongitude: 12.5562962; geodeticDatum: WGS84; coordinatePrecision: 0.0002; **Identification:** identifiedBy: M. Mei; **Event:** eventDate: 2022-08-03; **Record Level:** collectionID: UR3

#### Halictus scabiosae

(Rossi, 1790)

5ED563AA-ABB5-55BE-8952-6CB157524556

##### Materials

- **Type status:** Other material. **Occurrence:** catalogNumber: A1869, A1880; recordedBy: L. Fortini; individualCount: 2; sex: females; lifeStage: adult; occurrenceID: E6675EDE-BE72-5D9F-BF13-6D80BDE61885; **Taxon:** scientificName: Halictus (Hexataenites) scabiosae (Rossi, 1790); order: Hymenoptera; family: Halictidae; genus: Halictus; subgenus: Hexataenites; specificEpithet: scabiosae; scientificNameAuthorship: (Rossi, 1790); **Location:** country: Italy; countryCode: IT; stateProvince: Roma; locality: Riserva Naturale dell'Acquafredda; decimalLatitude: 41.8928408; decimalLongitude: 12.39932; geodeticDatum: WGS84; coordinatePrecision: 0.0002; **Identification:** identifiedBy: M. Mei; **Event:** eventDate: 2022-06-10; **Record Level:** collectionID: UR3
- **Type status:** Other material. **Occurrence:** catalogNumber: A1651, A1652, A1654, A1657, A1723, A1824, A1827, A1828, A1831, A1840, A1841, A1844, A1845, A1846, A1901, A1904; recordedBy: L. Fortini; individualCount: 16; sex: 4 males, 12 females; lifeStage: adult; occurrenceID: 9CD1A345-C530-52D2-BCC5-D542070A3977; **Taxon:** scientificName: Halictus (Hexataenites) scabiosae (Rossi, 1790); order: Hymenoptera; family: Halictidae; genus: Halictus; subgenus: Hexataenites; specificEpithet: scabiosae; scientificNameAuthorship: (Rossi, 1790); **Location:** country: Italy; countryCode: IT; stateProvince: Roma; locality: Riserva Naturale dell'Acquafredda; decimalLatitude: 41.8928408; decimalLongitude: 12.39932; geodeticDatum: WGS84; coordinatePrecision: 0.0002; **Identification:** identifiedBy: M. Mei; **Event:** eventDate: 2022-07-12; **Record Level:** collectionID: UR3
- **Type status:** Other material. **Occurrence:** catalogNumber: A1642, A1653, A1656, A1661, A1663, A1814; recordedBy: L. Fortini; individualCount: 6; sex: 5 males, 1 female; lifeStage: adult; occurrenceID: 5B3651D1-2DD7-51B2-B3E0-78BC693EB5FD; **Taxon:** scientificName: Halictus (Hexataenites) scabiosae (Rossi, 1790); order: Hymenoptera; family: Halictidae; genus: Halictus; subgenus: Hexataenites; specificEpithet: scabiosae; scientificNameAuthorship: (Rossi, 1790); **Location:** country: Italy; countryCode: IT; stateProvince: Roma; locality: Riserva Regionale dell'Appia Antica 1; decimalLatitude: 41.8623941; decimalLongitude: 12.524863; geodeticDatum: WGS84; coordinatePrecision: 0.0002; **Identification:** identifiedBy: M. Mei; **Event:** eventDate: 2022-07-22; **Record Level:** collectionID: UR3
- **Type status:** Other material. **Occurrence:** catalogNumber: A1865, A1870, A1872, A1886; recordedBy: L. Fortini; individualCount: 4; sex: females; lifeStage: adult; occurrenceID: F700A610-8B03-5FE8-9F3F-CFD719A35E72; **Taxon:** scientificName: Halictus (Hexataenites) scabiosae (Rossi, 1790); order: Hymenoptera; family: Halictidae; genus: Halictus; subgenus: Hexataenites; specificEpithet: scabiosae; scientificNameAuthorship: (Rossi, 1790); **Location:** country: Italy; countryCode: IT; stateProvince: Roma; locality: Riserva Regionale dell'Appia Antica 1; decimalLatitude: 41.8623941; decimalLongitude: 12.524863; geodeticDatum: WGS84; coordinatePrecision: 0.0002; **Identification:** identifiedBy: M. Mei; **Event:** eventDate: 2022-06-12; **Record Level:** collectionID: UR3
- **Type status:** Other material. **Occurrence:** catalogNumber: A1890; recordedBy: L. Fortini; individualCount: 1; sex: female; lifeStage: adult; occurrenceID: 5A42BD4F-93E4-5F9A-A8CA-82540CB54671; **Taxon:** scientificName: Halictus (Hexataenites) scabiosae (Rossi, 1790); order: Hymenoptera; family: Halictidae; genus: Halictus; subgenus: Hexataenites; specificEpithet: scabiosae; scientificNameAuthorship: (Rossi, 1790); **Location:** country: Italy; countryCode: IT; stateProvince: Roma; locality: Riserva Regionale dell'Appia Antica 2; decimalLatitude: 41.8402564; decimalLongitude: 12.532773; geodeticDatum: WGS84; coordinatePrecision: 0.0002; **Identification:** identifiedBy: M. Mei; **Event:** eventDate: 2022-05-24; **Record Level:** collectionID: UR3
- **Type status:** Other material. **Occurrence:** catalogNumber: A1817, A1842, A1848, A1849, A1858, A1903; recordedBy: L. Fortini; individualCount: 6; sex: females; lifeStage: adult; occurrenceID: 6B278CD1-3A65-5795-85E0-1A6F27F75466; **Taxon:** scientificName: Halictus (Hexataenites) scabiosae (Rossi, 1790); order: Hymenoptera; family: Halictidae; genus: Halictus; subgenus: Hexataenites; specificEpithet: scabiosae; scientificNameAuthorship: (Rossi, 1790); **Location:** country: Italy; countryCode: IT; stateProvince: Roma; locality: Riserva Regionale dell'Appia Antica 2; decimalLatitude: 41.8402564; decimalLongitude: 12.532773; geodeticDatum: WGS84; coordinatePrecision: 0.0002; **Identification:** identifiedBy: M. Mei; **Event:** eventDate: 2022-07-07; **Record Level:** collectionID: UR3
- **Type status:** Other material. **Occurrence:** catalogNumber: A1887; recordedBy: L. Fortini; individualCount: 1; sex: male; lifeStage: adult; occurrenceID: CB30EFA3-18A9-5261-B124-61251910D508; **Taxon:** scientificName: Halictus (Hexataenites) scabiosae (Rossi, 1790); order: Hymenoptera; family: Halictidae; genus: Halictus; subgenus: Hexataenites; specificEpithet: scabiosae; scientificNameAuthorship: (Rossi, 1790); **Location:** country: Italy; countryCode: IT; stateProvince: Roma; locality: Riserva Regionale dell'Appia Antica 3; decimalLatitude: 41.8298456; decimalLongitude: 12.5432538; geodeticDatum: WGS84; coordinatePrecision: 0.0002; **Identification:** identifiedBy: M. Mei; **Event:** eventDate: 2022-05-24; **Record Level:** collectionID: UR3
- **Type status:** Other material. **Occurrence:** catalogNumber: A1889, A1898; recordedBy: L. Fortini; individualCount: 2; sex: males; lifeStage: adult; occurrenceID: A3C9F272-0BDE-5C78-BD51-5E662A5BA63C; **Taxon:** scientificName: Halictus (Hexataenites) scabiosae (Rossi, 1790); order: Hymenoptera; family: Halictidae; genus: Halictus; subgenus: Hexataenites; specificEpithet: scabiosae; scientificNameAuthorship: (Rossi, 1790); **Location:** country: Italy; countryCode: IT; stateProvince: Roma; locality: Riserva Naturale dell'Insugherata 1; decimalLatitude: 41.9555045; decimalLongitude: 12.4292321; geodeticDatum: WGS84; coordinatePrecision: 0.0002; **Identification:** identifiedBy: M. Mei; **Event:** eventDate: 2022-05-27; **Record Level:** collectionID: UR3
- **Type status:** Other material. **Occurrence:** catalogNumber: A1646, A1879; recordedBy: L. Fortini; individualCount: 2; sex: 1 male, 1 female; lifeStage: adult; occurrenceID: 89CB35BA-92D1-5A83-8C67-8B7C1E07E2F8; **Taxon:** scientificName: Halictus (Hexataenites) scabiosae (Rossi, 1790); order: Hymenoptera; family: Halictidae; genus: Halictus; subgenus: Hexataenites; specificEpithet: scabiosae; scientificNameAuthorship: (Rossi, 1790); **Location:** country: Italy; countryCode: IT; stateProvince: Roma; locality: Riserva Naturale dell'Insugherata 1; decimalLatitude: 41.9555045; decimalLongitude: 12.4292321; geodeticDatum: WGS84; coordinatePrecision: 0.0002; **Identification:** identifiedBy: M. Mei; **Event:** eventDate: 2022-06-24; **Record Level:** collectionID: UR3
- **Type status:** Other material. **Occurrence:** catalogNumber: A1648; recordedBy: L. Fortini; individualCount: 1; sex: male; lifeStage: adult; occurrenceID: 0FC0B8BD-3372-5589-8F4C-42FA784422FC; **Taxon:** scientificName: Halictus (Hexataenites) scabiosae (Rossi, 1790); order: Hymenoptera; family: Halictidae; genus: Halictus; subgenus: Hexataenites; specificEpithet: scabiosae; scientificNameAuthorship: (Rossi, 1790); **Location:** country: Italy; countryCode: IT; stateProvince: Roma; locality: Riserva Naturale dell'Insugherata 1; decimalLatitude: 41.9555045; decimalLongitude: 12.4292321; geodeticDatum: WGS84; coordinatePrecision: 0.0002; **Identification:** identifiedBy: M. Mei; **Event:** eventDate: 2022-07-30; **Record Level:** collectionID: UR3
- **Type status:** Other material. **Occurrence:** catalogNumber: A1647, A1659, A1664; recordedBy: L. Fortini; individualCount: 3; sex: males; lifeStage: adult; occurrenceID: 8974FE5B-0A5A-5917-BA03-661F29C33B5D; **Taxon:** scientificName: Halictus (Hexataenites) scabiosae (Rossi, 1790); order: Hymenoptera; family: Halictidae; genus: Halictus; subgenus: Hexataenites; specificEpithet: scabiosae; scientificNameAuthorship: (Rossi, 1790); **Location:** country: Italy; countryCode: IT; stateProvince: Roma; locality: Riserva Naturale dell'Insugherata 2; decimalLatitude: 41.9599247; decimalLongitude: 12.433852; geodeticDatum: WGS84; coordinatePrecision: 0.0002; **Identification:** identifiedBy: M. Mei; **Event:** eventDate: 2022-07-30; **Record Level:** collectionID: UR3
- **Type status:** Other material. **Occurrence:** catalogNumber: A1662; recordedBy: L. Fortini; individualCount: 1; sex: male; lifeStage: adult; occurrenceID: 87E498FC-D9FA-59B9-AB00-4EC18B3C85C0; **Taxon:** scientificName: Halictus (Hexataenites) scabiosae (Rossi, 1790); order: Hymenoptera; family: Halictidae; genus: Halictus; subgenus: Hexataenites; specificEpithet: scabiosae; scientificNameAuthorship: (Rossi, 1790); **Location:** country: Italy; countryCode: IT; stateProvince: Roma; locality: Riserva Naturale dell'Insugherata 3; decimalLatitude: 41.9644829; decimalLongitude: 12.436101; geodeticDatum: WGS84; coordinatePrecision: 0.0002; **Identification:** identifiedBy: M. Mei; **Event:** eventDate: 2022-07-30; **Record Level:** collectionID: UR3
- **Type status:** Other material. **Occurrence:** catalogNumber: A1881; recordedBy: L. Fortini; individualCount: 1; sex: female; lifeStage: adult; occurrenceID: 0CEA8B29-C167-5E9C-BF87-84BE45AF06B3; **Taxon:** scientificName: Halictus (Hexataenites) scabiosae (Rossi, 1790); order: Hymenoptera; family: Halictidae; genus: Halictus; subgenus: Hexataenites; specificEpithet: scabiosae; scientificNameAuthorship: (Rossi, 1790); **Location:** country: Italy; countryCode: IT; stateProvince: Roma; locality: Riserva Naturale dell'Insugherata 3; decimalLatitude: 41.9644829; decimalLongitude: 12.436101; geodeticDatum: WGS84; coordinatePrecision: 0.0002; **Identification:** identifiedBy: M. Mei; **Event:** eventDate: 2022-05-27; **Record Level:** collectionID: UR3
- **Type status:** Other material. **Occurrence:** catalogNumber: A1836, A1838, A1839; recordedBy: L. Fortini; individualCount: 3; sex: females; lifeStage: adult; occurrenceID: 44B7CBE1-6CD8-55AF-8324-31773F33FF60; **Taxon:** scientificName: Halictus (Hexataenites) scabiosae (Rossi, 1790); order: Hymenoptera; family: Halictidae; genus: Halictus; subgenus: Hexataenites; specificEpithet: scabiosae; scientificNameAuthorship: (Rossi, 1790); **Location:** country: Italy; countryCode: IT; stateProvince: Roma; locality: Riserva Naturale Laurentino-Acqua Acetosa; decimalLatitude: 41.8079275; decimalLongitude: 12.4685548; geodeticDatum: WGS84; coordinatePrecision: 0.0002; **Identification:** identifiedBy: M. Mei; **Event:** eventDate: 2022-07-17; **Record Level:** collectionID: UR3
- **Type status:** Other material. **Occurrence:** catalogNumber: A1882, A1892; recordedBy: L. Fortini; individualCount: 2; sex: females; lifeStage: adult; occurrenceID: 8E9C73EF-213F-5E1F-9647-A8580AA71A83; **Taxon:** scientificName: Halictus (Hexataenites) scabiosae (Rossi, 1790); order: Hymenoptera; family: Halictidae; genus: Halictus; subgenus: Hexataenites; specificEpithet: scabiosae; scientificNameAuthorship: (Rossi, 1790); **Location:** country: Italy; countryCode: IT; stateProvince: Roma; locality: Riserva Naturale Laurentino-Acqua Acetosa; decimalLatitude: 41.8079275; decimalLongitude: 12.4685548; geodeticDatum: WGS84; coordinatePrecision: 0.0002; **Identification:** identifiedBy: M. Mei; **Event:** eventDate: 2022-05-12; **Record Level:** collectionID: UR3
- **Type status:** Other material. **Occurrence:** catalogNumber: A1867, A1868, A1894, A1906, A1907, A1910, A1911, A1916; recordedBy: L. Fortini; individualCount: 8; sex: females; lifeStage: adult; occurrenceID: 51A2B1C8-50DF-5446-9AB2-AD099A512D67; **Taxon:** scientificName: Halictus (Hexataenites) scabiosae (Rossi, 1790); order: Hymenoptera; family: Halictidae; genus: Halictus; subgenus: Hexataenites; specificEpithet: scabiosae; scientificNameAuthorship: (Rossi, 1790); **Location:** country: Italy; countryCode: IT; stateProvince: Roma; locality: Riserva Naturale di Monte Mario; decimalLatitude: 41.9386215; decimalLongitude: 12.4546223; geodeticDatum: WGS84; coordinatePrecision: 0.0002; **Identification:** identifiedBy: M. Mei; **Event:** eventDate: 2022-04-20; **Record Level:** collectionID: UR3
- **Type status:** Other material. **Occurrence:** catalogNumber: A1830, A1866, A1875, A1895, A1896; recordedBy: L. Fortini; individualCount: 5; sex: females; lifeStage: adult; occurrenceID: F1DED13B-28F4-5F3B-B6B9-F45691961668; **Taxon:** scientificName: Halictus (Hexataenites) scabiosae (Rossi, 1790); order: Hymenoptera; family: Halictidae; genus: Halictus; subgenus: Hexataenites; specificEpithet: scabiosae; scientificNameAuthorship: (Rossi, 1790); **Location:** country: Italy; countryCode: IT; stateProvince: Roma; locality: Riserva Naturale di Monte Mario; decimalLatitude: 41.9386215; decimalLongitude: 12.4546223; geodeticDatum: WGS84; coordinatePrecision: 0.0002; **Identification:** identifiedBy: M. Mei; **Event:** eventDate: 2022-05-20; **Record Level:** collectionID: UR3
- **Type status:** Other material. **Occurrence:** catalogNumber: A1850; recordedBy: L. Fortini; individualCount: 1; sex: female; lifeStage: adult; occurrenceID: 031AE32D-F953-5190-A5FA-098EC4CE5D1E; **Taxon:** scientificName: Halictus (Hexataenites) scabiosae (Rossi, 1790); order: Hymenoptera; family: Halictidae; genus: Halictus; subgenus: Hexataenites; specificEpithet: scabiosae; scientificNameAuthorship: (Rossi, 1790); **Location:** country: Italy; countryCode: IT; stateProvince: Roma; locality: Riserva Naturale di Monte Mario; decimalLatitude: 41.9386215; decimalLongitude: 12.4546223; geodeticDatum: WGS84; coordinatePrecision: 0.0002; **Identification:** identifiedBy: M. Mei; **Event:** eventDate: 2022-06-19; **Record Level:** collectionID: UR3
- **Type status:** Other material. **Occurrence:** catalogNumber: A1883, A1884, A1885, A1891, A1897, A1899, A1908, A1912, A1914; recordedBy: L. Fortini; individualCount: 9; sex: females; lifeStage: adult; occurrenceID: 26203007-ABE7-5326-9801-8CE40270A96F; **Taxon:** scientificName: Halictus (Hexataenites) scabiosae (Rossi, 1790); order: Hymenoptera; family: Halictidae; genus: Halictus; subgenus: Hexataenites; specificEpithet: scabiosae; scientificNameAuthorship: (Rossi, 1790); **Location:** country: Italy; countryCode: IT; stateProvince: Roma; locality: Riserva Naturale Tenuta dei Massimi 1; decimalLatitude: 41.8532859; decimalLongitude: 12.3842322; geodeticDatum: WGS84; coordinatePrecision: 0.0002; **Identification:** identifiedBy: M. Mei; **Event:** eventDate: 2022-04-23; **Record Level:** collectionID: UR3
- **Type status:** Other material. **Occurrence:** catalogNumber: A1835, A1856, A1857, A1861, A1863; recordedBy: L. Fortini; individualCount: 5; sex: females; lifeStage: adult; occurrenceID: BE5E8E12-33EB-5573-85B1-3D2B32AF0A9D; **Taxon:** scientificName: Halictus (Hexataenites) scabiosae (Rossi, 1790); order: Hymenoptera; family: Halictidae; genus: Halictus; subgenus: Hexataenites; specificEpithet: scabiosae; scientificNameAuthorship: (Rossi, 1790); **Location:** country: Italy; countryCode: IT; stateProvince: Roma; locality: Riserva Naturale Tenuta dei Massimi 1; decimalLatitude: 41.8532859; decimalLongitude: 12.3842322; geodeticDatum: WGS84; coordinatePrecision: 0.0002; **Identification:** identifiedBy: M. Mei; **Event:** eventDate: 2022-06-27; **Record Level:** collectionID: UR3
- **Type status:** Other material. **Occurrence:** catalogNumber: A1874, A1877, A1878, A1888; recordedBy: L. Fortini; individualCount: 4; sex: females; lifeStage: adult; occurrenceID: EB4EE296-475C-5979-A234-392B2AA5FC92; **Taxon:** scientificName: Halictus (Hexataenites) scabiosae (Rossi, 1790); order: Hymenoptera; family: Halictidae; genus: Halictus; subgenus: Hexataenites; specificEpithet: scabiosae; scientificNameAuthorship: (Rossi, 1790); **Location:** country: Italy; countryCode: IT; stateProvince: Roma; locality: Riserva Naturale Tenuta dei Massimi 1; decimalLatitude: 41.8532859; decimalLongitude: 12.3842322; geodeticDatum: WGS84; coordinatePrecision: 0.0002; **Identification:** identifiedBy: M. Mei; **Event:** eventDate: 2022-06-01; **Record Level:** collectionID: UR3
- **Type status:** Other material. **Occurrence:** catalogNumber: A1658, A1676, A1812, A1820, A1822, A1902, A1905; recordedBy: L. Fortini; individualCount: 7; sex: 2 males, 5 females; lifeStage: adult; occurrenceID: 37A48E68-56EF-5D25-8200-111DD64F4B45; **Taxon:** scientificName: Halictus (Hexataenites) scabiosae (Rossi, 1790); order: Hymenoptera; family: Halictidae; genus: Halictus; subgenus: Hexataenites; specificEpithet: scabiosae; scientificNameAuthorship: (Rossi, 1790); **Location:** country: Italy; countryCode: IT; stateProvince: Roma; locality: Riserva Naturale Tenuta dei Massimi 1; decimalLatitude: 41.8532859; decimalLongitude: 12.3842322; geodeticDatum: WGS84; coordinatePrecision: 0.0002; **Identification:** identifiedBy: M. Mei; **Event:** eventDate: 2022-07-28; **Record Level:** collectionID: UR3
- **Type status:** Other material. **Occurrence:** catalogNumber: A1893; recordedBy: L. Fortini; individualCount: 1; sex: female; lifeStage: adult; occurrenceID: 9545DA73-EFBB-56BD-BD49-FE2DE9CA0732; **Taxon:** scientificName: Halictus (Hexataenites) scabiosae (Rossi, 1790); order: Hymenoptera; family: Halictidae; genus: Halictus; subgenus: Hexataenites; specificEpithet: scabiosae; scientificNameAuthorship: (Rossi, 1790); **Location:** country: Italy; countryCode: IT; stateProvince: Roma; locality: Riserva Naturale Tenuta dei Massimi 2; decimalLatitude: 41.8316516; decimalLongitude: 12.3999927; geodeticDatum: WGS84; coordinatePrecision: 0.0002; **Identification:** identifiedBy: M. Mei; **Event:** eventDate: 2022-05-04; **Record Level:** collectionID: UR3
- **Type status:** Other material. **Occurrence:** catalogNumber: A1909, A1915; recordedBy: L. Fortini; individualCount: 2; sex: females; lifeStage: adult; occurrenceID: 9DB750CB-9056-5A73-BF60-3F1E2C2554DD; **Taxon:** scientificName: Halictus (Hexataenites) scabiosae (Rossi, 1790); order: Hymenoptera; family: Halictidae; genus: Halictus; subgenus: Hexataenites; specificEpithet: scabiosae; scientificNameAuthorship: (Rossi, 1790); **Location:** country: Italy; countryCode: IT; stateProvince: Roma; locality: Riserva Naturale Valle dell'Aniene 1; decimalLatitude: 41.9345179; decimalLongitude: 12.5453096; geodeticDatum: WGS84; coordinatePrecision: 0.0002; **Identification:** identifiedBy: M. Mei; **Event:** eventDate: 2022-04-28; **Record Level:** collectionID: UR3
- **Type status:** Other material. **Occurrence:** catalogNumber: A1644, A1650, A1655, A1660, A1665, A1815, A1818, A1823, A1851, A1852, A1853, A1854, A1855, A1859, A1860, A1864, A1900; recordedBy: L. Fortini; individualCount: 17; sex: 5 males, 12 females; lifeStage: adult; occurrenceID: E93B5203-775B-55B6-9170-1B7ABC5067E1; **Taxon:** scientificName: Halictus (Hexataenites) scabiosae (Rossi, 1790); order: Hymenoptera; family: Halictidae; genus: Halictus; subgenus: Hexataenites; specificEpithet: scabiosae; scientificNameAuthorship: (Rossi, 1790); **Location:** country: Italy; countryCode: IT; stateProvince: Roma; locality: Riserva Naturale Valle dell'Aniene 1; decimalLatitude: 41.9345179; decimalLongitude: 12.5453096; geodeticDatum: WGS84; coordinatePrecision: 0.0002; **Identification:** identifiedBy: M. Mei; **Event:** eventDate: 2022-07-01; **Record Level:** collectionID: UR3
- **Type status:** Other material. **Occurrence:** catalogNumber: A1832, A1834, A1837, A1862, A1873; recordedBy: L. Fortini; individualCount: 5; sex: females; lifeStage: adult; occurrenceID: FB502CB0-8D94-5F4E-A638-7DF4EEBAF31B; **Taxon:** scientificName: Halictus (Hexataenites) scabiosae (Rossi, 1790); order: Hymenoptera; family: Halictidae; genus: Halictus; subgenus: Hexataenites; specificEpithet: scabiosae; scientificNameAuthorship: (Rossi, 1790); **Location:** country: Italy; countryCode: IT; stateProvince: Roma; locality: Riserva Naturale Valle dell'Aniene 2; decimalLatitude: 41.928752; decimalLongitude: 12.5562962; geodeticDatum: WGS84; coordinatePrecision: 0.0002; **Identification:** identifiedBy: M. Mei; **Event:** eventDate: 2022-07-01; **Record Level:** collectionID: UR3
- **Type status:** Other material. **Occurrence:** catalogNumber: A1913; recordedBy: L. Fortini; individualCount: 1; sex: female; lifeStage: adult; occurrenceID: 047FA7CF-225B-5211-A358-E92766A11237; **Taxon:** scientificName: Halictus (Hexataenites) scabiosae (Rossi, 1790); order: Hymenoptera; family: Halictidae; genus: Halictus; subgenus: Hexataenites; specificEpithet: scabiosae; scientificNameAuthorship: (Rossi, 1790); **Location:** country: Italy; countryCode: IT; stateProvince: Roma; locality: Riserva Naturale Valle dei Casali 1; decimalLatitude: 41.8710627; decimalLongitude: 12.4336809; geodeticDatum: WGS84; coordinatePrecision: 0.0002; **Identification:** identifiedBy: M. Mei; **Event:** eventDate: 2022-04-07; **Record Level:** collectionID: UR3
- **Type status:** Other material. **Occurrence:** catalogNumber: A1649, A1871, A1876; recordedBy: L. Fortini; individualCount: 3; sex: females; lifeStage: adult; occurrenceID: 23126440-87CF-5C69-A052-27B71BD3FC08; **Taxon:** scientificName: Halictus (Hexataenites) scabiosae (Rossi, 1790); order: Hymenoptera; family: Halictidae; genus: Halictus; subgenus: Hexataenites; specificEpithet: scabiosae; scientificNameAuthorship: (Rossi, 1790); **Location:** country: Italy; countryCode: IT; stateProvince: Roma; locality: Riserva Naturale Valle dei Casali 1; decimalLatitude: 41.8710627; decimalLongitude: 12.4336809; geodeticDatum: WGS84; coordinatePrecision: 0.0002; **Identification:** identifiedBy: M. Mei; **Event:** eventDate: 2022-06-18; **Record Level:** collectionID: UR3
- **Type status:** Other material. **Occurrence:** catalogNumber: A1643, A1645, A1833; recordedBy: L. Fortini; individualCount: 3; sex: 2 males, 1 female; lifeStage: adult; occurrenceID: B927E666-AA7E-500D-BC81-ACFF4FBA6633; **Taxon:** scientificName: Halictus (Hexataenites) scabiosae (Rossi, 1790); order: Hymenoptera; family: Halictidae; genus: Halictus; subgenus: Hexataenites; specificEpithet: scabiosae; scientificNameAuthorship: (Rossi, 1790); **Location:** country: Italy; countryCode: IT; stateProvince: Roma; locality: Riserva Naturale Valle dei Casali 1; decimalLatitude: 41.8710627; decimalLongitude: 12.4336809; geodeticDatum: WGS84; coordinatePrecision: 0.0002; **Identification:** identifiedBy: M. Mei; **Event:** eventDate: 2022-07-13; **Record Level:** collectionID: UR3
- **Type status:** Other material. **Occurrence:** catalogNumber: A1813; recordedBy: L. Fortini; individualCount: 1; sex: female; lifeStage: adult; occurrenceID: 65F8CA46-848B-52D7-AB17-43B51CA49B16; **Taxon:** scientificName: Halictus (Hexataenites) scabiosae (Rossi, 1790); order: Hymenoptera; family: Halictidae; genus: Halictus; subgenus: Hexataenites; specificEpithet: scabiosae; scientificNameAuthorship: (Rossi, 1790); **Location:** country: Italy; countryCode: IT; stateProvince: Roma; locality: Riserva Naturale Valle dei Casali 2; decimalLatitude: 41.8596887; decimalLongitude: 12.4355075; geodeticDatum: WGS84; coordinatePrecision: 0.0002; **Identification:** identifiedBy: M. Mei; **Event:** eventDate: 2022-06-18; **Record Level:** collectionID: UR3
- **Type status:** Other material. **Occurrence:** catalogNumber: A1705, A1816, A1819, A1821, A1825, A1826, A1829, A1843, A1847; recordedBy: L. Fortini; individualCount: 9; sex: females; lifeStage: adult; occurrenceID: 3C97B979-520B-543F-9E95-ECACA4A6491D; **Taxon:** scientificName: Halictus (Hexataenites) scabiosae (Rossi, 1790); order: Hymenoptera; family: Halictidae; genus: Halictus; subgenus: Hexataenites; specificEpithet: scabiosae; scientificNameAuthorship: (Rossi, 1790); **Location:** country: Italy; countryCode: IT; stateProvince: Roma; locality: Riserva Naturale Valle dei Casali 2; decimalLatitude: 41.8596887; decimalLongitude: 12.4355075; geodeticDatum: WGS84; coordinatePrecision: 0.0002; **Identification:** identifiedBy: M. Mei; **Event:** eventDate: 2022-07-13; **Record Level:** collectionID: UR3
- **Type status:** Other material. **Occurrence:** catalogNumber: A1974, A1975, A1980; recordedBy: L. Fortini; individualCount: 3; sex: females; lifeStage: adult; occurrenceID: 99E4F5DC-A053-56E1-A489-7F274A592F68; **Taxon:** scientificName: Halictus (Hexataenites) scabiosae (Rossi, 1790); order: Hymenoptera; family: Halictidae; genus: Halictus; subgenus: Hexataenites; specificEpithet: scabiosae; scientificNameAuthorship: (Rossi, 1790); **Location:** country: Italy; countryCode: IT; stateProvince: Roma; locality: Riserva Regionale dell'Appia Antica 1; decimalLatitude: 41.8623941; decimalLongitude: 12.524863; geodeticDatum: WGS84; coordinatePrecision: 0.0002; **Identification:** identifiedBy: M. Mei; **Event:** eventDate: 2022-04-19; **Record Level:** collectionID: UR3
- **Type status:** Other material. **Occurrence:** catalogNumber: A2073, A2076, A2077; recordedBy: L. Fortini; individualCount: 3; sex: 1 male, 2 females; lifeStage: adult; occurrenceID: EC50ADC4-2819-5BB4-BD35-85A6FFF17BBC; **Taxon:** scientificName: Halictus (Hexataenites) scabiosae (Rossi, 1790); order: Hymenoptera; family: Halictidae; genus: Halictus; subgenus: Hexataenites; specificEpithet: scabiosae; scientificNameAuthorship: (Rossi, 1790); **Location:** country: Italy; countryCode: IT; stateProvince: Roma; locality: Riserva Naturale di Monte Mario; decimalLatitude: 41.9386215; decimalLongitude: 12.4546223; geodeticDatum: WGS84; coordinatePrecision: 0.0002; **Identification:** identifiedBy: M. Mei; **Event:** eventDate: 2022-07-24; **Record Level:** collectionID: UR3
- **Type status:** Other material. **Occurrence:** catalogNumber: A2085, A2086, A2087, A2088; recordedBy: L. Fortini; individualCount: 4; sex: 2 males, 2 females; lifeStage: adult; occurrenceID: E1AA37B1-C5D8-5DF7-9498-92C1228388E5; **Taxon:** scientificName: Halictus (Hexataenites) scabiosae (Rossi, 1790); order: Hymenoptera; family: Halictidae; genus: Halictus; subgenus: Hexataenites; specificEpithet: scabiosae; scientificNameAuthorship: (Rossi, 1790); **Location:** country: Italy; countryCode: IT; stateProvince: Roma; locality: Riserva Regionale dell'Appia Antica 2; decimalLatitude: 41.8402564; decimalLongitude: 12.532773; geodeticDatum: WGS84; coordinatePrecision: 0.0002; **Identification:** identifiedBy: M. Mei; **Event:** eventDate: 2022-08-06; **Record Level:** collectionID: UR3
- **Type status:** Other material. **Occurrence:** catalogNumber: A2092, A2093, A2094; recordedBy: L. Fortini; individualCount: 3; sex: 1 male, 2 females; lifeStage: adult; occurrenceID: 0ECB8F25-099E-5626-96E4-D8FF44580690; **Taxon:** scientificName: Halictus (Hexataenites) scabiosae (Rossi, 1790); order: Hymenoptera; family: Halictidae; genus: Halictus; subgenus: Hexataenites; specificEpithet: scabiosae; scientificNameAuthorship: (Rossi, 1790); **Location:** country: Italy; countryCode: IT; stateProvince: Roma; locality: Riserva Naturale Valle dell'Aniene 1; decimalLatitude: 41.9345179; decimalLongitude: 12.5453096; geodeticDatum: WGS84; coordinatePrecision: 0.0002; **Identification:** identifiedBy: M. Mei; **Event:** eventDate: 2022-08-03; **Record Level:** collectionID: UR3
- **Type status:** Other material. **Occurrence:** catalogNumber: A2123, A2124, A2137; recordedBy: L. Fortini; individualCount: 3; sex: 1 male, 2 females; lifeStage: adult; occurrenceID: BC02EA3B-99FC-56A9-9077-B3DFBB974F9B; **Taxon:** scientificName: Halictus (Hexataenites) scabiosae (Rossi, 1790); order: Hymenoptera; family: Halictidae; genus: Halictus; subgenus: Hexataenites; specificEpithet: scabiosae; scientificNameAuthorship: (Rossi, 1790); **Location:** country: Italy; countryCode: IT; stateProvince: Roma; locality: Riserva Naturale Valle dell'Aniene 2; decimalLatitude: 41.928752; decimalLongitude: 12.5562962; geodeticDatum: WGS84; coordinatePrecision: 0.0002; **Identification:** identifiedBy: M. Mei; **Event:** eventDate: 2022-08-03; **Record Level:** collectionID: UR3

#### Lasioglossum albocinctum

(Lucas, 1849)

918F3EF3-E15B-5B1E-9E8C-6A82F485E58A

##### Materials

- **Type status:** Other material. **Occurrence:** catalogNumber: A1638, A1771; recordedBy: L. Fortini; individualCount: 2; sex: 1 male, 1 female; lifeStage: adult; occurrenceID: C09C5934-6CA4-5C25-BA96-759DD174CD8C; **Taxon:** scientificName: Lasioglossum (Leuchalictus) albocinctum (Lucas, 1849); order: Hymenoptera; family: Halictidae; genus: Lasioglossum; subgenus: Leuchalictus; specificEpithet: albocinctum; scientificNameAuthorship: (Lucas, 1849); **Location:** country: Italy; countryCode: IT; stateProvince: Roma; locality: Riserva Naturale dell'Acquafredda; decimalLatitude: 41.8928408; decimalLongitude: 12.39932; geodeticDatum: WGS84; coordinatePrecision: 0.0002; **Identification:** identifiedBy: M. Mei; **Event:** eventDate: 2022-07-12; **Record Level:** collectionID: UR3
- **Type status:** Other material. **Occurrence:** catalogNumber: A1777; recordedBy: L. Fortini; individualCount: 1; sex: female; lifeStage: adult; occurrenceID: B144129E-5D62-50EE-9175-3BDF698BF0AA; **Taxon:** scientificName: Lasioglossum (Leuchalictus) albocinctum (Lucas, 1849); order: Hymenoptera; family: Halictidae; genus: Lasioglossum; subgenus: Leuchalictus; specificEpithet: albocinctum; scientificNameAuthorship: (Lucas, 1849); **Location:** country: Italy; countryCode: IT; stateProvince: Roma; locality: Riserva Naturale dell'Acquafredda; decimalLatitude: 41.8928408; decimalLongitude: 12.39932; geodeticDatum: WGS84; coordinatePrecision: 0.0002; **Identification:** identifiedBy: M. Mei; **Event:** eventDate: 2022-06-10; **Record Level:** collectionID: UR3
- **Type status:** Other material. **Occurrence:** catalogNumber: A1636, A1776; recordedBy: L. Fortini; individualCount: 2; sex: 1 male, 1 female; lifeStage: adult; occurrenceID: E2AA1525-E7D5-52AB-9E9B-3DBB92F3A421; **Taxon:** scientificName: Lasioglossum (Leuchalictus) albocinctum (Lucas, 1849); order: Hymenoptera; family: Halictidae; genus: Lasioglossum; subgenus: Leuchalictus; specificEpithet: albocinctum; scientificNameAuthorship: (Lucas, 1849); **Location:** country: Italy; countryCode: IT; stateProvince: Roma; locality: Riserva Naturale dell'Insugherata 1; decimalLatitude: 41.9555045; decimalLongitude: 12.4292321; geodeticDatum: WGS84; coordinatePrecision: 0.0002; **Identification:** identifiedBy: M. Mei; **Event:** eventDate: 2022-07-30; **Record Level:** collectionID: UR3
- **Type status:** Other material. **Occurrence:** catalogNumber: A1779; recordedBy: L. Fortini; individualCount: 1; sex: female; lifeStage: adult; occurrenceID: 92CF26C4-E8E2-559D-8172-0CFA2F4ED30F; **Taxon:** scientificName: Lasioglossum (Leuchalictus) albocinctum (Lucas, 1849); order: Hymenoptera; family: Halictidae; genus: Lasioglossum; subgenus: Leuchalictus; specificEpithet: albocinctum; scientificNameAuthorship: (Lucas, 1849); **Location:** country: Italy; countryCode: IT; stateProvince: Roma; locality: Riserva Naturale dell'Insugherata 2; decimalLatitude: 41.9599247; decimalLongitude: 12.433852; geodeticDatum: WGS84; coordinatePrecision: 0.0002; **Identification:** identifiedBy: M. Mei; **Event:** eventDate: 2022-09-01; **Record Level:** collectionID: UR3
- **Type status:** Other material. **Occurrence:** catalogNumber: A1780; recordedBy: L. Fortini; individualCount: 1; sex: female; lifeStage: adult; occurrenceID: EFF872B6-9682-52EF-8950-FC6F03FC3598; **Taxon:** scientificName: Lasioglossum (Leuchalictus) albocinctum (Lucas, 1849); order: Hymenoptera; family: Halictidae; genus: Lasioglossum; subgenus: Leuchalictus; specificEpithet: albocinctum; scientificNameAuthorship: (Lucas, 1849); **Location:** country: Italy; countryCode: IT; stateProvince: Roma; locality: Riserva Naturale dell'Insugherata 2; decimalLatitude: 41.9599247; decimalLongitude: 12.433852; geodeticDatum: WGS84; coordinatePrecision: 0.0002; **Identification:** identifiedBy: M. Mei; **Event:** eventDate: 2022-06-24; **Record Level:** collectionID: UR3
- **Type status:** Other material. **Occurrence:** catalogNumber: A1769; recordedBy: L. Fortini; individualCount: 1; sex: female; lifeStage: adult; occurrenceID: 0531753C-B4E6-52CB-9BC8-B395BD6C52D5; **Taxon:** scientificName: Lasioglossum (Leuchalictus) albocinctum (Lucas, 1849); order: Hymenoptera; family: Halictidae; genus: Lasioglossum; subgenus: Leuchalictus; specificEpithet: albocinctum; scientificNameAuthorship: (Lucas, 1849); **Location:** country: Italy; countryCode: IT; stateProvince: Roma; locality: Riserva Naturale Laurentino-Acqua Acetosa; decimalLatitude: 41.8079275; decimalLongitude: 12.4685548; geodeticDatum: WGS84; coordinatePrecision: 0.0002; **Identification:** identifiedBy: M. Mei; **Event:** eventDate: 2022-08-21; **Record Level:** collectionID: UR3
- **Type status:** Other material. **Occurrence:** catalogNumber: A1773, A1774, A1782; recordedBy: L. Fortini; individualCount: 3; sex: females; lifeStage: adult; occurrenceID: C13430FE-0F61-555B-A071-626217D82C49; **Taxon:** scientificName: Lasioglossum (Leuchalictus) albocinctum (Lucas, 1849); order: Hymenoptera; family: Halictidae; genus: Lasioglossum; subgenus: Leuchalictus; specificEpithet: albocinctum; scientificNameAuthorship: (Lucas, 1849); **Location:** country: Italy; countryCode: IT; stateProvince: Roma; locality: Riserva Naturale di Monte Mario; decimalLatitude: 41.9386215; decimalLongitude: 12.4546223; geodeticDatum: WGS84; coordinatePrecision: 0.0002; **Identification:** identifiedBy: M. Mei; **Event:** eventDate: 2022-06-19; **Record Level:** collectionID: UR3
- **Type status:** Other material. **Occurrence:** catalogNumber: A1778; recordedBy: L. Fortini; individualCount: 1; sex: female; lifeStage: adult; occurrenceID: DCE7DEA5-F0C7-539F-A1DF-6448906E5A50; **Taxon:** scientificName: Lasioglossum (Leuchalictus) albocinctum (Lucas, 1849); order: Hymenoptera; family: Halictidae; genus: Lasioglossum; subgenus: Leuchalictus; specificEpithet: albocinctum; scientificNameAuthorship: (Lucas, 1849); **Location:** country: Italy; countryCode: IT; stateProvince: Roma; locality: Riserva Naturale di Monte Mario; decimalLatitude: 41.9386215; decimalLongitude: 12.4546223; geodeticDatum: WGS84; coordinatePrecision: 0.0002; **Identification:** identifiedBy: M. Mei; **Event:** eventDate: 2022-05-20; **Record Level:** collectionID: UR3
- **Type status:** Other material. **Occurrence:** catalogNumber: A1634, A1639, A1640, A1768; recordedBy: L. Fortini; individualCount: 4; sex: 3 males, 1 female; lifeStage: adult; occurrenceID: F17DD5A6-AB1F-5232-83FC-ED016182E11A; **Taxon:** scientificName: Lasioglossum (Leuchalictus) albocinctum (Lucas, 1849); order: Hymenoptera; family: Halictidae; genus: Lasioglossum; subgenus: Leuchalictus; specificEpithet: albocinctum; scientificNameAuthorship: (Lucas, 1849); **Location:** country: Italy; countryCode: IT; stateProvince: Roma; locality: Riserva Naturale Tenuta dei Massimi 2; decimalLatitude: 41.8316516; decimalLongitude: 12.3999927; geodeticDatum: WGS84; coordinatePrecision: 0.0002; **Identification:** identifiedBy: M. Mei; **Event:** eventDate: 2022-07-28; **Record Level:** collectionID: UR3
- **Type status:** Other material. **Occurrence:** catalogNumber: A1785; recordedBy: L. Fortini; individualCount: 1; sex: female; lifeStage: adult; occurrenceID: 5857111B-F271-5FCD-901C-F96BF3896BC2; **Taxon:** scientificName: Lasioglossum (Leuchalictus) albocinctum (Lucas, 1849); order: Hymenoptera; family: Halictidae; genus: Lasioglossum; subgenus: Leuchalictus; specificEpithet: albocinctum; scientificNameAuthorship: (Lucas, 1849); **Location:** country: Italy; countryCode: IT; stateProvince: Roma; locality: Riserva Naturale Tenuta dei Massimi 2; decimalLatitude: 41.8316516; decimalLongitude: 12.3999927; geodeticDatum: WGS84; coordinatePrecision: 0.0002; **Identification:** identifiedBy: M. Mei; **Event:** eventDate: 2022-06-27; **Record Level:** collectionID: UR3
- **Type status:** Other material. **Occurrence:** catalogNumber: A1775; recordedBy: L. Fortini; individualCount: 1; sex: female; lifeStage: adult; occurrenceID: DFC31F53-08B5-544F-90B5-BB7C65E7C578; **Taxon:** scientificName: Lasioglossum (Leuchalictus) albocinctum (Lucas, 1849); order: Hymenoptera; family: Halictidae; genus: Lasioglossum; subgenus: Leuchalictus; specificEpithet: albocinctum; scientificNameAuthorship: (Lucas, 1849); **Location:** country: Italy; countryCode: IT; stateProvince: Roma; locality: Riserva Naturale Valle dell'Aniene 1; decimalLatitude: 41.9345179; decimalLongitude: 12.5453096; geodeticDatum: WGS84; coordinatePrecision: 0.0002; **Identification:** identifiedBy: M. Mei; **Event:** eventDate: 2022-07-01; **Record Level:** collectionID: UR3
- **Type status:** Other material. **Occurrence:** catalogNumber: A1781, A1783; recordedBy: L. Fortini; individualCount: 2; sex: females; lifeStage: adult; occurrenceID: 0C9DD0F8-E943-5FDF-978E-4108456F3EC5; **Taxon:** scientificName: Lasioglossum (Leuchalictus) albocinctum (Lucas, 1849); order: Hymenoptera; family: Halictidae; genus: Lasioglossum; subgenus: Leuchalictus; specificEpithet: albocinctum; scientificNameAuthorship: (Lucas, 1849); **Location:** country: Italy; countryCode: IT; stateProvince: Roma; locality: Riserva Naturale Valle dell'Aniene 2; decimalLatitude: 41.928752; decimalLongitude: 12.5562962; geodeticDatum: WGS84; coordinatePrecision: 0.0002; **Identification:** identifiedBy: M. Mei; **Event:** eventDate: 2022-06-05; **Record Level:** collectionID: UR3
- **Type status:** Other material. **Occurrence:** catalogNumber: A1633, A1635, A1637; recordedBy: L. Fortini; individualCount: 3; sex: males; lifeStage: adult; occurrenceID: C1040239-9523-5852-B410-019F9AD4E7A0; **Taxon:** scientificName: Lasioglossum (Leuchalictus) albocinctum (Lucas, 1849); order: Hymenoptera; family: Halictidae; genus: Lasioglossum; subgenus: Leuchalictus; specificEpithet: albocinctum; scientificNameAuthorship: (Lucas, 1849); **Location:** country: Italy; countryCode: IT; stateProvince: Roma; locality: Riserva Naturale Valle dell'Aniene 2; decimalLatitude: 41.928752; decimalLongitude: 12.5562962; geodeticDatum: WGS84; coordinatePrecision: 0.0002; **Identification:** identifiedBy: M. Mei; **Event:** eventDate: 2022-07-01; **Record Level:** collectionID: UR3
- **Type status:** Other material. **Occurrence:** catalogNumber: A1770; recordedBy: L. Fortini; individualCount: 1; sex: female; lifeStage: adult; occurrenceID: DBE8DD1C-7B06-5577-B075-C57BC93E3AC3; **Taxon:** scientificName: Lasioglossum (Leuchalictus) albocinctum (Lucas, 1849); order: Hymenoptera; family: Halictidae; genus: Lasioglossum; subgenus: Leuchalictus; specificEpithet: albocinctum; scientificNameAuthorship: (Lucas, 1849); **Location:** country: Italy; countryCode: IT; stateProvince: Roma; locality: Riserva Naturale Valle dei Casali 2; decimalLatitude: 41.8596887; decimalLongitude: 12.4355075; geodeticDatum: WGS84; coordinatePrecision: 0.0002; **Identification:** identifiedBy: M. Mei; **Event:** eventDate: 2022-07-13; **Record Level:** collectionID: UR3
- **Type status:** Other material. **Occurrence:** catalogNumber: A1772; recordedBy: L. Fortini; individualCount: 1; sex: female; lifeStage: adult; occurrenceID: 1AA9C545-6C21-52CF-96A5-DC1E154751A4; **Taxon:** scientificName: Lasioglossum (Leuchalictus) albocinctum (Lucas, 1849); order: Hymenoptera; family: Halictidae; genus: Lasioglossum; subgenus: Leuchalictus; specificEpithet: albocinctum; scientificNameAuthorship: (Lucas, 1849); **Location:** country: Italy; countryCode: IT; stateProvince: Roma; locality: Riserva Naturale Valle dei Casali 2; decimalLatitude: 41.8596887; decimalLongitude: 12.4355075; geodeticDatum: WGS84; coordinatePrecision: 0.0002; **Identification:** identifiedBy: M. Mei; **Event:** eventDate: 2022-05-14; **Record Level:** collectionID: UR3
- **Type status:** Other material. **Occurrence:** catalogNumber: A1784; recordedBy: L. Fortini; individualCount: 1; sex: female; lifeStage: adult; occurrenceID: 5D3A0698-E01C-55C8-868E-39425DF3D1BB; **Taxon:** scientificName: Lasioglossum (Leuchalictus) albocinctum (Lucas, 1849); order: Hymenoptera; family: Halictidae; genus: Lasioglossum; subgenus: Leuchalictus; specificEpithet: albocinctum; scientificNameAuthorship: (Lucas, 1849); **Location:** country: Italy; countryCode: IT; stateProvince: Roma; locality: Riserva Naturale Valle dei Casali 1; decimalLatitude: 41.8710627; decimalLongitude: 12.4336809; geodeticDatum: WGS84; coordinatePrecision: 0.0002; **Identification:** identifiedBy: M. Mei; **Event:** eventDate: 2022-05-14; **Record Level:** collectionID: UR3
- **Type status:** Other material. **Occurrence:** catalogNumber: A2078; recordedBy: L. Fortini; individualCount: 1; sex: female; lifeStage: adult; occurrenceID: 354015E3-A413-50AA-9FA1-198919C937D2; **Taxon:** scientificName: Lasioglossum (Leuchalictus) albocinctum (Lucas, 1849); order: Hymenoptera; family: Halictidae; genus: Lasioglossum; subgenus: Leuchalictus; specificEpithet: albocinctum; scientificNameAuthorship: (Lucas, 1849); **Location:** country: Italy; countryCode: IT; stateProvince: Roma; locality: Riserva Regionale dell'Appia Antica 2; decimalLatitude: 41.8402564; decimalLongitude: 12.532773; geodeticDatum: WGS84; coordinatePrecision: 0.0002; **Identification:** identifiedBy: M. Mei; **Event:** eventDate: 2022-08-06; **Record Level:** collectionID: UR3

#### Lasioglossum brevicorne

(Schenck, 1868)

94B17194-C51C-5608-AACA-3F0C95C09A1A

##### Materials

- **Type status:** Other material. **Occurrence:** catalogNumber: A1363, A1365; recordedBy: L. Fortini; individualCount: 2; sex: 1 male, 1 female; lifeStage: adult; occurrenceID: 24E81313-0B0D-5CDA-8B79-B374CB7C7E92; **Taxon:** scientificName: Lasioglossum (Hemihalictus) brevicorne (Schenck, 1868); order: Hymenoptera; family: Halictidae; genus: Lasioglossum; subgenus: Hemihalictus; specificEpithet: brevicorne; scientificNameAuthorship: (Schenck, 1868); **Location:** country: Italy; countryCode: IT; stateProvince: Roma; locality: Riserva Naturale Laurentino-Acqua Acetosa; decimalLatitude: 41.8079275; decimalLongitude: 12.4685548; geodeticDatum: WGS84; coordinatePrecision: 0.0002; **Identification:** identifiedBy: M. Mei; **Event:** eventDate: 2022-06-16; **Record Level:** collectionID: UR3
- **Type status:** Other material. **Occurrence:** catalogNumber: A1557; recordedBy: L. Fortini; individualCount: 1; sex: male; lifeStage: adult; occurrenceID: A5187911-685E-5B64-A995-8F74C4FA2A28; **Taxon:** scientificName: Lasioglossum (Hemihalictus) brevicorne (Schenck, 1868); order: Hymenoptera; family: Halictidae; genus: Lasioglossum; subgenus: Hemihalictus; specificEpithet: brevicorne; scientificNameAuthorship: (Schenck, 1868); **Location:** country: Italy; countryCode: IT; stateProvince: Roma; locality: Riserva Naturale Valle dei Casali 1; decimalLatitude: 41.8710627; decimalLongitude: 12.4336809; geodeticDatum: WGS84; coordinatePrecision: 0.0002; **Identification:** identifiedBy: M. Mei; **Event:** eventDate: 2022-09-18; **Record Level:** collectionID: UR3

#### Lasioglossum corvinum

(Morawitz, 1877)

8DD36E22-BD5A-5581-BFAD-6D2C9F063C36

##### Materials

- **Type status:** Other material. **Occurrence:** catalogNumber: A1262; recordedBy: L. Fortini; individualCount: 1; sex: female; lifeStage: adult; occurrenceID: 06819FCC-A859-5C36-AAE0-9329B8F283BC; **Taxon:** scientificName: Lasioglossum (Hemihalictus) corvinum (Morawitz, 1877); order: Hymenoptera; family: Halictidae; genus: Lasioglossum; subgenus: Hemihalictus; specificEpithet: corvinum; scientificNameAuthorship: (Morawitz, 1877); **Location:** country: Italy; countryCode: IT; stateProvince: Roma; locality: Riserva Naturale Valle dei Casali 1; decimalLatitude: 41.8710627; decimalLongitude: 12.4336809; geodeticDatum: WGS84; coordinatePrecision: 0.0002; **Identification:** identifiedBy: M. Mei; **Event:** eventDate: 2022-05-14; **Record Level:** collectionID: UR3
- **Type status:** Other material. **Occurrence:** catalogNumber: A1368; recordedBy: L. Fortini; individualCount: 1; sex: female; lifeStage: adult; occurrenceID: 14FDE5F3-5A43-5E89-A9DC-9AD6F3477C07; **Taxon:** scientificName: Lasioglossum (Hemihalictus) corvinum (Morawitz, 1877); order: Hymenoptera; family: Halictidae; genus: Lasioglossum; subgenus: Hemihalictus; specificEpithet: corvinum; scientificNameAuthorship: (Morawitz, 1877); **Location:** country: Italy; countryCode: IT; stateProvince: Roma; locality: Riserva Naturale dell'Insugherata 1; decimalLatitude: 41.9555045; decimalLongitude: 12.4292321; geodeticDatum: WGS84; coordinatePrecision: 0.0002; **Identification:** identifiedBy: M. Mei; **Event:** eventDate: 2022-06-24; **Record Level:** collectionID: UR3
- **Type status:** Other material. **Occurrence:** catalogNumber: A1394; recordedBy: L. Fortini; individualCount: 1; sex: female; lifeStage: adult; occurrenceID: 6DA11A98-CA4B-5859-819B-391DF0E86A3F; **Taxon:** scientificName: Lasioglossum (Hemihalictus) corvinum (Morawitz, 1877); order: Hymenoptera; family: Halictidae; genus: Lasioglossum; subgenus: Hemihalictus; specificEpithet: corvinum; scientificNameAuthorship: (Morawitz, 1877); **Location:** country: Italy; countryCode: IT; stateProvince: Roma; locality: Riserva Naturale dell'Insugherata 1; decimalLatitude: 41.9555045; decimalLongitude: 12.4292321; geodeticDatum: WGS84; coordinatePrecision: 0.0002; **Identification:** identifiedBy: M. Mei; **Event:** eventDate: 2022-05-27; **Record Level:** collectionID: UR3
- **Type status:** Other material. **Occurrence:** catalogNumber: A1447; recordedBy: L. Fortini; individualCount: 1; sex: female; lifeStage: adult; occurrenceID: D3162EAE-B385-53AD-BE74-41AC35B01FCF; **Taxon:** scientificName: Lasioglossum (Hemihalictus) corvinum (Morawitz, 1877); order: Hymenoptera; family: Halictidae; genus: Lasioglossum; subgenus: Hemihalictus; specificEpithet: corvinum; scientificNameAuthorship: (Morawitz, 1877); **Location:** country: Italy; countryCode: IT; stateProvince: Roma; locality: Riserva Naturale Tenuta dei Massimi 2; decimalLatitude: 41.8316516; decimalLongitude: 12.3999927; geodeticDatum: WGS84; coordinatePrecision: 0.0002; **Identification:** identifiedBy: M. Mei; **Event:** eventDate: 2022-06-27; **Record Level:** collectionID: UR3

#### Lasioglossum crassepunctatum

(Blüthgen, 1923)

3322EE2A-8F64-5D9C-9C92-3DAF1F0FB68A

##### Materials

- **Type status:** Other material. **Occurrence:** catalogNumber: A1693; recordedBy: L. Fortini; individualCount: 1; sex: male; lifeStage: adult; occurrenceID: 5BFE3DB1-AA1E-544C-A181-B9BC60A02731; **Taxon:** scientificName: Lasioglossum (Hemihalictus) crassepunctatum (Blüthgen, 1923); order: Hymenoptera; family: Halictidae; genus: Lasioglossum; subgenus: Hemihalictus; specificEpithet: crassepunctatum; scientificNameAuthorship: (Blüthgen, 1923); **Location:** country: Italy; countryCode: IT; stateProvince: Roma; locality: Riserva Naturale Tenuta dei Massimi 1; decimalLatitude: 41.8532859; decimalLongitude: 12.3842322; geodeticDatum: WGS84; coordinatePrecision: 0.0002; **Identification:** identifiedBy: M. Mei; **Event:** eventDate: 2022-06-27; **Record Level:** collectionID: UR3

#### Lasioglossum griseolum

(Morawitz, 1872)

E1419437-C812-58A7-BB98-6D8CDC7C8EEE

##### Materials

- **Type status:** Other material. **Occurrence:** catalogNumber: A1279; recordedBy: L. Fortini; individualCount: 1; sex: female; lifeStage: adult; occurrenceID: 424DAC64-F04D-5A14-AE84-5486AA8E6224; **Taxon:** scientificName: Lasioglossum (Hemihalictus) griseolum (Morawitz, 1872); order: Hymenoptera; family: Halictidae; genus: Lasioglossum; subgenus: Hemihalictus; specificEpithet: griseolum; scientificNameAuthorship: (Morawitz, 1872); **Location:** country: Italy; countryCode: IT; stateProvince: Roma; locality: Riserva Naturale dell'Insugherata 3; decimalLatitude: 41.9644829; decimalLongitude: 12.436101; geodeticDatum: WGS84; coordinatePrecision: 0.0002; **Identification:** identifiedBy: M. Mei; **Event:** eventDate: 2022-04-15; **Record Level:** collectionID: UR3
- **Type status:** Other material. **Occurrence:** catalogNumber: A1389; recordedBy: L. Fortini; individualCount: 1; sex: female; lifeStage: adult; occurrenceID: 630ACA09-909D-51FA-ABF3-B584891F96DE; **Taxon:** scientificName: Lasioglossum (Hemihalictus) griseolum (Morawitz, 1872); order: Hymenoptera; family: Halictidae; genus: Lasioglossum; subgenus: Hemihalictus; specificEpithet: griseolum; scientificNameAuthorship: (Morawitz, 1872); **Location:** country: Italy; countryCode: IT; stateProvince: Roma; locality: Riserva Naturale Valle dell'Aniene 2; decimalLatitude: 41.928752; decimalLongitude: 12.5562962; geodeticDatum: WGS84; coordinatePrecision: 0.0002; **Identification:** identifiedBy: M. Mei; **Event:** eventDate: 2022-06-05; **Record Level:** collectionID: UR3
- **Type status:** Other material. **Occurrence:** catalogNumber: A1430; recordedBy: L. Fortini; individualCount: 1; sex: female; lifeStage: adult; occurrenceID: 2CA29805-FBDF-5D5E-B4F5-A2FEC3B85F26; **Taxon:** scientificName: Lasioglossum (Hemihalictus) griseolum (Morawitz, 1872); order: Hymenoptera; family: Halictidae; genus: Lasioglossum; subgenus: Hemihalictus; specificEpithet: griseolum; scientificNameAuthorship: (Morawitz, 1872); **Location:** country: Italy; countryCode: IT; stateProvince: Roma; locality: Riserva Regionale dell'Appia Antica 2; decimalLatitude: 41.8402564; decimalLongitude: 12.532773; geodeticDatum: WGS84; coordinatePrecision: 0.0002; **Identification:** identifiedBy: M. Mei; **Event:** eventDate: 2022-07-07; **Record Level:** collectionID: UR3
- **Type status:** Other material. **Occurrence:** catalogNumber: A1485, A1583; recordedBy: L. Fortini; individualCount: 2; sex: males; lifeStage: adult; occurrenceID: 4A7297D0-F8F0-533E-9591-3866CBB465B6; **Taxon:** scientificName: Lasioglossum (Hemihalictus) griseolum (Morawitz, 1872); order: Hymenoptera; family: Halictidae; genus: Lasioglossum; subgenus: Hemihalictus; specificEpithet: griseolum; scientificNameAuthorship: (Morawitz, 1872); **Location:** country: Italy; countryCode: IT; stateProvince: Roma; locality: Riserva Naturale dell'Acquafredda; decimalLatitude: 41.8928408; decimalLongitude: 12.39932; geodeticDatum: WGS84; coordinatePrecision: 0.0002; **Identification:** identifiedBy: M. Mei; **Event:** eventDate: 2022-07-12; **Record Level:** collectionID: UR3

#### Lasioglossum glabriusculum

(Morawitz, 1872)

AB7FA0FE-702D-546A-BD9B-061F3F6E4F47

##### Materials

- **Type status:** Other material. **Occurrence:** catalogNumber: A1515; recordedBy: L. Fortini; individualCount: 1; sex: female; lifeStage: adult; occurrenceID: 8DDFAA1B-0E3C-5D19-85AC-0B6A09CA3CBE; **Taxon:** scientificName: Lasioglossum (Pyghalictus) glabrisculum (Morawitz, 1872); order: Hymenoptera; family: Halictidae; genus: Lasioglossum; subgenus: Pyghalictus; specificEpithet: glabrisculum; scientificNameAuthorship: (Morawitz, 1872); **Location:** country: Italy; countryCode: IT; stateProvince: Roma; locality: Riserva Naturale dell'Insugherata 1; decimalLatitude: 41.9555045; decimalLongitude: 12.4292321; geodeticDatum: WGS84; coordinatePrecision: 0.0002; **Identification:** identifiedBy: M. Mei; **Event:** eventDate: 2022-07-30; **Record Level:** collectionID: UR3
- **Type status:** Other material. **Occurrence:** catalogNumber: A2134; recordedBy: L. Fortini; individualCount: 1; sex: female; lifeStage: adult; occurrenceID: 6698B32D-2D99-5524-BAF4-8E012BA5FBF2; **Taxon:** scientificName: Lasioglossum (Pyghalictus) glabrisculum (Morawitz, 1872); order: Hymenoptera; family: Halictidae; genus: Lasioglossum; subgenus: Pyghalictus; specificEpithet: glabrisculum; scientificNameAuthorship: (Morawitz, 1872); **Location:** country: Italy; countryCode: IT; stateProvince: Roma; locality: Riserva Naturale Valle dell'Aniene 2; decimalLatitude: 41.928752; decimalLongitude: 12.5562962; geodeticDatum: WGS84; coordinatePrecision: 0.0002; **Identification:** identifiedBy: M. Mei; **Event:** eventDate: 2022-08-03; **Record Level:** collectionID: UR3

#### Lasioglossum interruptum

(Panzer, 1798)

65FF83B0-C9B4-5997-A2A7-9E65061AC773

##### Materials

- **Type status:** Other material. **Occurrence:** catalogNumber: A1274, A1277; recordedBy: L. Fortini; individualCount: 2; sex: females; lifeStage: adult; occurrenceID: D0C6ABF4-0C71-5C32-B9D7-B5E47684E584; **Taxon:** scientificName: Lasioglossum (Sphecodogastra) interruptum (Panzer, 1798); order: Hymenoptera; family: Halictidae; genus: Lasioglossum; subgenus: Sphecodogastra; specificEpithet: interruptum; scientificNameAuthorship: (Panzer, 1798); **Location:** country: Italy; countryCode: IT; stateProvince: Roma; locality: Riserva Naturale Valle dell'Aniene 2; decimalLatitude: 41.928752; decimalLongitude: 12.5562962; geodeticDatum: WGS84; coordinatePrecision: 0.0002; **Identification:** identifiedBy: M. Mei; **Event:** eventDate: 2022-04-28; **Record Level:** collectionID: UR3
- **Type status:** Other material. **Occurrence:** catalogNumber: A1287; recordedBy: L. Fortini; individualCount: 1; sex: female; lifeStage: adult; occurrenceID: 7322F9A6-3E8A-55D3-83E1-0ABA7CF4C215; **Taxon:** scientificName: Lasioglossum (Sphecodogastra) interruptum (Panzer, 1798); order: Hymenoptera; family: Halictidae; genus: Lasioglossum; subgenus: Sphecodogastra; specificEpithet: interruptum; scientificNameAuthorship: (Panzer, 1798); **Location:** country: Italy; countryCode: IT; stateProvince: Roma; locality: Riserva Naturale Tenuta dei Massimi 1; decimalLatitude: 41.8532859; decimalLongitude: 12.3842322; geodeticDatum: WGS84; coordinatePrecision: 0.0002; **Identification:** identifiedBy: M. Mei; **Event:** eventDate: 2022-04-23; **Record Level:** collectionID: UR3
- **Type status:** Other material. **Occurrence:** catalogNumber: A1358; recordedBy: L. Fortini; individualCount: 1; sex: female; lifeStage: adult; occurrenceID: 9307C1DF-1088-572C-B9A1-6109DAAA90D4; **Taxon:** scientificName: Lasioglossum (Sphecodogastra) interruptum (Panzer, 1798); order: Hymenoptera; family: Halictidae; genus: Lasioglossum; subgenus: Sphecodogastra; specificEpithet: interruptum; scientificNameAuthorship: (Panzer, 1798); **Location:** country: Italy; countryCode: IT; stateProvince: Roma; locality: Riserva Naturale dell'Acquafredda; decimalLatitude: 41.8928408; decimalLongitude: 12.39932; geodeticDatum: WGS84; coordinatePrecision: 0.0002; **Identification:** identifiedBy: M. Mei; **Event:** eventDate: 2022-06-10; **Record Level:** collectionID: UR3
- **Type status:** Other material. **Occurrence:** catalogNumber: A1375; recordedBy: L. Fortini; individualCount: 1; sex: female; lifeStage: adult; occurrenceID: A2E7F6F5-9A5B-5E5B-AA14-992FEA775163; **Taxon:** scientificName: Lasioglossum (Sphecodogastra) interruptum (Panzer, 1798); order: Hymenoptera; family: Halictidae; genus: Lasioglossum; subgenus: Sphecodogastra; specificEpithet: interruptum; scientificNameAuthorship: (Panzer, 1798); **Location:** country: Italy; countryCode: IT; stateProvince: Roma; locality: Riserva Naturale Valle dei Casali 2; decimalLatitude: 41.8596887; decimalLongitude: 12.4355075; geodeticDatum: WGS84; coordinatePrecision: 0.0002; **Identification:** identifiedBy: M. Mei; **Event:** eventDate: 2022-07-18; **Record Level:** collectionID: UR3
- **Type status:** Other material. **Occurrence:** catalogNumber: A1385; recordedBy: L. Fortini; individualCount: 1; sex: male; lifeStage: adult; occurrenceID: B26A3A3F-DBB6-5E36-B922-164D80846E60; **Taxon:** scientificName: Lasioglossum (Sphecodogastra) interruptum (Panzer, 1798); order: Hymenoptera; family: Halictidae; genus: Lasioglossum; subgenus: Sphecodogastra; specificEpithet: interruptum; scientificNameAuthorship: (Panzer, 1798); **Location:** country: Italy; countryCode: IT; stateProvince: Roma; locality: Riserva Naturale Tenuta dei Massimi 2; decimalLatitude: 41.8316516; decimalLongitude: 12.3999927; geodeticDatum: WGS84; coordinatePrecision: 0.0002; **Identification:** identifiedBy: M. Mei; **Event:** eventDate: 2022-06-01; **Record Level:** collectionID: UR3

#### Lasioglossum lativentre

(Schenck, 1853)

024CFE9D-BD06-5077-B0A8-E425CA30AED7

##### Materials

- **Type status:** Other material. **Occurrence:** catalogNumber: A1576, A1578; recordedBy: L. Fortini; individualCount: 2; sex: females; lifeStage: adult; occurrenceID: E3141B4C-8C0F-5129-81C4-300B0BD1CAB9; **Taxon:** scientificName: Lasioglossum (Lasioglossum) lativentre (Schenck, 1853); order: Hymenoptera; family: Halictidae; genus: Lasioglossum; subgenus: Lasioglossum; specificEpithet: lativentre; scientificNameAuthorship: (Schenck, 1853); **Location:** country: Italy; countryCode: IT; stateProvince: Roma; locality: Riserva Naturale Valle dell'Aniene 1; decimalLatitude: 41.9345179; decimalLongitude: 12.5453096; geodeticDatum: WGS84; coordinatePrecision: 0.0002; **Identification:** identifiedBy: M. Mei; **Event:** eventDate: 2022-04-28; **Record Level:** collectionID: UR3

#### Lasioglossum leucozonium

(Schrank, 1781)

E707E4C4-9736-5477-BD13-9507657B176F

##### Materials

- **Type status:** Other material. **Occurrence:** catalogNumber: A1588; recordedBy: L. Fortini; individualCount: 1; sex: female; lifeStage: adult; occurrenceID: E740C137-52BA-585C-B5CC-284F355690DB; **Taxon:** scientificName: Lasioglossum (Leuchalictus) leucozonium (Schrank, 1781); order: Hymenoptera; family: Halictidae; genus: Lasioglossum; subgenus: Leuchalictus; specificEpithet: leucozonium; scientificNameAuthorship: (Schrank, 1781); **Location:** country: Italy; countryCode: IT; stateProvince: Roma; locality: Riserva Naturale dell'Insugherata 1; decimalLatitude: 41.9555045; decimalLongitude: 12.4292321; geodeticDatum: WGS84; coordinatePrecision: 0.0002; **Identification:** identifiedBy: M. Mei; **Event:** eventDate: 2022-04-15; **Record Level:** collectionID: UR3
- **Type status:** Other material. **Occurrence:** catalogNumber: A1336, A1338, A1524, A1711; recordedBy: L. Fortini; individualCount: 4; sex: females; lifeStage: adult; occurrenceID: F94B9D0A-7966-5693-8508-28BF9A2D56ED; **Taxon:** scientificName: Lasioglossum (Leuchalictus) leucozonium (Schrank, 1781); order: Hymenoptera; family: Halictidae; genus: Lasioglossum; subgenus: Leuchalictus; specificEpithet: leucozonium; scientificNameAuthorship: (Schrank, 1781); **Location:** country: Italy; countryCode: IT; stateProvince: Roma; locality: Riserva Naturale di Monte Mario; decimalLatitude: 41.9386215; decimalLongitude: 12.4546223; geodeticDatum: WGS84; coordinatePrecision: 0.0002; **Identification:** identifiedBy: M. Mei; **Event:** eventDate: 2022-05-20; **Record Level:** collectionID: UR3
- **Type status:** Other material. **Occurrence:** catalogNumber: A1386; recordedBy: L. Fortini; individualCount: 1; sex: male; lifeStage: adult; occurrenceID: 17045F4C-7607-5C7B-86DA-3C04DB99C629; **Taxon:** scientificName: Lasioglossum (Leuchalictus) leucozonium (Schrank, 1781); order: Hymenoptera; family: Halictidae; genus: Lasioglossum; subgenus: Leuchalictus; specificEpithet: leucozonium; scientificNameAuthorship: (Schrank, 1781); **Location:** country: Italy; countryCode: IT; stateProvince: Roma; locality: Riserva Naturale dell'Insugherata 3; decimalLatitude: 41.9644829; decimalLongitude: 12.436101; geodeticDatum: WGS84; coordinatePrecision: 0.0002; **Identification:** identifiedBy: M. Mei; **Event:** eventDate: 2022-05-27; **Record Level:** collectionID: UR3
- **Type status:** Other material. **Occurrence:** catalogNumber: A1745; recordedBy: L. Fortini; individualCount: 1; sex: female; lifeStage: adult; occurrenceID: 18CECC98-891B-5D35-B3D2-C55769B68DDB; **Taxon:** scientificName: Lasioglossum (Leuchalictus) leucozonium (Schrank, 1781); order: Hymenoptera; family: Halictidae; genus: Lasioglossum; subgenus: Leuchalictus; specificEpithet: leucozonium; scientificNameAuthorship: (Schrank, 1781); **Location:** country: Italy; countryCode: IT; stateProvince: Roma; locality: Riserva Naturale dell'Insugherata 1; decimalLatitude: 41.9555045; decimalLongitude: 12.4292321; geodeticDatum: WGS84; coordinatePrecision: 0.0002; **Identification:** identifiedBy: M. Mei; **Event:** eventDate: 2022-06-24; **Record Level:** collectionID: UR3
- **Type status:** Other material. **Occurrence:** catalogNumber: A1629; recordedBy: L. Fortini; individualCount: 1; sex: male; lifeStage: adult; occurrenceID: E66E6CF3-E0BF-54E8-BA7E-4054D73EE1B4; **Taxon:** scientificName: Lasioglossum (Leuchalictus) leucozonium (Schrank, 1781); order: Hymenoptera; family: Halictidae; genus: Lasioglossum; subgenus: Leuchalictus; specificEpithet: leucozonium; scientificNameAuthorship: (Schrank, 1781); **Location:** country: Italy; countryCode: IT; stateProvince: Roma; locality: Riserva Regionale dell'Appia Antica 2; decimalLatitude: 41.8402564; decimalLongitude: 12.532773; geodeticDatum: WGS84; coordinatePrecision: 0.0002; **Identification:** identifiedBy: M. Mei; **Event:** eventDate: 2022-07-07; **Record Level:** collectionID: UR3
- **Type status:** Other material. **Occurrence:** catalogNumber: A1562; recordedBy: L. Fortini; individualCount: 1; sex: female; lifeStage: adult; occurrenceID: F10FDF7A-1AF5-5959-A217-550608A0AC23; **Taxon:** scientificName: Lasioglossum (Leuchalictus) leucozonium (Schrank, 1781); order: Hymenoptera; family: Halictidae; genus: Lasioglossum; subgenus: Leuchalictus; specificEpithet: leucozonium; scientificNameAuthorship: (Schrank, 1781); **Location:** country: Italy; countryCode: IT; stateProvince: Roma; locality: Riserva Naturale dell'Acquafredda; decimalLatitude: 41.8928408; decimalLongitude: 12.39932; geodeticDatum: WGS84; coordinatePrecision: 0.0002; **Identification:** identifiedBy: M. Mei; **Event:** eventDate: 2022-07-12; **Record Level:** collectionID: UR3
- **Type status:** Other material. **Occurrence:** catalogNumber: A1526; recordedBy: L. Fortini; individualCount: 1; sex: female; lifeStage: adult; occurrenceID: EBD3049A-034B-54F2-9C5A-D4A745B40CA4; **Taxon:** scientificName: Lasioglossum (Leuchalictus) leucozonium (Schrank, 1781); order: Hymenoptera; family: Halictidae; genus: Lasioglossum; subgenus: Leuchalictus; specificEpithet: leucozonium; scientificNameAuthorship: (Schrank, 1781); **Location:** country: Italy; countryCode: IT; stateProvince: Roma; locality: Riserva Naturale Valle dei Casali 2; decimalLatitude: 41.8596887; decimalLongitude: 12.4355075; geodeticDatum: WGS84; coordinatePrecision: 0.0002; **Identification:** identifiedBy: M. Mei; **Event:** eventDate: 2022-07-13; **Record Level:** collectionID: UR3
- **Type status:** Other material. **Occurrence:** catalogNumber: A1733; recordedBy: L. Fortini; individualCount: 1; sex: female; lifeStage: adult; occurrenceID: F2372C1B-F7F5-56C7-8BA2-7EBE7599FE41; **Taxon:** scientificName: Lasioglossum (Leuchalictus) leucozonium (Schrank, 1781); order: Hymenoptera; family: Halictidae; genus: Lasioglossum; subgenus: Leuchalictus; specificEpithet: leucozonium; scientificNameAuthorship: (Schrank, 1781); **Location:** country: Italy; countryCode: IT; stateProvince: Roma; locality: Riserva Naturale Laurentino-Acqua Acetosa; decimalLatitude: 41.8079275; decimalLongitude: 12.4685548; geodeticDatum: WGS84; coordinatePrecision: 0.0002; **Identification:** identifiedBy: M. Mei; **Event:** eventDate: 2022-07-17; **Record Level:** collectionID: UR3
- **Type status:** Other material. **Occurrence:** catalogNumber: A1628, A1675; recordedBy: L. Fortini; individualCount: 2; sex: males; lifeStage: adult; occurrenceID: 444F0426-8B8A-57D8-B218-7EE3660F5D09; **Taxon:** scientificName: Lasioglossum (Leuchalictus) leucozonium (Schrank, 1781); order: Hymenoptera; family: Halictidae; genus: Lasioglossum; subgenus: Leuchalictus; specificEpithet: leucozonium; scientificNameAuthorship: (Schrank, 1781); **Location:** country: Italy; countryCode: IT; stateProvince: Roma; locality: Riserva Regionale dell'Appia Antica 2; decimalLatitude: 41.8402564; decimalLongitude: 12.532773; geodeticDatum: WGS84; coordinatePrecision: 0.0002; **Identification:** identifiedBy: M. Mei; **Event:** eventDate: 2022-08-06; **Record Level:** collectionID: UR3
- **Type status:** Other material. **Occurrence:** catalogNumber: A1612; recordedBy: L. Fortini; individualCount: 1; sex: female; lifeStage: adult; occurrenceID: 296D4F52-6A3F-5EF3-9F3D-1EAD433D3D09; **Taxon:** scientificName: Lasioglossum (Leuchalictus) leucozonium (Schrank, 1781); order: Hymenoptera; family: Halictidae; genus: Lasioglossum; subgenus: Leuchalictus; specificEpithet: leucozonium; scientificNameAuthorship: (Schrank, 1781); **Location:** country: Italy; countryCode: IT; stateProvince: Roma; locality: Riserva Naturale di Monte Mario; decimalLatitude: 41.9386215; decimalLongitude: 12.4546223; geodeticDatum: WGS84; coordinatePrecision: 0.0002; **Identification:** identifiedBy: M. Mei; **Event:** eventDate: 2022-08-23; **Record Level:** collectionID: UR3
- **Type status:** Other material. **Occurrence:** catalogNumber: A1579; recordedBy: L. Fortini; individualCount: 1; sex: female; lifeStage: adult; occurrenceID: 97AD7EC4-E936-5430-AAA9-AC53F9AEB108; **Taxon:** scientificName: Lasioglossum (Leuchalictus) leucozonium (Schrank, 1781); order: Hymenoptera; family: Halictidae; genus: Lasioglossum; subgenus: Leuchalictus; specificEpithet: leucozonium; scientificNameAuthorship: (Schrank, 1781); **Location:** country: Italy; countryCode: IT; stateProvince: Roma; locality: Riserva Naturale Laurentino-Acqua Acetosa; decimalLatitude: 41.8079275; decimalLongitude: 12.4685548; geodeticDatum: WGS84; coordinatePrecision: 0.0002; **Identification:** identifiedBy: M. Mei; **Event:** eventDate: 2022-09-14; **Record Level:** collectionID: UR3
- **Type status:** Other material. **Occurrence:** catalogNumber: A1630; recordedBy: L. Fortini; individualCount: 1; sex: male; lifeStage: adult; occurrenceID: D44E9049-13E8-5F94-AAE9-1783C2756684; **Taxon:** scientificName: Lasioglossum (Leuchalictus) leucozonium (Schrank, 1781); order: Hymenoptera; family: Halictidae; genus: Lasioglossum; subgenus: Leuchalictus; specificEpithet: leucozonium; scientificNameAuthorship: (Schrank, 1781); **Location:** country: Italy; countryCode: IT; stateProvince: Roma; locality: Riserva Naturale Valle dei Casali 1; decimalLatitude: 41.8710627; decimalLongitude: 12.4336809; geodeticDatum: WGS84; coordinatePrecision: 0.0002; **Identification:** identifiedBy: M. Mei; **Event:** eventDate: 2022-09-18; **Record Level:** collectionID: UR3

#### Lasioglossum malachurum

(Kirby, 1802)

3040A565-FD2B-581A-A50A-6D110F645E96

##### Materials

- **Type status:** Other material. **Occurrence:** catalogNumber: A1353, A1360; recordedBy: L. Fortini; individualCount: 2; sex: females; lifeStage: adult; occurrenceID: 02085284-D366-5FD7-A626-808C9BBA8DA2; **Taxon:** scientificName: Lasioglossum (Sphecodogastra) malachurum (Kirby, 1802); order: Hymenoptera; family: Halictidae; genus: Lasioglossum; subgenus: Sphecodogastra; specificEpithet: malachurum; scientificNameAuthorship: (Kirby, 1802); **Location:** country: Italy; countryCode: IT; stateProvince: Roma; locality: Riserva Naturale dell'Acquafredda; decimalLatitude: 41.8928408; decimalLongitude: 12.39932; geodeticDatum: WGS84; coordinatePrecision: 0.0002; **Identification:** identifiedBy: M. Mei; **Event:** eventDate: 2022-06-10; **Record Level:** collectionID: UR3
- **Type status:** Other material. **Occurrence:** catalogNumber: A1735; recordedBy: L. Fortini; individualCount: 1; sex: female; lifeStage: adult; occurrenceID: 43C212B5-0E47-51C7-91ED-F534E3B5D94F; **Taxon:** scientificName: Lasioglossum (Sphecodogastra) malachurum (Kirby, 1802); order: Hymenoptera; family: Halictidae; genus: Lasioglossum; subgenus: Sphecodogastra; specificEpithet: malachurum; scientificNameAuthorship: (Kirby, 1802); **Location:** country: Italy; countryCode: IT; stateProvince: Roma; locality: Riserva Naturale dell'Acquafredda; decimalLatitude: 41.8928408; decimalLongitude: 12.39932; geodeticDatum: WGS84; coordinatePrecision: 0.0002; **Identification:** identifiedBy: M. Mei; **Event:** eventDate: 2022-04-13; **Record Level:** collectionID: UR3
- **Type status:** Other material. **Occurrence:** catalogNumber: A1673; recordedBy: L. Fortini; individualCount: 1; sex: male; lifeStage: adult; occurrenceID: 216D86BB-5935-585A-9EDC-115A0F332F79; **Taxon:** scientificName: Lasioglossum (Sphecodogastra) malachurum (Kirby, 1802); order: Hymenoptera; family: Halictidae; genus: Lasioglossum; subgenus: Sphecodogastra; specificEpithet: malachurum; scientificNameAuthorship: (Kirby, 1802); **Location:** country: Italy; countryCode: IT; stateProvince: Roma; locality: Riserva Naturale dell'Acquafredda; decimalLatitude: 41.8928408; decimalLongitude: 12.39932; geodeticDatum: WGS84; coordinatePrecision: 0.0002; **Identification:** identifiedBy: M. Mei; **Event:** eventDate: 2022-07-12; **Record Level:** collectionID: UR3
- **Type status:** Other material. **Occurrence:** catalogNumber: A1346, A1347, A1348, A1350, A1362; recordedBy: L. Fortini; individualCount: 5; sex: females; lifeStage: adult; occurrenceID: CA8B371D-304F-566D-A249-9DB1C75F31A9; **Taxon:** scientificName: Lasioglossum (Sphecodogastra) malachurum (Kirby, 1802); order: Hymenoptera; family: Halictidae; genus: Lasioglossum; subgenus: Sphecodogastra; specificEpithet: malachurum; scientificNameAuthorship: (Kirby, 1802); **Location:** country: Italy; countryCode: IT; stateProvince: Roma; locality: Riserva Regionale dell'Appia Antica 1; decimalLatitude: 41.8623941; decimalLongitude: 12.524863; geodeticDatum: WGS84; coordinatePrecision: 0.0002; **Identification:** identifiedBy: M. Mei; **Event:** eventDate: 2022-06-12; **Record Level:** collectionID: UR3
- **Type status:** Other material. **Occurrence:** catalogNumber: A1491; recordedBy: L. Fortini; individualCount: 1; sex: female; lifeStage: adult; occurrenceID: 59D64A4F-3279-50A0-B87C-0B9C7A5ABE13; **Taxon:** scientificName: Lasioglossum (Sphecodogastra) malachurum (Kirby, 1802); order: Hymenoptera; family: Halictidae; genus: Lasioglossum; subgenus: Sphecodogastra; specificEpithet: malachurum; scientificNameAuthorship: (Kirby, 1802); **Location:** country: Italy; countryCode: IT; stateProvince: Roma; locality: Riserva Regionale dell'Appia Antica 1; decimalLatitude: 41.8623941; decimalLongitude: 12.524863; geodeticDatum: WGS84; coordinatePrecision: 0.0002; **Identification:** identifiedBy: M. Mei; **Event:** eventDate: 2022-07-22; **Record Level:** collectionID: UR3
- **Type status:** Other material. **Occurrence:** catalogNumber: A1264, A1410; recordedBy: L. Fortini; individualCount: 2; sex: females; lifeStage: adult; occurrenceID: C039FF85-1EDD-595F-8D1C-77464D0B01FA; **Taxon:** scientificName: Lasioglossum (Sphecodogastra) malachurum (Kirby, 1802); order: Hymenoptera; family: Halictidae; genus: Lasioglossum; subgenus: Sphecodogastra; specificEpithet: malachurum; scientificNameAuthorship: (Kirby, 1802); **Location:** country: Italy; countryCode: IT; stateProvince: Roma; locality: Riserva Regionale dell'Appia Antica 2; decimalLatitude: 41.8402564; decimalLongitude: 12.532773; geodeticDatum: WGS84; coordinatePrecision: 0.0002; **Identification:** identifiedBy: M. Mei; **Event:** eventDate: 2022-05-24; **Record Level:** collectionID: UR3
- **Type status:** Other material. **Occurrence:** catalogNumber: A1414, A1418, A1421, A1423, A1449, A1450, A1457, A1458, A1460, A1461, A1466; recordedBy: L. Fortini; individualCount: 11; sex: females; lifeStage: adult; occurrenceID: BF9BD264-9178-515E-962C-27324BCAC97C; **Taxon:** scientificName: Lasioglossum (Sphecodogastra) malachurum (Kirby, 1802); order: Hymenoptera; family: Halictidae; genus: Lasioglossum; subgenus: Sphecodogastra; specificEpithet: malachurum; scientificNameAuthorship: (Kirby, 1802); **Location:** country: Italy; countryCode: IT; stateProvince: Roma; locality: Riserva Regionale dell'Appia Antica 2; decimalLatitude: 41.8402564; decimalLongitude: 12.532773; geodeticDatum: WGS84; coordinatePrecision: 0.0002; **Identification:** identifiedBy: M. Mei; **Event:** eventDate: 2022-07-07; **Record Level:** collectionID: UR3
- **Type status:** Other material. **Occurrence:** catalogNumber: A1622, A1623, A1624; recordedBy: L. Fortini; individualCount: 3; sex: males; lifeStage: adult; occurrenceID: 72F2BFFF-1993-5945-A3F7-CE4A4745D73A; **Taxon:** scientificName: Lasioglossum (Sphecodogastra) malachurum (Kirby, 1802); order: Hymenoptera; family: Halictidae; genus: Lasioglossum; subgenus: Sphecodogastra; specificEpithet: malachurum; scientificNameAuthorship: (Kirby, 1802); **Location:** country: Italy; countryCode: IT; stateProvince: Roma; locality: Riserva Regionale dell'Appia Antica 2; decimalLatitude: 41.8402564; decimalLongitude: 12.532773; geodeticDatum: WGS84; coordinatePrecision: 0.0002; **Identification:** identifiedBy: M. Mei; **Event:** eventDate: 2022-08-06; **Record Level:** collectionID: UR3
- **Type status:** Other material. **Occurrence:** catalogNumber: A1265, A1266, A1267, A1269, A1270; recordedBy: L. Fortini; individualCount: 5; sex: females; lifeStage: adult; occurrenceID: CB941981-3BC3-557E-93C1-3D6C531F1719; **Taxon:** scientificName: Lasioglossum (Sphecodogastra) malachurum (Kirby, 1802); order: Hymenoptera; family: Halictidae; genus: Lasioglossum; subgenus: Sphecodogastra; specificEpithet: malachurum; scientificNameAuthorship: (Kirby, 1802); **Location:** country: Italy; countryCode: IT; stateProvince: Roma; locality: Riserva Regionale dell'Appia Antica 3; decimalLatitude: 41.8298456; decimalLongitude: 12.5432538; geodeticDatum: WGS84; coordinatePrecision: 0.0002; **Identification:** identifiedBy: M. Mei; **Event:** eventDate: 2022-05-24; **Record Level:** collectionID: UR3
- **Type status:** Other material. **Occurrence:** catalogNumber: A1370; recordedBy: L. Fortini; individualCount: 1; sex: female; lifeStage: adult; occurrenceID: F12E608A-B2DC-5F3E-A93B-FA2E8A8E590E; **Taxon:** scientificName: Lasioglossum (Sphecodogastra) malachurum (Kirby, 1802); order: Hymenoptera; family: Halictidae; genus: Lasioglossum; subgenus: Sphecodogastra; specificEpithet: malachurum; scientificNameAuthorship: (Kirby, 1802); **Location:** country: Italy; countryCode: IT; stateProvince: Roma; locality: Riserva Naturale dell'Insugherata 1; decimalLatitude: 41.9555045; decimalLongitude: 12.4292321; geodeticDatum: WGS84; coordinatePrecision: 0.0002; **Identification:** identifiedBy: M. Mei; **Event:** eventDate: 2022-06-24; **Record Level:** collectionID: UR3
- **Type status:** Other material. **Occurrence:** catalogNumber: A1752; recordedBy: L. Fortini; individualCount: 1; sex: female; lifeStage: adult; occurrenceID: 1ED58D4B-CDFD-5EED-9198-7DBECD6D7423; **Taxon:** scientificName: Lasioglossum (Sphecodogastra) malachurum (Kirby, 1802); order: Hymenoptera; family: Halictidae; genus: Lasioglossum; subgenus: Sphecodogastra; specificEpithet: malachurum; scientificNameAuthorship: (Kirby, 1802); **Location:** country: Italy; countryCode: IT; stateProvince: Roma; locality: Riserva Naturale dell'Insugherata 1; decimalLatitude: 41.9555045; decimalLongitude: 12.4292321; geodeticDatum: WGS84; coordinatePrecision: 0.0002; **Identification:** identifiedBy: M. Mei; **Event:** eventDate: 2022-04-15; **Record Level:** collectionID: UR3
- **Type status:** Other material. **Occurrence:** catalogNumber: A1367, A1417, A1428, A1429; recordedBy: L. Fortini; individualCount: 4; sex: females; lifeStage: adult; occurrenceID: CC174DC2-04AA-52A7-B3AC-3B6EB5162422; **Taxon:** scientificName: Lasioglossum (Sphecodogastra) malachurum (Kirby, 1802); order: Hymenoptera; family: Halictidae; genus: Lasioglossum; subgenus: Sphecodogastra; specificEpithet: malachurum; scientificNameAuthorship: (Kirby, 1802); **Location:** country: Italy; countryCode: IT; stateProvince: Roma; locality: Riserva Naturale dell'Insugherata 3; decimalLatitude: 41.9644829; decimalLongitude: 12.436101; geodeticDatum: WGS84; coordinatePrecision: 0.0002; **Identification:** identifiedBy: M. Mei; **Event:** eventDate: 2022-06-24; **Record Level:** collectionID: UR3
- **Type status:** Other material. **Occurrence:** catalogNumber: A1392; recordedBy: L. Fortini; individualCount: 1; sex: female; lifeStage: adult; occurrenceID: 2E70FF16-0858-5CAF-A713-2B5E8AF0948C; **Taxon:** scientificName: Lasioglossum (Sphecodogastra) malachurum (Kirby, 1802); order: Hymenoptera; family: Halictidae; genus: Lasioglossum; subgenus: Sphecodogastra; specificEpithet: malachurum; scientificNameAuthorship: (Kirby, 1802); **Location:** country: Italy; countryCode: IT; stateProvince: Roma; locality: Riserva Naturale dell'Insugherata 3; decimalLatitude: 41.9644829; decimalLongitude: 12.436101; geodeticDatum: WGS84; coordinatePrecision: 0.0002; **Identification:** identifiedBy: M. Mei; **Event:** eventDate: 2022-05-27; **Record Level:** collectionID: UR3
- **Type status:** Other material. **Occurrence:** catalogNumber: A1245; recordedBy: L. Fortini; individualCount: 1; sex: female; lifeStage: adult; occurrenceID: 71D4F48D-C958-5877-A9B3-E7D1BED6E94F; **Taxon:** scientificName: Lasioglossum (Sphecodogastra) malachurum (Kirby, 1802); order: Hymenoptera; family: Halictidae; genus: Lasioglossum; subgenus: Sphecodogastra; specificEpithet: malachurum; scientificNameAuthorship: (Kirby, 1802); **Location:** country: Italy; countryCode: IT; stateProvince: Roma; locality: Riserva Naturale Laurentino-Acqua Acetosa; decimalLatitude: 41.8079275; decimalLongitude: 12.4685548; geodeticDatum: WGS84; coordinatePrecision: 0.0002; **Identification:** identifiedBy: M. Mei; **Event:** eventDate: 2022-04-12; **Record Level:** collectionID: UR3
- **Type status:** Other material. **Occurrence:** catalogNumber: A1268, A1271, A1323, A1329, A1334, A1335, A1339; recordedBy: L. Fortini; individualCount: 7; sex: females; lifeStage: adult; occurrenceID: A9F0D8E6-BAC4-5441-BE20-426A8BCFA230; **Taxon:** scientificName: Lasioglossum (Sphecodogastra) malachurum (Kirby, 1802); order: Hymenoptera; family: Halictidae; genus: Lasioglossum; subgenus: Sphecodogastra; specificEpithet: malachurum; scientificNameAuthorship: (Kirby, 1802); **Location:** country: Italy; countryCode: IT; stateProvince: Roma; locality: Riserva Naturale di Monte Mario; decimalLatitude: 41.9386215; decimalLongitude: 12.4546223; geodeticDatum: WGS84; coordinatePrecision: 0.0002; **Identification:** identifiedBy: M. Mei; **Event:** eventDate: 2022-05-20; **Record Level:** collectionID: UR3
- **Type status:** Other material. **Occurrence:** catalogNumber: A1416, A1419, A1420, A1422, A1424, A1426; recordedBy: L. Fortini; individualCount: 6; sex: females; lifeStage: adult; occurrenceID: B380AA6F-0811-5BC5-8226-4C275304D7DB; **Taxon:** scientificName: Lasioglossum (Sphecodogastra) malachurum (Kirby, 1802); order: Hymenoptera; family: Halictidae; genus: Lasioglossum; subgenus: Sphecodogastra; specificEpithet: malachurum; scientificNameAuthorship: (Kirby, 1802); **Location:** country: Italy; countryCode: IT; stateProvince: Roma; locality: Riserva Naturale di Monte Mario; decimalLatitude: 41.9386215; decimalLongitude: 12.4546223; geodeticDatum: WGS84; coordinatePrecision: 0.0002; **Identification:** identifiedBy: M. Mei; **Event:** eventDate: 2022-06-19; **Record Level:** collectionID: UR3
- **Type status:** Other material. **Occurrence:** catalogNumber: A1292, A1295, A1296, A1298, A1299, A1303, A1311, A1313, A1314; recordedBy: L. Fortini; individualCount: 9; sex: females; lifeStage: adult; occurrenceID: 91965303-B073-57C9-A8E0-46B7837AC9DD; **Taxon:** scientificName: Lasioglossum (Sphecodogastra) malachurum (Kirby, 1802); order: Hymenoptera; family: Halictidae; genus: Lasioglossum; subgenus: Sphecodogastra; specificEpithet: malachurum; scientificNameAuthorship: (Kirby, 1802); **Location:** country: Italy; countryCode: IT; stateProvince: Roma; locality: Riserva Naturale Tenuta dei Massimi 1; decimalLatitude: 41.8532859; decimalLongitude: 12.3842322; geodeticDatum: WGS84; coordinatePrecision: 0.0002; **Identification:** identifiedBy: M. Mei; **Event:** eventDate: 2022-04-23; **Record Level:** collectionID: UR3
- **Type status:** Other material. **Occurrence:** catalogNumber: A1415, A1427; recordedBy: L. Fortini; individualCount: 2; sex: females; lifeStage: adult; occurrenceID: 1004B092-99F6-532E-9B30-BAD78FF93620; **Taxon:** scientificName: Lasioglossum (Sphecodogastra) malachurum (Kirby, 1802); order: Hymenoptera; family: Halictidae; genus: Lasioglossum; subgenus: Sphecodogastra; specificEpithet: malachurum; scientificNameAuthorship: (Kirby, 1802); **Location:** country: Italy; countryCode: IT; stateProvince: Roma; locality: Riserva Naturale Tenuta dei Massimi 1; decimalLatitude: 41.8532859; decimalLongitude: 12.3842322; geodeticDatum: WGS84; coordinatePrecision: 0.0002; **Identification:** identifiedBy: M. Mei; **Event:** eventDate: 2022-06-27; **Record Level:** collectionID: UR3
- **Type status:** Other material. **Occurrence:** catalogNumber: A1471, A1472, A1473, A1548, A1620; recordedBy: L. Fortini; individualCount: 5; sex: 1 male, 4 females; lifeStage: adult; occurrenceID: E37697B2-BC2E-5BA9-9C3C-315C9B0D4725; **Taxon:** scientificName: Lasioglossum (Sphecodogastra) malachurum (Kirby, 1802); order: Hymenoptera; family: Halictidae; genus: Lasioglossum; subgenus: Sphecodogastra; specificEpithet: malachurum; scientificNameAuthorship: (Kirby, 1802); **Location:** country: Italy; countryCode: IT; stateProvince: Roma; locality: Riserva Naturale Tenuta dei Massimi 1; decimalLatitude: 41.8532859; decimalLongitude: 12.3842322; geodeticDatum: WGS84; coordinatePrecision: 0.0002; **Identification:** identifiedBy: M. Mei; **Event:** eventDate: 2022-07-28; **Record Level:** collectionID: UR3
- **Type status:** Other material. **Occurrence:** catalogNumber: A1351; recordedBy: L. Fortini; individualCount: 1; sex: female; lifeStage: adult; occurrenceID: 519F8B5A-7538-5AB0-973B-BDAD362FEE2C; **Taxon:** scientificName: Lasioglossum (Sphecodogastra) malachurum (Kirby, 1802); order: Hymenoptera; family: Halictidae; genus: Lasioglossum; subgenus: Sphecodogastra; specificEpithet: malachurum; scientificNameAuthorship: (Kirby, 1802); **Location:** country: Italy; countryCode: IT; stateProvince: Roma; locality: Riserva Naturale Valle dell'Aniene 2; decimalLatitude: 41.928752; decimalLongitude: 12.5562962; geodeticDatum: WGS84; coordinatePrecision: 0.0002; **Identification:** identifiedBy: M. Mei; **Event:** eventDate: 2022-06-05; **Record Level:** collectionID: UR3
- **Type status:** Other material. **Occurrence:** catalogNumber: A1413, A1425, A1435; recordedBy: L. Fortini; individualCount: 3; sex: females; lifeStage: adult; occurrenceID: 1AD0F3B3-5D2E-584A-A221-9FC9BC0A8358; **Taxon:** scientificName: Lasioglossum (Sphecodogastra) malachurum (Kirby, 1802); order: Hymenoptera; family: Halictidae; genus: Lasioglossum; subgenus: Sphecodogastra; specificEpithet: malachurum; scientificNameAuthorship: (Kirby, 1802); **Location:** country: Italy; countryCode: IT; stateProvince: Roma; locality: Riserva Naturale Valle dell'Aniene 2; decimalLatitude: 41.928752; decimalLongitude: 12.5562962; geodeticDatum: WGS84; coordinatePrecision: 0.0002; **Identification:** identifiedBy: M. Mei; **Event:** eventDate: 2022-07-01; **Record Level:** collectionID: UR3
- **Type status:** Other material. **Occurrence:** catalogNumber: A1248, A1288, A1525; recordedBy: L. Fortini; individualCount: 3; sex: females; lifeStage: adult; occurrenceID: 52D77D85-B8B3-5044-8750-7448254DCC9D; **Taxon:** scientificName: Lasioglossum (Sphecodogastra) malachurum (Kirby, 1802); order: Hymenoptera; family: Halictidae; genus: Lasioglossum; subgenus: Sphecodogastra; specificEpithet: malachurum; scientificNameAuthorship: (Kirby, 1802); **Location:** country: Italy; countryCode: IT; stateProvince: Roma; locality: Riserva Naturale Valle dei Casali 1; decimalLatitude: 41.8710627; decimalLongitude: 12.4336809; geodeticDatum: WGS84; coordinatePrecision: 0.0002; **Identification:** identifiedBy: M. Mei; **Event:** eventDate: 2022-04-07; **Record Level:** collectionID: UR3
- **Type status:** Other material. **Occurrence:** catalogNumber: A1246, A1291, A1300, A1302, A1306, A1308, A1310, A1750; recordedBy: L. Fortini; individualCount: 8; sex: females; lifeStage: adult; occurrenceID: A80A855A-4B98-5CDB-AA72-513444FB777D; **Taxon:** scientificName: Lasioglossum (Sphecodogastra) malachurum (Kirby, 1802); order: Hymenoptera; family: Halictidae; genus: Lasioglossum; subgenus: Sphecodogastra; specificEpithet: malachurum; scientificNameAuthorship: (Kirby, 1802); **Location:** country: Italy; countryCode: IT; stateProvince: Roma; locality: Riserva Naturale Valle dei Casali 2; decimalLatitude: 41.8596887; decimalLongitude: 12.4355075; geodeticDatum: WGS84; coordinatePrecision: 0.0002; **Identification:** identifiedBy: M. Mei; **Event:** eventDate: 2022-04-10; **Record Level:** collectionID: UR3
- **Type status:** Other material. **Occurrence:** catalogNumber: A1364, A1366, A1371, A1373, A1374, A1377, A1378; recordedBy: L. Fortini; individualCount: 7; sex: females; lifeStage: adult; occurrenceID: 8B590BBB-D8B6-5B38-9E80-4ED59F872F3A; **Taxon:** scientificName: Lasioglossum (Sphecodogastra) malachurum (Kirby, 1802); order: Hymenoptera; family: Halictidae; genus: Lasioglossum; subgenus: Sphecodogastra; specificEpithet: malachurum; scientificNameAuthorship: (Kirby, 1802); **Location:** country: Italy; countryCode: IT; stateProvince: Roma; locality: Riserva Naturale Valle dei Casali 2; decimalLatitude: 41.8596887; decimalLongitude: 12.4355075; geodeticDatum: WGS84; coordinatePrecision: 0.0002; **Identification:** identifiedBy: M. Mei; **Event:** eventDate: 2022-06-18; **Record Level:** collectionID: UR3
- **Type status:** Other material. **Occurrence:** catalogNumber: A1476, A1477, A1478, A1480, A1486, A1487, A1489, A1490; recordedBy: L. Fortini; individualCount: 8; sex: females; lifeStage: adult; occurrenceID: DE52F378-67DE-553D-8239-E72E8ED874E9; **Taxon:** scientificName: Lasioglossum (Sphecodogastra) malachurum (Kirby, 1802); order: Hymenoptera; family: Halictidae; genus: Lasioglossum; subgenus: Sphecodogastra; specificEpithet: malachurum; scientificNameAuthorship: (Kirby, 1802); **Location:** country: Italy; countryCode: IT; stateProvince: Roma; locality: Riserva Naturale Valle dei Casali 2; decimalLatitude: 41.8596887; decimalLongitude: 12.4355075; geodeticDatum: WGS84; coordinatePrecision: 0.0002; **Identification:** identifiedBy: M. Mei; **Event:** eventDate: 2022-07-13; **Record Level:** collectionID: UR3
- **Type status:** Other material. **Occurrence:** catalogNumber: A2069, A2070; recordedBy: L. Fortini; individualCount: 2; sex: females; lifeStage: adult; occurrenceID: 42690B56-EA31-5610-8E6A-43E279C40C02; **Taxon:** scientificName: Lasioglossum (Sphecodogastra) malachurum (Kirby, 1802); order: Hymenoptera; family: Halictidae; genus: Lasioglossum; subgenus: Sphecodogastra; specificEpithet: malachurum; scientificNameAuthorship: (Kirby, 1802); **Location:** country: Italy; countryCode: IT; stateProvince: Roma; locality: Riserva Naturale di Monte Mario; decimalLatitude: 41.9386215; decimalLongitude: 12.4546223; geodeticDatum: WGS84; coordinatePrecision: 0.0002; **Identification:** identifiedBy: M. Mei; **Event:** eventDate: 2022-07-24; **Record Level:** collectionID: UR3
- **Type status:** Other material. **Occurrence:** catalogNumber: A2082, A2084; recordedBy: L. Fortini; individualCount: 2; sex: 1 male, 1 female; lifeStage: adult; occurrenceID: AC50F90D-0142-59F4-AE23-3F2347FC556E; **Taxon:** scientificName: Lasioglossum (Sphecodogastra) malachurum (Kirby, 1802); order: Hymenoptera; family: Halictidae; genus: Lasioglossum; subgenus: Sphecodogastra; specificEpithet: malachurum; scientificNameAuthorship: (Kirby, 1802); **Location:** country: Italy; countryCode: IT; stateProvince: Roma; locality: Riserva Regionale dell'Appia Antica 2; decimalLatitude: 41.8402564; decimalLongitude: 12.532773; geodeticDatum: WGS84; coordinatePrecision: 0.0002; **Identification:** identifiedBy: M. Mei; **Event:** eventDate: 2022-08-06; **Record Level:** collectionID: UR3
- **Type status:** Other material. **Occurrence:** catalogNumber: A2100, A2101; recordedBy: L. Fortini; individualCount: 2; sex: females; lifeStage: adult; occurrenceID: 607DA092-9E8B-5723-8E76-D48084928E72; **Taxon:** scientificName: Lasioglossum (Sphecodogastra) malachurum (Kirby, 1802); order: Hymenoptera; family: Halictidae; genus: Lasioglossum; subgenus: Sphecodogastra; specificEpithet: malachurum; scientificNameAuthorship: (Kirby, 1802); **Location:** country: Italy; countryCode: IT; stateProvince: Roma; locality: Riserva Naturale Valle dell'Aniene 1; decimalLatitude: 41.9345179; decimalLongitude: 12.5453096; geodeticDatum: WGS84; coordinatePrecision: 0.0002; **Identification:** identifiedBy: M. Mei; **Event:** eventDate: 2022-08-03; **Record Level:** collectionID: UR3
- **Type status:** Other material. **Occurrence:** catalogNumber: A2119, A2120; recordedBy: L. Fortini; individualCount: 2; sex: females; lifeStage: adult; occurrenceID: C18471C7-A8CA-5D99-80FE-93585E868737; **Taxon:** scientificName: Lasioglossum (Sphecodogastra) malachurum (Kirby, 1802); order: Hymenoptera; family: Halictidae; genus: Lasioglossum; subgenus: Sphecodogastra; specificEpithet: malachurum; scientificNameAuthorship: (Kirby, 1802); **Location:** country: Italy; countryCode: IT; stateProvince: Roma; locality: Riserva Naturale Valle dell'Aniene 2; decimalLatitude: 41.928752; decimalLongitude: 12.5562962; geodeticDatum: WGS84; coordinatePrecision: 0.0002; **Identification:** identifiedBy: M. Mei; **Event:** eventDate: 2022-08-03; **Record Level:** collectionID: UR3

#### Lasioglossum medinai

(Vachal, 1895)

FBBEF3A5-BE45-5C70-93F1-4F0975FFC8B1

##### Materials

- **Type status:** Other material. **Occurrence:** catalogNumber: A1247, A1290, A1301, A1304; recordedBy: L. Fortini; individualCount: 4; sex: females; lifeStage: adult; occurrenceID: 783DC9E1-B077-5876-94EE-CF25E4AF86C8; **Taxon:** scientificName: Lasioglossum (Hemihalictus) medinai (Vachal, 1895); order: Hymenoptera; family: Halictidae; genus: Lasioglossum; subgenus: Hemihalictus; specificEpithet: medinai; scientificNameAuthorship: (Vachal, 1895); **Location:** country: Italy; countryCode: IT; stateProvince: Roma; locality: Riserva Naturale Valle dei Casali 1; decimalLatitude: 41.8710627; decimalLongitude: 12.4336809; geodeticDatum: WGS84; coordinatePrecision: 0.0002; **Identification:** identifiedBy: M. Mei; **Event:** eventDate: 2022-04-07; **Record Level:** collectionID: UR3
- **Type status:** Other material. **Occurrence:** catalogNumber: A1289; recordedBy: L. Fortini; individualCount: 1; sex: female; lifeStage: adult; occurrenceID: E91ED72A-C1DA-53DD-ADE5-FA8A8BEF8C84; **Taxon:** scientificName: Lasioglossum (Hemihalictus) medinai (Vachal, 1895); order: Hymenoptera; family: Halictidae; genus: Lasioglossum; subgenus: Hemihalictus; specificEpithet: medinai; scientificNameAuthorship: (Vachal, 1895); **Location:** country: Italy; countryCode: IT; stateProvince: Roma; locality: Riserva Naturale Valle dei Casali 2; decimalLatitude: 41.8596887; decimalLongitude: 12.4355075; geodeticDatum: WGS84; coordinatePrecision: 0.0002; **Identification:** identifiedBy: M. Mei; **Event:** eventDate: 2022-04-10; **Record Level:** collectionID: UR3
- **Type status:** Other material. **Occurrence:** catalogNumber: A1278, A1283, A1384, A1484; recordedBy: L. Fortini; individualCount: 4; sex: females; lifeStage: adult; occurrenceID: CDF05B58-3270-5058-BAD8-8D9EA4D062F6; **Taxon:** scientificName: Lasioglossum (Hemihalictus) medinai (Vachal, 1895); order: Hymenoptera; family: Halictidae; genus: Lasioglossum; subgenus: Hemihalictus; specificEpithet: medinai; scientificNameAuthorship: (Vachal, 1895); **Location:** country: Italy; countryCode: IT; stateProvince: Roma; locality: Riserva Naturale dell'Insugherata 1; decimalLatitude: 41.9555045; decimalLongitude: 12.4292321; geodeticDatum: WGS84; coordinatePrecision: 0.0002; **Identification:** identifiedBy: M. Mei; **Event:** eventDate: 2022-04-15; **Record Level:** collectionID: UR3
- **Type status:** Other material. **Occurrence:** catalogNumber: A1318, A1322, A1324, A1326, A1328; recordedBy: L. Fortini; individualCount: 5; sex: females; lifeStage: adult; occurrenceID: 892B47AC-A37B-58F5-BD6C-BB9882F66A8E; **Taxon:** scientificName: Lasioglossum (Hemihalictus) medinai (Vachal, 1895); order: Hymenoptera; family: Halictidae; genus: Lasioglossum; subgenus: Hemihalictus; specificEpithet: medinai; scientificNameAuthorship: (Vachal, 1895); **Location:** country: Italy; countryCode: IT; stateProvince: Roma; locality: Riserva Naturale di Monte Mario; decimalLatitude: 41.9386215; decimalLongitude: 12.4546223; geodeticDatum: WGS84; coordinatePrecision: 0.0002; **Identification:** identifiedBy: M. Mei; **Event:** eventDate: 2022-04-20; **Record Level:** collectionID: UR3
- **Type status:** Other material. **Occurrence:** catalogNumber: A1319; recordedBy: L. Fortini; individualCount: 1; sex: female; lifeStage: adult; occurrenceID: 02E1F4E6-EF9D-579A-BFEE-2D4DDEE606BD; **Taxon:** scientificName: Lasioglossum (Hemihalictus) medinai (Vachal, 1895); order: Hymenoptera; family: Halictidae; genus: Lasioglossum; subgenus: Hemihalictus; specificEpithet: medinai; scientificNameAuthorship: (Vachal, 1895); **Location:** country: Italy; countryCode: IT; stateProvince: Roma; locality: Riserva Naturale Tenuta dei Massimi 2; decimalLatitude: 41.8316516; decimalLongitude: 12.3999927; geodeticDatum: WGS84; coordinatePrecision: 0.0002; **Identification:** identifiedBy: M. Mei; **Event:** eventDate: 2022-05-04; **Record Level:** collectionID: UR3
- **Type status:** Other material. **Occurrence:** catalogNumber: A1398, A1400; recordedBy: L. Fortini; individualCount: 2; sex: females; lifeStage: adult; occurrenceID: A028D99E-CF3F-5A3E-A8ED-DCCDEA41DCC5; **Taxon:** scientificName: Lasioglossum (Hemihalictus) medinai (Vachal, 1895); order: Hymenoptera; family: Halictidae; genus: Lasioglossum; subgenus: Hemihalictus; specificEpithet: medinai; scientificNameAuthorship: (Vachal, 1895); **Location:** country: Italy; countryCode: IT; stateProvince: Roma; locality: Riserva Naturale Tenuta dei Massimi 1; decimalLatitude: 41.8532859; decimalLongitude: 12.3842322; geodeticDatum: WGS84; coordinatePrecision: 0.0002; **Identification:** identifiedBy: M. Mei; **Event:** eventDate: 2022-06-01; **Record Level:** collectionID: UR3
- **Type status:** Other material. **Occurrence:** catalogNumber: A0683; recordedBy: L. Fortini; individualCount: 1; sex: male; lifeStage: adult; occurrenceID: 63CA61A6-30AD-587B-B941-8B7EE4598DE8; **Taxon:** scientificName: Lasioglossum (Hemihalictus) medinai (Vachal, 1895); order: Hymenoptera; family: Halictidae; genus: Lasioglossum; subgenus: Hemihalictus; specificEpithet: medinai; scientificNameAuthorship: (Vachal, 1895); **Location:** country: Italy; countryCode: IT; stateProvince: Roma; locality: Riserva Naturale dell'Acquafredda; decimalLatitude: 41.8928408; decimalLongitude: 12.39932; geodeticDatum: WGS84; coordinatePrecision: 0.0002; **Identification:** identifiedBy: M. Mei; **Event:** eventDate: 2022-06-10; **Record Level:** collectionID: UR3
- **Type status:** Other material. **Occurrence:** catalogNumber: A1445; recordedBy: L. Fortini; individualCount: 1; sex: female; lifeStage: adult; occurrenceID: 7682C23D-3D96-5975-BB5D-E40D6305BF03; **Taxon:** scientificName: Lasioglossum (Hemihalictus) medinai (Vachal, 1895); order: Hymenoptera; family: Halictidae; genus: Lasioglossum; subgenus: Hemihalictus; specificEpithet: medinai; scientificNameAuthorship: (Vachal, 1895); **Location:** country: Italy; countryCode: IT; stateProvince: Roma; locality: Riserva Naturale Tenuta dei Massimi 1; decimalLatitude: 41.8532859; decimalLongitude: 12.3842322; geodeticDatum: WGS84; coordinatePrecision: 0.0002; **Identification:** identifiedBy: M. Mei; **Event:** eventDate: 2022-06-27; **Record Level:** collectionID: UR3
- **Type status:** Other material. **Occurrence:** catalogNumber: A1433; recordedBy: L. Fortini; individualCount: 1; sex: male; lifeStage: adult; occurrenceID: 340FBBFC-4B8E-5CCC-9B5F-47F98ABC158E; **Taxon:** scientificName: Lasioglossum (Hemihalictus) medinai (Vachal, 1895); order: Hymenoptera; family: Halictidae; genus: Lasioglossum; subgenus: Hemihalictus; specificEpithet: medinai; scientificNameAuthorship: (Vachal, 1895); **Location:** country: Italy; countryCode: IT; stateProvince: Roma; locality: Riserva Naturale Valle dell'Aniene 2; decimalLatitude: 41.928752; decimalLongitude: 12.5562962; geodeticDatum: WGS84; coordinatePrecision: 0.0002; **Identification:** identifiedBy: M. Mei; **Event:** eventDate: 2022-07-01; **Record Level:** collectionID: UR3
- **Type status:** Other material. **Occurrence:** catalogNumber: A1453; recordedBy: L. Fortini; individualCount: 1; sex: male; lifeStage: adult; occurrenceID: 41761FDA-8400-527E-8629-366EA4B33984; **Taxon:** scientificName: Lasioglossum (Hemihalictus) medinai (Vachal, 1895); order: Hymenoptera; family: Halictidae; genus: Lasioglossum; subgenus: Hemihalictus; specificEpithet: medinai; scientificNameAuthorship: (Vachal, 1895); **Location:** country: Italy; countryCode: IT; stateProvince: Roma; locality: Riserva Regionale dell'Appia Antica 2; decimalLatitude: 41.8402564; decimalLongitude: 12.532773; geodeticDatum: WGS84; coordinatePrecision: 0.0002; **Identification:** identifiedBy: M. Mei; **Event:** eventDate: 2022-07-07; **Record Level:** collectionID: UR3
- **Type status:** Other material. **Occurrence:** catalogNumber: A1481, A1586, A1587; recordedBy: L. Fortini; individualCount: 3; sex: 2 males, 1 female; lifeStage: adult; occurrenceID: E23F28A0-20E8-52CC-B0CF-48F6ADC979F4; **Taxon:** scientificName: Lasioglossum (Hemihalictus) medinai (Vachal, 1895); order: Hymenoptera; family: Halictidae; genus: Lasioglossum; subgenus: Hemihalictus; specificEpithet: medinai; scientificNameAuthorship: (Vachal, 1895); **Location:** country: Italy; countryCode: IT; stateProvince: Roma; locality: Riserva Naturale dell'Acquafredda; decimalLatitude: 41.8928408; decimalLongitude: 12.39932; geodeticDatum: WGS84; coordinatePrecision: 0.0002; **Identification:** identifiedBy: M. Mei; **Event:** eventDate: 2022-07-12; **Record Level:** collectionID: UR3
- **Type status:** Other material. **Occurrence:** catalogNumber: A1566; recordedBy: L. Fortini; individualCount: 1; sex: male; lifeStage: adult; occurrenceID: DC4CA4BE-6B7A-5A0C-9452-3BB9C8E35163; **Taxon:** scientificName: Lasioglossum (Hemihalictus) medinai (Vachal, 1895); order: Hymenoptera; family: Halictidae; genus: Lasioglossum; subgenus: Hemihalictus; specificEpithet: medinai; scientificNameAuthorship: (Vachal, 1895); **Location:** country: Italy; countryCode: IT; stateProvince: Roma; locality: Riserva Naturale Valle dei Casali 2; decimalLatitude: 41.8596887; decimalLongitude: 12.4355075; geodeticDatum: WGS84; coordinatePrecision: 0.0002; **Identification:** identifiedBy: M. Mei; **Event:** eventDate: 2022-07-13; **Record Level:** collectionID: UR3
- **Type status:** Other material. **Occurrence:** catalogNumber: A1470; recordedBy: L. Fortini; individualCount: 1; sex: male; lifeStage: adult; occurrenceID: 9CD9B6A7-2931-5C70-B32A-3F4A258B4417; **Taxon:** scientificName: Lasioglossum (Hemihalictus) medinai (Vachal, 1895); order: Hymenoptera; family: Halictidae; genus: Lasioglossum; subgenus: Hemihalictus; specificEpithet: medinai; scientificNameAuthorship: (Vachal, 1895); **Location:** country: Italy; countryCode: IT; stateProvince: Roma; locality: Riserva Naturale Tenuta dei Massimi 1; decimalLatitude: 41.8532859; decimalLongitude: 12.3842322; geodeticDatum: WGS84; coordinatePrecision: 0.0002; **Identification:** identifiedBy: M. Mei; **Event:** eventDate: 2022-07-28; **Record Level:** collectionID: UR3
- **Type status:** Other material. **Occurrence:** catalogNumber: A1537; recordedBy: L. Fortini; individualCount: 1; sex: male; lifeStage: adult; occurrenceID: 4F02A602-FF8C-5601-8E06-09C0291C5AE9; **Taxon:** scientificName: Lasioglossum (Hemihalictus) medinai (Vachal, 1895); order: Hymenoptera; family: Halictidae; genus: Lasioglossum; subgenus: Hemihalictus; specificEpithet: medinai; scientificNameAuthorship: (Vachal, 1895); **Location:** country: Italy; countryCode: IT; stateProvince: Roma; locality: Riserva Naturale Tenuta dei Massimi 2; decimalLatitude: 41.8316516; decimalLongitude: 12.3999927; geodeticDatum: WGS84; coordinatePrecision: 0.0002; **Identification:** identifiedBy: M. Mei; **Event:** eventDate: 2022-07-28; **Record Level:** collectionID: UR3
- **Type status:** Other material. **Occurrence:** catalogNumber: A1492; recordedBy: L. Fortini; individualCount: 1; sex: female; lifeStage: adult; occurrenceID: 4ACC1956-C39B-5057-A4AB-E4D4DA1A15ED; **Taxon:** scientificName: Lasioglossum (Hemihalictus) medinai (Vachal, 1895); order: Hymenoptera; family: Halictidae; genus: Lasioglossum; subgenus: Hemihalictus; specificEpithet: medinai; scientificNameAuthorship: (Vachal, 1895); **Location:** country: Italy; countryCode: IT; stateProvince: Roma; locality: Riserva Naturale dell'Insugherata 3; decimalLatitude: 41.9644829; decimalLongitude: 12.436101; geodeticDatum: WGS84; coordinatePrecision: 0.0002; **Identification:** identifiedBy: M. Mei; **Event:** eventDate: 2022-07-01; **Record Level:** collectionID: UR3

#### Lasioglossum minutissimum

(Kirby, 1802)

A50FFD3A-B496-5106-B4EE-E1E54C4E396F

##### Materials

- **Type status:** Other material. **Occurrence:** catalogNumber: A1282; recordedBy: L. Fortini; individualCount: 1; sex: female; lifeStage: adult; occurrenceID: E7E1E90F-C1CC-522C-8F46-18DEE53279F1; **Taxon:** scientificName: Lasioglossum (Hemihalictus) minutissimum (Kirby, 1802); order: Hymenoptera; family: Halictidae; genus: Lasioglossum; subgenus: Hemihalictus; specificEpithet: minutissimum; scientificNameAuthorship: (Kirby, 1802); **Location:** country: Italy; countryCode: IT; stateProvince: Roma; locality: Riserva Naturale dell'Insugherata 3; decimalLatitude: 41.9644829; decimalLongitude: 12.436101; geodeticDatum: WGS84; coordinatePrecision: 0.0002; **Identification:** identifiedBy: M. Mei; **Event:** eventDate: 2022-04-15; **Record Level:** collectionID: UR3
- **Type status:** Other material. **Occurrence:** catalogNumber: A1544; recordedBy: L. Fortini; individualCount: 1; sex: female; lifeStage: adult; occurrenceID: BEBDE7C8-5788-54F7-AB67-7BCC041389E9; **Taxon:** scientificName: Lasioglossum (Hemihalictus) minutissimum (Kirby, 1802); order: Hymenoptera; family: Halictidae; genus: Lasioglossum; subgenus: Hemihalictus; specificEpithet: minutissimum; scientificNameAuthorship: (Kirby, 1802); **Location:** country: Italy; countryCode: IT; stateProvince: Roma; locality: Riserva Naturale dell'Acquafredda; decimalLatitude: 41.8928408; decimalLongitude: 12.39932; geodeticDatum: WGS84; coordinatePrecision: 0.0002; **Identification:** identifiedBy: M. Mei; **Event:** eventDate: 2022-08-17; **Record Level:** collectionID: UR3

#### Lasioglossum morio

(Fabricius, 1793)

438730AF-6C77-55AA-938A-E67E0C2761B1

##### Materials

- **Type status:** Other material. **Occurrence:** catalogNumber: A1533, A1542; recordedBy: L. Fortini; individualCount: 2; sex: females; lifeStage: adult; occurrenceID: AAFA7235-55DB-52BC-86E5-84087C8503F5; **Taxon:** scientificName: Lasioglossum (Dialictus) morio (Fabricius, 1793); order: Hymenoptera; family: Halictidae; genus: Lasioglossum; subgenus: Dialictus; specificEpithet: morio; scientificNameAuthorship: (Fabricius, 1793); **Location:** country: Italy; countryCode: IT; stateProvince: Roma; locality: Riserva Naturale dell'Acquafredda; decimalLatitude: 41.8928408; decimalLongitude: 12.39932; geodeticDatum: WGS84; coordinatePrecision: 0.0002; **Identification:** identifiedBy: M. Mei; **Event:** eventDate: 2022-08-17; **Record Level:** collectionID: UR3
- **Type status:** Other material. **Occurrence:** catalogNumber: A2054; recordedBy: L. Fortini; individualCount: 1; sex: female; lifeStage: adult; occurrenceID: 1949EAF9-1917-5FA2-BB56-E8EBBC0C5D91; **Taxon:** scientificName: Lasioglossum (Dialictus) morio (Fabricius, 1793); order: Hymenoptera; family: Halictidae; genus: Lasioglossum; subgenus: Dialictus; specificEpithet: morio; scientificNameAuthorship: (Fabricius, 1793); **Location:** country: Italy; countryCode: IT; stateProvince: Roma; locality: Riserva Naturale dell'Insugherata 3; decimalLatitude: 41.9644829; decimalLongitude: 12.436101; geodeticDatum: WGS84; coordinatePrecision: 0.0002; **Identification:** identifiedBy: M. Mei; **Event:** eventDate: 2022-07-30; **Record Level:** collectionID: UR3

#### Lasioglossum nigripes

(Lepeletier, 1841)

01A4105A-B096-5903-AE5B-7FF42B4F5966

##### Materials

- **Type status:** Other material. **Occurrence:** catalogNumber: A1527, A1631, A1632, A1794; recordedBy: L. Fortini; individualCount: 4; sex: 2 males, 2 females; lifeStage: adult; occurrenceID: FD3D5038-7D71-5A3C-812F-7D661BD537C4; **Taxon:** scientificName: Lasioglossum (Sphecodogastra) nigripes (Lepeletier, 1841); order: Hymenoptera; family: Halictidae; genus: Lasioglossum; subgenus: Sphecodogastra; specificEpithet: nigripes; scientificNameAuthorship: (Lepeletier, 1841); **Location:** country: Italy; countryCode: IT; stateProvince: Roma; locality: Riserva Naturale dell'Acquafredda; decimalLatitude: 41.8928408; decimalLongitude: 12.39932; geodeticDatum: WGS84; coordinatePrecision: 0.0002; **Identification:** identifiedBy: M. Mei; **Event:** eventDate: 2022-07-12; **Record Level:** collectionID: UR3
- **Type status:** Other material. **Occurrence:** catalogNumber: A1805; recordedBy: L. Fortini; individualCount: 1; sex: female; lifeStage: adult; occurrenceID: A9C536CD-7453-5F5A-A400-D094B7A62A44; **Taxon:** scientificName: Lasioglossum (Sphecodogastra) nigripes (Lepeletier, 1841); order: Hymenoptera; family: Halictidae; genus: Lasioglossum; subgenus: Sphecodogastra; specificEpithet: nigripes; scientificNameAuthorship: (Lepeletier, 1841); **Location:** country: Italy; countryCode: IT; stateProvince: Roma; locality: Riserva Naturale dell'Acquafredda; decimalLatitude: 41.8928408; decimalLongitude: 12.39932; geodeticDatum: WGS84; coordinatePrecision: 0.0002; **Identification:** identifiedBy: M. Mei; **Event:** eventDate: 2022-08-17; **Record Level:** collectionID: UR3
- **Type status:** Other material. **Occurrence:** catalogNumber: A1455, A1465, A1786, A1787, A1788, A1790, A1793, A1798, A1801, A1803, A1804, A1806, A1807, A1808, A1809; recordedBy: L. Fortini; individualCount: 15; sex: females; lifeStage: adult; occurrenceID: 0CE76511-F160-5212-9372-4C7297A025DF; **Taxon:** scientificName: Lasioglossum (Sphecodogastra) nigripes (Lepeletier, 1841); order: Hymenoptera; family: Halictidae; genus: Lasioglossum; subgenus: Sphecodogastra; specificEpithet: nigripes; scientificNameAuthorship: (Lepeletier, 1841); **Location:** country: Italy; countryCode: IT; stateProvince: Roma; locality: Riserva Regionale dell'Appia Antica 2; decimalLatitude: 41.8402564; decimalLongitude: 12.532773; geodeticDatum: WGS84; coordinatePrecision: 0.0002; **Identification:** identifiedBy: M. Mei; **Event:** eventDate: 2022-07-07; **Record Level:** collectionID: UR3
- **Type status:** Other material. **Occurrence:** catalogNumber: A1619, A1797; recordedBy: L. Fortini; individualCount: 2; sex: females; lifeStage: adult; occurrenceID: 158F431A-472D-5AC5-9E1C-C5F15145A124; **Taxon:** scientificName: Lasioglossum (Sphecodogastra) nigripes (Lepeletier, 1841); order: Hymenoptera; family: Halictidae; genus: Lasioglossum; subgenus: Sphecodogastra; specificEpithet: nigripes; scientificNameAuthorship: (Lepeletier, 1841); **Location:** country: Italy; countryCode: IT; stateProvince: Roma; locality: Riserva Regionale dell'Appia Antica 2; decimalLatitude: 41.8402564; decimalLongitude: 12.532773; geodeticDatum: WGS84; coordinatePrecision: 0.0002; **Identification:** identifiedBy: M. Mei; **Event:** eventDate: 2022-08-06; **Record Level:** collectionID: UR3
- **Type status:** Other material. **Occurrence:** catalogNumber: A1792; recordedBy: L. Fortini; individualCount: 1; sex: female; lifeStage: adult; occurrenceID: 30D4F179-86F8-58D1-B737-C35C5AC1FB8C; **Taxon:** scientificName: Lasioglossum (Sphecodogastra) nigripes (Lepeletier, 1841); order: Hymenoptera; family: Halictidae; genus: Lasioglossum; subgenus: Sphecodogastra; specificEpithet: nigripes; scientificNameAuthorship: (Lepeletier, 1841); **Location:** country: Italy; countryCode: IT; stateProvince: Roma; locality: Riserva Naturale dell'Insugherata 3; decimalLatitude: 41.9644829; decimalLongitude: 12.436101; geodeticDatum: WGS84; coordinatePrecision: 0.0002; **Identification:** identifiedBy: M. Mei; **Event:** eventDate: 2022-06-24; **Record Level:** collectionID: UR3
- **Type status:** Other material. **Occurrence:** catalogNumber: A1715; recordedBy: L. Fortini; individualCount: 1; sex: female; lifeStage: adult; occurrenceID: BE08E51C-D879-5D14-8BBF-D725C41C776D; **Taxon:** scientificName: Lasioglossum (Sphecodogastra) nigripes (Lepeletier, 1841); order: Hymenoptera; family: Halictidae; genus: Lasioglossum; subgenus: Sphecodogastra; specificEpithet: nigripes; scientificNameAuthorship: (Lepeletier, 1841); **Location:** country: Italy; countryCode: IT; stateProvince: Roma; locality: Riserva Naturale Tenuta dei Massimi 1; decimalLatitude: 41.8532859; decimalLongitude: 12.3842322; geodeticDatum: WGS84; coordinatePrecision: 0.0002; **Identification:** identifiedBy: M. Mei; **Event:** eventDate: 2022-04-23; **Record Level:** collectionID: UR3
- **Type status:** Other material. **Occurrence:** catalogNumber: A1791; recordedBy: L. Fortini; individualCount: 1; sex: female; lifeStage: adult; occurrenceID: 9656E0F7-9432-52D4-BDFA-D7F634BC92C2; **Taxon:** scientificName: Lasioglossum (Sphecodogastra) nigripes (Lepeletier, 1841); order: Hymenoptera; family: Halictidae; genus: Lasioglossum; subgenus: Sphecodogastra; specificEpithet: nigripes; scientificNameAuthorship: (Lepeletier, 1841); **Location:** country: Italy; countryCode: IT; stateProvince: Roma; locality: Riserva Naturale Tenuta dei Massimi 1; decimalLatitude: 41.8532859; decimalLongitude: 12.3842322; geodeticDatum: WGS84; coordinatePrecision: 0.0002; **Identification:** identifiedBy: M. Mei; **Event:** eventDate: 2022-06-27; **Record Level:** collectionID: UR3
- **Type status:** Other material. **Occurrence:** catalogNumber: A1789, A1795, A1799, A1800, A1802; recordedBy: L. Fortini; individualCount: 5; sex: females; lifeStage: adult; occurrenceID: 44482588-F543-5CCE-8FDF-09308BEC07AB; **Taxon:** scientificName: Lasioglossum (Sphecodogastra) nigripes (Lepeletier, 1841); order: Hymenoptera; family: Halictidae; genus: Lasioglossum; subgenus: Sphecodogastra; specificEpithet: nigripes; scientificNameAuthorship: (Lepeletier, 1841); **Location:** country: Italy; countryCode: IT; stateProvince: Roma; locality: Riserva Naturale Valle dell'Aniene 2; decimalLatitude: 41.928752; decimalLongitude: 12.5562962; geodeticDatum: WGS84; coordinatePrecision: 0.0002; **Identification:** identifiedBy: M. Mei; **Event:** eventDate: 2022-07-01; **Record Level:** collectionID: UR3
- **Type status:** Other material. **Occurrence:** catalogNumber: A1796; recordedBy: L. Fortini; individualCount: 1; sex: female; lifeStage: adult; occurrenceID: 313E833B-D972-55F7-8DF3-BFA305F3E4F4; **Taxon:** scientificName: Lasioglossum (Sphecodogastra) nigripes (Lepeletier, 1841); order: Hymenoptera; family: Halictidae; genus: Lasioglossum; subgenus: Sphecodogastra; specificEpithet: nigripes; scientificNameAuthorship: (Lepeletier, 1841); **Location:** country: Italy; countryCode: IT; stateProvince: Roma; locality: Riserva Naturale Valle dell'Aniene 2; decimalLatitude: 41.928752; decimalLongitude: 12.5562962; geodeticDatum: WGS84; coordinatePrecision: 0.0002; **Identification:** identifiedBy: M. Mei; **Event:** eventDate: 2022-09-04; **Record Level:** collectionID: UR3
- **Type status:** Other material. **Occurrence:** catalogNumber: A2122, A2128; recordedBy: L. Fortini; individualCount: 2; sex: females; lifeStage: adult; occurrenceID: 9FC167C1-92D9-549D-865A-67D5938F5A51; **Taxon:** scientificName: Lasioglossum (Sphecodogastra) nigripes (Lepeletier, 1841); order: Hymenoptera; family: Halictidae; genus: Lasioglossum; subgenus: Sphecodogastra; specificEpithet: nigripes; scientificNameAuthorship: (Lepeletier, 1841); **Location:** country: Italy; countryCode: IT; stateProvince: Roma; locality: Riserva Naturale Valle dell'Aniene 2; decimalLatitude: 41.928752; decimalLongitude: 12.5562962; geodeticDatum: WGS84; coordinatePrecision: 0.0002; **Identification:** identifiedBy: M. Mei; **Event:** eventDate: 2022-08-03; **Record Level:** collectionID: UR3

#### Lasioglossum nitidulum

(Fabricius, 1804)

11BC3356-ABD9-56A3-90AB-97DD568F991C

##### Materials

- **Type status:** Other material. **Occurrence:** catalogNumber: A1561; recordedBy: L. Fortini; individualCount: 1; sex: female; lifeStage: adult; occurrenceID: F790C853-3A2D-5024-A268-F2794C3C6C54; **Taxon:** scientificName: Lasioglossum (Dialictus) nitidulum (Fabricius, 1804); order: Hymenoptera; family: Halictidae; genus: Lasioglossum; subgenus: Dialictus; specificEpithet: nitidulum; scientificNameAuthorship: (Fabricius, 1804); **Location:** country: Italy; countryCode: IT; stateProvince: Roma; locality: Riserva Regionale dell'Appia Antica 1; decimalLatitude: 41.8623941; decimalLongitude: 12.524863; geodeticDatum: WGS84; coordinatePrecision: 0.0002; **Identification:** identifiedBy: M. Mei; **Event:** eventDate: 2022-08-25; **Record Level:** collectionID: UR3
- **Type status:** Other material. **Occurrence:** catalogNumber: A2045; recordedBy: L. Fortini; individualCount: 1; sex: female; lifeStage: adult; occurrenceID: A0CE302E-20FB-5CED-8305-F11B344820EF; **Taxon:** scientificName: Lasioglossum (Dialictus) nitidulum (Fabricius, 1804); order: Hymenoptera; family: Halictidae; genus: Lasioglossum; subgenus: Dialictus; specificEpithet: nitidulum; scientificNameAuthorship: (Fabricius, 1804); **Location:** country: Italy; countryCode: IT; stateProvince: Roma; locality: Riserva Naturale Valle dei Casali 1; decimalLatitude: 41.8710627; decimalLongitude: 12.4336809; geodeticDatum: WGS84; coordinatePrecision: 0.0002; **Identification:** identifiedBy: M. Mei; **Event:** eventDate: 2022-08-19; **Record Level:** collectionID: UR3

#### Lasioglossum pauperatum

(Brullé, 1832)

52A4F477-AAF8-57D3-9D62-F86571CD5242

##### Materials

- **Type status:** Other material. **Occurrence:** catalogNumber: A1316; recordedBy: L. Fortini; individualCount: 1; sex: female; lifeStage: adult; occurrenceID: 15C91424-BBCC-5627-A226-DB5B28CA12C0; **Taxon:** scientificName: Lasioglossum (Hemihalictus) pauperatum (Brullé, 1832); order: Hymenoptera; family: Halictidae; genus: Lasioglossum; subgenus: Hemihalictus; specificEpithet: pauperatum; scientificNameAuthorship: (Brullé, 1832); **Location:** country: Italy; countryCode: IT; stateProvince: Roma; locality: Riserva Naturale Tenuta dei Massimi 1; decimalLatitude: 41.8532859; decimalLongitude: 12.3842322; geodeticDatum: WGS84; coordinatePrecision: 0.0002; **Identification:** identifiedBy: M. Mei; **Event:** eventDate: 2022-04-23; **Record Level:** collectionID: UR3
- **Type status:** Other material. **Occurrence:** catalogNumber: A1275, A1276; recordedBy: L. Fortini; individualCount: 2; sex: females; lifeStage: adult; occurrenceID: AE9D4EDF-F55A-589E-8F4F-7B9E9D94091A; **Taxon:** scientificName: Lasioglossum (Hemihalictus) pauperatum (Brullé, 1832); order: Hymenoptera; family: Halictidae; genus: Lasioglossum; subgenus: Hemihalictus; specificEpithet: pauperatum; scientificNameAuthorship: (Brullé, 1832); **Location:** country: Italy; countryCode: IT; stateProvince: Roma; locality: Riserva Naturale Valle dell'Aniene 1; decimalLatitude: 41.9345179; decimalLongitude: 12.5453096; geodeticDatum: WGS84; coordinatePrecision: 0.0002; **Identification:** identifiedBy: M. Mei; **Event:** eventDate: 2022-04-28; **Record Level:** collectionID: UR3
- **Type status:** Other material. **Occurrence:** catalogNumber: A1263; recordedBy: L. Fortini; individualCount: 1; sex: female; lifeStage: adult; occurrenceID: 315F7021-8233-5533-8A52-36E26B39B698; **Taxon:** scientificName: Lasioglossum (Hemihalictus) pauperatum (Brullé, 1832); order: Hymenoptera; family: Halictidae; genus: Lasioglossum; subgenus: Hemihalictus; specificEpithet: pauperatum; scientificNameAuthorship: (Brullé, 1832); **Location:** country: Italy; countryCode: IT; stateProvince: Roma; locality: Riserva Regionale dell'Appia Antica 3; decimalLatitude: 41.8298456; decimalLongitude: 12.5432538; geodeticDatum: WGS84; coordinatePrecision: 0.0002; **Identification:** identifiedBy: M. Mei; **Event:** eventDate: 2022-05-24; **Record Level:** collectionID: UR3
- **Type status:** Other material. **Occurrence:** catalogNumber: A1356, A1383; recordedBy: L. Fortini; individualCount: 2; sex: females; lifeStage: adult; occurrenceID: 33F2CB54-1557-529D-9D82-E3270B7F9CF6; **Taxon:** scientificName: Lasioglossum (Hemihalictus) pauperatum (Brullé, 1832); order: Hymenoptera; family: Halictidae; genus: Lasioglossum; subgenus: Hemihalictus; specificEpithet: pauperatum; scientificNameAuthorship: (Brullé, 1832); **Location:** country: Italy; countryCode: IT; stateProvince: Roma; locality: Riserva Naturale Valle dell'Aniene 2; decimalLatitude: 41.928752; decimalLongitude: 12.5562962; geodeticDatum: WGS84; coordinatePrecision: 0.0002; **Identification:** identifiedBy: M. Mei; **Event:** eventDate: 2022-06-05; **Record Level:** collectionID: UR3
- **Type status:** Other material. **Occurrence:** catalogNumber: A1357; recordedBy: L. Fortini; individualCount: 1; sex: male; lifeStage: adult; occurrenceID: 19144C5C-8EDA-5951-BEED-F4E24B6B3058; **Taxon:** scientificName: Lasioglossum (Hemihalictus) pauperatum (Brullé, 1832); order: Hymenoptera; family: Halictidae; genus: Lasioglossum; subgenus: Hemihalictus; specificEpithet: pauperatum; scientificNameAuthorship: (Brullé, 1832); **Location:** country: Italy; countryCode: IT; stateProvince: Roma; locality: Riserva Naturale dell'Acquafredda; decimalLatitude: 41.8928408; decimalLongitude: 12.39932; geodeticDatum: WGS84; coordinatePrecision: 0.0002; **Identification:** identifiedBy: M. Mei; **Event:** eventDate: 2022-06-10; **Record Level:** collectionID: UR3
- **Type status:** Other material. **Occurrence:** catalogNumber: A1369; recordedBy: L. Fortini; individualCount: 1; sex: females; lifeStage: adult; occurrenceID: D1F566EC-80EC-5C9E-A8FE-1206B31C5B7F; **Taxon:** scientificName: Lasioglossum (Hemihalictus) pauperatum (Brullé, 1832); order: Hymenoptera; family: Halictidae; genus: Lasioglossum; subgenus: Hemihalictus; specificEpithet: pauperatum; scientificNameAuthorship: (Brullé, 1832); **Location:** country: Italy; countryCode: IT; stateProvince: Roma; locality: Riserva Naturale dell'Insugherata 2; decimalLatitude: 41.9599247; decimalLongitude: 12.433852; geodeticDatum: WGS84; coordinatePrecision: 0.0002; **Identification:** identifiedBy: M. Mei; **Event:** eventDate: 2022-06-24; **Record Level:** collectionID: UR3
- **Type status:** Other material. **Occurrence:** catalogNumber: A2108, A2109; recordedBy: L. Fortini; individualCount: 2; sex: males; lifeStage: adult; occurrenceID: 46543982-8E88-5144-B84C-83BA0DFCCA0A; **Taxon:** scientificName: Lasioglossum (Hemihalictus) pauperatum (Brullé, 1832); order: Hymenoptera; family: Halictidae; genus: Lasioglossum; subgenus: Hemihalictus; specificEpithet: pauperatum; scientificNameAuthorship: (Brullé, 1832); **Location:** country: Italy; countryCode: IT; stateProvince: Roma; locality: Riserva Naturale Valle dell'Aniene 1; decimalLatitude: 41.9345179; decimalLongitude: 12.5453096; geodeticDatum: WGS84; coordinatePrecision: 0.0002; **Identification:** identifiedBy: M. Mei; **Event:** eventDate: 2022-08-03; **Record Level:** collectionID: UR3
- **Type status:** Other material. **Occurrence:** catalogNumber: A2127; recordedBy: L. Fortini; individualCount: 1; sex: female; lifeStage: adult; occurrenceID: 2B42CE1A-1CA4-54C2-B87A-65A6CE8DFCD1; **Taxon:** scientificName: Lasioglossum (Hemihalictus) pauperatum (Brullé, 1832); order: Hymenoptera; family: Halictidae; genus: Lasioglossum; subgenus: Hemihalictus; specificEpithet: pauperatum; scientificNameAuthorship: (Brullé, 1832); **Location:** country: Italy; countryCode: IT; stateProvince: Roma; locality: Riserva Naturale Valle dell'Aniene 2; decimalLatitude: 41.928752; decimalLongitude: 12.5562962; geodeticDatum: WGS84; coordinatePrecision: 0.0002; **Identification:** identifiedBy: M. Mei; **Event:** eventDate: 2022-08-03; **Record Level:** collectionID: UR3
- **Type status:** Other material. **Occurrence:** catalogNumber: A1519; recordedBy: L. Fortini; individualCount: 1; sex: female; lifeStage: adult; occurrenceID: AD201034-5ACE-5B36-B2B3-230E4A98FE9C; **Taxon:** scientificName: Lasioglossum (Hemihalictus) pauperatum (Brullé, 1832); order: Hymenoptera; family: Halictidae; genus: Lasioglossum; subgenus: Hemihalictus; specificEpithet: pauperatum; scientificNameAuthorship: (Brullé, 1832); **Location:** country: Italy; countryCode: IT; stateProvince: Roma; locality: Riserva Naturale Laurentino-Acqua Acetosa; decimalLatitude: 41.8079275; decimalLongitude: 12.4685548; geodeticDatum: WGS84; coordinatePrecision: 0.0002; **Identification:** identifiedBy: M. Mei; **Event:** eventDate: 2022-08-21; **Record Level:** collectionID: UR3

#### Lasioglossum pauxillum

(Schenck, 1853)

6FAB5911-6491-55AA-85C0-B44058D07383

##### Materials

- **Type status:** Other material. **Occurrence:** catalogNumber: A1307; recordedBy: L. Fortini; individualCount: 1; sex: female; lifeStage: adult; occurrenceID: D2F7DC7E-40C6-5266-845A-9DE0AAA367F9; **Taxon:** scientificName: Lasioglossum (Sphecodogastra) pauxillum (Schenck, 1853); order: Hymenoptera; family: Halictidae; genus: Lasioglossum; subgenus: Sphecodogastra; specificEpithet: pauxillum; scientificNameAuthorship: (Schenck, 1853); **Location:** country: Italy; countryCode: IT; stateProvince: Roma; locality: Riserva Naturale Valle dei Casali 1; decimalLatitude: 41.8710627; decimalLongitude: 12.4336809; geodeticDatum: WGS84; coordinatePrecision: 0.0002; **Identification:** identifiedBy: M. Mei; **Event:** eventDate: 2022-04-07; **Record Level:** collectionID: UR3
- **Type status:** Other material. **Occurrence:** catalogNumber: A1281, A1285; recordedBy: L. Fortini; individualCount: 2; sex: females; lifeStage: adult; occurrenceID: F3CF213A-9DE8-5344-A6D1-DC8079C4909E; **Taxon:** scientificName: Lasioglossum (Sphecodogastra) pauxillum (Schenck, 1853); order: Hymenoptera; family: Halictidae; genus: Lasioglossum; subgenus: Sphecodogastra; specificEpithet: pauxillum; scientificNameAuthorship: (Schenck, 1853); **Location:** country: Italy; countryCode: IT; stateProvince: Roma; locality: Riserva Naturale dell'Insugherata 3; decimalLatitude: 41.9644829; decimalLongitude: 12.436101; geodeticDatum: WGS84; coordinatePrecision: 0.0002; **Identification:** identifiedBy: M. Mei; **Event:** eventDate: 2022-04-15; **Record Level:** collectionID: UR3
- **Type status:** Other material. **Occurrence:** catalogNumber: A1317, A1341; recordedBy: L. Fortini; individualCount: 2; sex: females; lifeStage: adult; occurrenceID: 1CDF111C-49B7-537E-BBD1-88CAAF6DF37F; **Taxon:** scientificName: Lasioglossum (Sphecodogastra) pauxillum (Schenck, 1853); order: Hymenoptera; family: Halictidae; genus: Lasioglossum; subgenus: Sphecodogastra; specificEpithet: pauxillum; scientificNameAuthorship: (Schenck, 1853); **Location:** country: Italy; countryCode: IT; stateProvince: Roma; locality: Riserva Naturale di Monte Mario; decimalLatitude: 41.9386215; decimalLongitude: 12.4546223; geodeticDatum: WGS84; coordinatePrecision: 0.0002; **Identification:** identifiedBy: M. Mei; **Event:** eventDate: 2022-04-20; **Record Level:** collectionID: UR3
- **Type status:** Other material. **Occurrence:** catalogNumber: A1297, A1309, A1315; recordedBy: L. Fortini; individualCount: 3; sex: females; lifeStage: adult; occurrenceID: 1A3B3024-C782-5FD1-8132-CCA628762745; **Taxon:** scientificName: Lasioglossum (Sphecodogastra) pauxillum (Schenck, 1853); order: Hymenoptera; family: Halictidae; genus: Lasioglossum; subgenus: Sphecodogastra; specificEpithet: pauxillum; scientificNameAuthorship: (Schenck, 1853); **Location:** country: Italy; countryCode: IT; stateProvince: Roma; locality: Riserva Naturale Tenuta dei Massimi 1; decimalLatitude: 41.8532859; decimalLongitude: 12.3842322; geodeticDatum: WGS84; coordinatePrecision: 0.0002; **Identification:** identifiedBy: M. Mei; **Event:** eventDate: 2022-04-23; **Record Level:** collectionID: UR3
- **Type status:** Other material. **Occurrence:** catalogNumber: A1327, A1343; recordedBy: L. Fortini; individualCount: 2; sex: females; lifeStage: adult; occurrenceID: C9AD1669-19F2-5E20-993C-A61D4740AD30; **Taxon:** scientificName: Lasioglossum (Sphecodogastra) pauxillum (Schenck, 1853); order: Hymenoptera; family: Halictidae; genus: Lasioglossum; subgenus: Sphecodogastra; specificEpithet: pauxillum; scientificNameAuthorship: (Schenck, 1853); **Location:** country: Italy; countryCode: IT; stateProvince: Roma; locality: Riserva Naturale di Monte Mario; decimalLatitude: 41.9386215; decimalLongitude: 12.4546223; geodeticDatum: WGS84; coordinatePrecision: 0.0002; **Identification:** identifiedBy: M. Mei; **Event:** eventDate: 2022-05-20; **Record Level:** collectionID: UR3
- **Type status:** Other material. **Occurrence:** catalogNumber: A1249, A1251, A1252, A1253, A1254, A1256, A1259; recordedBy: L. Fortini; individualCount: 7; sex: females; lifeStage: adult; occurrenceID: 8ED5883F-59F2-515B-90C0-BB119570BF6B; **Taxon:** scientificName: Lasioglossum (Sphecodogastra) pauxillum (Schenck, 1853); order: Hymenoptera; family: Halictidae; genus: Lasioglossum; subgenus: Sphecodogastra; specificEpithet: pauxillum; scientificNameAuthorship: (Schenck, 1853); **Location:** country: Italy; countryCode: IT; stateProvince: Roma; locality: Riserva Regionale dell'Appia Antica 2; decimalLatitude: 41.8402564; decimalLongitude: 12.532773; geodeticDatum: WGS84; coordinatePrecision: 0.0002; **Identification:** identifiedBy: M. Mei; **Event:** eventDate: 2022-05-24; **Record Level:** collectionID: UR3
- **Type status:** Other material. **Occurrence:** catalogNumber: A1257, A1258, A1261; recordedBy: L. Fortini; individualCount: 3; sex: females; lifeStage: adult; occurrenceID: A216F372-1EA5-527B-BCDB-C0BFE352D6C1; **Taxon:** scientificName: Lasioglossum (Sphecodogastra) pauxillum (Schenck, 1853); order: Hymenoptera; family: Halictidae; genus: Lasioglossum; subgenus: Sphecodogastra; specificEpithet: pauxillum; scientificNameAuthorship: (Schenck, 1853); **Location:** country: Italy; countryCode: IT; stateProvince: Roma; locality: Riserva Regionale dell'Appia Antica 3; decimalLatitude: 41.8298456; decimalLongitude: 12.5432538; geodeticDatum: WGS84; coordinatePrecision: 0.0002; **Identification:** identifiedBy: M. Mei; **Event:** eventDate: 2022-05-24; **Record Level:** collectionID: UR3
- **Type status:** Other material. **Occurrence:** catalogNumber: A1407; recordedBy: L. Fortini; individualCount: 1; sex: female; lifeStage: adult; occurrenceID: 77B4FEB2-820F-52CA-88A6-640927BD1215; **Taxon:** scientificName: Lasioglossum (Sphecodogastra) pauxillum (Schenck, 1853); order: Hymenoptera; family: Halictidae; genus: Lasioglossum; subgenus: Sphecodogastra; specificEpithet: pauxillum; scientificNameAuthorship: (Schenck, 1853); **Location:** country: Italy; countryCode: IT; stateProvince: Roma; locality: Riserva Naturale Tenuta dei Massimi 1; decimalLatitude: 41.8532859; decimalLongitude: 12.3842322; geodeticDatum: WGS84; coordinatePrecision: 0.0002; **Identification:** identifiedBy: M. Mei; **Event:** eventDate: 2022-06-01; **Record Level:** collectionID: UR3
- **Type status:** Other material. **Occurrence:** catalogNumber: A1359, A1391; recordedBy: L. Fortini; individualCount: 2; sex: females; lifeStage: adult; occurrenceID: EE80777E-888B-5F2F-B2D6-2E7CDE807748; **Taxon:** scientificName: Lasioglossum (Sphecodogastra) pauxillum (Schenck, 1853); order: Hymenoptera; family: Halictidae; genus: Lasioglossum; subgenus: Sphecodogastra; specificEpithet: pauxillum; scientificNameAuthorship: (Schenck, 1853); **Location:** country: Italy; countryCode: IT; stateProvince: Roma; locality: Riserva Naturale Valle dell'Aniene 2; decimalLatitude: 41.928752; decimalLongitude: 12.5562962; geodeticDatum: WGS84; coordinatePrecision: 0.0002; **Identification:** identifiedBy: M. Mei; **Event:** eventDate: 2022-06-05; **Record Level:** collectionID: UR3
- **Type status:** Other material. **Occurrence:** catalogNumber: A1354; recordedBy: L. Fortini; individualCount: 1; sex: male; lifeStage: adult; occurrenceID: 43DD87E6-D134-57E1-8288-1EDE332BD2BD; **Taxon:** scientificName: Lasioglossum (Sphecodogastra) pauxillum (Schenck, 1853); order: Hymenoptera; family: Halictidae; genus: Lasioglossum; subgenus: Sphecodogastra; specificEpithet: pauxillum; scientificNameAuthorship: (Schenck, 1853); **Location:** country: Italy; countryCode: IT; stateProvince: Roma; locality: Riserva Naturale dell'Acquafredda; decimalLatitude: 41.8928408; decimalLongitude: 12.39932; geodeticDatum: WGS84; coordinatePrecision: 0.0002; **Identification:** identifiedBy: M. Mei; **Event:** eventDate: 2022-06-10; **Record Level:** collectionID: UR3
- **Type status:** Other material. **Occurrence:** catalogNumber: A1372; recordedBy: L. Fortini; individualCount: 1; sex: male; lifeStage: adult; occurrenceID: 36A97B0E-F80D-5BA2-AA2A-DA6194B0F27F; **Taxon:** scientificName: Lasioglossum (Sphecodogastra) pauxillum (Schenck, 1853); order: Hymenoptera; family: Halictidae; genus: Lasioglossum; subgenus: Sphecodogastra; specificEpithet: pauxillum; scientificNameAuthorship: (Schenck, 1853); **Location:** country: Italy; countryCode: IT; stateProvince: Roma; locality: Riserva Naturale Valle dei Casali 2; decimalLatitude: 41.8596887; decimalLongitude: 12.4355075; geodeticDatum: WGS84; coordinatePrecision: 0.0002; **Identification:** identifiedBy: M. Mei; **Event:** eventDate: 2022-06-18; **Record Level:** collectionID: UR3
- **Type status:** Other material. **Occurrence:** catalogNumber: A1412; recordedBy: L. Fortini; individualCount: 1; sex: female; lifeStage: adult; occurrenceID: 27AD91A8-1E4D-5819-ADED-70C43F757918; **Taxon:** scientificName: Lasioglossum (Sphecodogastra) pauxillum (Schenck, 1853); order: Hymenoptera; family: Halictidae; genus: Lasioglossum; subgenus: Sphecodogastra; specificEpithet: pauxillum; scientificNameAuthorship: (Schenck, 1853); **Location:** country: Italy; countryCode: IT; stateProvince: Roma; locality: Riserva Regionale dell'Appia Antica 1; decimalLatitude: 41.8623941; decimalLongitude: 12.524863; geodeticDatum: WGS84; coordinatePrecision: 0.0002; **Identification:** identifiedBy: M. Mei; **Event:** eventDate: 2022-06-12; **Record Level:** collectionID: UR3
- **Type status:** Other material. **Occurrence:** catalogNumber: A1440; recordedBy: L. Fortini; individualCount: 1; sex: female; lifeStage: adult; occurrenceID: 2AC8D886-9F16-573A-9057-6579517E3A6E; **Taxon:** scientificName: Lasioglossum (Sphecodogastra) pauxillum (Schenck, 1853); order: Hymenoptera; family: Halictidae; genus: Lasioglossum; subgenus: Sphecodogastra; specificEpithet: pauxillum; scientificNameAuthorship: (Schenck, 1853); **Location:** country: Italy; countryCode: IT; stateProvince: Roma; locality: Riserva Naturale di Monte Mario; decimalLatitude: 41.9386215; decimalLongitude: 12.4546223; geodeticDatum: WGS84; coordinatePrecision: 0.0002; **Identification:** identifiedBy: M. Mei; **Event:** eventDate: 2022-06-19; **Record Level:** collectionID: UR3
- **Type status:** Other material. **Occurrence:** catalogNumber: A1444, A1695; recordedBy: L. Fortini; individualCount: 2; sex: males; lifeStage: adult; occurrenceID: 57E63DDD-E5A9-5D64-A9A0-06A01B70BCD7; **Taxon:** scientificName: Lasioglossum (Sphecodogastra) pauxillum (Schenck, 1853); order: Hymenoptera; family: Halictidae; genus: Lasioglossum; subgenus: Sphecodogastra; specificEpithet: pauxillum; scientificNameAuthorship: (Schenck, 1853); **Location:** country: Italy; countryCode: IT; stateProvince: Roma; locality: Riserva Naturale dell'Insugherata 3; decimalLatitude: 41.9644829; decimalLongitude: 12.436101; geodeticDatum: WGS84; coordinatePrecision: 0.0002; **Identification:** identifiedBy: M. Mei; **Event:** eventDate: 2022-06-24; **Record Level:** collectionID: UR3
- **Type status:** Other material. **Occurrence:** catalogNumber: A1432; recordedBy: L. Fortini; individualCount: 1; sex: female; lifeStage: adult; occurrenceID: D6CF633E-35B9-5811-BE66-002AC62C6702; **Taxon:** scientificName: Lasioglossum (Sphecodogastra) pauxillum (Schenck, 1853); order: Hymenoptera; family: Halictidae; genus: Lasioglossum; subgenus: Sphecodogastra; specificEpithet: pauxillum; scientificNameAuthorship: (Schenck, 1853); **Location:** country: Italy; countryCode: IT; stateProvince: Roma; locality: Riserva Regionale dell'Appia Antica 2; decimalLatitude: 41.8402564; decimalLongitude: 12.532773; geodeticDatum: WGS84; coordinatePrecision: 0.0002; **Identification:** identifiedBy: M. Mei; **Event:** eventDate: 2022-07-07; **Record Level:** collectionID: UR3
- **Type status:** Other material. **Occurrence:** catalogNumber: A1448; recordedBy: L. Fortini; individualCount: 1; sex: female; lifeStage: adult; occurrenceID: 436350BD-23B5-57E3-84D0-EE819F5D0A9F; **Taxon:** scientificName: Lasioglossum (Sphecodogastra) pauxillum (Schenck, 1853); order: Hymenoptera; family: Halictidae; genus: Lasioglossum; subgenus: Sphecodogastra; specificEpithet: pauxillum; scientificNameAuthorship: (Schenck, 1853); **Location:** country: Italy; countryCode: IT; stateProvince: Roma; locality: Riserva Naturale Valle dell'Aniene 2; decimalLatitude: 41.928752; decimalLongitude: 12.5562962; geodeticDatum: WGS84; coordinatePrecision: 0.0002; **Identification:** identifiedBy: M. Mei; **Event:** eventDate: 2022-07-01; **Record Level:** collectionID: UR3
- **Type status:** Other material. **Occurrence:** catalogNumber: A1547; recordedBy: L. Fortini; individualCount: 1; sex: female; lifeStage: adult; occurrenceID: FCAD456C-2613-50A8-BECA-1D8D389F3384; **Taxon:** scientificName: Lasioglossum (Sphecodogastra) pauxillum (Schenck, 1853); order: Hymenoptera; family: Halictidae; genus: Lasioglossum; subgenus: Sphecodogastra; specificEpithet: pauxillum; scientificNameAuthorship: (Schenck, 1853); **Location:** country: Italy; countryCode: IT; stateProvince: Roma; locality: Riserva Regionale dell'Appia Antica 2; decimalLatitude: 41.8402564; decimalLongitude: 12.532773; geodeticDatum: WGS84; coordinatePrecision: 0.0002; **Identification:** identifiedBy: M. Mei; **Event:** eventDate: 2022-10-01; **Record Level:** collectionID: UR3

#### Lasioglossum perclavipes

(Blüthgen, 1934)

473F11C8-9611-5067-BDA4-63C706868B30

##### Materials

- **Type status:** Other material. **Occurrence:** catalogNumber: A1719; recordedBy: L. Fortini; individualCount: 1; sex: female; lifeStage: adult; occurrenceID: D41A013A-81C3-5A07-BB28-415FB2DB4865; **Taxon:** scientificName: Lasioglossum (Lasioglossum) perclavipes (Blüthgen, 1934); order: Hymenoptera; family: Halictidae; genus: Lasioglossum; subgenus: Lasioglossum; specificEpithet: perclavipes; scientificNameAuthorship: (Blüthgen, 1934); **Location:** country: Italy; countryCode: IT; stateProvince: Roma; locality: Riserva Regionale dell'Appia Antica 1; decimalLatitude: 41.8623941; decimalLongitude: 12.524863; geodeticDatum: WGS84; coordinatePrecision: 0.0002; **Identification:** identifiedBy: M. Mei; **Event:** eventDate: 2022-05-10; **Record Level:** collectionID: UR3

#### Lasioglossum politum

(Schenck, 1853)

94FDE8D6-B607-52AA-BC33-E04DFA0FDA19

##### Materials

- **Type status:** Other material. **Occurrence:** catalogNumber: A1454; recordedBy: L. Fortini; individualCount: 1; sex: female; lifeStage: adult; occurrenceID: B278341A-9320-55D9-BCE3-D5937379965D; **Taxon:** scientificName: Lasioglossum (Pyghalictus) politum (Schenck, 1853); order: Hymenoptera; family: Halictidae; genus: Lasioglossum; subgenus: Pyghalictus; specificEpithet: politum; scientificNameAuthorship: (Schenck, 1853); **Location:** country: Italy; countryCode: IT; stateProvince: Roma; locality: Riserva Naturale dell'Acquafredda; decimalLatitude: 41.8928408; decimalLongitude: 12.39932; geodeticDatum: WGS84; coordinatePrecision: 0.0002; **Identification:** identifiedBy: M. Mei; **Event:** eventDate: 2022-07-12; **Record Level:** collectionID: UR3
- **Type status:** Other material. **Occurrence:** catalogNumber: A1517, A1518, A1520, A1521, A1523, A1531, A1535, A1536; recordedBy: L. Fortini; individualCount: 8; sex: females; lifeStage: adult; occurrenceID: DE52E09B-EA94-580C-8AF8-66756D0F3F41; **Taxon:** scientificName: Lasioglossum (Pyghalictus) politum (Schenck, 1853); order: Hymenoptera; family: Halictidae; genus: Lasioglossum; subgenus: Pyghalictus; specificEpithet: politum; scientificNameAuthorship: (Schenck, 1853); **Location:** country: Italy; countryCode: IT; stateProvince: Roma; locality: Riserva Naturale dell'Acquafredda; decimalLatitude: 41.8928408; decimalLongitude: 12.39932; geodeticDatum: WGS84; coordinatePrecision: 0.0002; **Identification:** identifiedBy: M. Mei; **Event:** eventDate: 2022-08-17; **Record Level:** collectionID: UR3
- **Type status:** Other material. **Occurrence:** catalogNumber: A1500, A1502, A1505, A1509, A1510, A1511, A1512, A1513, A1514; recordedBy: L. Fortini; individualCount: 9; sex: females; lifeStage: adult; occurrenceID: CAB83942-FE26-5ED4-BC3F-9CF3C62DA6F4; **Taxon:** scientificName: Lasioglossum (Pyghalictus) politum (Schenck, 1853); order: Hymenoptera; family: Halictidae; genus: Lasioglossum; subgenus: Pyghalictus; specificEpithet: politum; scientificNameAuthorship: (Schenck, 1853); **Location:** country: Italy; countryCode: IT; stateProvince: Roma; locality: Riserva Regionale dell'Appia Antica 1; decimalLatitude: 41.8623941; decimalLongitude: 12.524863; geodeticDatum: WGS84; coordinatePrecision: 0.0002; **Identification:** identifiedBy: M. Mei; **Event:** eventDate: 2022-07-02; **Record Level:** collectionID: UR3
- **Type status:** Other material. **Occurrence:** catalogNumber: A1431, A1451, A1452, A1459, A1462, A1464, A1467; recordedBy: L. Fortini; individualCount: 7; sex: females; lifeStage: adult; occurrenceID: 05059CA6-3C5C-582D-B729-5AEE4C0AE53B; **Taxon:** scientificName: Lasioglossum (Pyghalictus) politum (Schenck, 1853); order: Hymenoptera; family: Halictidae; genus: Lasioglossum; subgenus: Pyghalictus; specificEpithet: politum; scientificNameAuthorship: (Schenck, 1853); **Location:** country: Italy; countryCode: IT; stateProvince: Roma; locality: Riserva Regionale dell'Appia Antica 2; decimalLatitude: 41.8402564; decimalLongitude: 12.532773; geodeticDatum: WGS84; coordinatePrecision: 0.0002; **Identification:** identifiedBy: M. Mei; **Event:** eventDate: 2022-07-07; **Record Level:** collectionID: UR3
- **Type status:** Other material. **Occurrence:** catalogNumber: A1540, A1543, A1549; recordedBy: L. Fortini; individualCount: 3; sex: females; lifeStage: adult; occurrenceID: D82F896F-F602-54C1-BB5A-B0D39FC4DE2F; **Taxon:** scientificName: Lasioglossum (Pyghalictus) politum (Schenck, 1853); order: Hymenoptera; family: Halictidae; genus: Lasioglossum; subgenus: Pyghalictus; specificEpithet: politum; scientificNameAuthorship: (Schenck, 1853); **Location:** country: Italy; countryCode: IT; stateProvince: Roma; locality: Riserva Regionale dell'Appia Antica 2; decimalLatitude: 41.8402564; decimalLongitude: 12.532773; geodeticDatum: WGS84; coordinatePrecision: 0.0002; **Identification:** identifiedBy: M. Mei; **Event:** eventDate: 2022-08-06; **Record Level:** collectionID: UR3
- **Type status:** Other material. **Occurrence:** catalogNumber: A1545, A1552, A1553; recordedBy: L. Fortini; individualCount: 3; sex: females; lifeStage: adult; occurrenceID: 73624D89-608E-5BAA-9249-4E4EF3E56CC4; **Taxon:** scientificName: Lasioglossum (Pyghalictus) politum (Schenck, 1853); order: Hymenoptera; family: Halictidae; genus: Lasioglossum; subgenus: Pyghalictus; specificEpithet: politum; scientificNameAuthorship: (Schenck, 1853); **Location:** country: Italy; countryCode: IT; stateProvince: Roma; locality: Riserva Regionale dell'Appia Antica 2; decimalLatitude: 41.8402564; decimalLongitude: 12.532773; geodeticDatum: WGS84; coordinatePrecision: 0.0002; **Identification:** identifiedBy: M. Mei; **Event:** eventDate: 2022-08-29; **Record Level:** collectionID: UR3
- **Type status:** Other material. **Occurrence:** catalogNumber: A1443; recordedBy: L. Fortini; individualCount: 1; sex: male; lifeStage: adult; occurrenceID: 9601241B-96F3-534A-9293-BB348435B7D5; **Taxon:** scientificName: Lasioglossum (Pyghalictus) politum (Schenck, 1853); order: Hymenoptera; family: Halictidae; genus: Lasioglossum; subgenus: Pyghalictus; specificEpithet: politum; scientificNameAuthorship: (Schenck, 1853); **Location:** country: Italy; countryCode: IT; stateProvince: Roma; locality: Riserva Naturale dell'Insugherata 3; decimalLatitude: 41.9644829; decimalLongitude: 12.436101; geodeticDatum: WGS84; coordinatePrecision: 0.0002; **Identification:** identifiedBy: M. Mei; **Event:** eventDate: 2022-06-24; **Record Level:** collectionID: UR3
- **Type status:** Other material. **Occurrence:** catalogNumber: A1499, A1501, A1503, A1506, A1507, A1508; recordedBy: L. Fortini; individualCount: 6; sex: females; lifeStage: adult; occurrenceID: E91AFD58-837C-5D09-AC96-F8E9F95C2F0F; **Taxon:** scientificName: Lasioglossum (Pyghalictus) politum (Schenck, 1853); order: Hymenoptera; family: Halictidae; genus: Lasioglossum; subgenus: Pyghalictus; specificEpithet: politum; scientificNameAuthorship: (Schenck, 1853); **Location:** country: Italy; countryCode: IT; stateProvince: Roma; locality: Riserva Naturale dell'Insugherata 3; decimalLatitude: 41.9644829; decimalLongitude: 12.436101; geodeticDatum: WGS84; coordinatePrecision: 0.0002; **Identification:** identifiedBy: M. Mei; **Event:** eventDate: 2022-07-30; **Record Level:** collectionID: UR3
- **Type status:** Other material. **Occurrence:** catalogNumber: A1532, A1534; recordedBy: L. Fortini; individualCount: 2; sex: females; lifeStage: adult; occurrenceID: FDEE7D76-8A4E-504A-BF1F-61B2B2F893EF; **Taxon:** scientificName: Lasioglossum (Pyghalictus) politum (Schenck, 1853); order: Hymenoptera; family: Halictidae; genus: Lasioglossum; subgenus: Pyghalictus; specificEpithet: politum; scientificNameAuthorship: (Schenck, 1853); **Location:** country: Italy; countryCode: IT; stateProvince: Roma; locality: Riserva Naturale Laurentino-Acqua Acetosa; decimalLatitude: 41.8079275; decimalLongitude: 12.4685548; geodeticDatum: WGS84; coordinatePrecision: 0.0002; **Identification:** identifiedBy: M. Mei; **Event:** eventDate: 2022-07-21; **Record Level:** collectionID: UR3
- **Type status:** Other material. **Occurrence:** catalogNumber: A1442; recordedBy: L. Fortini; individualCount: 1; sex: male; lifeStage: adult; occurrenceID: 6EDE982C-32FB-5F96-8FBC-EB86DDE3BEB0; **Taxon:** scientificName: Lasioglossum (Pyghalictus) politum (Schenck, 1853); order: Hymenoptera; family: Halictidae; genus: Lasioglossum; subgenus: Pyghalictus; specificEpithet: politum; scientificNameAuthorship: (Schenck, 1853); **Location:** country: Italy; countryCode: IT; stateProvince: Roma; locality: Riserva Naturale Valle dell'Aniene 1; decimalLatitude: 41.9345179; decimalLongitude: 12.5453096; geodeticDatum: WGS84; coordinatePrecision: 0.0002; **Identification:** identifiedBy: M. Mei; **Event:** eventDate: 2022-07-01; **Record Level:** collectionID: UR3
- **Type status:** Other material. **Occurrence:** catalogNumber: A1493, A1494, A1496; recordedBy: L. Fortini; individualCount: 3; sex: females; lifeStage: adult; occurrenceID: 7277A0E7-18B6-59D6-9C25-34102BCBF5C6; **Taxon:** scientificName: Lasioglossum (Pyghalictus) politum (Schenck, 1853); order: Hymenoptera; family: Halictidae; genus: Lasioglossum; subgenus: Pyghalictus; specificEpithet: politum; scientificNameAuthorship: (Schenck, 1853); **Location:** country: Italy; countryCode: IT; stateProvince: Roma; locality: Riserva Naturale Valle dell'Aniene 1; decimalLatitude: 41.9345179; decimalLongitude: 12.5453096; geodeticDatum: WGS84; coordinatePrecision: 0.0002; **Identification:** identifiedBy: M. Mei; **Event:** eventDate: 2022-09-04; **Record Level:** collectionID: UR3
- **Type status:** Other material. **Occurrence:** catalogNumber: A2031, A2032, A2043, A2044; recordedBy: L. Fortini; individualCount: 4; sex: females; lifeStage: adult; occurrenceID: 2643722A-D442-5F01-BD09-EFDEA52C062F; **Taxon:** scientificName: Lasioglossum (Pyghalictus) politum (Schenck, 1853); order: Hymenoptera; family: Halictidae; genus: Lasioglossum; subgenus: Pyghalictus; specificEpithet: politum; scientificNameAuthorship: (Schenck, 1853); **Location:** country: Italy; countryCode: IT; stateProvince: Roma; locality: Riserva Naturale Valle dei Casali 1; decimalLatitude: 41.8710627; decimalLongitude: 12.4336809; geodeticDatum: WGS84; coordinatePrecision: 0.0002; **Identification:** identifiedBy: M. Mei; **Event:** eventDate: 2022-08-19; **Record Level:** collectionID: UR3
- **Type status:** Other material. **Occurrence:** catalogNumber: A2052, A2053, A2058, A2059; recordedBy: L. Fortini; individualCount: 4; sex: females; lifeStage: adult; occurrenceID: 0714EA4B-2B9B-5D55-9396-3866652644A0; **Taxon:** scientificName: Lasioglossum (Pyghalictus) politum (Schenck, 1853); order: Hymenoptera; family: Halictidae; genus: Lasioglossum; subgenus: Pyghalictus; specificEpithet: politum; scientificNameAuthorship: (Schenck, 1853); **Location:** country: Italy; countryCode: IT; stateProvince: Roma; locality: Riserva Naturale dell'Insugherata 3; decimalLatitude: 41.9644829; decimalLongitude: 12.436101; geodeticDatum: WGS84; coordinatePrecision: 0.0002; **Identification:** identifiedBy: M. Mei; **Event:** eventDate: 2022-07-30; **Record Level:** collectionID: UR3
- **Type status:** Other material. **Occurrence:** catalogNumber: A2061, A2062, A2065, A2066; recordedBy: L. Fortini; individualCount: 4; sex: females; lifeStage: adult; occurrenceID: 82823CB4-A7AA-5139-9FC2-1210EF1A1F4D; **Taxon:** scientificName: Lasioglossum (Pyghalictus) politum (Schenck, 1853); order: Hymenoptera; family: Halictidae; genus: Lasioglossum; subgenus: Pyghalictus; specificEpithet: politum; scientificNameAuthorship: (Schenck, 1853); **Location:** country: Italy; countryCode: IT; stateProvince: Roma; locality: Riserva Naturale di Monte Mario; decimalLatitude: 41.9386215; decimalLongitude: 12.4546223; geodeticDatum: WGS84; coordinatePrecision: 0.0002; **Identification:** identifiedBy: M. Mei; **Event:** eventDate: 2022-07-24; **Record Level:** collectionID: UR3
- **Type status:** Other material. **Occurrence:** catalogNumber: A2111, A2112, A2117, A2118, A2129, A2130; recordedBy: L. Fortini; individualCount: 6; sex: females; lifeStage: adult; occurrenceID: EE673C63-9667-5CA2-AC08-D34042A541A5; **Taxon:** scientificName: Lasioglossum (Pyghalictus) politum (Schenck, 1853); order: Hymenoptera; family: Halictidae; genus: Lasioglossum; subgenus: Pyghalictus; specificEpithet: politum; scientificNameAuthorship: (Schenck, 1853); **Location:** country: Italy; countryCode: IT; stateProvince: Roma; locality: Riserva Naturale Valle dell'Aniene 2; decimalLatitude: 41.928752; decimalLongitude: 12.5562962; geodeticDatum: WGS84; coordinatePrecision: 0.0002; **Identification:** identifiedBy: M. Mei; **Event:** eventDate: 2022-08-03; **Record Level:** collectionID: UR3

#### Lasioglossum pygmaeum

(Schenck, 1853)

677FA6A1-8A93-54A8-A117-176B7D815F67

##### Materials

- **Type status:** Other material. **Occurrence:** catalogNumber: A0459; recordedBy: L. Fortini; individualCount: 1; sex: male; lifeStage: adult; occurrenceID: 5FD4403C-537F-51C8-85CD-05EAD828A4E8; **Taxon:** scientificName: Lasioglossum (Hemihalictus) pygmaeum (Schenck, 1853); order: Hymenoptera; family: Halictidae; genus: Lasioglossum; subgenus: Hemihalictus; specificEpithet: pygmaeum; scientificNameAuthorship: (Schenck, 1853); **Location:** country: Italy; countryCode: IT; stateProvince: Roma; locality: Riserva Naturale di Monte Mario; decimalLatitude: 41.9386215; decimalLongitude: 12.4546223; geodeticDatum: WGS84; coordinatePrecision: 0.0002; **Identification:** identifiedBy: M. Mei; **Event:** eventDate: 2022-06-19; **Record Level:** collectionID: UR3
- **Type status:** Other material. **Occurrence:** catalogNumber: A1463; recordedBy: L. Fortini; individualCount: 1; sex: female; lifeStage: adult; occurrenceID: 0F0221AF-6146-55C7-9334-00A8037B8F38; **Taxon:** scientificName: Lasioglossum (Hemihalictus) pygmaeum (Schenck, 1853); order: Hymenoptera; family: Halictidae; genus: Lasioglossum; subgenus: Hemihalictus; specificEpithet: pygmaeum; scientificNameAuthorship: (Schenck, 1853); **Location:** country: Italy; countryCode: IT; stateProvince: Roma; locality: Riserva Regionale dell'Appia Antica 2; decimalLatitude: 41.8402564; decimalLongitude: 12.532773; geodeticDatum: WGS84; coordinatePrecision: 0.0002; **Identification:** identifiedBy: M. Mei; **Event:** eventDate: 2022-07-07; **Record Level:** collectionID: UR3

#### Lasioglossum punctatissimum

(Schenck, 1853)

2C21CA2A-AE66-58C7-917D-3F70244C02AF

##### Materials

- **Type status:** Other material. **Occurrence:** catalogNumber: A1337; recordedBy: L. Fortini; individualCount: 1; sex: female; lifeStage: adult; occurrenceID: C972402A-345C-5721-A2D4-E024EAF87A2E; **Taxon:** scientificName: Lasioglossum (Hemihalictus) punctatissimum (Schenck, 1853); order: Hymenoptera; family: Halictidae; genus: Lasioglossum; subgenus: Hemihalictus; specificEpithet: punctatissimum; scientificNameAuthorship: (Schenck, 1853); **Location:** country: Italy; countryCode: IT; stateProvince: Roma; locality: Riserva Naturale Valle dei Casali 1; decimalLatitude: 41.8710627; decimalLongitude: 12.4336809; geodeticDatum: WGS84; coordinatePrecision: 0.0002; **Identification:** identifiedBy: M. Mei; **Event:** eventDate: 2022-05-14; **Record Level:** collectionID: UR3
- **Type status:** Other material. **Occurrence:** catalogNumber: A1344; recordedBy: L. Fortini; individualCount: 1; sex: female; lifeStage: adult; occurrenceID: 4DC61E34-C500-5911-A146-F91A02BD8A9E; **Taxon:** scientificName: Lasioglossum (Hemihalictus) punctatissimum (Schenck, 1853); order: Hymenoptera; family: Halictidae; genus: Lasioglossum; subgenus: Hemihalictus; specificEpithet: punctatissimum; scientificNameAuthorship: (Schenck, 1853); **Location:** country: Italy; countryCode: IT; stateProvince: Roma; locality: Riserva Naturale dell'Insugherata 3; decimalLatitude: 41.9644829; decimalLongitude: 12.436101; geodeticDatum: WGS84; coordinatePrecision: 0.0002; **Identification:** identifiedBy: M. Mei; **Event:** eventDate: 2022-04-15; **Record Level:** collectionID: UR3
- **Type status:** Other material. **Occurrence:** catalogNumber: A1468; recordedBy: L. Fortini; individualCount: 1; sex: female; lifeStage: adult; occurrenceID: 53BA6BF2-E9AE-54C5-9BC1-CC626D8FB7EC; **Taxon:** scientificName: Lasioglossum (Hemihalictus) punctatissimum (Schenck, 1853); order: Hymenoptera; family: Halictidae; genus: Lasioglossum; subgenus: Hemihalictus; specificEpithet: punctatissimum; scientificNameAuthorship: (Schenck, 1853); **Location:** country: Italy; countryCode: IT; stateProvince: Roma; locality: Riserva Regionale dell'Appia Antica 2; decimalLatitude: 41.8402564; decimalLongitude: 12.532773; geodeticDatum: WGS84; coordinatePrecision: 0.0002; **Identification:** identifiedBy: M. Mei; **Event:** eventDate: 2022-07-07; **Record Level:** collectionID: UR3
- **Type status:** Other material. **Occurrence:** catalogNumber: A1528; recordedBy: L. Fortini; individualCount: 1; sex: female; lifeStage: adult; occurrenceID: FB8D9CBF-481E-5BD2-85F1-8F630DDBBCC1; **Taxon:** scientificName: Lasioglossum (Hemihalictus) punctatissimum (Schenck, 1853); order: Hymenoptera; family: Halictidae; genus: Lasioglossum; subgenus: Hemihalictus; specificEpithet: punctatissimum; scientificNameAuthorship: (Schenck, 1853); **Location:** country: Italy; countryCode: IT; stateProvince: Roma; locality: Riserva Naturale dell'Insugherata 3; decimalLatitude: 41.9644829; decimalLongitude: 12.436101; geodeticDatum: WGS84; coordinatePrecision: 0.0002; **Identification:** identifiedBy: M. Mei; **Event:** eventDate: 2022-05-27; **Record Level:** collectionID: UR3
- **Type status:** Other material. **Occurrence:** catalogNumber: A1541; recordedBy: L. Fortini; individualCount: 1; sex: female; lifeStage: adult; occurrenceID: 16C5230A-B86C-537F-8AFF-D4A2CEF7CBDE; **Taxon:** scientificName: Lasioglossum (Hemihalictus) punctatissimum (Schenck, 1853); order: Hymenoptera; family: Halictidae; genus: Lasioglossum; subgenus: Hemihalictus; specificEpithet: punctatissimum; scientificNameAuthorship: (Schenck, 1853); **Location:** country: Italy; countryCode: IT; stateProvince: Roma; locality: Riserva Naturale Laurentino-Acqua Acetosa; decimalLatitude: 41.8079275; decimalLongitude: 12.4685548; geodeticDatum: WGS84; coordinatePrecision: 0.0002; **Identification:** identifiedBy: M. Mei; **Event:** eventDate: 2022-08-17; **Record Level:** collectionID: UR3

#### Lasioglossum puncticolle

(Morawitz, 1872)

7B5AC90A-A6A0-507A-B7B4-DB72C25362A3

##### Materials

- **Type status:** Other material. **Occurrence:** catalogNumber: A1320, A1331; recordedBy: L. Fortini; individualCount: 2; sex: females; lifeStage: adult; occurrenceID: 01ADB93D-66C1-5E72-9D92-A51113D9905E; **Taxon:** scientificName: Lasioglossum (Hemihalictus) puncticolle (Morawitz, 1872); order: Hymenoptera; family: Halictidae; genus: Lasioglossum; subgenus: Hemihalictus; specificEpithet: puncticolle; scientificNameAuthorship: (Morawitz, 1872); **Location:** country: Italy; countryCode: IT; stateProvince: Roma; locality: Riserva Naturale Tenuta dei Massimi 2; decimalLatitude: 41.8316516; decimalLongitude: 12.3999927; geodeticDatum: WGS84; coordinatePrecision: 0.0002; **Identification:** identifiedBy: M. Mei; **Event:** eventDate: 2022-05-04; **Record Level:** collectionID: UR3
- **Type status:** Other material. **Occurrence:** catalogNumber: A1380, A1382, A1390, A1393, A1395, A1396; recordedBy: L. Fortini; individualCount: 6; sex: females; lifeStage: adult; occurrenceID: 632CB6DF-2EF0-518D-A228-D62EEEF0CCF3; **Taxon:** scientificName: Lasioglossum (Hemihalictus) puncticolle (Morawitz, 1872); order: Hymenoptera; family: Halictidae; genus: Lasioglossum; subgenus: Hemihalictus; specificEpithet: puncticolle; scientificNameAuthorship: (Morawitz, 1872); **Location:** country: Italy; countryCode: IT; stateProvince: Roma; locality: Riserva Naturale dell'Insugherata 3; decimalLatitude: 41.9644829; decimalLongitude: 12.436101; geodeticDatum: WGS84; coordinatePrecision: 0.0002; **Identification:** identifiedBy: M. Mei; **Event:** eventDate: 2022-05-27; **Record Level:** collectionID: UR3
- **Type status:** Other material. **Occurrence:** catalogNumber: A1379, A1381, A1387, A1397, A1399, A1403, A1404, A1405, A1529; recordedBy: L. Fortini; individualCount: 9; sex: females; lifeStage: adult; occurrenceID: 81AF19BD-727C-5B81-BA80-8A7253078AEC; **Taxon:** scientificName: Lasioglossum (Hemihalictus) puncticolle (Morawitz, 1872); order: Hymenoptera; family: Halictidae; genus: Lasioglossum; subgenus: Hemihalictus; specificEpithet: puncticolle; scientificNameAuthorship: (Morawitz, 1872); **Location:** country: Italy; countryCode: IT; stateProvince: Roma; locality: Riserva Naturale Tenuta dei Massimi 1; decimalLatitude: 41.8532859; decimalLongitude: 12.3842322; geodeticDatum: WGS84; coordinatePrecision: 0.0002; **Identification:** identifiedBy: M. Mei; **Event:** eventDate: 2022-06-01; **Record Level:** collectionID: UR3
- **Type status:** Other material. **Occurrence:** catalogNumber: A1436, A1441, A1446; recordedBy: L. Fortini; individualCount: 3; sex: females; lifeStage: adult; occurrenceID: 7F764347-17AD-5224-A201-502C865521CA; **Taxon:** scientificName: Lasioglossum (Hemihalictus) puncticolle (Morawitz, 1872); order: Hymenoptera; family: Halictidae; genus: Lasioglossum; subgenus: Hemihalictus; specificEpithet: puncticolle; scientificNameAuthorship: (Morawitz, 1872); **Location:** country: Italy; countryCode: IT; stateProvince: Roma; locality: Riserva Naturale Tenuta dei Massimi 1; decimalLatitude: 41.8532859; decimalLongitude: 12.3842322; geodeticDatum: WGS84; coordinatePrecision: 0.0002; **Identification:** identifiedBy: M. Mei; **Event:** eventDate: 2022-06-27; **Record Level:** collectionID: UR3
- **Type status:** Other material. **Occurrence:** catalogNumber: A1434; recordedBy: L. Fortini; individualCount: 1; sex: female; lifeStage: adult; occurrenceID: E8A5B9BF-388B-5BCE-BCDA-DC97751571EB; **Taxon:** scientificName: Lasioglossum (Hemihalictus) puncticolle (Morawitz, 1872); order: Hymenoptera; family: Halictidae; genus: Lasioglossum; subgenus: Hemihalictus; specificEpithet: puncticolle; scientificNameAuthorship: (Morawitz, 1872); **Location:** country: Italy; countryCode: IT; stateProvince: Roma; locality: Riserva Naturale Valle dell'Aniene 2; decimalLatitude: 41.928752; decimalLongitude: 12.5562962; geodeticDatum: WGS84; coordinatePrecision: 0.0002; **Identification:** identifiedBy: M. Mei; **Event:** eventDate: 2022-07-01; **Record Level:** collectionID: UR3
- **Type status:** Other material. **Occurrence:** catalogNumber: A1627; recordedBy: L. Fortini; individualCount: 1; sex: male; lifeStage: adult; occurrenceID: 97F6297D-D988-5E57-A2BD-09662E47CD83; **Taxon:** scientificName: Lasioglossum (Hemihalictus) puncticolle (Morawitz, 1872); order: Hymenoptera; family: Halictidae; genus: Lasioglossum; subgenus: Hemihalictus; specificEpithet: puncticolle; scientificNameAuthorship: (Morawitz, 1872); **Location:** country: Italy; countryCode: IT; stateProvince: Roma; locality: Riserva Naturale Valle dei Casali 2; decimalLatitude: 41.8596887; decimalLongitude: 12.4355075; geodeticDatum: WGS84; coordinatePrecision: 0.0002; **Identification:** identifiedBy: M. Mei; **Event:** eventDate: 2022-07-13; **Record Level:** collectionID: UR3
- **Type status:** Other material. **Occurrence:** catalogNumber: A2071, A2072; recordedBy: L. Fortini; individualCount: 2; sex: females; lifeStage: adult; occurrenceID: 5844C2D5-02CB-5F1A-A7CF-82ACD2148448; **Taxon:** scientificName: Lasioglossum (Hemihalictus) puncticolle (Morawitz, 1872); order: Hymenoptera; family: Halictidae; genus: Lasioglossum; subgenus: Hemihalictus; specificEpithet: puncticolle; scientificNameAuthorship: (Morawitz, 1872); **Location:** country: Italy; countryCode: IT; stateProvince: Roma; locality: Riserva Naturale di Monte Mario; decimalLatitude: 41.9386215; decimalLongitude: 12.4546223; geodeticDatum: WGS84; coordinatePrecision: 0.0002; **Identification:** identifiedBy: M. Mei; **Event:** eventDate: 2022-07-24; **Record Level:** collectionID: UR3
- **Type status:** Other material. **Occurrence:** catalogNumber: A1474, A1475, A1504; recordedBy: L. Fortini; individualCount: 3; sex: females; lifeStage: adult; occurrenceID: 2404E72F-07E6-50CC-B6C9-814D08E8A608; **Taxon:** scientificName: Lasioglossum (Hemihalictus) puncticolle (Morawitz, 1872); order: Hymenoptera; family: Halictidae; genus: Lasioglossum; subgenus: Hemihalictus; specificEpithet: puncticolle; scientificNameAuthorship: (Morawitz, 1872); **Location:** country: Italy; countryCode: IT; stateProvince: Roma; locality: Riserva Naturale Tenuta dei Massimi 1; decimalLatitude: 41.8532859; decimalLongitude: 12.3842322; geodeticDatum: WGS84; coordinatePrecision: 0.0002; **Identification:** identifiedBy: M. Mei; **Event:** eventDate: 2022-07-28; **Record Level:** collectionID: UR3
- **Type status:** Other material. **Occurrence:** catalogNumber: A2121; recordedBy: L. Fortini; individualCount: 1; sex: female; lifeStage: adult; occurrenceID: FBD523BD-A358-5848-8B14-85231F98DACC; **Taxon:** scientificName: Lasioglossum (Hemihalictus) puncticolle (Morawitz, 1872); order: Hymenoptera; family: Halictidae; genus: Lasioglossum; subgenus: Hemihalictus; specificEpithet: puncticolle; scientificNameAuthorship: (Morawitz, 1872); **Location:** country: Italy; countryCode: IT; stateProvince: Roma; locality: Riserva Naturale Valle dell'Aniene 2; decimalLatitude: 41.928752; decimalLongitude: 12.5562962; geodeticDatum: WGS84; coordinatePrecision: 0.0002; **Identification:** identifiedBy: M. Mei; **Event:** eventDate: 2022-08-03; **Record Level:** collectionID: UR3
- **Type status:** Other material. **Occurrence:** catalogNumber: A1554, A1555, A1556; recordedBy: L. Fortini; individualCount: 3; sex: females; lifeStage: adult; occurrenceID: 82E33B82-1BA2-5AC9-A661-9ADD4B4C5DCC; **Taxon:** scientificName: Lasioglossum (Hemihalictus) puncticolle (Morawitz, 1872); order: Hymenoptera; family: Halictidae; genus: Lasioglossum; subgenus: Hemihalictus; specificEpithet: puncticolle; scientificNameAuthorship: (Morawitz, 1872); **Location:** country: Italy; countryCode: IT; stateProvince: Roma; locality: Riserva Naturale Tenuta dei Massimi 1; decimalLatitude: 41.8532859; decimalLongitude: 12.3842322; geodeticDatum: WGS84; coordinatePrecision: 0.0002; **Identification:** identifiedBy: M. Mei; **Event:** eventDate: 2022-08-27; **Record Level:** collectionID: UR3

#### Lasioglossum transitorium

(Schenck, 1868)

D548C5D9-FAAF-5F22-98D5-E83456980DBF

##### Materials

- **Type status:** Other material. **Occurrence:** catalogNumber: A1305; recordedBy: L. Fortini; individualCount: 1; sex: female; lifeStage: adult; occurrenceID: A2006EE1-8C8A-57C8-928C-B35E46CCD4ED; **Taxon:** scientificName: Lasioglossum (Hemihalictus) transitorium (Schenck, 1868); order: Hymenoptera; family: Halictidae; genus: Lasioglossum; subgenus: Hemihalictus; specificEpithet: transitorium; scientificNameAuthorship: (Schenck, 1868); **Location:** country: Italy; countryCode: IT; stateProvince: Roma; locality: Riserva Naturale Valle dei Casali 2; decimalLatitude: 41.8596887; decimalLongitude: 12.4355075; geodeticDatum: WGS84; coordinatePrecision: 0.0002; **Identification:** identifiedBy: M. Mei; **Event:** eventDate: 2022-04-10; **Record Level:** collectionID: UR3
- **Type status:** Other material. **Occurrence:** catalogNumber: A1280, A1284; recordedBy: L. Fortini; individualCount: 2; sex: females; lifeStage: adult; occurrenceID: 9E784F91-8FBB-588A-BB6F-EC99108877BC; **Taxon:** scientificName: Lasioglossum (Hemihalictus) transitorium (Schenck, 1868); order: Hymenoptera; family: Halictidae; genus: Lasioglossum; subgenus: Hemihalictus; specificEpithet: transitorium; scientificNameAuthorship: (Schenck, 1868); **Location:** country: Italy; countryCode: IT; stateProvince: Roma; locality: Riserva Naturale dell'Insugherata 2; decimalLatitude: 41.9599247; decimalLongitude: 12.433852; geodeticDatum: WGS84; coordinatePrecision: 0.0002; **Identification:** identifiedBy: M. Mei; **Event:** eventDate: 2022-04-15; **Record Level:** collectionID: UR3
- **Type status:** Other material. **Occurrence:** catalogNumber: A1294, A1312; recordedBy: L. Fortini; individualCount: 2; sex: females; lifeStage: adult; occurrenceID: DAF76DBB-891F-5244-9036-7980771668AB; **Taxon:** scientificName: Lasioglossum (Hemihalictus) transitorium (Schenck, 1868); order: Hymenoptera; family: Halictidae; genus: Lasioglossum; subgenus: Hemihalictus; specificEpithet: transitorium; scientificNameAuthorship: (Schenck, 1868); **Location:** country: Italy; countryCode: IT; stateProvince: Roma; locality: Riserva Naturale Tenuta dei Massimi 1; decimalLatitude: 41.8532859; decimalLongitude: 12.3842322; geodeticDatum: WGS84; coordinatePrecision: 0.0002; **Identification:** identifiedBy: M. Mei; **Event:** eventDate: 2022-04-23; **Record Level:** collectionID: UR3
- **Type status:** Other material. **Occurrence:** catalogNumber: A1255; recordedBy: L. Fortini; individualCount: 1; sex: female; lifeStage: adult; occurrenceID: 52DC1462-FF4D-57AC-8494-1EE9F7650532; **Taxon:** scientificName: Lasioglossum (Hemihalictus) transitorium (Schenck, 1868); order: Hymenoptera; family: Halictidae; genus: Lasioglossum; subgenus: Hemihalictus; specificEpithet: transitorium; scientificNameAuthorship: (Schenck, 1868); **Location:** country: Italy; countryCode: IT; stateProvince: Roma; locality: Riserva Naturale dell'Insugherata 2; decimalLatitude: 41.9599247; decimalLongitude: 12.433852; geodeticDatum: WGS84; coordinatePrecision: 0.0002; **Identification:** identifiedBy: M. Mei; **Event:** eventDate: 2022-04-27; **Record Level:** collectionID: UR3
- **Type status:** Other material. **Occurrence:** catalogNumber: A1355; recordedBy: L. Fortini; individualCount: 1; sex: female; lifeStage: adult; occurrenceID: 258CD726-B376-5381-A039-1B17DCB18D12; **Taxon:** scientificName: Lasioglossum (Hemihalictus) transitorium (Schenck, 1868); order: Hymenoptera; family: Halictidae; genus: Lasioglossum; subgenus: Hemihalictus; specificEpithet: transitorium; scientificNameAuthorship: (Schenck, 1868); **Location:** country: Italy; countryCode: IT; stateProvince: Roma; locality: Riserva Naturale Valle dell'Aniene 2; decimalLatitude: 41.928752; decimalLongitude: 12.5562962; geodeticDatum: WGS84; coordinatePrecision: 0.0002; **Identification:** identifiedBy: M. Mei; **Event:** eventDate: 2022-06-05; **Record Level:** collectionID: UR3

#### Lasioglossum truncaticolle

(Morawitz, 1877)

A5FE2997-C162-52B4-A1D2-2554A4758132

##### Materials

- **Type status:** Other material. **Occurrence:** catalogNumber: A1272, A1388; recordedBy: L. Fortini; individualCount: 2; sex: females; lifeStage: adult; occurrenceID: A774AD07-792F-5652-8DC4-E519FCF12A3E; **Taxon:** scientificName: Lasioglossum (Hemihalictus) truncaticolle (Morawitz, 1877); order: Hymenoptera; family: Halictidae; genus: Lasioglossum; subgenus: Hemihalictus; specificEpithet: truncaticolle; scientificNameAuthorship: (Morawitz, 1877); **Location:** country: Italy; countryCode: IT; stateProvince: Roma; locality: Riserva Naturale dell'Insugherata 1; decimalLatitude: 41.9555045; decimalLongitude: 12.4292321; geodeticDatum: WGS84; coordinatePrecision: 0.0002; **Identification:** identifiedBy: M. Mei; **Event:** eventDate: 2022-05-27; **Record Level:** collectionID: UR3
- **Type status:** Other material. **Occurrence:** catalogNumber: A1621; recordedBy: L. Fortini; individualCount: 1; sex: male; lifeStage: adult; occurrenceID: 4610195C-6654-53AC-8958-4EF400C911DA; **Taxon:** scientificName: Lasioglossum (Hemihalictus) truncaticolle (Morawitz, 1877); order: Hymenoptera; family: Halictidae; genus: Lasioglossum; subgenus: Hemihalictus; specificEpithet: truncaticolle; scientificNameAuthorship: (Morawitz, 1877); **Location:** country: Italy; countryCode: IT; stateProvince: Roma; locality: Riserva Naturale Tenuta dei Massimi 1; decimalLatitude: 41.8532859; decimalLongitude: 12.3842322; geodeticDatum: WGS84; coordinatePrecision: 0.0002; **Identification:** identifiedBy: M. Mei; **Event:** eventDate: 2022-06-01; **Record Level:** collectionID: UR3
- **Type status:** Other material. **Occurrence:** catalogNumber: A1694; recordedBy: L. Fortini; individualCount: 1; sex: male; lifeStage: adult; occurrenceID: 5F61D4AE-E69C-510A-BC86-DBC1E8F5DA13; **Taxon:** scientificName: Lasioglossum (Hemihalictus) truncaticolle (Morawitz, 1877); order: Hymenoptera; family: Halictidae; genus: Lasioglossum; subgenus: Hemihalictus; specificEpithet: truncaticolle; scientificNameAuthorship: (Morawitz, 1877); **Location:** country: Italy; countryCode: IT; stateProvince: Roma; locality: Riserva Naturale Tenuta dei Massimi 1; decimalLatitude: 41.8532859; decimalLongitude: 12.3842322; geodeticDatum: WGS84; coordinatePrecision: 0.0002; **Identification:** identifiedBy: M. Mei; **Event:** eventDate: 2022-06-27; **Record Level:** collectionID: UR3
- **Type status:** Other material. **Occurrence:** catalogNumber: A1626; recordedBy: L. Fortini; individualCount: 1; sex: male; lifeStage: adult; occurrenceID: 5663C5FC-217C-5C71-B89C-5B866C74606B; **Taxon:** scientificName: Lasioglossum (Hemihalictus) truncaticolle (Morawitz, 1877); order: Hymenoptera; family: Halictidae; genus: Lasioglossum; subgenus: Hemihalictus; specificEpithet: truncaticolle; scientificNameAuthorship: (Morawitz, 1877); **Location:** country: Italy; countryCode: IT; stateProvince: Roma; locality: Riserva Naturale Valle dei Casali 2; decimalLatitude: 41.8596887; decimalLongitude: 12.4355075; geodeticDatum: WGS84; coordinatePrecision: 0.0002; **Identification:** identifiedBy: M. Mei; **Event:** eventDate: 2022-07-13; **Record Level:** collectionID: UR3
- **Type status:** Other material. **Occurrence:** catalogNumber: A1530, A1538, A1539; recordedBy: L. Fortini; individualCount: 3; sex: females; lifeStage: adult; occurrenceID: 875CACD9-5A78-5006-AE64-ADBD7BEA20DE; **Taxon:** scientificName: Lasioglossum (Hemihalictus) truncaticolle (Morawitz, 1877); order: Hymenoptera; family: Halictidae; genus: Lasioglossum; subgenus: Hemihalictus; specificEpithet: truncaticolle; scientificNameAuthorship: (Morawitz, 1877); **Location:** country: Italy; countryCode: IT; stateProvince: Roma; locality: Riserva Naturale Tenuta dei Massimi 2; decimalLatitude: 41.8316516; decimalLongitude: 12.3999927; geodeticDatum: WGS84; coordinatePrecision: 0.0002; **Identification:** identifiedBy: M. Mei; **Event:** eventDate: 2022-07-28; **Record Level:** collectionID: UR3
- **Type status:** Other material. **Occurrence:** catalogNumber: A1482; recordedBy: L. Fortini; individualCount: 1; sex: female; lifeStage: adult; occurrenceID: 37381E23-CDA8-5E92-8CBF-01D776CB17B4; **Taxon:** scientificName: Lasioglossum (Hemihalictus) truncaticolle (Morawitz, 1877); order: Hymenoptera; family: Halictidae; genus: Lasioglossum; subgenus: Hemihalictus; specificEpithet: truncaticolle; scientificNameAuthorship: (Morawitz, 1877); **Location:** country: Italy; countryCode: IT; stateProvince: Roma; locality: Riserva Naturale dell'Insugherata 1; decimalLatitude: 41.9555045; decimalLongitude: 12.4292321; geodeticDatum: WGS84; coordinatePrecision: 0.0002; **Identification:** identifiedBy: M. Mei; **Event:** eventDate: 2022-07-30; **Record Level:** collectionID: UR3

#### Lasioglossum villosulum

(Kirby, 1802)

712A1C3F-E587-5FC5-BAAE-97773BE81D1A

##### Materials

- **Type status:** Other material. **Occurrence:** catalogNumber: A1286; recordedBy: L. Fortini; individualCount: 1; sex: female; lifeStage: adult; occurrenceID: 1A40D0E8-E0CC-5E21-9DE7-E0DE2E49FFCE; **Taxon:** scientificName: Lasioglossum (Hemihalictus) villosulum (Kirby, 1802); order: Hymenoptera; family: Halictidae; genus: Lasioglossum; subgenus: Hemihalictus; specificEpithet: villosulum; scientificNameAuthorship: (Kirby, 1802); **Location:** country: Italy; countryCode: IT; stateProvince: Roma; locality: Riserva Naturale Valle dei Casali 1; decimalLatitude: 41.8710627; decimalLongitude: 12.4336809; geodeticDatum: WGS84; coordinatePrecision: 0.0002; **Identification:** identifiedBy: M. Mei; **Event:** eventDate: 2022-04-07; **Record Level:** collectionID: UR3
- **Type status:** Other material. **Occurrence:** catalogNumber: A1321, A1325, A1330; recordedBy: L. Fortini; individualCount: 3; sex: females; lifeStage: adult; occurrenceID: 85AF3012-F882-5DB8-9E4C-7CA638C58007; **Taxon:** scientificName: Lasioglossum (Hemihalictus) villosulum (Kirby, 1802); order: Hymenoptera; family: Halictidae; genus: Lasioglossum; subgenus: Hemihalictus; specificEpithet: villosulum; scientificNameAuthorship: (Kirby, 1802); **Location:** country: Italy; countryCode: IT; stateProvince: Roma; locality: Riserva Naturale Tenuta dei Massimi 2; decimalLatitude: 41.8316516; decimalLongitude: 12.3999927; geodeticDatum: WGS84; coordinatePrecision: 0.0002; **Identification:** identifiedBy: M. Mei; **Event:** eventDate: 2022-05-04; **Record Level:** collectionID: UR3
- **Type status:** Other material. **Occurrence:** catalogNumber: A1340; recordedBy: L. Fortini; individualCount: 1; sex: female; lifeStage: adult; occurrenceID: 6689BA69-0FC0-5AD8-9121-C51284AA6A2E; **Taxon:** scientificName: Lasioglossum (Hemihalictus) villosulum (Kirby, 1802); order: Hymenoptera; family: Halictidae; genus: Lasioglossum; subgenus: Hemihalictus; specificEpithet: villosulum; scientificNameAuthorship: (Kirby, 1802); **Location:** country: Italy; countryCode: IT; stateProvince: Roma; locality: Riserva Naturale Laurentino-Acqua Acetosa; decimalLatitude: 41.8079275; decimalLongitude: 12.4685548; geodeticDatum: WGS84; coordinatePrecision: 0.0002; **Identification:** identifiedBy: M. Mei; **Event:** eventDate: 2022-05-12; **Record Level:** collectionID: UR3
- **Type status:** Other material. **Occurrence:** catalogNumber: A1250, A1333, A1342; recordedBy: L. Fortini; individualCount: 3; sex: females; lifeStage: adult; occurrenceID: 9F689F78-BDF6-511C-862B-8A4E63797902; **Taxon:** scientificName: Lasioglossum (Hemihalictus) villosulum (Kirby, 1802); order: Hymenoptera; family: Halictidae; genus: Lasioglossum; subgenus: Hemihalictus; specificEpithet: villosulum; scientificNameAuthorship: (Kirby, 1802); **Location:** country: Italy; countryCode: IT; stateProvince: Roma; locality: Riserva Naturale di Monte Mario; decimalLatitude: 41.9386215; decimalLongitude: 12.4546223; geodeticDatum: WGS84; coordinatePrecision: 0.0002; **Identification:** identifiedBy: M. Mei; **Event:** eventDate: 2022-05-20; **Record Level:** collectionID: UR3
- **Type status:** Other material. **Occurrence:** catalogNumber: A1349; recordedBy: L. Fortini; individualCount: 1; sex: male; lifeStage: adult; occurrenceID: 45E194A3-F817-51D2-944D-DA688F911510; **Taxon:** scientificName: Lasioglossum (Hemihalictus) villosulum (Kirby, 1802); order: Hymenoptera; family: Halictidae; genus: Lasioglossum; subgenus: Hemihalictus; specificEpithet: villosulum; scientificNameAuthorship: (Kirby, 1802); **Location:** country: Italy; countryCode: IT; stateProvince: Roma; locality: Riserva Regionale dell'Appia Antica 2; decimalLatitude: 41.8402564; decimalLongitude: 12.532773; geodeticDatum: WGS84; coordinatePrecision: 0.0002; **Identification:** identifiedBy: M. Mei; **Event:** eventDate: 2022-05-24; **Record Level:** collectionID: UR3
- **Type status:** Other material. **Occurrence:** catalogNumber: A1401, A1402; recordedBy: L. Fortini; individualCount: 2; sex: males; lifeStage: adult; occurrenceID: C93BD690-A87E-590E-884B-25FC86580518; **Taxon:** scientificName: Lasioglossum (Hemihalictus) villosulum (Kirby, 1802); order: Hymenoptera; family: Halictidae; genus: Lasioglossum; subgenus: Hemihalictus; specificEpithet: villosulum; scientificNameAuthorship: (Kirby, 1802); **Location:** country: Italy; countryCode: IT; stateProvince: Roma; locality: Riserva Naturale Tenuta dei Massimi 1; decimalLatitude: 41.8532859; decimalLongitude: 12.3842322; geodeticDatum: WGS84; coordinatePrecision: 0.0002; **Identification:** identifiedBy: M. Mei; **Event:** eventDate: 2022-06-01; **Record Level:** collectionID: UR3
- **Type status:** Other material. **Occurrence:** catalogNumber: A1406, A1409, A1411; recordedBy: L. Fortini; individualCount: 3; sex: males; lifeStage: adult; occurrenceID: 36195FE3-9819-5773-BE41-88DAF5FDE2E1; **Taxon:** scientificName: Lasioglossum (Hemihalictus) villosulum (Kirby, 1802); order: Hymenoptera; family: Halictidae; genus: Lasioglossum; subgenus: Hemihalictus; specificEpithet: villosulum; scientificNameAuthorship: (Kirby, 1802); **Location:** country: Italy; countryCode: IT; stateProvince: Roma; locality: Riserva Naturale Tenuta dei Massimi 2; decimalLatitude: 41.8316516; decimalLongitude: 12.3999927; geodeticDatum: WGS84; coordinatePrecision: 0.0002; **Identification:** identifiedBy: M. Mei; **Event:** eventDate: 2022-06-01; **Record Level:** collectionID: UR3
- **Type status:** Other material. **Occurrence:** catalogNumber: A1408; recordedBy: L. Fortini; individualCount: 1; sex: male; lifeStage: adult; occurrenceID: 862B216B-37AD-5D1B-A63D-FFC1A4035860; **Taxon:** scientificName: Lasioglossum (Hemihalictus) villosulum (Kirby, 1802); order: Hymenoptera; family: Halictidae; genus: Lasioglossum; subgenus: Hemihalictus; specificEpithet: villosulum; scientificNameAuthorship: (Kirby, 1802); **Location:** country: Italy; countryCode: IT; stateProvince: Roma; locality: Riserva Naturale Valle dell'Aniene 2; decimalLatitude: 41.928752; decimalLongitude: 12.5562962; geodeticDatum: WGS84; coordinatePrecision: 0.0002; **Identification:** identifiedBy: M. Mei; **Event:** eventDate: 2022-06-05; **Record Level:** collectionID: UR3
- **Type status:** Other material. **Occurrence:** catalogNumber: A1352; recordedBy: L. Fortini; individualCount: 1; sex: male; lifeStage: adult; occurrenceID: 8FF22F1D-8392-5BA7-B0F4-1725EBC53E9C; **Taxon:** scientificName: Lasioglossum (Hemihalictus) villosulum (Kirby, 1802); order: Hymenoptera; family: Halictidae; genus: Lasioglossum; subgenus: Hemihalictus; specificEpithet: villosulum; scientificNameAuthorship: (Kirby, 1802); **Location:** country: Italy; countryCode: IT; stateProvince: Roma; locality: Riserva Naturale dell'Acquafredda; decimalLatitude: 41.8928408; decimalLongitude: 12.39932; geodeticDatum: WGS84; coordinatePrecision: 0.0002; **Identification:** identifiedBy: M. Mei; **Event:** eventDate: 2022-06-10; **Record Level:** collectionID: UR3
- **Type status:** Other material. **Occurrence:** catalogNumber: A1345; recordedBy: L. Fortini; individualCount: 1; sex: female; lifeStage: adult; occurrenceID: 840D0E58-6370-5BD5-B97D-4877EA22F9D9; **Taxon:** scientificName: Lasioglossum (Hemihalictus) villosulum (Kirby, 1802); order: Hymenoptera; family: Halictidae; genus: Lasioglossum; subgenus: Hemihalictus; specificEpithet: villosulum; scientificNameAuthorship: (Kirby, 1802); **Location:** country: Italy; countryCode: IT; stateProvince: Roma; locality: Riserva Regionale dell'Appia Antica 1; decimalLatitude: 41.8623941; decimalLongitude: 12.524863; geodeticDatum: WGS84; coordinatePrecision: 0.0002; **Identification:** identifiedBy: M. Mei; **Event:** eventDate: 2022-06-12; **Record Level:** collectionID: UR3
- **Type status:** Other material. **Occurrence:** catalogNumber: A1376; recordedBy: L. Fortini; individualCount: 1; sex: female; lifeStage: adult; occurrenceID: 063E89EA-0E8D-5295-A9D5-F2E25DAEF0F6; **Taxon:** scientificName: Lasioglossum (Hemihalictus) villosulum (Kirby, 1802); order: Hymenoptera; family: Halictidae; genus: Lasioglossum; subgenus: Hemihalictus; specificEpithet: villosulum; scientificNameAuthorship: (Kirby, 1802); **Location:** country: Italy; countryCode: IT; stateProvince: Roma; locality: Riserva Naturale Laurentino-Acqua Acetosa; decimalLatitude: 41.8079275; decimalLongitude: 12.4685548; geodeticDatum: WGS84; coordinatePrecision: 0.0002; **Identification:** identifiedBy: M. Mei; **Event:** eventDate: 2022-06-16; **Record Level:** collectionID: UR3
- **Type status:** Other material. **Occurrence:** catalogNumber: A1361; recordedBy: L. Fortini; individualCount: 1; sex: male; lifeStage: adult; occurrenceID: 9928B5B6-76C3-5849-BEC0-F51986437318; **Taxon:** scientificName: Lasioglossum (Hemihalictus) villosulum (Kirby, 1802); order: Hymenoptera; family: Halictidae; genus: Lasioglossum; subgenus: Hemihalictus; specificEpithet: villosulum; scientificNameAuthorship: (Kirby, 1802); **Location:** country: Italy; countryCode: IT; stateProvince: Roma; locality: Riserva Naturale Valle dei Casali 1; decimalLatitude: 41.8710627; decimalLongitude: 12.4336809; geodeticDatum: WGS84; coordinatePrecision: 0.0002; **Identification:** identifiedBy: M. Mei; **Event:** eventDate: 2022-06-18; **Record Level:** collectionID: UR3
- **Type status:** Other material. **Occurrence:** catalogNumber: A1437, A1439; recordedBy: L. Fortini; individualCount: 2; sex: males; lifeStage: adult; occurrenceID: 81613A75-1849-5556-9044-3278D0FF125B; **Taxon:** scientificName: Lasioglossum (Hemihalictus) villosulum (Kirby, 1802); order: Hymenoptera; family: Halictidae; genus: Lasioglossum; subgenus: Hemihalictus; specificEpithet: villosulum; scientificNameAuthorship: (Kirby, 1802); **Location:** country: Italy; countryCode: IT; stateProvince: Roma; locality: Riserva Naturale di Monte Mario; decimalLatitude: 41.9386215; decimalLongitude: 12.4546223; geodeticDatum: WGS84; coordinatePrecision: 0.0002; **Identification:** identifiedBy: M. Mei; **Event:** eventDate: 2022-06-19; **Record Level:** collectionID: UR3
- **Type status:** Other material. **Occurrence:** catalogNumber: A1691; recordedBy: L. Fortini; individualCount: 1; sex: male; lifeStage: adult; occurrenceID: E4150CD1-5DED-530B-80A0-3DF69EE923AE; **Taxon:** scientificName: Lasioglossum (Hemihalictus) villosulum (Kirby, 1802); order: Hymenoptera; family: Halictidae; genus: Lasioglossum; subgenus: Hemihalictus; specificEpithet: villosulum; scientificNameAuthorship: (Kirby, 1802); **Location:** country: Italy; countryCode: IT; stateProvince: Roma; locality: Riserva Naturale Tenuta dei Massimi 1; decimalLatitude: 41.8532859; decimalLongitude: 12.3842322; geodeticDatum: WGS84; coordinatePrecision: 0.0002; **Identification:** identifiedBy: M. Mei; **Event:** eventDate: 2022-06-27; **Record Level:** collectionID: UR3
- **Type status:** Other material. **Occurrence:** catalogNumber: A1625; recordedBy: L. Fortini; individualCount: 1; sex: male; lifeStage: adult; occurrenceID: B5660D15-3BC7-5351-8B2F-511FADB5E644; **Taxon:** scientificName: Lasioglossum (Hemihalictus) villosulum (Kirby, 1802); order: Hymenoptera; family: Halictidae; genus: Lasioglossum; subgenus: Hemihalictus; specificEpithet: villosulum; scientificNameAuthorship: (Kirby, 1802); **Location:** country: Italy; countryCode: IT; stateProvince: Roma; locality: Riserva Naturale Valle dell'Aniene 2; decimalLatitude: 41.928752; decimalLongitude: 12.5562962; geodeticDatum: WGS84; coordinatePrecision: 0.0002; **Identification:** identifiedBy: M. Mei; **Event:** eventDate: 2022-07-01; **Record Level:** collectionID: UR3
- **Type status:** Other material. **Occurrence:** catalogNumber: A1692; recordedBy: L. Fortini; individualCount: 1; sex: male; lifeStage: adult; occurrenceID: DF22AB06-37F4-5238-BB8D-C5FD1CF32263; **Taxon:** scientificName: Lasioglossum (Hemihalictus) villosulum (Kirby, 1802); order: Hymenoptera; family: Halictidae; genus: Lasioglossum; subgenus: Hemihalictus; specificEpithet: villosulum; scientificNameAuthorship: (Kirby, 1802); **Location:** country: Italy; countryCode: IT; stateProvince: Roma; locality: Riserva Regionale dell'Appia Antica 2; decimalLatitude: 41.8402564; decimalLongitude: 12.532773; geodeticDatum: WGS84; coordinatePrecision: 0.0002; **Identification:** identifiedBy: M. Mei; **Event:** eventDate: 2022-07-07; **Record Level:** collectionID: UR3
- **Type status:** Other material. **Occurrence:** catalogNumber: A1483, A1574, A1585; recordedBy: L. Fortini; individualCount: 3; sex: males; lifeStage: adult; occurrenceID: 27E8783E-080C-505F-9687-8A98B95225DC; **Taxon:** scientificName: Lasioglossum (Hemihalictus) villosulum (Kirby, 1802); order: Hymenoptera; family: Halictidae; genus: Lasioglossum; subgenus: Hemihalictus; specificEpithet: villosulum; scientificNameAuthorship: (Kirby, 1802); **Location:** country: Italy; countryCode: IT; stateProvince: Roma; locality: Riserva Naturale dell'Acquafredda; decimalLatitude: 41.8928408; decimalLongitude: 12.39932; geodeticDatum: WGS84; coordinatePrecision: 0.0002; **Identification:** identifiedBy: M. Mei; **Event:** eventDate: 2022-07-12; **Record Level:** collectionID: UR3
- **Type status:** Other material. **Occurrence:** catalogNumber: A1479, A1488; recordedBy: L. Fortini; individualCount: 2; sex: females; lifeStage: adult; occurrenceID: 23242934-7BD9-5F6B-A8DE-C5B33A079A47; **Taxon:** scientificName: Lasioglossum (Hemihalictus) villosulum (Kirby, 1802); order: Hymenoptera; family: Halictidae; genus: Lasioglossum; subgenus: Hemihalictus; specificEpithet: villosulum; scientificNameAuthorship: (Kirby, 1802); **Location:** country: Italy; countryCode: IT; stateProvince: Roma; locality: Riserva Naturale Valle dei Casali 2; decimalLatitude: 41.8596887; decimalLongitude: 12.4355075; geodeticDatum: WGS84; coordinatePrecision: 0.0002; **Identification:** identifiedBy: M. Mei; **Event:** eventDate: 2022-07-13; **Record Level:** collectionID: UR3
- **Type status:** Other material. **Occurrence:** catalogNumber: A1469; recordedBy: L. Fortini; individualCount: 1; sex: female; lifeStage: adult; occurrenceID: 00251AE6-E76E-56A9-9C53-F8551ED34E80; **Taxon:** scientificName: Lasioglossum (Hemihalictus) villosulum (Kirby, 1802); order: Hymenoptera; family: Halictidae; genus: Lasioglossum; subgenus: Hemihalictus; specificEpithet: villosulum; scientificNameAuthorship: (Kirby, 1802); **Location:** country: Italy; countryCode: IT; stateProvince: Roma; locality: Riserva Naturale Tenuta dei Massimi 1; decimalLatitude: 41.8532859; decimalLongitude: 12.3842322; geodeticDatum: WGS84; coordinatePrecision: 0.0002; **Identification:** identifiedBy: M. Mei; **Event:** eventDate: 2022-07-28; **Record Level:** collectionID: UR3
- **Type status:** Other material. **Occurrence:** catalogNumber: A1607; recordedBy: L. Fortini; individualCount: 1; sex: male; lifeStage: adult; occurrenceID: 4E563492-4636-585F-9D33-670C0D6992FA; **Taxon:** scientificName: Lasioglossum (Hemihalictus) villosulum (Kirby, 1802); order: Hymenoptera; family: Halictidae; genus: Lasioglossum; subgenus: Hemihalictus; specificEpithet: villosulum; scientificNameAuthorship: (Kirby, 1802); **Location:** country: Italy; countryCode: IT; stateProvince: Roma; locality: Riserva Naturale dell'Acquafredda; decimalLatitude: 41.8928408; decimalLongitude: 12.39932; geodeticDatum: WGS84; coordinatePrecision: 0.0002; **Identification:** identifiedBy: M. Mei; **Event:** eventDate: 2022-08-17; **Record Level:** collectionID: UR3
- **Type status:** Other material. **Occurrence:** catalogNumber: A2036, A2037, A2038, A2039, A2040; recordedBy: L. Fortini; individualCount: 5; sex: females; lifeStage: adult; occurrenceID: 36B684E6-69A7-560C-BF16-57B9469F0714; **Taxon:** scientificName: Lasioglossum (Hemihalictus) villosulum (Kirby, 1802); order: Hymenoptera; family: Halictidae; genus: Lasioglossum; subgenus: Hemihalictus; specificEpithet: villosulum; scientificNameAuthorship: (Kirby, 1802); **Location:** country: Italy; countryCode: IT; stateProvince: Roma; locality: Riserva Naturale Valle dei Casali 1; decimalLatitude: 41.8710627; decimalLongitude: 12.4336809; geodeticDatum: WGS84; coordinatePrecision: 0.0002; **Identification:** identifiedBy: M. Mei; **Event:** eventDate: 2022-08-19; **Record Level:** collectionID: UR3
- **Type status:** Other material. **Occurrence:** catalogNumber: A1516; recordedBy: L. Fortini; individualCount: 1; sex: female; lifeStage: adult; occurrenceID: 34A81601-9A85-57FF-B1CA-3B091CCCBF7F; **Taxon:** scientificName: Lasioglossum (Hemihalictus) villosulum (Kirby, 1802); order: Hymenoptera; family: Halictidae; genus: Lasioglossum; subgenus: Hemihalictus; specificEpithet: villosulum; scientificNameAuthorship: (Kirby, 1802); **Location:** country: Italy; countryCode: IT; stateProvince: Roma; locality: Riserva Naturale Laurentino-Acqua Acetosa; decimalLatitude: 41.8079275; decimalLongitude: 12.4685548; geodeticDatum: WGS84; coordinatePrecision: 0.0002; **Identification:** identifiedBy: M. Mei; **Event:** eventDate: 2022-08-21; **Record Level:** collectionID: UR3
- **Type status:** Other material. **Occurrence:** catalogNumber: A1522, A1550; recordedBy: L. Fortini; individualCount: 2; sex: females; lifeStage: adult; occurrenceID: D605D6B1-E455-5437-BC83-7A47AE660402; **Taxon:** scientificName: Lasioglossum (Hemihalictus) villosulum (Kirby, 1802); order: Hymenoptera; family: Halictidae; genus: Lasioglossum; subgenus: Hemihalictus; specificEpithet: villosulum; scientificNameAuthorship: (Kirby, 1802); **Location:** country: Italy; countryCode: IT; stateProvince: Roma; locality: Riserva Naturale di Monte Mario; decimalLatitude: 41.9386215; decimalLongitude: 12.4546223; geodeticDatum: WGS84; coordinatePrecision: 0.0002; **Identification:** identifiedBy: M. Mei; **Event:** eventDate: 2022-08-23; **Record Level:** collectionID: UR3
- **Type status:** Other material. **Occurrence:** catalogNumber: A1611; recordedBy: L. Fortini; individualCount: 1; sex: female; lifeStage: adult; occurrenceID: 5C315DBB-F936-5981-9700-6DF2EA0365B6; **Taxon:** scientificName: Lasioglossum (Hemihalictus) villosulum (Kirby, 1802); order: Hymenoptera; family: Halictidae; genus: Lasioglossum; subgenus: Hemihalictus; specificEpithet: villosulum; scientificNameAuthorship: (Kirby, 1802); **Location:** country: Italy; countryCode: IT; stateProvince: Roma; locality: Riserva Regionale dell'Appia Antica 1; decimalLatitude: 41.8623941; decimalLongitude: 12.524863; geodeticDatum: WGS84; coordinatePrecision: 0.0002; **Identification:** identifiedBy: M. Mei; **Event:** eventDate: 2022-08-25; **Record Level:** collectionID: UR3
- **Type status:** Other material. **Occurrence:** catalogNumber: A1546; recordedBy: L. Fortini; individualCount: 1; sex: female; lifeStage: adult; occurrenceID: 65A331B2-E7D4-5D5C-BF6E-671F0BA335D0; **Taxon:** scientificName: Lasioglossum (Hemihalictus) villosulum (Kirby, 1802); order: Hymenoptera; family: Halictidae; genus: Lasioglossum; subgenus: Hemihalictus; specificEpithet: villosulum; scientificNameAuthorship: (Kirby, 1802); **Location:** country: Italy; countryCode: IT; stateProvince: Roma; locality: Riserva Naturale Tenuta dei Massimi 2; decimalLatitude: 41.8316516; decimalLongitude: 12.3999927; geodeticDatum: WGS84; coordinatePrecision: 0.0002; **Identification:** identifiedBy: M. Mei; **Event:** eventDate: 2022-08-27; **Record Level:** collectionID: UR3
- **Type status:** Other material. **Occurrence:** catalogNumber: A1551, A1558; recordedBy: L. Fortini; individualCount: 2; sex: females; lifeStage: adult; occurrenceID: 00131DEE-319E-59AA-B404-4534869BA7BB; **Taxon:** scientificName: Lasioglossum (Hemihalictus) villosulum (Kirby, 1802); order: Hymenoptera; family: Halictidae; genus: Lasioglossum; subgenus: Hemihalictus; specificEpithet: villosulum; scientificNameAuthorship: (Kirby, 1802); **Location:** country: Italy; countryCode: IT; stateProvince: Roma; locality: Riserva Naturale Tenuta dei Massimi 1; decimalLatitude: 41.8532859; decimalLongitude: 12.3842322; geodeticDatum: WGS84; coordinatePrecision: 0.0002; **Identification:** identifiedBy: M. Mei; **Event:** eventDate: 2022-08-27; **Record Level:** collectionID: UR3
- **Type status:** Other material. **Occurrence:** catalogNumber: A1605; recordedBy: L. Fortini; individualCount: 1; sex: male; lifeStage: adult; occurrenceID: 86224284-353E-553E-AB8E-D16773A63E61; **Taxon:** scientificName: Lasioglossum (Hemihalictus) villosulum (Kirby, 1802); order: Hymenoptera; family: Halictidae; genus: Lasioglossum; subgenus: Hemihalictus; specificEpithet: villosulum; scientificNameAuthorship: (Kirby, 1802); **Location:** country: Italy; countryCode: IT; stateProvince: Roma; locality: Riserva Naturale Valle dell'Aniene 2; decimalLatitude: 41.928752; decimalLongitude: 12.5562962; geodeticDatum: WGS84; coordinatePrecision: 0.0002; **Identification:** identifiedBy: M. Mei; **Event:** eventDate: 2022-09-04; **Record Level:** collectionID: UR3
- **Type status:** Other material. **Occurrence:** catalogNumber: A1495; recordedBy: L. Fortini; individualCount: 1; sex: female; lifeStage: adult; occurrenceID: 68B4D44E-BC01-5257-8C9E-DDC1F69048E3; **Taxon:** scientificName: Lasioglossum (Hemihalictus) villosulum (Kirby, 1802); order: Hymenoptera; family: Halictidae; genus: Lasioglossum; subgenus: Hemihalictus; specificEpithet: villosulum; scientificNameAuthorship: (Kirby, 1802); **Location:** country: Italy; countryCode: IT; stateProvince: Roma; locality: Riserva Naturale dell'Acquafredda; decimalLatitude: 41.8928408; decimalLongitude: 12.39932; geodeticDatum: WGS84; coordinatePrecision: 0.0002; **Identification:** identifiedBy: M. Mei; **Event:** eventDate: 2022-09-10; **Record Level:** collectionID: UR3
- **Type status:** Other material. **Occurrence:** catalogNumber: A1559; recordedBy: L. Fortini; individualCount: 1; sex: female; lifeStage: adult; occurrenceID: 66322FA9-D2DA-5B33-8283-2ACF107AAF9E; **Taxon:** scientificName: Lasioglossum (Hemihalictus) villosulum (Kirby, 1802); order: Hymenoptera; family: Halictidae; genus: Lasioglossum; subgenus: Hemihalictus; specificEpithet: villosulum; scientificNameAuthorship: (Kirby, 1802); **Location:** country: Italy; countryCode: IT; stateProvince: Roma; locality: Riserva Naturale Valle dei Casali 1; decimalLatitude: 41.8710627; decimalLongitude: 12.4336809; geodeticDatum: WGS84; coordinatePrecision: 0.0002; **Identification:** identifiedBy: M. Mei; **Event:** eventDate: 2022-09-18; **Record Level:** collectionID: UR3

#### Lasioglossum zonulus

(Smith, 1848)

085A9480-2015-5FD9-B152-1CDECC31D6ED

##### Materials

- **Type status:** Other material. **Occurrence:** catalogNumber: A1779; recordedBy: L. Fortini; individualCount: 1; sex: female; lifeStage: adult; occurrenceID: 90B89861-003B-5ED9-9EA3-19053F3CAEC5; **Taxon:** scientificName: Lasioglossum (Leuchalictus) zonulus (Smith, 1848); order: Hymenoptera; family: Halictidae; genus: Lasioglossum; subgenus: Leuchalictus; specificEpithet: zonulus; scientificNameAuthorship: (Smith, 1848); **Location:** country: Italy; countryCode: IT; stateProvince: Roma; locality: Riserva Naturale dell'Insugherata 2; decimalLatitude: 41.9599247; decimalLongitude: 12.433852; geodeticDatum: WGS84; coordinatePrecision: 0.0002; **Identification:** identifiedBy: M. Mei; **Event:** eventDate: 2022-09-01; **Record Level:** collectionID: UR3
- **Type status:** Other material. **Occurrence:** catalogNumber: A2056, A2057; recordedBy: L. Fortini; individualCount: 2; sex: females; lifeStage: adult; occurrenceID: D9102CDA-2552-53F8-8C37-B7765B01BF88; **Taxon:** scientificName: Lasioglossum (Leuchalictus) zonulus (Smith, 1848); order: Hymenoptera; family: Halictidae; genus: Lasioglossum; subgenus: Leuchalictus; specificEpithet: zonulus; scientificNameAuthorship: (Smith, 1848); **Location:** country: Italy; countryCode: IT; stateProvince: Roma; locality: Riserva Naturale dell'Insugherata 3; decimalLatitude: 41.9644829; decimalLongitude: 12.436101; geodeticDatum: WGS84; coordinatePrecision: 0.0002; **Identification:** identifiedBy: M. Mei; **Event:** eventDate: 2022-07-30; **Record Level:** collectionID: UR3

#### Seladonia gemmea

(Dours, 1872)

97897337-1973-53DE-B89D-29E8EBC12F46

##### Materials

- **Type status:** Other material. **Occurrence:** catalogNumber: A1569, A1570; recordedBy: L. Fortini; individualCount: 2; sex: females; lifeStage: adult; occurrenceID: 53F9366E-C0D9-51CE-9998-30435936DCAB; **Taxon:** scientificName: Seladonia (Seladonia) gemmea (Dours, 1872); order: Hymenoptera; family: Halictidae; genus: Seladonia; subgenus: Seladonia; specificEpithet: gemmea; scientificNameAuthorship: (Dours, 1872); **Location:** country: Italy; countryCode: IT; stateProvince: Roma; locality: Riserva Naturale dell'Acquafredda; decimalLatitude: 41.8928408; decimalLongitude: 12.39932; geodeticDatum: WGS84; coordinatePrecision: 0.0002; **Identification:** identifiedBy: M. Mei; **Event:** eventDate: 2022-07-12; **Record Level:** collectionID: UR3
- **Type status:** Other material. **Occurrence:** catalogNumber: A1568; recordedBy: L. Fortini; individualCount: 1; sex: female; lifeStage: adult; occurrenceID: E2D6B345-C9C2-5711-A3B4-271E92A48640; **Taxon:** scientificName: Seladonia (Seladonia) gemmea (Dours, 1872); order: Hymenoptera; family: Halictidae; genus: Seladonia; subgenus: Seladonia; specificEpithet: gemmea; scientificNameAuthorship: (Dours, 1872); **Location:** country: Italy; countryCode: IT; stateProvince: Roma; locality: Riserva Regionale dell'Appia Antica 2; decimalLatitude: 41.8402564; decimalLongitude: 12.532773; geodeticDatum: WGS84; coordinatePrecision: 0.0002; **Identification:** identifiedBy: M. Mei; **Event:** eventDate: 2022-07-07; **Record Level:** collectionID: UR3
- **Type status:** Other material. **Occurrence:** catalogNumber: A1573, A1591; recordedBy: L. Fortini; individualCount: 2; sex: females; lifeStage: adult; occurrenceID: 6355E47E-FE9F-5F1B-BC76-8FA54FC6701F; **Taxon:** scientificName: Seladonia (Seladonia) gemmea (Dours, 1872); order: Hymenoptera; family: Halictidae; genus: Seladonia; subgenus: Seladonia; specificEpithet: gemmea; scientificNameAuthorship: (Dours, 1872); **Location:** country: Italy; countryCode: IT; stateProvince: Roma; locality: Riserva Regionale dell'Appia Antica 1; decimalLatitude: 41.8623941; decimalLongitude: 12.524863; geodeticDatum: WGS84; coordinatePrecision: 0.0002; **Identification:** identifiedBy: M. Mei; **Event:** eventDate: 2022-07-22; **Record Level:** collectionID: UR3
- **Type status:** Other material. **Occurrence:** catalogNumber: A1577, A1598, A1599; recordedBy: L. Fortini; individualCount: 3; sex: females; lifeStage: adult; occurrenceID: 8E1A620B-1D74-5B5C-8493-6B801780B961; **Taxon:** scientificName: Seladonia (Seladonia) gemmea (Dours, 1872); order: Hymenoptera; family: Halictidae; genus: Seladonia; subgenus: Seladonia; specificEpithet: gemmea; scientificNameAuthorship: (Dours, 1872); **Location:** country: Italy; countryCode: IT; stateProvince: Roma; locality: Riserva Naturale dell'Insugherata 1; decimalLatitude: 41.9555045; decimalLongitude: 12.4292321; geodeticDatum: WGS84; coordinatePrecision: 0.0002; **Identification:** identifiedBy: M. Mei; **Event:** eventDate: 2022-07-30; **Record Level:** collectionID: UR3
- **Type status:** Other material. **Occurrence:** catalogNumber: A1560; recordedBy: L. Fortini; individualCount: 1; sex: female; lifeStage: adult; occurrenceID: E5F650BB-C724-5FFD-9D97-636E75FBC481; **Taxon:** scientificName: Seladonia (Seladonia) gemmea (Dours, 1872); order: Hymenoptera; family: Halictidae; genus: Seladonia; subgenus: Seladonia; specificEpithet: gemmea; scientificNameAuthorship: (Dours, 1872); **Location:** country: Italy; countryCode: IT; stateProvince: Roma; locality: Riserva Naturale Laurentino-Acqua Acetosa; decimalLatitude: 41.8079275; decimalLongitude: 12.4685548; geodeticDatum: WGS84; coordinatePrecision: 0.0002; **Identification:** identifiedBy: M. Mei; **Event:** eventDate: 2022-08-21; **Record Level:** collectionID: UR3
- **Type status:** Other material. **Occurrence:** catalogNumber: A1604; recordedBy: L. Fortini; individualCount: 1; sex: female; lifeStage: adult; occurrenceID: E3CC9C8C-C8C6-5B3F-8B39-8016FD9C13C4; **Taxon:** scientificName: Seladonia (Seladonia) gemmea (Dours, 1872); order: Hymenoptera; family: Halictidae; genus: Seladonia; subgenus: Seladonia; specificEpithet: gemmea; scientificNameAuthorship: (Dours, 1872); **Location:** country: Italy; countryCode: IT; stateProvince: Roma; locality: Riserva Naturale Laurentino-Acqua Acetosa; decimalLatitude: 41.8079275; decimalLongitude: 12.4685548; geodeticDatum: WGS84; coordinatePrecision: 0.0002; **Identification:** identifiedBy: M. Mei; **Event:** eventDate: 2022-07-17; **Record Level:** collectionID: UR3
- **Type status:** Other material. **Occurrence:** catalogNumber: A1594, A1602; recordedBy: L. Fortini; individualCount: 2; sex: females; lifeStage: adult; occurrenceID: BF3FBFB8-E007-5B6F-84D6-F84949824C3F; **Taxon:** scientificName: Seladonia (Seladonia) gemmea (Dours, 1872); order: Hymenoptera; family: Halictidae; genus: Seladonia; subgenus: Seladonia; specificEpithet: gemmea; scientificNameAuthorship: (Dours, 1872); **Location:** country: Italy; countryCode: IT; stateProvince: Roma; locality: Riserva Naturale Tenuta dei Massimi 2; decimalLatitude: 41.8316516; decimalLongitude: 12.3999927; geodeticDatum: WGS84; coordinatePrecision: 0.0002; **Identification:** identifiedBy: M. Mei; **Event:** eventDate: 2022-07-28; **Record Level:** collectionID: UR3
- **Type status:** Other material. **Occurrence:** catalogNumber: A1677, A1678, A1680, A1681, A1682, A1683, A1684, A1689; recordedBy: L. Fortini; individualCount: 8; sex: females; lifeStage: adult; occurrenceID: 80936835-A1D5-5F20-AE2F-5DEA98A1DFCA; **Taxon:** scientificName: Seladonia (Seladonia) gemmea (Dours, 1872); order: Hymenoptera; family: Halictidae; genus: Seladonia; subgenus: Seladonia; specificEpithet: gemmea; scientificNameAuthorship: (Dours, 1872); **Location:** country: Italy; countryCode: IT; stateProvince: Roma; locality: Riserva Naturale Valle dell'Aniene 1; decimalLatitude: 41.9345179; decimalLongitude: 12.5453096; geodeticDatum: WGS84; coordinatePrecision: 0.0002; **Identification:** identifiedBy: M. Mei; **Event:** eventDate: 2022-07-01; **Record Level:** collectionID: UR3
- **Type status:** Other material. **Occurrence:** catalogNumber: A1679, A1685, A1686, A1688; recordedBy: L. Fortini; individualCount: 4; sex: females; lifeStage: adult; occurrenceID: 13E81BE2-85D5-5846-98E2-29C3A0858D0D; **Taxon:** scientificName: Seladonia (Seladonia) gemmea (Dours, 1872); order: Hymenoptera; family: Halictidae; genus: Seladonia; subgenus: Seladonia; specificEpithet: gemmea; scientificNameAuthorship: (Dours, 1872); **Location:** country: Italy; countryCode: IT; stateProvince: Roma; locality: Riserva Naturale Valle dell'Aniene 2; decimalLatitude: 41.928752; decimalLongitude: 12.5562962; geodeticDatum: WGS84; coordinatePrecision: 0.0002; **Identification:** identifiedBy: M. Mei; **Event:** eventDate: 2022-07-01; **Record Level:** collectionID: UR3
- **Type status:** Other material. **Occurrence:** catalogNumber: A1754; recordedBy: L. Fortini; individualCount: 1; sex: female; lifeStage: adult; occurrenceID: 8703921D-B469-5CC9-8E3E-729B9FFF36E2; **Taxon:** scientificName: Seladonia (Seladonia) gemmea (Dours, 1872); order: Hymenoptera; family: Halictidae; genus: Seladonia; subgenus: Seladonia; specificEpithet: gemmea; scientificNameAuthorship: (Dours, 1872); **Location:** country: Italy; countryCode: IT; stateProvince: Roma; locality: Riserva Naturale Valle dell'Aniene 2; decimalLatitude: 41.928752; decimalLongitude: 12.5562962; geodeticDatum: WGS84; coordinatePrecision: 0.0002; **Identification:** identifiedBy: M. Mei; **Event:** eventDate: 2022-06-05; **Record Level:** collectionID: UR3
- **Type status:** Other material. **Occurrence:** catalogNumber: A1572, A1589; recordedBy: L. Fortini; individualCount: 2; sex: females; lifeStage: adult; occurrenceID: A31D865E-B746-5F5D-A58F-DF60264AF829; **Taxon:** scientificName: Seladonia (Seladonia) gemmea (Dours, 1872); order: Hymenoptera; family: Halictidae; genus: Seladonia; subgenus: Seladonia; specificEpithet: gemmea; scientificNameAuthorship: (Dours, 1872); **Location:** country: Italy; countryCode: IT; stateProvince: Roma; locality: Riserva Naturale Valle dei Casali 1; decimalLatitude: 41.8710627; decimalLongitude: 12.4336809; geodeticDatum: WGS84; coordinatePrecision: 0.0002; **Identification:** identifiedBy: M. Mei; **Event:** eventDate: 2022-07-13; **Record Level:** collectionID: UR3
- **Type status:** Other material. **Occurrence:** catalogNumber: A1575, A1582; recordedBy: L. Fortini; individualCount: 2; sex: females; lifeStage: adult; occurrenceID: D39D5E3E-E366-59F3-9814-9EB5C19966A3; **Taxon:** scientificName: Seladonia (Seladonia) gemmea (Dours, 1872); order: Hymenoptera; family: Halictidae; genus: Seladonia; subgenus: Seladonia; specificEpithet: gemmea; scientificNameAuthorship: (Dours, 1872); **Location:** country: Italy; countryCode: IT; stateProvince: Roma; locality: Riserva Naturale Valle dei Casali 2; decimalLatitude: 41.8596887; decimalLongitude: 12.4355075; geodeticDatum: WGS84; coordinatePrecision: 0.0002; **Identification:** identifiedBy: M. Mei; **Event:** eventDate: 2022-07-13; **Record Level:** collectionID: UR3
- **Type status:** Other material. **Occurrence:** catalogNumber: A2033, A2034, A2046, A2050; recordedBy: L. Fortini; individualCount: 4; sex: females; lifeStage: adult; occurrenceID: 20F57EFD-20B9-5C50-B33D-7868B8107BA2; **Taxon:** scientificName: Seladonia (Seladonia) gemmea (Dours, 1872); order: Hymenoptera; family: Halictidae; genus: Seladonia; subgenus: Seladonia; specificEpithet: gemmea; scientificNameAuthorship: (Dours, 1872); **Location:** country: Italy; countryCode: IT; stateProvince: Roma; locality: Riserva Naturale Valle dei Casali 1; decimalLatitude: 41.8710627; decimalLongitude: 12.4336809; geodeticDatum: WGS84; coordinatePrecision: 0.0002; **Identification:** identifiedBy: M. Mei; **Event:** eventDate: 2022-08-19; **Record Level:** collectionID: UR3
- **Type status:** Other material. **Occurrence:** catalogNumber: A2135, A2136; recordedBy: L. Fortini; individualCount: 2; sex: females; lifeStage: adult; occurrenceID: 2F93EC2D-4959-5C84-B0F8-E9D2A6EF7BC5; **Taxon:** scientificName: Seladonia (Seladonia) gemmea (Dours, 1872); order: Hymenoptera; family: Halictidae; genus: Seladonia; subgenus: Seladonia; specificEpithet: gemmea; scientificNameAuthorship: (Dours, 1872); **Location:** country: Italy; countryCode: IT; stateProvince: Roma; locality: Riserva Naturale Valle dell'Aniene 2; decimalLatitude: 41.928752; decimalLongitude: 12.5562962; geodeticDatum: WGS84; coordinatePrecision: 0.0002; **Identification:** identifiedBy: M. Mei; **Event:** eventDate: 2022-08-03; **Record Level:** collectionID: UR3

#### Seladonia pollinosa

(Sichel, 1860)

1DBEAF14-4AD1-5F39-8DA6-5BD84151AA03

##### Materials

- **Type status:** Other material. **Occurrence:** catalogNumber: A1744; recordedBy: L. Fortini; individualCount: 1; sex: female; lifeStage: adult; occurrenceID: 6033CC4B-82A1-5678-A942-BD886A85BA5D; **Taxon:** scientificName: Seladonia (Mucoreohalictus) pollinosa (Sichel, 1860); order: Hymenoptera; family: Halictidae; genus: Seladonia; subgenus: Mucoreohalictus; specificEpithet: pollinosa; scientificNameAuthorship: (Sichel, 1860); **Location:** country: Italy; countryCode: IT; stateProvince: Roma; locality: Riserva Naturale di Monte Mario; decimalLatitude: 41.9386215; decimalLongitude: 12.4546223; geodeticDatum: WGS84; coordinatePrecision: 0.0002; **Identification:** identifiedBy: M. Mei; **Event:** eventDate: 2022-06-19; **Record Level:** collectionID: UR3

#### Seladonia smaragdula

(Vachal, 1895)

F3081E1A-378B-5755-84D6-2D1065778046

##### Materials

- **Type status:** Other material. **Occurrence:** catalogNumber: A1571; recordedBy: L. Fortini; individualCount: 1; sex: female; lifeStage: adult; occurrenceID: 751C95F0-7D49-5ED7-9014-30F3933006AF; **Taxon:** scientificName: Seladonia (Seladonia) smaragdula (Vachal, 1895); order: Hymenoptera; family: Halictidae; genus: Seladonia; subgenus: Seladonia; specificEpithet: smaragdula; scientificNameAuthorship: (Vachal, 1895); **Location:** country: Italy; countryCode: IT; stateProvince: Roma; locality: Riserva Naturale dell'Acquafredda; decimalLatitude: 41.8928408; decimalLongitude: 12.39932; geodeticDatum: WGS84; coordinatePrecision: 0.0002; **Identification:** identifiedBy: M. Mei; **Event:** eventDate: 2022-07-12; **Record Level:** collectionID: UR3
- **Type status:** Other material. **Occurrence:** catalogNumber: A1592, A1595, A1596; recordedBy: L. Fortini; individualCount: 3; sex: females; lifeStage: adult; occurrenceID: 3F9EE130-B1E9-52E1-9645-D214DF231151; **Taxon:** scientificName: Seladonia (Seladonia) smaragdula (Vachal, 1895); order: Hymenoptera; family: Halictidae; genus: Seladonia; subgenus: Seladonia; specificEpithet: smaragdula; scientificNameAuthorship: (Vachal, 1895); **Location:** country: Italy; countryCode: IT; stateProvince: Roma; locality: Riserva Regionale dell'Appia Antica 1; decimalLatitude: 41.8623941; decimalLongitude: 12.524863; geodeticDatum: WGS84; coordinatePrecision: 0.0002; **Identification:** identifiedBy: M. Mei; **Event:** eventDate: 2022-07-22; **Record Level:** collectionID: UR3
- **Type status:** Other material. **Occurrence:** catalogNumber: A1593, A1597; recordedBy: L. Fortini; individualCount: 2; sex: females; lifeStage: adult; occurrenceID: 6D580D05-08ED-5D90-B780-75DD93C9D05A; **Taxon:** scientificName: Seladonia (Seladonia) smaragdula (Vachal, 1895); order: Hymenoptera; family: Halictidae; genus: Seladonia; subgenus: Seladonia; specificEpithet: smaragdula; scientificNameAuthorship: (Vachal, 1895); **Location:** country: Italy; countryCode: IT; stateProvince: Roma; locality: Riserva Naturale Laurentino-Acqua Acetosa; decimalLatitude: 41.8079275; decimalLongitude: 12.4685548; geodeticDatum: WGS84; coordinatePrecision: 0.0002; **Identification:** identifiedBy: M. Mei; **Event:** eventDate: 2022-07-17; **Record Level:** collectionID: UR3
- **Type status:** Other material. **Occurrence:** catalogNumber: A1608; recordedBy: L. Fortini; individualCount: 1; sex: female; lifeStage: adult; occurrenceID: 346C5C8A-D633-5055-8FF0-681EA423C380; **Taxon:** scientificName: Seladonia (Seladonia) smaragdula (Vachal, 1895); order: Hymenoptera; family: Halictidae; genus: Seladonia; subgenus: Seladonia; specificEpithet: smaragdula; scientificNameAuthorship: (Vachal, 1895); **Location:** country: Italy; countryCode: IT; stateProvince: Roma; locality: Riserva Naturale Laurentino-Acqua Acetosa; decimalLatitude: 41.8079275; decimalLongitude: 12.4685548; geodeticDatum: WGS84; coordinatePrecision: 0.0002; **Identification:** identifiedBy: M. Mei; **Event:** eventDate: 2022-08-21; **Record Level:** collectionID: UR3
- **Type status:** Other material. **Occurrence:** catalogNumber: A1600, A1601, A1606, A1609, A1610; recordedBy: L. Fortini; individualCount: 5; sex: females; lifeStage: adult; occurrenceID: 6A26A504-CA2F-5B0C-88E2-5963AAA159F8; **Taxon:** scientificName: Seladonia (Seladonia) smaragdula (Vachal, 1895); order: Hymenoptera; family: Halictidae; genus: Seladonia; subgenus: Seladonia; specificEpithet: smaragdula; scientificNameAuthorship: (Vachal, 1895); **Location:** country: Italy; countryCode: IT; stateProvince: Roma; locality: Riserva Naturale Tenuta dei Massimi 2; decimalLatitude: 41.8316516; decimalLongitude: 12.3999927; geodeticDatum: WGS84; coordinatePrecision: 0.0002; **Identification:** identifiedBy: M. Mei; **Event:** eventDate: 2022-07-28; **Record Level:** collectionID: UR3
- **Type status:** Other material. **Occurrence:** catalogNumber: A1687, A1690; recordedBy: L. Fortini; individualCount: 2; sex: females; lifeStage: adult; occurrenceID: 66131622-DD00-54CB-8097-D75AFA705E7D; **Taxon:** scientificName: Seladonia (Seladonia) smaragdula (Vachal, 1895); order: Hymenoptera; family: Halictidae; genus: Seladonia; subgenus: Seladonia; specificEpithet: smaragdula; scientificNameAuthorship: (Vachal, 1895); **Location:** country: Italy; countryCode: IT; stateProvince: Roma; locality: Riserva Naturale Valle dell'Aniene 2; decimalLatitude: 41.928752; decimalLongitude: 12.5562962; geodeticDatum: WGS84; coordinatePrecision: 0.0002; **Identification:** identifiedBy: M. Mei; **Event:** eventDate: 2022-07-01; **Record Level:** collectionID: UR3
- **Type status:** Other material. **Occurrence:** catalogNumber: A1581; recordedBy: L. Fortini; individualCount: 1; sex: female; lifeStage: adult; occurrenceID: DE745321-074A-50BE-8BD4-96C0D29CA4A3; **Taxon:** scientificName: Seladonia (Seladonia) smaragdula (Vachal, 1895); order: Hymenoptera; family: Halictidae; genus: Seladonia; subgenus: Seladonia; specificEpithet: smaragdula; scientificNameAuthorship: (Vachal, 1895); **Location:** country: Italy; countryCode: IT; stateProvince: Roma; locality: Riserva Naturale Valle dei Casali 2; decimalLatitude: 41.8596887; decimalLongitude: 12.4355075; geodeticDatum: WGS84; coordinatePrecision: 0.0002; **Identification:** identifiedBy: M. Mei; **Event:** eventDate: 2022-07-13; **Record Level:** collectionID: UR3
- **Type status:** Other material. **Occurrence:** catalogNumber: A2051; recordedBy: L. Fortini; individualCount: 1; sex: female; lifeStage: adult; occurrenceID: 9354DD6D-380C-55E4-89F5-984DFE2A4554; **Taxon:** scientificName: Seladonia (Seladonia) smaragdula (Vachal, 1895); order: Hymenoptera; family: Halictidae; genus: Seladonia; subgenus: Seladonia; specificEpithet: smaragdula; scientificNameAuthorship: (Vachal, 1895); **Location:** country: Italy; countryCode: IT; stateProvince: Roma; locality: Riserva Naturale Valle dei Casali 1; decimalLatitude: 41.8710627; decimalLongitude: 12.4336809; geodeticDatum: WGS84; coordinatePrecision: 0.0002; **Identification:** identifiedBy: M. Mei; **Event:** eventDate: 2022-08-19; **Record Level:** collectionID: UR3
- **Type status:** Other material. **Occurrence:** catalogNumber: A2055, A2060; recordedBy: L. Fortini; individualCount: 2; sex: females; lifeStage: adult; occurrenceID: 4199BCEB-C3AD-56F9-8D3D-AC1CC8EC3E9A; **Taxon:** scientificName: Seladonia (Seladonia) smaragdula (Vachal, 1895); order: Hymenoptera; family: Halictidae; genus: Seladonia; subgenus: Seladonia; specificEpithet: smaragdula; scientificNameAuthorship: (Vachal, 1895); **Location:** country: Italy; countryCode: IT; stateProvince: Roma; locality: Riserva Naturale dell'Insugherata 3; decimalLatitude: 41.9644829; decimalLongitude: 12.436101; geodeticDatum: WGS84; coordinatePrecision: 0.0002; **Identification:** identifiedBy: M. Mei; **Event:** eventDate: 2022-07-30; **Record Level:** collectionID: UR3
- **Type status:** Other material. **Occurrence:** catalogNumber: A2064; recordedBy: L. Fortini; individualCount: 1; sex: female; lifeStage: adult; occurrenceID: F3F5B97A-E299-5F33-9834-9B26295ED748; **Taxon:** scientificName: Seladonia (Seladonia) smaragdula (Vachal, 1895); order: Hymenoptera; family: Halictidae; genus: Seladonia; subgenus: Seladonia; specificEpithet: smaragdula; scientificNameAuthorship: (Vachal, 1895); **Location:** country: Italy; countryCode: IT; stateProvince: Roma; locality: Riserva Naturale di Monte Mario; decimalLatitude: 41.9386215; decimalLongitude: 12.4546223; geodeticDatum: WGS84; coordinatePrecision: 0.0002; **Identification:** identifiedBy: M. Mei; **Event:** eventDate: 2022-07-24; **Record Level:** collectionID: UR3
- **Type status:** Other material. **Occurrence:** catalogNumber: A2113; recordedBy: L. Fortini; individualCount: 1; sex: female; lifeStage: adult; occurrenceID: 3FE2FEFF-9008-5464-9388-55CA208CFE58; **Taxon:** scientificName: Seladonia (Seladonia) smaragdula (Vachal, 1895); order: Hymenoptera; family: Halictidae; genus: Seladonia; subgenus: Seladonia; specificEpithet: smaragdula; scientificNameAuthorship: (Vachal, 1895); **Location:** country: Italy; countryCode: IT; stateProvince: Roma; locality: Riserva Naturale Valle dell'Aniene 2; decimalLatitude: 41.928752; decimalLongitude: 12.5562962; geodeticDatum: WGS84; coordinatePrecision: 0.0002; **Identification:** identifiedBy: M. Mei; **Event:** eventDate: 2022-08-03; **Record Level:** collectionID: UR3

#### Seladonia subaurata

(Rossi, 1792)

2AC4CA88-01A6-5DE5-AA8A-BA22A39F91E2

##### Materials

- **Type status:** Other material. **Occurrence:** catalogNumber: A1672, A1674; recordedBy: L. Fortini; individualCount: 2; sex: 1 male, 1 female; lifeStage: adult; occurrenceID: A9863F2B-AF50-5A3B-867F-7CEE4CB0C00C; **Taxon:** scientificName: Seladonia (Seladonia) subaurata (Rossi, 1792); order: Hymenoptera; family: Halictidae; genus: Seladonia; subgenus: Seladonia; specificEpithet: subaurata; scientificNameAuthorship: (Rossi, 1792); **Location:** country: Italy; countryCode: IT; stateProvince: Roma; locality: Riserva Regionale dell'Appia Antica 2; decimalLatitude: 41.8402564; decimalLongitude: 12.532773; geodeticDatum: WGS84; coordinatePrecision: 0.0002; **Identification:** identifiedBy: M. Mei; **Event:** eventDate: 2022-08-29; **Record Level:** collectionID: UR3
- **Type status:** Other material. **Occurrence:** catalogNumber: A1753; recordedBy: L. Fortini; individualCount: 1; sex: female; lifeStage: adult; occurrenceID: B8C212E2-1D4C-5066-BF46-E0E3D93434A7; **Taxon:** scientificName: Seladonia (Seladonia) subaurata (Rossi, 1792); order: Hymenoptera; family: Halictidae; genus: Seladonia; subgenus: Seladonia; specificEpithet: subaurata; scientificNameAuthorship: (Rossi, 1792); **Location:** country: Italy; countryCode: IT; stateProvince: Roma; locality: Riserva Regionale dell'Appia Antica 1; decimalLatitude: 41.8623941; decimalLongitude: 12.524863; geodeticDatum: WGS84; coordinatePrecision: 0.0002; **Identification:** identifiedBy: M. Mei; **Event:** eventDate: 2022-05-10; **Record Level:** collectionID: UR3
- **Type status:** Other material. **Occurrence:** catalogNumber: A1671; recordedBy: L. Fortini; individualCount: 1; sex: female; lifeStage: adult; occurrenceID: 7C6047D7-8FFE-5309-B7C2-BAA620F47D46; **Taxon:** scientificName: Seladonia (Seladonia) subaurata (Rossi, 1792); order: Hymenoptera; family: Halictidae; genus: Seladonia; subgenus: Seladonia; specificEpithet: subaurata; scientificNameAuthorship: (Rossi, 1792); **Location:** country: Italy; countryCode: IT; stateProvince: Roma; locality: Riserva Naturale Valle dei Casali 1; decimalLatitude: 41.8710627; decimalLongitude: 12.4336809; geodeticDatum: WGS84; coordinatePrecision: 0.0002; **Identification:** identifiedBy: M. Mei; **Event:** eventDate: 2022-07-13; **Record Level:** collectionID: UR3

#### Seladonia vestita

(Lepeletier, 1841)

2F1D628E-625D-5C13-826C-C62E959C218B

##### Materials

- **Type status:** Other material. **Occurrence:** catalogNumber: A1603; recordedBy: L. Fortini; individualCount: 1; sex: female; lifeStage: adult; occurrenceID: F82646E5-2D6A-58E0-B57E-3362E78C5E78; **Taxon:** scientificName: Seladonia (Vestitohalictus) vestita (Lepeletier, 1841); order: Hymenoptera; family: Halictidae; genus: Seladonia; subgenus: Vestitohalictus; specificEpithet: vestita; scientificNameAuthorship: (Lepeletier, 1841); **Location:** country: Italy; countryCode: IT; stateProvince: Roma; locality: Riserva Regionale dell'Appia Antica 1; decimalLatitude: 41.8623941; decimalLongitude: 12.524863; geodeticDatum: WGS84; coordinatePrecision: 0.0002; **Identification:** identifiedBy: M. Mei; **Event:** eventDate: 2022-07-22; **Record Level:** collectionID: UR3

#### Sphecodes gibbus

(Linnaeus, 1758)

DA9EC2E9-1D3A-5675-AEB4-140B18E7F6E6

##### Materials

- **Type status:** Other material. **Occurrence:** catalogNumber: A0461; recordedBy: L. Fortini; individualCount: 1; sex: female; lifeStage: adult; occurrenceID: 1A9A9146-CABB-5F82-B521-456D883D25FE; **Taxon:** scientificName: Sphecodes (Sphecodes) gibbus (Linnaeus,1758); order: Hymenoptera; family: Halictidae; genus: Sphecodes; subgenus: Sphecodes; specificEpithet: gibbus; scientificNameAuthorship: (Linnaeus, 1758); **Location:** country: Italy; countryCode: IT; stateProvince: Roma; locality: Riserva Naturale di Monte Mario; decimalLatitude: 41.9386215; decimalLongitude: 12.4546223; geodeticDatum: WGS84; coordinatePrecision: 0.0002; **Identification:** identifiedBy: M. Mei; **Event:** eventDate: 2022-04-20; **Record Level:** collectionID: UR3
- **Type status:** Other material. **Occurrence:** catalogNumber: A0462; recordedBy: L. Fortini; individualCount: 1; sex: female; lifeStage: adult; occurrenceID: 7439D143-5369-5D88-A02B-B5068FF27D03; **Taxon:** scientificName: Sphecodes (Sphecodes) gibbus (Linnaeus,1758); order: Hymenoptera; family: Halictidae; genus: Sphecodes; subgenus: Sphecodes; specificEpithet: gibbus; scientificNameAuthorship: (Linnaeus, 1758); **Location:** country: Italy; countryCode: IT; stateProvince: Roma; locality: Riserva Regionale dell'Appia Antica 1; decimalLatitude: 41.8623941; decimalLongitude: 12.524863; geodeticDatum: WGS84; coordinatePrecision: 0.0002; **Identification:** identifiedBy: M. Mei; **Event:** eventDate: 2022-06-12; **Record Level:** collectionID: UR3

#### Sphecodes longulus

Hagens, 1882

572E26E5-E416-5150-A64E-81DDADC888A0

##### Materials

- **Type status:** Other material. **Occurrence:** catalogNumber: A0460; recordedBy: L. Fortini; individualCount: 1; sex: female; lifeStage: adult; occurrenceID: 2E9219B9-2B82-50C3-989D-81ACD666F08D; **Taxon:** scientificName: Sphecodes (Sphecodes) longulus Hagens, 1882; order: Hymenoptera; family: Halictidae; genus: Sphecodes; subgenus: Sphecodes; specificEpithet: longulus; scientificNameAuthorship: Hagens, 1882; **Location:** country: Italy; countryCode: IT; stateProvince: Roma; locality: Riserva Naturale dell'Insugherata 3; decimalLatitude: 41.9644829; decimalLongitude: 12.436101; geodeticDatum: WGS84; coordinatePrecision: 0.0002; **Identification:** identifiedBy: M. Mei; **Event:** eventDate: 2022-04-15; **Record Level:** collectionID: UR3

#### Nomioides facilis

(Smith, 1853)

13F84836-C698-5E19-A28F-612F423F925C

##### Materials

- **Type status:** Other material. **Occurrence:** catalogNumber: A0324; recordedBy: L. Fortini; individualCount: 1; sex: male; lifeStage: adult; occurrenceID: D92FDCF5-DFE9-5F7B-B1C5-16A37F29A178; **Taxon:** scientificName: Nomioides (Nomioides) facilis (Smith, 1853); order: Hymenoptera; family: Halictidae; genus: Nomioides; subgenus: Nomioides; specificEpithet: facilis; scientificNameAuthorship: (Smith, 1853); **Location:** country: Italy; countryCode: IT; stateProvince: Roma; locality: Riserva Regionale dell'Appia Antica 2; decimalLatitude: 41.8402564; decimalLongitude: 12.532773; geodeticDatum: WGS84; coordinatePrecision: 0.0002; **Identification:** identifiedBy: M. Mei; **Event:** eventDate: 2022-07-07; **Record Level:** collectionID: UR3
- **Type status:** Other material. **Occurrence:** catalogNumber: A0326, A0328; recordedBy: L. Fortini; individualCount: 2; sex: females; lifeStage: adult; occurrenceID: 6EDD0C4D-1FCA-5468-888A-CF2CE66F27EB; **Taxon:** scientificName: Nomioides (Nomioides) facilis (Smith, 1853); order: Hymenoptera; family: Halictidae; genus: Nomioides; subgenus: Nomioides; specificEpithet: facilis; scientificNameAuthorship: (Smith, 1853); **Location:** country: Italy; countryCode: IT; stateProvince: Roma; locality: Riserva Regionale dell'Appia Antica 2; decimalLatitude: 41.8402564; decimalLongitude: 12.532773; geodeticDatum: WGS84; coordinatePrecision: 0.0002; **Identification:** identifiedBy: M. Mei; **Event:** eventDate: 2022-08-29; **Record Level:** collectionID: UR3
- **Type status:** Other material. **Occurrence:** catalogNumber: A0327; recordedBy: L. Fortini; individualCount: 1; sex: female; lifeStage: adult; occurrenceID: 82C7F235-A404-5592-BF0C-2C34CD762E5B; **Taxon:** scientificName: Nomioides (Nomioides) facilis (Smith, 1853); order: Hymenoptera; family: Halictidae; genus: Nomioides; subgenus: Nomioides; specificEpithet: facilis; scientificNameAuthorship: (Smith, 1853); **Location:** country: Italy; countryCode: IT; stateProvince: Roma; locality: Riserva Regionale dell'Appia Antica 1; decimalLatitude: 41.8623941; decimalLongitude: 12.524863; geodeticDatum: WGS84; coordinatePrecision: 0.0002; **Identification:** identifiedBy: M. Mei; **Event:** eventDate: 2022-05-10; **Record Level:** collectionID: UR3
- **Type status:** Other material. **Occurrence:** catalogNumber: A0325; recordedBy: L. Fortini; individualCount: 1; sex: male; lifeStage: adult; occurrenceID: 50BCBA60-A2E8-5180-A3B6-603969528A0F; **Taxon:** scientificName: Nomioides (Nomioides) facilis (Smith, 1853); order: Hymenoptera; family: Halictidae; genus: Nomioides; subgenus: Nomioides; specificEpithet: facilis; scientificNameAuthorship: (Smith, 1853); **Location:** country: Italy; countryCode: IT; stateProvince: Roma; locality: Riserva Naturale dell'Insugherata 1; decimalLatitude: 41.9555045; decimalLongitude: 12.4292321; geodeticDatum: WGS84; coordinatePrecision: 0.0002; **Identification:** identifiedBy: M. Mei; **Event:** eventDate: 2022-07-30; **Record Level:** collectionID: UR3
- **Type status:** Other material. **Occurrence:** catalogNumber: A0329, A0330; recordedBy: L. Fortini; individualCount: 2; sex: females; lifeStage: adult; occurrenceID: 3A786BEC-BE6A-560D-BC6B-9544F839C283; **Taxon:** scientificName: Nomioides (Nomioides) facilis (Smith, 1853); order: Hymenoptera; family: Halictidae; genus: Nomioides; subgenus: Nomioides; specificEpithet: facilis; scientificNameAuthorship: (Smith, 1853); **Location:** country: Italy; countryCode: IT; stateProvince: Roma; locality: Riserva Naturale Tenuta dei Massimi 2; decimalLatitude: 41.8316516; decimalLongitude: 12.3999927; geodeticDatum: WGS84; coordinatePrecision: 0.0002; **Identification:** identifiedBy: M. Mei; **Event:** eventDate: 2022-06-01; **Record Level:** collectionID: UR3

#### Systropha curvicornis

(Scopoli, 1770)

FEBF0F46-DBC4-5E2A-AF60-0311B6252737

##### Materials

- **Type status:** Other material. **Occurrence:** catalogNumber: A0001; recordedBy: L. Fortini; individualCount: 1; sex: male; lifeStage: adult; occurrenceID: 79087FEC-5BAA-5690-8197-53DC517319CD; **Taxon:** scientificName: Systropha (Systropha) curvicornis (Scopoli, 1770); order: Hymenoptera; family: Halictidae; genus: Systropha; subgenus: Systropha; specificEpithet: curvicornis; scientificNameAuthorship: (Scopoli 1770); **Location:** country: Italy; countryCode: IT; stateProvince: Roma; locality: Riserva Naturale dell'Insugherata 1; decimalLatitude: 41.9555045; decimalLongitude: 12.4292321; geodeticDatum: WGS84; coordinatePrecision: 0.0002; **Identification:** identifiedBy: M. Mei; **Event:** eventDate: 2022-06-24; **Record Level:** collectionID: UR3
- **Type status:** Other material. **Occurrence:** catalogNumber: A0002, A0006, A0007; recordedBy: L. Fortini; individualCount: 3; sex: 2 males, 1 female; lifeStage: adult; occurrenceID: B4B25F32-8D05-5955-8D4B-7DE644D66151; **Taxon:** scientificName: Systropha (Systropha) curvicornis (Scopoli, 1770); order: Hymenoptera; family: Halictidae; genus: Systropha; subgenus: Systropha; specificEpithet: curvicornis; scientificNameAuthorship: (Scopoli 1770); **Location:** country: Italy; countryCode: IT; stateProvince: Roma; locality: Riserva Naturale Valle dei Casali 2; decimalLatitude: 41.8596887; decimalLongitude: 12.4355075; geodeticDatum: WGS84; coordinatePrecision: 0.0002; **Identification:** identifiedBy: M. Mei; **Event:** eventDate: 2022-06-18; **Record Level:** collectionID: UR3
- **Type status:** Other material. **Occurrence:** catalogNumber: A0003, A0004, A0005, A0010; recordedBy: L. Fortini; individualCount: 4; sex: males; lifeStage: adult; occurrenceID: 28383AC2-2C2F-59C2-BA3F-A328EB9436F9; **Taxon:** scientificName: Systropha (Systropha) curvicornis (Scopoli, 1770); order: Hymenoptera; family: Halictidae; genus: Systropha; subgenus: Systropha; specificEpithet: curvicornis; scientificNameAuthorship: (Scopoli 1770); **Location:** country: Italy; countryCode: IT; stateProvince: Roma; locality: Riserva Naturale dell'Acquafredda; decimalLatitude: 41.8928408; decimalLongitude: 12.39932; geodeticDatum: WGS84; coordinatePrecision: 0.0002; **Identification:** identifiedBy: M. Mei; **Event:** eventDate: 2022-06-10; **Record Level:** collectionID: UR3
- **Type status:** Other material. **Occurrence:** catalogNumber: A0008; recordedBy: L. Fortini; individualCount: 1; sex: female; lifeStage: adult; occurrenceID: ECED3097-640A-5E28-8DC9-03AC047576F7; **Taxon:** scientificName: Systropha (Systropha) curvicornis (Scopoli, 1770); order: Hymenoptera; family: Halictidae; genus: Systropha; subgenus: Systropha; specificEpithet: curvicornis; scientificNameAuthorship: (Scopoli 1770); **Location:** country: Italy; countryCode: IT; stateProvince: Roma; locality: Riserva Naturale dell'Acquafredda; decimalLatitude: 41.8928408; decimalLongitude: 12.39932; geodeticDatum: WGS84; coordinatePrecision: 0.0002; **Identification:** identifiedBy: M. Mei; **Event:** eventDate: 2022-07-12; **Record Level:** collectionID: UR3
- **Type status:** Other material. **Occurrence:** catalogNumber: A0009; recordedBy: L. Fortini; individualCount: 1; sex: male; lifeStage: adult; occurrenceID: E130C3A4-5CE0-5450-A6D1-20DE302255C8; **Taxon:** scientificName: Systropha (Systropha) curvicornis (Scopoli, 1770); order: Hymenoptera; family: Halictidae; genus: Systropha; subgenus: Systropha; specificEpithet: curvicornis; scientificNameAuthorship: (Scopoli 1770); **Location:** country: Italy; countryCode: IT; stateProvince: Roma; locality: Riserva Naturale dell'Insugherata 3; decimalLatitude: 41.9644829; decimalLongitude: 12.436101; geodeticDatum: WGS84; coordinatePrecision: 0.0002; **Identification:** identifiedBy: M. Mei; **Event:** eventDate: 2022-06-24; **Record Level:** collectionID: UR3
- **Type status:** Other material. **Occurrence:** catalogNumber: A0011; recordedBy: L. Fortini; individualCount: 1; sex: female; lifeStage: adult; occurrenceID: 60B2DC8C-CFCA-5732-98A7-981AD1093C78; **Taxon:** scientificName: Systropha (Systropha) curvicornis (Scopoli, 1770); order: Hymenoptera; family: Halictidae; genus: Systropha; subgenus: Systropha; specificEpithet: curvicornis; scientificNameAuthorship: (Scopoli 1770); **Location:** country: Italy; countryCode: IT; stateProvince: Roma; locality: Riserva Naturale dell'Insugherata 2; decimalLatitude: 41.9599247; decimalLongitude: 12.433852; geodeticDatum: WGS84; coordinatePrecision: 0.0002; **Identification:** identifiedBy: M. Mei; **Event:** eventDate: 2022-06-24; **Record Level:** collectionID: UR3

### Megachilidae Latreille, 1802

#### Anthidiellum strigatum

(Panzer, 1805)

A66B8230-7FCF-551F-AB8C-38F1A79134B7

##### Materials

- **Type status:** Other material. **Occurrence:** catalogNumber: A0182; recordedBy: L. Fortini; individualCount: 1; sex: male; lifeStage: adult; occurrenceID: 33BE0626-24CA-5645-871D-1D32C6B91816; **Taxon:** scientificName: Anthidiellum (Anthidiellum) strigatum (Panzer, 1804); order: Hymenoptera; family: Megachilidae; genus: Anthidiellum; subgenus: Anthidiellum; specificEpithet: strigatum; scientificNameAuthorship: (Panzer, 1804); **Location:** country: Italy; countryCode: IT; stateProvince: Roma; locality: Riserva Regionale dell'Appia Antica 2; decimalLatitude: 41.8402564; decimalLongitude: 12.532773; geodeticDatum: WGS84; coordinatePrecision: 0.0002; **Identification:** identifiedBy: M. Mei; **Event:** eventDate: 2022-07-13; **Record Level:** collectionCode: UR3
- **Type status:** Other material. **Occurrence:** catalogNumber: A0183; recordedBy: L. Fortini; individualCount: 1; sex: male; lifeStage: adult; occurrenceID: D4E4DBBA-79C9-53A1-9006-A8CBA3825F9C; **Taxon:** scientificName: Anthidiellum (Anthidiellum) strigatum (Panzer, 1804); order: Hymenoptera; family: Megachilidae; genus: Anthidiellum; subgenus: Anthidiellum; specificEpithet: strigatum; scientificNameAuthorship: (Panzer, 1804); **Location:** country: Italy; countryCode: IT; stateProvince: Roma; locality: Riserva Regionale dell'Appia Antica 2; decimalLatitude: 41.8402564; decimalLongitude: 12.532773; geodeticDatum: WGS84; coordinatePrecision: 0.0002; **Identification:** identifiedBy: M. Mei; **Event:** eventDate: 2022-06-05; **Record Level:** collectionCode: UR3
- **Type status:** Other material. **Occurrence:** catalogNumber: A0184; recordedBy: L. Fortini; individualCount: 1; sex: male; lifeStage: adult; occurrenceID: 876F8020-CE42-5C19-9B9F-FF7B1D3F3471; **Taxon:** scientificName: Anthidiellum (Anthidiellum) strigatum (Panzer, 1804); order: Hymenoptera; family: Megachilidae; genus: Anthidiellum; subgenus: Anthidiellum; specificEpithet: strigatum; scientificNameAuthorship: (Panzer, 1804); **Location:** country: Italy; countryCode: IT; stateProvince: Roma; locality: Riserva Regionale dell'Appia Antica 2; decimalLatitude: 41.8402564; decimalLongitude: 12.532773; geodeticDatum: WGS84; coordinatePrecision: 0.0002; **Identification:** identifiedBy: M. Mei; **Event:** eventDate: 2022-08-29; **Record Level:** collectionCode: UR3
- **Type status:** Other material. **Occurrence:** catalogNumber: A0181; recordedBy: L. Fortini; individualCount: 1; sex: male; lifeStage: adult; occurrenceID: C2B22AD0-F514-553C-BDFB-C0113DEBC7E1; **Taxon:** scientificName: Anthidiellum (Anthidiellum) strigatum (Panzer, 1804); order: Hymenoptera; family: Megachilidae; genus: Anthidiellum; subgenus: Anthidiellum; specificEpithet: strigatum; scientificNameAuthorship: (Panzer, 1804); **Location:** country: Italy; countryCode: IT; stateProvince: Roma; locality: Riserva Naturale Valle dell'Aniene 1; decimalLatitude: 41.9345179; decimalLongitude: 12.5453096; geodeticDatum: WGS84; coordinatePrecision: 0.0002; **Identification:** identifiedBy: M. Mei; **Event:** eventDate: 2022-08-29; **Record Level:** collectionCode: UR3
- **Type status:** Other material. **Occurrence:** catalogNumber: A0180; recordedBy: L. Fortini; individualCount: 1; sex: male; lifeStage: adult; occurrenceID: 7A206DCD-2637-5475-9DCA-57D1CBB94A3F; **Taxon:** scientificName: Anthidiellum (Anthidiellum) strigatum (Panzer, 1804); order: Hymenoptera; family: Megachilidae; genus: Anthidiellum; subgenus: Anthidiellum; specificEpithet: strigatum; scientificNameAuthorship: (Panzer, 1804); **Location:** country: Italy; countryCode: IT; stateProvince: Roma; locality: Riserva Naturale Valle dei Casali 1; decimalLatitude: 41.8710627; decimalLongitude: 12.4336809; geodeticDatum: WGS84; coordinatePrecision: 0.0002; **Identification:** identifiedBy: M. Mei; **Event:** eventDate: 2022-08-29; **Record Level:** collectionCode: UR3
- **Type status:** Other material. **Occurrence:** catalogNumber: A0590; recordedBy: L. Fortini; individualCount: 1; sex: female; lifeStage: adult; occurrenceID: 34733CA1-AAB2-52E8-9E05-3D9217F40B94; **Taxon:** scientificName: Anthidiellum (Anthidiellum) strigatum (Panzer, 1804); order: Hymenoptera; family: Megachilidae; genus: Anthidiellum; subgenus: Anthidiellum; specificEpithet: strigatum; scientificNameAuthorship: (Panzer, 1804); **Location:** country: Italy; countryCode: IT; stateProvince: Roma; locality: Riserva Naturale Laurentino-Acqua Acetosa; decimalLatitude: 41.8079275; decimalLongitude: 12.4685548; geodeticDatum: WGS84; coordinatePrecision: 0.0002; **Identification:** identifiedBy: M. Mei; **Event:** eventDate: 2022-09-14; **Record Level:** collectionCode: UR3

#### Anthidium florentinum

(Fabricius, 1775)

454068F3-5265-5C38-8187-584091EACC0D

##### Materials

- **Type status:** Other material. **Occurrence:** catalogNumber: A0121; recordedBy: L. Fortini; individualCount: 1; sex: male; lifeStage: adult; occurrenceID: E019D163-1999-5F95-8CDA-E795241ECC5A; **Taxon:** scientificName: Anthidium (Anthidium) florentinum (Fabricius, 1775); order: Hymenoptera; family: Megachilidae; genus: Anthidium; subgenus: Anthidium; specificEpithet: florentinum; scientificNameAuthorship: (Fabricius, 1775); **Location:** country: Italy; countryCode: IT; stateProvince: Roma; locality: Riserva Naturale dell'Acquafredda; decimalLatitude: 41.8928408; decimalLongitude: 12.39932; geodeticDatum: WGS84; coordinatePrecision: 0.0002; **Identification:** identifiedBy: M. Mei; **Event:** eventDate: 2022-06-10; **Record Level:** collectionCode: UR3
- **Type status:** Other material. **Occurrence:** catalogNumber: A0124; recordedBy: L. Fortini; individualCount: 1; sex: male; lifeStage: adult; occurrenceID: B9B52DF3-A119-5B34-830B-9C4B6273528E; **Taxon:** scientificName: Anthidium (Anthidium) florentinum (Fabricius, 1775); order: Hymenoptera; family: Megachilidae; genus: Anthidium; subgenus: Anthidium; specificEpithet: florentinum; scientificNameAuthorship: (Fabricius, 1775); **Location:** country: Italy; countryCode: IT; stateProvince: Roma; locality: Riserva Naturale dell'Acquafredda; decimalLatitude: 41.8928408; decimalLongitude: 12.39932; geodeticDatum: WGS84; coordinatePrecision: 0.0002; **Identification:** identifiedBy: M. Mei; **Event:** eventDate: 2022-07-12; **Record Level:** collectionCode: UR3
- **Type status:** Other material. **Occurrence:** catalogNumber: A0119, A0120, A0126, A0128, A0131, A0135; recordedBy: L. Fortini; individualCount: 6; sex: 2 males, 4 females; lifeStage: adult; occurrenceID: A1779C28-07E5-51B4-9069-ABFF5BF5D137; **Taxon:** scientificName: Anthidium (Anthidium) florentinum (Fabricius, 1775); order: Hymenoptera; family: Megachilidae; genus: Anthidium; subgenus: Anthidium; specificEpithet: florentinum; scientificNameAuthorship: (Fabricius, 1775); **Location:** country: Italy; countryCode: IT; stateProvince: Roma; locality: Riserva Regionale dell'Appia Antica 1; decimalLatitude: 41.8623941; decimalLongitude: 12.524863; geodeticDatum: WGS84; coordinatePrecision: 0.0002; **Identification:** identifiedBy: M. Mei; **Event:** eventDate: 2022-06-12; **Record Level:** collectionCode: UR3
- **Type status:** Other material. **Occurrence:** catalogNumber: A0127, A0129, A0137; recordedBy: L. Fortini; individualCount: 3; sex: females; lifeStage: adult; occurrenceID: C822E67B-3E2D-58FC-B986-2977F3CAB802; **Taxon:** scientificName: Anthidium (Anthidium) florentinum (Fabricius, 1775); order: Hymenoptera; family: Megachilidae; genus: Anthidium; subgenus: Anthidium; specificEpithet: florentinum; scientificNameAuthorship: (Fabricius, 1775); **Location:** country: Italy; countryCode: IT; stateProvince: Roma; locality: Riserva Naturale dell'Insugherata 3; decimalLatitude: 41.9644829; decimalLongitude: 12.436101; geodeticDatum: WGS84; coordinatePrecision: 0.0002; **Identification:** identifiedBy: M. Mei; **Event:** eventDate: 2022-07-30; **Record Level:** collectionCode: UR3
- **Type status:** Other material. **Occurrence:** catalogNumber: A0133, A0136; recordedBy: L. Fortini; individualCount: 2; sex: females; lifeStage: adult; occurrenceID: 346060CE-D7F0-5890-B5A9-9123F59BFE83; **Taxon:** scientificName: Anthidium (Anthidium) florentinum (Fabricius, 1775); order: Hymenoptera; family: Megachilidae; genus: Anthidium; subgenus: Anthidium; specificEpithet: florentinum; scientificNameAuthorship: (Fabricius, 1775); **Location:** country: Italy; countryCode: IT; stateProvince: Roma; locality: Riserva Naturale Valle dell'Aniene 1; decimalLatitude: 41.9345179; decimalLongitude: 12.5453096; geodeticDatum: WGS84; coordinatePrecision: 0.0002; **Identification:** identifiedBy: M. Mei; **Event:** eventDate: 2022-07-01; **Record Level:** collectionCode: UR3
- **Type status:** Other material. **Occurrence:** catalogNumber: A0122, A0123, A0132, A0134; recordedBy: L. Fortini; individualCount: 4; sex: 2 males, 2 females; lifeStage: adult; occurrenceID: EC94F170-C76F-5E02-B364-B92C895014BE; **Taxon:** scientificName: Anthidium (Anthidium) florentinum (Fabricius, 1775); order: Hymenoptera; family: Megachilidae; genus: Anthidium; subgenus: Anthidium; specificEpithet: florentinum; scientificNameAuthorship: (Fabricius, 1775); **Location:** country: Italy; countryCode: IT; stateProvince: Roma; locality: Riserva Naturale Valle dell'Aniene 2; decimalLatitude: 41.928752; decimalLongitude: 12.5562962; geodeticDatum: WGS84; coordinatePrecision: 0.0002; **Identification:** identifiedBy: M. Mei; **Event:** eventDate: 2022-07-01; **Record Level:** collectionCode: UR3
- **Type status:** Other material. **Occurrence:** catalogNumber: A0130; recordedBy: L. Fortini; individualCount: 1; sex: female; lifeStage: adult; occurrenceID: 94E10E18-ADC1-5E88-96E7-201FBEC0722F; **Taxon:** scientificName: Anthidium (Anthidium) florentinum (Fabricius, 1775); order: Hymenoptera; family: Megachilidae; genus: Anthidium; subgenus: Anthidium; specificEpithet: florentinum; scientificNameAuthorship: (Fabricius, 1775); **Location:** country: Italy; countryCode: IT; stateProvince: Roma; locality: Riserva Naturale Valle dell'Aniene 2; decimalLatitude: 41.928752; decimalLongitude: 12.5562962; geodeticDatum: WGS84; coordinatePrecision: 0.0002; **Identification:** identifiedBy: M. Mei; **Event:** eventDate: 2022-06-05; **Record Level:** collectionCode: UR3
- **Type status:** Other material. **Occurrence:** catalogNumber: A0125; recordedBy: L. Fortini; individualCount: 1; sex: male; lifeStage: adult; occurrenceID: 8E999DC5-9999-5838-B6C0-592001DA045C; **Taxon:** scientificName: Anthidium (Anthidium) florentinum (Fabricius, 1775); order: Hymenoptera; family: Megachilidae; genus: Anthidium; subgenus: Anthidium; specificEpithet: florentinum; scientificNameAuthorship: (Fabricius, 1775); **Location:** country: Italy; countryCode: IT; stateProvince: Roma; locality: Riserva Naturale Valle dei Casali 1; decimalLatitude: 41.8710627; decimalLongitude: 12.4336809; geodeticDatum: WGS84; coordinatePrecision: 0.0002; **Identification:** identifiedBy: M. Mei; **Event:** eventDate: 2022-07-13; **Record Level:** collectionCode: UR3

#### Anthidium loti

Perris, 1852

7D5BE18D-5DBE-5BD4-9FDD-BC729ADDB542

##### Materials

- **Type status:** Other material. **Occurrence:** catalogNumber: A0156, A0158, A0161; recordedBy: L. Fortini; individualCount: 3; sex: 2 males, 1 female; lifeStage: adult; occurrenceID: 286403E1-B594-5C86-93AB-D94AF28308AA; **Taxon:** scientificName: Anthidium (Anthidium) loti Perris, 1852; order: Hymenoptera; family: Megachilidae; genus: Anthidium; subgenus: Anthidium; specificEpithet: loti; scientificNameAuthorship: Perris, 1852; **Location:** country: Italy; countryCode: IT; stateProvince: Roma; locality: Riserva Regionale dell'Appia Antica 1; decimalLatitude: 41.8623941; decimalLongitude: 12.524863; geodeticDatum: WGS84; coordinatePrecision: 0.0002; **Identification:** identifiedBy: M. Mei; **Event:** eventDate: 2022-06-12; **Record Level:** collectionCode: UR3
- **Type status:** Other material. **Occurrence:** catalogNumber: A0157, A0159; recordedBy: L. Fortini; individualCount: 2; sex: 1 male, 1 female; lifeStage: adult; occurrenceID: BE05B343-84CF-5AEB-B101-8ECC63DC3DCD; **Taxon:** scientificName: Anthidium (Anthidium) loti Perris, 1852; order: Hymenoptera; family: Megachilidae; genus: Anthidium; subgenus: Anthidium; specificEpithet: loti; scientificNameAuthorship: Perris, 1852; **Location:** country: Italy; countryCode: IT; stateProvince: Roma; locality: Riserva Naturale Valle dell'Aniene 1; decimalLatitude: 41.9345179; decimalLongitude: 12.5453096; geodeticDatum: WGS84; coordinatePrecision: 0.0002; **Identification:** identifiedBy: M. Mei; **Event:** eventDate: 2022-06-05; **Record Level:** collectionCode: UR3
- **Type status:** Other material. **Occurrence:** catalogNumber: A0160; recordedBy: L. Fortini; individualCount: 1; sex: female; lifeStage: adult; occurrenceID: C17B2DCC-817A-5FD0-B779-E0909F3C109C; **Taxon:** scientificName: Anthidium (Anthidium) loti Perris, 1852; order: Hymenoptera; family: Megachilidae; genus: Anthidium; subgenus: Anthidium; specificEpithet: loti; scientificNameAuthorship: Perris, 1852; **Location:** country: Italy; countryCode: IT; stateProvince: Roma; locality: Riserva Naturale Valle dell'Aniene 2; decimalLatitude: 41.928752; decimalLongitude: 12.5562962; geodeticDatum: WGS84; coordinatePrecision: 0.0002; **Identification:** identifiedBy: M. Mei; **Event:** eventDate: 2022-06-05; **Record Level:** collectionCode: UR3

#### Anthidium manicatum

(Linnaeus, 1758)

402A4BD7-2D62-5A9E-BF3C-DD39B7ABB277

##### Materials

- **Type status:** Other material. **Occurrence:** catalogNumber: A0138, A0139, A0140, A0141, A0143, A0145, A0146, A0147, A0148, A0149, A051, A0153, A0155; recordedBy: L. Fortini; individualCount: 13; sex: 10 males, 3 females; lifeStage: adult; occurrenceID: F74A99E2-6B72-59C0-8101-EFE9D9541A1F; **Taxon:** scientificName: Anthidium (Anthidium) manicatum (Linnaeus,1758); order: Hymenoptera; family: Megachilidae; genus: Anthidium; subgenus: Anthidium; specificEpithet: manicatum; scientificNameAuthorship: (Linnaeus,1758); **Location:** country: Italy; countryCode: IT; stateProvince: Roma; locality: Riserva Regionale dell'Appia Antica 1; decimalLatitude: 41.8623941; decimalLongitude: 12.524863; geodeticDatum: WGS84; coordinatePrecision: 0.0002; **Identification:** identifiedBy: M. Mei; **Event:** eventDate: 2022-06-12; **Record Level:** collectionCode: UR3
- **Type status:** Other material. **Occurrence:** catalogNumber: A0142, A0150, A0152, A0154; recordedBy: L. Fortini; individualCount: 4; sex: 2 males, 2 females; lifeStage: adult; occurrenceID: 3518ADFE-C662-5FE2-95FB-FB523F64BB50; **Taxon:** scientificName: Anthidium (Anthidium) manicatum (Linnaeus,1758); order: Hymenoptera; family: Megachilidae; genus: Anthidium; subgenus: Anthidium; specificEpithet: manicatum; scientificNameAuthorship: (Linnaeus,1758); **Location:** country: Italy; countryCode: IT; stateProvince: Roma; locality: Riserva Naturale Valle dell'Aniene 1; decimalLatitude: 41.9345179; decimalLongitude: 12.5453096; geodeticDatum: WGS84; coordinatePrecision: 0.0002; **Identification:** identifiedBy: M. Mei; **Event:** eventDate: 2022-06-05; **Record Level:** collectionCode: UR3
- **Type status:** Other material. **Occurrence:** catalogNumber: A0144; recordedBy: L. Fortini; individualCount: 1; sex: male; lifeStage: adult; occurrenceID: 8A931231-4EAC-5946-8A09-F53FEA674956; **Taxon:** scientificName: Anthidium (Anthidium) manicatum (Linnaeus,1758); order: Hymenoptera; family: Megachilidae; genus: Anthidium; subgenus: Anthidium; specificEpithet: manicatum; scientificNameAuthorship: (Linnaeus,1758); **Location:** country: Italy; countryCode: IT; stateProvince: Roma; locality: Riserva Naturale Valle dell'Aniene 1; decimalLatitude: 41.9345179; decimalLongitude: 12.5453096; geodeticDatum: WGS84; coordinatePrecision: 0.0002; **Identification:** identifiedBy: M. Mei; **Event:** eventDate: 2022-06-05; **Record Level:** collectionCode: UR3

#### Chelostoma campanularum

(Kirby, 1802)

18E83960-2D0B-592F-BE4E-39254477F36B

##### Materials

- **Type status:** Other material. **Occurrence:** catalogNumber: A0650, A0654; recordedBy: L. Fortini; individualCount: 2; sex: males; lifeStage: adult; occurrenceID: 261096EC-2C02-50A6-872B-F2BE0BAF9B36; **Taxon:** scientificName: Chelostoma (Foveosmia) campanularum (Kirby, 1802); order: Hymenoptera; family: Megachilidae; genus: Chelostoma; subgenus: Foveosmia; specificEpithet: campanularum; scientificNameAuthorship: (Kirby, 1802); **Location:** country: Italy; countryCode: IT; stateProvince: Roma; locality: Riserva Regionale dell'Appia Antica 3; decimalLatitude: 41.8298456; decimalLongitude: 12.5432538; geodeticDatum: WGS84; coordinatePrecision: 0.0002; **Identification:** identifiedBy: M. Mei; **Event:** eventDate: 2022-05-24; **Record Level:** collectionCode: UR3
- **Type status:** Other material. **Occurrence:** catalogNumber: A0655, A0656, A0657, A0951; recordedBy: L. Fortini; individualCount: 4; sex: 3 males, 1 female; lifeStage: adult; occurrenceID: 5C9A4C90-F4DB-5603-9C26-D33A662DEC17; **Taxon:** scientificName: Chelostoma (Foveosmia) campanularum (Kirby, 1802); order: Hymenoptera; family: Megachilidae; genus: Chelostoma; subgenus: Foveosmia; specificEpithet: campanularum; scientificNameAuthorship: (Kirby, 1802); **Location:** country: Italy; countryCode: IT; stateProvince: Roma; locality: Riserva Naturale Valle dei Casali 1; decimalLatitude: 41.8710627; decimalLongitude: 12.4336809; geodeticDatum: WGS84; coordinatePrecision: 0.0002; **Identification:** identifiedBy: M. Mei; **Event:** eventDate: 2022-05-14; **Record Level:** collectionCode: UR3

#### Chelostoma emarginatum

(Nylander, 1856)

20992DC6-BA31-54AB-962A-244A8FC24E0F

##### Materials

- **Type status:** Other material. **Occurrence:** catalogNumber: A0651, A0652; recordedBy: L. Fortini; individualCount: 2; sex: males; lifeStage: adult; occurrenceID: 73D71645-61F6-5A8D-B32F-AC029E5AF513; **Taxon:** scientificName: Chelostoma (Chelostoma) emarginatum (Nylander, 1856); order: Hymenoptera; family: Megachilidae; genus: Chelostoma; subgenus: Chelostoma; specificEpithet: emarginatum; scientificNameAuthorship: (Nylander, 1856); **Location:** country: Italy; countryCode: IT; stateProvince: Roma; locality: Riserva Naturale Valle dei Casali 1; decimalLatitude: 41.8710627; decimalLongitude: 12.4336809; geodeticDatum: WGS84; coordinatePrecision: 0.0002; **Identification:** identifiedBy: M. Mei; **Event:** eventDate: 2022-04-07; **Record Level:** collectionCode: UR3
- **Type status:** Other material. **Occurrence:** catalogNumber: A0653; recordedBy: L. Fortini; individualCount: 1; sex: female; lifeStage: adult; occurrenceID: A4C8366C-62CC-5775-B495-CD865F51EF61; **Taxon:** scientificName: Chelostoma (Chelostoma) emarginatum (Nylander, 1856); order: Hymenoptera; family: Megachilidae; genus: Chelostoma; subgenus: Chelostoma; specificEpithet: emarginatum; scientificNameAuthorship: (Nylander, 1856); **Location:** country: Italy; countryCode: IT; stateProvince: Roma; locality: Riserva Naturale Tenuta dei Massimi 2; decimalLatitude: 41.8316516; decimalLongitude: 12.3999927; geodeticDatum: WGS84; coordinatePrecision: 0.0002; **Identification:** identifiedBy: M. Mei; **Event:** eventDate: 2022-05-04; **Record Level:** collectionCode: UR3
- **Type status:** Other material. **Occurrence:** catalogNumber: A0684; recordedBy: L. Fortini; individualCount: 1; sex: male; lifeStage: adult; occurrenceID: 27E7F688-5C3C-589B-B386-8B98DE89FAA8; **Taxon:** scientificName: Chelostoma (Chelostoma) emarginatum (Nylander, 1856); order: Hymenoptera; family: Megachilidae; genus: Chelostoma; subgenus: Chelostoma; specificEpithet: emarginatum; scientificNameAuthorship: (Nylander, 1856); **Location:** country: Italy; countryCode: IT; stateProvince: Roma; locality: Riserva Naturale di Monte Mario; decimalLatitude: 41.9386215; decimalLongitude: 12.4546223; geodeticDatum: WGS84; coordinatePrecision: 0.0002; **Identification:** identifiedBy: M. Mei; **Event:** eventDate: 2022-04-20; **Record Level:** collectionCode: UR3
- **Type status:** Other material. **Occurrence:** catalogNumber: A0687; recordedBy: L. Fortini; individualCount: 1; sex: male; lifeStage: adult; occurrenceID: BF1688E1-92F9-5C8C-8143-B3B02E72531A; **Taxon:** scientificName: Chelostoma (Chelostoma) emarginatum (Nylander, 1856); order: Hymenoptera; family: Megachilidae; genus: Chelostoma; subgenus: Chelostoma; specificEpithet: emarginatum; scientificNameAuthorship: (Nylander, 1856); **Location:** country: Italy; countryCode: IT; stateProvince: Roma; locality: Riserva Naturale dell'Insugherata 3; decimalLatitude: 41.9644829; decimalLongitude: 12.436101; geodeticDatum: WGS84; coordinatePrecision: 0.0002; **Identification:** identifiedBy: M. Mei; **Event:** eventDate: 2022-04-15; **Record Level:** collectionCode: UR3

#### Chelostoma rapunculi

(Lepeletier, 1841)

51348DBB-610D-5985-9370-555F8072D59E

##### Materials

- **Type status:** Other material. **Occurrence:** catalogNumber: A0685; recordedBy: L. Fortini; individualCount: 1; sex: male; lifeStage: adult; occurrenceID: 09E28C78-E374-5589-B4F4-267AF60141F5; **Taxon:** scientificName: Chelostoma (Gyrodromella) rapunculi (Lepeletier, 1841); order: Hymenoptera; family: Megachilidae; genus: Chelostoma; subgenus: Gyrodromella; specificEpithet: rapunculi; scientificNameAuthorship: (Lepeletier, 1841); **Location:** country: Italy; countryCode: IT; stateProvince: Roma; locality: Riserva Regionale dell'Appia Antica 3; decimalLatitude: 41.8298456; decimalLongitude: 12.5432538; geodeticDatum: WGS84; coordinatePrecision: 0.0002; **Identification:** identifiedBy: M. Mei; **Event:** eventDate: 2022-05-24; **Record Level:** collectionCode: UR3
- **Type status:** Other material. **Occurrence:** catalogNumber: A0686; recordedBy: L. Fortini; individualCount: 1; sex: male; lifeStage: adult; occurrenceID: 7718F537-081F-5E4B-9F33-6C064C0EC0D8; **Taxon:** scientificName: Chelostoma (Gyrodromella) rapunculi (Lepeletier, 1841); order: Hymenoptera; family: Megachilidae; genus: Chelostoma; subgenus: Gyrodromella; specificEpithet: rapunculi; scientificNameAuthorship: (Lepeletier, 1841); **Location:** country: Italy; countryCode: IT; stateProvince: Roma; locality: Riserva Naturale Valle dei Casali 1; decimalLatitude: 41.8710627; decimalLongitude: 12.4336809; geodeticDatum: WGS84; coordinatePrecision: 0.0002; **Identification:** identifiedBy: M. Mei; **Event:** eventDate: 2022-05-14; **Record Level:** collectionCode: UR3
- **Type status:** Other material. **Occurrence:** catalogNumber: A0688; recordedBy: L. Fortini; individualCount: 1; sex: male; lifeStage: adult; occurrenceID: A10D3248-072A-5898-8E15-C93DFA1C0749; **Taxon:** scientificName: Chelostoma (Gyrodromella) rapunculi (Lepeletier, 1841); order: Hymenoptera; family: Megachilidae; genus: Chelostoma; subgenus: Gyrodromella; specificEpithet: rapunculi; scientificNameAuthorship: (Lepeletier, 1841); **Location:** country: Italy; countryCode: IT; stateProvince: Roma; locality: Riserva Naturale di Monte Mario; decimalLatitude: 41.9386215; decimalLongitude: 12.4546223; geodeticDatum: WGS84; coordinatePrecision: 0.0002; **Identification:** identifiedBy: M. Mei; **Event:** eventDate: 2022-05-20; **Record Level:** collectionCode: UR3

#### Coelioxys afer

Lepeletier, 1841

DD2E8CF2-88E5-524B-B018-7890190F5FEB

##### Materials

- **Type status:** Other material. **Occurrence:** catalogNumber: A0439; recordedBy: L. Fortini; individualCount: 1; sex: female; lifeStage: adult; occurrenceID: 41A29AA3-4433-5C28-A195-91B17C8C173E; **Taxon:** scientificName: Coelioxys (Austrocleptria) afer Lepeletier, 1841; order: Hymenoptera; family: Megachilidae; genus: Coelioxys; subgenus: Austrocleptria; specificEpithet: afra; scientificNameAuthorship: Lepeletier, 1841; **Location:** country: Italy; countryCode: IT; stateProvince: Roma; locality: Riserva Naturale Laurentino-Acqua Acetosa; decimalLatitude: 41.8079275; decimalLongitude: 12.4685548; geodeticDatum: WGS84; coordinatePrecision: 0.0002; **Identification:** identifiedBy: M. Mei; **Event:** eventDate: 2022-06-16; **Record Level:** collectionCode: UR3

#### Coelioxys aurolimbatus

Förster, 1853

A2BDC84F-1B8C-560F-AEF5-3DB80CC1EC07

##### Materials

- **Type status:** Other material. **Occurrence:** catalogNumber: A0438; recordedBy: L. Fortini; individualCount: 1; sex: male; lifeStage: adult; occurrenceID: 73BFB3F9-2573-5495-9672-BD44CCB0EB52; **Taxon:** scientificName: Coelioxys (Rozeniana) aurolimbatus Förster, 1853; order: Hymenoptera; family: Megachilidae; genus: Coelioxys; subgenus: Rozeniana; specificEpithet: aurolimbatus; scientificNameAuthorship: Förster, 1853; **Location:** country: Italy; countryCode: IT; stateProvince: Roma; locality: Riserva Naturale di Monte Mario; decimalLatitude: 41.9386215; decimalLongitude: 12.4546223; geodeticDatum: WGS84; coordinatePrecision: 0.0002; **Identification:** identifiedBy: M. Mei; **Event:** eventDate: 2022-09-12; **Record Level:** collectionCode: UR3

#### Coelioxys emarginatus

Förster, 1853

D3FE6EB3-AACC-5BEE-9F0C-688CA527B355

##### Materials

- **Type status:** Other material. **Occurrence:** catalogNumber: A0437; recordedBy: L. Fortini; individualCount: 1; sex: male; lifeStage: adult; occurrenceID: 213D00EE-C391-5E10-A3BF-4454B6B77144; **Taxon:** scientificName: Coelioxys (Allocoelioxys) emarginatus Förster, 1853; order: Hymenoptera; family: Megachilidae; genus: Coelioxys; subgenus: Allocoelioxys; specificEpithet: emarginata; scientificNameAuthorship: Förster, 1853; **Location:** country: Italy; countryCode: IT; stateProvince: Roma; locality: Riserva Naturale Valle dei Casali 1; decimalLatitude: 41.8710627; decimalLongitude: 12.4336809; geodeticDatum: WGS84; coordinatePrecision: 0.0002; **Identification:** identifiedBy: M. Mei; **Event:** eventDate: 2022-07-13; **Record Level:** collectionCode: UR3

#### Heriades crenulata

Nylander, 1856

F28F76A2-0380-5429-92D8-6E2B54D1DFFD

##### Materials

- **Type status:** Other material. **Occurrence:** catalogNumber: A0639; recordedBy: L. Fortini; individualCount: 1; sex: male; lifeStage: adult; occurrenceID: 0472EB44-8A76-5060-B759-821B9C462B36; **Taxon:** scientificName: Heriades (Heriades) crenulata Nylander, 1856; order: Hymenoptera; family: Megachilidae; genus: Heriades; subgenus: Heriades; specificEpithet: crenulata; scientificNameAuthorship: Nylander, 1856; **Location:** country: Italy; countryCode: IT; stateProvince: Roma; locality: Riserva Regionale dell'Appia Antica 1; decimalLatitude: 41.8623941; decimalLongitude: 12.524863; geodeticDatum: WGS84; coordinatePrecision: 0.0002; **Identification:** identifiedBy: M. Mei; **Event:** eventDate: 2022-06-12; **Record Level:** collectionCode: UR3
- **Type status:** Other material. **Occurrence:** catalogNumber: A0601, A0602, A0605; recordedBy: L. Fortini; individualCount: 3; sex: females; lifeStage: adult; occurrenceID: F87F2BBD-D702-523D-A46A-0242A0F3195C; **Taxon:** scientificName: Heriades (Heriades) crenulata Nylander, 1856; order: Hymenoptera; family: Megachilidae; genus: Heriades; subgenus: Heriades; specificEpithet: crenulata; scientificNameAuthorship: Nylander, 1856; **Location:** country: Italy; countryCode: IT; stateProvince: Roma; locality: Riserva Regionale dell'Appia Antica 2; decimalLatitude: 41.8402564; decimalLongitude: 12.532773; geodeticDatum: WGS84; coordinatePrecision: 0.0002; **Identification:** identifiedBy: M. Mei; **Event:** eventDate: 2022-08-29; **Record Level:** collectionCode: UR3
- **Type status:** Other material. **Occurrence:** catalogNumber: A0632, A0643; recordedBy: L. Fortini; individualCount: 2; sex: 1 male, 1 female; lifeStage: adult; occurrenceID: AD389911-8CC3-5972-8626-4B014031AF7A; **Taxon:** scientificName: Heriades (Heriades) crenulata Nylander, 1856; order: Hymenoptera; family: Megachilidae; genus: Heriades; subgenus: Heriades; specificEpithet: crenulata; scientificNameAuthorship: Nylander, 1856; **Location:** country: Italy; countryCode: IT; stateProvince: Roma; locality: Riserva Regionale dell'Appia Antica 2; decimalLatitude: 41.8402564; decimalLongitude: 12.532773; geodeticDatum: WGS84; coordinatePrecision: 0.0002; **Identification:** identifiedBy: M. Mei; **Event:** eventDate: 2022-07-07; **Record Level:** collectionCode: UR3
- **Type status:** Other material. **Occurrence:** catalogNumber: A0614, A0615, A0618, A0619, A0621, A0622, A0636, A0644, A0645; recordedBy: L. Fortini; individualCount: 9; sex: 3 males, 6 females; lifeStage: adult; occurrenceID: 8BF7278A-7F6B-5B48-B622-FB001B478711; **Taxon:** scientificName: Heriades (Heriades) crenulata Nylander, 1856; order: Hymenoptera; family: Megachilidae; genus: Heriades; subgenus: Heriades; specificEpithet: crenulata; scientificNameAuthorship: Nylander, 1856; **Location:** country: Italy; countryCode: IT; stateProvince: Roma; locality: Riserva Naturale dell'Insugherata 1; decimalLatitude: 41.9555045; decimalLongitude: 12.4292321; geodeticDatum: WGS84; coordinatePrecision: 0.0002; **Identification:** identifiedBy: M. Mei; **Event:** eventDate: 2022-07-30; **Record Level:** collectionCode: UR3
- **Type status:** Other material. **Occurrence:** catalogNumber: A0626; recordedBy: L. Fortini; individualCount: 1; sex: female; lifeStage: adult; occurrenceID: 270145BD-E26D-5CB1-BBA0-B95B1501E29F; **Taxon:** scientificName: Heriades (Heriades) crenulata Nylander, 1856; order: Hymenoptera; family: Megachilidae; genus: Heriades; subgenus: Heriades; specificEpithet: crenulata; scientificNameAuthorship: Nylander, 1856; **Location:** country: Italy; countryCode: IT; stateProvince: Roma; locality: Riserva Naturale dell'Insugherata 2; decimalLatitude: 41.9599247; decimalLongitude: 12.433852; geodeticDatum: WGS84; coordinatePrecision: 0.0002; **Identification:** identifiedBy: M. Mei; **Event:** eventDate: 2022-05-27; **Record Level:** collectionCode: UR3
- **Type status:** Other material. **Occurrence:** catalogNumber: A0631; recordedBy: L. Fortini; individualCount: 1; sex: male; lifeStage: adult; occurrenceID: FB714273-B35C-5C07-8626-F1BACF178625; **Taxon:** scientificName: Heriades (Heriades) crenulata Nylander, 1856; order: Hymenoptera; family: Megachilidae; genus: Heriades; subgenus: Heriades; specificEpithet: crenulata; scientificNameAuthorship: Nylander, 1856; **Location:** country: Italy; countryCode: IT; stateProvince: Roma; locality: Riserva Naturale di Monte Mario; decimalLatitude: 41.9386215; decimalLongitude: 12.4546223; geodeticDatum: WGS84; coordinatePrecision: 0.0002; **Identification:** identifiedBy: M. Mei; **Event:** eventDate: 2022-06-19; **Record Level:** collectionCode: UR3
- **Type status:** Other material. **Occurrence:** catalogNumber: A0633; recordedBy: L. Fortini; individualCount: 1; sex: female; lifeStage: adult; occurrenceID: B25DF1F3-3F43-5A66-B097-BC8C4F84F8B5; **Taxon:** scientificName: Heriades (Heriades) crenulata Nylander, 1856; order: Hymenoptera; family: Megachilidae; genus: Heriades; subgenus: Heriades; specificEpithet: crenulata; scientificNameAuthorship: Nylander, 1856; **Location:** country: Italy; countryCode: IT; stateProvince: Roma; locality: Riserva Naturale di Monte Mario; decimalLatitude: 41.9386215; decimalLongitude: 12.4546223; geodeticDatum: WGS84; coordinatePrecision: 0.0002; **Identification:** identifiedBy: M. Mei; **Event:** eventDate: 2022-07-24; **Record Level:** collectionCode: UR3
- **Type status:** Other material. **Occurrence:** catalogNumber: A0635; recordedBy: L. Fortini; individualCount: 1; sex: male; lifeStage: adult; occurrenceID: 37F68642-ABDE-5E98-AF60-770ADB96FCE3; **Taxon:** scientificName: Heriades (Heriades) crenulata Nylander, 1856; order: Hymenoptera; family: Megachilidae; genus: Heriades; subgenus: Heriades; specificEpithet: crenulata; scientificNameAuthorship: Nylander, 1856; **Location:** country: Italy; countryCode: IT; stateProvince: Roma; locality: Riserva Naturale Valle dei Casali 1; decimalLatitude: 41.8710627; decimalLongitude: 12.4336809; geodeticDatum: WGS84; coordinatePrecision: 0.0002; **Identification:** identifiedBy: M. Mei; **Event:** eventDate: 2022-07-13; **Record Level:** collectionCode: UR3
- **Type status:** Other material. **Occurrence:** catalogNumber: A0628, A0629, A0630, A0640, A0641; recordedBy: L. Fortini; individualCount: 5; sex: 1 male, 4 females; lifeStage: adult; occurrenceID: A63E2924-1989-5AC0-BED4-AFCB013C57CD; **Taxon:** scientificName: Heriades (Heriades) crenulata Nylander, 1856; order: Hymenoptera; family: Megachilidae; genus: Heriades; subgenus: Heriades; specificEpithet: crenulata; scientificNameAuthorship: Nylander, 1856; **Location:** country: Italy; countryCode: IT; stateProvince: Roma; locality: Riserva Naturale Valle dei Casali 1; decimalLatitude: 41.8710627; decimalLongitude: 12.4336809; geodeticDatum: WGS84; coordinatePrecision: 0.0002; **Identification:** identifiedBy: M. Mei; **Event:** eventDate: 2022-06-18; **Record Level:** collectionCode: UR3
- **Type status:** Other material. **Occurrence:** catalogNumber: A2131, A2132; recordedBy: L. Fortini; individualCount: 2; sex: males; lifeStage: adult; occurrenceID: 8FE8EA1B-1A57-5AA8-814C-8E8FD44B4AC9; **Taxon:** scientificName: Heriades (Heriades) crenulata Nylander, 1856; order: Hymenoptera; family: Megachilidae; genus: Heriades; subgenus: Heriades; specificEpithet: crenulata; scientificNameAuthorship: Nylander, 1856; **Location:** country: Italy; countryCode: IT; stateProvince: Roma; locality: Riserva Naturale Valle dell'Aniene 2; decimalLatitude: 41.928752; decimalLongitude: 12.5562962; geodeticDatum: WGS84; coordinatePrecision: 0.0002; **Identification:** identifiedBy: M. Mei; **Event:** eventDate: 2022-08-03; **Record Level:** collectionCode: UR3

#### Heriades rubicola

Pérez, 1890

CA2FAB1A-8FDA-5501-9683-97ABAEA5DC24

##### Materials

- **Type status:** Other material. **Occurrence:** catalogNumber: A0638; recordedBy: L. Fortini; individualCount: 1; sex: male; lifeStage: adult; occurrenceID: AF5E8C69-4337-539C-AB5C-D9EA21A4D18D; **Taxon:** scientificName: Heriades (Heriades) rubicola Pérez, 1890; order: Hymenoptera; family: Megachilidae; genus: Heriades; subgenus: Heriades; specificEpithet: rubicola; scientificNameAuthorship: Pérez, 1890; **Location:** country: Italy; countryCode: IT; stateProvince: Roma; locality: Riserva Naturale dell'Acquafredda; decimalLatitude: 41.8928408; decimalLongitude: 12.39932; geodeticDatum: WGS84; coordinatePrecision: 0.0002; **Identification:** identifiedBy: M. Mei; **Event:** eventDate: 2022-06-10; **Record Level:** collectionCode: UR3
- **Type status:** Other material. **Occurrence:** catalogNumber: A0623; recordedBy: L. Fortini; individualCount: 1; sex: female; lifeStage: adult; occurrenceID: 0B289D5A-DFC1-5B33-8BA6-E74EE3A4DB04; **Taxon:** scientificName: Heriades (Heriades) rubicola Pérez, 1890; order: Hymenoptera; family: Megachilidae; genus: Heriades; subgenus: Heriades; specificEpithet: rubicola; scientificNameAuthorship: Pérez, 1890; **Location:** country: Italy; countryCode: IT; stateProvince: Roma; locality: Riserva Regionale dell'Appia Antica 1; decimalLatitude: 41.8623941; decimalLongitude: 12.524863; geodeticDatum: WGS84; coordinatePrecision: 0.0002; **Identification:** identifiedBy: M. Mei; **Event:** eventDate: 2022-07-11; **Record Level:** collectionCode: UR3
- **Type status:** Other material. **Occurrence:** catalogNumber: A0603, A0604, A0606, A0607, A0608, A0609, A0610, A0611, A0611, A0612; recordedBy: L. Fortini; individualCount: 9; sex: females; lifeStage: adult; occurrenceID: 110A6BAD-55E2-52F6-85FA-CC1396307410; **Taxon:** scientificName: Heriades (Heriades) rubicola Pérez, 1890; order: Hymenoptera; family: Megachilidae; genus: Heriades; subgenus: Heriades; specificEpithet: rubicola; scientificNameAuthorship: Pérez, 1890; **Location:** country: Italy; countryCode: IT; stateProvince: Roma; locality: Riserva Regionale dell'Appia Antica 2; decimalLatitude: 41.8402564; decimalLongitude: 12.532773; geodeticDatum: WGS84; coordinatePrecision: 0.0002; **Identification:** identifiedBy: M. Mei; **Event:** eventDate: 2022-08-29; **Record Level:** collectionCode: UR3
- **Type status:** Other material. **Occurrence:** catalogNumber: A0613, A0620, A0634, A0646; recordedBy: L. Fortini; individualCount: 4; sex: 3 males, 1 female; lifeStage: adult; occurrenceID: 6B2BB613-D4B5-529F-A87D-F061A087AC79; **Taxon:** scientificName: Heriades (Heriades) rubicola Pérez, 1890; order: Hymenoptera; family: Megachilidae; genus: Heriades; subgenus: Heriades; specificEpithet: rubicola; scientificNameAuthorship: Pérez, 1890; **Location:** country: Italy; countryCode: IT; stateProvince: Roma; locality: Riserva Naturale dell'Insugherata 1; decimalLatitude: 41.9555045; decimalLongitude: 12.4292321; geodeticDatum: WGS84; coordinatePrecision: 0.0002; **Identification:** identifiedBy: M. Mei; **Event:** eventDate: 2022-07-30; **Record Level:** collectionCode: UR3
- **Type status:** Other material. **Occurrence:** catalogNumber: A0625, A0627; recordedBy: L. Fortini; individualCount: 2; sex: males; lifeStage: adult; occurrenceID: 55D18BAF-5453-532E-B859-B9AAB6E0E697; **Taxon:** scientificName: Heriades (Heriades) rubicola Pérez, 1890; order: Hymenoptera; family: Megachilidae; genus: Heriades; subgenus: Heriades; specificEpithet: rubicola; scientificNameAuthorship: Pérez, 1890; **Location:** country: Italy; countryCode: IT; stateProvince: Roma; locality: Riserva Naturale dell'Insugherata 2; decimalLatitude: 41.9599247; decimalLongitude: 12.433852; geodeticDatum: WGS84; coordinatePrecision: 0.0002; **Identification:** identifiedBy: M. Mei; **Event:** eventDate: 2022-05-27; **Record Level:** collectionCode: UR3
- **Type status:** Other material. **Occurrence:** catalogNumber: A0616, A0617, A0624; recordedBy: L. Fortini; individualCount: 3; sex: females; lifeStage: adult; occurrenceID: B7051F44-BFE8-5C18-8FBC-2A02D750D710; **Taxon:** scientificName: Heriades (Heriades) rubicola Pérez, 1890; order: Hymenoptera; family: Megachilidae; genus: Heriades; subgenus: Heriades; specificEpithet: rubicola; scientificNameAuthorship: Pérez, 1890; **Location:** country: Italy; countryCode: IT; stateProvince: Roma; locality: Riserva Naturale Valle dell'Aniene 2; decimalLatitude: 41.928752; decimalLongitude: 12.5562962; geodeticDatum: WGS84; coordinatePrecision: 0.0002; **Identification:** identifiedBy: M. Mei; **Event:** eventDate: 2022-09-04; **Record Level:** collectionCode: UR3

#### Heriades truncorum

(Linnaeus, 1758)

6F6EE690-C137-5A25-8BFA-6D4CD769BFA4

##### Materials

- **Type status:** Other material. **Occurrence:** catalogNumber: A0642; recordedBy: L. Fortini; individualCount: 1; sex: female; lifeStage: adult; occurrenceID: B5195795-C4CC-5179-97F5-4FB057E1D4F8; **Taxon:** scientificName: Heriades (Heriades) truncorum (Linnaeus, 1758); order: Hymenoptera; family: Megachilidae; genus: Heriades; subgenus: Heriades; specificEpithet: truncorum; scientificNameAuthorship: (Linnaeus, 1758); **Location:** country: Italy; countryCode: IT; stateProvince: Roma; locality: Riserva Naturale di Monte Mario; decimalLatitude: 41.9386215; decimalLongitude: 12.4546223; geodeticDatum: WGS84; coordinatePrecision: 0.0002; **Identification:** identifiedBy: M. Mei; **Event:** eventDate: 2022-06-19; **Record Level:** collectionCode: UR3

#### Hoplitis adunca

(Panzer, 1798)

785E4BBD-8BBC-5A01-8B80-15A92149AB02

##### Materials

- **Type status:** Other material. **Occurrence:** catalogNumber: A0450, A0451; recordedBy: L. Fortini; individualCount: 2; sex: females; lifeStage: adult; occurrenceID: 31137F4D-B702-5D72-8500-4F180D7A161C; **Taxon:** scientificName: Hoplitis (Hoplitis) adunca (Panzer, 1798); order: Hymenoptera; family: Megachilidae; genus: Hoplitis; subgenus: Hoplitis; specificEpithet: adunca; scientificNameAuthorship: (Panzer, 1798); **Location:** country: Italy; countryCode: IT; stateProvince: Roma; locality: Riserva Regionale dell'Appia Antica 2; decimalLatitude: 41.8402564; decimalLongitude: 12.532773; geodeticDatum: WGS84; coordinatePrecision: 0.0002; **Identification:** identifiedBy: M. Mei; **Event:** eventDate: 2022-05-24; **Record Level:** collectionCode: UR3
- **Type status:** Other material. **Occurrence:** catalogNumber: A0690; recordedBy: L. Fortini; individualCount: 1; sex: male; lifeStage: adult; occurrenceID: ADAAB9EA-55FB-5D07-A8FA-450E561EB445; **Taxon:** scientificName: Hoplitis (Hoplitis) adunca (Panzer, 1798); order: Hymenoptera; family: Megachilidae; genus: Hoplitis; subgenus: Hoplitis; specificEpithet: adunca; scientificNameAuthorship: (Panzer, 1798); **Location:** country: Italy; countryCode: IT; stateProvince: Roma; locality: Riserva Naturale dell'Insugherata 2; decimalLatitude: 41.9599247; decimalLongitude: 12.433852; geodeticDatum: WGS84; coordinatePrecision: 0.0002; **Identification:** identifiedBy: M. Mei; **Event:** eventDate: 2022-05-27; **Record Level:** collectionCode: UR3
- **Type status:** Other material. **Occurrence:** catalogNumber: A0691; recordedBy: L. Fortini; individualCount: 1; sex: male; lifeStage: adult; occurrenceID: AD79FEF1-996A-523E-840C-1EB082B16D63; **Taxon:** scientificName: Hoplitis (Hoplitis) adunca (Panzer, 1798); order: Hymenoptera; family: Megachilidae; genus: Hoplitis; subgenus: Hoplitis; specificEpithet: adunca; scientificNameAuthorship: (Panzer, 1798); **Location:** country: Italy; countryCode: IT; stateProvince: Roma; locality: Riserva Regionale dell'Appia Antica 1; decimalLatitude: 41.8623941; decimalLongitude: 12.524863; geodeticDatum: WGS84; coordinatePrecision: 0.0002; **Identification:** identifiedBy: M. Mei; **Event:** eventDate: 2022-06-12; **Record Level:** collectionCode: UR3

#### Hoplitis anthocopoides

(Schenck, 1853)

57804CD8-D2A9-53EC-8856-A2C17EEF5942

##### Materials

- **Type status:** Other material. **Occurrence:** catalogNumber: A0692; recordedBy: L. Fortini; individualCount: 1; sex: male; lifeStage: adult; occurrenceID: E8A13865-AA0B-5CD2-AFA4-A0ECCD184A83; **Taxon:** scientificName: Hoplitis (Hoplitis) anthocopoides (Schenck, 1853); order: Hymenoptera; family: Megachilidae; genus: Hoplitis; subgenus: Hoplitis; specificEpithet: anthocopoides; scientificNameAuthorship: (Schenck, 1853); **Location:** country: Italy; countryCode: IT; stateProvince: Roma; locality: Riserva Regionale dell'Appia Antica 3; decimalLatitude: 41.8298456; decimalLongitude: 12.5432538; geodeticDatum: WGS84; coordinatePrecision: 0.0002; **Identification:** identifiedBy: M. Mei; **Event:** eventDate: 2022-05-24; **Record Level:** collectionCode: UR3

#### Hoplitis bisulca

(Gerstaecker, 1869)

E5EC0C52-5952-56F1-A85E-1B39CA2478C5

##### Materials

- **Type status:** Other material. **Occurrence:** catalogNumber: A0449; recordedBy: L. Fortini; individualCount: 1; sex: female; lifeStage: adult; occurrenceID: 00DAEC0F-2A88-5175-BFEE-B25E02FFB29D; **Taxon:** scientificName: Hoplitis (Anthocopa) bisulca (Gerstäcker, 1869); order: Hymenoptera; family: Megachilidae; genus: Hoplitis; subgenus: Anthocopa; specificEpithet: bisulca; scientificNameAuthorship: (Gerstäcker, 1869); **Location:** country: Italy; countryCode: IT; stateProvince: Roma; locality: Riserva Naturale Tenuta dei Massimi 2; decimalLatitude: 41.8316516; decimalLongitude: 12.3999927; geodeticDatum: WGS84; coordinatePrecision: 0.0002; **Identification:** identifiedBy: M. Mei; **Event:** eventDate: 2022-06-01; **Record Level:** collectionCode: UR3
- **Type status:** Other material. **Occurrence:** catalogNumber: A0658; recordedBy: L. Fortini; individualCount: 1; sex: male; lifeStage: adult; occurrenceID: B26CA098-F4D5-56DD-B490-8DC743FCF20A; **Taxon:** scientificName: Hoplitis (Anthocopa) bisulca (Gerstäcker, 1869); order: Hymenoptera; family: Megachilidae; genus: Hoplitis; subgenus: Anthocopa; specificEpithet: bisulca; scientificNameAuthorship: (Gerstäcker, 1869); **Location:** country: Italy; countryCode: IT; stateProvince: Roma; locality: Riserva Naturale Tenuta dei Massimi 2; decimalLatitude: 41.8316516; decimalLongitude: 12.3999927; geodeticDatum: WGS84; coordinatePrecision: 0.0002; **Identification:** identifiedBy: M. Mei; **Event:** eventDate: 2022-06-27; **Record Level:** collectionCode: UR3

#### Hoplitis cristatula

(var der Zanden, 1990)

BB2A6186-D44A-579A-9F6D-306E4CA59E14

##### Materials

- **Type status:** Other material. **Occurrence:** catalogNumber: A0448; recordedBy: L. Fortini; individualCount: 1; sex: female; lifeStage: adult; occurrenceID: EC521FB3-90C6-5141-91DC-3D2E137173AA; **Taxon:** scientificName: Hoplitis (Anthocopa) cristatula (van der Zanden, 1990); order: Hymenoptera; family: Megachilidae; genus: Hoplitis; subgenus: Anthocopa; specificEpithet: cristatula; scientificNameAuthorship: (van der Zanden, 1990); **Location:** country: Italy; countryCode: IT; stateProvince: Roma; locality: Riserva Regionale dell'Appia Antica 1; decimalLatitude: 41.8623941; decimalLongitude: 12.524863; geodeticDatum: WGS84; coordinatePrecision: 0.0002; **Identification:** identifiedBy: M. Mei; **Event:** eventDate: 2022-06-12; **Record Level:** collectionCode: UR3
- **Type status:** Other material. **Occurrence:** catalogNumber: A0689; recordedBy: L. Fortini; individualCount: 1; sex: male; lifeStage: adult; occurrenceID: 70F0A75D-2FA0-5364-86E0-07BF483FCA47; **Taxon:** scientificName: Hoplitis (Anthocopa) cristatula (van der Zanden, 1990); order: Hymenoptera; family: Megachilidae; genus: Hoplitis; subgenus: Anthocopa; specificEpithet: cristatula; scientificNameAuthorship: (van der Zanden, 1990); **Location:** country: Italy; countryCode: IT; stateProvince: Roma; locality: Riserva Regionale dell'Appia Antica 2; decimalLatitude: 41.8402564; decimalLongitude: 12.532773; geodeticDatum: WGS84; coordinatePrecision: 0.0002; **Identification:** identifiedBy: M. Mei; **Event:** eventDate: 2022-05-24; **Record Level:** collectionCode: UR3
- **Type status:** Other material. **Occurrence:** catalogNumber: A0452; recordedBy: L. Fortini; individualCount: 1; sex: male; lifeStage: adult; occurrenceID: 28E165C3-9F08-58F5-BB26-BEEBFE3970F4; **Taxon:** scientificName: Hoplitis (Anthocopa) cristatula (van der Zanden, 1990); order: Hymenoptera; family: Megachilidae; genus: Hoplitis; subgenus: Anthocopa; specificEpithet: cristatula; scientificNameAuthorship: (van der Zanden, 1990); **Location:** country: Italy; countryCode: IT; stateProvince: Roma; locality: Riserva Naturale Laurentino-Acqua Acetosa; decimalLatitude: 41.8079275; decimalLongitude: 12.4685548; geodeticDatum: WGS84; coordinatePrecision: 0.0002; **Identification:** identifiedBy: M. Mei; **Event:** eventDate: 2022-06-16; **Record Level:** collectionCode: UR3
- **Type status:** Other material. **Occurrence:** catalogNumber: A0445, A0446, A0447, A0453; recordedBy: L. Fortini; individualCount: 4; sex: 1 male, 3 females; lifeStage: adult; occurrenceID: B88BB7EA-5102-5BE7-B0DA-1DE58F7542B5; **Taxon:** scientificName: Hoplitis (Anthocopa) cristatula (van der Zanden, 1990); order: Hymenoptera; family: Megachilidae; genus: Hoplitis; subgenus: Anthocopa; specificEpithet: cristatula; scientificNameAuthorship: (van der Zanden, 1990); **Location:** country: Italy; countryCode: IT; stateProvince: Roma; locality: Riserva Naturale Valle dell'Aniene 2; decimalLatitude: 41.928752; decimalLongitude: 12.5562962; geodeticDatum: WGS84; coordinatePrecision: 0.0002; **Identification:** identifiedBy: M. Mei; **Event:** eventDate: 2022-06-05; **Record Level:** collectionCode: UR3

#### Hoplitis tridentata

(Dufour & Perris, 1840)

F68194CF-B034-57EF-84F2-52ADC3004AA6

##### Materials

- **Type status:** Other material. **Occurrence:** catalogNumber: A1166; recordedBy: L. Fortini; individualCount: 1; sex: female; lifeStage: adult; occurrenceID: 3ED66678-C5AB-5034-AC2C-701A3DCC0F3A; **Taxon:** scientificName: Hoplitis (Alcidamea) tridentata (Dufour & Perris, 1840); order: Hymenoptera; family: Megachilidae; genus: Hoplitis; subgenus: Alcidamea; specificEpithet: tridentata; scientificNameAuthorship: (Dufour & Perris, 1840); **Location:** country: Italy; countryCode: IT; stateProvince: Roma; locality: Riserva Regionale dell'Appia Antica 1; decimalLatitude: 41.8623941; decimalLongitude: 12.524863; geodeticDatum: WGS84; coordinatePrecision: 0.0002; **Identification:** identifiedBy: M. Mei; **Event:** eventDate: 2022-06-12; **Record Level:** collectionCode: UR3

#### Icteranthidium grohmanni

(Spinola, 1838)

D4F3793B-9A4E-5B34-9100-AA129C72BADF

##### Materials

- **Type status:** Other material. **Occurrence:** catalogNumber: A0179; recordedBy: L. Fortini; individualCount: 1; sex: female; lifeStage: adult; occurrenceID: DA901621-887D-5852-891C-19D27F70118B; **Taxon:** scientificName: Icteranthidium (Icteranthidium) grohmanni (Spinola 1838); order: Hymenoptera; family: Megachilidae; genus: Icteranthidium; subgenus: Icteranthidium; specificEpithet: grohmanni; scientificNameAuthorship: (Spinola 1838); **Location:** country: Italy; countryCode: IT; stateProvince: Roma; locality: Riserva Regionale dell'Appia Antica 1; decimalLatitude: 41.8623941; decimalLongitude: 12.524863; geodeticDatum: WGS84; coordinatePrecision: 0.0002; **Identification:** identifiedBy: M. Mei; **Event:** eventDate: 2022-06-12; **Record Level:** collectionCode: UR3

#### Lithurgus chrysurus

Fonscolombe, 1834

095FC465-2C81-50A8-9362-F47804EEBE8D

##### Materials

- **Type status:** Other material. **Occurrence:** catalogNumber: A1188, A1189, A1190, A1191, A1193, A1194, A1195; recordedBy: L. Fortini; individualCount: 7; sex: females; lifeStage: adult; occurrenceID: 779D2673-DFC1-5785-8AE2-BD36A9E7BA73; **Taxon:** scientificName: Lithurgus (Lithurgus) chrysurus Fonscolombe, 1834; order: Hymenoptera; family: Megachilidae; genus: Lithurgus; subgenus: Lithurgus; specificEpithet: chrysurus; scientificNameAuthorship: Fonscolombe, 1834; **Location:** country: Italy; countryCode: IT; stateProvince: Roma; locality: Riserva Naturale dell'Insugherata 1; decimalLatitude: 41.9555045; decimalLongitude: 12.4292321; geodeticDatum: WGS84; coordinatePrecision: 0.0002; **Identification:** identifiedBy: M. Mei; **Event:** eventDate: 2022-07-30; **Record Level:** collectionCode: UR3
- **Type status:** Other material. **Occurrence:** catalogNumber: A1196; recordedBy: L. Fortini; individualCount: 1; sex: male; lifeStage: adult; occurrenceID: 64A6C69D-95E6-5242-8421-7B59AEFDC616; **Taxon:** scientificName: Lithurgus (Lithurgus) chrysurus Fonscolombe, 1834; order: Hymenoptera; family: Megachilidae; genus: Lithurgus; subgenus: Lithurgus; specificEpithet: chrysurus; scientificNameAuthorship: Fonscolombe, 1834; **Location:** country: Italy; countryCode: IT; stateProvince: Roma; locality: Riserva Naturale dell'Insugherata 1; decimalLatitude: 41.9555045; decimalLongitude: 12.4292321; geodeticDatum: WGS84; coordinatePrecision: 0.0002; **Identification:** identifiedBy: M. Mei; **Event:** eventDate: 2022-06-24; **Record Level:** collectionCode: UR3
- **Type status:** Other material. **Occurrence:** catalogNumber: A1192, A1197; recordedBy: L. Fortini; individualCount: 2; sex: 1 male, 1 female; lifeStage: adult; occurrenceID: 11E8EBBF-F2FF-500D-A973-640C0918123F; **Taxon:** scientificName: Lithurgus (Lithurgus) chrysurus Fonscolombe, 1834; order: Hymenoptera; family: Megachilidae; genus: Lithurgus; subgenus: Lithurgus; specificEpithet: chrysurus; scientificNameAuthorship: Fonscolombe, 1834; **Location:** country: Italy; countryCode: IT; stateProvince: Roma; locality: Riserva Naturale Valle dei Casali 1; decimalLatitude: 41.8710627; decimalLongitude: 12.4336809; geodeticDatum: WGS84; coordinatePrecision: 0.0002; **Identification:** identifiedBy: M. Mei; **Event:** eventDate: 2022-06-18; **Record Level:** collectionCode: UR3
- **Type status:** Other material. **Occurrence:** catalogNumber: A2133; recordedBy: L. Fortini; individualCount: 1; sex: female; lifeStage: adult; occurrenceID: 47FDBA6F-FAE1-59E2-8F41-B866AC3C27FA; **Taxon:** scientificName: Lithurgus (Lithurgus) chrysurus Fonscolombe, 1834; order: Hymenoptera; family: Megachilidae; genus: Lithurgus; subgenus: Lithurgus; specificEpithet: chrysurus; scientificNameAuthorship: Fonscolombe, 1834; **Location:** country: Italy; countryCode: IT; stateProvince: Roma; locality: Riserva Naturale Valle dell'Aniene 2; decimalLatitude: 41.928752; decimalLongitude: 12.5562962; geodeticDatum: WGS84; coordinatePrecision: 0.0002; **Identification:** identifiedBy: M. Mei; **Event:** eventDate: 2022-08-03; **Record Level:** collectionCode: UR3

#### Megachile albisecta

(Klug, 1817)

45A75C0F-27E0-5AC9-8A33-4173ABD8CC54

##### Materials

- **Type status:** Other material. **Occurrence:** catalogNumber: A1114; recordedBy: L. Fortini; individualCount: 1; sex: male; lifeStage: adult; occurrenceID: 1C39BE5C-D287-5F60-B183-7C7AD036A874; **Taxon:** scientificName: Megachile (Creightonella) albisecta (Klug, 1817); order: Hymenoptera; family: Megachilidae; genus: Megachile; subgenus: Creightonella; specificEpithet: albisecta; scientificNameAuthorship: (Klug, 1817); **Location:** country: Italy; countryCode: IT; stateProvince: Roma; locality: Riserva Naturale dell'Insugherata 2; decimalLatitude: 41.9599247; decimalLongitude: 12.433852; geodeticDatum: WGS84; coordinatePrecision: 0.0002; **Identification:** identifiedBy: M. Mei; **Event:** eventDate: 2022-07-30; **Record Level:** collectionCode: UR3
- **Type status:** Other material. **Occurrence:** catalogNumber: A1136; recordedBy: L. Fortini; individualCount: 1; sex: female; lifeStage: adult; occurrenceID: 436A26C1-3DA4-5DDE-88B3-99C7FFAA894D; **Taxon:** scientificName: Megachile (Creightonella) albisecta (Klug, 1817); order: Hymenoptera; family: Megachilidae; genus: Megachile; subgenus: Creightonella; specificEpithet: albisecta; scientificNameAuthorship: (Klug, 1817); **Location:** country: Italy; countryCode: IT; stateProvince: Roma; locality: Riserva Naturale dell'Insugherata 1; decimalLatitude: 41.9555045; decimalLongitude: 12.4292321; geodeticDatum: WGS84; coordinatePrecision: 0.0002; **Identification:** identifiedBy: M. Mei; **Event:** eventDate: 2022-06-24; **Record Level:** collectionCode: UR3
- **Type status:** Other material. **Occurrence:** catalogNumber: A1118; recordedBy: L. Fortini; individualCount: 1; sex: female; lifeStage: adult; occurrenceID: A3135BC5-A795-575B-AF21-0C79F1D86538; **Taxon:** scientificName: Megachile (Creightonella) albisecta (Klug, 1817); order: Hymenoptera; family: Megachilidae; genus: Megachile; subgenus: Creightonella; specificEpithet: albisecta; scientificNameAuthorship: (Klug, 1817); **Location:** country: Italy; countryCode: IT; stateProvince: Roma; locality: Riserva Naturale di Monte Mario; decimalLatitude: 41.9386215; decimalLongitude: 12.4546223; geodeticDatum: WGS84; coordinatePrecision: 0.0002; **Identification:** identifiedBy: M. Mei; **Event:** eventDate: 2022-07-19; **Record Level:** collectionCode: UR3
- **Type status:** Other material. **Occurrence:** catalogNumber: A1115, A1117, A1138, A1139, A1140, A1141, A1142, A1144; recordedBy: L. Fortini; individualCount: 8; sex: 2 males, 6 females; lifeStage: adult; occurrenceID: 396BB123-F878-5991-A72B-B214FB4DD402; **Taxon:** scientificName: Megachile (Creightonella) albisecta (Klug, 1817); order: Hymenoptera; family: Megachilidae; genus: Megachile; subgenus: Creightonella; specificEpithet: albisecta; scientificNameAuthorship: (Klug, 1817); **Location:** country: Italy; countryCode: IT; stateProvince: Roma; locality: Riserva Naturale Valle dell'Aniene 1; decimalLatitude: 41.9345179; decimalLongitude: 12.5453096; geodeticDatum: WGS84; coordinatePrecision: 0.0002; **Identification:** identifiedBy: M. Mei; **Event:** eventDate: 2022-07-01; **Record Level:** collectionCode: UR3
- **Type status:** Other material. **Occurrence:** catalogNumber: A1135, A1137, A1143; recordedBy: L. Fortini; individualCount: 3; sex: females; lifeStage: adult; occurrenceID: 1BC9033E-11AC-5B9E-854B-F59821B0BA37; **Taxon:** scientificName: Megachile (Creightonella) albisecta (Klug, 1817); order: Hymenoptera; family: Megachilidae; genus: Megachile; subgenus: Creightonella; specificEpithet: albisecta; scientificNameAuthorship: (Klug, 1817); **Location:** country: Italy; countryCode: IT; stateProvince: Roma; locality: Riserva Naturale Valle dell'Aniene 2; decimalLatitude: 41.928752; decimalLongitude: 12.5562962; geodeticDatum: WGS84; coordinatePrecision: 0.0002; **Identification:** identifiedBy: M. Mei; **Event:** eventDate: 2022-07-01; **Record Level:** collectionCode: UR3
- **Type status:** Other material. **Occurrence:** catalogNumber: A1113, A1116, A1134; recordedBy: L. Fortini; individualCount: 3; sex: 2 males, 1 female; lifeStage: adult; occurrenceID: F4C058EA-BCBA-5010-8B65-152DCDB399C5; **Taxon:** scientificName: Megachile (Creightonella) albisecta (Klug, 1817); order: Hymenoptera; family: Megachilidae; genus: Megachile; subgenus: Creightonella; specificEpithet: albisecta; scientificNameAuthorship: (Klug, 1817); **Location:** country: Italy; countryCode: IT; stateProvince: Roma; locality: Riserva Naturale Valle dei Casali 1; decimalLatitude: 41.8710627; decimalLongitude: 12.4336809; geodeticDatum: WGS84; coordinatePrecision: 0.0002; **Identification:** identifiedBy: M. Mei; **Event:** eventDate: 2022-06-18; **Record Level:** collectionCode: UR3

#### Megachile apicalis

Spinola, 1808

7740C766-A06A-5218-B75B-982A73FA8E73

##### Materials

- **Type status:** Other material. **Occurrence:** catalogNumber: A1124; recordedBy: L. Fortini; individualCount: 1; sex: male; lifeStage: adult; occurrenceID: 4444E908-6B95-5B8F-AFC0-9F65D9416191; **Taxon:** scientificName: Megachile (Eutricharaea) apicalis Spinola, 1808; order: Hymenoptera; family: Megachilidae; genus: Megachile; subgenus: Eutricharaea; specificEpithet: apicalis; scientificNameAuthorship: Spinola, 1808; **Location:** country: Italy; countryCode: IT; stateProvince: Roma; locality: Riserva Regionale dell'Appia Antica 1; decimalLatitude: 41.8623941; decimalLongitude: 12.524863; geodeticDatum: WGS84; coordinatePrecision: 0.0002; **Identification:** identifiedBy: M. Mei; **Event:** eventDate: 2022-06-12; **Record Level:** collectionCode: UR3

#### Megachile argentata

(Fabricius, 1793)

9450E82D-07F8-5496-B005-6888FF3914FA

##### Materials

- **Type status:** Other material. **Occurrence:** catalogNumber: A1122; recordedBy: L. Fortini; individualCount: 1; sex: male; lifeStage: adult; occurrenceID: 17045FB1-6DE5-5AED-98EA-19D33A7E0F31; **Taxon:** scientificName: Megachile (Eutricharaea) argentata (Fabricius, 1793); order: Hymenoptera; family: Megachilidae; genus: Megachile; subgenus: Eutricharaea; specificEpithet: argentata; scientificNameAuthorship: (Fabricius, 1793); **Location:** country: Italy; countryCode: IT; stateProvince: Roma; locality: Riserva Naturale Valle dei Casali 1; decimalLatitude: 41.8710627; decimalLongitude: 12.4336809; geodeticDatum: WGS84; coordinatePrecision: 0.0002; **Identification:** identifiedBy: M. Mei; **Event:** eventDate: 2022-07-13; **Record Level:** collectionCode: UR3

#### Megachile centuncularis

(Linnaeus, 1758)

AE4BF772-1DD5-5288-9DE9-E3F4CC432A2C

##### Materials

- **Type status:** Other material. **Occurrence:** catalogNumber: A1171, A1175, A1179, A1186; recordedBy: L. Fortini; individualCount: 4; sex: females; lifeStage: adult; occurrenceID: 420D04E2-C1DF-57EE-B9E1-78B991340DD8; **Taxon:** scientificName: Megachile (Megachile) centuncularis (Linnaeus, 1758); order: Hymenoptera; family: Megachilidae; genus: Megachile; subgenus: Megachile; specificEpithet: centuncularis; scientificNameAuthorship: (Linnaeus, 1758); **Location:** country: Italy; countryCode: IT; stateProvince: Roma; locality: Riserva Naturale dell'Acquafredda; decimalLatitude: 41.8928408; decimalLongitude: 12.39932; geodeticDatum: WGS84; coordinatePrecision: 0.0002; **Identification:** identifiedBy: M. Mei; **Event:** eventDate: 2022-07-12; **Record Level:** collectionCode: UR3
- **Type status:** Other material. **Occurrence:** catalogNumber: A1126, A1133; recordedBy: L. Fortini; individualCount: 2; sex: male; lifeStage: adult; occurrenceID: A2690F5F-C58D-5A8B-A101-B3D772C2BFB7; **Taxon:** scientificName: Megachile (Megachile) centuncularis (Linnaeus, 1758); order: Hymenoptera; family: Megachilidae; genus: Megachile; subgenus: Megachile; specificEpithet: centuncularis; scientificNameAuthorship: (Linnaeus, 1758); **Location:** country: Italy; countryCode: IT; stateProvince: Roma; locality: Riserva Regionale dell'Appia Antica 2; decimalLatitude: 41.8402564; decimalLongitude: 12.532773; geodeticDatum: WGS84; coordinatePrecision: 0.0002; **Identification:** identifiedBy: M. Mei; **Event:** eventDate: 2022-08-29; **Record Level:** collectionCode: UR3
- **Type status:** Other material. **Occurrence:** catalogNumber: A1173; recordedBy: L. Fortini; individualCount: 1; sex: female; lifeStage: adult; occurrenceID: 6E1C2B7C-9FDB-590A-8A83-7C4B68A42446; **Taxon:** scientificName: Megachile (Megachile) centuncularis (Linnaeus, 1758); order: Hymenoptera; family: Megachilidae; genus: Megachile; subgenus: Megachile; specificEpithet: centuncularis; scientificNameAuthorship: (Linnaeus, 1758); **Location:** country: Italy; countryCode: IT; stateProvince: Roma; locality: Riserva Naturale dell'Insugherata 2; decimalLatitude: 41.9599247; decimalLongitude: 12.433852; geodeticDatum: WGS84; coordinatePrecision: 0.0002; **Identification:** identifiedBy: M. Mei; **Event:** eventDate: 2022-07-30; **Record Level:** collectionCode: UR3
- **Type status:** Other material. **Occurrence:** catalogNumber: A1174; recordedBy: L. Fortini; individualCount: 1; sex: female; lifeStage: adult; occurrenceID: 708D2FAE-6B3B-5B84-A9DA-7DF2956AEC3D; **Taxon:** scientificName: Megachile (Megachile) centuncularis (Linnaeus, 1758); order: Hymenoptera; family: Megachilidae; genus: Megachile; subgenus: Megachile; specificEpithet: centuncularis; scientificNameAuthorship: (Linnaeus, 1758); **Location:** country: Italy; countryCode: IT; stateProvince: Roma; locality: Riserva Naturale dell'Insugherata 3; decimalLatitude: 41.9644829; decimalLongitude: 12.436101; geodeticDatum: WGS84; coordinatePrecision: 0.0002; **Identification:** identifiedBy: M. Mei; **Event:** eventDate: 2022-07-30; **Record Level:** collectionCode: UR3
- **Type status:** Other material. **Occurrence:** catalogNumber: A1178; recordedBy: L. Fortini; individualCount: 1; sex: female; lifeStage: adult; occurrenceID: F0585EB3-DFD1-5F85-9A82-8DE0BC938ADA; **Taxon:** scientificName: Megachile (Megachile) centuncularis (Linnaeus, 1758); order: Hymenoptera; family: Megachilidae; genus: Megachile; subgenus: Megachile; specificEpithet: centuncularis; scientificNameAuthorship: (Linnaeus, 1758); **Location:** country: Italy; countryCode: IT; stateProvince: Roma; locality: Riserva Naturale Laurentino-Acqua Acetosa; decimalLatitude: 41.8079275; decimalLongitude: 12.4685548; geodeticDatum: WGS84; coordinatePrecision: 0.0002; **Identification:** identifiedBy: M. Mei; **Event:** eventDate: 2022-07-17; **Record Level:** collectionCode: UR3
- **Type status:** Other material. **Occurrence:** catalogNumber: A1132, A1169, A1172, A1176; recordedBy: L. Fortini; individualCount: 4; sex: 1 male, 3 female; lifeStage: adult; occurrenceID: 637DC5B5-6C90-51BC-9169-1F256FB1EB1C; **Taxon:** scientificName: Megachile (Megachile) centuncularis (Linnaeus, 1758); order: Hymenoptera; family: Megachilidae; genus: Megachile; subgenus: Megachile; specificEpithet: centuncularis; scientificNameAuthorship: (Linnaeus, 1758); **Location:** country: Italy; countryCode: IT; stateProvince: Roma; locality: Riserva Naturale di Monte Mario; decimalLatitude: 41.9386215; decimalLongitude: 12.4546223; geodeticDatum: WGS84; coordinatePrecision: 0.0002; **Identification:** identifiedBy: M. Mei; **Event:** eventDate: 2022-09-21; **Record Level:** collectionCode: UR3
- **Type status:** Other material. **Occurrence:** catalogNumber: A1177; recordedBy: L. Fortini; individualCount: 1; sex: female; lifeStage: adult; occurrenceID: 4F57F630-38CD-5B44-9B33-B866D578CA70; **Taxon:** scientificName: Megachile (Megachile) centuncularis (Linnaeus, 1758); order: Hymenoptera; family: Megachilidae; genus: Megachile; subgenus: Megachile; specificEpithet: centuncularis; scientificNameAuthorship: (Linnaeus, 1758); **Location:** country: Italy; countryCode: IT; stateProvince: Roma; locality: Riserva Naturale di Monte Mario; decimalLatitude: 41.9386215; decimalLongitude: 12.4546223; geodeticDatum: WGS84; coordinatePrecision: 0.0002; **Identification:** identifiedBy: M. Mei; **Event:** eventDate: 2022-05-20; **Record Level:** collectionCode: UR3
- **Type status:** Other material. **Occurrence:** catalogNumber: A1170; recordedBy: L. Fortini; individualCount: 1; sex: female; lifeStage: adult; occurrenceID: 7F12163C-4AEA-5E92-947C-218A49C1F1B8; **Taxon:** scientificName: Megachile (Megachile) centuncularis (Linnaeus, 1758); order: Hymenoptera; family: Megachilidae; genus: Megachile; subgenus: Megachile; specificEpithet: centuncularis; scientificNameAuthorship: (Linnaeus, 1758); **Location:** country: Italy; countryCode: IT; stateProvince: Roma; locality: Riserva Naturale Valle dell'Aniene 2; decimalLatitude: 41.928752; decimalLongitude: 12.5562962; geodeticDatum: WGS84; coordinatePrecision: 0.0002; **Identification:** identifiedBy: M. Mei; **Event:** eventDate: 2022-09-04; **Record Level:** collectionCode: UR3
- **Type status:** Other material. **Occurrence:** catalogNumber: A1187; recordedBy: L. Fortini; individualCount: 1; sex: female; lifeStage: adult; occurrenceID: 0A576D90-45A5-532D-9F35-F26BD4A569F0; **Taxon:** scientificName: Megachile (Megachile) centuncularis (Linnaeus, 1758); order: Hymenoptera; family: Megachilidae; genus: Megachile; subgenus: Megachile; specificEpithet: centuncularis; scientificNameAuthorship: (Linnaeus, 1758); **Location:** country: Italy; countryCode: IT; stateProvince: Roma; locality: Riserva Naturale Valle dell'Aniene 2; decimalLatitude: 41.928752; decimalLongitude: 12.5562962; geodeticDatum: WGS84; coordinatePrecision: 0.0002; **Identification:** identifiedBy: M. Mei; **Event:** eventDate: 2022-06-05; **Record Level:** collectionCode: UR3
- **Type status:** Other material. **Occurrence:** catalogNumber: A2041, A2042; recordedBy: L. Fortini; individualCount: 2; sex: males; lifeStage: adult; occurrenceID: 68C3DE34-C5AA-5862-987D-609CD5ED7A1C; **Taxon:** scientificName: Megachile (Megachile) centuncularis (Linnaeus, 1758); order: Hymenoptera; family: Megachilidae; genus: Megachile; subgenus: Megachile; specificEpithet: centuncularis; scientificNameAuthorship: (Linnaeus, 1758); **Location:** country: Italy; countryCode: IT; stateProvince: Roma; locality: Riserva Naturale Valle dei Casali 1; decimalLatitude: 41.8710627; decimalLongitude: 12.4336809; geodeticDatum: WGS84; coordinatePrecision: 0.0002; **Identification:** identifiedBy: M. Mei; **Event:** eventDate: 2022-08-19; **Record Level:** collectionCode: UR3
- **Type status:** Other material. **Occurrence:** catalogNumber: A2067, A2068; recordedBy: L. Fortini; individualCount: 2; sex: females; lifeStage: adult; occurrenceID: F2142B86-5F6D-5C7A-9963-F9582239F023; **Taxon:** scientificName: Megachile (Megachile) centuncularis (Linnaeus, 1758); order: Hymenoptera; family: Megachilidae; genus: Megachile; subgenus: Megachile; specificEpithet: centuncularis; scientificNameAuthorship: (Linnaeus, 1758); **Location:** country: Italy; countryCode: IT; stateProvince: Roma; locality: Riserva Naturale di Monte Mario; decimalLatitude: 41.9386215; decimalLongitude: 12.4546223; geodeticDatum: WGS84; coordinatePrecision: 0.0002; **Identification:** identifiedBy: M. Mei; **Event:** eventDate: 2022-07-24; **Record Level:** collectionCode: UR3

#### Megachile ericetorum

(Lepeletier, 1841)

4C567464-FC21-5F8B-8089-497668AA7A2D

##### Materials

- **Type status:** Other material. **Occurrence:** catalogNumber: A1111, A1112, A1121; recordedBy: L. Fortini; individualCount: 3; sex: males; lifeStage: adult; occurrenceID: 6E6C611A-F9F3-5874-A939-4499849B590F; **Taxon:** scientificName: Megachile (Pseudomegachile) ericetorum Lepeletier, 1841; order: Hymenoptera; family: Megachilidae; genus: Megachile; subgenus: Pseudomegachile; specificEpithet: ericetorum; scientificNameAuthorship: Lepeletier, 1841; **Location:** country: Italy; countryCode: IT; stateProvince: Roma; locality: Riserva Regionale dell'Appia Antica 1; decimalLatitude: 41.8623941; decimalLongitude: 12.524863; geodeticDatum: WGS84; coordinatePrecision: 0.0002; **Identification:** identifiedBy: M. Mei; **Event:** eventDate: 2022-06-12; **Record Level:** collectionCode: UR3
- **Type status:** Other material. **Occurrence:** catalogNumber: A1123; recordedBy: L. Fortini; individualCount: 1; sex: male; lifeStage: adult; occurrenceID: E9030002-C8AE-5DFA-829B-F5BBA7B271F5; **Taxon:** scientificName: Megachile (Pseudomegachile) ericetorum Lepeletier, 1841; order: Hymenoptera; family: Megachilidae; genus: Megachile; subgenus: Pseudomegachile; specificEpithet: ericetorum; scientificNameAuthorship: Lepeletier, 1841; **Location:** country: Italy; countryCode: IT; stateProvince: Roma; locality: Riserva Regionale dell'Appia Antica 1; decimalLatitude: 41.8623941; decimalLongitude: 12.524863; geodeticDatum: WGS84; coordinatePrecision: 0.0002; **Identification:** identifiedBy: M. Mei; **Event:** eventDate: 2022-05-10; **Record Level:** collectionCode: UR3
- **Type status:** Other material. **Occurrence:** catalogNumber: A1109; recordedBy: L. Fortini; individualCount: 1; sex: male; lifeStage: adult; occurrenceID: CDA9E148-226F-5377-A505-24ABAB25CA59; **Taxon:** scientificName: Megachile (Pseudomegachile) ericetorum Lepeletier, 1841; order: Hymenoptera; family: Megachilidae; genus: Megachile; subgenus: Pseudomegachile; specificEpithet: ericetorum; scientificNameAuthorship: Lepeletier, 1841; **Location:** country: Italy; countryCode: IT; stateProvince: Roma; locality: Riserva Naturale dell'Insugherata 2; decimalLatitude: 41.9599247; decimalLongitude: 12.433852; geodeticDatum: WGS84; coordinatePrecision: 0.0002; **Identification:** identifiedBy: M. Mei; **Event:** eventDate: 2022-05-20; **Record Level:** collectionCode: UR3
- **Type status:** Other material. **Occurrence:** catalogNumber: A1145, A1147; recordedBy: L. Fortini; individualCount: 2; sex: females; lifeStage: adult; occurrenceID: 40F21782-CEE1-5F38-AA9E-EB2B6CE7F26A; **Taxon:** scientificName: Megachile (Pseudomegachile) ericetorum Lepeletier, 1841; order: Hymenoptera; family: Megachilidae; genus: Megachile; subgenus: Pseudomegachile; specificEpithet: ericetorum; scientificNameAuthorship: Lepeletier, 1841; **Location:** country: Italy; countryCode: IT; stateProvince: Roma; locality: Riserva Naturale dell'Insugherata 2; decimalLatitude: 41.9599247; decimalLongitude: 12.433852; geodeticDatum: WGS84; coordinatePrecision: 0.0002; **Identification:** identifiedBy: M. Mei; **Event:** eventDate: 2022-06-24; **Record Level:** collectionCode: UR3
- **Type status:** Other material. **Occurrence:** catalogNumber: A1108; recordedBy: L. Fortini; individualCount: 1; sex: male; lifeStage: adult; occurrenceID: 17951200-57CF-53ED-990F-853CF8772FAE; **Taxon:** scientificName: Megachile (Pseudomegachile) ericetorum Lepeletier, 1841; order: Hymenoptera; family: Megachilidae; genus: Megachile; subgenus: Pseudomegachile; specificEpithet: ericetorum; scientificNameAuthorship: Lepeletier, 1841; **Location:** country: Italy; countryCode: IT; stateProvince: Roma; locality: Riserva Naturale Laurentino-Acqua Acetosa; decimalLatitude: 41.8079275; decimalLongitude: 12.4685548; geodeticDatum: WGS84; coordinatePrecision: 0.0002; **Identification:** identifiedBy: M. Mei; **Event:** eventDate: 2022-06-16; **Record Level:** collectionCode: UR3
- **Type status:** Other material. **Occurrence:** catalogNumber: A1146, A1148, A1149, A1152, A1153, A1161; recordedBy: L. Fortini; individualCount: 6; sex: female; lifeStage: adult; occurrenceID: 4FC353E1-7A50-5063-8453-AA2E8F4D8B71; **Taxon:** scientificName: Megachile (Pseudomegachile) ericetorum Lepeletier, 1841; order: Hymenoptera; family: Megachilidae; genus: Megachile; subgenus: Pseudomegachile; specificEpithet: ericetorum; scientificNameAuthorship: Lepeletier, 1841; **Location:** country: Italy; countryCode: IT; stateProvince: Roma; locality: Riserva Naturale Valle dell'Aniene 1; decimalLatitude: 41.9345179; decimalLongitude: 12.5453096; geodeticDatum: WGS84; coordinatePrecision: 0.0002; **Identification:** identifiedBy: M. Mei; **Event:** eventDate: 2022-06-05; **Record Level:** collectionCode: UR3
- **Type status:** Other material. **Occurrence:** catalogNumber: A1110, A1120, A1150, A1151, A1154, A1155, A1156, A1162; recordedBy: L. Fortini; individualCount: 8; sex: 2 males, 6 females; lifeStage: adult; occurrenceID: 5E7BCFD0-0769-548D-9450-34E2DEE73FC4; **Taxon:** scientificName: Megachile (Pseudomegachile) ericetorum Lepeletier, 1841; order: Hymenoptera; family: Megachilidae; genus: Megachile; subgenus: Pseudomegachile; specificEpithet: ericetorum; scientificNameAuthorship: Lepeletier, 1841; **Location:** country: Italy; countryCode: IT; stateProvince: Roma; locality: Riserva Naturale Valle dell'Aniene 2; decimalLatitude: 41.928752; decimalLongitude: 12.5562962; geodeticDatum: WGS84; coordinatePrecision: 0.0002; **Identification:** identifiedBy: M. Mei; **Event:** eventDate: 2022-06-05; **Record Level:** collectionCode: UR3

#### Megachile flabellipes

Pérez, 1895

83002E85-6D7F-50FB-BB91-CE0A329F7963

##### Materials

- **Type status:** Other material. **Occurrence:** catalogNumber: A1125; recordedBy: L. Fortini; individualCount: 1; sex: male; lifeStage: adult; occurrenceID: 9FE7BB66-45A8-5928-A6EE-31E721A9C942; **Taxon:** scientificName: Megachile (Eutricharaea) flabellipes Pérez, 1895; order: Hymenoptera; family: Megachilidae; genus: Megachile; subgenus: Eutricharaea; specificEpithet: flabellipes; scientificNameAuthorship: Pérez, 1895; **Location:** country: Italy; countryCode: IT; stateProvince: Roma; locality: Riserva Naturale Valle dei Casali 1; decimalLatitude: 41.8710627; decimalLongitude: 12.4336809; geodeticDatum: WGS84; coordinatePrecision: 0.0002; **Identification:** identifiedBy: M. Mei; **Event:** eventDate: 2022-06-18; **Record Level:** collectionCode: UR3

#### Megachile genalis

Morawitz, 1880

F1C566F1-91FB-54F1-A5C2-4833B0341712

##### Materials

- **Type status:** Other material. **Occurrence:** catalogNumber: A1130; recordedBy: L. Fortini; individualCount: 1; sex: male; lifeStage: adult; occurrenceID: 73E61E05-CE24-511E-AE01-8AC6F08596CC; **Taxon:** scientificName: Megachile (Megachile) genalis Morawitz, 1880; order: Hymenoptera; family: Megachilidae; genus: Megachile; subgenus: Megachile; specificEpithet: genalis; scientificNameAuthorship: Morawitz, 1880; **Location:** country: Italy; countryCode: IT; stateProvince: Roma; locality: Riserva Naturale Tenuta dei Massimi 2; decimalLatitude: 41.8316516; decimalLongitude: 12.3999927; geodeticDatum: WGS84; coordinatePrecision: 0.0002; **Identification:** identifiedBy: M. Mei; **Event:** eventDate: 2022-07-28; **Record Level:** collectionCode: UR3

#### Megachile marginata

Smith, 1853

42497A7D-460C-5843-B607-CEEF82C3602C

##### Materials

- **Type status:** Other material. **Occurrence:** catalogNumber: A1128, A1129; recordedBy: L. Fortini; individualCount: 2; sex: males; lifeStage: adult; occurrenceID: F068F718-8FE5-52F8-82E6-1852D6B7068F; **Taxon:** scientificName: Megachile (Eutricharaea) marginata Smith, 1853; order: Hymenoptera; family: Megachilidae; genus: Megachile; subgenus: Eutricharaea; specificEpithet: marginata; scientificNameAuthorship: Smith, 1853; **Location:** country: Italy; countryCode: IT; stateProvince: Roma; locality: Riserva Naturale dell'Insugherata 1; decimalLatitude: 41.9555045; decimalLongitude: 12.4292321; geodeticDatum: WGS84; coordinatePrecision: 0.0002; **Identification:** identifiedBy: M. Mei; **Event:** eventDate: 2022-07-30; **Record Level:** collectionCode: UR3

#### Megachile maritima

(Kirby, 1802)

A878AAC0-4ACB-52C8-8ABB-A49C9F0B768C

##### Materials

- **Type status:** Other material. **Occurrence:** catalogNumber: A1158; recordedBy: L. Fortini; individualCount: 1; sex: female; lifeStage: adult; occurrenceID: F978DFBA-6E2D-5BD3-BC70-3EF70A7A38AB; **Taxon:** scientificName: Megachile (Xanthosarus) maritima (Kirby, 1802); order: Hymenoptera; family: Megachilidae; genus: Megachile; subgenus: Xanthosarus; specificEpithet: maritima; scientificNameAuthorship: (Kirby, 1802); **Location:** country: Italy; countryCode: IT; stateProvince: Roma; locality: Riserva Regionale dell'Appia Antica 2; decimalLatitude: 41.8402564; decimalLongitude: 12.532773; geodeticDatum: WGS84; coordinatePrecision: 0.0002; **Identification:** identifiedBy: M. Mei; **Event:** eventDate: 2022-08-06; **Record Level:** collectionCode: UR3

#### Megachile melanophyga

Costa, 1863

A9D4B969-C010-5D75-95CF-A9E709431A59

##### Materials

- **Type status:** Other material. **Occurrence:** catalogNumber: A1119; recordedBy: L. Fortini; individualCount: 1; sex: male; lifeStage: adult; occurrenceID: E5991F73-544D-5A42-827A-896823D8687E; **Taxon:** scientificName: Megachile (Megachile) melanopyga Costa, 1863; order: Hymenoptera; family: Megachilidae; genus: Megachile; subgenus: Megachile; specificEpithet: melanopyga; scientificNameAuthorship: Costa, 1863; **Location:** country: Italy; countryCode: IT; stateProvince: Roma; locality: Riserva Regionale dell'Appia Antica 1; decimalLatitude: 41.8623941; decimalLongitude: 12.524863; geodeticDatum: WGS84; coordinatePrecision: 0.0002; **Identification:** identifiedBy: M. Mei; **Event:** eventDate: 2022-07-22; **Record Level:** collectionCode: UR3
- **Type status:** Other material. **Occurrence:** catalogNumber: A1165; recordedBy: L. Fortini; individualCount: 1; sex: female; lifeStage: adult; occurrenceID: 35960970-7183-5F75-9121-2B347139B105; **Taxon:** scientificName: Megachile (Megachile) melanopyga Costa, 1863; order: Hymenoptera; family: Megachilidae; genus: Megachile; subgenus: Megachile; specificEpithet: melanopyga; scientificNameAuthorship: Costa, 1863; **Location:** country: Italy; countryCode: IT; stateProvince: Roma; locality: Riserva Naturale Laurentino-Acqua Acetosa; decimalLatitude: 41.8079275; decimalLongitude: 12.4685548; geodeticDatum: WGS84; coordinatePrecision: 0.0002; **Identification:** identifiedBy: M. Mei; **Event:** eventDate: 2022-07-17; **Record Level:** collectionCode: UR3
- **Type status:** Other material. **Occurrence:** catalogNumber: A1164; recordedBy: L. Fortini; individualCount: 1; sex: female; lifeStage: adult; occurrenceID: 3304C2F7-E30D-5447-B00C-4D8483A7BBC0; **Taxon:** scientificName: Megachile (Megachile) melanopyga Costa, 1863; order: Hymenoptera; family: Megachilidae; genus: Megachile; subgenus: Megachile; specificEpithet: melanopyga; scientificNameAuthorship: Costa, 1863; **Location:** country: Italy; countryCode: IT; stateProvince: Roma; locality: Riserva Naturale di Monte Mario; decimalLatitude: 41.9386215; decimalLongitude: 12.4546223; geodeticDatum: WGS84; coordinatePrecision: 0.0002; **Identification:** identifiedBy: M. Mei; **Event:** eventDate: 2022-08-23; **Record Level:** collectionCode: UR3
- **Type status:** Other material. **Occurrence:** catalogNumber: A1163; recordedBy: L. Fortini; individualCount: 1; sex: female; lifeStage: adult; occurrenceID: 27A44336-BE7C-5CAA-AF7D-492B50D3B461; **Taxon:** scientificName: Megachile (Megachile) melanopyga Costa, 1863; order: Hymenoptera; family: Megachilidae; genus: Megachile; subgenus: Megachile; specificEpithet: melanopyga; scientificNameAuthorship: Costa, 1863; **Location:** country: Italy; countryCode: IT; stateProvince: Roma; locality: Riserva Naturale Tenuta dei Massimi 2; decimalLatitude: 41.8316516; decimalLongitude: 12.3999927; geodeticDatum: WGS84; coordinatePrecision: 0.0002; **Identification:** identifiedBy: M. Mei; **Event:** eventDate: 2022-09-28; **Record Level:** collectionCode: UR3
- **Type status:** Other material. **Occurrence:** catalogNumber: A1167; recordedBy: L. Fortini; individualCount: 1; sex: male; lifeStage: adult; occurrenceID: CCB42D1B-9C4E-5432-B6E7-B1D57411E765; **Taxon:** scientificName: Megachile (Megachile) melanopyga Costa, 1863; order: Hymenoptera; family: Megachilidae; genus: Megachile; subgenus: Megachile; specificEpithet: melanopyga; scientificNameAuthorship: Costa, 1863; **Location:** country: Italy; countryCode: IT; stateProvince: Roma; locality: Riserva Naturale Valle dei Casali 1; decimalLatitude: 41.8710627; decimalLongitude: 12.4336809; geodeticDatum: WGS84; coordinatePrecision: 0.0002; **Identification:** identifiedBy: M. Mei; **Event:** eventDate: 2022-06-18; **Record Level:** collectionCode: UR3

#### Megachile octosignata

Nylander, 1852

D92C3247-585C-5822-AB28-7C485DD774B1

##### Materials

- **Type status:** Other material. **Occurrence:** catalogNumber: A1127; recordedBy: L. Fortini; individualCount: 1; sex: male; lifeStage: adult; occurrenceID: 9596B171-D681-5F17-B7B6-F83B44B0A535; **Taxon:** scientificName: Megachile (Megachile) octosignata Nylander, 1852; order: Hymenoptera; family: Megachilidae; genus: Megachile; subgenus: Megachile; specificEpithet: octosignata; scientificNameAuthorship: Nylander, 1852; **Location:** country: Italy; countryCode: IT; stateProvince: Roma; locality: Riserva Regionale dell'Appia Antica 2; decimalLatitude: 41.8402564; decimalLongitude: 12.532773; geodeticDatum: WGS84; coordinatePrecision: 0.0002; **Identification:** identifiedBy: M. Mei; **Event:** eventDate: 2022-08-29; **Record Level:** collectionCode: UR3

#### Megachile parietina

(Geoffroy, 1785)

C3B865A8-33A7-5204-8C42-4A45F6358C8B

##### Materials

- **Type status:** Other material. **Occurrence:** catalogNumber: A1180, A1181; recordedBy: L. Fortini; individualCount: 2; sex: males; lifeStage: adult; occurrenceID: C4C87FB2-E9A1-5D29-A566-35067BCD5AC7; **Taxon:** scientificName: Megachile (Chalicodoma) parietina (Geoffroy, 1785); order: Hymenoptera; family: Megachilidae; genus: Megachile; subgenus: Chalicodoma; specificEpithet: parietina; scientificNameAuthorship: (Geoffroy, 1785); **Location:** country: Italy; countryCode: IT; stateProvince: Roma; locality: Riserva Regionale dell'Appia Antica 2; decimalLatitude: 41.8402564; decimalLongitude: 12.532773; geodeticDatum: WGS84; coordinatePrecision: 0.0002; **Identification:** identifiedBy: M. Mei; **Event:** eventDate: 2022-05-24; **Record Level:** collectionCode: UR3
- **Type status:** Other material. **Occurrence:** catalogNumber: A1182; recordedBy: L. Fortini; individualCount: 1; sex: male; lifeStage: adult; occurrenceID: 760C99C0-F792-552C-BF21-7D740D258F39; **Taxon:** scientificName: Megachile (Chalicodoma) parietina (Geoffroy, 1785); order: Hymenoptera; family: Megachilidae; genus: Megachile; subgenus: Chalicodoma; specificEpithet: parietina; scientificNameAuthorship: (Geoffroy, 1785); **Location:** country: Italy; countryCode: IT; stateProvince: Roma; locality: Riserva Regionale dell'Appia Antica 3; decimalLatitude: 41.8298456; decimalLongitude: 12.5432538; geodeticDatum: WGS84; coordinatePrecision: 0.0002; **Identification:** identifiedBy: M. Mei; **Event:** eventDate: 2022-05-24; **Record Level:** collectionCode: UR3
- **Type status:** Other material. **Occurrence:** catalogNumber: A1185; recordedBy: L. Fortini; individualCount: 1; sex: female; lifeStage: adult; occurrenceID: 0A5A7BA6-3C55-51D8-BCD0-A119D53E5764; **Taxon:** scientificName: Megachile (Chalicodoma) parietina (Geoffroy, 1785); order: Hymenoptera; family: Megachilidae; genus: Megachile; subgenus: Chalicodoma; specificEpithet: parietina; scientificNameAuthorship: (Geoffroy, 1785); **Location:** country: Italy; countryCode: IT; stateProvince: Roma; locality: Riserva Regionale dell'Appia Antica 3; decimalLatitude: 41.8298456; decimalLongitude: 12.5432538; geodeticDatum: WGS84; coordinatePrecision: 0.0002; **Identification:** identifiedBy: M. Mei; **Event:** eventDate: 2022-05-04; **Record Level:** collectionCode: UR3
- **Type status:** Other material. **Occurrence:** catalogNumber: A1183, A1184; recordedBy: L. Fortini; individualCount: 2; sex: females; lifeStage: adult; occurrenceID: 76354EFE-BF54-5253-86A5-9F81AE4B6A7F; **Taxon:** scientificName: Megachile (Chalicodoma) parietina (Geoffroy, 1785); order: Hymenoptera; family: Megachilidae; genus: Megachile; subgenus: Chalicodoma; specificEpithet: parietina; scientificNameAuthorship: (Geoffroy, 1785); **Location:** country: Italy; countryCode: IT; stateProvince: Roma; locality: Riserva Naturale Tenuta dei Massimi 2; decimalLatitude: 41.8316516; decimalLongitude: 12.3999927; geodeticDatum: WGS84; coordinatePrecision: 0.0002; **Identification:** identifiedBy: M. Mei; **Event:** eventDate: 2022-05-04; **Record Level:** collectionCode: UR3

#### Megachile sculpturalis

Smith, 1853

B79B286C-E0E7-51D3-A582-50D822C5C1AA

##### Materials

- **Type status:** Other material. **Occurrence:** catalogNumber: A1168; recordedBy: L. Fortini; individualCount: 1; sex: male; lifeStage: adult; occurrenceID: 007E01F5-5997-5449-B740-130B6CA7F3E4; **Taxon:** scientificName: Megachile (Callomegachile) sculpturalis Smith, 1853; order: Hymenoptera; family: Megachilidae; genus: Megachile; subgenus: Callomegachile; specificEpithet: sculpturalis; scientificNameAuthorship: Smith, 1853; **Location:** country: Italy; countryCode: IT; stateProvince: Roma; locality: Riserva Naturale dell'Insugherata 2; decimalLatitude: 41.9599247; decimalLongitude: 12.433852; geodeticDatum: WGS84; coordinatePrecision: 0.0002; **Identification:** identifiedBy: M. Mei; **Event:** eventDate: 2022-06-24; **Record Level:** collectionCode: UR3

#### Megachile versicolor

Smith, 1844

E04C4981-C877-54C1-9B08-A153BE92E5C2

##### Materials

- **Type status:** Other material. **Occurrence:** catalogNumber: A1131; recordedBy: L. Fortini; individualCount: 1; sex: male; lifeStage: adult; occurrenceID: 87B7385B-DA79-575B-A7E9-904AEF40EE73; **Taxon:** scientificName: Megachile (Megachile) versicolor Smith, 1844; order: Hymenoptera; family: Megachilidae; genus: Megachile; subgenus: Megachile; specificEpithet: versicolor; scientificNameAuthorship: Smith, 1844; **Location:** country: Italy; countryCode: IT; stateProvince: Roma; locality: Riserva Naturale dell'Insugherata 3; decimalLatitude: 41.9644829; decimalLongitude: 12.436101; geodeticDatum: WGS84; coordinatePrecision: 0.0002; **Identification:** identifiedBy: M. Mei; **Event:** eventDate: 2022-06-24; **Record Level:** collectionCode: UR3

#### Megachile willughbiella

(Kirby, 1802)

E9DE2DEA-1BF2-5034-BEEF-42B870E3F5EC

##### Materials

- **Type status:** Other material. **Occurrence:** catalogNumber: A1157; recordedBy: L. Fortini; individualCount: 1; sex: female; lifeStage: adult; occurrenceID: 2C2A51F7-7CA0-5FD6-8374-5CCB8032858A; **Taxon:** scientificName: Megachile (Xanthosarus) willughbiella (Kirby, 1802); order: Hymenoptera; family: Megachilidae; genus: Megachile; subgenus: Xanthosarus; specificEpithet: willughbiella; scientificNameAuthorship: (Kirby, 1802); **Location:** country: Italy; countryCode: IT; stateProvince: Roma; locality: Riserva Naturale Valle dell'Aniene 2; decimalLatitude: 41.928752; decimalLongitude: 12.5562962; geodeticDatum: WGS84; coordinatePrecision: 0.0002; **Identification:** identifiedBy: M. Mei; **Event:** eventDate: 2022-06-05; **Record Level:** collectionCode: UR3
- **Type status:** Other material. **Occurrence:** catalogNumber: A1159; recordedBy: L. Fortini; individualCount: 1; sex: female; lifeStage: adult; occurrenceID: 8D2D6F57-56C2-5344-9D91-8B5B61FF2EE5; **Taxon:** scientificName: Megachile (Xanthosarus) willughbiella (Kirby, 1802); order: Hymenoptera; family: Megachilidae; genus: Megachile; subgenus: Xanthosarus; specificEpithet: willughbiella; scientificNameAuthorship: (Kirby, 1802); **Location:** country: Italy; countryCode: IT; stateProvince: Roma; locality: Riserva Naturale Valle dell'Aniene 1; decimalLatitude: 41.9345179; decimalLongitude: 12.5453096; geodeticDatum: WGS84; coordinatePrecision: 0.0002; **Identification:** identifiedBy: M. Mei; **Event:** eventDate: 2022-06-05; **Record Level:** collectionCode: UR3
- **Type status:** Other material. **Occurrence:** catalogNumber: A1160; recordedBy: L. Fortini; individualCount: 1; sex: female; lifeStage: adult; occurrenceID: 98B8CD32-AEFD-5FB3-9B75-9E8131B84113; **Taxon:** scientificName: Megachile (Xanthosarus) willughbiella (Kirby, 1802); order: Hymenoptera; family: Megachilidae; genus: Megachile; subgenus: Xanthosarus; specificEpithet: willughbiella; scientificNameAuthorship: (Kirby, 1802); **Location:** country: Italy; countryCode: IT; stateProvince: Roma; locality: Riserva Naturale Laurentino-Acqua Acetosa; decimalLatitude: 41.8079275; decimalLongitude: 12.4685548; geodeticDatum: WGS84; coordinatePrecision: 0.0002; **Identification:** identifiedBy: M. Mei; **Event:** eventDate: 2022-06-16; **Record Level:** collectionCode: UR3

#### Osmia aurulenta

Panzer, 1799

0989A796-4542-54C5-88B7-614C1BF42740

##### Materials

- **Type status:** Other material. **Occurrence:** catalogNumber: A0412; recordedBy: L. Fortini; individualCount: 1; sex: female; lifeStage: adult; occurrenceID: E4946332-A8AA-57CF-93E7-A15667AC5B4C; **Taxon:** scientificName: Osmia (Helicosmia) aurulenta (Panzer, 1799); order: Hymenoptera; family: Megachilidae; genus: Osmia; subgenus: Helicosmia; specificEpithet: aurulenta; scientificNameAuthorship: (Panzer, 1799); **Location:** country: Italy; countryCode: IT; stateProvince: Roma; locality: Riserva Naturale Laurentino-Acqua Acetosa; decimalLatitude: 41.8079275; decimalLongitude: 12.4685548; geodeticDatum: WGS84; coordinatePrecision: 0.0002; **Identification:** identifiedBy: M. Mei; **Event:** eventDate: 2022-05-12; **Record Level:** collectionCode: UR3

#### Osmia bicornis

(Linnaeus, 1758)

44C9A67B-A27B-5F35-BF94-C5C5988386F9

##### Materials

- **Type status:** Other material. **Occurrence:** catalogNumber: A0418; recordedBy: L. Fortini; individualCount: 1; sex: female; lifeStage: adult; occurrenceID: 9521DF49-8F2D-5718-B4EE-A360274EEC11; **Taxon:** scientificName: Osmia (Osmia) bicornis (Linnaeus, 1758); order: Hymenoptera; family: Megachilidae; genus: Osmia; subgenus: Osmia; specificEpithet: bicornis; scientificNameAuthorship: (Linnaeus,1758); **Location:** country: Italy; countryCode: IT; stateProvince: Roma; locality: Riserva Naturale Valle dell'Aniene 1; decimalLatitude: 41.9345179; decimalLongitude: 12.5453096; geodeticDatum: WGS84; coordinatePrecision: 0.0002; **Identification:** identifiedBy: M. Mei; **Event:** eventDate: 2022-04-28; **Record Level:** collectionCode: UR3
- **Type status:** Other material. **Occurrence:** catalogNumber: A0419; recordedBy: L. Fortini; individualCount: 1; sex: female; lifeStage: adult; occurrenceID: 65D42DA8-6026-5410-82FB-81AF7AAA71DF; **Taxon:** scientificName: Osmia (Osmia) bicornis (Linnaeus, 1758); order: Hymenoptera; family: Megachilidae; genus: Osmia; subgenus: Osmia; specificEpithet: bicornis; scientificNameAuthorship: (Linnaeus,1758); **Location:** country: Italy; countryCode: IT; stateProvince: Roma; locality: Riserva Naturale di Monte Mario; decimalLatitude: 41.9386215; decimalLongitude: 12.4546223; geodeticDatum: WGS84; coordinatePrecision: 0.0002; **Identification:** identifiedBy: M. Mei; **Event:** eventDate: 2022-04-20; **Record Level:** collectionCode: UR3
- **Type status:** Other material. **Occurrence:** catalogNumber: A0420; recordedBy: L. Fortini; individualCount: 1; sex: female; lifeStage: adult; occurrenceID: FB775090-7E59-5DCA-97C5-8454E212DA06; **Taxon:** scientificName: Osmia (Osmia) bicornis (Linnaeus, 1758); order: Hymenoptera; family: Megachilidae; genus: Osmia; subgenus: Osmia; specificEpithet: bicornis; scientificNameAuthorship: (Linnaeus,1758); **Location:** country: Italy; countryCode: IT; stateProvince: Roma; locality: Riserva Naturale Valle dell'Aniene 2; decimalLatitude: 41.928752; decimalLongitude: 12.5562962; geodeticDatum: WGS84; coordinatePrecision: 0.0002; **Identification:** identifiedBy: M. Mei; **Event:** eventDate: 2022-04-28; **Record Level:** collectionCode: UR3

#### Osmia brevicornis

(Fabricius, 1798)

B6F45F2B-8FBB-5235-A0E9-0118E0EB768B

##### Materials

- **Type status:** Other material. **Occurrence:** catalogNumber: A0429; recordedBy: L. Fortini; individualCount: 1; sex: female; lifeStage: adult; occurrenceID: 9772FBAE-00D8-5DCA-B533-C9BFE6CC9F9F; **Taxon:** scientificName: Osmia (Metallinella) brevicornis (Fabricius, 1798); order: Hymenoptera; family: Megachilidae; genus: Osmia; subgenus: Metallinella; specificEpithet: brevicornis; scientificNameAuthorship: (Fabricius, 1798); **Location:** country: Italy; countryCode: IT; stateProvince: Roma; locality: Riserva Regionale dell'Appia Antica 2; decimalLatitude: 41.8402564; decimalLongitude: 12.532773; geodeticDatum: WGS84; coordinatePrecision: 0.0002; **Identification:** identifiedBy: M. Mei; **Event:** eventDate: 2022-05-24; **Record Level:** collectionCode: UR3
- **Type status:** Other material. **Occurrence:** catalogNumber: A0426, A0427; recordedBy: L. Fortini; individualCount: 2; sex: males; lifeStage: adult; occurrenceID: 2E4CCC0D-2F62-5C22-8DC7-ADEC3654D634; **Taxon:** scientificName: Osmia (Metallinella) brevicornis (Fabricius, 1798); order: Hymenoptera; family: Megachilidae; genus: Osmia; subgenus: Metallinella; specificEpithet: brevicornis; scientificNameAuthorship: (Fabricius, 1798); **Location:** country: Italy; countryCode: IT; stateProvince: Roma; locality: Riserva Naturale dell'Insugherata 1; decimalLatitude: 41.9555045; decimalLongitude: 12.4292321; geodeticDatum: WGS84; coordinatePrecision: 0.0002; **Identification:** identifiedBy: M. Mei; **Event:** eventDate: 2022-04-15; **Record Level:** collectionCode: UR3
- **Type status:** Other material. **Occurrence:** catalogNumber: A0432; recordedBy: L. Fortini; individualCount: 1; sex: female; lifeStage: adult; occurrenceID: 6D3F2E8A-DA85-5A35-81F9-2C754CDA9F96; **Taxon:** scientificName: Osmia (Metallinella) brevicornis (Fabricius, 1798); order: Hymenoptera; family: Megachilidae; genus: Osmia; subgenus: Metallinella; specificEpithet: brevicornis; scientificNameAuthorship: (Fabricius, 1798); **Location:** country: Italy; countryCode: IT; stateProvince: Roma; locality: Riserva Naturale dell'Insugherata 1; decimalLatitude: 41.9555045; decimalLongitude: 12.4292321; geodeticDatum: WGS84; coordinatePrecision: 0.0002; **Identification:** identifiedBy: M. Mei; **Event:** eventDate: 2022-05-27; **Record Level:** collectionCode: UR3
- **Type status:** Other material. **Occurrence:** catalogNumber: A0430, A0431, A0434; recordedBy: L. Fortini; individualCount: 3; sex: females; lifeStage: adult; occurrenceID: BFA2CCBE-0348-51A7-981B-9303146734E9; **Taxon:** scientificName: Osmia (Metallinella) brevicornis (Fabricius, 1798); order: Hymenoptera; family: Megachilidae; genus: Osmia; subgenus: Metallinella; specificEpithet: brevicornis; scientificNameAuthorship: (Fabricius, 1798); **Location:** country: Italy; countryCode: IT; stateProvince: Roma; locality: Riserva Naturale dell'Insugherata 2; decimalLatitude: 41.9599247; decimalLongitude: 12.433852; geodeticDatum: WGS84; coordinatePrecision: 0.0002; **Identification:** identifiedBy: M. Mei; **Event:** eventDate: 2022-05-27; **Record Level:** collectionCode: UR3
- **Type status:** Other material. **Occurrence:** catalogNumber: A0435; recordedBy: L. Fortini; individualCount: 1; sex: female; lifeStage: adult; occurrenceID: A203230B-A1B8-58AE-9D74-2FB38527C2E0; **Taxon:** scientificName: Osmia (Metallinella) brevicornis (Fabricius, 1798); order: Hymenoptera; family: Megachilidae; genus: Osmia; subgenus: Metallinella; specificEpithet: brevicornis; scientificNameAuthorship: (Fabricius, 1798); **Location:** country: Italy; countryCode: IT; stateProvince: Roma; locality: Riserva Naturale dell'Insugherata 3; decimalLatitude: 41.9644829; decimalLongitude: 12.436101; geodeticDatum: WGS84; coordinatePrecision: 0.0002; **Identification:** identifiedBy: M. Mei; **Event:** eventDate: 2022-05-27; **Record Level:** collectionCode: UR3
- **Type status:** Other material. **Occurrence:** catalogNumber: A0421, A0433; recordedBy: L. Fortini; individualCount: 2; sex: 1 male, 1 female; lifeStage: adult; occurrenceID: 1DD1AAC1-D63B-5A9B-9758-20CF49F502E8; **Taxon:** scientificName: Osmia (Metallinella) brevicornis (Fabricius, 1798); order: Hymenoptera; family: Megachilidae; genus: Osmia; subgenus: Metallinella; specificEpithet: brevicornis; scientificNameAuthorship: (Fabricius, 1798); **Location:** country: Italy; countryCode: IT; stateProvince: Roma; locality: Riserva Naturale Laurentino-Acqua Acetosa; decimalLatitude: 41.8079275; decimalLongitude: 12.4685548; geodeticDatum: WGS84; coordinatePrecision: 0.0002; **Identification:** identifiedBy: M. Mei; **Event:** eventDate: 2022-05-12; **Record Level:** collectionCode: UR3
- **Type status:** Other material. **Occurrence:** catalogNumber: A0425; recordedBy: L. Fortini; individualCount: 1; sex: male; lifeStage: adult; occurrenceID: 9FF45CAF-94E5-5F90-A207-938320878831; **Taxon:** scientificName: Osmia (Metallinella) brevicornis (Fabricius, 1798); order: Hymenoptera; family: Megachilidae; genus: Osmia; subgenus: Metallinella; specificEpithet: brevicornis; scientificNameAuthorship: (Fabricius, 1798); **Location:** country: Italy; countryCode: IT; stateProvince: Roma; locality: Riserva Naturale Laurentino-Acqua Acetosa; decimalLatitude: 41.8079275; decimalLongitude: 12.4685548; geodeticDatum: WGS84; coordinatePrecision: 0.0002; **Identification:** identifiedBy: M. Mei; **Event:** eventDate: 2022-04-12; **Record Level:** collectionCode: UR3
- **Type status:** Other material. **Occurrence:** catalogNumber: A0423, A0428, A0436; recordedBy: L. Fortini; individualCount: 3; sex: 2 males, 1 female; lifeStage: adult; occurrenceID: CF3524BD-3395-54E6-B0D6-A1921B1EB234; **Taxon:** scientificName: Osmia (Metallinella) brevicornis (Fabricius, 1798); order: Hymenoptera; family: Megachilidae; genus: Osmia; subgenus: Metallinella; specificEpithet: brevicornis; scientificNameAuthorship: (Fabricius, 1798); **Location:** country: Italy; countryCode: IT; stateProvince: Roma; locality: Riserva Naturale di Monte Mario; decimalLatitude: 41.9386215; decimalLongitude: 12.4546223; geodeticDatum: WGS84; coordinatePrecision: 0.0002; **Identification:** identifiedBy: M. Mei; **Event:** eventDate: 2022-05-20; **Record Level:** collectionCode: UR3
- **Type status:** Other material. **Occurrence:** catalogNumber: A0422; recordedBy: L. Fortini; individualCount: 1; sex: male; lifeStage: adult; occurrenceID: 9A70610D-7B26-519B-93CF-63A43B85BB3C; **Taxon:** scientificName: Osmia (Metallinella) brevicornis (Fabricius, 1798); order: Hymenoptera; family: Megachilidae; genus: Osmia; subgenus: Metallinella; specificEpithet: brevicornis; scientificNameAuthorship: (Fabricius, 1798); **Location:** country: Italy; countryCode: IT; stateProvince: Roma; locality: Riserva Naturale Tenuta dei Massimi 2; decimalLatitude: 41.8316516; decimalLongitude: 12.3999927; geodeticDatum: WGS84; coordinatePrecision: 0.0002; **Identification:** identifiedBy: M. Mei; **Event:** eventDate: 2022-06-01; **Record Level:** collectionCode: UR3
- **Type status:** Other material. **Occurrence:** catalogNumber: A0424; recordedBy: L. Fortini; individualCount: 1; sex: male; lifeStage: adult; occurrenceID: 36D9625D-8655-5E02-A545-9E89B38523CC; **Taxon:** scientificName: Osmia (Metallinella) brevicornis (Fabricius, 1798); order: Hymenoptera; family: Megachilidae; genus: Osmia; subgenus: Metallinella; specificEpithet: brevicornis; scientificNameAuthorship: (Fabricius, 1798); **Location:** country: Italy; countryCode: IT; stateProvince: Roma; locality: Riserva Naturale Tenuta dei Massimi 2; decimalLatitude: 41.8316516; decimalLongitude: 12.3999927; geodeticDatum: WGS84; coordinatePrecision: 0.0002; **Identification:** identifiedBy: M. Mei; **Event:** eventDate: 2022-05-04; **Record Level:** collectionCode: UR3
- **Type status:** Other material. **Occurrence:** catalogNumber: A0647, A0649; recordedBy: L. Fortini; individualCount: 2; sex: males; lifeStage: adult; occurrenceID: 139FE308-C6AC-5BFC-B233-FC38DE750E70; **Taxon:** scientificName: Osmia (Metallinella) brevicornis (Fabricius, 1798); order: Hymenoptera; family: Megachilidae; genus: Osmia; subgenus: Metallinella; specificEpithet: brevicornis; scientificNameAuthorship: (Fabricius, 1798); **Location:** country: Italy; countryCode: IT; stateProvince: Roma; locality: Riserva Naturale di Monte Mario; decimalLatitude: 41.9386215; decimalLongitude: 12.4546223; geodeticDatum: WGS84; coordinatePrecision: 0.0002; **Identification:** identifiedBy: M. Mei; **Event:** eventDate: 2022-05-20; **Record Level:** collectionCode: UR3
- **Type status:** Other material. **Occurrence:** catalogNumber: A1984; recordedBy: L. Fortini; individualCount: 1; sex: male; lifeStage: adult; occurrenceID: 2889777E-0592-5EDC-BD02-361FAE3476A9; **Taxon:** scientificName: Osmia (Metallinella) brevicornis (Fabricius, 1798); order: Hymenoptera; family: Megachilidae; genus: Osmia; subgenus: Metallinella; specificEpithet: brevicornis; scientificNameAuthorship: (Fabricius, 1798); **Location:** country: Italy; countryCode: IT; stateProvince: Roma; locality: Riserva Regionale dell'Appia Antica 1; decimalLatitude: 41.8623941; decimalLongitude: 12.524863; geodeticDatum: WGS84; coordinatePrecision: 0.0002; **Identification:** identifiedBy: M. Mei; **Event:** eventDate: 2022-04-19; **Record Level:** collectionCode: UR3

#### Osmia caerulescens

(Linnaeus, 1758)

6E0D7838-7B3E-5D11-AAB7-EC99525ACB80

##### Materials

- **Type status:** Other material. **Occurrence:** catalogNumber: A0415; recordedBy: L. Fortini; individualCount: 1; sex: male; lifeStage: adult; occurrenceID: CB802776-B158-5B44-89C0-E0DF6C84E01A; **Taxon:** scientificName: Osmia (Helicosmia) caerulescens (Linnaeus,1758); order: Hymenoptera; family: Megachilidae; genus: Osmia; subgenus: Helicosmia; specificEpithet: caerulescens; scientificNameAuthorship: (Linnaeus,1758); **Location:** country: Italy; countryCode: IT; stateProvince: Roma; locality: Riserva Naturale Valle dei Casali 2; decimalLatitude: 41.8596887; decimalLongitude: 12.4355075; geodeticDatum: WGS84; coordinatePrecision: 0.0002; **Identification:** identifiedBy: M. Mei; **Event:** eventDate: 2022-05-14; **Record Level:** collectionCode: UR3
- **Type status:** Other material. **Occurrence:** catalogNumber: A0416; recordedBy: L. Fortini; individualCount: 1; sex: female; lifeStage: adult; occurrenceID: 60D05E35-7C24-506E-A546-E1BA8D23BEAD; **Taxon:** scientificName: Osmia (Helicosmia) caerulescens (Linnaeus,1758); order: Hymenoptera; family: Megachilidae; genus: Osmia; subgenus: Helicosmia; specificEpithet: caerulescens; scientificNameAuthorship: (Linnaeus,1758); **Location:** country: Italy; countryCode: IT; stateProvince: Roma; locality: Riserva Regionale dell'Appia Antica 1; decimalLatitude: 41.8623941; decimalLongitude: 12.524863; geodeticDatum: WGS84; coordinatePrecision: 0.0002; **Identification:** identifiedBy: M. Mei; **Event:** eventDate: 2022-06-12; **Record Level:** collectionCode: UR3
- **Type status:** Other material. **Occurrence:** catalogNumber: A0417; recordedBy: L. Fortini; individualCount: 1; sex: female; lifeStage: adult; occurrenceID: 14245EEB-D963-52BA-AACA-F32B57C114B6; **Taxon:** scientificName: Osmia (Helicosmia) caerulescens (Linnaeus,1758); order: Hymenoptera; family: Megachilidae; genus: Osmia; subgenus: Helicosmia; specificEpithet: caerulescens; scientificNameAuthorship: (Linnaeus,1758); **Location:** country: Italy; countryCode: IT; stateProvince: Roma; locality: Riserva Naturale Valle dell'Aniene 1; decimalLatitude: 41.9345179; decimalLongitude: 12.5453096; geodeticDatum: WGS84; coordinatePrecision: 0.0002; **Identification:** identifiedBy: M. Mei; **Event:** eventDate: 2022-07-01; **Record Level:** collectionCode: UR3

#### Osmia cephalotes

Morawitz, 1870

87E51D4D-C6CB-520F-8CC7-A7EFF372663E

##### Materials

- **Type status:** Other material. **Occurrence:** catalogNumber: A0414; recordedBy: L. Fortini; individualCount: 1; sex: female; lifeStage: adult; occurrenceID: A5406B6B-E183-533B-94FA-8EB222395939; **Taxon:** scientificName: Osmia (Pyrosmia) cephalotes Morawitz, 1870; order: Hymenoptera; family: Megachilidae; genus: Osmia; subgenus: Pyrosmia; specificEpithet: cephalotes; scientificNameAuthorship: Morawitz, 1870; **Location:** country: Italy; countryCode: IT; stateProvince: Roma; locality: Riserva Naturale dell'Insugherata 2; decimalLatitude: 41.9599247; decimalLongitude: 12.433852; geodeticDatum: WGS84; coordinatePrecision: 0.0002; **Identification:** identifiedBy: M. Mei; **Event:** eventDate: 2022-05-27; **Record Level:** collectionCode: UR3

#### Osmia latreillei

(Spinola, 1806)

C5F0FFD2-FB4B-56CA-9626-79E4B485CB5A

##### Materials

- **Type status:** Other material. **Occurrence:** catalogNumber: A0408, A0409, A0410, A0411; recordedBy: L. Fortini; individualCount: 4; sex: females; lifeStage: adult; occurrenceID: 0A0CCF3B-2363-5DC9-B1F6-05CABD4B2670; **Taxon:** scientificName: Osmia (Helicosmia) latreillei (Spinola, 1806); order: Hymenoptera; family: Megachilidae; genus: Osmia; subgenus: Helicosmia; specificEpithet: latreillei; scientificNameAuthorship: (Spinola, 1806); **Location:** country: Italy; countryCode: IT; stateProvince: Roma; locality: Riserva Naturale Valle dell'Aniene 1; decimalLatitude: 41.9345179; decimalLongitude: 12.5453096; geodeticDatum: WGS84; coordinatePrecision: 0.0002; **Identification:** identifiedBy: M. Mei; **Event:** eventDate: 2022-04-28; **Record Level:** collectionCode: UR3
- **Type status:** Other material. **Occurrence:** catalogNumber: A0407; recordedBy: L. Fortini; individualCount: 1; sex: male; lifeStage: adult; occurrenceID: C647E15E-F9B4-5AFC-BB86-EDB03B99742F; **Taxon:** scientificName: Osmia (Helicosmia) latreillei (Spinola, 1806); order: Hymenoptera; family: Megachilidae; genus: Osmia; subgenus: Helicosmia; specificEpithet: latreillei; scientificNameAuthorship: (Spinola, 1806); **Location:** country: Italy; countryCode: IT; stateProvince: Roma; locality: Riserva Naturale Valle dell'Aniene 2; decimalLatitude: 41.928752; decimalLongitude: 12.5562962; geodeticDatum: WGS84; coordinatePrecision: 0.0002; **Identification:** identifiedBy: M. Mei; **Event:** eventDate: 2022-04-28; **Record Level:** collectionCode: UR3

#### Osmia leaiana

(Kirby, 1802)

469E6903-A9AA-5819-B254-705A155CFC3E

##### Materials

- **Type status:** Other material. **Occurrence:** catalogNumber: A0413; recordedBy: L. Fortini; individualCount: 1; sex: female; lifeStage: adult; occurrenceID: D3BD39BA-8992-568D-924F-D3B6D93A5095; **Taxon:** scientificName: Osmia (Helicosmia) leaiana (Kirby, 1802); order: Hymenoptera; family: Megachilidae; genus: Osmia; subgenus: Helicosmia; specificEpithet: leaiana; scientificNameAuthorship: (Kirby, 1802); **Location:** country: Italy; countryCode: IT; stateProvince: Roma; locality: Riserva Naturale Valle dei Casali 1; decimalLatitude: 41.8710627; decimalLongitude: 12.4336809; geodeticDatum: WGS84; coordinatePrecision: 0.0002; **Identification:** identifiedBy: M. Mei; **Event:** eventDate: 2022-07-13; **Record Level:** collectionCode: UR3

#### Osmia ligurica

Morawitz, 1868

06CD8F17-C564-5853-8241-4AABDD036EB6

##### Materials

- **Type status:** Other material. **Occurrence:** catalogNumber: A0659; recordedBy: L. Fortini; individualCount: 1; sex: male; lifeStage: adult; occurrenceID: F932FDB9-9995-5B06-BB98-638ABB181E8C; **Taxon:** scientificName: Osmia (Hoplosmia) ligurica Morawitz, 1868; order: Hymenoptera; family: Megachilidae; genus: Osmia; subgenus: Hoplosmia; specificEpithet: ligurica; scientificNameAuthorship: Morawitz, 1868; **Location:** country: Italy; countryCode: IT; stateProvince: Roma; locality: Riserva Regionale dell'Appia Antica 1; decimalLatitude: 41.8623941; decimalLongitude: 12.524863; geodeticDatum: WGS84; coordinatePrecision: 0.0002; **Identification:** identifiedBy: M. Mei; **Event:** eventDate: 2022-05-10; **Record Level:** collectionCode: UR3
- **Type status:** Other material. **Occurrence:** catalogNumber: A0662; recordedBy: L. Fortini; individualCount: 1; sex: female; lifeStage: adult; occurrenceID: 49AF52CE-4AB0-5122-A4EF-E7BCD70B9911; **Taxon:** scientificName: Osmia (Hoplosmia) ligurica Morawitz, 1868; order: Hymenoptera; family: Megachilidae; genus: Osmia; subgenus: Hoplosmia; specificEpithet: ligurica; scientificNameAuthorship: Morawitz, 1868; **Location:** country: Italy; countryCode: IT; stateProvince: Roma; locality: Riserva Naturale di Monte Mario; decimalLatitude: 41.9386215; decimalLongitude: 12.4546223; geodeticDatum: WGS84; coordinatePrecision: 0.0002; **Identification:** identifiedBy: M. Mei; **Event:** eventDate: 2022-05-20; **Record Level:** collectionCode: UR3

#### Osmia melanogaster

Spinola, 1808

32C2FFEB-779F-5900-8F42-4CEFE2E4F199

##### Materials

- **Type status:** Other material. **Occurrence:** catalogNumber: A0375; recordedBy: L. Fortini; individualCount: 1; sex: female; lifeStage: adult; occurrenceID: 1E3ABF07-F368-5658-B2E2-7C5DF75083D5; **Taxon:** scientificName: Osmia (Helicosmia) melanogaster Spinola, 1808; order: Hymenoptera; family: Megachilidae; genus: Osmia; subgenus: Helicosmia; specificEpithet: melanogaster; scientificNameAuthorship: Spinola, 1808; **Location:** country: Italy; countryCode: IT; stateProvince: Roma; locality: Riserva Naturale Laurentino-Acqua Acetosa; decimalLatitude: 41.8079275; decimalLongitude: 12.4685548; geodeticDatum: WGS84; coordinatePrecision: 0.0002; **Identification:** identifiedBy: M. Mei; **Event:** eventDate: 2022-05-12; **Record Level:** collectionCode: UR3

#### Osmia niveata

Fabricius, 1804

16A74B2B-A10C-58E3-A2F4-F45B33C79490

##### Materials

- **Type status:** Other material. **Occurrence:** catalogNumber: A0378, A0385; recordedBy: L. Fortini; individualCount: 2; sex: 1 male, 1 female; lifeStage: adult; occurrenceID: 59F1C0ED-6805-5784-80C3-EDA31DCEEE08; **Taxon:** scientificName: Osmia (Helicosmia) niveata (Fabricius, 1804); order: Hymenoptera; family: Megachilidae; genus: Osmia; subgenus: Helicosmia; specificEpithet: niveata; scientificNameAuthorship: (Fabricius, 1804); **Location:** country: Italy; countryCode: IT; stateProvince: Roma; locality: Riserva Regionale dell'Appia Antica 1; decimalLatitude: 41.8623941; decimalLongitude: 12.524863; geodeticDatum: WGS84; coordinatePrecision: 0.0002; **Identification:** identifiedBy: M. Mei; **Event:** eventDate: 2022-05-10; **Record Level:** collectionCode: UR3
- **Type status:** Other material. **Occurrence:** catalogNumber: A0396; recordedBy: L. Fortini; individualCount: 1; sex: female; lifeStage: adult; occurrenceID: 3861304C-606A-56E8-AE23-F622056F1138; **Taxon:** scientificName: Osmia (Helicosmia) niveata (Fabricius, 1804); order: Hymenoptera; family: Megachilidae; genus: Osmia; subgenus: Helicosmia; specificEpithet: niveata; scientificNameAuthorship: (Fabricius, 1804); **Location:** country: Italy; countryCode: IT; stateProvince: Roma; locality: Riserva Regionale dell'Appia Antica 3; decimalLatitude: 41.8298456; decimalLongitude: 12.5432538; geodeticDatum: WGS84; coordinatePrecision: 0.0002; **Identification:** identifiedBy: M. Mei; **Event:** eventDate: 2022-05-24; **Record Level:** collectionCode: UR3
- **Type status:** Other material. **Occurrence:** catalogNumber: A0381; recordedBy: L. Fortini; individualCount: 1; sex: female; lifeStage: adult; occurrenceID: 29DDF718-9D51-593F-BA5A-9AA86F4D39C6; **Taxon:** scientificName: Osmia (Helicosmia) niveata (Fabricius, 1804); order: Hymenoptera; family: Megachilidae; genus: Osmia; subgenus: Helicosmia; specificEpithet: niveata; scientificNameAuthorship: (Fabricius, 1804); **Location:** country: Italy; countryCode: IT; stateProvince: Roma; locality: Riserva Naturale dell'Insugherata 1; decimalLatitude: 41.9555045; decimalLongitude: 12.4292321; geodeticDatum: WGS84; coordinatePrecision: 0.0002; **Identification:** identifiedBy: M. Mei; **Event:** eventDate: 2022-06-24; **Record Level:** collectionCode: UR3
- **Type status:** Other material. **Occurrence:** catalogNumber: A0400; recordedBy: L. Fortini; individualCount: 1; sex: female; lifeStage: adult; occurrenceID: EBAB0AD4-6146-5C4B-B98C-463FB2D925B8; **Taxon:** scientificName: Osmia (Helicosmia) niveata (Fabricius, 1804); order: Hymenoptera; family: Megachilidae; genus: Osmia; subgenus: Helicosmia; specificEpithet: niveata; scientificNameAuthorship: (Fabricius, 1804); **Location:** country: Italy; countryCode: IT; stateProvince: Roma; locality: Riserva Naturale dell'Insugherata 1; decimalLatitude: 41.9555045; decimalLongitude: 12.4292321; geodeticDatum: WGS84; coordinatePrecision: 0.0002; **Identification:** identifiedBy: M. Mei; **Event:** eventDate: 2022-05-27; **Record Level:** collectionCode: UR3
- **Type status:** Other material. **Occurrence:** catalogNumber: A0404; recordedBy: L. Fortini; individualCount: 1; sex: female; lifeStage: adult; occurrenceID: 7B594063-2B85-51F8-993C-A50F80EBFDFB; **Taxon:** scientificName: Osmia (Helicosmia) niveata (Fabricius, 1804); order: Hymenoptera; family: Megachilidae; genus: Osmia; subgenus: Helicosmia; specificEpithet: niveata; scientificNameAuthorship: (Fabricius, 1804); **Location:** country: Italy; countryCode: IT; stateProvince: Roma; locality: Riserva Naturale dell'Insugherata 2; decimalLatitude: 41.9599247; decimalLongitude: 12.433852; geodeticDatum: WGS84; coordinatePrecision: 0.0002; **Identification:** identifiedBy: M. Mei; **Event:** eventDate: 2022-05-27; **Record Level:** collectionCode: UR3
- **Type status:** Other material. **Occurrence:** catalogNumber: A0397, A0398, A0401, A0405, A0406; recordedBy: L. Fortini; individualCount: 5; sex: females; lifeStage: adult; occurrenceID: A8F4E8BB-ACC4-5FC7-B187-C811A1DBDBF0; **Taxon:** scientificName: Osmia (Helicosmia) niveata (Fabricius, 1804); order: Hymenoptera; family: Megachilidae; genus: Osmia; subgenus: Helicosmia; specificEpithet: niveata; scientificNameAuthorship: (Fabricius, 1804); **Location:** country: Italy; countryCode: IT; stateProvince: Roma; locality: Riserva Naturale dell'Insugherata 3; decimalLatitude: 41.9644829; decimalLongitude: 12.436101; geodeticDatum: WGS84; coordinatePrecision: 0.0002; **Identification:** identifiedBy: M. Mei; **Event:** eventDate: 2022-05-27; **Record Level:** collectionCode: UR3
- **Type status:** Other material. **Occurrence:** catalogNumber: A0380, A0388, A0394, A0395, A0402, A0403; recordedBy: L. Fortini; individualCount: 6; sex: 1 male, 5 females; lifeStage: adult; occurrenceID: 59426B29-E740-52AA-A6A0-CACCF3BE664A; **Taxon:** scientificName: Osmia (Helicosmia) niveata (Fabricius, 1804); order: Hymenoptera; family: Megachilidae; genus: Osmia; subgenus: Helicosmia; specificEpithet: niveata; scientificNameAuthorship: (Fabricius, 1804); **Location:** country: Italy; countryCode: IT; stateProvince: Roma; locality: Riserva Naturale di Monte Mario; decimalLatitude: 41.9386215; decimalLongitude: 12.4546223; geodeticDatum: WGS84; coordinatePrecision: 0.0002; **Identification:** identifiedBy: M. Mei; **Event:** eventDate: 2022-05-20; **Record Level:** collectionCode: UR3
- **Type status:** Other material. **Occurrence:** catalogNumber: A0379; recordedBy: L. Fortini; individualCount: 1; sex: male; lifeStage: adult; occurrenceID: 56DBA81B-4914-50AC-80D8-E0623EBA86A7; **Taxon:** scientificName: Osmia (Helicosmia) niveata (Fabricius, 1804); order: Hymenoptera; family: Megachilidae; genus: Osmia; subgenus: Helicosmia; specificEpithet: niveata; scientificNameAuthorship: (Fabricius, 1804); **Location:** country: Italy; countryCode: IT; stateProvince: Roma; locality: Riserva Naturale Tenuta dei Massimi 2; decimalLatitude: 41.8316516; decimalLongitude: 12.3999927; geodeticDatum: WGS84; coordinatePrecision: 0.0002; **Identification:** identifiedBy: M. Mei; **Event:** eventDate: 2022-05-04; **Record Level:** collectionCode: UR3
- **Type status:** Other material. **Occurrence:** catalogNumber: A0376, A0386, A0389, A0391, A0393; recordedBy: L. Fortini; individualCount: 5; sex: 1 male, 4 females; lifeStage: adult; occurrenceID: F0DC214E-1B94-5AB9-BD5B-3CE7933E7038; **Taxon:** scientificName: Osmia (Helicosmia) niveata (Fabricius, 1804); order: Hymenoptera; family: Megachilidae; genus: Osmia; subgenus: Helicosmia; specificEpithet: niveata; scientificNameAuthorship: (Fabricius, 1804); **Location:** country: Italy; countryCode: IT; stateProvince: Roma; locality: Riserva Naturale Valle dell'Aniene 1; decimalLatitude: 41.9345179; decimalLongitude: 12.5453096; geodeticDatum: WGS84; coordinatePrecision: 0.0002; **Identification:** identifiedBy: M. Mei; **Event:** eventDate: 2022-04-28; **Record Level:** collectionCode: UR3
- **Type status:** Other material. **Occurrence:** catalogNumber: A0377, A0383, A0384, A0387, A0390, A0392, A0399; recordedBy: L. Fortini; individualCount: 7; sex: 1 male, 6 females; lifeStage: adult; occurrenceID: DB484478-EACD-5B84-9FE8-AF3D4CF2ECB1; **Taxon:** scientificName: Osmia (Helicosmia) niveata (Fabricius, 1804); order: Hymenoptera; family: Megachilidae; genus: Osmia; subgenus: Helicosmia; specificEpithet: niveata; scientificNameAuthorship: (Fabricius, 1804); **Location:** country: Italy; countryCode: IT; stateProvince: Roma; locality: Riserva Naturale Valle dei Casali 1; decimalLatitude: 41.8710627; decimalLongitude: 12.4336809; geodeticDatum: WGS84; coordinatePrecision: 0.0002; **Identification:** identifiedBy: M. Mei; **Event:** eventDate: 2022-05-14; **Record Level:** collectionCode: UR3
- **Type status:** Other material. **Occurrence:** catalogNumber: A0382; recordedBy: L. Fortini; individualCount: 1; sex: female; lifeStage: adult; occurrenceID: 9CF98BE5-C9F5-5415-92AE-BCDC6E4D92E7; **Taxon:** scientificName: Osmia (Helicosmia) niveata (Fabricius, 1804); order: Hymenoptera; family: Megachilidae; genus: Osmia; subgenus: Helicosmia; specificEpithet: niveata; scientificNameAuthorship: (Fabricius, 1804); **Location:** country: Italy; countryCode: IT; stateProvince: Roma; locality: Riserva Naturale Valle dei Casali 2; decimalLatitude: 41.8596887; decimalLongitude: 12.4355075; geodeticDatum: WGS84; coordinatePrecision: 0.0002; **Identification:** identifiedBy: M. Mei; **Event:** eventDate: 2022-05-14; **Record Level:** collectionCode: UR3

#### Osmia rufohirta

Latreille, 1811

838DA931-157A-51CE-B05B-D5420531ED2D

##### Materials

- **Type status:** Other material. **Occurrence:** catalogNumber: A0373, A0374; recordedBy: L. Fortini; individualCount: 2; sex: 1 male, 1 female; lifeStage: adult; occurrenceID: 09A5220E-56D8-5FE8-81EF-2414A5886AB9; **Taxon:** scientificName: Osmia (Allosmia) rufohirta Latreille, 1811; order: Hymenoptera; family: Megachilidae; genus: Osmia; subgenus: Allosmia; specificEpithet: rufohirta; scientificNameAuthorship: Latreille, 1811; **Location:** country: Italy; countryCode: IT; stateProvince: Roma; locality: Riserva Naturale Tenuta dei Massimi 2; decimalLatitude: 41.8316516; decimalLongitude: 12.3999927; geodeticDatum: WGS84; coordinatePrecision: 0.0002; **Identification:** identifiedBy: M. Mei; **Event:** eventDate: 2022-05-04; **Record Level:** collectionCode: UR3

#### Osmia scutellaris

Morawitz, 1868

A68435FB-78CA-5684-BE0B-1399AF225617

##### Materials

- **Type status:** Other material. **Occurrence:** catalogNumber: A0637, A0660; recordedBy: L. Fortini; individualCount: 2; sex: females; lifeStage: adult; occurrenceID: 8686CFDB-318C-5E68-9CE9-D4C0B93C244B; **Taxon:** scientificName: Osmia (Hoplosmia) scutellaris Morawitz, 1868; order: Hymenoptera; family: Megachilidae; genus: Osmia; subgenus: Hoplosmia; specificEpithet: scutellaris; scientificNameAuthorship: Morawitz, 1868; **Location:** country: Italy; countryCode: IT; stateProvince: Roma; locality: Riserva Naturale di Monte Mario; decimalLatitude: 41.9386215; decimalLongitude: 12.4546223; geodeticDatum: WGS84; coordinatePrecision: 0.0002; **Identification:** identifiedBy: M. Mei; **Event:** eventDate: 2022-05-20; **Record Level:** collectionCode: UR3

#### Osmia spinulosa

(Kirby, 1802)

3647201E-83B2-5C24-AE5E-B917E2DD591E

##### Materials

- **Type status:** Other material. **Occurrence:** catalogNumber: A0661; recordedBy: L. Fortini; individualCount: 1; sex: male; lifeStage: adult; occurrenceID: 9229E143-0527-5ADE-98E5-A3DF9ECD61E2; **Taxon:** scientificName: Osmia (Hoplosmia) spinulosa (Kirby,1802); order: Hymenoptera; family: Megachilidae; genus: Osmia; subgenus: Hoplosmia; specificEpithet: spinulosa; scientificNameAuthorship: (Kirby,1802); **Location:** country: Italy; countryCode: IT; stateProvince: Roma; locality: Riserva Naturale dell'Acquafredda; decimalLatitude: 41.8928408; decimalLongitude: 12.39932; geodeticDatum: WGS84; coordinatePrecision: 0.0002; **Identification:** identifiedBy: M. Mei; **Event:** eventDate: 2022-07-12; **Record Level:** collectionCode: UR3

#### Pseudoanthidium melanurum

(Klug, 1832)

3F2FA00E-0265-51AA-BD29-F26B5A11B13A

##### Materials

- **Type status:** Other material. **Occurrence:** catalogNumber: A0176; recordedBy: L. Fortini; individualCount: 1; sex: male; lifeStage: adult; occurrenceID: D5E691A5-A4A6-54AD-8272-48E0F7A4B9E4; **Taxon:** scientificName: Pseudoanthidium (Royanthidium) melanurum (Klug, 1832); order: Hymenoptera; family: Megachilidae; genus: Pseudoanthidium; subgenus: Royanthidium; specificEpithet: melanurum; scientificNameAuthorship: (Klug, 1832); **Location:** country: Italy; countryCode: IT; stateProvince: Roma; locality: Riserva Naturale Valle dei Casali 1; decimalLatitude: 41.8710627; decimalLongitude: 12.4336809; geodeticDatum: WGS84; coordinatePrecision: 0.0002; **Identification:** identifiedBy: M. Mei; **Event:** eventDate: 2022-06-18; **Record Level:** collectionCode: UR3
- **Type status:** Other material. **Occurrence:** catalogNumber: A0177; recordedBy: L. Fortini; individualCount: 1; sex: male; lifeStage: adult; occurrenceID: 4A7D57D4-B889-5414-A12A-4ADD18148214; **Taxon:** scientificName: Pseudoanthidium (Royanthidium) melanurum (Klug, 1832); order: Hymenoptera; family: Megachilidae; genus: Pseudoanthidium; subgenus: Royanthidium; specificEpithet: melanurum; scientificNameAuthorship: (Klug, 1832); **Location:** country: Italy; countryCode: IT; stateProvince: Roma; locality: Riserva Naturale Valle dei Casali 1; decimalLatitude: 41.8710627; decimalLongitude: 12.4336809; geodeticDatum: WGS84; coordinatePrecision: 0.0002; **Identification:** identifiedBy: M. Mei; **Event:** eventDate: 2022-05-14; **Record Level:** collectionCode: UR3
- **Type status:** Other material. **Occurrence:** catalogNumber: A0178; recordedBy: L. Fortini; individualCount: 1; sex: female; lifeStage: adult; occurrenceID: F5B05EA9-1E0D-5677-8B35-04A5DA9E34B0; **Taxon:** scientificName: Pseudoanthidium (Royanthidium) melanurum (Klug, 1832); order: Hymenoptera; family: Megachilidae; genus: Pseudoanthidium; subgenus: Royanthidium; specificEpithet: melanurum; scientificNameAuthorship: (Klug, 1832); **Location:** country: Italy; countryCode: IT; stateProvince: Roma; locality: Riserva Naturale Laurentino-Acqua Acetosa; decimalLatitude: 41.8079275; decimalLongitude: 12.4685548; geodeticDatum: WGS84; coordinatePrecision: 0.0002; **Identification:** identifiedBy: M. Mei; **Event:** eventDate: 2022-05-12; **Record Level:** collectionCode: UR3

#### Rhodanthidium septemdentatum

(Latreille, 1809)

841C539D-62BA-5467-BD38-7182242394CF

##### Materials

- **Type status:** Other material. **Occurrence:** catalogNumber: A0164; recordedBy: L. Fortini; individualCount: 1; sex: male; lifeStage: adult; occurrenceID: 997085B8-A53A-5AE0-B63F-C577874905B2; **Taxon:** scientificName: Rhodanthidium (Rhodanthidium) septemdentatum (Latreille, 1809); order: Hymenoptera; family: Megachilidae; genus: Rhodanthidium; subgenus: Rhodanthidium; specificEpithet: septemdentatum; scientificNameAuthorship: (Latreille, 1809); **Location:** country: Italy; countryCode: IT; stateProvince: Roma; locality: Riserva Regionale dell'Appia Antica 2; decimalLatitude: 41.8402564; decimalLongitude: 12.532773; geodeticDatum: WGS84; coordinatePrecision: 0.0002; **Identification:** identifiedBy: M. Mei; **Event:** eventDate: 2022-05-24; **Record Level:** collectionCode: UR3
- **Type status:** Other material. **Occurrence:** catalogNumber: A0166; recordedBy: L. Fortini; individualCount: 1; sex: male; lifeStage: adult; occurrenceID: B102BB5B-BD73-54F2-9A5A-EC8DAF7D3F26; **Taxon:** scientificName: Rhodanthidium (Rhodanthidium) septemdentatum (Latreille, 1809); order: Hymenoptera; family: Megachilidae; genus: Rhodanthidium; subgenus: Rhodanthidium; specificEpithet: septemdentatum; scientificNameAuthorship: (Latreille, 1809); **Location:** country: Italy; countryCode: IT; stateProvince: Roma; locality: Riserva Regionale dell'Appia Antica 1; decimalLatitude: 41.8623941; decimalLongitude: 12.524863; geodeticDatum: WGS84; coordinatePrecision: 0.0002; **Identification:** identifiedBy: M. Mei; **Event:** eventDate: 2022-05-10; **Record Level:** collectionCode: UR3
- **Type status:** Other material. **Occurrence:** catalogNumber: A0167; recordedBy: L. Fortini; individualCount: 1; sex: male; lifeStage: adult; occurrenceID: 7A6C6C42-FCFF-502D-A071-12BCF9C95D5A; **Taxon:** scientificName: Rhodanthidium (Rhodanthidium) septemdentatum (Latreille, 1809); order: Hymenoptera; family: Megachilidae; genus: Rhodanthidium; subgenus: Rhodanthidium; specificEpithet: septemdentatum; scientificNameAuthorship: (Latreille, 1809); **Location:** country: Italy; countryCode: IT; stateProvince: Roma; locality: Riserva Regionale dell'Appia Antica 1; decimalLatitude: 41.8623941; decimalLongitude: 12.524863; geodeticDatum: WGS84; coordinatePrecision: 0.0002; **Identification:** identifiedBy: M. Mei; **Event:** eventDate: 2022-06-12; **Record Level:** collectionCode: UR3
- **Type status:** Other material. **Occurrence:** catalogNumber: A0163, A0168; recordedBy: L. Fortini; individualCount: 2; sex: male; lifeStage: adult; occurrenceID: 12986F23-3505-5FD4-B413-D9A999BFEA50; **Taxon:** scientificName: Rhodanthidium (Rhodanthidium) septemdentatum (Latreille, 1809); order: Hymenoptera; family: Megachilidae; genus: Rhodanthidium; subgenus: Rhodanthidium; specificEpithet: septemdentatum; scientificNameAuthorship: (Latreille, 1809); **Location:** country: Italy; countryCode: IT; stateProvince: Roma; locality: Riserva Naturale Laurentino-Acqua Acetosa; decimalLatitude: 41.8079275; decimalLongitude: 12.4685548; geodeticDatum: WGS84; coordinatePrecision: 0.0002; **Identification:** identifiedBy: M. Mei; **Event:** eventDate: 2022-06-16; **Record Level:** collectionCode: UR3
- **Type status:** Other material. **Occurrence:** catalogNumber: A0171; recordedBy: L. Fortini; individualCount: 1; sex: female; lifeStage: adult; occurrenceID: 364697BC-8EF8-5C7A-A6E1-3190F5872F60; **Taxon:** scientificName: Rhodanthidium (Rhodanthidium) septemdentatum (Latreille, 1809); order: Hymenoptera; family: Megachilidae; genus: Rhodanthidium; subgenus: Rhodanthidium; specificEpithet: septemdentatum; scientificNameAuthorship: (Latreille, 1809); **Location:** country: Italy; countryCode: IT; stateProvince: Roma; locality: Riserva Naturale Laurentino-Acqua Acetosa; decimalLatitude: 41.8079275; decimalLongitude: 12.4685548; geodeticDatum: WGS84; coordinatePrecision: 0.0002; **Identification:** identifiedBy: M. Mei; **Event:** eventDate: 2022-05-12; **Record Level:** collectionCode: UR3
- **Type status:** Other material. **Occurrence:** catalogNumber: A0165; recordedBy: L. Fortini; individualCount: 1; sex: male; lifeStage: adult; occurrenceID: 2DDE89F9-4254-56A6-8386-7C581B03E670; **Taxon:** scientificName: Rhodanthidium (Rhodanthidium) septemdentatum (Latreille, 1809); order: Hymenoptera; family: Megachilidae; genus: Rhodanthidium; subgenus: Rhodanthidium; specificEpithet: septemdentatum; scientificNameAuthorship: (Latreille, 1809); **Location:** country: Italy; countryCode: IT; stateProvince: Roma; locality: Riserva Naturale di Monte Mario; decimalLatitude: 41.9386215; decimalLongitude: 12.4546223; geodeticDatum: WGS84; coordinatePrecision: 0.0002; **Identification:** identifiedBy: M. Mei; **Event:** eventDate: 2022-06-19; **Record Level:** collectionCode: UR3
- **Type status:** Other material. **Occurrence:** catalogNumber: A0162; recordedBy: L. Fortini; individualCount: 1; sex: male; lifeStage: adult; occurrenceID: 5317D046-18EC-5078-9EBB-C770411856D5; **Taxon:** scientificName: Rhodanthidium (Rhodanthidium) septemdentatum (Latreille, 1809); order: Hymenoptera; family: Megachilidae; genus: Rhodanthidium; subgenus: Rhodanthidium; specificEpithet: septemdentatum; scientificNameAuthorship: (Latreille, 1809); **Location:** country: Italy; countryCode: IT; stateProvince: Roma; locality: Riserva Naturale Valle dell'Aniene 2; decimalLatitude: 41.928752; decimalLongitude: 12.5562962; geodeticDatum: WGS84; coordinatePrecision: 0.0002; **Identification:** identifiedBy: M. Mei; **Event:** eventDate: 2022-06-05; **Record Level:** collectionCode: UR3
- **Type status:** Other material. **Occurrence:** catalogNumber: A0169, A0170; recordedBy: L. Fortini; individualCount: 2; sex: 1 male, 1 female; lifeStage: adult; occurrenceID: 57626599-DF95-5C7B-BC74-42B8E9048BED; **Taxon:** scientificName: Rhodanthidium (Rhodanthidium) septemdentatum (Latreille, 1809); order: Hymenoptera; family: Megachilidae; genus: Rhodanthidium; subgenus: Rhodanthidium; specificEpithet: septemdentatum; scientificNameAuthorship: (Latreille, 1809); **Location:** country: Italy; countryCode: IT; stateProvince: Roma; locality: Riserva Naturale Valle dell'Aniene 1; decimalLatitude: 41.9345179; decimalLongitude: 12.5453096; geodeticDatum: WGS84; coordinatePrecision: 0.0002; **Identification:** identifiedBy: M. Mei; **Event:** eventDate: 2022-07-01; **Record Level:** collectionCode: UR3

#### Trachusa integra

(Eversmann, 1852)

3A09A79D-7AFA-599C-8117-7F1F912E2BE6

##### Materials

- **Type status:** Other material. **Occurrence:** catalogNumber: A0174; recordedBy: L. Fortini; individualCount: 1; sex: male; lifeStage: adult; occurrenceID: A6C36ACB-3331-55AE-927A-25262B76894E; **Taxon:** scientificName: Trachusa (Paraanthidium) integra (Eversmann, 1852); order: Hymenoptera; family: Megachilidae; genus: Trachusa; subgenus: Paraanthidium; specificEpithet: integra; scientificNameAuthorship: (Eversmann, 1852); **Location:** country: Italy; countryCode: IT; stateProvince: Roma; locality: Riserva Naturale Tenuta dei Massimi 2; decimalLatitude: 41.8316516; decimalLongitude: 12.3999927; geodeticDatum: WGS84; coordinatePrecision: 0.0002; **Identification:** identifiedBy: M. Mei; **Event:** eventDate: 2022-07-01; **Record Level:** collectionCode: UR3
- **Type status:** Other material. **Occurrence:** catalogNumber: A0175; recordedBy: L. Fortini; individualCount: 1; sex: male; lifeStage: adult; occurrenceID: 27170E4C-8156-50D9-BCB7-EA5B31E4D0BD; **Taxon:** scientificName: Trachusa (Paraanthidium) integra (Eversmann, 1852); order: Hymenoptera; family: Megachilidae; genus: Trachusa; subgenus: Paraanthidium; specificEpithet: integra; scientificNameAuthorship: (Eversmann, 1852); **Location:** country: Italy; countryCode: IT; stateProvince: Roma; locality: Riserva Naturale dell'Insugherata 3; decimalLatitude: 41.9644829; decimalLongitude: 12.436101; geodeticDatum: WGS84; coordinatePrecision: 0.0002; **Identification:** identifiedBy: M. Mei; **Event:** eventDate: 2022-05-27; **Record Level:** collectionCode: UR3

#### Trachusa interrupta

(Fabricius, 1781)

4EB49B09-39F8-5165-8807-3794028CACD8

##### Materials

- **Type status:** Other material. **Occurrence:** catalogNumber: A0172; recordedBy: L. Fortini; individualCount: 1; sex: male; lifeStage: adult; occurrenceID: D5E94FEA-F3B6-5FD8-AD09-C115C8DA2F1D; **Taxon:** scientificName: Trachusa (Paraanthidium) interrupta (Fabricius, 1781); order: Hymenoptera; family: Megachilidae; genus: Trachusa; subgenus: Paraanthidium; specificEpithet: interrupta; scientificNameAuthorship: (Fabricius, 1781); **Location:** country: Italy; countryCode: IT; stateProvince: Roma; locality: Riserva Naturale di Monte Mario; decimalLatitude: 41.9386215; decimalLongitude: 12.4546223; geodeticDatum: WGS84; coordinatePrecision: 0.0002; **Identification:** identifiedBy: M. Mei; **Event:** eventDate: 2022-06-19; **Record Level:** collectionCode: UR3
- **Type status:** Other material. **Occurrence:** catalogNumber: A0173; recordedBy: L. Fortini; individualCount: 1; sex: female; lifeStage: adult; occurrenceID: 6E412B0C-FD82-5A58-B8CE-A8823A28DF6C; **Taxon:** scientificName: Trachusa (Paraanthidium) interrupta (Fabricius, 1781); order: Hymenoptera; family: Megachilidae; genus: Trachusa; subgenus: Paraanthidium; specificEpithet: interrupta; scientificNameAuthorship: (Fabricius, 1781); **Location:** country: Italy; countryCode: IT; stateProvince: Roma; locality: Riserva Regionale dell'Appia Antica 1; decimalLatitude: 41.8623941; decimalLongitude: 12.524863; geodeticDatum: WGS84; coordinatePrecision: 0.0002; **Identification:** identifiedBy: M. Mei; **Event:** eventDate: 2022-06-12; **Record Level:** collectionCode: UR3

### Melittidae Michener, 2000

#### Dasypoda hirtipes

(Fabricius, 1793)

176C8443-7866-5D55-B133-8C3C59A05BFA

##### Materials

- **Type status:** Other material. **Occurrence:** catalogNumber: A0218, A0220, A0240; recordedBy: L. Fortini; individualCount: 3; sex: 2 males, 1 female; lifeStage: adult; occurrenceID: F5B9DE86-9BCC-5F15-8D83-45C2744694DA; **Taxon:** scientificName: Dasypoda (Dasypoda) hirtipes (Fabricius, 1793); order: Hymenoptera; family: Melittidae; genus: Dasypoda; subgenus: Dasypoda; specificEpithet: hirtipes; scientificNameAuthorship: (Fabricius, 1793); **Location:** country: Italy; countryCode: IT; stateProvince: Roma; locality: Riserva Naturale dell'Acquafredda; decimalLatitude: 41.892841; decimalLongitude: 12.399320; geodeticDatum: WGS84; coordinatePrecision: 0.0002; **Identification:** identifiedBy: L. Fortini; **Event:** eventDate: 2022-06-10; **Record Level:** collectionID: UR3
- **Type status:** Other material. **Occurrence:** catalogNumber: A0221, A0222, A0223, A0224, A0225, A0226, A0227, A0228, A0229, A0230, A0231, A0234, A0235, A0236, A0237, A0239; recordedBy: L. Fortini; individualCount: 16; sex: 11 males, 5 females; lifeStage: adult; occurrenceID: 1FA9B15F-1EF6-5B1A-8258-19051198CCF3; **Taxon:** scientificName: Dasypoda (Dasypoda) hirtipes (Fabricius, 1793); order: Hymenoptera; family: Melittidae; genus: Dasypoda; subgenus: Dasypoda; specificEpithet: hirtipes; scientificNameAuthorship: (Fabricius, 1793); **Location:** country: Italy; countryCode: IT; stateProvince: Roma; locality: Riserva Naturale dell'Acquafredda; decimalLatitude: 41.892841; decimalLongitude: 12.399320; geodeticDatum: WGS84; coordinatePrecision: 0.0002; **Identification:** identifiedBy: L. Fortini; **Event:** eventDate: 2022-07-12; **Record Level:** collectionID: UR3
- **Type status:** Other material. **Occurrence:** catalogNumber: A0219; recordedBy: L. Fortini; individualCount: 1; sex: male; lifeStage: adult; occurrenceID: 4EE88335-CE8B-5FA0-B73A-576638134B73; **Taxon:** scientificName: Dasypoda (Dasypoda) hirtipes (Fabricius, 1793); order: Hymenoptera; family: Melittidae; genus: Dasypoda; subgenus: Dasypoda; specificEpithet: hirtipes; scientificNameAuthorship: (Fabricius, 1793); **Location:** country: Italy; countryCode: IT; stateProvince: Roma; locality: Riserva Naturale dell'Insugherata 2; decimalLatitude: 41.959925; decimalLongitude: 12.433852; geodeticDatum: WGS84; coordinatePrecision: 0.0002; **Identification:** identifiedBy: L. Fortini; **Event:** eventDate: 2022-07-30; **Record Level:** collectionID: UR3
- **Type status:** Other material. **Occurrence:** catalogNumber: A0238; recordedBy: L. Fortini; individualCount: 1; sex: female; lifeStage: adult; occurrenceID: C739AE5B-5E3E-5BBA-A50B-810501370B82; **Taxon:** scientificName: Dasypoda (Dasypoda) hirtipes (Fabricius, 1793); order: Hymenoptera; family: Melittidae; genus: Dasypoda; subgenus: Dasypoda; specificEpithet: hirtipes; scientificNameAuthorship: (Fabricius, 1793); **Location:** country: Italy; countryCode: IT; stateProvince: Roma; locality: Riserva Naturale dell'Insugherata 3; decimalLatitude: 41.964483; decimalLongitude: 12.436101; geodeticDatum: WGS84; coordinatePrecision: 0.0002; **Identification:** identifiedBy: L. Fortini; **Event:** eventDate: 2022-07-30; **Record Level:** collectionID: UR3
- **Type status:** Other material. **Occurrence:** catalogNumber: A0217; recordedBy: L. Fortini; individualCount: 1; sex: male; lifeStage: adult; occurrenceID: 63556E66-BC55-58F2-94F3-C1D1457E8C8F; **Taxon:** scientificName: Dasypoda (Dasypoda) hirtipes (Fabricius, 1793); order: Hymenoptera; family: Melittidae; genus: Dasypoda; subgenus: Dasypoda; specificEpithet: hirtipes; scientificNameAuthorship: (Fabricius, 1793); **Location:** country: Italy; countryCode: IT; stateProvince: Roma; locality: Riserva Naturale Laurentino-Acqua Acetosa; decimalLatitude: 41.807927; decimalLongitude: 12.468555; geodeticDatum: WGS84; coordinatePrecision: 0.0002; **Identification:** identifiedBy: L. Fortini; **Event:** eventDate: 2022-09-14; **Record Level:** collectionID: UR3
- **Type status:** Other material. **Occurrence:** catalogNumber: A0232; recordedBy: L. Fortini; individualCount: 1; sex: female; lifeStage: adult; occurrenceID: C04987AF-E1C9-57D1-BE64-3BEB2C6DF18E; **Taxon:** scientificName: Dasypoda (Dasypoda) hirtipes (Fabricius, 1793); order: Hymenoptera; family: Melittidae; genus: Dasypoda; subgenus: Dasypoda; specificEpithet: hirtipes; scientificNameAuthorship: (Fabricius, 1793); **Location:** country: Italy; countryCode: IT; stateProvince: Roma; locality: Riserva Naturale Laurentino-Acqua Acetosa; decimalLatitude: 41.807927; decimalLongitude: 12.468555; geodeticDatum: WGS84; coordinatePrecision: 0.0002; **Identification:** identifiedBy: L. Fortini; **Event:** eventDate: 2022-06-16; **Record Level:** collectionID: UR3
- **Type status:** Other material. **Occurrence:** catalogNumber: A0233; recordedBy: L. Fortini; individualCount: 1; sex: female; lifeStage: adult; occurrenceID: E8A05375-6B34-50CC-87F9-6BBBD6D905C3; **Taxon:** scientificName: Dasypoda (Dasypoda) hirtipes (Fabricius, 1793); order: Hymenoptera; family: Melittidae; genus: Dasypoda; subgenus: Dasypoda; specificEpithet: hirtipes; scientificNameAuthorship: (Fabricius, 1793); **Location:** country: Italy; countryCode: IT; stateProvince: Roma; locality: Riserva Naturale Tenuta dei Massimi 1; decimalLatitude: 41.8532859; decimalLongitude: 12.3842322; geodeticDatum: WGS84; coordinatePrecision: 0.0002; **Identification:** identifiedBy: L. Fortini; **Event:** eventDate: 2022-06-27; **Record Level:** collectionID: UR3

## Analysis

**Figure 2.**
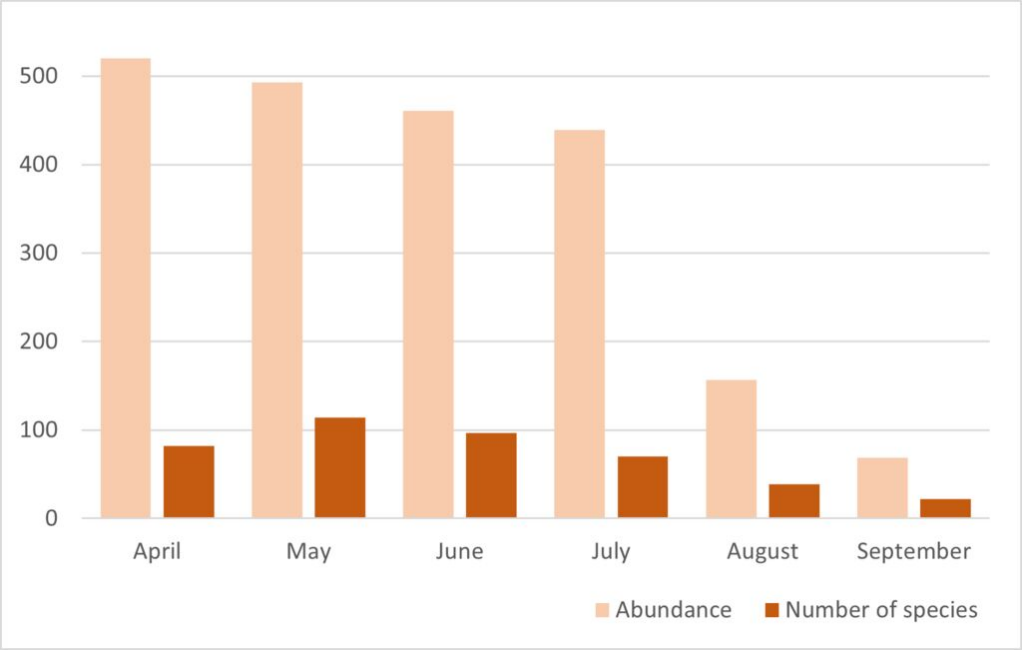
Total abundances and number of species of Apoidea collected during each sampling session, from April to September, in the urban Nature Reserves of Rome, Italy.

The survey allowed the collection of 2139 specimens (spc.) of Apoidea belonging to 208 species (spp.) in 36 genera and six different families. Amongst the areas we investigated, APP presented both the greatest number of specimens and the greatest number of species collected (448 spc., 110 spp.), followed by INS (329 spc., 93 spp.), VDC (278 spc., 84 spp.), VAN (313 spc., 83 spp.), TDM (269 spc., 73 spp.), LAU (145 spc., 68 spp.), MMA (186 spc., 58 spp.) and ACQ (171 spc., 53 spp.). LAU presented the smallest number of specimens, whereas ACQ presented the lowest number of species (Table 1). The most abundant species were *Halictusscabiosae* (Rossi, 1790) (148 spc.), *Lasioglossummalachurum* (Kirby, 1802) (110 spc.) and *Bombuspascuorum* (Scopoli, 1763) (63 spc.). The species we recorded in the nature reserves selected for the study represent approximately 74% of the known bee species for the area within the GRA of Rome since 1997, 46% of the Latium Region and approximately 20% of Italy. In April, the first month of sampling, we collected the greatest number of bees compared to the other months, while we observed a clear decrease in abundance in August and September compared with the previous months. We found the highest number of species in May (114 spp.), while we found the lowest number of species in September (22 spp.) (Fig. 2).

Halictidae is the most common family, with 788 spc. representing 36% of the samples (Fig. 3a) belonging to 40 species and three genera (19% of the total species) (Fig. 3b). This bee family is the most abundant in six out of the eight nature reserves, except for INS and LAU (Fig. 4a). Apidae counts 505 spc., representing 24% of the samples (Fig. 3a) and belonging to 48 species and 11 genera (23% of the total species) (Fig. 3b). Apidae is the most abundant family in INS and LAU (Fig. 4a). Andrenidae represents 17% of the samples (Fig. 3a), belonging to 48 species and two genera (23% of the total species) (Fig. 3b). Megachilidae represents 15% of the samples (Fig. 3a), belonging to 52 species and 14 genera (25% of the total species) (Fig. 3b). Colletidae counts 148 spc., representing 7% of the samples (Fig. 3a) and belonging to 19 species and two genera (10% of the total species) (Fig. 3b). Mellittidae is represented just by *Dasypodahirtipes* (Fabricius, 1793). It represents 1% of the total samples (Fig. 3a). We did not collect Mellittidae from APP, MMA or VDC, whereas, in ACQ, it represented 11% of all the samples (Fig. 4). We found eight species included in the European Wild Bees Red List. One species, *Trachusainterrupta* (Fabricius, 1781), is classified in the category Endangered (EN) and seven species are in the category Near Threatened (NT) (Table 2). Twenty-four species amongst those collected are new records for the urban area of Rome (Table 3).

## Discussion

Although the entire experiment and sampling took place over a single year, carried out in eight green areas, using only one collection method (entomological net along fixed transects) and performed by only one operator (LF), we collected 208 species, representing 74% of the wild bee species reported within the urban area of Rome (Comba and Comba 1997), 46% of the Latium Region (Comba and Comba 1991) and 20% of the species reported for Italy (Lhomme et al. 2020, Wood et al. 2023, Cornalba et al. 2024). The percentage of species detected in this experiment, relative to all those cumulative and historically reported for the study area and the addition of twenty-four new taxa represents, in our opinion, a good achievement, showing that an intensive sampling effort may yield new results regarding the distribution of wild bees in southern Europe. To highlight the effectiveness of the work and show with as little approximation as possible the state of the diversity of bees, it is possible to compare the methodological strategy and faunal results reported with the recent article by Geppert et al. (2022), which represents a landmark study on the topic of wild bees in Rome from an ecological point of view. The two sampling strategies are not comparable in terms of collection effort, but they provide an idea of what may be the best approach to use if the goal is to study the alpha diversity of an area. The work of Geppert et al. (2022) resulted in the collection of 3280 wild bees belonging to 96 species, representing 34.5% of the species reported in the urban area of Rome. Geppert's data were collected during four months of sampling, which was carried out at thirty-six sites of different sizes within the GRA and using yellow pan traps. Compared with passive sampling methods such as yellow pan traps, active sampling using entomological netting more exhaustively captures alpha diversity, but results in a bias in the abundances of the groups collected because it is highly dependent on sampler capabilities (Westphal et al. 2008). In this study, we collected data over two more months (April and May), but in only eight large green areas and we collected more than twice the number of species recorded by Geppert et al. (2022). In this way, we provide a recent snapshot of the presence and distribution of wild bee species in the nature reserves of Rome, representing a starting point for monitoring them in the future. In addition, not all the species collected were reported for the area; in fact, twenty-four species (11% of the spp. collected) are new reports for the urban area of Rome.

In terms of the abundance data, April was the month in which the greatest number of bees were collected overall (Fig. 2). As we expected, from April to July, wild bees were more abundant than they were in August and September, the months in which a significant decrease in abundance was evident. The peak in the species diversity was detected during the May session (114 spp.) and declined steadily until September when only 22 species were detected. In April, the first month of sampling, the number of species detected already amounted to 82. Flower plants provide sustenance to bees and almost all herbaceous plants observed during sampling had completed their flowering period by August. The sampling period was selected according to the guidelines given in the literature for sampling pollinating insects in the Mediterranean environment (Westphal et al. 2008), but in April, the peak of abundance for wild bees may have already been reached and a relatively good number of species is already active. The “heat island effect” produced by large urban centres and the average warmer and drier climate cause pollinators to be active early. For this reason, it would probably be correct to start sampling for pollinator insect studies in early March or even mid-February to obtain more complete information on the presence and abundance of species with early phenology.

Amongst those species we collected, eight are included in the European Red List of bees (Nieto et al. 2014). The presence of Red-listed species within the city shows that large urban green areas can be a refuge not only for wild bee species with a generalist ecology, but also for species with a more restricted ecology and that the nature reserves of Rome allow the survival of rarer species. The work of Fusco et al. (2024), for example, studied ground spider diversity in APP itself and showed that it is true for other groups of arthropods. Focusing on APP, it is the largest urban nature reserve in Europe. It represents a unique case, being a single continuous green area that extends from the city centre to the area outside the city. In this way, APP offers ecological continuity and hosts an exceptionally high biodiversity of botanical species (Iamonico 2022). The diversity of plants in the area is due to the presence of spontaneous, non-native and ornamental herbaceous and shrubby species in villas and home gardens distributed throughout the Reserve. Many plant species can have many different flowering periods that overlap. It can ensure continuity in the supply of resources for pollinators and spontaneous plants, promoting more functionally diverse bee communities (Zaninotto et al. 2023).

This study revealed that, given its unique combination of natural and semi-natural elements embedded in the urban matrix, Rome hosts and offers shelter to a remarkable variety of Apoidea Anthophila, including multiple threatened species. Notably, the bee community was probably not subject to any particular erosion in terms of richness between 1997 and 2022, indicating that the current complexity and repartition of green areas within or at the margin of Rome possibly buffered some of the drivers causing pollinators to decline. However, from a conservation perspective, the protection of urban fauna has to pass through the adoption of correct green management and planning strategies. Furthermore, faunistic surveys aimed at investigating bee diversity in urban ecosystems should consider the ‘heat island effect’ that occurs in large urban centres, a phenomenon that affects bee phenology and enhances flowering in plants in an urban context. In this regard, an earlier sampling period in February and March would be desirable to intercept the more precocious species, which we probably did not encounter in the present survey.

## Figures and Tables

**Figure 3a.**
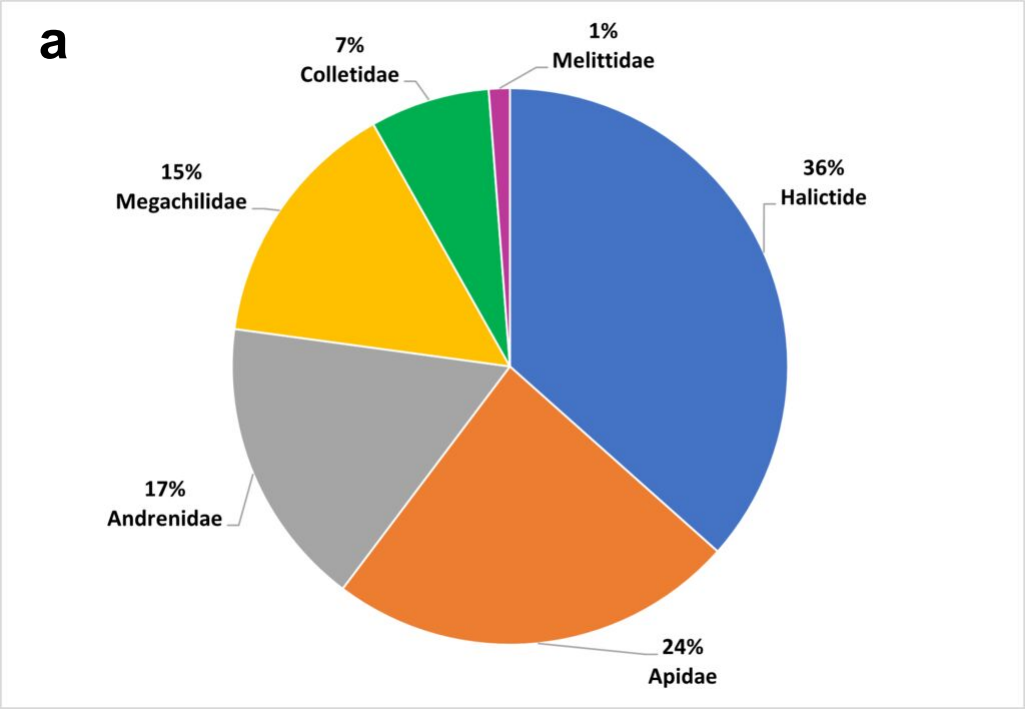
Abundance (% of sampled individuals) of wild bees per family;

**Figure 3b.**
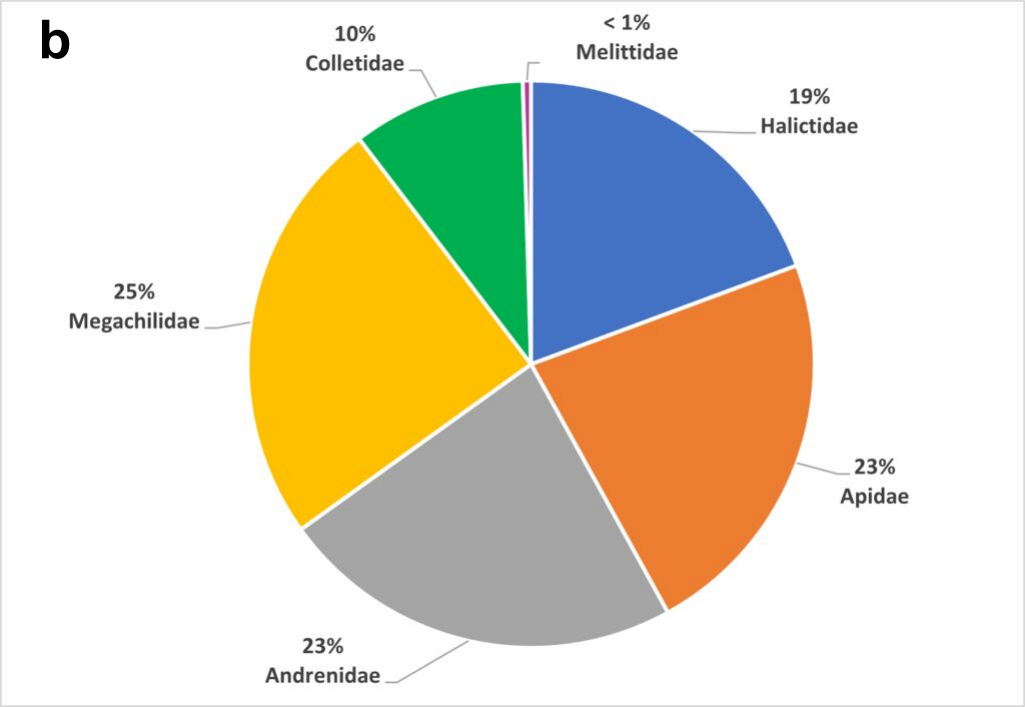
Taxonomic composition (% of species) of wild bees per family.

**Figure 4a.**
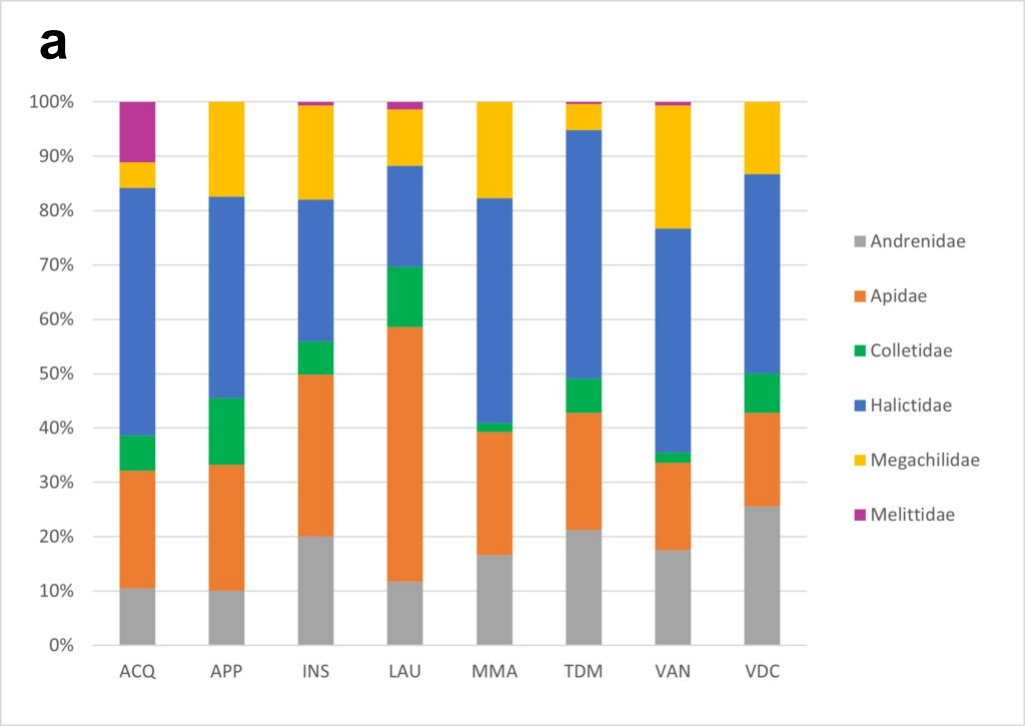
Abundance (% of sampled individuals) of wild bees per family;

**Figure 4b.**
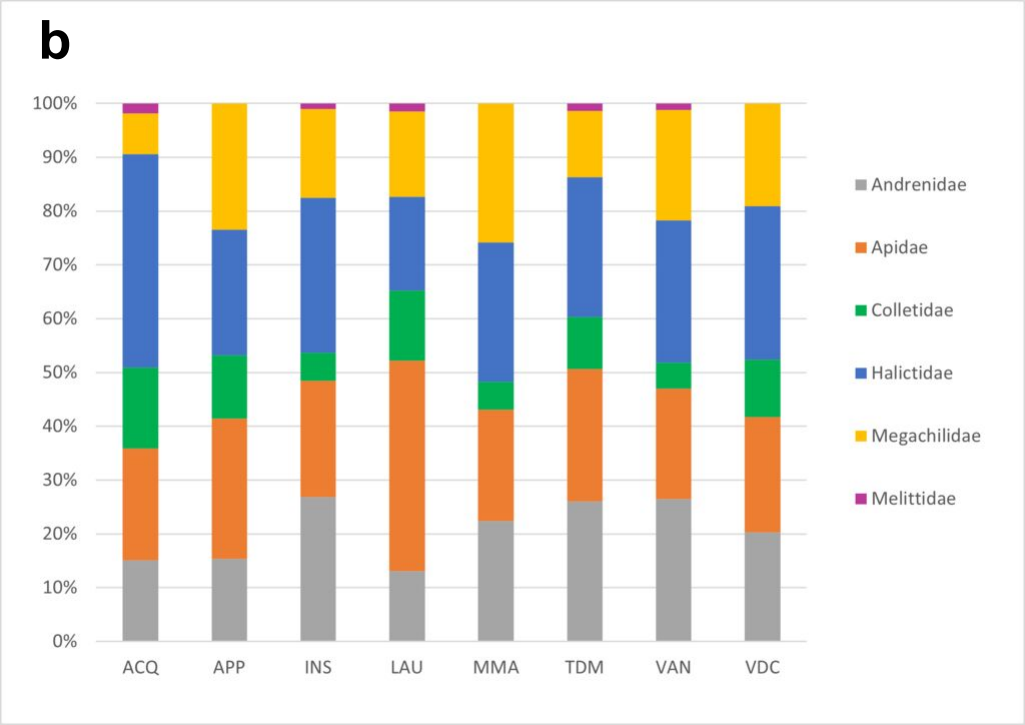
Taxonomic composition (% of species) of wild bees per family.

**Table 1.** Absolute abundance and number of species collected in the different urban Nature Reserves of Rome, Italy and relative sampling effort made.

**Green area**	**Abbreviation**	**n. transects**	**n. specimens**	**n. species**
Parco Regionale dell'Appia Antica	APP	3	448	110
Riserva Naturale dell'Insugherata	INS	3	329	93
Riserva Naturale Valle dei Casali	VDC	2	278	84
Riserva Naturale Valle dell'Aniene	VAN	2	313	83
Riserva Naturale Tenuta dei Massimi	TDM	2	269	73
Riserva Naturale Laurentino-Acqua Acetosa	LAU	1	145	68
Riserva Naturale di Monte Mario	MMA	1	186	58
Riserva Naturale dell'Acquafredda	ACQ	1	171	53

**Table 2.** List of species of Hymenoptera Apoidea collected included in the European Red List (Nieto et al. 2014) and the Italian Red List (Quaranta et al. 2018) of wild bees with a risk classification equal or higher than Least Concern (LC) and sites where they were recorded.

Family	Species	status EU	status IT	Area of recording
Andrenidae	*Andrenahattorfiana* Fabricius, 1775	NT	DD	INS
Apidae	*Epeoluscruciger* (Panzer, 1799)	NT	DD	MMA; TDM
Apidae	*Nomadaargentata* Herrich-Schäffer, 1839	NT	NT	APP
Halictidae	*Halictusquadricinctus* (Fabricius, 1776)	NT	DD	APP; INS; LAU; VAN; VDC
Halictidae	*Lasioglossumbrevicorne* (Schenck, 1868)	NT	DD	LAU; VDC
Halictidae	*Lasioglossumpygmaeum* (Schenck, 1853)	NT	DD	APP; MMA
Halictidae	*Systrophacurvicornis* (Scopoli, 1770)	NT	DD	ACQ; INS; VDC
Megachilidae	*Trachusainterrupta* (Fabricius, 1781)	EN	DD	APP; MMA

**Table 3.** List of Hymenoptera Apoidea collected that are new records for the Rome area.

**Family**	**Species**	**Area of recording**
Andrenidae	*Andrenabraunsiana* Friese, 1887	INS
Apidae	*Nomadabasalis* Herrich-Schäffer, 1839	LAU; TDM; VDC
Apidae	*Nomadainsignipes* Schmiedeknecht, 1882	LAU
Apidae	*Nomadaverna* Schmiedeknecht, 1882	INS; MMA
Colletidae	*Colleteshederae* Schmidt & Westrich, 1993	APP
Colletidae	*Colletesmarginatus* Smith, 1846	LAU
Colletidae	*Hylaeuscornutus* Curtis, 1831	LAU
Colletidae	*Hylaeusdifformis* (Eversmann, 1852)	INS; VAN
Colletidae	*Hylaeushyalinatus* Smith, 1842	APP
Colletidae	*Hylaeuspunctulatssimus* Smith, 1842	LAU
Colletidae	*Hylaeusstyriacus* Förster, 1871	ACQ; TDM
Colletidae	*Hylaeustyrolensis* Förster, 1871	ACQ; APP; MMA; TDM; VDC
Halictidae	*Lasioglossumcrassepunctatum* (Blüthgen, 1923)	TDM
Halictidae	*Lasioglossummedinai* (Vachal, 1895)	ACQ; APP; INS; MMA; TDM; VAN; VDC
Halictidae	*Lasioglossumperclavipes* (Blüthgen, 1934)	APP
Halictidae	*Lasioglossumzonulum* (Smith, 1848)	INS
Megachilidae	*Coelioxysemarginatus* Förster, 1853	VDC
Megachilidae	*Hoplitistridentata* (Dufour & Perris, 1840)	APP
Megachilidae	*Megachileflabellipes* Pérez, 1895	VDC
Megachilidae	*Megachileoctosignata* Nylander, 1852	APP
Megachilidae	*Megachilemarginata* Smith, 1853	INS
Megachilidae	*Osmiacephalotes* Morawitz, 1870	INS
Megachilidae	*Osmiamelanogaster* Spinola, 1808	LAU
Megachilidae	*Pseudoanthidiummelanurum* (Klug, 1832)	LAU; VDC

## References

[B11882440] Aguilera Guillermo, Ekroos Johan, Persson Anna S., Pettersson Lars B., Öckinger Erik (2018). Intensive management reduces butterfly diversity over time in urban green spaces. Urban Ecosystems.

[B12211004] Amiet F., Müller A., Neumeyer R. (1999). Fauna Helvetica 4, Apidae 2 (Colletes, Dufourea, Hylaeus, Nomia, Nomioides, Rhophitoides, Rhopites, Sphecodes, Systropha). Schweizerische Entomologische Gesellschaft.

[B12204061] Amiet F., Herrmann M., Müller A., Neumeyer R. (2001). Fauna Helvetica 6. Apidae 3: Halictus, Lasioglossum.

[B12204050] Amiet F., Herrmann M., Müller A., Neumeyer R. (2004). Fauna Helvetica 9. Apidae 4: Anthidium, Chelostoma, Coelioxys, Dioxys, Heriades, Lithurgus, Megachile, Osmia, Stelis.

[B12210835] Amiet F., Herrmann M., Müller A., Neumeyer R. (2007). Fauna Helvetica 20, Apidae 5. Schweizerische Entomologische Gesellschaft.

[B11884448] Angold P. G., Sadler J. P., Hill M. O., Pullin A., Rushton S., Austin K., Small E., Wood B., Wadsworth R., Sanderson R., Thompson K. (2006). Biodiversity in urban habitat patches. Science of The Total Environment.

[B12203788] Ascher J. S., Pickering J. Discover Life bee species guide and world checklist (Hymenoptera: Apoidea: Anthophila). https://www.discoverlife.org/mp/20q?guide=Apoidea_species&flags=HAS:.

[B11882468] Baldock Katherine C. R., Goddard Mark A., Hicks Damien M., Kunin William E., Mitschunas Nadine, Osgathorpe Lynne M., Potts Simon G., Robertson Kirsty M., Scott Anna V., Stone Graham N., Vaughan Ian P., Memmott Jane (2015). Where is the UK's pollinator biodiversity? The importance of urban areas for flower-visiting insects. Proceedings of the Royal Society B: Biological Sciences.

[B11882557] Banaszak-Cibicka Weronika, Ratyńska Halina, Dylewski Łukasz (2016). Features of urban green space favourable for large and diverse bee populations (Hymenoptera: Apoidea: Apiformes). Urban Forestry & Urban Greening.

[B11884371] Chick Lacy D., Strickler Stephanie A., Perez Abe, Martin Ryan A., Diamond Sarah E. (2019). Urban heat islands advance the timing of reproduction in a social insect. Journal of Thermal Biology.

[B11882604] Ciach Michał, Fröhlich Arkadiusz (2019). Ungulates in the city: light pollution and open habitats predict the probability of roe deer occurring in an urban environment. Urban Ecosystems.

[B11890614] Comba Livio, Comba Mario (1991). Catalogo degli Apoidei Laziali (Hymenoptera, Aculeata). Fragmenta Entomologica.

[B11920260] Comba L, Comba M, Zapparoli M (1997). Gli Insetti di Roma..

[B11884569] Comba Mario Hymenoptera Apoidea: Anthophila of Italy. Bibliographic checklist of Italian wild bees with notes on taxonomy, biology, and distribution. https://digilander.libero.it/mario.comba/.

[B11890011] Connor Edward F., Hafernik John, Levy Jacqueline, Lee Moore Vicki, Rickman Jancy K. (2002). Insect conservation in an urban biodiversity hotspot: The San Francisco Bay Area. Journal of Insect Conservation.

[B11884822] Cornalba Maurizio, Quaranta Marino, Selis Marco, Flaminio Simone, Gamba Sirio, Mei Maurizio, Bonifacino Marco, Cappellari Andree, Catania Roberto, Niolu Pietro, Tempesti Stefano, Biella Paolo (2024). Exploring the hidden riches: Recent remarkable faunistic records and range extensions in the bee fauna of Italy (Hymenoptera, Apoidea, Anthophila). Biodiversity Data Journal.

[B12210946] Dathe Holger H. (1980). Die Hylaeus-Arten einer apidologischen Sammelreise in den Iran (Hymenoptera, Apoidea). 5. Mai 1980.

[B11889231] Istat Demo https://demo.istat.it/.

[B11882336] Díaz Sandra, Fargione Joseph, Chapin F. Stuart, Tilman David (2006). Biodiversity loss threatens human well-being. PLOS Biology.

[B11904285] Di Pietro Stefano, Mantoni Cristina, Fattorini Simone (2021). Influence of urbanization on the avian species-area relationship: insights from the breeding birds of Rome. Urban Ecosystems.

[B11882566] Dyderski Marcin K., Wrońska-Pilarek Dorota, Jagodziński Andrzej M. (2016). Ecological lands for conservation of vascular plant diversity in the urban environment. Urban Ecosystems.

[B11882621] Dylewski Łukasz, Maćkowiak Łukasz, Banaszak‐Cibicka Weronika (2019). Are all urban green spaces a favourable habitat for pollinator communities? Bees, butterflies and hoverflies in different urban green areas. Ecological Entomology.

[B11885065] Fattorini Simone (2011). Insect rarity, extinction and conservation in urban Rome (Italy): a 120-year-long study of tenebrionid beetles. Insect Conservation and Diversity.

[B12134278] Fattorini Simone, Galassi Diana M. P. (2016). Role of urban green spaces for saproxylic beetle conservation: a case study of tenebrionids in Rome, Italy. Journal of Insect Conservation.

[B11883140] Fattorini Simone, Mantoni Cristina, De Simoni Livia, Galassi Diana M. P. (2017). Island biogeography of insect conservation in urban green spaces. Environmental Conservation.

[B11883184] Fattorini Simone, Mantoni Cristina, Bergamaschi Davide, Fortini Lorenzo, Sánchez Francisco J., Di Biase Letizia, Di Giulio Andrea (2020). Activity density of carabid beetles along an urbanisation gradient. Acta Zoologica Academiae Scientiarum Hungaricae.

[B11882485] Fenoglio María Silvina, Calviño Ana, González Ezequiel, Salvo Adriana, Videla Martin (2021). Urbanisation drivers and underlying mechanisms of terrestrial insect diversity loss in cities. Ecological Entomology.

[B11884430] Forman Richard T. T. (1995). Some general principles of landscape and regional ecology. Landscape Ecology.

[B11883894] Francoeur Xavier W., Dagenais Danielle, Paquette Alain, Dupras Jérôme, Messier Christian (2021). Complexifying the urban lawn improves heat mitigation and arthropod biodiversity. Urban Forestry & Urban Greening.

[B12134163] Fusco Tommaso, Fattorini Simone, Fortini Lorenzo, Ruzzier Enrico, Di Giulio Andrea (2024). Ground spiders (Chelicerata, Araneae) of an urban green space: intensive sampling in a protected area of Rome (Italy) reveals a high diversity and new records to the Italian territory. Biodiversity Data Journal.

[B11885654] Geppert Costanza, Cappellari Andree, Corcos Daria, Caruso Valerio, Cerretti Pierfilippo, Mei Maurizio, Marini Lorenzo (2022). Temperature and not landscape composition shapes wild bee communities in an urban environment. Insect Conservation and Diversity.

[B11886031] Ghisbain G., Rosa P., Bogusch P., Flaminio S., Divelec R. L., Dorchin A., Kasparek M., Kuhlmann M., Litman J., Mignot M., Müller A., Praz C., Radchenko V. G., Rasmont P., Risch S., Roberts S. P.M., Smit J., Wood Thomas J., Michenez D., Reverté S. (2023). The new annotated checklist of the wild bees of Europe (Hymenoptera: Anthophila). Zootaxa.

[B11882575] Hall Damon M., Camilo Gerardo R., Tonietto Rebecca K., Ollerton Jeff, Ahrné Karin, Arduser Mike, Ascher John S., Baldock Katherine C. R., Fowler Robert, Frankie Gordon, Goulson Dave, Gunnarsson Bengt, Hanley Mick E., Jackson Janet I., Langellotto Gail, Lowenstein David, Minor Emily S., Philpott Stacy M., Potts Simon G., Sirohi Muzafar H., Spevak Edward M., Stone Graham N., Threlfall Caragh G. (2017). The city as a refuge for insect pollinators. Conservation Biology.

[B11889806] Iamonico Duilio (2022). Biodiversity in urban areas: The extraordinary case of Appia Antica Regional Park (Rome, Italy). Plants.

[B11884466] Jones Elizabeth L., Leather Simon R. (2012). Invertebrates in urban areas: A review. European Journal of Entomology.

[B11906731] Kuttler W, Marzluff John M., Bradley Gordon, Ryan Clare, Shulenberger Eric, Endlicher Wilfried, Alberti Marina, Simon Ute, ZumBrunnen Craig (2008). Urban Ecology.

[B12206194] Lhomme Patrick, Michez Denis, Christmann Stefanie, Scheuchl Erwin, Abdouni Insafe El, Hamroud Laila, Ihsane Oumayma, Sentil Ahlam, Smaili Moulay Chrif, Schwarz Maximilian, Dathe Holger H, Straka Jakub, Pauly Alain, Schmid-Egger Christian, Patiny Sebastien, Terzo Michael, MÜller Andreas, Praz Christophe, Risch Stephan, Kasparek Max, Kuhlmann Michael, Wood Thomas J, Bogusch Petr, Ascher John S, Rasmont Pierre (2020). The wild bees (Hymenoptera: Apoidea) of Morocco.. Zootaxa.

[B11882732] Magura Tibor, Horváth Roland, Tóthmérész Béla (2010). Effects of urbanization on ground-dwelling spiders in forest patches, in Hungary. Landscape Ecology.

[B11875141] Matteson KC., Langellotto GA. (2011). Small scale additions of native plants fail to increase beneficial insect richness in urban gardens. Insect Conservation and Diversity.

[B11882288] McKinney Michael L. (2008). Effects of urbanization on species richness: A review of plants and animals. Urban Ecosystems.

[B12211066] Michez Denis, Rasmont Pierre, Terzo Michaël, Vereecken J. Nicolas (2019). Hymenoptera of Europe 1. Bees of Europe.

[B11882752] Moorhead Leigh C., Philpott Stacy M. (2013). Richness and composition of spiders in urban green spaces in Toledo, Ohio. The Journal of Arachnology.

[B11893576] Neumüller Ulrich, Burger Hannah, Krausch Sabrina, Blüthgen Nico, Ayasse Manfred (2020). Interactions of local habitat type, landscape composition and flower availability moderate wild bee communities. Landscape Ecology.

[B11882548] Nielsen Anders Busse, van den Bosch Matilda, Maruthaveeran Sreetheran, van den Bosch Cecil Konijnendijk (2013). Species richness in urban parks and its drivers: A review of empirical evidence. Urban Ecosystems.

[B12138329] Nieto Ana, Roberts Stuart P. M., Kemp James, Rasmont Pierre, Kuhlmann Michael, Criado Mariana García, Biesmeijer Jacobus C., Bogusch Petr, Dathe Holger H., De la Rúa Pilar, De Meulemeester Thibaut, Dehon Manuel, Dewulf Alexandre, Ortiz-Sánchez Francisco Javier, Lhomme Patrick, Pauly Alain, Potts Simon G., Praz Christophe, Quaranta Marino, Radchenko Vladimir G., Scheuchl Erwin, Smit Jan, Straka Jakub, Terzo Michael, Tomozii Bogdan, Window Jemma, Michez Denis (2014). European Red List of bees. Publication Office of the European Union.

[B11882525] Öckinger Erik, Dannestam Åse, Smith Henrik G. (2009). The importance of fragmentation and habitat quality of urban grasslands for butterfly diversity. Landscape and Urban Planning.

[B11884319] Oke T. R. (1973). City size and the urban heat island. Atmospheric Environment (1967).

[B11886674] Orr Michael C., Hughes Alice C., Chesters Douglas, Pickering John, Zhu Chao-Dong, Ascher John S. (2021). Global patterns and drivers of bee distribution. Current Biology.

[B12205209] Orr Michael C., Chesters Douglas, Williams Paul H., Wood Thomas J., Zhou Qingsong, Bossert Silas, Sless Trevor, Warrit Natapot, Rasmont Pierre, Ghisbain Guillaume, Boustani Mira, Luo A’rong, Feng Yuan, Niu Ze-Qing, Zhu Chao-Dong (2024). Integrative taxonomy of a new species of a bumble bee-mimicking brood parasitic bee, Tetralonioidella mimetica (Hymenoptera, Apoidea, Apidae), investigated through phylogenomics. Journal of Hymenoptera Research.

[B12204042] Pauly A. (2015). Clés Illustrées Pour L’identification des Abeilles de Belgique et des Régions Limitrophes (Hymenoptera: Apoidae) I. Halictidae. Document de travail du projet BELBEES..

[B11889714] Quaranta M, Cornalba M, Biella P, Comba M, Battistoni A, Rondinini C, Teofili C (2018). Lista Rossa IUCN delle api italiane minacciate. https://www.iucn.it/pdf/Comitato_IUCN_Lista_Rossa_delle_Api_italiane_minacciate.pdf.

[B11893565] Schindler Matthias, Diestelhorst Olaf, Haertel Stephan, Saure Christoph, Scharnowski Arno, Schwenninger Hans. R. (2013). Monitoring agricultural ecosystems by using wild bees as environmental indicators. BioRisk.

[B12211030] Smith Jan (2018). Identification key to the European species of the bee genus *Nomada* Scopoli, 1770 (Hymenoptera: Apidae), including 23 new species. Entomofauna, Zeitschrift für Entomologie.

[B11875597] Vanbergen Adam J, the Insect Pollinators Initiative (2013). Threats to an ecosystem service: pressures on pollinators. Frontiers in Ecology and the Environment.

[B11883491] Wenzel Arne, Grass Ingo, Belavadi Vasuki V., Tscharntke Teja (2020). How urbanization is driving pollinator diversity and pollination – A systematic review. Biological Conservation.

[B11893528] Westphal Catrin, Bommarco Riccardo, Carré Gabriel, Lamborn Ellen, Morison Nicolas, Petanidou Theodora, Potts Simon G., Roberts Stuart P. M., Szentgyörgyi Hajnalka, Tscheulin Thomas, Vaissière Bernard E., Woyciechowski Michal, Biesmeijer Jacobus C., Kunin William E., Settele Josef, Steffan-Dewenter Ingolf (2008). Measuring bee diversity in different European habitats and biogeographical region. Ecological Monographs.

[B12204069] Wood Thomas James (2023). The genus *Andrena* in Belgium: revisions, clarifications, and a key for their identification (Hymenoptera: Andrenidae). Belgian Journal of Entomology.

[B11884752] Wood Thomas James, Praz Christophe, Selis Marco, Flaminio Simone, Mei Maurizio, Cornalba Maurizio, Rosa Paolo, Le Divelec Romain, Michez Denis (2023). Revisions to the *Andrena* fauna of Italy, with the description of a new species (Hymenoptera: Andrenidae). Fragmenta Entomologica.

[B12205200] Wood T. J. (2024). Further revisions to the Palaearctic Andrena fauna (Hymenoptera: Andrenidae). Zootaxa.

[B11889836] Zaninotto Vincent, Thebault Elisa, Dajoz Isabelle (2023). Native and exotic plants play different roles in urban pollination networks across seasons. Oecologia.

